# A systematic review on patient and public attitudes toward health monitoring technologies across countries

**DOI:** 10.1038/s41746-025-01762-4

**Published:** 2025-07-12

**Authors:** Tiantian Chen, Ekaterina Hertog, Adam Mahdi, Samantha Vanderslott

**Affiliations:** 1 Global Health 50/50, Cambridge, UK; 2 Oxford Internet Institute, University of Oxford, Oxford, UK; 3 Institute for Ethics in AI, University of Oxford, Oxford, UK; 4 Pandemic Sciences Institute, University of Oxford, Oxford, UK

**Keywords:** Sociology, Public health, Quality of life

## Abstract

The market for digital health monitoring is expanding rapidly, with technologies that track health information and provide access to medical data promising benefits for users, particularly in areas with limited healthcare resources. To understand user attitudes toward these technologies, we conducted a systematic review of literature with primary data about patient and public perspectives. We synthesized 562 studies (2000–2023) from PubMed, Embase, ACM Digital Library, IEEE Xplore, Web of Science, and Scopus, including qualitative, quantitative, and mixed-methods research. We revealed a significant geographic bias, with most research concentrated in few countries, and identified access gaps in both Global South and Global North. While users generally showed positive attitudes toward health monitoring technologies, they expressed various concerns. We provide suggestions for future research to enhance the socially responsible integration of technology in healthcare. One important limitation of our approach is using English-language search terms. This potentially excluded relevant studies from underrepresented countries.

## Introduction

Health monitoring technologies are increasingly adopted in healthcare, a trend accelerated during the COVID-19 pandemic for more digital and remote modes of working fueled by the need to reduce virus exposure among patients and healthcare professionals^1^. Techno-optimists who place strong faith in technological progress believe these technologies will improve the quality of data available to doctors, reduce the cost while increasing the efficiency of monitoring patients and recording their details, ultimately benefiting patients^2^. By removing geographical health barriers, digital health monitoring is seen as a way to improve healthcare access, especially in areas with limited healthcare resources, provided that the necessary digital infrastructure exists.

Such benefits can only become reality if all the users are equally able and willing to adopt digital health monitoring effectively. This, however, cannot be taken for granted. The distribution and practices of health monitoring technologies predominantly assume users are white, well-paid, educated, cisgender, with good internet access, capable of managing their own healthcare, and live in high-income countries^3^. This leaves out many groups, such as carers who are not regarded as users in many studies and ethnic minorities whose needs are often neglected in technology development.

Existing systematic reviews focus narrowly on specific users and technologies such as older adults’ attitudes toward smart home technologies^4^ and users’ experience of wearable technologies (“wearables”)^5^. This narrow focus makes reviews easier to conduct but limits understanding of how attitudes toward various monitoring technologies vary across different socioeconomic backgrounds and geographies. Understanding how the under-resourced communities and less advantaged individuals who potentially stand to benefit the most from such technologies, compare with more advantaged individuals is crucial if health monitoring technologies are to bring about greater equality in healthcare outcomes. Our systematic review assesses and synthesizes existing literature on patient and public attitudes toward health monitoring technologies, with a focus on identifying key factors that influence adoption across different socioeconomic and geographic groups. By summarizing the current knowledge on broadly defined health monitoring technologies, highlighting the gaps in the literature, and proposing directions for future research, our review seeks to provide insights into more equitable and public/patient-centered implementation of digital health monitoring.

## Results

### Characteristics of included studies

562 studies were included in this review. All the included studies were published between 2004 and 2023, with publications reaching their highest frequency in 2021—coinciding with the increased adoption of remote health monitoring during the COVID-19 pandemic^6^. Our sample included 240 quantitative studies, 201 qualitative studies, and 121 mixed methods studies.

**Fig. 1:**
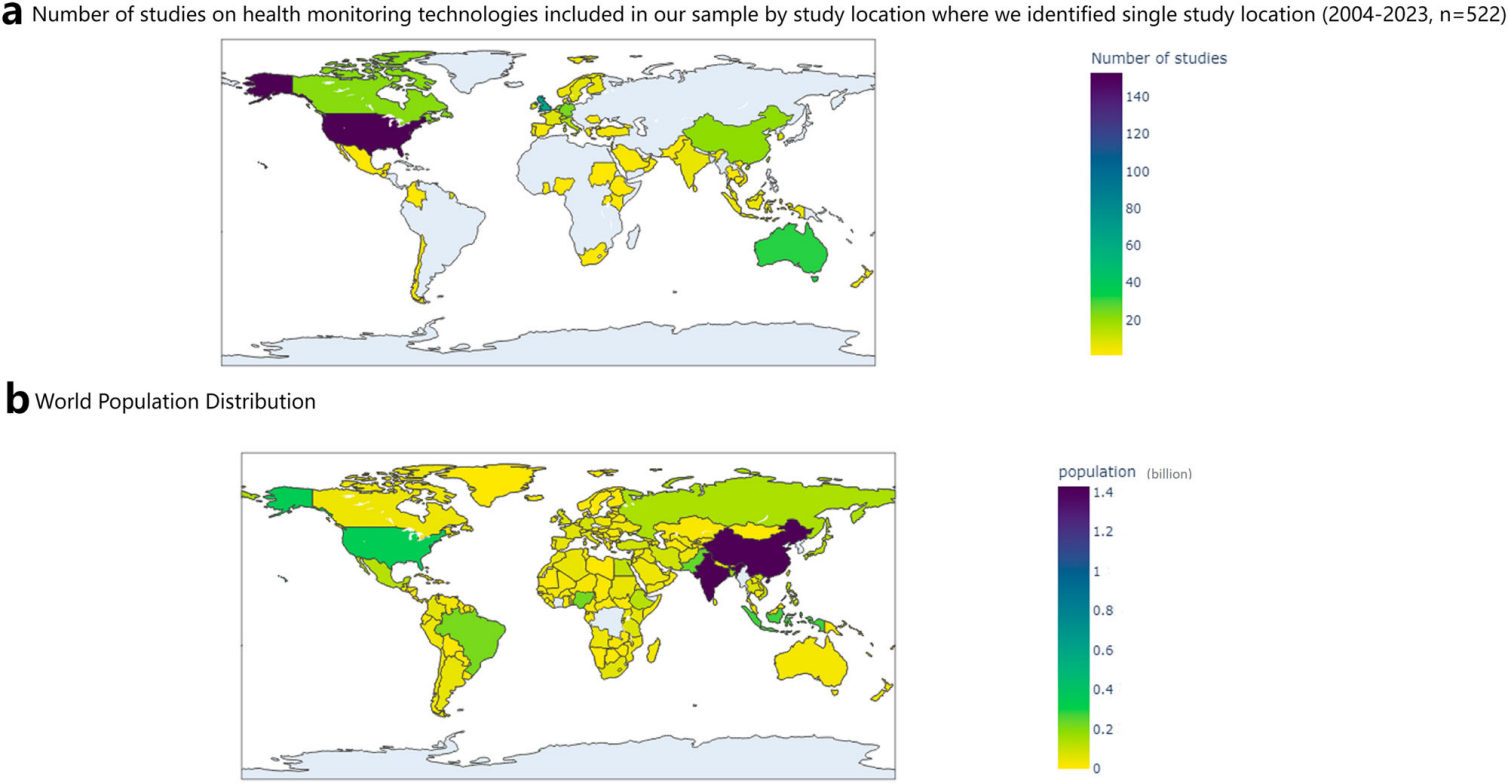
Number of studies on patient and public attitudes to health monitoring technologies vs world population distribution. The maps illustrate that highly studied regions have smaller populations, whereas densely populated areas are underrepresented in studies about health monitoring technologies. **a** Number of studies on health monitoring technologies included in our sample by study location where we identified single study location (2004–2023, *n* = 522). **b** World Population Distribution. We use the population data from World Population Prospect 2024. See https://population.un.org/wpp/ for more information.

In our sample, 522 studies specified a single study location (16 articles did not specify the study location, and 24 studies included more than one study location. We excluded these articles in the analysis of geography to avoid confusion of numbers). Four countries accounted for over half of these single-location studies: the United States (153), the United Kingdom (67), Australia (32), and Germany (24) (see Fig. 1). Of the 546 studies that mentioned any location, 444 were conducted in the Global North. The Global South, particularly low-income countries, was severely underrepresented. Only five studies were conducted in low-income countries: Ethiopia, Sudan, and Uganda.

Among 562 studies, 420 reviewed medical applications of health monitoring technologies, with diabetes (54), cardiology (39), and psychiatry (37) being the most frequently represented specialties. Smart home systems for older adults (71), activity trackers (36), and workplace well-being monitoring (11) are the most-mentioned non-medical applications in our sample. Studies in Global North covered more specialties than those in Global South. No studies on technologies for cancer treatment, smoking cessation, and alcohol addiction used samples from Global South, and only one study on respiratory diseases used a sample from Global South.

Our ability to compare findings across demographic groups was constrained by limitations of the demographic data available in most studies in our sample. While most studies reported basic information like gender and age (see Table 1), few captured other key demographics such as ethnicity, education level, or place of residence. In analyzing sampling methods, we distinguished between convenience sampling (nonprobability recruitment) and representative sampling (probability-based recruitment or a study mention being based on a representative sample). Although convenience sampling and smaller sample sizes (fewer than 50 participants) are standard in qualitative research, caution is warranted when applying statistical analysis to such samples. Among quantitative studies, only 47 (19.5%) used representative samples, and 78 (35.6%) had fewer than 50 participants. We also evaluated inclusion of economically disadvantaged, educationally disadvantaged, rural, or gender minority populations and noted a representation gap. For example, only 34 of the 122 studies on fitness trackers recruited participants from disadvantaged socio-economic backgrounds.

**Table 1:** Number of articles reporting respondents’ gender, age, ethnicity, education, and rural/urban residence (2004–2023, *n* = 562)

Demographic characteristics	Number of studies mentioning (percentage)
Sex/gender	447 (79.5%) (9 articles specified sexual orientation)
Age	464 (82.6%)
Ethnicity	114 (20.3%)
Educational background	154 (27.4%)
Rural/urban residence/distance to hospitals	21 (3.7%)
Income level	106 (18.9%)

Among 562 studies, 504 focused on primary users (patients and public), five on secondary users (paid carers, parents, and family members), and 53 on both; the most commonly studied technology types were wearables, apps, and smart home systems.18 studies featured artificial intelligence (AI) technologies, with 15 in Global North and three in Global South. Notably, all studies from Global South originated in China.

### Thematic analysis

We took an inductive approach to identify codes and group them into six themes reflecting public and patient perceptions of monitoring technologies: prior knowledge of monitoring technology, acceptability, usability, motivations and challenges, perceived benefits, and perceived risks. The themes introduced below are ordered in the way individuals engage with health monitoring technologies, from initial awareness to ongoing evaluation of benefits and risks. Due to the large number of studies in this review, we provide typical examples to illustrate these themes. Table 2 summarizes the key findings. We also present how codes and sub-codes were developed into the findings in Table 2. The full list of themes as well as explanation of codes and sub-codes is available in Supplementary Table 6.

**Table 2:** Summary of the key findings (2004–2023, *n* = 562)

Theme		Meaning	Codes and sub-codes	Number of articles with this theme	Number of studies based in Global North	Key findings about the theme
Knowledge	Awareness and familiarity	Prior knowledge about health monitoring technologies	Familiar, unfamiliar	59	44	62.7% studies reported unfamiliarity, while 37.3% studies reported familiarity The findings on the relationship between health risks and knowledge about technologies are contradictory. For example, a focus group study with 17 participants found patients’ anxiety about health issues motivated them to familiarize themselves with activity monitors^15^. At the same time, a community-based population survey with 317 respondents found that hypertension was not correlated with their use of wearable blood pressure technology^16^.
	Determinants of knowledge		Socio-demographic factors, perceived usefulness	6	6	
Acceptability	Acceptance	Acceptance of technologies	Mixed, low interest/negative, positive	294	233	88.1% found positive attitudes to technologies, 8.8% found negative or low interest in them, and 3.1% found mixed Some found a correlation between respondents’ socio-economic background characteristics and acceptability, but others did not.
	Determinants of acceptance		Correlation or no correlation with socio-demographic factors, self-control, appearance, external factors, technology difference, desire, location, knowledge, relationship with others, perceived ease of use, perceived usefulness, security	136	100	
Usability	User experience	User experience of technologies	Ease of use, comfortable, appealing appearance, durability, language, self-application, technical difficulties	335	141	Of 270 articles examining perceived ease of use, 214 found users regarded technologies easy to use. Of 99 articles examining perceived comfort, 83 reported comfortable to use. Of 90 studies about opinions on device appearance, 39 studies found devices visually appealing. Perceived usability problems varied with respondents’ socio-demographic characteristics. For example, interviews with 20 pregnant women in their second trimester found a finger ring to monitor sleep/stress data might be too big for women^45^. Interviews with seven patients found old people felt difficult to seal an oxygen device^46^.
	Improvement of usability		Information storage, eliminate Ads, offline activity, more inclusive, Context of use, justification, comfort, wording, loss, unobtrusiveness, functionality, location, gamification, tailored feedback, data interpretation, appearance features	231	197	
Motivations and barriers	Motivations	Factors motivating or preventing informants from using technologies	Perceived usefulness, perceived ease of use, social relationship, competition, curiosity, anxiety, pleasure, fun/coolness, brand, external environment, giving back	163	136	Anxiety about health risks and perceived usefulness to mitigate risks were major motivations for use of tracking devices.
	Barriers		Unfit body shape, not inclusive, usability issue, no correlation, not convinced, the foreign body, lack of autonomy, lack of collaboration, knowledge, forget, incompatible with other devices, access issues, cost (time, money, etc), functionality, interfere current life, replace existing care, no need, embarrassment, data sharing concern, distrust	187	153	Affordability is a direct barrier preventing people from using monitoring technologies. Certain groups are digitally excluded. For example, interviews and workshops with the queer community indicate that gender options for wearables lack queer option^59^. Another focus group study found age prevents the elderly from using wireless healthcare sensors independently^27^.
	How to motivate		Compatibility, reduce cost, award/stimulus, promote understanding, one-to-one basis/build relationship	34	28	The most frequently mentioned incentive for technology adoption is a trusting relationship between manufacturers and potential users.
Perceived benefits		Perceived advantages of technologies	Technology development, individualized treatment, contribute to research/social good, promote policy/infrastructure, improve privacy, supplement/replace routine test, security, good relationship, legitimize their symptoms, behavior change, awareness, reduction burden, improving their adherence, support, improve confidence/accomplishment, self-control	284	250	Increasing health consciousness, changing behaviors, and improving social relationships are three major perceived benefits.
Perceived risks		Concerns about technologies	Impact professionalism, dehumanizing care, decrease opportunity, bias Accuracy, surveillance, burden, reduced autonomy, adverse events, privacy, malfunction	213	194	Three major concerns are privacy, accuracy, and feeling burdened. Certain groups were more concerned about risks of monitoring technologies. For example, a survey of 241 women using fertility trackers found women trying to conceive were the most skeptical about the accuracy of fertility apps^69^ Research also indicates that women and those with university education were more perceptive to privacy risks^92^.

Of the 59 articles on patients’ or general public’s prior *knowledge of monitoring technologies*, 37 found most respondents were unfamiliar with the technology under scrutiny, while 22 found most respondents reported familiarity. Six studies analyzed factors influencing knowledge about technologies, four revealing correlations with participants’ socio-economic characteristics like age and gender. For instance, a study found that patients over 65, who could benefit most from tablet monitoring, were least familiar with the technology^7^. The findings on the associations between health risks and technology knowledge are inconsistent. One focus group study with 17 older patients found anxiety about health issues increased familiarity with activity monitors^8^, while a survey with 317 patients found no correlation between known hypertension and knowledge about wearable blood pressure technology^9^.

Of 294 articles studying *acceptability* of health monitoring technologies, 259 found positive attitudes to technologies, 26 found negative or low interest in them, and 9 documented mixed attitudes. 56 out of the 294 studies containing this theme used samples from Global South and virtually all of these studies recorded positive attitudes. All but 31 studies documenting negative or mixed attitudes used samples from Global North^10^.

136 articles analyzed factors influencing acceptability. 65 found correlations with socio-economic characteristics, showing that women, educated respondents, and high earners were more receptive to such technologies^11,12^. Six studies found no such correlation. For example, a surveyed sample of 60 senior citizens found age did not influence their acceptance of Ambient Intelligence, an app monitoring home environment^13^. Remaining papers did not focus on socio-economic characteristics but rather considered other factors. Perceived technology usefulness and ease of use were uniformly positively related to acceptability in 40 and 20 papers respectively^14,15^. 17 studies associated awareness of risks with increased acceptability^16^, while 15 found no link between prior technology knowledge and acceptability^17^. Two studies linked respondents’ personality traits with technology adoption. For example, interviews with 22 Parkinson’s patients found that those with a positive outlook were more likely to wear sensors^18^. 16 studies found fewer social relations increased acceptability. For example, research has indicated that those with pets^19^ or with good social bonds^20^ were less likely to accept monitoring technologies. Other influential external factors included usage scenario^21^ and device appearance^22^. For example, a test with 20 dementia patients revealed they were less satisfied with wearable GPS devices at home^20^.

Of 270 articles examining perceived *usability*, in 214 studies, most users found the technologies tested easy to use. For some patients and elderly people, ease of use meant independence in applying devices, which was highly valued by both carers and patients, especially after activities like showering^23,24^. Usability complaints included complex operation manuals, data loss, connection failures, false alarms, lack of waterproofing, susceptibility to damage, excessive advertisements, and short battery life^25,26^. Suggestions for improvement included clear data interpretation, visualization, and comparison features^27,28^. Some patients required manufacturer to justify the usefulness of function, as indicated in interviews from kidney transplant patients^29^. Some users requested less paternalistic language^30^ and proposed gamifying devices to increase engagement^31^. Some respondents requested offline activities, such as a dementia education workshop for carers of dementia patients to supplement wearable devices^32^.

Regarding comfort, 83 out of 99 studies reported that monitoring technologies were comfortable to use, with suggestions to secure device attachment and reduce skin irritation^29^. In terms of device appearance, 51 of 90 studies noted issues with size, color, and style^33^, while 39 found devices visually appealing^34^. Users preferred non-obtrusive designs, as seen with diabetes patients who concealed glucose monitoring devices under clothing^35^. Some devices were criticized for medicalizing or stigmatizing users, which caused social pressure, such as children concealing bedwetting alarms^36^.

50 studies revealed that perceived usability problems varied with respondents’ socio-demographic characteristics, calling for tailored improvements. For instance, interviews with pregnant women found a sleep/stress monitoring ring too large^37^, and interviews with older patients found they struggled with sealing oxygen devices^38^. Individual health conditions also influenced preferences: elderly individuals with poor eyesight valued visible fall detectors^21^, brain injury patients favored voice recognition for health diaries^27^, and migraine sufferers preferred dim screens^39^. Some comparative studies indicated potential cultural differences in user evaluations; for example, one study found Arab users were more likely to focus on physiological measurements of fitness trackers compared to non-Arab users who focused more on goal achievement^10^.

163 studies examined *motivations* of using health monitoring technologies. Among them, 129 studies identified anxiety over health risks and the perceived usefulness of tracking devices to mitigate these risks as key motivations for technology adoption^13,35^. For instance, patients with peripheral arterial disease used disease detectors due to concerns about limb loss^35^. Other intrinsic motivations included being able to share achievements with peers^40^ and curiosity about personal health metrics^41^. Social influences were significant extrinsic motivators, with recommendations from family members^25^ or healthcare providers driving adoption^38^. Peer competition also spurred usage, particularly in fitness tracking^25^. Brand appeal^41^ and contributing to scientific research^42^ were additional factors.

Of 187 articles identifying *challenges* to using monitoring technologies, affordability was a major challenge in 72 studies. For example, older adults in Ireland reported that inadequate insurance coverage prevented them from purchasing activity trackers^43^. Lack of interest was noted in 58 studies, often due to unawareness of health risks^40^ or skepticism about technology benefits^34^. For example, carers of adults with developmental disabilities refused a smart home system for lacking essential human interaction functionality^44^. Users also lost interest if they were the only person in the community to use technologies^43^ or if they felt uncomfortable with being watched^45^. With lack of interest, people tended to forget to use monitoring technology^46^. User skills and knowledge gaps were highlighted in 56 articles, with users finding learning new devices burdensome and disruptive to daily routines^18^.

Compatibility issues with current mobile phones or hospital devices^47^, lack of preferred functions^34^, and technical usability concerns like discomfort or poor fit^35^ were also reported challenges. For example, some elderly users found that hip protectors for detecting falls did not fit with other assistive equipment or failed to work when falls occurred onto knees^32^. Additionally, discomfort with implanted monitors was likened to feeling foreign to the body^48^. Regulations also posed challenges. For instance, school mobile phone policies hindered students’ use of glucose monitors^49^, and lack of government endorsement of AI significantly hindered the widespread adoption of AI-enabled wearable medical devices in China^50^.

Furthermore, certain populations are digitally excluded. For example, interviews with the queer community indicated that gender options for wearables are binary^51^. In a focus group study, elderly people reported difficulty understanding health monitors^20^. Trusting relationships between manufacturers and users, fostered by communication efforts such as providing user support, emerged as critical for technology adoption^52^. For example, one qualitative study found community education facilitated acceptance of bednet use monitors in Uganda^53^. Family members^54^ and doctors^55^ also played an essential role in facilitating adoption. Other suggested strategies to enhance adoption included improving device compatibility, offering monetary incentives like cost reduction or insurance reimbursement^55^, and non-monetary incentives like positive verbal reinforcement praising users for using monitoring products, gamification of medicare, and peer competition^56^.

Among 284 studies on *perceived benefits*, three major advantages were increased health consciousness (146 studies), behavior change (49), and improved social relationships (29). For example, users reported they could track effects of medications^27^, stages of disease^57^, and body functions such as basal body temperature and cervical mucus^58^. They increased activity levels^33^, stopped smoking^46^, and took medicines more punctually^59^. Users also reported monitoring technologies facilitated greater interaction with healthcare professionals, particularly during COVID-19^60,61^.

Additionally, 24 studies found users felt supported and empowered by these technologies. For example, contact tracing apps in the US during the pandemic gave users hope that their life would soon be normalized^62^. Users also reported increased confidence through greater control over their lives. With smart home systems detecting falls, for example, elderly people felt they could live independently^14^, and their family members felt relieved of caregiver burden^63^. Both patients^30^ and caregivers^63^ reported improved healthcare efficiency, such as reduced hospital visits with monitoring technologies. Moreover, they reported satisfaction with clinical experience due to personalized care^64^, better data storage^65^^,^ and accurate treatment^66^. For example, a qualitative study found patients with depression felt gamified data collection more enjoyable^67^. Other reported benefits included enhanced treatment methods, research advancements, and digital infrastructure development in rural areas^68,69^.

213 studies mentioned *perceived risks*. Notably, some benefits of monitoring technologies were also perceived as risks. Privacy was a major concern in 131 studies, with worries about non-transparent data collection and misuse of personal data by caregivers, manufacturers, insurance companies, or criminals^70,71^. In 58 studies, users experienced various types of burdens with monitoring technologies. First, they mentioned data obsession, where over-reliance on technology may undermine autonomy^67^ or result in over-treatment^72^. For example, patients with depression in focus groups reported sleep loss from constant mood tracking^66^. Second, caregivers felt overwhelmed by the additional work of reviewing monitor results, especially during COVID-19^73^. Third, users were worried about bias and stigmas produced by health monitoring. For example, a survey with 245 older adults showed they felt sad about their age when using contactless monitoring^54^. People were also concerned about health data being used to discriminate in insurance pricing and employment^74^. 51 studies highlighted concerns about technology errors, such as inaccurate sensor data affecting health decisions^67^, lost connections^75^, device crashes^76^, and malware infections^16^.

Beyond privacy infringement, burdens, and technology errors, monitoring technologies were seen to dehumanize healthcare by reducing the direct human contact that is expected of healthcare. For instance, carers in a retirement home faced residents’ doubts about their professionalism when using mobile devices for alarms^77^. Many patients preferred face-to-face communication^70^. These technologies could also decrease communication with family^74^ and healthcare providers, as seen in a study with 19 patients where a smart health platform reduced referrals to private practices^78^. Aside from social and emotional risks, research participants noted physical risks such as skin rash^79^ and radiation from wireless technology^80^.

Socio-demographic factors influenced concerns about technology risks. For instance, a survey of 241 women found women trying to conceive were skeptical about fertility app accuracy^58^. Research also indicated women and those with higher education levels particularly worried about privacy^81^.

## Discussion

Research on health monitoring technologies has accelerated alongside their increasing adoption in medical and consumer settings. This review analyzed existing studies, examining their geographic scope, sampling methods, user demographics, and types of technologies deployed. We synthesized current understanding of patient and public attitudes through six key themes: knowledge, acceptability, usability, motivations and challenges, perceived benefits, and perceived risks. Looking at our core themes first, we found that while many patients and members of the public were unfamiliar with existing health monitoring technologies, research documented generally positive attitudes towards them. In studies where users tested health monitoring technologies, most users found them easy to use, and patients and their carers highly valued the ability to utilize devices independently. In many studies, familiarity, acceptability, and perceived usability of monitoring technologies varied by socio-demographic characteristics. Certain populations, such as older adults^28^, or queer people^51^, felt digitally excluded. This finding highlights how the digital health divide extends beyond unequal access to technologies. Even with seemingly equal access, benefits may still not be equally distributed. To maximize the benefits of these technologies, manufacturers and policymakers need to consider groups that would most benefit, such as older adults, and eliminate barriers to adoption. Sociality influences the adoption of these technologies^19,20^. Individuals with richer social connections may be less interested in adopting these technologies initially, but family, friends, and effective communication efforts by manufacturers could promote knowledge about these technologies and encourage use.

Several properties of health monitoring technologies identified as bringing benefits to their users have also been flagged as potential sources of risk. Respondents reported behavior changes but also feared over-reliance on technology and over-treatment^27,33,46,57–59,67,72^. While some experienced more interaction with healthcare professionals, others worried about reduced communication with family and clinicians^33,72,74,77,78^. Some praised these technologies for personalized care, but there were also concerned about the misuse of personal data^64–66,70,71^. In the cases, where the same characteristics of technologies could bring benefits to some users but result in negative unintended consequences for others, the potential benefits and risks should be carefully considered before these technologies are widely adopted.

Health monitoring devices, like other digital technologies that collect personal information, raise privacy concerns. Perceived privacy risks underscore a divide between users and the companies handling their data. This review found that users feared data misuse by companies, necessitating robust policies and guidelines for inclusivity and trustworthiness^70,71^. While research indicates the need for comprehensive policies to build trust and ensure inclusive practices, regulatory approaches vary significantly across nations. For example, the US classifies monitoring technologies as either medical or non-medical devices, with medical devices regulated by the 21st Century Cures Act^82^. In contrast, Japan classifies devices by risk level, requiring approval for higher risk devices^83^. Studying contrasting regulatory models could inform the development of more effective global standards for responsible health data monitoring.

In all, our review identified several knowledge gaps. Ensuring high-quality samples is challenging and costly, leading many studies to rely on small, convenience samples that limit generalizability. Many studies provide limited demographic details for their samples, with few studies specifying participants’ ethnicity, income, education, or urban/rural locations. These practices complicate interpretation of the findings, especially when they are contradictory. For example, some studies show socio-economic factors affect technology acceptability, while others do not^11–13^. Some documented contradictions might result from different methodologies and research contexts. More details about sampling strategies in future research would inform an understanding of applicability and generalizability of research findings and to help build a fuller picture of attitudes to health monitoring in different populations and across different contexts. Future research would also benefit from open research approaches, including pre-registering designs, hypotheses, methods, and analyses to guide confirmatory tests^84^. Additionally, researchers should share research materials, anonymized data, and analysis codes to facilitate examination and reproduction of findings. These practices improve the efficiency and accuracy of science, such that the growing literature on digital monitoring technologies can ultimately provide clearer answers about where evidence is strong, and where evidence is limited, or applicable only to certain populations. Such processes are especially important in the healthcare and technology space, where hype and fear tactics surrounding technology innovation and public health crisis too often drive policy.

Virtually all the knowledge about attitudes to monitoring technologies comes from Global North, especially from Anglophone countries. People from Global South are under-represented, despite Global South being home to the majority of the world’s population. Monitoring technologies are often used very differently in Global North and Global South. To give one example, fertility apps are used to enhance pregnancy chances in Global North and to limit reproduction opportunities in Global South^85^. A few comparative studies also suggest cultural differences in attitudes to technologies^10^. This means that we cannot extrapolate findings from research based on people living in Global North to the rest of the world. Health monitoring could improve healthcare access in areas with limited resources, but current recruitment strategies under-represent those who might benefit most. Research with more diverse samples is necessary to understand how under-represented populations interact with health monitoring technologies.

In addition to identifying sampling gaps, our analysis revealed significant gaps in health monitoring technologies across both Global South and Global North regions. First, in the Global South, we identified a critical mismatch between available health technologies and actual disease burden. While these regions face a dual challenge of managing infectious diseases (like malaria and HIV/AIDS) while confronting rising rates of non-communicable diseases (such as cancer and respiratory illnesses), few studies address the latter. The ongoing epidemiological transition driven by urbanization and lifestyle changes in Global South underscores the need for technological developments tailored to these contexts^86,87^. Second, we found that AI-powered technologies like smart home systems and robots are predominantly studied in Global North (15 studies), with only three such studies emerging from Global South. This disparity reflects broader challenges in the Global South, including underdeveloped digital infrastructure, limited data models, and insufficient financial investment schemes^88^. As AI technology continues to advance, this gap risks further widening in resource-constrained regions. Third, Access challenges persist even in the Global North, where many individuals struggle with financial constraints, technological literacy, and reliable internet connectivity^22,34,43^. Current research has primarily focused on building trust to increase technology adoption^52,53^. However, there is an urgent need for studies examining how underserved communities in the Global North can overcome practical barriers, particularly regarding affordability and device compatibility.

While this review identified key aspects of the digital divide in health monitoring technologies, it has limitations, that warrant discussion. First, our predominantly English-language search methodology captured only seven non-English studies, potentially excluding crucial research from underrepresented regions and limiting our understanding of the North-South divide. Future research would benefit from incorporating non-English search terms and multilingual databases to improve inclusivity. Second, while we distinguished between primary and secondary technology users, our analysis of caregivers’ perspectives was limited. Some studies revealed caregivers’ concerns about dehumanized care, which contrasts with common assumptions about job displacement through digitization. A more thorough examination of caregivers’ viewpoints is needed in future reviews. Third, we identified complex patterns in health monitoring technology adoption. Notably, technology hesitancy and refusal were predominantly reported in Global North countries, with 31 of 35 studies describing negative or mixed attitudes originating from this region. We also found some studies identified correlation between respondents’ socio-economic characteristics and acceptability, while some did not. These patterns may be due to varying methodologies and research contexts. Given the limited number of studies to draw on, we refrained from drawing definitive conclusions. A detailed discussion of these findings is available in Supplementary Table 8 and Supplementary Note 2 due to space constraints. Further research is needed to explore and address these patterns more comprehensively. Finally, our brief analysis of conflicts of interest in studies revealed mixed impacts. While industry funding enabled research in specialized areas like tetraplegia and idiopathic scoliosis, studies with declared conflicts of interest tended to report more favorable outcomes compared to studies without such conflicts. Further research should examine how corporate influence, and financial interests affect research findings in this field.

In conclusion, we systematically reviewed patient and public attitudes toward health monitoring technologies and found that participants are generally positive about adopting these technologies as part of their care and find these technologies useful. At the same time the knowledge about health monitoring technologies is limited. There is a need to balance carefully the perceived risks and benefits of these technologies, and obstacles to adoption affect some populations more than others. Our findings particularly highlight a critical need for research to address significant knowledge gaps regarding these technologies in regions outside the Global North.

## Methods

### Search strategy and inclusion criteria

In this review, we included peer-reviewed study and pre-prints with primary data about patients’ and public attitudes toward health monitoring technology, published between 2000 and 2023, which provided enough data for thematic analysis. Where there were multiple reports of the same study (e.g., a pre-print followed by a peer-reviewed research publication), we included only the peer-reviewed publication. The early 2000s marked significant advancements in health monitoring technologies, including the development of wearables, the widespread adoption of smartphones, and progress in health data analytics, which have profoundly shaped the landscape of health monitoring^89^. This time period is crucial for understanding the rise and growth of health monitoring technologies, making it an essential focus for this review. The review’s end date is 2023, aligning with the year the search was conducted to ensure the inclusion of the most current and relevant studies. We excluded commentaries, conference abstracts, and reviews lacking detailed information to explain their conclusions. Although we did not apply language filters, our search was conducted in English, since a majority of studies were published in English^90^. Besides English studies, we found two Chinese, three French, and two Arabic studies, which we accessed using Google Translator.

We defined health monitoring technologies as digital products that collect human health-related data without direct intervention of healthcare providers, therefore putting the onus on usage by individual patients and (where also specified) their carers. Our definition of health was broad. We included studies focusing on both clinical measurements (like blood pressure and heart rhythm) and other factors that influence health (such as physical activity and mood). We excluded technologies that require direct healthcare provider participation, such as telehealth and videoconferencing systems.

We excluded population-level disease surveillance systems, focusing instead on individual user attitudes. We defined attitudes broadly to include technology acceptance, prior knowledge, user experience, motivations, and perceived benefits and risks. Our definition of users encompassed patients, members of the general public, and both formal and informal caregivers who use monitoring technologies. We adopted the concept of primary (patients and members of the public targeted by technologies) and secondary users (carers) to highlight carers’ unique needs^91^. We excluded studies where the users were professionals, such as doctors, nurses, industry representatives, or policymakers.

The review was registered with PROSPERO before data extraction (CRD42023446772). Since studies on health monitoring technologies are interdisciplinary, to minimize bias, we searched multiple databases on August 1, 2023: PubMed, Embase, ACM Digital Library, IEEE Xplore, Web of Science, and Scopus. The selected databases are well-established for their comprehensive coverage of health, technology, and multidisciplinary research. Specifically, PubMed and Embase are essential resources for accessing medical and clinical studies, while IEEE Xplore and the ACM Digital Library provide critical insights into the engineering and computational dimensions of health technologies. Web of Science and Scopus offer broad multidisciplinary coverage, facilitating the inclusion of research spanning diverse fields. These databases are also recognized for their extensive international reach, supporting the inclusion of studies from various regions. Despite these databases’ broad coverage, we acknowledge that some regional studies may not be included due to database indexing limitations. We conducted a title and abstract search of literature published between January 1, 2000, and July 31, 2023. We consulted MeSH to identify search terms, but it did not provide specific terms for monitoring technologies. We therefore adopted a multiple-synonym search strategy (see Supplementary Table 1) to maximize the number of usable studies.

**Fig. 2:**
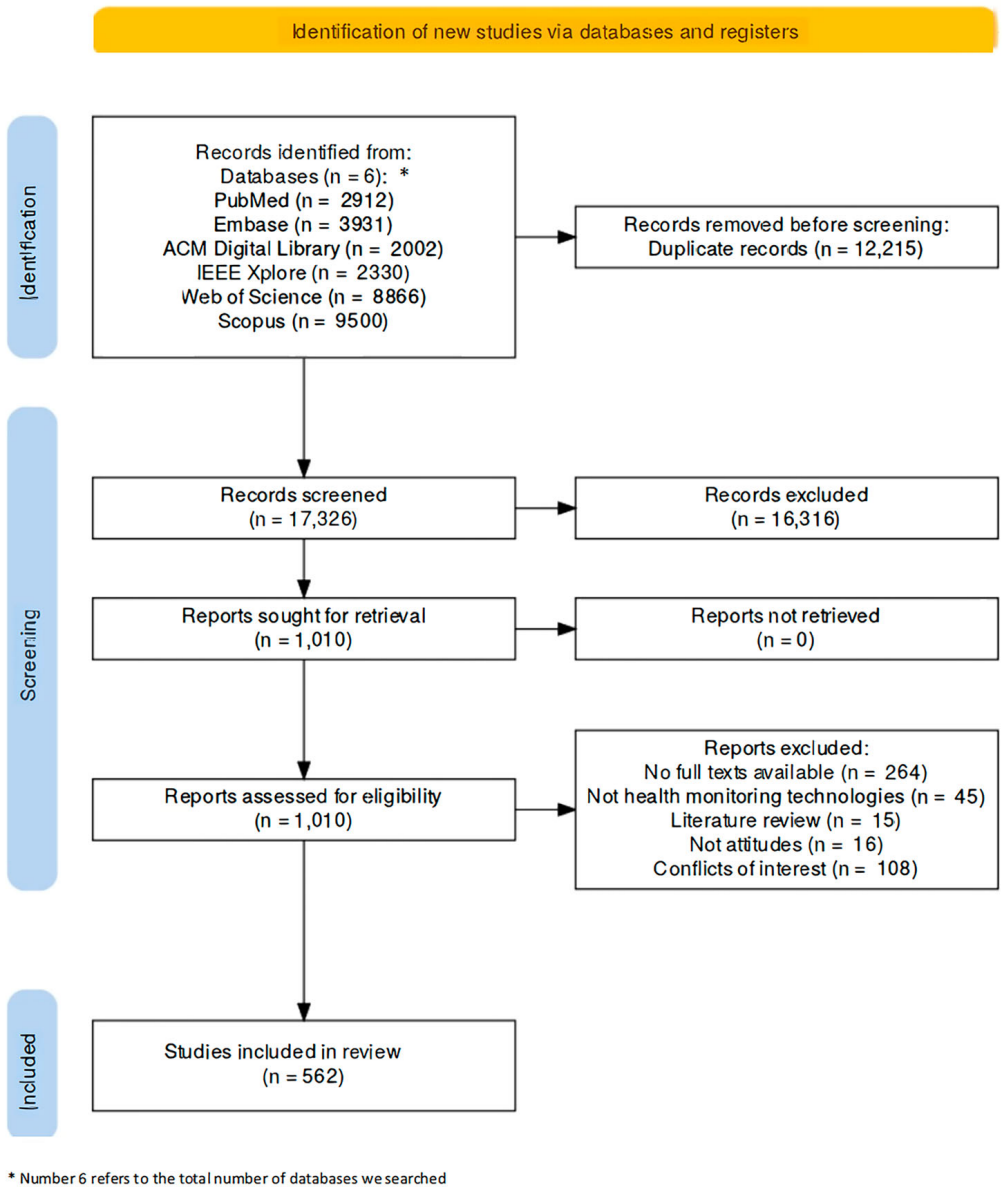
Preferred Reporting Items for Systematic reviews and Meta-Analyses (PRISMA) Chart. This flow chart illustrates the study selection process for this systematic review.

We imported the bibliographic search results into Rayyan^92^, a systematic review management platform, and eliminated duplicate entries. Two authors (TC and SV) independently screened study titles and abstracts. We defined our inclusion and exclusion criteria using the Population, Intervention, Comparison, Outcomes and Study (PICOS) framework to ensure transparency and methodological rigor. Table 3 summarizes the criteria used to screen studies for inclusion. The authors then independently reviewed the full texts of potentially eligible studies against exclusion criteria. TC and SV discussed and resolved any discrepancies between their assessments (see Supplementary Table 2) before finalizing the list of included literature. For example, we excluded an article about an automated call monitoring intervention for older wheelchair users^93^. Though automated calls monitor patients’ health information, we decided to exclude this article because it did not specify how automated calls would be answered or whether these calls were embedded in a device. Figure 2 summarizes the exclusion process.

**Table 3:** The PICOS framework for inclusion and exclusion criteria

PICOS element	Inclusion criteria	Exclusion criteria
Population	Patients, the general public, or formal/informal caregivers who use health monitoring technologies	Healthcare professionals, developers, industry representatives, or policymakers
Intervention	Studies involving the use or experience of health monitoring technologies (e.g., wearables, sensors)	Studies not involving health monitoring technologies (e.g., Population-level disease surveillance systems)
Comparator	N/A	N/A
Outcomes	Attitudes, perceptions, motivations, barriers, or experiences of users	Studies only reporting technical performance without user perspectives
Study design	Empirical studies using primary data (quantitative, qualitative, or mixed methods) published between 2000 and 2023. Both peer-reviewed study and pre-prints were included.	Reviews, commentaries, protocols, editorials, studies not using primary data, studies published out of the time frame

## Data analysis

Our systematic review identified 670 studies that met the inclusion criteria and passed quality assessment using the Mixed Methods Appraisal Tool^94^ (MMAT; see Supplementary Table 3). The Mixed Methods Appraisal Tool assesses study internal validity. TC and SV independently assessed each study. Our assessment focused several key aspects: the clarity of the research questions and the alignment between research questions and methodology, how representative the study participants are of the target population, and the transparency of data collection procedures. We excluded 108 studies that reported potential or actual conflicts of interest to mitigate potential biases that may have affected the internal validity of the studies (see Supplementary Table 4). Of the remaining 562 studies included in our synthesis, 348 explicitly declared no conflicts of interest, while 214 provided no conflict-of-interest information (see Fig. 2). For comparative purposes, we separately summarized the 108 excluded studies with declared conflicts in Supplementary Note 1. While we acknowledge the extensive literature examining conflicts of interest in research^95^, analyzing this body of work fell outside the scope of our systematic review. Our analysis focused solely on conflicts of interest as defined by journal publication requirements.

We piloted an Excel data extraction sheet (see Supplementary Table 5) using five randomly selected articles. TC and SV independently extracted publication year, study design, study location, technology type, target users, sample characteristics, sampling strategies, and attitude results. Due to the number of studies included, we only used information made available in the studies rather than contacting authors to obtain any information that was not included.

We conducted a numeric analysis to calculate the number of studies by various categories such as publication year and study design and a thematic analysis of attitude results using Virginia Braun and Victoria Clarke’s six-stage thematic analysis protocol^96^. We did not use meta-analysis because we included heterogeneous study designs. TC then thematically coded the results on attitudes using MAXQDA, merging similar codes and identifying the most significant or frequent initial qualitative analysis codes which could be used to develop themes. Initial coding was discussed with EH, and the codebook was modified (see Supplementary Table 6). Finally, to infer analytical themes, TC organized the codes according to different aspects of users’ attitudes such as acceptability and usability. Analytical themes were refined through discussion among all authors.

## Supplementary information

Supplementary information

## Data Availability

Data supporting this study are available within the main article and Supplementary Table 9.

## Code availability

The codebook is available in Supplementary Table 6.
