# Supplementary material for: A systematic review on patient and public attitudes toward health monitoring technologies across countries
Source: NPJ Digit Med. 2025 Jul 12;8:433. doi: 10.1038/s41746-025-01762-4 (PMC12255672; doi:10.1038/s41746-025-01762-4)
Supplement: Supplementary file 1 — Supplementary information [file 41746_2025_1762_MOESM1_ESM.pdf]

Supplementary information

Supplementary Table 1: Search strategies

Database	Search strings*
PubMed	((public[Title/Abstract] OR citizen[Title/Abstract] OR patient[Title/Abstract] OR user[Title/Abstract] OR people[Title/Abstract] OR community[Title/Abstract]) AND (attitude[Title/Abstract] OR knowledge[Title/Abstract] OR views[Title/Abstract] OR viewpoints[Title/Abstract] OR opinions[Title/Abstract] OR perspectives[Title/Abstract] OR perception[Title/Abstract] OR beliefs[Title/Abstract] OR acceptability[Title/Abstract] OR experience[Title/Abstract] OR questionnaire[Title/Abstract] OR survey[Title/Abstract] OR consultation[Title/Abstract] OR discussion[Title/Abstract] OR debate[Title/Abstract])) AND ("monitoring technology"[Title/Abstract] OR "monitoring system"[Title/Abstract] OR "monitoring device"[Title/Abstract] OR wearable[Title/Abstract] OR sensor[Title/Abstract]) NOT (review[Title])
Embase	(public OR citizen OR patient OR user OR people OR community) AND (attitude OR knowledge OR views OR viewpoints OR opinions OR perspectives OR perception OR beliefs OR acceptability OR experience OR questionnaire OR survey OR consultation OR discussion OR debate) AND ("monitoring technology" OR "monitoring system" OR "monitoring device" OR wearable OR sensor) NOT review {No Related Terms}
ACM Digital Library	[[Abstract: public] OR [Abstract: citizen] OR [Abstract: patient] OR [Abstract: user] OR [Abstract: people] OR [Abstract: community]] AND [[Abstract: attitude] OR [Abstract: knowledge] OR [Abstract: views] OR [Abstract: viewpoints] OR [Abstract: opinions] OR [Abstract: perspectives] OR [Abstract: perception] OR [Abstract: beliefs] OR [Abstract: acceptability] OR [Abstract: experience] OR [Abstract: questionnaire] OR [Abstract: survey] OR [Abstract: consultation] OR [Abstract: discussion] OR [Abstract: debate]] AND [[Abstract: "monitoring technology"] OR [Abstract: "monitoring system"] OR [Abstract: "monitoring Device"] OR [Abstract: wearable] OR [Abstract: sensor]] AND [[Abstract: medic*] OR [Abstract: clinic*] OR [Abstract: health*]] AND [E-Publication Date: (01/01/2000 TO 12/31/2023)]
IEEE Xplore	("Abstract":public OR "Abstract":citizen OR "Abstract":patient OR "Abstract":user OR "Abstract":people OR "Abstract":community) AND ("Abstract":attitude OR "Abstract":knowledge OR "Abstract":views OR "Abstract":viewpoints OR "Abstract":opinions OR "Abstract":perspectives OR "Abstract":perception OR "Abstract":beliefs OR "Abstract":acceptability OR "Abstract":experience OR "Abstract":questionnaire OR "Abstract":survey OR "Abstract":consultation OR "Abstract":discussion OR "Abstract":debate) AND ("Abstract": "monitoring technology" OR "Abstract": "monitoring system" OR "Abstract":"monitoring device" OR "Abstract":wearable OR "Abstract":sensor) AND ("Abstract":medic* OR "Abstract":clinic* OR "Abstract":health*) NOT ("Document Title":review)
Web of Science	public OR citizen OR patient OR user OR people OR community (Abstract) AND attitude OR knowledge OR views OR viewpoints OR opinions OR perspectives OR perception OR beliefs OR acceptability OR experience OR questionnaire OR survey OR consultation OR discussion OR debate (Abstract) AND "monitoring technology" OR "monitoring system" OR "monitoring device" OR wearable OR sensor (Abstract) AND medic* OR clinic* OR health* (Abstract) NOT review (Title) and 2023 or 2022 or 2021 or 2020 or 2019 or 2018 or 2017 or 2016 or 2015 or 2014 or 2013 or 2012 or 2011 or 2010 or 2009 or 2008 or 2007 or 2006 or 2005 or 2004 or 2003 or 2002 or 2001 or 2000 (Publication Years)
Scopus	(ABS (public OR citizen OR patient OR user OR people OR community) AND ABS (attitude OR knowledge OR views OR viewpoints OR opinions OR perspectives OR perception OR beliefs OR acceptability OR experience OR questionnaire OR survey OR consultation OR discussion OR debate) AND ABS ("monitoring technology" OR "monitoring system" OR "monitoring device" OR wearable OR sensor) AND ABS (medic* OR clinic* OR health*) AND NOT TITLE (review)) AND PUBYEAR > 1999 AND PUBYEAR < 2024 AND (LIMIT-TO (DOCTYPE , "ar") OR LIMIT-TO (DOCTYPE , "cp") OR LIMIT-TO (DOCTYPE , "ch") OR LIMIT-TO (DOCTYPE , "cr") OR LIMIT-TO (DOCTYPE , "bk") OR LIMIT-TO (DOCTYPE , "sh") OR LIMIT-TO (DOCTYPE , "dp") OR LIMIT-TO (DOCTYPE , "no")) AND (EXCLUDE (SUBJAREA , "ENER") OR EXCLUDE (SUBJAREA , "EART") OR EXCLUDE (SUBJAREA , "VETE") OR EXCLUDE (SUBJAREA , "ENVI"))

* We adopted a multiple-synonym search strategy. For instance, for attitudes, we searched synonyms such as experiences, perspectives, and surveys, etc. For monitoring technologies, we searched synonyms such as sensors, wearables, and trackers. For health, we used synonyms such as wellness and clinical. To reduce search results, we set a filter to exclude reviews. In addition, as Scopus brought excessive search results, we also filter subjects to exclude environmental science, vet, earth science, and energy.

Supplementary Table 2: Abstract screening process

Date	Activity	Abstract Screened
2023.8.1	TC created the review project on Rayyan. 17326 abstracts to double screen	0
9.28	18% disagreement rate; TC ans SV reviewed disagreements	14717
10.5	Update meeting; 16.7% disagreement rate	15919
10.19	Updated meeting; 12% disagreement rate	16921
10.26	Screening complete; 9.8% disagreement rate	17326
11.2	Reconciliation: 4.5% disagreement rate	17326
11.9	Reconciliation complete: 0% disagreements	17326

Supplementary Table 3: The Mixed Methods Appraisal Tool (Example)

**Title**	**Security Risks and User Perception towards Adopting Wearable Internet of Medical Things**
Year	2023
Journal	International Journal of Environmental Research and Public Health
Authors	Thapa S, Bello A, Maurushat A, Farid F.
Doi	10.3390/ijerph20085519
Are there clear research questions? （YES/NO/CANNOT TELL）	Yes
Do the collected data allow to address the research questions? （YES/NO/CANNOT TELL）	Yes
Are the participants representative of the target population? （YES/NO/CANNOT TELL）	Yes
Does the study provide transparent and detailed data collection procedures (Yes/NO/CANNOT TELL)	Yes

Supplementary Table 4: Conflicts of Interests (Example)

**Title**	**Security Risks and User Perception towards Adopting Wearable Internet of Medical Things**
Conflict of interests (YES if the article declares conflicted interests or if the authors include technology manufactures/ NO if the article declares no conflicts of interest/UNDECLARED if the interests of conflict section is blank)	No

Supplementary Table 5: Data extraction sheet (Example)

**Title**	**Security Risks and User Perception towards Adopting Wearable Internet of Medical Things**
Year	2023
Journal	International Journal of Environmental Research and Public Health
Authors	Thapa S, Bello A, Maurushat A, Farid F.
Doi	10.3390/ijerph20085519
Abstract	The Wearable Internet of Medical Things (WIoMT) is a collective term for all wearable medical devices connected to the internet to facilitate the collection and sharing of health data such as blood pressure, heart rate, oxygen level, and more. Standard wearable devices include smartwatches and fitness bands. This evolving phenomenon due to the IoT has become prevalent in managing health and poses severe security and privacy risks to personal information. For better implementation, performance, adoption, and secured wearable medical devices, observing users' perception is crucial. This study examined users' perspectives of trust in the WIoMT while also exploring the associated security risks. Data analysed from 189 participants indicated a significant variance (R2 = 0.553) on intention to use WIoMT devices, which was determined by the significant predictors (95% Confidence Interval; p < 0.05) perceived usefulness, perceived ease of use, and perceived security and privacy. These were found to have important consequences, with WIoMT users intending to use the devices based on the trust factors of usefulness, easy to use, and security and privacy features. Further outcomes of the study identified how users' security matters while adopting the WIoMT and provided implications for the healthcare industry to ensure regulated devices that secure confidential data.
Study design	Survey
Study location	Australia
Technology type	Wearable internet of medical things
Target users	The general public
User characteristics	189 participants that belonged to diverse cultures, backgrounds, and age groups. Starting with age, more than half (42.9%) belonged to the 25 to 34 years age group, followed by 37% of those between ages 18 and 24, 9.5% between 35 and 44, and 6.9% between 45 and 54. There were seven respondents aged 55 or above. A little more than half of the respondents identified themselves as female. Males comprised 46.6% of the total respondents.
Sampling strategies	The survey was conducted online, with participants signing up for the study, and data were collected via Qualtrics (an online-based survey platform)
Key findings on attitudes	More than half (65.6%) of them were familiar with WIoMT devices, and among them, 77 mentioned they used such devices, and 47 said they did not. Similarly, when those who used such devices were asked if they were aware of any of the listed WIoMT devices, only one-fourth (23.8%) selected Wearable Fitness Trackers, 3.2% selected Wearable ECG Monitors, 6.9% Wearable Blood Pressure Monitors, 2.1% Smart Patches, and 4.8% mentioned they were aware of none of the listed devices.<br><br>It means that the respondents were ready to adopt WIoMT devices provided that the devices have proper functionality. An increase in functionality was found to increase users' perception of security and privacy. This creates more awareness of the potential risks of the use of WIoMT. It leads to trust, which finally results in the high possibility of adopting WIoMT devices, as intention to use becomes strong.<br><br>This means that, with the increase in the perceived usefulness of WIoMT devices, there is a significant increase in the security and privacy perception of such devices among consumers. There was a moderate positive correlation between perceived ease of use and perceived security and privacy, and it was significant at p < 0.001. (Table 10). This means that, with the increase in the perceived ease of use of WIoMT devices, there is a significant increase in the perceived security and privacy of such devices among consumers.The correlation analysis showed that there was a significant correlation between product- and security-related factors and intention to use<br><br>Malware infections and vulnerabilities; lack of regulation and compliance; unsecured network connectivity; lack of encryption; and lack of patching and device updates. Most of them felt "unauthorised access to data" was the most prominent risk for these devices, followed by "unsecured network connectivity" and "malware infections and vulnerabilities".

Supplementary Table 6: Codebook

Variables/Themes	Codes	Sub-codes
Study design	Quantitative (eg. surveys and questionnaire-based clinical trials), qualitative (eg. interviews, focus groups and discourse analysis), mixed	
Study location	Global South, Global North, Both, Unspecified (We coded "unspecified" if the geographical location of the studied sample was not mentioned. We did not use the primary author affiliation as a proxy since it does not always align with the study location.) We employed the 2021 regional classifications provided by International Telecommunications Union	
	High Income countries, Upper Middle, Lower Middle, Low, Upper middle+high, Low+middle+high, lower middle+high, lower middle+upper middle+high, Unspecified The World Bank assigns the world's economies to four income groups —low, lower-middle, upper-middle, and high income. We coded the geographical areas according to its 2024 data.	
Sample reporting	Gender	Y/N/NA (*sexual orientation)
	Age	Y/N/NA
	Ethnicity	Y/N/NA
	Income level	Y/N/NA
	Education	Y/N/NA
	Rural/urban residence;distance from hospital	Y/N/NA
	Representative/ convenient sampling	
	Disadvantaged sampling	Y/N
	Sample size	Less than 50, between 50 and 100, between 100 and 500, more than 500
Target user	Primary/secondary users	
Health conditions	Specialty	Gerontology, diabetes, physical activity, mental health, etc
	Diseases or Not	Y/N
Technology type	Wearable, app, smart home system, non-wearable, implanted device, etc.	
	AI powered or Not (We defined a technology is powered by AI when the article specified "artificial intelligence" or "machine learning.")	Y/N
Technology knowledge	Familiar (Respondents who were unfamiliar about technologies outnumber those who were familiar about technologies.)	
	Unfamiliar (Respondents who were familiar about technologies outnumber those who were unfamiliar about technologies.)	
Social determinants of knowledge	Demographic factors	
	Perceived usefulness	
Acceptability	Mixed (We coded "mixed" if a study presented both positive and negative attitudes.)	
	Low interest/negative (We coded "negative/low interest" if a study only presented that most of their respondents did not accept or had no interests in technologies.)	
	Positive (We coded "positive" if a study only presented that most of their respondents accepted technologies.)	
Determinants of acceptability	Demographic factors	
	No correlation with demographic factors	
	Self control	
	Appearance	
	External	
	Technology difference	
	desires	
	Location	
	Knowledge	
	Relationship with others	
	Perceived ease of use	
	Perceived usefulness	
	Security	
Usability	Ease of use	
	Comfortable	
	Appearance appealing	
	Durability	
	Language	
	Self-application	
	Technical difficulties	

Motivations of using technology	Perceived usefulness	
	Perceived ease of use	
	Social relationship	
	Competition	
	Curiosity	
	Anxiety	
	Pleasure	
	Fun/coolness	
	brand	
	External environment	
	Giving back	
User requirement	Information storage	
	Eliminate Ads	
	Offline activity	
	More inclusive	
	Context of use	
	Justification	
	Comfort	
	Wording	
	Loss	
	Unobtrusiveness	
	Functionality	
	Location	
	Gamification	
	Tailored feedback	
	Data interpretation	
	Appearance features	
Barriers	Unfit body shape	
	Not inclusive	
	Usability issue	
	No correlation	
	Not convinced	
	The foreign body	
	Lack of autonomy	
	Lack of collaboration	
	Knowledge	
	Forget	
	Incompatible with other devices	
	Access issues	
	Cost (time, money, etc)	
	Functionality	
	Interfere current life	
	Replace existing care	
	No need	
	Embarrassment	
	Data sharing concern	
	distrust	
How to motivate	Compatibility	
	Reduce cost	
	Award/stimulus	
	Promote understanding	
	One-to-one basis/build relationship	
Benefits	Technology development	
	Individualized treatment	
	Contribute to research/social good	
	Promote policy/infrastructure	
	Improve privacy	
	Supplement/replace routine test	
	Security	
	Good relationship	
	Legitimize their symptoms	
	Behavior change	
	awareness	
	Reduction burden	
	Improving their adherence	
	Support	
	Improve confidence/accomplishment	
	Self-control	
Risks	Impact professionalism	
	Dehumanizing care	
	Decrease opportunity	

	Bias	
	Accuracy	
	Surveillance	
	Burden	
	Reduced autonomy	
	Adverse events	
	Privacy	
	Malfunction	

Supplementary Table 7: PRISMA Checklist

Section and Topic	Item #	Checklist item	Location where item is reported
**TITLE**			
Title	1	Identify the report as a systematic review.	P.1
**ABSTRACT**			
Abstract	2	See the PRISMA 2020 for Abstracts checklist.	P.2
**INTRODUCTION**			
Rationale	3	Describe the rationale for the review in the context of existing knowledge.	P.3
Objectives	4	Provide an explicit statement of the objective(s) or question(s) the review addresses.	P.3
**METHODS**			
Eligibility criteria	5	Specify the inclusion and exclusion criteria for the review and how studies were grouped for the syntheses.	P.9
Information sources	6	Specify all databases, registers, websites, organisations, reference lists and other sources searched or consulted to identify studies. Specify the date when each source was last searched or consulted.	P.9
Search strategy	7	Present the full search strategies for all databases, registers and websites, including any filters and limits used.	P.9/Supplementary Information
Selection process	8	Specify the methods used to decide whether a study met the inclusion criteria of the review, including how many reviewers screened each record and each report retrieved, whether they worked independently, and if applicable, details of automation tools used in the process.	P.9
Data collection process	9	Specify the methods used to collect data from reports, including how many reviewers collected data from each report, whether they worked independently, any processes for obtaining or confirming data from study investigators, and if applicable, details of automation tools used in the process.	P.10
Data items	10a	List and define all outcomes for which data were sought. Specify whether all results that were compatible with each outcome domain in each study were sought (e.g. for all measures, time points, analyses), and if not, the methods used to decide which results to collect.	P.10
	10b	List and define all other variables for which data were sought (e.g. participant and intervention characteristics, funding sources). Describe any assumptions made about any missing or unclear information.	P.10/Supplementary Information
Study risk of bias assessment	11	Specify the methods used to assess risk of bias in the included studies, including details of the tool(s) used, how many reviewers assessed each study and whether they worked independently, and if applicable, details of automation tools used in the process.	P.10
Effect measures	12	Specify for each outcome the effect measure(s) (e.g. risk ratio, mean difference) used in the synthesis or presentation of results.	N/A
Synthesis methods	13a	Describe the processes used to decide which studies were eligible for each synthesis (e.g. tabulating the study intervention characteristics and comparing against the planned groups for each synthesis (item #5)).	P.10
	13b	Describe any methods required to prepare the data for presentation or synthesis, such as handling of missing summary statistics, or data conversions.	P.10

	13c	Describe any methods used to tabulate or visually display results of individual studies and syntheses.	P.10
	13d	Describe any methods used to synthesize results and provide a rationale for the choice(s). If meta-analysis was performed, describe the model(s), method(s) to identify the presence and extent of statistical heterogeneity, and software package(s) used.	P.10
	13e	Describe any methods used to explore possible causes of heterogeneity among study results (e.g. subgroup analysis, meta-regression).	P.10/Supplementary information
	13f	Describe any sensitivity analyses conducted to assess robustness of the synthesized results.	N/A
Reporting bias assessment	14	Describe any methods used to assess risk of bias due to missing results in a synthesis (arising from reporting biases).	P.10/Supplementary information
Certainty assessment	15	Describe any methods used to assess certainty (or confidence) in the body of evidence for an outcome.	P.10/Supplementary information
**RESULTS**			
Study selection	16a	Describe the results of the search and selection process, from the number of records identified in the search to the number of studies included in the review, ideally using a flow diagram.	P.20
	16b	Cite studies that might appear to meet the inclusion criteria, but which were excluded, and explain why they were excluded.	P.9
Study characteristics	17	Cite each included study and present its characteristics.	Supplementary Information
Risk of bias in studies	18	Present assessments of risk of bias for each included study.	Supplementary Information
Results of individual studies	19	For all outcomes, present, for each study: (a) summary statistics for each group (where appropriate) and (b) an effect estimate and its precision (e.g. confidence/credible interval), ideally using structured tables or plots.	Supplementary Information
Results of syntheses	20a	For each synthesis, briefly summarise the characteristics and risk of bias among contributing studies.	P.3
	20b	Present results of all statistical syntheses conducted. If meta-analysis was done, present for each the summary estimate and its precision (e.g. confidence/credible interval) and measures of statistical heterogeneity. If comparing groups, describe the direction of the effect.	P.3
	20c	Present results of all investigations of possible causes of heterogeneity among study results.	P.4
	20d	Present results of all sensitivity analyses conducted to assess the robustness of the synthesized results.	N/A
Reporting biases	21	Present assessments of risk of bias due to missing results (arising from reporting biases) for each synthesis assessed.	Supplementary Information
Certainty of evidence	22	Present assessments of certainty (or confidence) in the body of evidence for each outcome assessed.	Supplementary Information
**DISCUSSION**			
Discussion	23a	Provide a general interpretation of the results in the context of other evidence.	P.6
	23b	Discuss any limitations of the evidence included in the review.	P.8

	23c	Discuss any limitations of the review processes used.	P.8
	23d	Discuss implications of the results for practice, policy, and future research.	P.7
**OTHER INFORMATION**			
Registration and protocol	24a	Provide registration information for the review, including register name and registration number, or state that the review was not registered.	P.9
	24b	Indicate where the review protocol can be accessed, or state that a protocol was not prepared.	P.9
	24c	Describe and explain any amendments to information provided at registration or in the protocol.	N/A
Support	25	Describe sources of financial or non-financial support for the review, and the role of the funders or sponsors in the review.	P.11
Competing interests	26	Declare any competing interests of review authors.	P.11
Availability of data, code and other materials	27	Report which of the following are publicly available and where they can be found: template data collection forms; data extracted from included studies; data used for all analyses; analytic code; any other materials used in the review.	P.11

Supplementary Note 1: Summary of studies declaring conflicts of interest

Of the 670 studies that fulfilled the inclusion criteria, 214 studies had no section on conflicts of interest, 348 studies declared no conflicts of interest, and 108 studies disclosed potential or actual conflicts of interest. Examples of conflicted interests included studies funded by companies producing healthcare monitoring technologies and studies written by staff from technology companies. The following is the summary of studies declaring potential or actual conflicts of interest.

- Year of publish

They were published between 2011 and 2021.

- Study design

53.8% studies were quantitative studies, 23. 1% studies were qualitative studies, and 23. 1% studies were mixed methods studies.

- Study location

40 studies were from the US, 19 studies were from the UK, and 7 studies were from Australia. 95 studies were from Global North. 98 studies were from High Income Countries. Only 1 study was from Low Income Countries.

- Sample reporting

84.2% studies reported gender, 87% studies reported age, 30.5% studies reported ethnicity, 19.4% studies reported income, 30.5% studies reported education, and 1.8% studies reported residency.
97 studies adopted convenience sampling.The sample size of 64 studies was less than 50.

- User type

95 studies targeted primary users.

- Health conditions

90 studies focused on a specific disease.

- Technology type

32 studies featured wearables, 13 studies featured apps, and 5 studies featured smart home systems. None of the studies featured technologies powered by AI.

- Knowledge about technologies

Of 15 studies declaring potential or actual conflicts of interest, 7 (46.7%) studies reported unfamiliarity.In comparison, of 59 studies without disclosure of interest conflicts, 37 (62.7%) studies reported unfamiliarity.

- Attitudes

Of 69 studies declaring conflicts of interest, 67 (97. 1%) reported positive attitudes. In comparison, Of 294 articles studying acceptability of health monitoring technologies, 259 (88. 1%) studies found positive attitudes to technologies.

Supplementary Table 8: Sample characteristics of studies showing negative or mixed attitudes toward health monitoring technologies

paper id	paper title	publication year	country	region	device	methodology	sample	sample size	Gender	Age	Race	Ethnicity	Nationality	Education	Cohabitation	Marital Status	Job	Annual income	Ideology	Religion
22	Perceived Need and Acceptability of an App to Support Activities of Daily Living in People With Cognitive Impairment and Their Carers: Pilot Survey Study	2020	Germany	Global North	Cognitive Impairment	Mixed	participants with mild cognitive impairment or dementia and their family carers	48	patients: 8M 16 F Carers: 15 M 9F	patients(SD, range): Mean 74.5 (6.1, 57-84) Carers: Mean 62.4 (16.0, 31-83)				Education, > 12 years, Patients: 8 Carers: 11						
95	Toward a precision behavioral medicine approach to addressing high-risk sun exposure: A qualitative analysis	2019	USA	Global North	skin cancer	qualitative	Early stage adult melanoma survivors	50	Focus group: 5M 6F Observation: 15M 24F	Focus group (SD, range): Mean 46.82 (13.08, 28-65) Observation: Mean 58.59 (13.44, 28-84)		All study participants identified as White/Non-Hispanic.								
107	Acceptability of an intelligent wireless sensor system for the rapid detection of health issues: Findings among home-dwelling older adults and their informal caregivers	2016	switherland	Global North	smart home systems for older adults	Quantitative	home-dwelling older adults	34	11M 23F	Mean 83.2 (7.2)					31 live alone, and 3 live with spouse	5 are unmarried, 1 is married, 3 are divorced, 24 are widowed, and 1 is missing.				
67	Usability Study of a wireless monitoring system among Alzheimer's disease elderly population	2014	Italy	Global North	Alzheimer	qualitative	the elderly with advanced Alzheimer's disease	4		75-92										
97	Accuracy and Adoption of Wearable Technology Used by Active Citizens: A Marathon Event Field Study	2017	Germany	Global North	Activity tracker	Quantitative	runner	771	Full marathon: 133M 36F Half marathon: 396M 239F	Full marathon: 16-79 Half marathon: 16-79										
205	User Preferences and Design Recommendations for an mHealth App to Promote Cystic Fibrosis Self-Management	2014	USA	Global North	cystic fibrosis management app	Mixed	people with cystic fibrosis	15	8M 7F	Mean 30.2 (5.9, 21-43)		15 Caucasian		11 have college degree or beyond		11 are married/partnered	11 work full or part time	9 have % ≥US $100,000		
234	Qualitative investigation into a wearable system for chronic obstructive pulmonary disease: the stakeholders' perspective	2016	Greece,UK, Ireland and the Netherlands	Global North	a wearable system for chronic obstructive pulmonary disease	qualitative	patients with COPD with heart failure, diabetes, anxiety or depression, informal carers and	82	patients:17M 15F	37.5% (51-60) 28.1% (61-70) 28.1% (71-80) 6.3% (81-90)			10 British, 7 Greek, 8 Dutch, 7 Irish							
245	Use of wearable sensors for pregnancy health and environmental monitoring: Descriptive findings from the perspective of patients and providers	2019	USA	Global North	wearable sensors for pregnancy health and environmental monitoring	Quantitative	pregnant women and their providers	131	providers: 21M 7F	Pregnant women: Mean 27 (6.4) Providers: 21-50	Pregnant women:89 white, 5 black, 1 Asian, 7 Mixed race, 1 other Providers; 24white, 2 Asian, 1 Native Hawiian, 1	Pregnant women: 97 not hispanic/latino, 6 hispanic/latino Providers: 0 hispanic/latino, 27 not hispanic/latino		Pregnant women: 7 some high school, 51 high school, 13 associate degree, 32 college degree		Pregnant women: 58 married, 1 separated, 10 single, 32 unmarried relationship, 1 divorced, 0 widowed, 1 declined to answer				
248	The challenges of using medication event monitoring technology with pediatric transplant patients	2007	USA	Global North	medication event monitoring technology with pediatric transplant patients	Quantitative	pediatric kidney transplant patients	29	21M 8F	Mean 14.03(3.34, 8-19)		Caucasian, 13.8% African-American, 3.4% Asian, 6.9% Latino, 6.9% multi-ethnic						The median annual household income was approximately $61 000–70 000 (range <$12 000 to >$80 000).		
264	Women's Attitudes Toward Self-Monitoring of Their Pregnancy Using Noninvasive Electronic Devices: Cross-Sectional Multicenter Study	2019	Germany	Global North	fetal monitoring	Quantitative	pregnant women	509		12 are 18-20, 47 are 21-25, 168 are 26-30, 178 are 31-35, 84 are 36-40, 17 are >41 31-35 178 (35.2) 36-40 84 (16.6) >41 17 (3.4)				11 Dropped out of school, 41 Secondary education ending with ninth grade, 147 Secondary education ending with tenth grade, 101 University entrance diploma, 207 University degree		living with spouse, 9 Married, living separated from spouse, 16 Single without children , 11 Single with children,	143 Full-time (>35 hours per week) 68 Part-time (15-34 hours per week) 1 By the hour (1-14 hours per week) 20 Educational training (student) 41 Housewife 14 Unemployed 219 Leave of absence (ie, maternity leave)			
278	Beyond novelty effect: a mixed-methods exploration into the motivation for long-term activity	2019	USA	Global North	Activity tracker	Mixed	Fitbit device users	23	7M 16F											
280	Understanding and meeting injection device needs in multiple sclerosis: a survey of patient attitudes and practices	2011	Canada, France, Germany, Italy, Spain, and the US	Global North	injection monitor for multiple sclerosis	Quantitative	patients	422	127M 295F	Mean 43 (10.7,18-64)										
283	Needs of Lung Cancer Patients Receiving Immunotherapy and Acceptance of Digital and Sensor-Based Scenarios for Monitoring Symptoms at Home-A Qualitative-Explorative Study	2022	Germany	Global North	immunotherapy monitor	qualitative	patients with lung cancer	21	14M 7F	Median 65 (50-81)					2 Living alone, 18 Living with partner or family, 1 Other arrangement					
289	The Design and Evaluation of Personalised Ambient Mental Health Monitors	2010	UK	Global North	Mental health monitor	Mixed	people with Bipolar Disorder	4	4M											
300	Attitudes of Professional Caregivers and Family Members Regarding the Use of Monitoring Devices to Improve Assessments of Pain and Discomfort During Continuous Sedation Until	2020	Belgium	Global North	Palliative care monitor	qualitative	caregiver and family members.	15	8M 7F	2 are 40-49, and 13 are older than 50										
313	Stakeholder Perspectives on In-home Passive Remote Monitoring to Support Aging in Place in the Province of New Brunswick, Canada: Rapid Qualitative Investigation	2022	Canada	Global North	smart home systems for older adults	qualitative	caregivers	26												
328	Perspectives on wellness self-monitoring tools for older adults	2013	USA	Global North	self monitor for older adults	qualitative	HCP and community-dwelling older adults	41		older adults were aged 62 years or older.					older adults were residents of the independent retirement community					
333	Health technology identities and self. Patients' appropriation of an assistive device for self-management of chronic illness	2020	Denmark	Global North	assistive device for self-management of chronic illness	qualitative	patients with chronic obstructive pulmonary disease	39	25M 14F											

336	Perceptions of seniors with heart failure regarding autonomous zero-effort monitoring of physiological parameters in the smart-home environment	2017	Canada	Global North	autonomous zero-effort monitoring of physiological parameters	qualitative	seniors with heart failure	26	19M 7F	74.96 ± 6.69						
347	Young people's uses of wearable healthy lifestyle technologies; surveillance, self-surveillance and resistance	2019	UK	Global North	Activity tracker	qualitative	young people	100	47M 53F	A minimum age of 13 was selected due to Fitbit's terms and conditions on age of use			School one was a non-selective private school in the South-East of England. School two was a comprehensive state school based in the North-West of England.			
392	Using a Proximity-Detection Technology to Nudge for Physical Distancing in a Swedish Workplace during the COVID-19 Pandemic: Retrospective Case Study	2022	Sweden	Global North	distance detector for COVID-19	Mixed	employee	38	interview: 5M 3F, survey: 18M 12F	Mean 46.2 (1.96, 28-68)						
422	Prospective Intention-Based Lifestyle Contracts: mHealth Technology and Responsibility in Healthcare	2021	UK	Global North	general	Quantitative	public	81	30.9% M, 69.1% F Female 69.1 Non-binary 0 Prefer not to say 0	23.5% 18–24, 32.1% 25–34,21% 35–44, 8.6% 45–54,9.9% 55–64,4.9% 65+, 0% Prefer not to say			0% Primary school education, 27.2% Secondary school education,48.1% Undergraduate tertiary education, 24.7% Postgraduate tertiary education, 0% Prefer not to say		11.1% Very left-wing/liberal, 19.8% Moderately left-wing/liberal, 23.5% Somewhat left-wing/liberal， 28.4% Neither left-wing/liberal nor right-wing/conservative， 7.4% Somewhat right-wing/conservative, 6.2% Moderately right-wing/conservative, 0% Very right-wing/conservative, 3.7% Prefer not to say	
453	Immersive Virtual Reality and Gamification Evaluation on Treadmill Exercise by Using Electrophysiological Monitoring Device	2020	Indonesia	Global South	Activity tracker	Quantitative	public	24	13M 11F	Mean 20.33 (0.99, 19-23)			undergraduate students			
464	Wearable devices and measurement data: An empirical study on ehealth and data sharing	2021	sweden and ireland	Global North	wearable	qualitative	public	29	16M, 12F, 1 prefers 'not to say' their gender.	21–60						
256	Facilitators and barriers to a contingency management alcohol intervention involving a transdermal alcohol sensor	2020	USA	Global North	alcohol sensor	Qualitative	heavy drinkers	37	18M 19F		16 African American 18 White 13 Multiracial	11 Hispanic, 26 Not Hispanic				
537	Tracking down a solution: exploring the acceptability and value of wearable GPS devices for older persons, individuals with a disability and their support persons	2017	Australia	Global North	a wearable GPS device	Quantitative	older persons and their carers	12	patient 2M, 4F, carer 3F 3M	patient age 20-92, carer 47-68						
545	Toward patient-centered telerehabilitation design: Understanding chronic pain patients' preferences for web-based exercise telerehabilitation using a discrete	2017	Netherlands	Global North	Web-Based Exercise Telerehabilitation	Mixed	Chronic Pain Patients	104	38M, 66F	Mean 43.8 (14.8)			51 middle-high education	69 were unemployed		
588	Medical technology in smart homes: Exploring the user's perspective on privacy, intimacy and trust	2011	Germany	Global North	smart home for elderly or disabled people	qualitative	elderly or disabled people	165	78M, 87F	Mean 47.9 (18.7, 17-95)						
612	Sociocultural Dimensions of Tracking Health and Taking Care	2020	India	Global South	Chronic disease management	qualitative	patients with cardiac diseases	18	12M, 6F	Mean 60 (20-74)						
617	Patients, Pacemakers, and Implantable Defibrillators: Human Values and Security for Wireless Implantable Medical Devices - Proceedings of the SIGCHI Conference on Human Factors in Computing Systems	2010	Isarael	Global North	wireless Implantable devices	Qualitative	individuals with implanted cardiac de-vices.	13		Mean 67.9, median 69, range 41-80						
619	The 'Self' as Barrier for Self-Management Technologies in Healthcare? - Proceedings of the 12th EAI International Conference on Pervasive Computing Technologies for Healthcare	2018	Belgium	Global North	a technology solution to support transplant patients	qualitative	transplant patients	19	11M 8F	Mean 52 (21-69)						
657	How Mass Surveillance Crowds Out Installations of COVID-19 Contact Tracing Applications	2023	Isarael	Global North	contact tracing COVID 19	Quantitative	public	515	A total of [illegible] of theparticipants were women, 46% were men, and few participants had chosen the"other"category.	Mean 38 (13.8)			3 Elementary, 112 High school, 138 non, 184 Bachelor, 81 graduate	141 st below average, 165 below average, 138 average, 61 above average, 14 st above average		Jews (82%),Arab Muslims(10.5%),ArabChristians(2%),andDruze (4%).
671	Irish Attitudes Toward COVID Tracker App & Privacy: Sentiment Analysis on Twitter and Survey Data	2021	Ireland	Global North	contact tracing COVID 19	Quantitative	public	40820								
134	A Quantitative Research for Determining the User Requirements for Developing a System to Detect Depression	2021	Romania	Global South	depression detector	qualitative	public	205	73M 132F	under 35 – 80.1%, between 35 and 50 –10,7%, between 50 and 65 – 8.3% and between 65 and 76 –1.0%						
670	Mood Self-Assessment on Smartphones - Proceedings of the Conference on Wireless Health	2015	Singapore	Global South	mood self-assessment	Quantitative	students	40	60% males, 40% females	The survey involves participants only in the age range above 18 with no involvement of children or young people						

Supplementary Note 2: Summary of findings by socio-demographic factors

Supplementary Table 8 lists the characteristics of respondents from the studies which show negative or mixed attitudes toward health monitoring technologies. We did not draw definitive conclusions regarding the influence of socio-demographic factors on technology adoption since the samples in these studies are too diverse to make direct comparison. However, we provide a summary of some key findings related to socio-demographic factors to highlight areas requiring further research attention.

- Age

Research findings indicate that age significantly influences familiarity with and usability of health monitoring technologies, though the evidence is sometimes contradictory. For instance, a qualitative study involving focus groups with individuals aged over 65 taking cardiovascular medications in East London community centers found that older individuals were less familiar with such technologies[1]. Similarly, interviews with seven patients highlighted the challenges older adults face in operating an oxygen device, while another focus group study found that age often prevents elderly individuals from independently using wireless healthcare sensors[2,3]. However, not all studies support a direct correlation between age and technology acceptance. Some research suggests that socio-economic factors mediate this relationship, while others do not find such correlations. For example, a survey of 60 senior citizens found that age did not influence acceptance of Ambient Intelligence, an application for monitoring home environments[4]. These mixed findings highlight the need for further research to clarify the complex interactions between age and technology adoption.

- Gender

Research findings reveal diverse gender-based factors influencing the adoption of health monitoring technologies. Men demonstrated greater familiarity with and trust in certain technologies, as evidenced by an online survey showing men's trust in glucose monitoring and another survey indicating men were more likely to have heard of wireless sensor devices like Smart Dust[5,6]. However, no significant gender differences were found in attitudes toward ambient intelligence[4]. Additionally, women using fertility trackers, particularly those trying to conceive, expressed skepticism about the accuracy of fertility apps[7].

People's adoption of technologies often reflected preferences for gender-sensitive design. Female participants suggested feminizing adaptations for products such as hip protectors and underwear to improve their appeal, recommending different colors, patterns, or lace[8]. Similarly, interviews with pregnant women highlighted the need for appropriately sized wearables, as devices like sleep-monitoring rings designed for men were deemed too large for women[9]. For men, Manufacturers should consider special requests such as hairy chest for cardiac monitor[10]. These findings underscore the importance of gender-sensitive design and marketing in promoting the adoption of health monitoring technologies.

- Gender identity

Research found monitoring technology design has largely overlooked queer identities. For example, interviews and workshops with queer participants highlighted that queer individuals often find technology difficult to understand or use due to lack of queer options[11]. The findings emphasize the need for inclusive design considerations.

- Income

Higher income levels are positively associated with greater familiarity with health monitoring technologies, as found in a study about self-tracking devices[12].

- Education

Research indicates that individuals with higher levels of education are more perceptive to privacy risks associated with health monitoring technologies[13].

- Profession

Studies found that caregivers face challenges related to health monitoring technologies. They may feel burdened by the added responsibilities of reviewing daily results, instructing patients, and managing frequent calls[14]. Systems that unnecessarily direct patients to emergency departments can contribute to ED backlogs and increase caregiver frustration[15]. In times of crisis, caregivers often feel overwhelmed and hesitant to take on new responsibilities, although they acknowledge the potential benefits of

passive monitoring systems[16]. Concerns about mobile devices for receiving alarms were also raised, as they could affect the professionalism of healthcare operators[17].

- Region

Regional differences significantly influence attitudes toward technology adoption. For example, a study on fitness tracking in Egypt highlighted a deeper social context in Arabic communities, where users prioritized physiological measurements over goal achievement, unlike non-Arab Western users who focused more on achieving fitness goals[18]. These findings suggest the need for inclusive design dimensions that cater to diverse cultural and regional contexts.

[1] Holender, A., Sutton, S., and De Simoni, A. Opinions on the use of technology to improve tablet taking in >65-year-old patients on cardiovascular medications. *Journal of International Medical Research.* **46,** 2754–2768; https://doi.org/ 10.1177/0300060518770578 (2018).

[2] Hall, A., Boulton, E. and Stanmore, E. Older adults' perceptions of wearable technology hip protectors: implications for further research and development strategies. *Disability and Rehabilitation: Assistive Technology.* **14,** 663–668; https://doi.org/10.1080/17483107.2018.1491647 (2018).

[3] Virbel-Fleischman, C.et al. Body-Worn Sensors for Parkinson's disease: A qualitative approach with patients and healthcare professionals. PloS one. **17,** e0265438; https://doi.org/10.1371/journal.pone.0265438 (2022).

[4] Kirchbuchner, F., Grosse-Puppendahl, T., Hastall, M.R., Distler, M., and Kuijper, A. Ambient Intelligence from Senior Citizens' Perspectives: Understanding Privacy Concerns, Technology Acceptance, and Expectations. In: De Ruyter, B., Kameas, A., Chatzimisios, P., Mavrommati, I. eds *Ambient Intelligence 2015: Lecture Notes in Computer Science.* https://doi.org/10.1007/978-3-319-26005-1_4 (2015).

[5] Taleb, N., Quintal, A., Rakheja, R., Messier, V., Legault, L., Racine, E. and Rabasa-Lhoret, R. Perceptions and expectations of adults with type 1 diabetes for the use of artificial pancreas systems with and without glucagon addition: Results of an online survey. *Nutr Metab Cardiovasc Dis. 31,* 658-665; doi: 10.1016/j.numecd.2020.10.006 (2020).

[6] Lubrin, E., Lawrence, A., Zmijewska, K. F. Navarro and Culjak, G. Exploring the Benefits of Using Motes to Monitor Health: An Acceptance Survey. *International Conference on Networking,International Conference on Systems and International Conference on Mobile Communications and Learning Technologies (ICNICONSMCL '06).* doi: 10.1109/ ICNICONSMCL.2006.94 (2006).

[7] Gambier-Ross, K., McLernon, DJ., and Morgan, H. M. A mixed methods exploratory study of women's relationships with and uses of fertility tracking apps. DIGITAL HEALTH. **4,** 1; 10.1177/2055207618785077 (2018).

[8] Hall, A., Boulton, E. and Stanmore, E. Older adults' perceptions of wearable technology hip protectors: implications for further research and development strategies. *Disability and Rehabilitation: Assistive Technology.* **14,** 663–668; https://doi.org/10.1080/17483107.2018.1491647 (2018).

[9] Virbel-Fleischman, C.et al. Body-Worn Sensors for Parkinson's disease: A qualitative approach with patients and healthcare professionals. PloS one. **17,** e0265438; https://doi.org/10.1371/journal.pone.0265438 (2022).

[10] Honey, M.L., Smith, C., Parsons, J.G., Parsons, M., and Burgess, L. Consumers' perspectives of wireless cardiac monitoring: Results of a small New Zealand telehealth project. *Health Care and Informatics Review Online.* 15, 2-7 (2011).

[11] Adrian, B., Cochrane, K., and Girouard, A. Wearable Identities: Understanding Wearables' Potential for Supporting the Expression of Queer Identities. *Proceedings of the 2023 CHI Conference on Human Factors in Computing Systems (CHI '23).* **393,** 1–1 https://doi.org/10.1145/3544548.3581327 (2023).

[12] Paré, G., Leaver, C. and Bourget, C. Diffusion of the Digital Health Self-Tracking Movement in Canada: Results of a National Survey. *J Med Internet Res.* **20,** e177; doi: 10.2196/jmir.9388 (2018).

[13] Krutheeka, B. and Saji, K. M. Danger vs Fear: An Empirical Study on Wearable Users' Privacy Coping. *Proceedings of the 2020 on Computers and People Research Conference (SIGMIS-CPR '20)* : 123–132. https://doi.org/10.1145/3378539.3393856 (2020).

[14] Lew,S.Q., Sikka, N., Thompson, C., Cherian, T. and Magnus M. Adoption of Telehealth: Remote Biometric Monitoring Among Peritoneal Dialysis Patients in the United States. *Perit Dial Int.* **37,** 576-578; doi: 10.3747/pdi.2016.00272 (2017).

[15] Seto, E et al. Attitudes of heart failure patients and health care providers towards mobile phone-based remote monitoring. *J Med Internet Res.* **12,** e55; doi: 10.2196/jmir.1627 (2010).

[16] Read, E.A., Gagnon, D., Donelle, L., Ledoux, K., Warner, G. and Sharma, R.Stakeholder perspectives on in-home passive remote monitoring to support aging in place in the province of New Brunswick, Canada: A rapid qualitative analysis (Preprint). https://doi.org/10.2196/31486(2021).

[17] Bacchin, D., Pernice, G.F.A., Sardena, M., Malvestio, M., Gamberini, L. Caregivers' Perceived Usefulness of an IoT-Based Smart Bed. In: Streitz, N.A., Konomi, S. (eds) *Distributed, Ambient and Pervasive Interactions. Smart Environments, Ecosystems, and Cities*. https://doi.org/10.1007/978-3-031-05463-1_18 (2022).

[18] Niess, J. et al. "I Don't Need a Goal": Attitudes and Practices in Fitness Tracking beyond WEIRD User Groups. *Proceedings of the 23rd International Conference on Mobile Human-Computer Interaction.* https://doi.org/10.1145/3447526.3472062 (2021).

Supplementary Table 9: Full dataset
https://docs.google.com/spreadsheets/d/1XZI1bdaFOcSTooUXbiAGZ8BbbwoFl74XxaUCTJS9ekw/edit?usp=sharing

Title	Year	Journal	Authors	Doi	Study aim	Study design	Study location	User type	User characteristics	Type of monitoring technology	Knowledge of technology	Social determinants of knowledge	Acceptability	Determinants of acceptability	Usability	Motivations of using technology	User requirement	Barriers	How to motivate	Benefits	Risks	Conflict of interests (YES if the aricle declares conflicted interests/ COMPANY if the authors include technology manufactures)
Security Risks and User Perception towards Adopting Wearable Internet of Medical Things	2023	International Journal of Environmental Research and Public Health	Thapa S, Bello A, Maurushat A, Farid F.	10.3390/ijerph20085519	This study examined users' perspectives of trust in the Wearable Internet of Medical Things (WIoMT) while also exploring the associated security risks.	Quantitative	Australia	General public	189 participants that belonged to diverse cultures, backgrounds, and age groups. Starting with age, more than half (42.9%) belonged to the 25 to 34 years age group, followed by 37% of those between ages 18 and 24, 9.5% between 35 and 44, and 6.9% between 45 and 54. There were seven respondents aged 55 or above. A little more than half of the respondents identified themselves as female. Males comprised 46.6% of the total respondents.	Wearable internet of medical things	More than half (65.6%) of them were familiar with WIoMT devices, and among them, 77 mentioned they used such devices, and 47 said they did not. Similarly, when those who used such devices were asked if they were aware of any of the listed WIoMT devices, only one-fourth (23.8%) selected Wearable Fitness Trackers, 3.2% selected Wearable ECG Monitors, 6.9% Wearable Blood Pressure Monitors, 2.1% Smart Patches, and 4.8% mentioned they were aware of none of the listed devices.			It means that the respondents were ready to adopt WIoMT devices provided that the devices have proper functionality. An increase in functionality was found to increase users' perception of security and privacy. This creates more awareness of the potential risks of the use of WIoMT. It leads to trust, which finally results in the high possibility of adopting WIoMT devices, as intention to use becomes strong.		This means that, with the increase in the perceived usefulness of WIoMT devices, there is a significant increase in the security and privacy perception of such devices among consumers. There was a moderate positive correlation between perceived ease of use and perceived security and privacy, and it was significant at p < 0.001. (Table 10). This means that, with the increase in the perceived ease of use of WIoMT devices, there is a significant increase in the perceived security and privacy of such devices among consumers.The correlation analysis showed that there was a significant correlation between product- and security-related factors and intention to use					Malware infections and vulnerabilities; lack of regulation and compliance; unsecured network connectivity; lack of encryption; and lack of patching and device updates. Most of them felt "unauthorised access to data" was the most prominent risk for these devices, followed by "unsecured network connectivity" and "malware infections and vulnerabilities".	N
Community-dwelling older adults' acceptance of smartwatches for health and location tracking	2023	Int J Older People Nurs	Chung J, Brakey HR, Reeder B, Myers O, Demiris G.	10.1111/opn.12490	We aimed to examine older adults' perceptions of GPS-enabled smartwatches and to identify potential barriers and facilitators of smartwatch and sensor data use.	Mixed	USA	Older adults	Participants were required to be 60 years or older, speak and read English, not mostly bed- or wheelchair-bound. Thirty older adults participated in the study (mean age 67.4, 63% female). Twenty-one (70%) had more than a bachelor's degree. Fifty-three per cent were married or partnered. Two-thirds of participants had one or more chronic conditions, such as arthritis, hypertension or diabetes mellitus.	Fitbit Surge, a smartwatch with location tracking and other activity-tracking functions (e.g. steps, floors climbed, distance moved, heart rates) to collect life-space mobility data.				as healthy or physically active did not feel the need to use activity sensors. Nevertheless, its potential to motivate people to move more was mentioned. Participants did not seem to be fully aware of the watch's capability, thereby leading to decreased interest in smartwatch adoption. Older adults stated activity trackers would not be useful nor encourage healthier habits because functions seemed limited to tracking steps and heart rates without more in-depth health information.The interview data reveal that usefulness is dependent on lifestyle. Participants confirmed the design of display and visibility had a significant influence on acceptance.	Most were satisfied with the display (M = 5.59, SD = 1.68) and its functions (M = 5.64, SD = 1.76). Overall, participants thought the device was easy to use (M = 5.5, SD = 1.58, Table 2).	Participants identified potential uses for these data, such as monitoring level of physical activity, finding certain periods of the day for scheduling exercise, generating an alarm about a subtle change in sleep or activity patterns, tracking diet and monitoring health-related indicators.	Participants reported several physical characteristics of the device that made them feel uncomfortable or were perceived as less appealing. The uncomfortable features include a bulky design, device size and material. Sharing data with healthcare providers, family or friends Would you like to be able to turn on or off the device depending on your preference? They were satisfied with the display showing calories, steps and heart rates. However, a desire to learn about the meaning of those values in relation to their health was expressed.	However, we observed hesitancy to check the data because 'It would be disappointing (P16)' The interviews reveal several factors that might prevent them from sharing data, such as distrust in healthcare providers, provider lack of interest, concerns about inappropriate data use or sharing, or potential embarrassment due to low activity levels. Reasons for not wanting to share data with family were provided: 'I wouldn't share it with my family… Because mostly, I don't think everybody needs to know how active I am (P30)'. Another said she would not share with family because they live far away and would prevent them from being actively engaged in a caregiving role if they could know her activity level.		The potential value for measuring indicators of health (e.g. heart rate, blood pressure and diabetes) or physical activity levels was discussed.	When asked about privacy, people had mixed opinions. Overall, participants reported no major privacy issues because they thought activity trackers collect simple health data without much meaning. Regarding location tracking, there were mixed responses.	N
Acceptability and compliance with a remote monitoring system to track smoking and abstinence among young smokers	2018	Am J Drug Alcohol Abuse	McClure EA, Tomko RL, Carpenter MJ, Treiber FA, Gray KM.	10.1080/00952990.2018.1467431	This study describes a mobile system that captures smoking using breath carbon monoxide (CO) and real-time self-reports of smoking behavior. Compliance, feasibility, acceptability, and accuracy of the system were measured during a quit attempt and subsequent monitoring period.	Quantitative	USA	Young smokers	± 2.0 years old. The majority were female (75%), White (88%),with the remaining 12% being African American/Black, and 6% being Hispanic or Latino. 100% unmarried, 100% partner smoked, 75% lives with other smokers. 44% in college. 75% employed. 75% less than 25K	A mobile system that captures smoking using breath carbon monoxide (CO) and real-time self-reports of smoking behavior.			Overall, responses were positive regarding the app and use of the CO monitor.		The app was easy to use.					Monitoring my CO was helpful during my quit attempt.The app made me feel like I was supported in my quit attempt.The app improved my confidence in quitting smoking.The app made me feel in control of my smoking.		N
Questionnaire results of user experiences with wearable exoskeletons and their preferences for sensory feedback	2018	Journal of NeuroEngineering and Rehabilitation	Muijzer-Witteveen H, Sibum N, van Dijsseldonk R, Keijsers N, van Asseldonk E.	10.1186/s12984-018-0445-0	This study investigates the preferences of users of an exoskeleton	Quantitative	Netherlands	exoskeleton users	5 male, 5 female,	The ReWalk wearable exoskeleton with sensors						The respondents who preferred discrete feedback commented that with discrete feedback the focus can be more on other things instead of the feedback and that continuous feedback can become annoying after a while. On the other hand the respondents who preferred the continuous feedback commented that you can always feel the feedback and won't miss important changes when you are distracted for a while.	respondents commented that it might also be useful to provide feedback when the weight shift is too large, because she was afraid to fall when moving too much, while usually the movements were not large enough to create enough space for a step. The option to provide feedback when you exceed a safety boundary was also mentioned by another respondent at the last open question. The fact that you don't know exactly what your body position is, was mentioned twice as a reason to prefer weight shift feedback of both types (mediolateral and anteroposterior): "It is really peculiar that you feel that something goes wrong, but you don't know why".Foot position feedback and feedback about the different gait phases (in continuous or discrete way) were not considered useful by the majority of the respondents. As mentioned by two of the respondents, a feedback signal for each gait phase might even easily become annoying. According to one of the respondents the gait phase feedback could be helpful to get used to the rhythm of the exoskeleton, which would however not be needed anymore after a certain amount of training time.			Three of the feedback parameters were considered helpful by more than half of the respondents: feedback about 1) the step initiation, feedback about the 2) mediolateral and 3) anteroposterior weight shift (6 or more votes for "a little" or "a lot"). Feedback about the weight shift was also indicated as helpful by most of the respondents, in which the mediolateral weight shift received 1 vote more than the anteroposterior weight shift (7 to 6 votes respectively).		N
In-hospital and home-based long-term monitoring of focal epilepsy with a wearable electroencephalographic device: Diagnostic yield and user experience	2023	Epilepsia	Macea J, Bhagubai M, Broux V, De Vos M, Van Paesschen W.	10.1111/epi.17517	The aim is to report the performance of an electroencephalogram (EEG) seizure-detector algorithm on data obtained with a wearable device (WD) in patients with focal refractory epilepsy and their experience.	Quantitative	Belgium	Patients with focal refractory epilepsy	The median age of the inpatients was 30 years (interquartile range [IR] = 26–35), with a median age of seizure onset of 13 years (IR = 9–23).7/16 female. The median age for ourpatient was 38 years (IR = 28–48), with a median age of seizure onset of 20 years (IR = 11–24). 8/16 female.	An electroencephalogram (EEG) seizure-detector algorithm on data obtained with a wearable device (WD)					After using the patches and SD, patients reported a median score of 7 (IR = 6–8) for future daily usability (future usability: zero = very unlikely, 10 = very likely) for up to 4 weeks. The worriedness about how they looked wearing the device had a median score of 3 and a maximum of 5 (zero = completely not worried, 10 = completely worried). They considered the SD and patches comfortable to use during the day (median score = 7; 0 = very uncomfortable, 10 = very comfortable) and during the night (median score = 8, IR = 6–8).			The reasons for dropping out included side effects in four of five cases, being the main reason in three of five cases. One patient lost interest after 2 months of measurements, and another found that the system interfered with his daily work routine and stopped his participation in the study.			Five more adverse events occurred, namely skin irritation and patch imprinting, which did not preclude the completion of the study.Also, one patient with a history of migraine reported headaches when using the patch.	W.V.P. and M.D.V. have consultancy agreements with Byteflies, outside the scope of this research. None of the other authors has any conflict of interest to disclose. We confirm that we have read the Journal's position on issues involved in ethical publication and affirm that this report is consistent with those guidelines.
Statistical Study of User Perception of Smart Homes during Vital Signal Monitoring with an Energy-Saving Algorithm	2022	International Journal of Environmental Research and Public Health	Del-Valle-Soto C, Nolazco-Flores JA, Del Puerto-Flores JA, Vel醊quez R, Valdivia LJ, Rosas-Caro J, Visconti P.	10.3390/ijerph19169966	This work highlights the benefits of a sensor network implemented in the homes of a group of users between 55 and 75 years old, which encompasses a simple home energy optimization algorithm based on user behavior. We analyze variables related to vital signs to establish users' comfort and tranquility thresholds. We statistically study the perception of security that users exhibit, differentiating between men and women, examining how it affects the person's development at home, as well as the reactivity of the sensor algorithm, to optimize its performance.	Qualitative	Mexico	Older adults	(20 women and 20 men) aged between 55 and 75 years old to participate in our research.	Smart home with vital signal monitoring			95% are satisfied with the system.		They may think that the system is complex. However, they did not need to do any installation or configuration.					Specific parameters make her life easier and more comfortable. The results show that 90% think that the monitoring system has Effectiveness. 92.5% think that the system has Efficiency.		N
Wearable Sensor-Based Exercise Biofeedback for Orthopaedic Rehabilitation: A Mixed Methods User Evaluation of a Prototype System	2019	SENSORS	Argent R, Slevin P, Bevilacqua A, Neligan M, Daly A, Caulfield B.	10.3390/s19020432	This study sought to evaluate an exemplar sensor-based biofeedback system, investigating the feasibility, usability, perceived impact and user experience of using the platform.	Mixed	Ireland	Patients during orthopaedic Rehabilitation	15 patients volunteered to participate in the study (nine females, six males; age: 63 [standard deviation (SD): 8.32]). 86.6% married, 46.4% live with spouse, 40% live with family; 73.3% degree educated; Mobile Phone 100% Smart Phone 86.6% Tablet 66.6% WiFi 93.3% Health/Fitness App 26.6%	Exercise biofeedback system for orthopaedic rehabilitation in the home.					The system achieved a mean SUS score of 90.8 (SD 7.8).	Participants reported a motivation for using the system, whether as a result of the monitoring aspect, or from the enjoyment they achieved from using it and improving their self-efficacy.	classification and felt this was an integral part of the support the system provided. A large number of participants (n = 9) requested that additional exercises were included in the system, including progressions beyond the basic regime. Participants also felt that given the specific outcomes and targets following knee replacement surgery, a measurement of their joint angle would be beneficial. Other suggestions were based on some of the usability issues discussed previously, with requests for greater clarity in the progress reports, a quality score after each exercise session and improving the graphical interfaces within the system. Finally, a small number of participants talked about gamification ideas, yet there were mixed opinions on the relevance and the benefit of incorporating gamified features and customisation within the system	Some usability issues were highlighted by participants however, with many reporting inconsistencies in the repetition counter and the technique feedback. Participants also noted that the information presented in the progress section was not displayed intuitively and, on a number of occasions, a bug led to the exercises not being recorded correctly		Almost all participants made a reference to the system improving their adherence to the rehabilitation programme, whether that was in the quantity of exercises performed, or the quality with which they completed them. the system gave them an added incentive to complete their exercises, and provided them with support once they were at home:		N
Views of wheelchair users and caregivers regarding a passive safety monitoring system for electric powered wheelchair operators with cognitive impairment	2022	Assistive Technology The Official Journal of RESNA	Kearns WD, Becker AJ, Condon JP, Molinari V, Hanson A, Conover W, Fozard JL.	10.1080/10400435.2019.1689537	The opinions of cognitively intact current wheelchair users and their professional caregivers were solicited to explore acceptability of the concept of a passive electric wheelchair-mounted movement monitor to track driving safety and cognitive impairment.	Mixed	USA	wheelchair users and carers	Nine electric wheelchair users and 8 caregivers were recruited. The wheelchair users group was comprised of 3 men and 6 women; the caregivers' group was comprised of 1 man and 7 women.	The Passive Safety Monitoring Wheelchair System			8/8 caregivers and 5/7 wheelchair users said that they would recommend the system.			The fourth question was "What would be critical circumstances that would make you decide to recommend the use of, or want to use, the Passive Safety Monitoring Wheelchair System?" The majority of the caregivers (N = 5) indicated safety, but two gave responses suggesting need for autonomy ("A person who struggles to walk but still wants to have the independence to do things"). The majority (N = 5) of wheelchair users focused much more on declining health circumstances (e.g., "failing mental capacity", "stroke", etc.).	Both sets of participants, the wheelchair users and caregivers, believe safety is a major issue, both in terms of the device making wheelchair use safe (Personal use) but also not injuring anyone when it is in use (Safety of others). Both groups agreed that such a device needed to be simple, unobtrusive, easy to use, weatherproof, and reliable. Both groups also stressed marketing the device through mobility stores, catalogs of safety items, medical equipment stores, online stores (e.g., Amazon), and advocacy groups (e.g., AARP). Both groups also wanted the device covered by insurance, and as mentioned in Design and Legal/Ethical Issues, the device should not be sold separately but as a regular part of the wheelchair or industry standard. Both groups preferred a 'straightforward' advertising approach, talking about user safety and independent living.Each group was willing to recommend the device if it were developed, but CG FGs were willing to pay significantly more for it than wheelchair users. A number of practical issues for both the WCUs and the CGs, such as how rules would be communicated, how liability was framed, and who was responsible for maintenance and assistance, were identified. However, questions on how the monitoring device would communicate to the user also were raised. The fifth question was "If you had one thing you could change about the Passive Safety Monitoring Wheelchair System, what would that be?" Design issues were predominant. The caregivers were quite variable in their recommendations, suggesting pressure sensors on seats "to track where they actually go", the need to make it mandatory, the	The third question was "If the Passive Safety Monitoring Wheelchair System evaluated by the group was available at the price you selected in Question #2, but NOT paid for by third party insurance, how likely would you be to purchase it out of pocket or recommend its purchase to another?" Six caregivers endorsed 'Probably' and two caregivers said 'Probably Not'. The responses were much more variable among the wheelchair users, with 2 respondents indicating 'Definitely', 2 indicating 'Probably', 2 indicating 'Probably Not', and 1 indicating 'Definitely Not'.			This category had three subthemes: Privacy, Autonomy, and Liability. WCU FGs mentioned their concerns that a family member or a person with power of attorney may misuse this information to reduce their autonomy and mobility. Both groups were especially concerned about the legal ramifications of using the data to prevent wheelchair users from operating their powered wheelchair. Liability (individual and/or facility) issues were either plainly stated as above or contextualized around cognitive status and safety.	Professors Kearns and Fozard are co-inventors of US Patent #7978085-1 "Human and Physical Asset Movement Pattern Analyzer", which is described in this research.
Care4AD: A Technology-Driven Platform for Care Coordination and Management: Acceptability Study in Dementia	2023	Gerontology	Mishra RK, Park C, Momin AS, Rafaei NE, Kunik M, York MK, Najafi B.	10.1159/000526219	This study aims to assess the acceptability and feasibility of a technology-driven platform to facilitate care coordination platform, Care4AD, from the end-user perspective.	Qualitative	USA	Individuals with dementia	14 caregivers (age: 68 ± 10.2, 12 female) 10 IWD (age: 73.4 ± 10.2, 4 female)	Care4AD includes three components: (1) Care4AD app: the app is used by caregivers to coordinate care, monitor physical activity, and schedule reminders; (2) Care4AD tablet: a smart tablet is used by the care recipient to display scheduled reminders; and (3) Care4AD tags: a series of wireless sensor tags attached to various objects of daily care to facilitate monitoring instrumental activities of daily living (IADL) and adherence to scheduled tasks.	The majority of caregivers mentioned that they were not aware of any technology and used pen and paper to coordinate with IWD.		All stakeholders rated favorably towards using the platform	The only significant predictor for attitude towards the use among caregivers was perceived ease of use. Moreover, attitude towards the use was not significantly correlated with age and education for the caregivers. For IWD only age was a significant predictor of attitude towards use (online suppl. Fig. 1). Furthermore, attitude towards use was not significantly correlated with perceived ease of use, perceived usefulness, and education for IWD.			The platform should be connected with myChart so that managing appointments will become easier for me. One common way used by caregivers and IWD to coordinate is through handwritten notes. On similar lines, two caregivers (43-year-old female and 71-year-old female) and an IWD (73-year-old male) suggested including a note section in the platform. Additionally, a caregiver (71-year-old female) suggested to add a "safety card" with necessary info for a patient, such as "don't shower until nurse arrives"; and create a separate tasks list that does require assistance from a caregiver or aid. Including a section for notes can be useful for the caregivers and IWD to manage crucial information related to safety and contact details and better coordinate the tasks remotely.			perceived usefulness." IWD found the Care4AD useful, as it can help them to be more independent. Additionally, voice instructions will help IWD to know what IWD is supposed to do, as often IWD get confused about the steps IWD should take in response to a regular alarm." Another caregiver (59-year-old female) said, "Being able to remotely track activity within the home, daily activities, and medication will help me? To understand how IWD is doing and better manage my? Caregiving responsibilities as I? Often lives away from IWD." On a similar note, a caregiver (53-year-old female) mentioned, "I want my mother to socialize more and improve her quality of life by going to all activities and training available to her, so I think that the Care4AD platform will be a good tool to help my mom remember all the tasks/activities she needs to complete per day." We asked the caregivers if they were aware of or using any technology to better coordinate with IWD and manage caregiving responsibilities.One caregiver (53-year-old female) said, "If my mother has a tool like Care4AD, then I would be a little more trusting for my mom to complete more tasks without the assistance of hospice." Another caregiver (72-year-old female)	There were no major concerns related to data sharing.	Bijan Najafi, the coauthor, is serving as a consultant for BioSensics LLC. However, his consultation is not relevant to the scope of this study. He was also not involved in data analysis from this study. A part of the algorithms described in this study is protected by a patent-pending application, held by Baylor College of Medicine. Bijan Najafi is one of the coinventors of this patent.
Does Being Ill Improve Acceptance of Medical Technology?-A Patient Survey with the Technology Usage Inventory	2021	Int J Environ Res Public Health	Safi S, Danzer G, Raha S, Nassar E, Hufert FT, Schmailzl KJG.	10.3390/ijerph18179367	We studied this acceptance by hypothesizing that current and former COVID-19 patients would be more likely to accept an electrocardiogram (ECG) "patch" (attached to the chest) that allows continuous monitoring of the heart than individuals who did not have the disease and thus the respective experience.	Quantitative	Germany	General public	A total of 607 participants took part in the study, including 155 men (25.5%). The COVID group included 130 participants, of whom 70 (53.7%) were male. The COVID-recovered group consisted of 127 participants, of whom 59 (46.5%) were male. Group 3, in which participants had no personal current or previous experience with COVID-19, consisted of 350 participants, of whom 26 (7.4%) were male. Group 3 had a significantly higher proportion of study participants in the 18–24 and 25–34 age groups, whereas Groups 1 and 2 had a higher proportion of participants in the 35–44 and 45–54 age groups, respectively (p < 0.001).Significant differences between groups existed in terms of marital status, smoking status, education level, and place of residence, in addition to gender and age. Groups 1 and 2 were relatively similar to each other and different from Group 3, but the groups did not differ with respect to the	Electrocardiogram (ECG) "patch" (attached to the chest) that allows continuous monitoring of the heart				Although it is important to mention that the sociodemographic variety between Groups 1–3 had some influence on the responses to the surveyed questions, in the majority of cases, the demographic characteristics did not have a significant impact on the participants' answers. This should be taken into account when interpreting the group differences. The presence of various comorbidities, such as hypertension, respiratory disease, diabetes mellitus, and cardiovascular disease, had a significant influence on some, but not all, of the responses and generally increased the response tendencies of COVID-affected and recovered individuals. Scores for both curiosity about the technology and interest in using it were highest in the COVID group and lowest in the healthy group. Conversely, an opposite tendency was found for fear of technology and skepticism toward technology. The COVID group also had the highest expectations concerning ease of use (p = 0.003), accessibility (p = 0.001), and usefulness (p = 0.005) of the ECG patch. Here, too, the recovered study participants had values that lay		the COVID-19 pandemic could be a major reason for ambulatory use of medical devices to monitor their health status	All three groups tended to agree with the statement that it was important for sensory equipment to be aesthetically pleasing or as unobtrusive as possible.				Interestingly, it was the recovered group that was most willing to share contact tracing data with government agencies while having the least concern about data security (p = 0.028 and p = 0.001, Figure 9 and Figure 10, respectively). The healthy group was least likely to agree that medical records should be available to treating physicians, but again the group differences were not significan. The differences in these two statements reached statistical significance. The recovered group was also most likely to agree that patients should make private co-payments for such sensor technology, but these differences were not significant (p = 0.176, Figure 11).	N
Embodiment of Wearable Technology: Qualitative Longitudinal Study	2020	JMIR Mhealth Uhealth	Nelson EC, Sools AM, Vollenbroek-Hutten MMR, Verhagen T, Noordzij ML.	10.2196/16973	In one of the first qualitative studies to track wearables in a longitudinal investigation, we explore whether a wearable can be embodied similar to a physical prosthesis. We hoped to gain insights and compare the phases of embodiment (ie, initial adjustment to the prosthesis) and the psychological responses (ie, accept the prosthesis as part of their body) between wearables and limb prostheses. This approach allowed us to find out whether this pattern was part of a cyclical (ie, period of different usage intensity) or asymptotic (ie, abandonment of the technology) pattern.	Qualitative	Netherlands	Employee	Of the 43 participants, 12 (mean age 35 years, SD 6.5 years, range 25-50 years; five female and seven male participants)	Wearables, specifically the Jawbone UP Move, which consists of a wristband and mobile app, as well as a web platform with login	Most of the participants were experiencing a wearable for the first time (10 out of 12).				The wearable in this study was quite small compared with others on the market, so neither women nor men complained about its bulkiness. In general, participants in this study described the device as "comfortable." The level of perceived attractiveness seemed to add to the ease of wear. Most participants (n=10) found the device attractive, and many (n=9) also enjoyed being asked what the device was.	overall, we saw a process of engaging, disengaging, and reengaging over the long term. All participants experienced "surprising" and "discomforting" feedback regarding their sleep and/or activity.						N
Assessing acceptability and patient experience of a behavioral lifestyle intervention using fitbit technology in older adults to manage type 2 diabetes amid COVID-19 pandemic: A focus group study	2021	Geriatr Nurs	Jiwani R, Dennis B, Bess C, Monk S, Meyer K, Wang J, Espinoza S.	10.1016/j.gerinurse.2020.11.007	Using a semi-structured interview guide, focus groups were conducted to explore participants' acceptability and experiences following a behavioral lifestyle intervention that integrated Fitbit in overweight/obese older adults with T2D amid the COVID-19 pandemic which began during the time of this study.	Qualitative	USA	Overweight/obese older adults (65 and older)	The mean age was 72 (± 5.4) years old. Over half (56%) were female and 50% of participants were Hispanic. 44.4% married	The smartphone-based self-monitoring of their diet and physical activity (fitbit)										Participants Through the Process focused on the positive impacts, benefits, accountability, and helpfulness that Fitbit provided, such as participants entering their daily food intake facilitating adherence to the study intervention. Other examples include participants being able to see the number of calories taken in versus expended and how the nutritional breakdown of food helped them further determine which foods were better to eat over others. In addition, participants noted that Fitbit helped them to track progress, structure physical activities, create and work on goals, and to know when goals were met/unmet—all of which increased their motivation to engage in physical activity.		N
The acceptability of home monitoring technology among community-dwelling older adults and baby boomers	2008	Assist Technol	Mihailidis A, Cockburn A, Longley C, Boger J.	10.1080/10400435.2008.10131927	This pilot study investigated the willingness of two generational cohorts (current baby boomers and older adults) to accept home monitoring technology.	Mixed	Canada	People over 40	The a verageagerange for thebabyboomerswas45t o 49years,whereastheaverageage r a nge fort heolde radults was 70 to 74yea rs. most postsecondary school, live with family and own house.	HMTs and sensing systems.			Baby boomers favor health physiological monitoring type A, while older adults favor personal emergency response systems.		positive about technology's appearance and video cameras	Perceived usefulness, perceived need, the availability and preferences of alternative technologies, perceived impracticality and exhaustion of alternatives	Older adults would like sensors to be fitted in kitchen where they perceived great risks, while baby boomers no pereference.				Forbabyboomer s ,th er ewere severa lconcernssurroundingsensingsyste ms concerning aper-ceive dinvasionofprivacy,particularly inthebed-r oom andbathroom. olderadultswerealsoconcernedwiththelackofh umantouchandassistance,invasionofprivacywi thcameras,user-friendlinessofthetechnology,andeducationgea redtowardolderadults'learningabilities.Thesef actorswerealsothemostprominentinthisstudy. Throughtheex-aminationoftheprosandconsidentified forthedi fferenttechnologiesasindicatorsofthequalities babyboomersandolderadultsvaluethemost,gen eralprinciplescanbeextrapolatedforthefu-turedesignanddeploymentofHMT. Thesethemesincludethefollowing:	UNDECLARED
The Unfolding Space Glove: A Wearable Spatio-Visual to Haptic Sensory Substitution			Kilian J, Neugebauer A,		This paper documents the design, implementation and evaluation of the Unfolding Space Glove—an open source sensory substitution device. It transmits the relative position and distance of nearby objects as vibratory stimuli to the back of the hand and thus enables blind people to haptically explore the depth of their surrounding space, assisting with navigation tasks such as object recognition and wayfinding.				The age of the remaining participants (six female, five male, one not specified) averaged 45 ± 16.65 years and ranged from 25 to 72 years. All were healthy—apart from visual impairments—and stated that they were able to assess and perform the physical	Unfolding Space Glove—an open	None had prior experience with the Unfolding Space Glove or other				The SSD achieved an average SUS of 50 in this study, while the cane scored quite similarly at 53. As expected, the cane performed slightly better in the blind group (54) than in the sighted (52), while the SSD performed better in the sighted (51) than		The two biggest wishes addressed two well-known problems of the prototype: detection of objects close to the ground (e.g., steps, thresholds, unevenness, …) was requested by five subjects and the detection of objects closer than 10 cm (where the prototype currently cannot measure and display anything) was requested by four of them. Additionally, subjects (number in brackets) wished that they did not have to wear a battery on their arm (3) and wished that the device was generally more comfortable (2). Some individuals mentioned that they would like to customise the configuration (e.g., adjust the range). Some wished for the detection of certain objects (e.g., stairs) or characteristics of the room (brightness/darkness) to be communicated to them via	The cane is described by some as easy to learn but therefore less challenging and less fun On the other hand, subjects also report that they feel safer and more comfortable with the cane. Learning		more spatial awareness is possible; one can survey a		We declare that Siegfried Wahl is scientist at the University of Tübingen and employee of Carl Zeiss Vision International GmbH, as detailed in the affiliations. There were no conflict of interest regarding this study. The funders had no role in the design of the study; in the collection, analyses, or interpretation of data; in the writing

Older adults' perceptions of wearable technology hip protectors: implications for further research and development strategies	2019	Disabil Rehabil Assist Technol	Hall A, Boulton E, Stanmore E.	10.1080/17483107.2018.1491647	Hip fractures are an important public health issue. Ninety-five percent of hip fractures are caused by falls, with those at greatest risk including residents of long-term care facilities. Hip protectors can be effective in preventing hip fractures, but levels of acceptance and adherence may be low. We report on work to develop research into a new hip protector that aims to overcome some of the acceptance and adherence challenges.	Qualitative	UK	older adults and care sector staff	In total, we included the views of 147 older adults and 10 care sector staff. patients 35 female, 11 male, age range 55–96 staff 8 female, 2 male, age range 39–52	wearable technology hip protectors the 'Assist' aspect of the product refers to an embedded electronic sensor system on a single chip (processor, memory and tri-axial accelerometer, algorithms developed by BioSensics, Boston, MA).			The majority of participants thought the Fall-Safe Assist system was a good idea.			When asked about the 'tipping point' for agreeing to wear the hip protectors, many participants stated that this would be after their first fall, as esthetic concerns would be overridden by the desire to protect themselves from further physical damage. Some also mentioned that another tipping point might be if people had returned home from hospital. A staff member thought that this point might be an optimal time to encourage use of the hip protectors, as people are often physically weak and need to regain strength and independence. Some participants living in the specialist housing facilities were largely happy with their existing pendant-based alarm system, but could see the potential for wearing the Fall-Safe Assist hip protectors outdoors, where the pendant system did not work.	Each group advocated for choice and variety of different styles of underwear. Some female participants thought that the underwear looked like men's boxer shorts, and suggested adaptations such as different colors, patterns, lace, or other feminizing factors. Others suggested using different material that could act as 'control' underwear, or a 'girdle'. Some male participants expressed a preference for a 'fly' or open front version. The idea of having an open front was discussed at length in two of the groups and was thought to be a good idea for women too, as it would make putting them on and taking them off much easier. A hook and loop fastening was preferred to a button, as manual dexterity was often an issue.	However, many participants said that they themselves would not wear the hip protectors. Some proffered an innate dislike of any assistive technology, either because it felt unnatural or because it represented an unwelcome indication of ageing.A small number of participants thought that the hip protectors would not help them because their falls had been onto their knees, or in another way that had not affected their hips. Others stated that they were not at risk of falls, and had not yet reached the point at which they felt that falls were a concern. A major concern expressed at all events was the reliance on a mobile phone for the Fall-Safe Assist system to work. Within the specialist housing facilities, the mobile phone and internet reception were reported to be very poor, with text messages sent within and outside the building at times taking over 24 h to be received. cost. the style of the accompanying underwear was unappealing. There were also concerns about the compatibility of the hip protectors and underwear with other assistive equipment, such as incontinence pads.Participants reminded us that increasing age was not necessarily accompanied by disinterest in style of underwear.	Staff members also thought that they would have a better chance of encouraging older adults to try the hip protectors on a one-to-one basis, as opposed to in a group setting. Participants reflected on perceived low levels of understanding about hip fractures amongst their peer group. They suggested that if greater numbers of older adults understood the potential adverse effects of a hip fracture on their physical and mental health and quality of life, more people would likely accept the hip protectors as a preventative measure.	wearing the hip protectors might provide reassurance and help them to feel more confident moving around.		N
Usability Experience of a Personal Sleep Monitoring Device to Self-manage Sleep Among Persons 65 Years or Older With Self-reported Sleep Disturbances	2022	Comput Inform Nurs	LeBlanc RG, Czarnecki P, Howard J, Jacelon CS, Marquard J.	10.1097/CIN.0000000000000911	The purpose of this study was to describe qualitatively the experience of using a personal sleep monitoring device for sleep self-management in adults 65 years or older with self-reported sleep disturbances.	Qualitative	USA	Older adults (over 65) with sleeping disturbances	Twenty-six people, 12 males, 14 females with a Mean age of 72 (SD=4.99) participated in the study.	Personal Sleep Monitoring Device	The participant users were strong in their ability to use the device based on learning. While several participants were curious about the device, they felt they understood how it worked enough to monitor their sleep.				The PSMD did have some issues with battery life and participants found this confusing. Negative performance expectancy was related to the function of the device and perceived inaccuracies that mediated their trust in the device.Ease of use was identified as a facilitating condition. Battery life and needing to change the small battery frequently were issues that occurred commonly for study participants. This was described as a significant drawback within the sub-theme of component difficulty. While participants in this study were provided the device for free and had the option of keeping the PSMD after the study ended, the device was specifically selected because of prior reviews of its accuracy and its low cost, thus wider possible access.	This idea that someone "else" may be watching or monitoring them was described in interacting with the device but also as a possibility in being able to easily share with a health care provider, for example, their sleep health information. Participants' intentional use was to self-monitor, to watch themselves, to know more about their own sleep patterns, as a way of understanding sleep disturbances as part of their health self-management.T	However, many participants, despite all having reported sleep disturbances, were also interested in the alternative options of activity monitoring.	For some, not having a smartphone/tablet was the obstacle to ongoing use of a PSMD. Cost as a facilitating condition was discussed and most participants felt the cost was a big issue in considering using a PSMD and balancing that against accuracy and features. Most participants reported cost would be an issue but not all. Some also mentioned the cost of time. The time it takes to use a device and pay attention to the aspects of personal sleep self-monitoring were recognized as a condition to continue use. These open-ended interviews did suggest age was not a barrier to technology use.		The sub-theme of capability reflects how mastering the use of the device made the participant feel more capable in managing their own health. The use of the PSMD served as a reminder in attending to fundamental sleep-health practices. Improving awareness of sleep patterns and associated behaviors was described as a positive performance expectation that included evaluation of the information from the device and how the participants used it to change sleep related behavior.	Participants described this as "being monitored." They commented on how pervasive these types of technologies are and expressed a range in degree of concern over their privacy. Neutral social influence was also a more dominant theme than negative social influence. This sub-theme emphasized that participants did not feel their social influences cared one way or another about their use of the PSMD, noticed it or were aware of it. Negative social influence was described as close others ridiculing their use of the device as a negative appraisal. Additionally, when asked if they would be willing to share their sleep data with close others or monitor together there was at times the negative social threat of compromising privacy that was a concern.Concerns over the safety of their personal sleep data are described as conditions that would facilitate use or not and is related to the earlier sub-theme of privacy. Over twelve participant interviews stated data protection as a concern that was small but still present. In not having	N
Acceptability of and Willingness to Take Digital Pills by Patients, the Public, and Health Care Professionals: Qualitative Content Analysis of a Large Online Survey	2022	J Med Internet Res	Chevance A, Fortel A, Jouannin A, Denis F, Mamzer MF, Ravaud P, Sidorkiewicz S.	10.2196/25597	As recommended for the evaluation of health technologies, this study assesses the prospective acceptability of and willingness to take digital pills among patients, the public, and health care professionals.	Quantitative	France	Patient with chronic condition	Among patients, 396/767 (51.6%) were women and the mean age was 54.6 (SD 15.6) years (Table 1). Hypertension was the most reported chronic condition (169/767, 22%), followed by diabetes (135/767, 17.6%) and chronic pain (81/767, 10.6%) . patient, public Patients were adults (≥18 years old) with at least one self-reported chronic condition for >6 months and who were prescribed long-term treatment for >1 month. Public participants included adults (≥18 years old) who did not meet the eligibility criteria to be classified as patients. Health care professionals were professionals prescribing, delivering, or monitoring pharmaceutical treatments (physicians, nurses, pharmacists, and midwives).	Digital Pills			We identified 8 affective attitudes toward digital pills: enthusiasm (100/767 patients, 13%; 146/1238 public participants, 11.8%; 20/246 health care professionals, 8.1%), curiosity (141/767 patients, 18.4%; 170/1238 public participants, 13.7%; 31/246 health care professionals, 12.6%), balanced attitude (70/767 patients, 9.1%; 111/1238 public participants, 9%; 41/246 health care professionals, 16.7%), unease (32/767 patients, 4.2%; 70/1238 public participants, 5.7%; 7/246 health care professionals, 2.9%), skepticism (114/767 patients, 14.9%; 168/1238 public participants, 13.6%; 31/246 health care professionals, 5.3%); fear (56/767 patients, 7.3%; 175/1238 public participants, 14.1%; 13/246 health care professionals, 5.3%), anger (178/767 patients, 23.2%; 255/1238 public participants, 20.6%; 108/246 health care professionals, 43.9%), and disgust (7/767 patients, 0.9%; 4/1238 public participants, 0.3%; 1/246 health care professional, 0.4%).	Compared to public participants, patients had more positive reactions (316/1238, 25.5% vs 241/767, 31.4%) and less negative reactions (672/1238, 54.3% vs 387/ 767, 50.5%). Compared to patients and public participants, health care professionals expressed more negative attitudes (142/246, 57.7%) and less positive attitudes (51/246, 20.7%).	Patients and public participants mostly cited the complexity of the device (32/767, 4.2% and 46/ 1238, 3.7%, respectively) and the wearing of a cumbersome device (34/767, 4.4% and 41/1238, 3.3%, respectively) to be burdensome. Participants (62/767 patients, 8.1%; 65/1238 public participants, 5.3%) also reported the increased workload due to the processing of a large amount of data to be a problem.<br><br>Participants (12/767 patients, 1.6%; 14/1238 public participants, 1.1%; 15/246 health care professionals, 6.1%) identified contradictions in digital pills and found it unconvincing. Participants reported that taking medication seemed easier than applying a patch, activating the Bluetooth function, and ingesting the medication, especially for people who needed assistance with taking medication (eg, older people, individuals with cognitive impairment).	Participants described whether taking a digital pill aligned with their values related to technology (7/98 codes, 7%), human rights (4/98 codes, 4%), and medical practice (8/98 codes). Participants also suggested precautions to limit the unethical use of digital pills (6/98 codes).In the representative sample, 647/2005 (32.3%) participants declared that they would use a digital pill: 271/767 (35.3%) patients, 376/1238 (30.4%) public participants, and 39/246 (15.8%) health care professionals. After adjustment, willingness to take digital pills by patients and public participants was significantly associated with male sex (OR 1.98, 95% CI 1.62-2.43) and integration of currently used connected devices to monitor health daily (OR 3.42, 95% CI 2.43-4.79) or rarely (OR 1.76, 95% CI 1.30-2.40), with a dose–response relationship (Figure 3). the association of the willingness to take connected drugs by sex, age, profession, population density of the residential area ("residential area"), chronic condition, frequency of health data recording with a connected device, and ease to talk about treatments with the doctor.				improve medication adherence (7/98 codes, 7%).<br><br>Patients (105/767, 13.7%) and public participants (170/1238, 13.7%) believed that digital pills could improve follow-up. Participants identified advantages (6/98 codes, 6%), disadvantages (8/98 codes, 8%), and specific target populations for whom they considered digital pills would be beneficial (16/98 codes, 16%).<br><br>Health care professionals pointed out the precision of digital pills (15/246, 6.1%) and the ease to discuss treatment (eg, tolerance, benefits, adherence) (11/246, 4.5%) as advantages and the cost (41/246, 16.7%) as a disadvantage. They identified people with cognitive impairment (29/246, 11.8%) and chronic conditions (19/246, 7.8%) as the main target population.	Potential harms cited were adverse events due to sensor ingestion (190/767 patients, 24.8%; 415/1238 public participants, 33.5%) or radiation from wireless technology (102/767 patients, 13.3%; 212/1238 public participants, 17.1%). The underlying mechanisms cited to lead to a potential improvement in adherence included behavioral mechanisms such as a smartphone schedule reminder (96/767 patients, 12.5%; 122/1238 public participants, 9.9%) and the pressure of surveillance (77/767 patients, 10%; 141/1238 public participants, 11.4%).Participants described the pressure of surveillance as a disciplinary mechanism based on social desirability, fear of being reprimanded, fear of social disqualification as a "bad patient," and fear of shame or guilt. Therefore, participants raised concerns about a policing patient–doctor relationship and the meaning of care in the future.Regarding ethicality of the technology, patients and public participants mentioned that digital pills align with their values related to	N
Sensor-Based Passive Remote Monitoring and Discordant Values: Qualitative Study of the Experiences of Low-Income Immigrant Elders in the United States	2019	JMIR Mhealth Uhealth	Berridge C, Chan KT, Choi Y.	10.2196/11516	This study aimed to examine the experiences and insights of low-income, immigrant senior residents, family contacts, and staff of housing that offered a sensor-based passive monitoring system designed to track changes in movement around the home and trigger alerts for caregivers. The senior housing organization had been offering the QuietCare sensor system to its residents for 6 years at the time of the study. We are interested in adoption and discontinuation decisions and use over time, rather than projected acceptance. Our research question is how do cultural differences influence use and experiences with this remote monitoring technology? The study does not draw generalizable conclusions about how cultural groups interact with a given technology, but rather, it examines how values are made visible in elder care technology interactions.	Qualitative	USA	Low-Income Immigrant Elders and carers	[illegible] individuals born in the United States, Poland, Czech Republic, Puerto Rico, Peru, Malaysia, Japan, China, and South Korea. family members These were daughters, sons, granddaughters, sisters, and 1 long-term home aide born in China, Korea, Guyana, India, and the United States. All technology, housing, and social work staff who had contact with the technology participated. The 10 staff members included 3 US- and 7 foreign-born participants from Russia, China, Israel, India, and Korea. Staff members were multilingual. Language and cultural congruence between staff and the residents was often achieved. All social work staff were women (n=6), and all housing and technology staff were men (n=4).	Sensor-Based Passive Remote Monitoring QuietCare comprised 5 interconnected sensors placed in predetermined locations throughout the resident's apartment (bathroom door, bedroom door, apartment door, refrigerator, and environmental temperature sensors).				differing expectations for how one should receive care and support and reactions of hope versus skepticism that the sensors will help, different reactions to the involvement of family members as emergency contacts, and experiences of intergenerational family cultural conflict.Social workers reported that English-speaking and US-born residents adopted the technology because they had a fall history and genuinely hoped it would help them, whereas Asian-born residents were less optimistic that it would help them, accepting the monitoring system only if they had no one to depend on or little family contact.	This delayed language access presented a problem for some residents because it was alarming and confusing. Another issue social workers drew our attention to is what they called a lack of cultural awareness and humility expressed by the technology company and their representatives.	Compared with their US-born residents, Chinese- and Korean-born residents who adopted the sensor system were particularly concerned about troubling or burdening their children.This feeling that the false alarms caused them to be a burden on their children was particularly disturbing to women who were accustomed to taking care of their younger family members and did not wish to become the care recipient in the family while living away from the family in senior housing. All social workers noted that their relationship with their clients was key to getting them to adopt the technology. Chinese and Korean residents may have accepted monitoring because their social workers wanted them to have it.						N
Day and night comfort and stability on the body of four wearable devices for seizure detection: A direct user-experience	2020	Epilepsy Behav	Bruno E, Biondi A, B[illegible]tcher S, Lees S, Schulze-Bonhage A, Richardson MP and RADAR-CNS Consortium.	10.1016/j.yebeh.2020.107478	Wearable devices are progressively becoming an available tool for continuous seizure detection. Motivation to use wearables is not only driven by the accuracy and reliability of the performance but also by the form factor, comfort, and stability on the body. We collected direct feedback and device placement-related issues experienced by a cohort of people with epilepsy (PWE) to investigate to what extent available devices are nonintrusive, comfortable, and stable on the body.	Mixed	UK	person with epilepsy	Female 58 (50.4) Male 57 (49.6) Age (years) mean ± SD 35.4 ± 13.7 Marital status Single 52 (45.2) Married 31 (26.9) Cohabiting with partner 24 (20.9) Divorced 7 (6.1) Widowed 1 (0.9) Level of education (London cohort only, N=72) Secondary school 31 (43.1) Undergraduate degree 26 (36.1) Postgraduate degree (University) 15 (20.8) Employment Employed 53 (46.1) Unemployed 28 (24.3) Student 13 (11.3) Retired 14 (12.2) Other 7 (6.1)	four wearable devices for seizure detection	Experience with wearables Yes 25 (21.7) No 88 (76.5)				all the devices were considered comfortable to be worn during the day and during sleep as demonstrated by the scores obtained for two additional questions related to comfort presented with the TAM-FF. The TAM-FF mean scores for each device ranged between 1.9 ± 0.2 (E4) and 3.0 ± 1.3 points (Epilog), indicating that overall the use of the technology was considered effortless.		the importance of adding on flexibility and to the importance to easily take the device off when needed	However, more negative judgments were reserved for those devices demonstrating a lesser degree of stability on the body, independently from their position.Moreover, participants age was correlated with TAM-FF mean scores, and older participants attributed higher scores (worse judgment) compared with younger participants (p = 0.035). specific comments on wires or patch electrodes (IMEC) and electrodes glued to the skin/scalp (Epilog) were collected. The wires on the IMEC were considered restrictive for a normal everyday routine. Moreover, the prolonged IMEC use caused discomfort on the skin. Patch electrodes in general were not considered practical for daily use				Declaration of competing interest MR is supported by the NIHR Biomedical Research Centre at the South London and Maudsley Hospital; the MRC Centre for Neurodevelopmental Disorders (MR/N026063/1); the EPSRC Centre for Predictive Modelling in Healthcare (EP/N014391/1); the remaining authors have no conflicts of interest.
A battery-less and wireless wearable sensor system for identifying bed and chair exits in a pilot trial in hospitalized older people	2017	PLoS One	Shinmoto Torres RL, Visvanathan R, Abbott D, Hill KD, Ranasinghe DC.	10.1371/journal.pone.0185670	In this pilot study, we investigate a movement monitoring sensor system for identifying bed and chair exits using a wireless wearable sensor worn by hospitalized older patients. The system's acceptability was measured using two surveys with 0-10 likert scales.	Quantitative	Australia	Hospitalized older people	patients were 71 years and older, a male to female ratio of about 1 : 2, with no cognitive impairment and with the ability to mobilize independently or use a walking aid.	A battery-less and wireless wearable sensor system for identifying bed and chair exits			The pre-trial assessment of the system had an overall average score of 7.83 with partial overall scores ≥ 6.65 as shown in Fig 7 (red line). On the other hand, the post-trial assessment achieved an overall average score of 9.03 (larger outer plot with scores higher than the smaller inner plot).	[illegible] significantly different. Considering gender differences in our patient group, the difference in response among male participants were not statistically significant (P > 0.08). However, the female participants' pre and post-trial response differences for question Q2: "I believe if I wear this device I will have difficulties doing daily activities" (U = 90.5, P = 0.030), and Q3: "I am worried the equipment will not give good enough signals for the research" (U = 84, P = 0.023) were statistically significant. For all other questions, the median differences were not significant (P ≥ 0.11) although having consistently higher average scores in the post trial responses.				Analysis of the second survey shown in Fig 8 indicates an overall average score of 8.99 for all four factors (≥ 6.68 overall) of the Sensor Acceptance Model (physical activity, anxiety, equipment and privacy). The lowest score was for question E2: "I just forgot I am wearing it" in the overall, and gender based analysis, demonstrating an overall concern for the high visibility of the sensor during the trials.				N
Perceived Need and Acceptability of an App to Support Activities of Daily Living in People With Cognitive Impairment and Their Carers: Pilot Survey Study	2020	JMIR Mhealth Uhealth	Lai R, Tensil M, Kurz A, Lautenschlager NT, Diehl-Schmid J.	10.2196/16928	This pilot study included older adults with cognitive impairment and their carers, and explored the perceived needs for and acceptability of an app that was designed to be a simple assistive tool for activities of daily living. In particular, this study aimed to assess the acceptability of common app functions such as communication, reminder, navigation, and emergency tools in this population, and to compare patients' and carers' responses to them.	Mixed	Germany	People With Cognitive Impairment and Their Carers	Age (years), mean (SD, range) 74.5 (6.1, 57-84) 62.4 (16.0, 31-83)<br>Gender, n (%)<br><br>Men 8 (33) 15 (62)<br><br>Women 16 (67) 9 (38)<br>Education, > 12 years, n (%) 8 (33) 11 (46)	The app used was a pilot developed in collaboration with professionals in the fields of informatics and dementia. It used a simple interface and had four key functions: (1) simplified phone calls and voice or text messages to predefined contacts; (2) a memory aid, used to save speech and text notes as reminders, such as reminders for taking medications; (3) a one-click emergency call to the predefined carer; and (4) a simplified pedestrian navigation system. The app interface was in German.			"low." Despite this, half of the 24 carers reported that they had "high" (7/24, 29%) or "very high" (5/24, 21%) levels of preparedness for using an app, indicating their interest in using such an assistive tool. In the predemonstration interview, most patients (18/24, 75%) reported "low" interest in getting a short introduction to the app and its use, which corresponded with the 19 (19/24, 79%) carers who believed their relative's willingness and ability to use an app would be "low." These responses were given despite the fact that 10 (42%) of the 24 patients reported frequent use of aids like calendars or notebooks for everyday tasks (while 4 patients, 17%, reported using such aids "occasionally").<br><br>After the demonstration of the app, patients' willingness to try the app only changed moderately—15 (63%) of the 24 patients still reported "low" interest in trying the app despite most of them (18/24, 75%) reporting that the layout was "clear."		Of the 24 carers, 19 (79%) reported that the patient's ability to successfully use an app would be "low," while the remaining 5 carers believed it would be "moderate."					However, their perceptions about the usefulness of each function varied (see Table 3). Patients rated the reminder and emergency functions more highly than the navigation function. Interestingly, carers were more likely to feel safe if they could see the patient's location, whereas patients did not rate the usefulness of the navigation and location function highly. Surprisingly, a large proportion of patients thought that having the app in a wearable form, such as on a smartwatch, would be useful—7 (29%) of the 24 patients reported "moderate" or higher usefulness.		N
User's acceptability of OvuSense: a novel vaginal temperature sensor for prediction of the fertile period	2013	Journal of Obstetrics and Gynaecology	Papaioannou S, Aslam M, Al Wattar BH, Milnes RC, Knowles TG.	10.3109/01443615.2013.817984	OvuSense is a novel vaginal sensor that can predict the fertility window by recording and analysing the changes in intravaginal temperature records. This study aimed to determine patients 'acceptability and satisfaction after using OvuSense.	Quantitative	UK	women	(1) age between 20 and 37 years	'OvuSense' is a novel vaginal sensor that can predict the fertility window by recording and analysing the changes in intravaginal temperature records.					never forgot to insert it. Overall, how comfortable was your experience of wearing the sensor on a nightly basis?.10 extremely comfortable.How would you describe the sensor insertion and removal? 10 very easy. The majority of the participants found the usage of the reader to be very easy: 76.9% of the women said it was extremely comfortable to use the device during the night; 23.1% said it was comfortable and none said it was uncomfortable (Figure 2).<br><br>Furthermore, 76.9% found the idea of using the intravaginal reader to be very convenient and never forgot to insert it (Figure 3); 76.9% found the insertion and removal of the reader to be very easy (Figure 4) and 91.6% found cleaning the reader to be extremely easy or easy (Figure 5).The device software was very user friendly and received good feedback from the participants; none of the participants needed more than 5 s to work out what to do next (Figure 6) and 53.8% never had a problem downloading the data from the sensor to the reader. Overall, 69.2% of women were extremely satisfied using the device; 23.1% were moderately satisfied and only 7.75% were moderately dissatisfied							The authors alone are responsible for the content and writing of the paper. S. Papaioannou and T.G. Knowles are paid consultants of Fertility Focus Ltd. M. Aslam is an employee of Fertility Focus Ltd. R.C. Milnes is the CEO of Fertility Focus Ltd. B.H.AL Wattar has no conflict of interest.
In-home monitoring of older adults with vision impairment: exploring patients', caregivers' and professionals' views	2014	J Am Med Inform Assoc.	Larizza MF, Zukerman I, Bohnert F, Busija L, Bentley SA, Russell RA, Rees G.	10.1136/amiajnl-2012-001586	To develop a conceptual framework for the design of an in-home monitoring system (IMS) based on the requirements of older adults with vision impairment (VI), informal caregivers and eye-care rehabilitation professionals.	Quantitative	Ireland	older adults with vision impairment, caregivers	Most patients who completed the brainstorming, sorting/rating tasks and questionnaire were men (75.0%, 80.0% and 65.4%, respectively) and had mild (6/12–6/18) to moderate (6/19–6/60) VI. In contrast, most caregivers were women (62.5%, 57.1% and 72.0%, respectively), and all professionals were women. most married. most Secondary school completed	in-home monitoring system (IMS)			Most patients and caregivers thought that an IMS would benefit the patient in the future (62.8% and 64.0%, respectively) with only a minority nominating now (9.0% and 28.0%, respectively). Likewise, most caregivers (56.0%) believed an IMS would benefit them (the caregiver) in the future, with only a third (32.0%) thinking an IMS would benefit them now.	Compared to patients, caregivers were more likely to think that an IMS would be of benefit to the patient now or in the future (p=0.038).		An exception was the use of the bathroom for bathing (61.0% and 68.0%, respectively), which is a potentially hazardous activity. This, together with the patients' and caregivers' acceptance of monitoring presence in the house (66.2% and 60.0%, respectively) and movement around the house (58.4% and 68.0%, respectively), is consistent with the stakeholders' concerns regarding fall detection and safety—the most highly rated cluster among the clusters pertaining to services provided by an IMS.Most caregivers gave a rating from 'important' (3) to 'critical' (5) to their ability to control what information about the patient they receive and how often they receive it (84.0% and 88.0%, respectively).		Most patients (71.8%) and less than half of the caregivers (44.0%) had reasons to reject an IMS, with some reporting more than one reason. Of these, the most frequently reported reasons were 'lack of perceived need' (n=23 and n=3, respectively) and 'cost' (n=18 and n=5, respectively).		Most patients and caregivers gave the following reasons for accepting an IMS: 'detect falls or other potentially dangerous situations' (85.1% and 95.7%, respectively), 'safety' (76.1% and 91.3%, respectively), 'maintain independence' (86.6% and 56.5%, respectively; p=0.002), 'peace of mind for the family' (74.6% and 82.6%, respectively), and 'assist with visual or hearing impairments' (68.7% and 73.9%, respectively). Less than half of the patients and caregivers selected the following reasons: 'remind the patient and caregiver of upcoming appointments' (47.8% and 43.5%, respectively), 'manage medications' (43.3% and 39.1%, respectively), and 'reduce isolation' (40.3% and 21.7%, respectively).<br><br>The majority of patients and caregivers agreed that an IMS would help the older adult live a safer (70.5% and 76.0%, respectively) and more independent (69.2% and 72.0%, respectively) life, but only about half the patients and caregivers (46.2% and 52.0%, respectively) thought that monitoring the patient in the home would improve the patient's quality of life.	Patients and caregivers were more concerned with how accessible and useable the IMS would be (eg, cost, privacy, security, accuracy and ease of operation) than with the aesthetics of the system or the imposition of transmitting or receiving the information obtained from monitoring (figure 4).	N
E-health Support in People with Parkinson's Disease with Smart Glasses: A Survey of User Requirements and Expectations in the Netherlands	2015	J Parkinsons Dis	Zhao Y, Heida T, van Wegen EE, Bloem BR, van Wezel RJ.	10.3233/JPD-150568	Recent advances in smart glasses, wearable computers in the form of glasses, bring new therapeutic and monitoring possibilities for people with Parkinson's disease (PD). To provide guidelines for developers in creating new PD applications for smart glasses, a self-reported questionnaire was designed to survey the requirements, constraints, and attitudes of people with PD with respect to this new technology.	Quantitative	Netherlands	people with PD	62 persons (54.8% men and 45.2% women) responded to the survey. On average, the respondents were 65.7 ± 9.1 years old (n=61, range: 39–87) and have been diagnosed with PD for 9.5 ± 6.8 years (n=60, range: 2–36). 86.9% cohabitate with a partner, while 9.8% live alone and 3.3% reported other living arrangements (n=61). The participants represented a variety of occupations, educational background, and technical proficiency.	smart glass			how willing they were to use smart glasses in various situations on a scale of 0 to 10 (0 corresponding to completely uncomfortable and 10 to extremely comfortable). The ratings distributions for use at home (n=52), outdoors (n=51), in busy areas (n=52), and in general (n=52) were centered around 6–8 and left-skewed (i.e. the distribution is concentrated on the right), indicating that participants were overall receptive towards wearing smart glasses (Fig. 1A-D).		To predict how easily people with PD could adapt to smart glasses, we asked the participants to rate how comfortable they were in using mobile technologies (e.g. smart phones and tablets) on a scale of 0 to 10 (0 corresponding to totally uncomfortable and 10 to extremely comfortable). 57.4% of the respondents (n=61) reported a comfort level of 6 or above (Fig. 2A). These ratings showed a significant inverse correlation with the participants' age (rs=−0.45, p < 0.001) but not with the number of years they had PD (Fig. 2B-C).		We also asked participants to list the features they would like to see in smart glasses and categorized their responses into three subcategories: function, design, and applications (Table 1). First, respondents most frequently requested features for improving the quality of their gait and maintaining balance, thereby allowing them to gain more independence and confidence. Equally important was that side effects (e.g. headaches, dizziness, disorientations, eye strain, etc.) be minimal. Design wise, the control interface must be easy to operate, especially for the elderly, and allow hands-free operation. Furthermore, the device should be comfortable to wear, aesthetically pleasing, and compatible with prescription glasses. Besides the basic smart phone applications (e.g. GPS, calendar, address book, media player, etc.), the participants desired more specialized PD functions such as guide-dog like navigational guidance and object avoidance, external cueing (e.g. metronome, music, and stripes on the floor), real-time detection of motor fluctuations, warnings or instructions on how to improve performance, medication alerts, and logging of daily activities. One participant even suggested using smart glasses to provide virtual realityphysiotherapy.<br><br>We further surveyed how much people with PD were willing to pay (out of pocket) for such a device. The percentage of participants who would spend at most €100, €200, €300, €400, €500, or over €500 were respectively 17.7, 27.4, 14.5, 1.6, 17.7, and 8.1% (n=62). The	Respondents who never use mobile devices (24.6%) commented that they were more interested in other activities (n=1), had poor eyesight (n=1), disliked the sensory experience of using such devices (n=1), or did not own any mobile devices (n=1). Since smart glasses are relatively new to the consumer market, 4 participants explicitly indicated that they could not choose an exact rating while 8 others indicated that they required more hands on experience to make a precise decision. 5 participants expressed concerns about multitasking with smart glasses, namely that "people with PD can only do one thing at a time and might become disoriented… " by the extra sensory information provided by smart glasses. This was particularly worrisome in busy environments and may exacerbate their motor and cognitive disabilities.				N
Readiness among people with epilepsy to carry body-worn monitor devices in everyday life: A qualitative study	2020	Epilepsy Behav	Beck M, Simony C, Zibrandtsen I, Kjaer TW.	10.1016/j.yebeh.2020.107390	There have been intensive efforts to design and develop new wearable technology for epileptic seizure detection. Several studies have focused on the technical aspects, but the readiness of patients with epilepsy (PWEs) to use wearables in everyday life, which is crucial, remains relatively unexplored.	Qualitative	Denmark	people with epilepsy	3female, 5 male; age 21-77	body-worn monitor devices						professionals was emphasized as an important factor underlying the readiness to use wearables. The participants considered integrating wearables into their everyday lives, especially given their earlier experiences of being undiagnosed.				The participants anticipated that wearables would legitimize their symptoms.		N
Use of a real-time continuous glucose monitoring system in children and young adults on insulin pump therapy: patients' and caregivers' perception of benefit	2010	Pediatr Diabetes	Cemeroglu AP, Stone R, Kleis L, Racine MS, Postellon DC, Wood MA.	10.1111/j.1399-5448.2009.00549.x	To assess patient and caregiver's perception of benefits and disadvantages of RT-CGMS in children or young adults with type 1 diabetes mellitus (DM) on insulin pump therapy.	Quantitative	USA	children and young adults on insulin pump therapy: patients'and caregivers	Atotal of 34 patients were included in this group. Themean age was 14.6±0.9 yr and age range was 3 to 25yr. The second group consisted of patients who had beenon a RT-CGMS (Medtronic, Northridge, CA, USA orDexcom, San Diego, CA, USA) for 2 months or longer,with or without a previous trial period (LT group). Atotal of nine patients were included. The mean age was13.4±1.6 yr and age range was 7 to 21 yr.	a real-time continuous glucosemonitoring system			All subjects in the LT group reported a desireto continue using the device. In the ST group, foursubjects (12%) decided against using the device eitherintermittently or continuously, but 88% indicateda desire to use RT-CGMS continuously (72%) orintermittently (28%)					Undesirable effects(Table 2) included irritation/annoyance from the sensoralarm (48%), skin irritation (43%), interference in dailyroutine by the sensor alarm (38%), and the sensorfeeling too bulky (22%).		perceived benefit(88%), followed by elimination of hypoglycemia-relatedanxiety (83%), ease of pattern management (85%),improvement in diabetes control (80%), improvementin quality of life (78%) and ease of diabetes care (78%). Although all demonstrated a drop in their HbA1C,four of the ST subjects (12%) decided against LT use,even if covered by their insurer. All four subjects feltthe RT-CGMS improved their diabetes control, andmost agreed that it decreased fear of hypoglycemia.The most common negative effects reported in thesefour patients were irritation/annoyance from the alarmand skin irritatio		UNDECLARED

Patient acceptance of a novel electronic auto-injector device to administer recombinant human growth hormone: results from an open-label, user survey of everyday use	2007	Current Medical Research and Opinion	Dahlgren J, Veimo D, Johansson L, Bech I.	10.1185/030079907x210589	To measure the user acceptance of a electronic auto-injector device (easypod) for recombinant human growth hormone (r-hGH) administration.	Quantitative	France, Finland, Norway, Sweden, and Switzerland,	patient	were recruited to the study. Nine patients were recruited from Finland; 13 from Norway; 37 from Sweden; and two from Switzerland. The mean (SD) age of patients was 11.83 (7.57) years, their median (range) age was 11.0 (4–64) years, with most patients (59/61, 97%) aged less than 17 years and 11 of the patients aged 7 years or under. Twenty-five of the patients were female and 36 were male.Five of the patients were treatment naïve; two of the patients had experience with the cool.click needle-free device and the remainder (54) with the one.click device. The total number of nurses and physicians involved was 30	novel electronic auto-injector device to administer recombinant human growth hormone		After 15 days of use, 98% (50/51) of responders reported a 'good' or 'very good' overall impression of the device.		When asked to comment on the device, most of the positive comments focussed on the ease of use; most of the negative comments concerned the size of the device.				The pre-programmed dose feature was identified by 78% of participants as 'very useful' (39/50) and by the remainder (11/50) as 'useful'. Other features identified by the majority of patients as 'very useful' included the skin sensor, on-screen instructions, display of remaining dose, confirmation of injected dose and automatic needle attachment (Table 2). No participants considered the pre-programmed dose, the on-screen instructions or the display of remaining dose as 'not useful'.		UNDECLARED
Understanding adherence to the CardioMEMS pulmonary artery pressure monitoring system for heart failure: A qualitative study	2020	Heart Lung	Haynes SC, Pallin R, Tong K, Henderson S, Romano PS.	10.1016/j.hrtlng.2020.01.011	The CardioMEMS HF system is a remote monitoring device that allows patients to transmit pulmonary artery pressure readings to providers, who are alerted when pressures increase above the patient's normal threshold.The purpose of this study was to gain a better understanding of patient adherence to the CardioMEMS system and to compare patients' self-reported use of the system with actual adherence.	Qualitative	USA	patient with cardio diseases	Four of the 12 patients who participated in the study were female and eight were male. The median age of participants was 76 (range:45–86). The length of time with a CardioMEMS device ranged from 106 days to 1522 days, with a median of 1038 days. Eight patients identified as white, two as black, and two as Hispanic.	CardioMEMS pulmonary artery pressure monitoring system for heart failure					aiming to adhere, to the CardioMEMS system. These reasons did not vary by level of actual or self-reported adherence.Participants described their underlying motivation for using CardioMEMS as important for sustaining adherence to the device. Participants primarily described two underlying motivations for using CardioMEMS: avoiding hospitalizations and decreasing anxiety. Prior to CardioMEMS, a majority of participants reported being in the hospital frequently, a criterion for selection to receive a CardioMEMS device.			In interviews, patients suggested that CardioMEMS provided increased knowledge of their condition, deepened their insight, and allowed for more proactive behavior.		UNDECLARED
Comparison of four different mobile devices for measuring heart rate and ECG with respect to aspects of usability and acceptance by older people	2012	Applied Ergonomics	Ehmen H, Haesner M, Steinke I, Dorn M, G靚ercin M, Steinhagen-Thiessen E.	10.1016/j.apergo.2011.09.003	In the present study, the usability and acceptability of four different devices meant to be worn for the measurement of heart rate or ECG were analyzed on the basis of qualitative subjective user ratings and structured interviews of twelve older participants.	Mixed	Germany	old patients	Twelve participants were recruited for the study (4 female; 8 male; mean age 71 yrs; age range: 55–90 yrs) from the hospital and the sport health park of the Evangelisches Geriatriezentrum Berlin (EGZB).	mobile devices for measuring heart rate and ECG				No significant change in subjective rating appeared as a consequence of long term wearing of the belts in comparison to the participants' initial impressions. The fit and material of the Polar and the Garmin belt were graded as good to very good. The material of the belts was described as very comfortable and soft to wear, while the belts themselves were judged positively as being inconspicuous as well as simple to handle and use.<br><br>The subjective rankings of the two heart rate monitors showed that the majority of the participants did not feel uncomfortable during the three hours that they were wearing the belt and imagined that they could wear the belt on a daily basis without difficulty.For each of the four belts some usability problems were noticed by the expert. Some difficulties appeared to be typical problems for older adults, such as handling very small snap-fastener and clasps, whereas other problems appeared to be related to specific design aspects of an individual belt.<br><br>The majority of problems were observed during the processes of length-adjustment of the belt, closing the locking mechanism and positioning the sensors correctly.<br><br>Two of the belts (Polar, Corscience) had a single clasp to adjust the length whereas one belt had two clasps (Garmin). The Zephyr belt was not user adjustable. Most participants had problems manipulating these small devices a task demanding a high order of fine motor skill.<br><br>Similar problems arose during the process of closing the different locking			older participants to experience more problems with the manipulation of the devices.			The study was conducted by the Geriatrics Research Group, Charité – Universitätsmedizin Berlin and the department User Interface Design of Siemens Corporate Technology within the SmartSenior Project sponsored by the German Federal Ministry of Education and Research.
Usability of a wearable fall detection prototype from the perspective of older people-A real field testing approach	2019	J Clin Nurs	Thilo FJS, Hahn S, Halfens RJG, Schols JMGA.	10.1111/jocn.14599	Community-dwelling older people were involved in the testing of a fall detection device to improve its utilisation and acceptance in everyday life. Older people were involved in this research to consider their perspective during prototype development.	Qualitative	Switzerland	Older adults	A total of 15 participants with a mean age of 81 years participated in the real field testing of the fall detection prototype. Gender<br>Female 11<br>Male 4<br>Living alone 9<br>Assistance in daily living 6<br>History of fall(s) 4<br>Fear of falling 8<br>Use of walking aid 2<br>Instability during walking 6<br>Smartphone use prior to study participation 4<br>Mobile phone use prior to study participation 8<br>Internet use at home 10	a wearable fall detection prototyp				Sensor: Overall, the size, weight, thickness and the material of the body-worn waterproof sensor were experienced as being comfortable.<br><br>The battery duration of 24 to 36 hr was considered as acceptable by the participants, as they had two sensors each. Charging the sensor by simply placing it on the charging shell was considered to be very convenient App<br>4.3.1 Positive aspects<br>Overall, the app was described as being easy to manipulate and the textual content was clear, comprehensible and readable. "It (the application) was very positive, with big writing and touch buttons, and "change sensor" was clearly written in the lower part." [FgPA]<br><br>The suggested colours (green, blue, yellow and orange) of the app were evaluated as pleasant. The automatic fall alert function along with the possibility to manually deactivate an alert was judged to be crucial for use of the device in everyday life. Two to three minutes allowance time for manual deactivation was thought to be optimal, as reaction time declines with age and not disturbing contact persons without a good reason was viewed as being important.<br><br>It was considered helpful that the alert text message contained the localisation data (GPS) of the alarming person. The participants described themselves as active and on the move. Hence, the outdoor localisation seemed very important to them.		4.3.2 Aspects requiring improvement<br>Several times during real field testing, the contact person only recognised an alert hours after it had been sent and became very frightened:<br>"Two hours later, my neighbour came to my apartment very agitated and said 'I am very sorry, I didn't notice your alert. Is something wrong with you?' And I said, happily nothing happened to me; I was only testing [the fall detection device]. She was so hugely relieved…"<br>[FgPA]<br><br>Hence, it was considered crucial that the contact person who could not answer the alert should be informed by an automatic text message that the alert no longer required their attention. Additionally, the participants believed that it may not be reasonable to expect that relatives, friends or neighbours are available 24 hr a day. This is why an institutional telephone number, a specialised call centre and healthcare professionals, such as nurses, should always be on the contact person's list. It was suggested that it might also be desirable to develop an app for the contact persons, to allow them to easily validate the alert.<br><br>The participants revealed some challenges regarding the choice of a contact person. It could be burdensome for relatives to act as a contact person because they are asked to be available even during the night and	The smartphone represented a considerable barrier to the use of this fall detection device.4.2.1 Aspects requiring improvements<br>In general, several participants felt unable coping with the smartphone. They added that most of their generation would need extensive training and coaching regarding handling a smartphone, to be able to deal with it on a day to day basis. However, some participants argued that with training, it would be possible to feel more comfortable with it.<br><br>The touch screen sensitivity of the smartphone was repeatedly criticised: Either the touch was too brief, too long, too weak, too strong or several keys were activated simultaneously. Once reactivated from the standby mode, several windows of different apps were often open. This caused problems and frustration because it was difficult to find the fall detection app:Therefore, the participants suggested as follows: The smartphone should only provide the apps that are actually used by the person.<br><br>For some participants, the font size, contrast and brightness were inadequate. To enable older people with deteriorating eyesight to use a smartphone, the participants revealed the need to individualise its colour, font size, contrast or brightness.<br><br>The duration of the smartphone battery was approximately six hours, due to the continuous processing of data, which were unanimously			UNDECLARED
Body-Worn Sensors in Parkinson's Disease: Evaluating Their Acceptability to Patients	2016	Telemed J E Health	Fisher JM, Hammerla NY, Rochester L, Andras P, Walker RW.	10.1089/tmj.2015.0026	Remote monitoring of symptoms in Parkinson's disease (PD) using body-worn sensors would assist treatment decisions and evaluation of new treatments. Participants' experiences of wearing the sensors were evaluated using a Likert-style questionnaire after each phase.	Mixed	UK	PD patient	questionnaire after both study phases. The mean age of the study cohort was 69 years (range, 50–86 years), and the average duration of PD was 10 years (range, 2–26 years). Mean Mini-Mental State Examination score was 28.6 (range, 26–30). All participants wore the sensors for the duration of Phase 1; 32 did so for the entirety of Phase 2. Two patients did not complete Phase 2: 1 withdrew after 5 days (unwell) and 1 after 4 days (discomfort wearing the sensor); however, both participants completed the Phase 2 questionnaire. Of note, 10 patients did decline participation in the study; none cited unwillingness to wear the sensors as their reason for nonparticipation.	Body-Worn Sensors in Parkinson's Disease:		After completion of Phase 2, 32/34 (94.1%) participants agreed that they were willing to wear the sensors at home, and 29/34 (85.3%) participants agreed that they were willing to wear the sensors in public.		Only one participant reported a preference for keeping a symptom diary as opposed to wearing the sensors; this was the participant who withdrew due to sensor discomfort. Content analysis, performed as described above, revealed three overarching themes: "Appearance" (Table 3), "Usability" (Table 4), and "Comfort" (Table 5). "Appearance" was subdivided into "Physical properties" and "Wearing in public." Both "Usability" and "Comfort" were subdivided according to sentiment (positive or negative).						N
Wearables in real life: A qualitative study of experiences of people with epilepsy who use home seizure monitoring devices	2021	Epilepsy Behav	Olsen LS, Nielsen JM, Simon C, Kj綠 TW, Beck M.	10.1016/j.yebeh.2021.108398	To explore the experiences of people with epilepsy using wearables for home seizure monitoring.	Qualitative	Denmark	people with epilepsy	Ten participants were invited to participate; one declined the invitation due to personal reasons. Five women and four men were included in the study (Table 1). Participants ranged in age from 28 to 44 years. 5 living with partner; 4 employed	home seizure monitoring devices		Furthermore, the analysis showed that although the participants initially expressed readiness to use the wearables, they were less willing to do so after a few days of monitoring.					The visibility of the devices influenced how they experienced themselves and how they were perceived by others.	them feel affected by their condition in an existential way; hence, wearables encouraged new insights into their being-in-the-world. At the same time, wearables provide a long-awaited experience of an attempt to validate their epilepsy symptoms. This reduced uncertainty caused by the epilepsy provides comfort and hope for clarification of their experienced symptoms.		N
A survey of older Hong Kong people's perceptions of telecommunication technologies and telecare devices	2010	J Telemed Telecare	Lai CK, Chung JC, Leung NK, Wong JC, Mak DP.	10.1258/jtt.2010.090905	We investigated how older Hong Kong people perceive the application of telecommunication technologies in products that could enhance their safety at home.	Qualitative	Hong Kong, China	Older adults	The majority of the respondents were female (73%), aged 75 years or above (66%), and had received only primary level education or below (80%). About two-thirds (64%) of respondents reported that their monthly disposable income was less than HK$2000 (HK$1 = US$0.13). About half of them (49%) lived in rental housing. Regarding their living arrangements, about two-thirds (34%) lived with their spouse and/or adult children's family, while 36% lived alone. With regard to health condition, most respondents (79%) reported that they suffered from chronic disease. Eighteen percent of the respondents reported having three or more types of chronic illness. Hypertension was the chronic disease most commonly reported (63%). One-fifth of respondents reported having diabetes and/or heart disease	The telecare devices in the present study were: (1) the Personal Emergency Link Service (PELS), a 24-hour personal emergency link service; (2) a home-based non-intrusive motion monitoring system; and (3) a wearable vital signs monitoring system.	PELS has been available in Hong Kong since 1996, and most (93%) respondents reported that they had heard of it, and among them 96% knew about the functions of the PELS. In contrast, the two more recently developed telecare devices were new to many respondents.	More than 90% thought that the Motion Monitoring device and 84% the Vital Signs Monitoring Device would be useful	Regression analysis indicated that the type of service users, those aged 75 years or over, with secondary education or above, living alone, and owning their own home (with those in rental housing more likely to subscribe to PELS, $\chi2$ = 34.4, P < 0.001) were factors associated with the use of PELS. Respondents who lived alone and who were DECC in-home service users were nine times more likely to use PELS. Those who had secondary education or above were almost three times more likely to use PELS. Those aged 75 years and above were two and a half times more likely to use PELS than those aged between 65 and 74 years (Table 6). When examining the predictor variables that accounted for the likelihood of using the Motion Monitoring Device, only secondary education or above (odds ratio [OR]: 0.40, 95% confidence interval [CI]: 0.17 to 0.95) and gender (OR: 0.46, 95% CI: 0.21 to 0.99) were significant. However, this model explained only 13% of the variance.In the model that tested the predictors of the use of the Vital Signs Monitoring Device, those with three or more chronic illnesses were almost six times more likely		Reasons for willingness/unwillingness to use telecare devices<br>About one-third of respondents said they would consider using the two telecare devices developed by our project team, and provided reasons. A simple keyword analysis was performed and the responses were grouped into five main categories (Table 4). Most of those who said 'yes' to trying out the two telecare devices were attracted to the real-time monitoring function. About one-fifth mentioned health and safety reasons. The remaining respondents, although willing to try new products, raised concerns about using telecare, such as the financial implications and effectiveness.		Thirty-seven percent said they were in good health, another 37% said they had family support, and the remainder said the device was not user-friendly (10%) or that they faced financial constraints (4%) About two-thirds of the respondents indicated that they would not try the two telecare products. Apart from financial considerations and the availability of family support and other alternatives, many respondents (39% and 43% for the Motion Monitoring and Vital Signs Monitoring Devices, respectively) said they were in good health and did not require these devices. Also, one-fifth of the respondents said they would not try out the Motion Monitoring Device because of the availability of family support (21%). For the Vital Signs Monitoring Device, complicated operational procedure discouraged one-fifth of respondents (20%).			UNDECLARED
Taking a Load Off: User Perceptions of Smart Offloading Walkers for Diabetic Foot Ulcers Using the Technology Acceptance Model	2023	Sensors (Basel)	Finco MG, Cay G, Lee M, Garcia J, Salazar E, Tan TW, Armstrong DG, Najafi B.	10.3390/s23052768	People with diabetic foot ulcers (DFUs) are commonly prescribed offloading walkers, but inadequate adherence to prescribed use can be a barrier to ulcer healing. This study examined user perspectives of offloading walkers to provide insight on ways to help promote adherence.	Qualitative	USA	Diabetes patients	A total of 21 adults with DFUs (age 61.5 ± 11.8 years; 85.7% male) were randomized to use an iRCW (n = 10), oRCW (n = 6), or sRCW (n = 5). Participant characteristics by ethnicity and fall status are depicted in Table 1. People who identified as Hispanic or Latino, compared to those who did not, had significantly higher cadence (Table 1). Fallers, compared to non-fallers, had significantly higher T-scores on PROMIS-Cognitive Function and PROMIS-Depression items, which is interpreted as having higher indications of cognitive function and depression	Smart Offloading Walkers for Diabetic Foot Ulcers			significant correlations with any of their self-reported TAM ratings, which are presented in Supplementary Table S1. Correlations with significance are depicted in Figure 4. Due to the high number of correlations with a p-value of p ≤ 0.05, only those with a p-value of p ≤ 0.001 are discussed.<br><br>Individuals who identified as Hispanic or Latino, compared to those who did not, reported they liked using the boot (p = 0.05) and would like to use it in the future (p = 0.04). Individuals with fewer retrospective falls reported the boot's design made them want to wear it longer (ρ = 0.65), they liked using it (ρ = 0.55), and would like to use it more in the future (ρ = 0.55) (all p < 0.001). Non-fallers, compared to fallers, reported the design of the boot made them want to wear it longer (p = 0.04) and it was easy to take on and off (p = 0.04).	Participants who used the smart boot (ρ = −0.82, p < 0.001) reported that learning how to use the boot was easy (Figure 4).				Participants who had lower cadence (ρ = 0.74, p < 0.001) or deeper ulcers (ρ = −0.55, p < 0.001) reported that the boot helped them follow physician orders (Figure 4). Participants with lower T-scores on the PROMIS-Pain Interference, indicating less pain, reported feeling more connected to their care provider (ρ = 0.66, p < 0.001) (Figure 4).Individuals who identified as Hispanic or Latino reported the boot helped with their daily activities (ρ = −0.59, p < 0.001) and looked good (ρ = −0.57, p < 0.001).		N
Multimodal Remote Home Monitoring of Lung Transplant Recipients during COVID-19 Vaccinations: Usability Pilot Study of the COVIDA Desk Incorporating Wearable Devices	2023	Medicina	Schuurmans MM, Muszynski M, Li X, Marcinkevics R, Zimmerli L, Monserrat Lopez D, Michel B, Weiss J, Hage R, Roeder M, Vogt JE, Brunschwiler T.	10.3390/medicina59030617	Remote patient monitoring (RPM) of vital signs and symptoms for lung transplant recipients (LTRs) has become increasingly relevant in many situations. Nevertheless, RPM research integrating multisensory home monitoring in LTRs is scarce. We developed a novel multisensory home monitoring device and tested it in the context of COVID-19 vaccinations.	Quantitative	Switherland	Lung Transplant Recipients	Age (years), median (range) 47.5 (19 - 69)<br>Sex (female/male), n (%) 4/6 (40%/60%)	Multimodal Remote Home Monitoring Wearable Devices		Regarding the COVIDA desk, in general, six (60%) patients indicated that they would be willing to use the device further in its present form. Four (40%) patients indicated that they would not want to continue the use. The willingness to continue using the whole COVIDA desk or the components of it was answered affirmatively by 60% of patients for the COVIDA desk and by 10, 40, 70, 80 and 100% of LTRs for the ring-oximeter, CORE temperature device, wrist-accelerometer, infrared thermometer, COVIDA application and spirometer, respectively								N
Safety assessment of a home-based telecare system for adults with developmental disabilities in Indiana: a multi-stakeholder perspective	2010	J Telemed Telecare	Brewer JL, Taber-Doughty T, Kubik S.	10.1258/jtt.2010.090902	We investigated the perceptions of people about the safety, security and privacy of a telecare monitoring system for adults with developmental disabilities living in residential settings.	Mixed	USA	adults with developmental disabilities	Clients (n = 45) were adults between 19 and 72 years of age with a mild or moderate developmental disability.	home-based telecare system							system was more of a one-way system that provided safety whereas the standard care system, which had staff on-site, was a two-way system that provided more interaction with the client and companionship. One standard care client stated that he missed the staff when they were not there. Similarly, a volunteer advocate discussed the benefit of having on-site staff for the client with 'She is a very special person; she thrives on the one-to-one conversation with the staff. She has someone she can relay her feelings to.' The clients that had the telecare system in their homes acknowledged that the people on the camera 'watched everything', and this often produced negative associations. One telecare client stated, 'they follow me, I don't like people watching me.' Another client offered a possible solution to the negative, distant experience when he stated that he would like to 'see their faces when they talk.' However, both sets of administrators responded that implementation of a telecare system had negative consequences for the staff. One telecare administrator said 'Some case managers don't consider [the telecare system] as 'staff'. They have fought against installation.' One standard care administrator said 'Direct care staff refused to work at sites [which used the telecare system]. Not sure how to fix this.'	Thirty seven percent of the telecare administrators defined the telecare system as 'extra monitoring' or 'providing an extra set of eyes', which were beneficial in the homes of clients who were non-ambulatory or non-verbal.	except for the service providers who felt more strongly that the telecare system provided more privacy. The results for security were also fairly balanced except the responses from the telecare case coordinators: only 4 of 11 agreed that the telecare system provided a secure environment. This may have been due to a lack of training and awareness because they were the group most removed from the day-to-day living of the clients. The results for safety showed no significant differences, i.e. the independent case coordinators perceived telecare to be secure, but not as safe, as standard care.Another telecare administrator gave a possible reason for staff resistance to the use of a telecare system, 'As a provider it helps us monitor staff actions also, even though it was not meant for this purpose'.	UNDECLARED
Interactive web-based breastfeeding monitoring: feasibility, usability, and acceptability	2012	J Hum Lact	Ahmed AH, Ouzzani M.	10.1177/0890334412451869	To develop an interactive Web-based breastfeeding monitoring system (LACTOR) and examine its feasibility, usability, and acceptability among breastfeeding mothers.	Mixed	USA	Mothers	Mothers' ages ranged from 20-39 years, with a mean of 29.88 (SD = 5.08). Most mothers were white (78.93%), which reflects the nature of the state population, and 50% worked as professionals. most BA or BS degree. most more than 25000 income	Interactive Web-Based Breastfeeding Monitoring		In general, mothers were very positive about the user friendliness of LACTOR. All of the mothers agreed on the usefulness of the monitoring and appreciated the interactive feature of the system and the notifications.		from 0-4 points. The total mean score for the scale was 3.35 (SD = 0.33). Ninety-two percent of the mothers thought that they did not need to learn many skills before they started to use LACTOR (mean 3.68 [SD = 0.55]) and did not need any technical support to use LACTOR (mean 3.68 [SD = 0.55]). Eighty-five percent reported that the system was easy to use (mean 3.4 [SD = 0.70]) and that it was not complex (mean 3.4 [SD = 0.82]). No mother reported any difficulty in data entry or logistical access problem. More than 70% of the mothers reported that the data entry was not time consuming, and 77% reported that it was not a burden to complete. One mother said it was a burden when she started working outside the home. Another parent said that it was time consuming in the beginning, but the system got easier and faster when she became used to it. One mother said it became easier when she started to use the mobile phone version.		Mothers also gave suggestions on how to improve the system: "The mobile LACTOR helped a lot. I would have liked to see the mobile LACTOR have a function that you can look at the diary or at least the last entered times."	Two mothers, however, expressed that it was a burden. Both had older children and suggested using the system until breastfeeding is well established: "It did become overwhelming because I have a 2-year-old, and with feeding and trying to take care of his needs, it was difficult to get to the computer to enter. I would suggest that this is a great tool until breastfeeding is established or problems are solved, but after that it might not be as useful." Mothers reported that they enjoyed their experience with the system and described it as "very good," "interesting," "nice," "great idea," and "helpful." However, the mothers said that the monitoring did not replace the face-to-face consultation with a lactation consultant.	Most of the mothers said that the monitoring was beneficial and encouraged them to continue breastfeeding.<br>One mother summarized the whole benefits of LACTOR: "It kept my baby on the right track, because by entering the data, the system told me if there was any problem and it would give me some solutions to do, and I tried these solutions, and guess what? It worked!!!!! Thanks to you!"		UNDECLARED
Acceptability and utility of, and preference for wearable activity trackers amongst non-metropolitan cancer survivors	2018	PLoS One	Hardcastle SJ, Galliott M, Lynch BM, Nguyen NH, Cohen PA, Mohan GR, Johansen NJ, Saunders C.	10.1371/journal.pone.0210039	The study purpose was to investigate the acceptability and utility of, and preference for, wearable activity trackers (WATs) amongst cancer survivors living in regional and remote areas of Western Australia.	Qualitative	Australia	non-metropolitan cancer survivors	Twenty survivors participated in the trial (26%). Sixteen (14 female and 2 male) completed the study with a mean age of 63 (SD 13) years. middle class full time job post secondary degree	wearable activity tracker	There were few issues reported concerning limited tech-knowledge even amongst the older participants			The most popular feature across all WATs was the step counting which promoted self-awareness. Trackers that were easy to sync (i.e., Fitbit) were also viewed favorably. Participants often spoke about experiencing difficulties putting the band on	The provision of prompts and feedback were the most motivational aspects of the WATs. Participants also found the automated prompts to move motivating. Several participants also referred to the feedback and praise concerning achievements as important and motivational	The accuracy of statistics was identified as a theme and related predominantly to the accuracy of the Fitbit which had a tendency to count arm movements as steps The inaccuracy of the Fitbit led to a false sense of achievement for some.	The less preferred features have been highlighted and include issues relating to the accuracy of trackers and disappointment that WATs did not effectively capture all activities.	Most participants reported that the trackers made them more conscious of their PA and SB		UNDECLARED
The Association of Falls with Instability: An Analysis of Perceptions and Expectations toward the Use of Fall Detection Devices Among Older Adults in Malaysia	2021	Front Public Health	Abdul Rahman K, Ahmad SA, Che Soh A, Ashari A, Wada C, Gopalai AA.	10.3389/fpubh.2021.612538	This study aimed to investigate the association between the prevalence of falls with instability. This study also presents a survey that explores older adults' perceptions and expectations toward fall detection devices.	Quantitative	Malaysia	older adults	A total of 336 community-dwelling older adults aged 50 years and older participated in the study. 83.3% female; 36% live with spouse, 31.3% with family member.	Fall Detection Devices	the respondents were initially asked whether have known or heard of a fall detection device, and 91.7% of them answered No.	The device was believed to provide safety and helpful in reducing the risk of falls.	device, especially among older adults who are generally technologically illiterate. their history of falls showed higher prevalence compared to those who were not interested. Similarly, respondents perceived to have instability showed a positive interest in using the fall detection device. The percentage of interest was surprisingly high among older respondents and those living with children.	The most answered feature was easy to use or user-friendly (n = 170) followed by affordable price (n = 146), accurate (n = 63), and effective in terms of fast response (n = 52). The rest of the answers are illustrated in Figure 2.			Nonetheless, respondents who answered Yes, stated that they refuse to use the device due to these reasons; they have never experienced falls (n = 17), no information on how or where to purchase the device (n = 8), and because the device is expensive (n = 2).The respondent stopped using the device after a few months because the wearable device was mentioned to be uncomfortable.	Almost 99% of 336 respondents agreed that fall detection devices could help to increase safety		N
Remote COVID-19 patient monitoring system: a qualitative evaluation	2022	BMJ Open	Oliver J, Dutch M, Rojek A, Putland M, Knott JC.	10.1136/bmjopen-2021-054601	To evaluate the feasibility and acceptability of the remote COVID-19 patient monitoring system from a patient perspective.	Qualitative	Australia	COVID-19 patients	Age (years) 45.8 36.8–61.6<br><br>Sex No. of Home Monitoring Programme patients (N) Proportion (%)<br>Male 20 43.5<br>Female 26 56.5	Home Monitoring System		All 16 participants praised the HMP. All participants recommended using the HMP, with almost all (N=14; 88%) saying they would recommend it to someone in their situation with COVID-19.		All participants said they found the HMP extremely easy to use. No issues with the pulse oximeters were noted, and all thought inputting data were extremely easy, except one participant who reported difficulty using the sliding scales on multiple electronic devices.		The most common criticism was that only a few signs (temperature and blood oxygen) were formally monitored. Monitoring a greater number and breadth of symptoms and signs was the most commonly suggested improvement, including respiratory rate, pain levels, neurological and gastrointestinal symptoms. Concerns that people with atypical, or more severe symptoms, could be missed by the HMP were expressed. Four participants mentioned that incorporating monitoring for comorbid conditions would have been an improvement. One participant suggested modifying the HMP and applying it to other conditions besides COVID-19.	One participant was frustrated by a lack of quality internet connection in her isolation facility which made it harder for her to use the HMP. Another emphasised the need for HMP clinicians to understand their importance as an information conduit when public health authorities are unresponsive.	Almost all of the participants mentioned they felt reassured knowing that medical staff were monitoring their health remotely while they were isolating with COVID-19. The benefits of having good communication with the HMP clinicians while isolating were often raised. Considerable suffering due to COVID-19 symptoms and aggravating comorbidities were often mentioned. Many participants discussed the HMP supplementing their clinical care and enabling them to isolate outside of hospital settings.		N

Factors influencing community-dwelling older adults' readiness to adopt smart home technology: A qualitative exploratory study	2021	J Adv Nurs	Dermody G, Fritz R, Glass C, Dunham M, Whitehead L.	10.1111/jan.14996	Ageing-in-place for older people could be more feasible with the support of smart home technology. Ageing in-place may maximize the independence of older adults and enhance their well-being and quality of life, while decreasing the financial burden of residential care costs, and addressing workforce shortages. However, the uptake of smart home technology is very low among older adults. Accordingly, the aim of this study was to explore factors influencing community-dwelling older adults' readiness to adopt smart home technology.	Qualitative	Australia	older adults	A total of 19 participants (aged 68–87) The average age was 78.9 years (SD= 5.79), the majority were female (73.7%) and married (63.2%) Secondary education10 (52.6)Tertiary education6 (31.6)Unemployed/Retired17 (94.4)Less than $30,00011 (57.9)Religion, n (%)Christian13 (68.4	smart home technology	Participants expressed having a basic understanding about how the smart home system works, and they were able to explain in their own words the benefits of smart homes, describing it as a method of collecting sensor data of everyday activities.		[illegible] technology included negativity related to the potential for privacy infringements and data breaches. However, perceptions transitioned organically during the latter half of the interview/focus group into recognition of the potential benefits, and how the ben-efits may outweigh some of the negative aspects. By the end of the focus group/interview while some concerns remained, there was a general acceptance of the potential usefulness of the smart homes to facilitate ageing-in- place and independence, with an awareness of potential trade-off's, such as invasion of privacy (participant 8).Participants understood that in a smart home, a clinical team would be monitoring their health status using sensor data in graph or chart form. Participants identified that those who have limited knowledge or experience with using technology may be more in-clined to reject the idea of living in a smart home, recognizing in-creased age as a barrier to learning something new. Some older adults did not acknowledge that smart home technology was already being deployed in some locations, perceiving smart home technol-ogy to be 'futuristic' (Participant 7). Several participants were un-aware of smart home technology and stated that further education and advertising tailored to the older adult would give older people a better understanding of the smart home technology and the	The sub-theme of 'support' was a positive factor influencing older adults' perceptions on adopting smart homes.Participants who had some ex-perience using personal computers or smartphones, were generally more accepting of the concept of the smart home and expressed readiness for adoption if this technology was available to them. Those who were less involved with using computers or smartphones tended to be more sceptical of the smart home, also describing greater concerns about privacy of their data		The right time to have a smart home installed depended on several factors including health status, living arrangements and family, for example, the death of a partner.		Several older adults asked about the cost of smart homes and indicated that cost was a primary concern and may be a barrier to installing smart home technology.		They saw the ad-vantages of smart home technology as an enabler for older adults to stay in their own homes, maintaining their independence for as long as possible, with less reliance on family members	Concerns were also raised about trusting clinicians to main-tain privacy.Participants expressed that security was important for feeling safe, for detecting intruders while the participant was at home or away from home and in pro-tecting health data against data breaches. Additionally, two couples were keen to know how or if the smart home could detect intruders. One couple expressed concerns about the system potentially being abused to identify when people would be away and that could create a security risk.	This study was funded by the Western Australia Nurses Memorial Charitable Trust, and the Centre for Nursing, Midwifery & Health Services Research, Edith Cowan University
Performing Dynamic Weight-Shifting Balance Exercises With a Smartphone-Based Wearable Telerehabilitation System for Home Use by Individuals With Parkinson's Disease: A Proof-of-Concept Study	2023	IEEE Trans Neural Syst Rehabil Eng	Lee BC, An J, Kim J, Lai EC.	10.1109/TNSRE.2022.3226368	Telerehabilitation technology often helps individuals with Parkinson's disease (PD) to control their balance and improve postural stability.A Technology Acceptance Model (TAM) questionnaire completed by participants validated its acceptability and use.	Quantitative	USA	Individuals With Parkinson's Disease	Age (years) 69.86 ± 11.84 Male Gender (n) 7.00 ± 0.00	a Smartphone-Based Wearable Telerehabilitation System for Home Use by Individuals With Parkinson's Disease			Notably, most of them indicated a willingness to use the SBS in the future if it was readily available (questions 7 and 8).		Participants strongly agreed on user-friendliness and ease of use while performing in-home dynamic WSBEs with the SBS (questions 1 through 4).					Most of them benefited from performing in-home dynamic WSBEs with the SBS to improve their balance stability and perform exercises regularly (questions 5 and 6).		UNDECLARED
Perceptions and Understanding of Diabetes Mellitus Technology in Adults with Type 1 or Type 2 DM: A Pilot Survey from Pakistan	2021	J Diabetes Sci Technol	Nadeem S, Siddiqi U, Martins RS, Badini K.	10.1177/19322968211011199	Diabetes mellitus technology (DMT) is increasingly used for routine management in developed countries, yet its uptake in developing countries is not as consistent. Multiple factors may influence this, including country specific patient perception regarding DMT. We conducted a pilot study in Pakistan to understand this important question which has not been studied yet.	Quantitative	Pakistan	Adults with Type 1 or Type 2 DM	A total of 40 patients with diabetes mellitus (DM) responded to our pilot survey. The greatest percentage of respondents were aged between 35-55 years (n = 16; 40%) and married (n = 30; 75%). Almost half of the respondents (n = 19; 47.5%) reported having a bachelor's degree as their highest qualification.	Diabetes Mellitus Technology				.				[illegible] diabetes care (80%) and decreases the risk of diabetic complications (55%), barriers to use included high-costs, lack of availability, and lack of awareness.However, only 5 (12.5%) respondents felt that DM technology was easily available in Pakistan, and 15 (37.5%) felt that it increased costs of DM management. The commonest barriers to a wearable technology (CGM and CSII) were that patients' diabetes care teams had never discussed wearable technology as management options (n = 16; 41.5%), patients did not like having a wearable DM device on their bodies (n = 11; 27.5%), and that patients did not like wearable DM technology due to the attention they attracted from other people (n = 10; 25%). Perceptions regarding DM technology and barriers to wearable DM technology are shown in Table 3. Of the total respondents some DM patients may not have been on insulin and on oral diabetes agents alone		Most respondents felt that DM technology improves diabetes care (n = 32; 80%) and decreases the risk of complications (n = 22; 55%).		N
Long-Term Home-Monitoring Sensor Technology in Patients with Parkinson's Disease-Acceptance and Adherence	2019	Sensors (Basel)	Camenzind M, Urwyler P, Bolliger D, Vanbellingen T, Kistler R, Bohlhalter S, M[illegible]i RM, Mosimann UP, Nef T.	10.3390/s19235169	Parkinson's disease (PD) is characterized by a highly individual disease-profile as well as fluctuating symptoms. Consequently, 24-h home monitoring in a real-world environment would be an ideal solution for precise symptom diagnostics.In a pilot study of PD patients (N = 4), adherence (wearing time) and acceptance (questionnaires) of a multi-part sensor set was evaluated over a 4-week timespan.	Quantitative	Switherland	PD patient	Four adults (2 male, 2 female; age 65–70; disease duration 10–14 years, UPDRS 11–49) were included in this study.	Long-Term Home-Monitoring Sensor Technology			All participants strongly favored the sensor set over the self-reporting method and strongly disagreed with the statement to rather use the self-reporting method over the sensor set, Q7.		The mean SUS score was at 71.5% (SD 12.8).			[illegible] sensors in public, Q8, as the score of 73.3% (SD 3.8) indicates. Patients indicate high willingness to use the sensor set over short time periods, Q9, i.e., less than 2 months (86.7% SD 23.1). The patient's willingness to use the system over longer time periods, Q10, i.e., 6 months, is lower (60% SD 34.6).				N
A qualitative study on participants' experiences with a community-based mindful walking intervention and mobile device activity measurement	2021	Complementary Therapies in Medicine	Jones KO, Lopes SS, Kelly C, Welsh RS, Chen L, Wilson M, Jindal M, Zinzow H, Zhang L, Shi L.	10.1016/j.ctim.2020.102640	Mindful walking (MW) interventions employ mindfulness training combined with physical activity. Wearable mobile devices have been increasingly used to measure outcomes of physical activity interventions. The purpose of this study was to understand MW participants' attitudes towards MW and the use of mobile devices in health promotion interventions, including barriers and facilitators of intervention engagement and adherence. Few qualitative studies have documented participant experience with these two types of interventions.	Qualitative	Unspecified	General public	For the participants included in our study, the mean age was 49.3 (SD = 13.96). About 54 % of the participants were in the 40–60 age group, 87 % were female, 74 % had BMI 25 and above, 76 % were White, and 95 % were US-born.	mHealth technology (Fitbit)			Two participants from the intervention group and two participants from the control group indicated not enjoying using the device, but admitted that the device was helpful.		14 participants from the intervention group and 154 participants from the control group clearly indicated having enjoyed using the device. Conversely, two participants from the intervention group and five participants from the control group found the device uncomfortable to wear.	Regarding the influence of the Fitbit device features on motivation to meet fitness goals, fifteen participants from the intervention group and twelve participants from the control group attributed their motivation to meet their fitness goals to the Fitbit device.		[illegible] participants were asked to describe any technical problems they had encountered during the intervention that may have influenced the accuracy of their physical activity assessment (e.g. battery charging issues, inaccurate step counts, etc.). This question elicited responses from 30 participants in total, 17 of which said they experienced no problems with the device, others reported problems with battery charging (four participants), inaccurate step count (three participants), trouble syncing with app (three participants), activity count when there was none (for example: while riding a motorcycle; one participant), inaccurate calories count (one participant), inaccurate sleep log (one participant), zig-zag motion mistaken for steps on stairs (one participant), no heart beats shown (one participant), and error messages while using the app (one participant).		In terms of assessing the helpfulness of the device in meeting physical activity goals, 16 participants from the intervention group and 16 participants from the control group reported the device helped by raising awareness about activity level "The Fitbit was a great reminder and motivator to reach my step goal."		N
Acceptance of a new non-invasive fetal monitoring system and attitude for telemedicine approaches in obstetrics: a case-control study	2018	Arch Gynecol Obstet	Schramm K, Lapert F, Nees J, Lempersz C, Oei SG, Haun MW, Maatouk I, Bruckner T, Sohn C, Schott S.	10.1007/s00404-018-4918-y	Reduction of maternal morbidity and mortality is a major worldwide objective anchored in the millennium goals of the United Nations. To improve fetal and maternal care, a constant attempt to discover groundbreaking technologies is ongoing. One approach is the enhancement of non-invasive fetal monitoring devices. Most importantly, acceptance of new technologies by pregnant women is a prerequisite for successful implementation.	Quantitative	Germany	pregnant women	the majority of participants were married women, educated to at least high school level (study subjects (A) 50.9%; aged-matched control group (B) 66.1%, groups as explained in Table 3) having a part-time profession (A 43.4%; B 39.7%) with an average monthly net income of < 2000€. Almost half of the study group had previously given birth (A 45.3%; B 47.2%) with a C-section rate of 46.2% (A), respectively, 32.3% (B) without significant differences between the groups.	non-invasive fetal monitoring system			an exceedingly positive attitude towards the device after a single exposure. .		It was categorized as comfortable (n = 51, Likert scale Ø 4.4) with an appealing design (n = 52, Ø 4.0) and without any remarks on discomfort.							SS, KS, JN, FL, CS declare no conflict of interest. Author professor Oei is gynecologist and head of the obstetric department of Máxima Medical Center and leader of the fundamental perinatology research group from Eindhoven University of Technology. From this scientific research, the described EHG device and the company Nemo Healthcare has originated. Máxima Medical Centre has a research collaboration with Nemo Healthcare. Professor Oei has no affiliations with or involvement in Nemo Healthcare with any financial interest. During the study, authors\ CL is being paid by a grant from the European Framework Research and Innovation Program 'Horizon 2020' (Grant number 719500). Nemo Healthcare, Veldhoven, the Netherlands supported the study and provided the prototype.
Heartrate variability biofeedback for migraine using a smartphone application and sensor: A randomized controlled trial	2021	Gen Hosp Psychiatry	Minen MT, Corner S, Berk T, Levitan V, Friedman S, Adhikari S, Seng EB.	10.1016/j.genhosppsych.2020.12.008	2-arm randomized trial comparing an 8-week app-based heartrate variability (HRV) biofeedback (HeartMath) to waitlist control. Feasibility/acceptability outcomes included number and duration of sessions, satisfaction, barriers and adverse events.	Quantitative	USA	patient	We enrolled 52 participants; the majority were White, Not-Hispanic and Female (Table 1). A substantial minority (38.5%, 20/52) of participants had sought care for migraine at the emergency room.	app-based HRV biofeedback (HeartMath) to waitlist control			Regarding recommending this device to others, between 21–28% of participants responded "Not likely" and "Definitely not" at any given follow-up, 29–42% reported "Neutral", and 31–43% reported "likely" or "very likely".									Dr. Minen received funding from the NIH NCCIH (K23 AT009706-01) for salary support. Ms. Corner reports no disclosures. Dr. Berk receives honoraria from Medlink neurology and is on the advisory board for Biohaven. Dr. Levitan reports no disclosures. Mr. Friedman reports no disclosures. Dr. Adhikari receives research support from the NIH and Johnson & Johnson. Dr. Seng receives research support from the NINDS (K23 NS096107 PI: Seng) and has consulted for GlaxoSmithKline, Eli Lilly, and Click Therapeutics. Dr. Seng has received travel funds from the American Psychological Association, the American Academy of Neurology, the American Association of Pain Medicine Foundation, and the American Headache Society.
A Longitudinal Study of Fitbit Usage Behavior Among College Students	2022	Cyberpsychol Behav Soc Netw	Wang C, Lizardo O, Hachen DS.	10.1089/cyber.2021.0047	Fitbit wearable devices provide users with objective data on their physical activity and sleep habits. This study evaluates how users develop their usage patterns and the key mechanisms underlying the development of such patterns.	Quantitative	USA	General public	Table 1 shows that most participants were women (52 percent), whites (65 percent), and Catholics (73 percent).	fitbit			most believed that the Fitbit data were very accurate or pretty accurate most of the time (78 percent for physical activity data and 76 percent for sleep data) and did not change their activity based on the Fitbit data (51 percent for physical activity and 88 percent for sleep).									N
Acceptability and user satisfaction of a smartphone-based, interactive blood glucose management system in women with gestational diabetes mellitus	2015	J Diabetes Sci Technol	Hirst JE, Mackillop L, Loerup L, Kevat DA, Bartlett K, Gibson O, Kenworthy Y, Levy JC, Tarassenko L, Farmer A.	10.1177/1932296814556506	The increase in gestational diabetes mellitus (GDM) is challenging maternity services. We have developed an interactive, smartphone-based, remote blood glucose (BG) monitoring system, GDm-health. The objective was to determine women's satisfaction with using the GDm-health system and their attitudes toward their diabetes care.	Mixed	UK	Women With Gestational Diabetes Mellitus	[illegible] system, 40% were nulliparous and 69% identified as white British. Body mass index (BMI) ranged from 20 to 55 kg/m2 (median 31.5, SD 8.9). There were 32 women with new diagnoses of GDM, (mean age 34.8 years, SD 5.2) with mean gestation at recruitment of 29 ± 4 weeks (mean ± SD). For the remaining 17 women with previous GDM (mean age 30.4 years, SD 4.3), mean gestation at recruitment was 18 ± 6 weeks.	a Smartphone-Based, Interactive Blood Glucose Management System			Overall, responses to the questionnaire were favorable, with no women scoring any question with the most negative scores, strongly disagree (−3) or disagree (−2).Women particularly appreciated the system if they lived far from the hospital or had other children or care commitments.		When asked specifically about the technology, the majority agreed or strongly agreed that it was convenient, with 1 woman giving a negative score (−1, slightly disagreeing that the technology was convenient).			While 48 of 49 women agreed with the statement that the equipment was reliable, 4 women provided written comments that they had problems transferring results automatically via the 3G network, due to poor local network coverage. This led 1 woman discontinuing using the system.		The relationship with the diabetes team was rated very highly overall, with 1 negative score (−1) and 3 neutral (0) scores. Comments supported these scores, indicating women felt very supported using the app, with good understanding of their diabetes, and had a positive relationship with the maternity diabetes team, particularly the diabetes midwife.		N
A qualitative evaluation of breast cancer survivors' acceptance of and preferences for consumer wearable technology activity trackers	2017	Support Care Cancer	Nguyen NH, Hadgraft NT, Moore MM, Rosenberg DE, Lynch C, Reeves MM, Lynch BM.	10.1007/s00520-017-3756-y	The purpose of the study is to explore the acceptability and usability of consumer wearable activity trackers (WATs) amongst postmenopausal breast cancer survivors.	Qualitative	Unspecified	breast cancer survivors	age 51-62	trackers from six available models (Fitbit One, Jawbone Up 24, Garmin Vivofit 2, Garmin Vivosmart, Garmin Vivoactive and Polar A300)					[illegible] "technology skilled", yet most (11 participants) did not encounter technical difficulties in operating the basic features of the tracker and its application (smartphone or web-based). In addition, many participants reported that they did not use all of the features of the trackers and mostly relied upon the step counts. Participants reported several features of WATs which were perceived as unhelpful or made them feel uncomfortable or annoyed. One of these was the auto-goal function which automatically adjusts a user's daily step goal based on their previous activity levels. Participants reported the auto-reduced goals made them less motivated compared with the fixed goals on other trackers. Two participants did not like wearing the device to bed as they found them uncomfortable (one was using the Polar A300, one the Garmin Vivofit2). Two women reported the annoyance of the buzzing indicator for sedentary time (both were referring to the Garmin Vivofit2), particularly in circumstances where participants could not alter their behaviour (e.g. while driving, seeing movies) or when they were already tired at the end of the day. Due to frustration with the buzzing trigger in such circumstances, participants gradually tended to ignore its effect.The aesthetics of the trackers were considered to be crucial in determining preference and likelihood to use. Two women reported that they would not wear the tracker going out due to the way they looked (both using the	Preferred features: Step counting was most frequently mentioned by participants as the favoured feature. In terms of prompts about SB, it appeared that vibration or buzzing was a more effective prompt than the coloured bar or beeps featured on some devices. All nine participants using devices with a buzzing feature concurred with this idea (six participants using the Garmin Vivofit2, two using the Garmin Vivosmart and one using the Jawbone Up24. Many (12 participants; six using the Garmin Vivofit2, six using the Garmin Vivosmart, two using the Fitbit One and two using the Garmin Vivoactive) liked getting real-time feedback of their behaviour on the device or on the apps, although a few preferred using the web- or computer-based feedback (two using the Fitbit One, one using the Jawbone Up24 and one using the Polar A300).	Some participants preferred trackers which had a smaller design (e.g. Fitbit One, Garmin Vivoactive, Jawbone Up 24) but also said that. The majority of participants liked the WATS that had a larger screen and clear text (Garmins and Polar) (10 out of 14 participants; eight referring to the Garmin Vivofit2 and Garmin Vivosmart, two referring to the Polar A300) which make the device easier to read than small screen or non-visual display. The convenience in operating the WATs was also very important to participants.	There were, however, two women who found it challenging to set up the device, get them to sync or understand how to use the app (both participants used the Garmin Vivoactive, one of them also used the Fitbit One). These women also felt disinterested and demonstrated a lack of readiness to incorporate new technical platforms into their existing practices. Limited technical abilities could be one potential explanation that hindered the exploration of more advanced features, which in turn might sub-optimise the effect of trackers on behavioural change.		WATs increased self-awareness and motivation of physical activity	A few participants were concerned about the accuracy of the devices, noting that some trackers had not accurately measured their activity. Some noticeable concerns participants brought up involved the potential adverse effect of these trackers on the treatment (radiation therapy), stimulation of cancer tissues and effect on physical health. Two BCS noted their concern about the potential adverse effects that WATs could have on their health.	N
Usability Evaluation of a Blood Glucose Monitoring System With a Spill-Resistant Vial, Easier Strip Handling, and Connectivity to a Mobile App: Improvement of Patient Convenience and Satisfaction	2016	J Diabetes Sci Technol.	Harvey C, Koubek R, B[illegible]at V, Jacob S.	10.1177/1932296816658058	Proper management of diabetes mellitus requires regular self-monitoring of blood glucose (SMBG). This research evaluated the usability of the Accu-Chek® Guide Meter that includes a spill-resistant vial, easier strip handling, and wireless connectivity to a mobile app.	Quantitative	USA and France	Diabetes patients	Participants were roughly evenly divided by gender. The age range was 18-69 years old with approximately 50% of the total participants falling into the 35-59 years age group. More than 70% of the participants were considered technologically adept (defined as using a smartphone and downloading at least 1 application in the past 6 months and using apps at least 1 time per week).	a Blood Glucose Monitoring System					significantly more participants agreed with the usability statement questions than disagreed.							UNDECLARED
Assessment of a Personal Interactive Carbon Monoxide Breath Sensor in People Who Smoke Cigarettes: Single-Arm Cohort Study	2020	J Med Internet Res.	Marler JD, Fujii CA, Wong KS, Galanko JA, Balbierz DJ, Utley DS.	10.2196/22811	The objective of this study is to assess the Pivot Breath Sensor in people who smoke cigarettes, evaluating changes in attitudes toward quitting smoking, changes in smoking behavior, and use experience.	Quantitative	USA	smoker	The study sample consisted of 52.6% women (123/234), had a mean age of 39.9 (SD 11.3) years, smoked a mean of 20.3 (SD 8.0) CPD at baseline, and had been smoking for a mean of 21.7 (SD 11.5) years. White 209 (89.3). US Region: South 112 (47.9) Midwest 48 (20.5) High school/General educational development 50 (21.4) Some college 107 (45.7) Associate's (2-year) degree 37 (15.8) Bachelor's (4-year) degree 25 (10.7) Employed ≥20 hours/week 194 (82.9) <US $25,000 53 (22.6) US $25,000 to US $34,999 53 (22.6) US $35,000 to US $49,999 47 (20.1) US $50,000 to US $74,999 42 (17.9)	a Personal Interactive Carbon Monoxide Breath Sensor								At 12 weeks, among the 79 participants who indicated they were not using the breath sensor at least once per day, the top 3 reasons were "I forget to keep the breath sensor with me" (25% 20/79), "I quit smoking so I don't need to sample anymore" (23%, 18/79), and "I keep the breath sensor with me but forget to use it" (20%, 16/79; Figure 15).		At 1 week, 75.3% (171/227) reported that using the breath sensor increased their motivation to quit smoking (Figure 6)At 3 weeks, the majority (>90%, >196/214) of participants indicated the breath sensor had taught them about their CO levels and smoking behavior		JM, CF, KW, and DB are employees of Carrot Inc, the developer of the Pivot Breath Sensor used in this study. They receive salary and stock options from Carrot Inc. DU is the President and CEO of Carrot Inc and an investor in the company.
DigiPrEP: A Pilot Trial to Evaluate the Feasibility, Acceptability, and Accuracy of a Digital Pill System to Measure PrEP Adherence in Men Who Have Sex With Men Who Use Substances	2022	J Acquir Immune Defic Syndr	Chai PR, Mohamed Y, Bustamante MJ, Goodman GR, Najarro J, Castillo-Mancilla J, Baez A, Bronzi O, Sullivan MC, Pereira LM, Baumgartner SL, Carnes TC, Mayer KH, Rosen RK, Boyer EW, O'Cleirigh C.	10.1097/QAI.0000000000002854	Adherence to once daily oral preexposure prophylaxis (PrEP) for HIV prevention can be challenging for men who have sex with men (MSM) with substance use. Digital pill systems (DPS) comprise a radiofrequency emitter integrated into a gelatin capsule containing PrEP, which transmits data to a wearable Reader following ingestion, thereby enabling direct, real-time adherence measurement. This study evaluated the feasibility, acceptability, and accuracy of a DPS to measure PrEP adherence.	Mixed	USA	Men Who Have Sex With Men Who Use Substances	Among the 15 completers, all participants identified as cisgender MSM with a median age of 32 years (range, 24–49 years). Participants were primarily white (67%) and non-Hispanic (73%). College degree 7 47 Graduate degree/professional 6 40 Sexual orientation Homosexual or gay 13 87 Bisexual 2 13 Relationship status Single 10 67 Domestic partnership 2 13 Married 2 13 Separated 1 7	Digital Pill			I think the digital pill is pretty awesome to be able to track when you're taking PrEP.		Most participants reported that the DPS was easy to learn and did not interfere with their daily routines.			I found one thing that could be a little bit annoying is that if the device Reader was far away from the phone…		I think that this is something that I'd recommend to others and that I like to actually tell my friends about, because I think it's how we can build awareness, and I think there's a lot of cool ways where we can use this technology for PrEP for lots of different populations that need help with that adherence, and just the general public.		N
Acceptability and Effects of Commercially Available Activity Trackers for Chronic Pain Management Among Older African American Adults	2020	Pain Med	Janevic MR, Shute V, Murphy SL, Piette JD.	10.1093/pm/pnz215	Wearable activity trackers may facilitate walking for chronic pain management. We assessed the acceptability of a commercially available tracker and three alternative modes of reporting daily steps among older adults in a low-income, urban community. We examined whether using the tracker (Fitbit ZipTM) was associated with improvements in functioning and activity.	Mixed	USA	Older African American Adults	As shown in Table 1, the mean age of participants was 70.4 years; 88% of participants were female, and all participants were African American or African American plus another race. The sample was highly educated, with 86% having completed at least some college. About two-thirds of participants reported good or very good health; the remainder reported fair or poor health. The most common chronic conditions were hypertension (94%), arthritis (77%), and low back pain (71%). The mean pain intensity (SD) was 5.2 (1.8) at baseline. There were no significant differences between treatment groups on these Married or partnered 7 (12)	Commercially Available Activity Trackers			Mean adherence to each reporting mode across participants was as follows: SMS = 79%, IVR = 68%, and sync = 68%.		In follow-up surveys, the vast majority of participants strongly agreed (61%) or agreed (36%) that the tracker was easy to use, and similar percentages strongly agreed (57%) or agreed (36%) that they would continue to track steps after the study ended (Table 2). Challenges included trouble replacing the battery (N = 4, 14%), step counts that were perceived as inaccurate (N = 3, 11%), difficulty clipping the device onto clothing (N = 3, 11%), remembering to put the device on in the morning (N = 3, 11%), getting the screen to respond to touch (one person who lacked feeling in the tip of her fingers found this especially challenging; N = 2, 7%), having the device pop out of its rubber case (N = 2, 7%), locating the step count display (N = 1, 4%), or losing the device (there were two lost devices out of 30 distributed during the study period).					many (N = 21, 75%) reported that the device was motivating (N = 19, 68%) and/or increased their awareness of their activity levels (N = 13, 46%).		N

	2016	Telemed J E Health	Boatin AA, Wylie BJ, Goldfarb I, Azevedo R, Pittel E, Ng C, Haberer JE.	10.1089/tmj.2015.0173	To test the functionality and acceptability of a wireless vital sign monitor in an inpatient obstetric unit.	Mixed	USA	pregnant women	In total, 38 participants were enrolled; 32 pregnant women wore the sensor, and six non–study nurses observed the monitoring sessions. Maternal age (years) at enrollment 33.1 ± 9.7<br>>18–34 19 (59.4%)<br>≥35 13 (40.6%)<br>Race or ethnic groupa<br>Black 1 (3.12%)<br>White (non-Hispanic) 22 (68.8%)<br>White (Hispanic) 3 (9.3%)<br>Asian 3 (9.3%)<br>Other or unknown 3 (9.3%)<br>Parity<br>0 17 (53.1%)<br>1 7 (21.9%)<br>2 8 (25%)<br>Gestational age (weeks) 39.2 ± 1<br>Body mass index (kg/m2) at enrollment 25 ± 6.6<br>Education levela<br>Some high school 2 (6.25%)<br>High school diploma 3 (9.38%)<br>Some college 4 (12.5%)	Wireless Vital Sign Monitoring		Most pregnant women and clinicians found the sensor likeable or very likeable (81% and 84%, respectively) and useful or very useful (97% and 67%, respectively).	Wireless Vital Sign Monitoring in Pregnant Women: A Functionality and Acceptability Study		The majority of pregnant women found it comfortable or very comfortable (78%), although many were neutral. Most would either wear the sensor again or have another patient wear them (78% and 83%, respectively). All assessments were either positive or neutral.The only thing is it's kind of sticky—not sure how that would feel if I would have to wear it for a longer time.		Most pregnant women reported that increased mobility was a desirable feature of wireless fetal monitoring and a reason for recommending the technology. Pregnant women also expressed some added comfort in knowing the device could extend the amount of monitoring done				Neither patients nor clinicians had any safety concerns regarding the device. Most appreciated the size of the device, and comments about discomfort were minimal:	N
Long-term patient satisfaction with implanted device remote monitoring: a comparison among different systems	2019	J Cardiovasc Med (Hagerstown)	Artico J, Zecchin M, Zorzin Fantasia A, Skerl G, Ortis B, Franco S, Albani S, Barbati G, Cristallini J, Cannata' A, Sinagra G.	10.2459/JCM.0000000000000818	Remote monitoring is an effective strategy to improve patients' outcomes and reduce hospitalization in patients with cardiac implantable electronic devices. However, data on patients' satisfaction are scarce. The aim of the current study was to assess patients' satisfaction, ease of use and impact on daily activities of the remote monitoring and to investigate whether there are differences among different devices and different manufacturers.	Quantitative	Italy	chronical patient	The mean age was 71 ± 14 years, and 355 patients (76%) were males. Living status and employment of the whole population and according to device and manufacturers are reported in Tables 1 and 2.	remote monitoring implanted device			Overall, very few patients (3.0%) were dissatisfied or were only slightly satisfied about the information received on remote monitoring both at delivery and during follow-up and 76.4% were fully satisfied by the system (question nos. 1 and 2).	Patients with a pacemaker were slightly more frequently 'fully satisfied' than patients with ICD by information received at implantation (83.1 vs. 72.9%, respectively) and during follow-up (81.7 vs. 70.7%, respectively) but more frequently missed (even if slightly) the dialogue with the doctors/nurses (52.8 vs. 72.6%, respectively) (question nos. 1, 2 and 11). .	Similarly, for nearly all patients (96.8%) the transmitter was very or quite easy to connect, switch on and keep working during follow-up (question no. 3). A change in daily activities was found in only 3.0% of patients, whereas for most patients (97.0%) remote monitoring did not affect everyday activities. light is diturbing					great sense of security, while for nearly 10% of patients it gave no (4.3%) or only a slight (5.6%) sense of security (question no. 8). According to the majority of patients (91.2%), remote monitoring made doctors'/nurses' everyday practice much/considerably easier (question no. 9). Remote monitoring was considered to have an effect on health for most patients (90.3%), while for a not negligible proportion (10%) it had no or little effect (question no. 10). Finally, only a few patients (6.2%) greatly/considerably missed the dialogue with the doctors/nurses and 27.3% slightly miss it (question no. 11).	For 0.9% of patients it could be considered a nuisance, and for only 1.1% it significantly reduced the privacy, while it did not bother or affect patients' privacy in almost all patients (99.1 and 98.9%, respectively) (question no. 7).	N
Assessing the Clinical Utility of a Wearable Device for Physiological Monitoring of Heart Rate Variability in Military Service Members with Traumatic Brain Injury	2022	Telemed J E Health	Uomoto JM, Skopp N, Jenkins-Guarnieri M, Reini J, Thomas D, Adams RJ, Tsui M, Miller SR, Scott BR, Pasquina PF.	10.1089/tmj.2021.0627	Autonomic dysfunction has been implicated as a consequence of traumatic brain injury (TBI). Heart rate variability (HRV) may be a viable measure of autonomic dysfunction that could enhance rehabilitative interventions for individuals with TBI. This pilot study sought to assess the feasibility and validity of using the Zeriscope™ platform system in a real-world clinical setting to measure HRV in active-duty service members with TBI who were participating in an intensive outpatient program.	Quantitative	USA	Military Service Members with Traumatic Brain Injury	The composition of the military service members who participated in this study is summarized in Table 1. Those who were screened (N = 25) were predominantly male (92%), married (92%), enlisted (88%), served in the army (80%) and were between the ages of 23 and 48. All but one participant had been deployed to a combat environment, with the average number of deployments across all participants being three.Age, years<br>Mean (SD) 38.2 (6.54)<br>Median 39.0<br>Range 23–48<br>Gender, n (%)<br>Male 23 (92)<br>Female 2 (8)<br>Marital status, n (%)<br>Single/never married 2 (8)<br>Married 23 (92)<br>Race, n (%)<br>White 19 (76)<br>Black or African American 4 (16)<br>Asian 1 (4)	a Wearable Device for Physiological Monitoring of Heart Rate Variability			responses suggest that participants were favorable toward the idea of receiving greater objective physiological measures when receiving treatment.		These finding suggest that from a usability standpoint, the Zeriscope device is fairly easily incorporated into the clinical environment and is associated with a reasonable level of effort to set up, record, and capture data.							The views expressed in this article are those of the authors and do not necessarily represent the official policy or position of the Defense Health Agency, Department of Defense, Department of Veterans Affairs, any other U.S. government agency, the U.S. government, or the Henry M. Jackson Foundation for the Advancement of Military Medicine, Inc. This work was prepared under Contract HT0014-21-C-0012 with the DHA Contracting Office (CO-NCR) HT0014 and, therefore, is defined as U.S. government work under Title 17 U.S.C.§101. Per Title 17 U.S.C.§105, copyright protection is not available for any work of the U.S. government. The investigators have adhered to the policies for protection of human subjects as prescribed in 45 CFR 46. For more information, please contact dha.TBICOEinfo@mail.mil. UNCLASSIFIED.
Effects of a remote patient monitoring system for patients on automated peritoneal dialysis: a randomized crossover controlled trial	2022	Int Urol Nephrol	Uchiyama K, Morimoto K, Washida N, Kusahana E, Nakayama T, Itoh T, Kasai T, Wakino S, Itoh H.	10.1007/s11255-022-03178-5	Remote patient monitoring (RPM) has contributed to improved patient-centered outcomes and prognosis in patients with end-stage renal disease on automated peritoneal dialysis (APD). However, evidence from prospective trials is lacking.The participants (n = 15; median age: 65 years; males: 10; peritoneal dialysis vintage: 6.4 ± 3.5 years) randomly received APD therapy using the Kaguya® APD system either with or without the connective use of the cloud-based RPM software Sharesource® for 12 weeks. The primary outcome was patient satisfaction assessed using a modified nine-item Treatment Satisfaction Questionnaire for Medication (TSQM-9) questionnaire. The secondary outcomes were healthcare resource consumption, the health-related quality of life (HRQOL) subscales assessed with the Kidney Disease Quality of Life-Short Form questionnaire, and clinical laboratory parameters.	Quantitative	Japan	Patients on automated peritoneal dialysis	The overall cohort of 15 participants who completed the study consisted of 10 male and 5 female patients with a median age of 65 years (range 62–67 years) and PD vintage of 6.4 ± 3.5 years.	a remote patient monitoring system			Among the subscales of TSQM-9, effectiveness and convenience were significantly higher during the APD trial with Sharesource® (64.4 ± 18.8 vs. 57.8 ± 18.8; P = 0.006; and 76.3 ± 15.4 vs. 63.3 ± 17.3; P < 0.001, respectively) (Table (Table1).1).									We have read and understood International Urology and Nephrology's policy on disclosing conflicts of interest and declare that KM and NW received salaries from an endowed chair at Baxter Ltd. and that and HI received research funding, also from Baxter Ltd.
Factors influencing behavioural intention to use a smart shoe insole in regionally based adults with diabetes: a mixed methods study	2019	Journal of Foot and Ankle Research	Macdonald EM, Perrin BM, Hyett N, Kingsley MIC.	10.1186/s13047-019-0340-3	Smart insole technologies that provide biofeedback on foot health can support foot-care in adults with diabetes. However, the factors that influence patient uptake and acceptance of this technology are unclear. Therefore, the aim of this mixed-methods study was to use an established theoretical framework to determine a model of psychosocial factors that best predicts participant intention to use smart insoles.	Mixed	Australia	Diabetes patients	The focus group had five participants (n = 4 men) aged 32 to 70 years, three with a history of previous foot ulceration, four with type 2 diabetes and four with current access to the internet.The 53 participants had a mean age of 61(12.6) years of age, with the majority of participants Australian born men and with type 2 diabetes https://jfootankleres.biomedcentral.com/articles/10.1186/s13047-019-0340-3#Tab2	smart shoe insole	Most participants reported high levels of technological self-efficacy. They identified that technology was easily accessible to them, and they were confident in their ability to use a smart insole (Table 4). While participants reported high self-efficacy with technology and with the idea of a smart insole, they queried how they would interact with the device and how the device would function (performance expectancy and effort expectancy) (Table 4).		81% of participants wanted to use the insole.			The most commonly cited reasons related to areas of performance expectancy such as the desire to prevent foot ulcerations, to assist with daily foot checks and an expectation that use of the insole would improve quality of life.	Performance expectancy: who is responsible? Locus of responsibility	Of the participants who did not want to use the insole, reasons included the belief that it would not help them, not having previously used the technology, and concerns about its cost. anxiety about using technology. Additional issues that were raised by participants were durability and cost. Participants were concerned about the longevity of the smart insoles, and whether this would add to or replace existing footwear and orthotics (Table 4).<br><br>Participants suggested financial issues should be addressed through government-subsidies, or possibly some kind of technology loan scheme, believing the device would prevent foot ulceration and hospitalisation (Table 4). Another option floated was to have health care organisations own the device, and issue them to high-risk patients for temporary use for added monitoring (Table 4).	Only 15% of respondents reported anxiety about using the system, however they felt that with training they would be able to use it.		concerns about technology intrusiveness and burden<br>Participants were concerned that the device may become intrusive if alerts were provided too frequently (effort expectancy). One participant was concerned about notifications and noise (Table 4). Participants were concerned that a smart insole might add to their daily self-management burden (effort expectancy) (Table 4).	UNDECLARED
Safety and user experience with off-label use of a flash glucose monitor (FreeStyle Libre(? 1) among very young children with type 1 diabetes mellitus	2023	J Pediatr Endocrinol Metab	Abulqasim J, Alotaibi F, Al Khalifah RA.	10.1515/jpem-2022-0452	FreeStyle Libre 1 (FGM) has been approved by the FDA for children older than 4 years. It enables noninvasive glucose monitoring without the need for finger pricking. Parents of very young children often use the device off-label. Thus, we aimed to evaluate the safety of and user experience with FreeStyle Libre® 1 and determine its impact on sleep and glycemic control.	Quantitative	Saudi Arabia	parents of very young children with type 1 diabetes mellitus (T1DM) who were less than 5 years old	e included 31 children with mean age 3.88 ± 1.10 years (youngest 1.46 years) at the time they started using FGM, the mean diabetes duration was 2.6 ± 1.6 years, and the mean duration of FGM device use was 2.3 ± 1.30 years (Table 1). Three children were using insulin pump, 11 (36.6%) were using the Miao Miao transmitter along with the FGM, and one have used Dexcom. The mean of the parents' total FHS-PYC score was 83.45 ± 18.96 points, with the mean of the behavior rating scale being 35 ± 5.48 points and the mean of the worry scale being 48.38 ± 17.29 points.<br>n (%)<br>Current age, years 6.18 ± 1.86<br>Age at starting FreeStyle Libre® 1a 3.88 ± 1.10<br>Gender, male 18 (60%)<br>Caregiver<br>Mother 27 (87.1%)<br>Father 1 (3.2%)<br>Both 3 (9.7%)	a flash glucose monitor (FreeStyle Libre® 1)			However, the parents rated their overall experience of using FreeStyle Libre® 9.12 ± 1.20 out of 10.		, ease of use for 7 (22.5%), and its convenience and ability to allow frequent glucose monitoring for 6 (19.3%).	The main reasons for using FGM were to avoid finger pricking for 16 (51.4%) parents.	Participants expressed concern about who should receive alerts from a smart insole, and in what circumstances (performance expectancy and effort expectancy). Some participants felt that their general practitioners should receive the alerts and act as 'gatekeepers', providing direction to the patient about how to respond and where to go for help (Table 4).	Three parents discontinued the use of FGM due to the following reasons: lack of alarms for hypoglycemia or hyperglycemia, the use of integrated pump sensor is better, or to prevent scarring.			For the adverse events, general dermatological conditions were observed. The most common complications were scarring at the application site reported by 18 (58%) parents, skin allergy by 16 (51.6%), itching by 15 (48%), skin discoloration by 13 (41.9%), and localized bleeding by 12 (38.7%). Some parents reported local pain, local swelling, discomfort from insertion, frequent sensor error, and frequent sensor detachment. Majority of the parents needed to use extra adhesive to keep the FGM sensor attached.	N
Measure It Super Simple (MISS) activity tracker: (re)design of a user-friendly interface and evaluation of experiences in daily life	2022	Disabil Rehabil Assist Technol	Ummels D, Braun S, Stevens A, Beekman E, Beurskens A.	10.1080/17483107.2020.1815089	The purposes of this study were, first, to (re)design the user-interface of the activity tracker known as the MOX with the help of input from elderly individuals living independently and, second, to assess the use of and experiences with the adapted Measure It Super Simple (MISS) activity tracker in daily life.	Mixed	Netherlands	Older adults	Characteristics Participants shape and user location (n=3) Participants feedback (n=17) Participants use (n=20) Gender, male, n (%) 2 (66) 6 (50) 10 (50) Age (year), median (range) 66 (66–74) 71 (66–77) 72.5 (65–85)	Measure It Super Simple (MISS) activity tracker in daily life	Knowledge of technologyTable Footnotea, median, (range) 7 (5.5–8) 7 (0–7) 6 (0–8)		Eleven (42%) participants were absolutely satisfied with the MISS activity, nine (35%) were very satisfied, four (16%) were somewhat satisfied and two (7%) participants were neutral (n = 26, 2 missing participants).		Manual was clear.<br>Help desk provided a reassuring feeling.Activity tracker was easy to use.<br>Goal setting was clear.<br>Lights on the activity tracker were not of additional value.<br>Step count and active minutes were sufficient as variables.<br>No problems wearing the activity tracker.		Preference to read data on the app.		Participants who were not motivated by the activity trackers said that they would be if they saw that they were not active enough.	into their physical activity level, six (16%) gained a little more insight, and eleven (42%) did not gain any insight. One (3%) participant increased his physical activity level much, three (11%) participants increased their physical activity level a little, and 24 (86%) did not perceive any increase. One participant (3%) noticed that he divided his activities much more over the day, six (22%) participant divided their activities a little more over the day and 21 (75%) participants did not perceive any change.	No privacy issues.<br><br>A few participants felt they had to be especially active because they knew someone could see their results.	N
An intervention study to assess potential effect and user experience of an mHealth intervention to reduce sedentary behaviour among older office workers	2019	BMJ Health Care Inform	Boerema S, van Velsen L, Hermens H.	10.1136/bmjhci-2019-100014	Office workers spend a high percentage of their time sitting, often in long periods of time. Research suggests that it is healthier to break these long bouts into shorter periods by being physically active. We evaluated the effect of a context-aware activity coach, called the PEARL app, an mHealth intervention that provides activity suggestions, based on a physical activity prediction model, consisting of past and current physical activity and digital agendas.	Quantitative	Netherlands	Office workers	age was 58.93±5.4 and seven were male. Their professions were categorised as researcher (n=5), administration (n=4), technician (n=3), (project) management (n=2) and care professional (n=1). Participants indicated to work, on average, 69% of their worktime with a PC, varying from 30% for some researchers up to 100% for some technicians.	decrease total sitting time during working hours and (2) break up long sitting periods of more than 45 min. It provides intervention messages and feedback via a smartphone application, tailored to the personal physical activity rhythm of the user and a user's personal agenda (based on calendar items in Outlook).			the user experience was rated 'acceptable' to 'good' with an overall score of 3.59 on the uMARS.		The usability was rated 'acceptable', with a score of 77.9 on the SUS. Engagement, Functionality, Aesthetics and Information were 3.08, 3.68, 3.71 and 3.88, respectively. Additionally, the average UMARS subjective quality score was 'good' (2.98).		Participants also identified that smart insole summary data should be provided to their podiatrist prior to appointments, particularly if the process was automated (Table 4). They did, however, acknowledge that health and medical professionals were busy and may not have time to review large quantities of data from individual patients and provide timely advice (Table 4).Physical activity suggestions (n=3) were appreciated, as well as the graphical user interface (n=2).	intervention, but expected their employer to pay. Finally, the UMARS perceived impact scale was rated, on average, as 3.18, indicating that participants slightly agreed that the coach increased their knowledge, attitudes and intentions.<br><br>Negative aspects mentioned by the participants mainly concerned the timing of suggestions (n=10), the size of the sensor (n=6) and losing wireless connections (n=2). Participants suggested to improve the timing of suggestions in such a way that it is not disturbing them (eg, not when being in a creative process, while lecturing or a while after		PEARL app could be of added value (n=11). They mentioned that it created awareness on the need to become more physically active during the workday and provided insight into their physical activity pattern. Additionally, it motivated them to become active if the coach indicated that they were not active enough. Most participants stated that the intervention created awareness on their physical activity and sitting behaviour (n=9) and provided insight in their physical activity pattern (n=9).		UNDECLARED
Design and Development of a Digital Weight Management Intervention (ToDAy): Qualitative Study	2020	JMIR Mhealth Uhealth	Shoneye CL, Mullan B, Begley A, Pollard CM, Jancey J, Kerr DA.	10.2196/17919	The Tailored Diet and Activity (ToDAy) study aims to build on the campaign by adding a digital intervention with the potential to provide wide-reaching, cost-effective weight management support.The ToDAy study aims to build a tailored intervention using mobile technology to improve diet and physical activity behaviours in adults with overweight and obesity. The main objectives were to identify behavior change techniques for diet and physical activity (PA) change for weight loss and explore preferences for digital intervention features that would be effective in changing diet and PA behaviors.	Qualitative	Australia	people with overweight or obesity	As 85% of the sample were women, additional recruitment strategies were employed to encourage male participants, such as offering a one-to-one telephone call and men-only focus groups. When these additional approaches were not successful, a workplace with a high proportion of males was contacted and an onsite focus group was arranged, with 14 men in attendance.Age 25-40 34 (61)<br>Gender<br>Male 24 (43)<br>Female 32 (57)<br>Ethnicity<br>Australian 47 (84)<br>Indigenous Australian 5 (10)<br>Asian 4 (7)<br>Employment status<br>Employed full time or part time 39 (70)<br>Unemployed 8 (14)<br>Retired 9 (16)<br>Household income Aus $ (US $)<br><$50,000 (<$35,695) 21 (38)<br>$50,000-$150,000 ($35,695-$107,085)	a Digital Weight Management Intervention					Participants found the mFR app to be intuitive and convenient in comparison with other digital tools or paper-based methods.		Rejected ideas included individual assessment of vitamin and mineral status and details on how much weight could be lost with a specific number of steps, as this was not consistent with evidence-based advice. Accepted ideas were the inclusion of participant food and beverage images from the mFR into the tailored dietary feedback. For PA, accepted ideas included using graphs for self-comparison of PA levels throughout the study, positive re-enforcement to acknowledge improvements in activity, and regular goal setting. There was a strong preference for visual feedback for both diet and PA. This was not feasible with text messages; therefore, feedback was primarily provided by email.			Feedback on the clinical aims highlighted gaps in their understanding of the guidelines for diet and PA. Participants agreed that personalized feedback would promote health-enhancing habits by enhancing confidence and motivation.		N
Telemetric non-contact intraocular pressure monitoring with an implanted sensor in patients with glaucoma: long-term safety report and monitoring data	2023	Br J Ophthalmol	Schmidt I, Plange N, Walter P, Koutsonas A.	10.1136/bjophthalmol-2021-319786	Glaucoma is a chronic disease that requires lifelong monitoring and treatment. However, its control is limited due to discontinuous intraocular pressure (IOP) monitoring related to the practitioners' office hours. Implantable telemetric IOP sensors have made self-measurements possible and provide important information regarding the IOP profiles of patients. However, limited long-term monitoring data are currently available.This study reports telemetric monitoring data collected by self-tonometry and automated measurements and during outpatient visits, including an analysis of one active patient with several years of follow-up. The long-term safety, tolerability and functionality were assessed in the remaining patients during the last visit.	Qualitative	Germany	patients with glaucoma	a 79- year- old man with open- angle glaucoma.	Telemetric non- contact intraocular pressure monitoring with an implanted sensor					The active patient reported easy handling of self- tonometry and did not experience long- term restrictions in activities of daily living due to the implanted sensor.							N
Acceptability and validity of using the BACtrack skyn wrist-worn transdermal alcohol concentration sensor to capture alcohol use across 28 days under naturalistic conditions - A pilot study	2023	Alcohol	Courtney JB, Russell MA, Conroy DE.	10.1016/j.alcohol.2022.11.004	Wrist-worn transdermal alcohol concentration (TAC) sensors have the potential to provide detailed information about day-level features of alcohol use but have rarely been used in field-based research or in early adulthood (i.e., 26-40 years) alcohol users. This pilot study assessed the acceptability, user burden, and validity of using the BACtrack Skyn across 28 days in individuals' natural settings.	Quantitative	USA	alcoholic patient	The sample (N = 11) was 55% female and 73% non-Hispanic white with a mean age of 31.2 ± 3.3 years. The majority of participants (64%) worked full time and 36% were graduate students. ethnicity [n (%)]a<br>White 8 (72.7%)<br>Asian 1 (9.1%)<br>Black 2 (18.2%)	BACtrack skyn wrist-worn transdermal alcohol concentration sensor			One participant dropped the study after three days due to discomfort wearing the SCRAM, which implies low device acceptability. At midterm (study day 15), Skyn acceptability was 4.4 ± 0.6 (SD) and SCRAM acceptability was 2.5 ± 0.8 out of 5.		(10) = −6.79, p < .001, d = −2.74] and for the perceived comfort [t (10) = −6.66, p < .001, d = −2.77], facilitating conditions [t (10) = −8.32, p < .001, d = −3.00], and effort expectancy [t (10) = −4.34, p < .01, d = −1.33) subscales. The 'B's/C's' for difficulty of use and physical burden were mirrored by interview responses, with themes related to issues the participants experienced due to skin discomfort, the fragility and limited durability of the Skyn, and difficulties related to battery life (reported by all 11 participants), charging, and syncing the device (see Supplemental Table 1 for details).Mean user burden for the Skyn was 0.2 ± 0.2, and it received A's (top 15% of scores, ranging from 86 to 100%) for the time and social (0.2 ± 0.2), mental and emotional (0.2 ± 0.3), and privacy (0.1 ± 0.2) subscales, received a B (next 30% of scores, ranging from 56 to 85%) for the difficulty of use (0.3 ± 0.4) subscale, and received a C (next 40% of scores, ranging from 16 to 55%) for the physical burden (0.4 ± 0.6) subscale (Suh et al., 2016).							N
Continuous glucose monitoring technology for personal use: an educational program that educates and supports the patient	2009	Diabetes Educ	Evert A, Trence D, Catton S, Huynh P.	10.1177/0145721709335467	The purpose of this article is to describe the development and implementation of an educational program for the initiation of real-time continuous glucose monitoring (CGM) technology for personal use, not 3-day CGMS diagnostic studies.	Qualitative	USA	Diabetes patients		Continuous Glucose Monitoring Technology									involves more than just patient enthusiasm or interest in a new technology. Channeling patient interest into a structured educational setting that includes the benefits and limitations of real-time CGM helps to manage patient expectation			UNDECLARED
Voice activated remote monitoring technology for heart failure patients: Study design, feasibility and observations from a pilot randomized control trial	2022	PLoS One	Shara N, Bjarnadottir MV, Falah N, Chou J, Alqutri HS, Asch FM, Anderson KM, Bennett SS, Kuhn A, Montalvo B, Sanchez O, Loveland A, Mohammed SF.	10.1371/journal.pone.0267794	Heart failure (HF) is a serious health condition, associated with high health care costs, and poor outcomes. Patient empowerment and self-care are a key component of successful HF management. The emergence of telehealth may enable providers to remotely monitor patients' statuses, support adherence to medical guidelines, improve patient wellbeing, and promote daily awareness of overall patients' health.To assess the feasibility of a voice activated technology for monitoring of HF patients, and its impact on HF clinical outcomes and health care utilization.	Quantitative	USA	heart failure patients	Age, years (mean ±SD) 54.45 ±13.02 55.30 ±12.3 52.93 ±12.16 Sex (%) Female 7 (35.0) 12 (40.0) 15 (50.0) Male 13 (65.0) 18 (60.0) 15 (50.0) Race (%) Black 15 (75.0) 22 (73.3) 25 (83.3) Asian 0 (0.0) 0 (0.0) 1 (3.3) White 5 (25.0) 7 (23.3) 3 (10.0) Ethnicity (%) Hispanic 0 (0.0) 0 (0.0) 1 (3.3) Non-Hispanic 20 (100.0) 29 (96.7) 28 (93.3) South American 0 (0.0) 0 (0.0) 1 (3.3)	Voice activated remote monitoring technology for heart failure patients					The technology comfort survey completion rate was 71% (17/20 patients).		3 participants felt answering the questionnaire daily was repetitive or redundant;1 participant had difficulty of hearing and responding to device, hence, the participant could not complete without assistance. Moreover, 13 (77%) participants said "Yes" to considering using a voice-activated personal assistant in the future to help to manage their health.					UNDECLARED
Patients' experiences with commercially available activity trackers embedded in physiotherapy treatment: a qualitative study	2020	Disabil Rehabil	Ummels D, Beekman E, Moser A, Braun SM, Beurskens AJ.	10.1080/09638288.2019.1590470	The aim of this study was to describe the experience with commercially available activity trackers embedded in the physiotherapy treatment of patients with a chronic disease.	Qualitative	Netherlands	physiotherapy treatment patient	Gender, n male (%) 7 (27)<br>Age (years), median (range) 55 (22–78)<br>Education, n (%)<br>Secondary Education 6 (26)<br>College 9 (39)<br>University 8 (35)	commercially available activity trackers	Most of the participants were unfamiliar with activity trackers and had no idea about their average cost		All participants, except one, perceived the feedback of the activity tracker as positive and pleasant		Participants expressed that activity trackers should be as easy to use as possible (e.g., one button). It was hard for them to navigate and find their data in the corresponding app or computer dashboard. Because of the technical and procedural complexity of the activity tracker, they did not try to understand it fully.The visual stimulant of achievement, for instance a growing flower or a smiley, was well received by almost all participants. It motivated them to gain a reward from the activity tracker (e.g., fully grown flower). Some appreciated that feedback was an objective measurement [illegible]	A few used the goals as a 'brake' to limit their physical activity	Older participants said that if they had received more information from their physiotherapists about using the activity tracker, they could have used them independently. Some participants consulted the physiotherapist for additional information. Almost all had to ask their partner, children, or physiotherapist for extra help.ome participants found the instructions very clear, while others said they were too vague. Many participants would like a clear step-by-step manual. One major issue was that the guides were written in English instead of the participants' mother tongue (Dutch). Most participants felt that their technical abilities were insufficient	One other difficulty in purchasing an activity tracker was the lack of information about them. The amount of effort needed to find information hindered them from buying one. When asked about how much they were willing to pay, participants said they would be willing to spend between 20–50 euros but that there should be no other costs, e.g., subscription fees. Some participants wanted to buy an activity tracker but refrained due to the perceived high costs. it was demotivating when participants noticed that the activity trackers did not measure certain activities (cycling, walking stairs).Participants found it demotivating to deal with unrealistically high goals. Participants would have liked to create an individual goal together with their physiotherapist. Some became too active for their condition, which led to physical complaints	The visual stimulant of achievement, for instance a growing flower or a smiley, was well received by almost all participants. It motivated them to gain a reward from the activity tracker (e.g., fully grown flower). Some appreciated that feedback was an objective measurement.It motivated them to gain a reward from the activity tracker (e.g., fully grown flower)	They used the feedback as a motivator to reach their goals; almost all stated that they went for an extra walk or climbed some more stairs to achieve their target. Participants also reported that they became more aware of their physical activity level in general.	The experiences with the validity and reliability of the activity trackers varied. Small movements such as household activities were sometimes not measured. In some instances the activity tracker measured activities that were not actually performed by the participants.<br><br>Participants were positive about sharing data with their family or friends. This gave them a confirmation of their physical activity level and simultaneously challenged them to be more physically active.<br><br>None expressed reticence about sharing their data with healthcare professionals, and none wished to share their data with companies, including the manufacturer of the activity tracker	N

Can Older Adult Emergency Department Patients Successfully Use the Apple Watch to Monitor Health?	2021	R I Med J (2013)	Chen K, Dandapani H, Guthrie KM, Goldberg E.		To determine usability of the Apple Watch in older adult emergency department (ED) patients after a fall.	Qualitative	USA	Older Adult Emergency Department Patients	As a result, 16 participants were recruited, of whom eight consented to participate in semi-structured interviews (Figure 1). The average participant age was 77.6 years (standard deviation 10.0 years), 2/8 were non-white, 7/8 had used a cellphone previously, and 3/8 had used a wearable device (e.g. Fitbit) previously (Table 1). 6 living alone	the Apple Watch					[illegible]		[illegible]	[illegible]		[illegible]		UNDECLARED
Real-time continuous glucose monitoring versus internet-based blood glucose monitoring in adults with type 2 diabetes: a study of treatment satisfaction	2014	Diabetes Res Clin Pract	Tang TS, Digby EM, Wright AM, Chan JH, Mazanderani AB, Ross SA, Tildesley HG, Lee AM, White AS, Tildesley HD.	10.1016/j.diabres.2014.09.050	To compare treatment satisfaction between real-time continuous glucose monitoring (RT-CGM) and internet-blood glucose monitoring (IBGM) in adults with type 2 diabetes treated with insulin.	Quantitative	Canada	Diabetes patients	Of the total sample, mean age of the sample was 60 years (SD = 9.4); mean duration of diabetes was 18 years (SD = 6.6); 59% were male (n = 19). The groups did not differ significantly on any demographic or clinical variables.	Real-time continuous glucose monitoring			The mean overall treatment satisfaction score was significantly higher for the IBGM group than the RT-CGM group (33.41 vs. 24.80, p < 0.000).		[illegible]							
Users' experience of physical activity monitoring technology in rheumatoid arthritis	2013	Musculoskeletal Care	Tierney M, Fraser A, Kennedy N.	10.1002/msc.1034	The aim of the present study was to qualitatively explore users' experiences of home monitoring of health with specific regard to physical activity monitors.	Qualitative	Unspecified	Rheumatoid Arthritis patients	Gender (female/male)8/23/111/3Age (years_x005f_	Physical Activity Monitoring Technology			Overwhelmingly, participants felt that having theirhealth monitored in the home was a positive thingand were'delighted'with it.		[illegible]					[illegible]		UNDECLARED
Acceptance and use of a smartphone application in cirrhosis	2020	Liver Int	Louissaint J, Lok AS, Fortune BE, Tapper EB.	10.1111/liv.14494	The development of cirrhosis-related smartphone applications for remote monitoring is increasing. Whether patients with cirrhosis will welcome such new technology, however, is uncertain.We prospectively enrolled patients with cirrhosis (N = 102) to determine predictors of acceptance and utilization of a smartphone application for cirrhosis management using a 12-item Technology Acceptance Model (TAM) survey.	Quantitative	USA	patients with cirrhosis	Participants had a median age of 61.3 years, were predominantly white (90.2%) and 56.9% were men (Table 1). Cirrhosis was most commonly related to alcohol use disorder (37.3%) and non-alcoholic fatty liver disease (30.4%). A history of decompensation was present in 63.7% of participants at the time of enrolment. A history of ascites and hepatic encephalopathy was present in 48 (47.1%) and 40 (39.2%) patients, respectively. The median MELD-Na was 10. Smartphone ownership was 93.1%.Caregiver 76 (74.5)	cirrhosis-related smartphone applications for remote monitoring			The remaining 91 (89.2%) patients were shown the EncephalApp© and 65 (71.4%) patients expressed willingness to download the EncephalApp©.	On univariable logistic regression, agreement to download the application was increased in the presence of an informal caregiver (OR: 4.03, 95% CI: 1.44–11.29), a history of hepatic decompensation (OR: 3.07, 95% CI: 1.20–7.85), a history of ascites (OR: 3.44, 95% CI: 1.22–9.67) or the presence of any cirrhosis-related medication (OR: 2.56, 95% CI: 1.01–6.52), in particular, the presence of an ascites-related medication (OR: 3.04, 95% CI: 1.08–8.54) (Table 4).	[illegible]			[illegible]				[illegible]
Men on the Move-Nashville: Feasibility and Acceptability of a Technology-Enhanced Physical Activity Pilot Intervention for Overweight and Obese Middle and Older Age African American Men	2018	Am J Mens Health	Dean DAL, Griffith DM, McKissic SA, Cornish EK, Johnson-Lawrence V.	10.1177/1557988316644174	Men on the Move-Nashville was a quasi-experimental, 10-week pilot physical activity intervention. A total of 40 overweight or obese African American men ages 30 to 70 (mean age = 47) enrolled in the intervention. Participants attended 8 weekly, 90-minute small group sessions with a certified personal trainer. Each session consisted of discussions aimed to educate and motivate men to be more physically active, and an exercise component aimed to increase endurance, strength, and flexibility.	Qualitative	USA	overweight or obese men	[illegible] years (SD = 9.8), but ages ranged from 31 to 67 years. Most men were either married or in a relationship (75%); 55% of men had household incomes of $50,000 or more, and 50% of the men indicated it was somewhat or very difficult to pay bills. Table 2 highlights baseline health and physical activity characteristics of participants. Over 67% of men were obese (BMI ≥ 30), and 60% had a waist circumference of 40 inches or more. Fewer than half (47.5%) of men indicated that they were confident that they could set aside 30 minutes of physical activity at least three times a week. Only 45% of men self-reported they were engaging in regular physical activity.	wearable activity trackers			Overall, participants felt they benefited from Men on the Move–Nashville in multiple ways.	There was a significant difference in age of Fitbit users compared with Fitbit nonusers, t(1) = 2.10; p < .05. Participants who successfully set up and used their activity tracker were on average 7 years younger than those who did not use their activity tracker.	[illegible]		[illegible]	[illegible]		[illegible]		[illegible]
Mobile Phone Intervention for Heart Failure in a Minority Urban County Hospital Population: Usability and Patient Perspectives	2017	Telemed J E Health	Dang S, Karanam C, G酶ez-Orozco C, G酶ez-Mar 韩 O.	10.1089/tmj.2016.0224	Chronic heart failure (HF) is a complex and costly disease. Daily weight and symptom monitoring is the cornerstone of HF management. Little information exists about feasibility of a mobile monitoring intervention among minority patients with HF. We developed and tested usability of a mobile-monitoring system in minority patients with HF in a 2:1 randomized controlled trial. We tracked usage and obtained feedback on usability and the system overall at 1, 2, and 3 months.	Quantitative	USA	heart failure patients Minority Urban County Hospital Population	Gender Male 28 (66.7) Female 14 (33.3) Race White 33 (78.6) Black 9 (21.4) Ethnicity Hispanic/Latino 32 (76.2) Non-Hispanic 10 (23.8) Marital status Marrieda 23 (54.8) Otherb 19 (45.2) Education </=High School 22 (52.4) Some college 11 (26.2) College graduate or more 9 (21.4) Employed Yes 13 (31.0) No 29 (69.0) Income per year <$10,000 1 (2.4)$10,000–29,999 5 (12.0)$30,000–49,999 3 (7.1)Disability/not working 29 (69.0)Did not want to report 4 (9.5) Own mobile phone Yes 41 (97.6) Missing/Not Applicable 1 (2.4) Use texting	Mobile Phone Intervention for Heart Failure			The scores for overall satisfaction with the program were >6.8 at all three time points, showing a high level of satisfaction overall.		[illegible]		[illegible]		[illegible]	[illegible]		[illegible]
Digital medicines for adherence support: A conceptual framework and qualitative study of adherence among chronically ill patients	2021	Health Informatics J	Castillo AF, Davis AL, Fischhoff B, Krishnamurti T.	10.1177/14604582211059463	Digital medicine programs (DMPs) are emerging technologies that use sensor-enabled medicine to detect when patients have taken their medication and then provide feedback about adherence. We use qualitative methods to understand how patients change their behavioral patterns while participating in a DMP intervention.	Qualitative	USA	chronically ill patients	Their ages ranged from 51 to 85 years (mean = 69.7), consistent with the ages of patients most affected by these chronic conditions. About two-thirds (65%) identified themselves as non-Hispanic Caucasian, 30% as Hispanic, and one as African American. Most (70%) indicated an educational level of "Some College" or higher. Participants had been using the intervention between one and 3 months and were planning to continue to use the intervention while completing the study.	[illegible] that includes (1) an ingestible transmitter co-encapsulated with medication, which sends a signal when ingested; (2) an adhesive wearable patch that detects that signal, as well as physiological measures and physical activity; and (3) information provision through a patient app and provider portal, which can be integrated with electronic medical records. The patient-facing app gives patients the ability to access immediate feedback about their medication-taking behavior.						[illegible]				[illegible]		[illegible]
How Women Use Digital Technologies for Health: Qualitative Interview and Focus Group Study	2019	J Med Internet Res	Lupton D, Maslen S.	10.2196/11481	A range of digital technologies are available to lay people to find, share, and generate health-related information. Few studies have directed attention specifically to how women are using these technologies from the diverse array available to them. Even fewer have focused on Australian women's use of digital health.The Australian Women and Digital Health Project aimed to investigate which types of digital technologies women used regularly for health-related purposes and which they found most helpful and useful. Qualitative methods-semistructured interviews and focus groups-were employed to shed light on the situated complexities of the participants' enactments of digital health technologies. The project adopted a feminist new materialism theoretical perspective, focusing on the affordances, relational connections, and affective forces that came together to open up or close off the agential capacities generated with and through these enactments.	Qualitative	Australia	women	Focus group 1 included 4 women with young children who were part of a support group for mothers living with mental health conditions (aged 25 to 30 years), focus group 2 comprised 6 women (aged 25 to 33 years) with young children, and focus group 3 included 3 women aged in their mid-to-late 50s. Of the total of 36 women involved across these Canberra participant groups, 28 identified their ancestry as Anglo-Celtic and 8 as Asian. Twenty-two participants reported university-level education, while 14 had high school or technical qualifications. This group of participants was recruited using subquotas based on age to ensure a good spread of ages: 10 women aged 18 to 40 years, 10 women aged 41 to 60 years, and 10 women aged 61 years and over. Participants ranged in age from 22 to 74 years. Two-thirds lived in major cities; one-third lived in rural or remote Australia. Twenty participants lived in	digital health technologies						[illegible]				[illegible]	[illegible]	
Implementing monitoring technologies in care homes for people with dementia: A qualitative exploration using Normalization Process Theory	2017	Int J Nurs Stud	Hall A, Wilson CB, Stanmore E, Todd C.	10.1016/j.ijnurstu.2017.04.008	Ageing societies and a rising prevalence of dementia are associated with increasing demand for care home places. Monitoring technologies (e.g. bed-monitoring systems; wearable location-tracking devices) are appealing to care homes as they may enhance safety, increase resident freedom, and reduce staff burden. However, there are ethical concerns about the use of such technologies, and it is unclear how they might be implemented to deliver their full range of potential benefits.This study explored facilitators and barriers to the implementation of monitoring technologies in care homes.	Qualitative	UK	people with dementia	[illegible] senior management, registered nurses, and care workers from day and night teams; 9 relatives; 3 residents)The mean age of staff was 39.75 years (range 21–64; SD 12.62). Most relatives and residents had been involved with the homes for between one and two years. The mean age of relatives was 55.67 years (range 41–78; SD 12.67), and of residents was 81.33 years (range 72–95; SD 8.22). The vast majority of staff, residents and relatives were of White British ethic origin, and female.	bed monitoring technologies in care homes	Staff suggested that this understanding seemed less acknowledged among relatives. However, some relatives did understand that bed-monitoring technologies were not guarantees of safety				[illegible]	[illegible]				[illegible]	[illegible]	
Efficacy, Safety, and Satisfaction with the Accu-Chek Insight with Diabeloop Closed-Loop System in Subjects with Type 1 Diabetes: A Multicenter Real-World Study	2023	Diabetes Technol Ther	Chico A, Navas de Sol韩 S, Lainez M, Rius F, Cuesta M.	10.1089/dia.2022.0449	To evaluate the efficacy, safety and satisfaction of the closed-loop system Accu-Chek® Insight with Diabeloop™ (DBLG1™) in adults with type 1 diabetes (T1D) in real-world conditions.	Quantitative	Spain	Diabetes patients	A total of 62 adults with T1D were included in the present study N = 62 Age, years 44 ± 11.7 Gender, male/female, % 31/69	the Accu-Chek Insight with Diabeloop Closed-Loop System			In general, the satisfaction with the system at 3 months was considered high, with a mean score of over four for most questions (Table 1).		[illegible]		[illegible]					[illegible]
Wearable Devices for Assessing Function in Alzheimer's Disease: A European Public Involvement Activity About the Features and Preferences of Patients and Caregivers	2021	Frontiers in Aging Neuroscience	Stavropoulos T.G. and Lazarou I. and Diaz A. and Gove D. and Georges J. and Manyakov N.V. and Pich E.M. and Hinds C. and and Tsolaki M. and Nikolopoulos S. and Kompatsiaris I.	https://dx.doi.org/10.3389/fnagi.2021.643135 PT - Article	Alzheimer's Disease (AD) impairs the ability to carry out daily activities, reduces independence and quality of life and increases caregiver burden. Our understanding of functional decline has traditionally relied on reports by family and caregivers, which are subjective and vulnerable to recall bias. The Internet of Things (IoT) and wearable sensor technologies promise to provide objective, affordable, and reliable means for monitoring and understanding function. However, human factors for its acceptance are relatively unexplored. The Public Involvement (PI) activity presented in this paper aims to capture the preferences, priorities and concerns of people with AD and their caregivers for using monitoring wearables. Their feedback will drive device selection for clinical research, starting with the study of the RADAR-AD project.	Mixed	11 different countries, namely, from the Czech Republic, Bosnia and Herzegovina, the Republic of Ireland, England/Wales, Scotland, Portugal, Belgium, Sweden, Finland, Austria, and Germany.	Alzheimer patient and caregiver		Wearable Devices for Assessing Function in Alzheimer's Disease					[illegible]	[illegible]		[illegible]				[illegible]
Continuous glucose monitoring in older people with diabetes receiving home care-a feasibility study	2021	Pilot and Feasibility Studies	Larsen A.B. and Hermann M. and Graue M. AO - Graue, Marit; ORCID: https://orcid.org/0000-0002-3149-6227	https://dx.doi.org/10.1186/s40814-020-00754-3 PT - Article	Hypoglycemic incidents in older people can cause severe health problems, enhance general age-related disabilities, and cause frailty. Little is known about incidences of hypoglycemia in older home-dwelling people with diabetes. Thus, the aim of this study was to examine the feasibility of capturing hypoglycemia and issues associated with increased risk of hypoglycemia by use of continuous glucose monitoring (CGM) and standardized questionnaires among older home-dwelling individuals with diabetes type 2 receiving home care.	Quantitative	Norway	Older people with diabetes	We identified all older home-dwelling individuals ≥ 75 years diagnosed with diabetes type 2 and receiving home care (n = 20). In this small municipality, we identified 20 patients with diabetes type 2 of whom 12 were men and 8 women.	Continuous glucose monitoring					[illegible]			[illegible]				[illegible]

CAREGIVER WILLINGNESS TO PAY FOR DIGITAL TOOLS TO MONITOR MEDICATION ADHERENCE AND HEALTH OF PATIENTS WITH SERIOUS MENTAL ILLNESS	2021	Journal of Managed Care and Specialty Pharmacy	Forma F. and Chiu K. and Shafrin J. and Hadzi Boskovic D. and Veeranki P.		Adherence to antipsychotic medication is critical for effective psychopharmacologic therapy to prevent relapse and hospitalizations among patients diagnosed with serious mental illness (SMI) such as bipolar disorder, major depression and schizophrenia. Advances in information technology have resulted in emergence of digital tools that track medication adherence along with general health. Evidence on physician or patient preferences for such tools exist, but there is limited research on how family caregivers who support SMI patients would value these tools.To assess willingness-to-pay (WTP) for digital medication adherence and health monitoring tools among family caregivers of SMI patients.	Quantitative	USA	caregiver	A majority of caregivers were female (59%), Caucasian (69%), married (69%), had some college education (72%), and reported >$75,000 mean annual household income (47%). One-third of caregivers were parents of the patients with SMI, 30% were spouses/partners, 14% were children, and 22% were other types of family members. It should be noted that we did not purposively exclude non-family members from participation in the survey, and potential participants could also include friends, neighbors, or other unpaid caregivers. However, the analytic sample ultimately included participants who reported being related to the individual with SMI. Among the 184 caregivers, 57 (31%), 61 (33%) and 66 (36%) provided care to patients diagnosed with BPD, MDD and SCZ, respectively. Sixty four percent of the caregivers reported using medication adherence tools, and among them, 85% used the tools daily. In addition, although	health technology tools to monitor medication adherence among patients with serious mental illness			Overall, caregivers highly preferred digital adherence monitoring tools as compared to non-digital. Based on these preferences, a plurality of caregivers preferred a pill embedded with IEM sensor. Specifically, 39.5% of caregivers preferred a pill embedded with IEM sensor followed by a mobile app (26.6%) and a non-digital pill organizer (23.8%)					The disutility weight of the out-of-pocket cost was 0.0078 (95% confidence interval (CI): 0.011, 0.0038). Caregivers of patients with SMI highly preferred (OR 7.34, 95% CI: 5.00–10.79) a pill embedded with IEM sensor and were willing to pay $255.04 (95% CI $123.21–$386.86) per month more for this tool than a non-digital pill organizer. Similarly, caregivers had 2.64 times higher preference (95% CI: 1.90–3.70) with a WTP $124.50 (95% CI: $48.18–$200.81) per month more for a mobile app relative to a non-digital pill organizer. The smart pill organizer was found to be less preferred by caregivers (OR) 0.54 95% CI: 0.40–0.73) with a WTP of −$78.40 (95% CI: −$129.88, −$26.92) per month, which can be interpreted as needing to pay a caregiver $78.40 per month to use the smart pill dispenser				Declaration of conflicting interests: The author(s) declared the following potential conflicts of interest with respect to the research, authorship, and/or publication of this article: PRECISIONheor received funding from Otsuka Pharmaceutical Development & Commercialization Inc. to conduct this research. KC is an employee of PRECISIONheor, PV and JS are former employees of PRECISIONheor. DH is an employee of Otsuka Pharmaceutical Development & Commercialization, Inc., and FF is a former employee of Otsuka Pharmaceutical Development & Commercialization, Inc.
The future of vital sign monitoring: Testing and comparing ambulatory monitoring devices accuracy and wearability	2021	Physiotherapy (United Kingdom)	Areia C. and Vollam S. and Santos M. and Young L. and King E. and Tarassenko L. and Watkinson P.	https://dx.doi.org/10.1016/j.physio.2021.10.160 PT - Conference Abstract	Timely recognition of the deteriorating patient remains a challenge that has been recognised for decades. The growth of digital health technologies offers opportunities to improve patient safety and health outcomes. Ambulatory monitoring systems (AMS) may provide support to monitoring practices, however these systems need to be thoroughly tested before use in the clinical environment. The Virtual High Dependency Unit (vHDU) project is a set of mixed-methods studies with the objective of testing the feasibility of implementing ambulatory continuous vital-sign monitoring, collecting heart rate (HR), respiratory rate (RR) and oxygen saturations (SpO2) through AMS in hospital wards. Besides device performance, wearability is crucial to ensure successful implementation as devices need to be comfortable for patients to be compliant with monitoring. The objective was to investigate the wearability of a selection of commercially available AMS, as well as their sensitivity and specificity, in a hypoxia simulated environment and during movement; exploring the most promising AMS to	Quantitative	UK	patients	For the wearability study, 20 healthy volunteers wore each device for up to 72 h and completed a questionnaire per device.	vital sign monitoring					its performance was one of the worst during movement testing and its wearability score also significantly inferior in several sections of the comfort ratingscale(P<0.001).On the other hand the most wearable device was by farthe least accurate and reliable during hypoxia and movement.							UNDECLARED
Validity and usability of a wearable, multi-sensor system for monitoring upper and lower limb activity in chronic stroke survivors in a community setting	2021	Neurorehabilitation and Neural Repair	Rowe J. and Demers M. and Bishop L. and Zondervan D. and Winstein C.	https://dx.doi.org/10.1177/15459683211027047 PT - Conference Abstract	One of the most impenetrable problems after stroke is the gap between the motor capacity a stroke survivor regains and the amount of motor activity performed in the community.While there are useful tools for measuring upper (UL) and lower limb (LL) motor capacity in a clinical setting, few systems exist for accurately measuring UL and LL activity in an unsupervised community setting, especially for individuals with motor impairments. The goal of this research is to develop a wearable, multi-sensor system that accurately measures natural UL and LL activity, while leveraging end-user feedback to optimize usability.	Quantitative	Switzerland	Persons after Stroke	[illegible] were included in the study. The subjects' ages ranged from 44 to 90 years old, with 19 men and 9 women. The body height and BMI ranged from 149 to 187cm and 19.23 to 35.58 kg/m2, respectively. A total of eight participants had pronounced deficits in ambulation; eight were community ambulators; and 12 showed walking speeds comparable to the general age-matched population. In addition, nine participants used a walking stick for ambulation in daily life, and four participants used a foot orthosis. Further, all gait parameters for normal distribution were tested with the Shapiro-Wilk test and visually inspected in QQ-plots. The Shapiro-Wilk test did not confirm a normal distribution for one of the gait metrics. However, the inspection of the QQ-plots revealed a linear distribution of data points along the reference line for most of the	a Wearable Sensing System and Online Gait Analysis Report in Persons after Stroke					One of the participants reported some discomfort due to the attached sensors. More specifically, the patient had the illusion that the sensor he was wearing on the affected upper leg was too loosely attached and falling down.							N
A 2-year single-center experience with v-lap implantable remote monitoring system in advanced heart failure patients	2021	Giornale Italiano di Cardiologia	Restivo A. and D'Amario D. and Paglianiti D.A. and Aspromonte N. and Locorotondo G. and Burzotta F. and Trani C. and Crea F.		Heart failure (HF) is a complex syndrome affecting millions of people worldwide. Despite current therapies and disease management approaches, heart failure-related hospitalizations remain an unsolved issue. Worsening signs and symptoms are late manifestations of heart failure exacerbations leading to hospital admission. The increase of ventricular filling pressures anticipates clinical manifestations. Thus, remote intracardiac-pressure monitoring by using implantable devices may reprcoent a Key tool to personalize treatment and prevent hemodynamic destabilization. Methods. In our Institution, four patients with chronic HF were enrolled in the VECTOR-HF trial (NCT03775161), a prospective, international, multicenter, clinical trial designed to	Qualitative	Italy	heart failure patients	over 65; 4 male/1 female;	Left Atrial Pressure Remote Monitoring			Importantly, the overall adherence of our patients to daily LAP measurements amounted to 87.6 ± 11.8%, attesting to remarkable patient usability and acceptance.		Importantly, the overall adherence of our patients to daily LAP measurements amounted to 87.6 ± 11.8%, attesting to remarkable patient usability and acceptance.							N
Use of wireless respiratory sensor for vital signs monitoring in women with patient controlled analgesia after gynaecological surgery	2021	Anesthesia and Analgesia	Eng Z. and En Hong L. and Xue Lian X. and In C.J.J. and Ming C.S. and Wen T.C. and Rehena S. and Leong S.B.		Respiratory depression is a rare but severe adverse event of opioid patient controlled analgesia (PCA). Currently, manual intermittent monitoring by nurses remains the standard of care and is labour intensive. We aimed to evaluate the use of a patient-worn wireless vital-signs sensor (Aingeal) as part of an integrated opioid delivery system to continuously monitor respiratory rate, heart rate and temperature in the post-operative gynaecology population.	Mixed	USA	opioid patient-controlled analgesia after gynaecological surgery	Female patients of American Society of Anesthesiologists (ASA) I-III, aged 21-70 years old, undergoing gynaecological surgery and requiring postoperative analgesia via intravenous PCA were recruited. Age (years) 46.7±9.0 Race Chinese 24 (68.6) Malay 8 (22.9) Indian 0 (0.0) Others 3 (8.5)	wireless respiratory rate sensor			Patients expressed high satisfaction regarding the use of sensor device		Patients expressed high satisfaction regarding the use of sensor device, although a few patients reported discomfort or skin itchiness upon application [Table 2]. Some of them also had difficulties with self-application especially after showering.		Adequate information 4 (3-5) Comfort all the time 4 (2-5) No skin irritations 4 (2-5) Able to continue with daily activities 4 (2-5) Comfortable applying the device independently 4 (2-5) Comfortable having HR and RR monitored 4 (2-5) Comfortable having monitored remotely without nurses' presence 4 (2-5) Feel more secure with continuous monitoring than with periodic checks 4 (2-5) Keen to continue remote monitoring if warded again 4 (2-5)					N
Designing and Evaluating a Scale-Driven Wearable Diagnostic Assessment System for Children with Neurodevelopmental Disorders	2021	International Journal of Psychophysiology	Chen Y.	https://dx.doi.org/10.1016/j.ijpsycho.2021.07.174 PT - Conference Abstract	Attention Deficit Hyperactivity Disorder (ADHD) is a common neurodevelopmental disorder among school-age children. Children with ADHD show inappropriate inattention, impulsivity, or hyperactivity compared to their peers. We designed and developeda scale-driven wearable diagnostic assessment system named WeDA, a prototype system that integrates interactive screen, interactive objects, and wearable sensors. In a bid to answer our question about the diagnosis effect, we explicitly aimed to assess the current diagnostic accuracy of ADHD diagnoses and the coverage of the DSM-5.	Mixed	China	Children with ADHD	ADHD Age 7-13, M=9.22, SD=2.79 Gender B=42, G=8 ; TD 7-13, M=9.08, SD=3.46 B=35, G=15 ;ADHD-30 7-13, M=9.20, SD=2.92 B=30, G=0 ;TD-30 7-13, M=9.17, SD=2.90 B=30, G=0	A Scale-driven Wearable Diagnostic Assessment System			Doctors have good confidence in the diagnosis results of the WeDA system (Q7 Median=4). However, they only maintain a slightly better attitude towards the effectiveness of the WeDA system (Q3 Median=3.5), in comparison with the traditional approaches (Q5 Median=3.5). Moreover, they thought the time consumption was almost the same as usual (Q4 Median=3). We also got feedback from parents of their acceptance of the system and their assessment of children's use. From the results shown in Table 6, we can find parents hold more positive attitude (Q3 Median=4) toward wearable based diagnosis (Q2 Median=5), and they think the equipment interesting (Q1 Median=5) and does not cause anxiety in their children (Q4 Median=5).		of 20 children thought it was attractive and wanted to play again if possible. 4 of them had no particular feeling about it. Only one child did not like it as it was not as attractive as expected. For the time consumption, 15 of 20 said the length of the task did not make them feel uncomfortable, and the other five children said they persevered to the end to accomplish their task. We also care about children's acceptance of the wearable devices. Fortunately, from the feedback we got from both ADHD and Control groups, half of them did not have any strong feeling about the wearable equipment. And 35% said they felt good and had a good experience of finishing tasks with wearable devices. Moreover, our wearable device weighs only 25 grams, almost half the weight of an Apple watch. Eighty percent of them agree that wearing a watch does not affect their physical activity at all.		Finally, we provided an open question of "Q9: What do you think WeDA system needs to be improved?", and the most common feedback was "Can you design the wearable sensors smaller?". The comfort of the equipment is the next step we need to consider, not only the size of equipment but also the quantity of sensors to wear and their positioning.					UNDECLARED
Goals, life events, and transitions: Examining fertility apps for holistic health tracking	2021	JAMIA Open	Costa Figueiredo M. and Huynh T. and Takei A. and Epstein D.A. and Chen Y.	https://dx.doi.org/10.1093/jamiaopen/ooab013 PT - Article	Fertility is becoming increasingly supported by consumer health technologies, especially mobile apps that support self-tracking activities. However, it is not clear whether the apps support the variety of goals and life events of those who menstruate, especially during transitions between them. Thirty-one of the most popular fertility apps were evaluated, analyzing data from three sources: the content of app store pages, app features, and user reviews.	Qualitative	USA	women		fertility app			Users' reviews express desiring a similarly vast and varied list of indicators. Many users highlight how they enjoy "how extensive tracking is" [A1]. Some reviewers request the ability to track more, frequently asking for additional indicators. Requested indicators are often associated with periods or general symptoms (eg, "I wish I could track other things such as headaches, hunger, mood, cramps, and etc. I currently use another app to capture these other data" [A31]). In addition, people's health circumstances often lead to other suggested indicators. For example, a user with diabetes and TTC wished to also track insulin levels to avoid complications around her glucose level:49 "the app should include the option to track sugar levels for woman with diabetes and TTC. We should be allowed to track our insulin level within the app" [A1]. Another user who is pumping wished to track how her menstrual cycle influenced milk production: "I know that moms who are solely pumping are a minority, but [if] things like milk production in ounces […] appeared as a line in the fertility graph, I would be able see how ovulation and menstruation actually affect my milk output" [A1]		Given the value of storing long-term data, many users complain about data loss. Many user reviews complain about how apps support pregnancy, miscarriage, and breastfeeding, events that are directly related with conceiving. Not all pregnancies come to full term: 20% of all pregnancies are estimated to end in a miscarriage.50 Only 5 apps among the 31 offer some level of support after a miscarriage, for example by allowing registration of a miscarriage, providing articles and supportive messages, providing guidance for tracking after a miscarriage, and resetting or stopping predictions and emails. Some users comment that apps, even ones that offer support for TTC, do not offer an option to input a miscarriage (Q4), or even impact predictions in unexpected and potentially irreversible ways when a miscarriage is tracked (Q5). Additionally, apps' pregnancy messages, if not configurable, could serve as a reminder of miscarriages (Q6). Finally, even when pregnancy comes to term, other events can interfere with app use and fertility cycles. For instance, some users expressed that apps did not support their needs while breastfeeding post-partum (Q7).apps generally do not adequately support users apart from their reproductive years, during which apps often presume people have conception goals.Reviews suggest that apps tend to lack support particularly for the initial menstruation cycles during adolescence or the increased irregularity during menopause. For adolescence, similar to prior findings,8 some teenage users found it inappropriate when apps focused on conception (Q8). Only 10 apps ask user age during the onboarding process, even though this information can additionally help		Although many users comment how they like being able to see their data through calendar and temperature graphs, user reviews highlight that these visualizations do not fully support peoples' fertility goals. People's goals for understanding their symptoms and mood are not particularly well-supported by fertility apps. Users suggest wanting to compare their mood and symptoms by cycle. Despite apps' focus on conception, even some users who are TTC complain they do not support identifying and understanding patterns of symptoms throughout the cycle. All app visualizations analyzed are pre-defined and not customizable, so users cannot choose which indicators they want plotted, analyzed, or correlated within a cycle.Graphs are not customizable and pattern analysis features are uncommon, showing only one indicator at a time.			Reviews revealed that many people use fertility apps as data storage tools, so they can "have all my information in one spot so I can look back on" [A6]. Additionally, some users felt that storing long-term data could be useful to prepare for goal changes	However, apps can also normalize particular experiences and ignore others (Q16). Fertility apps also often make heteronormative assumptions about users' genders or sexuality.8 Participants who did not fit these assumptions noted that their data needs were not being well-supported (Q17–18).	N
Access to smart devices and utilization of online health resources among older cardiac rehabilitation participants	2020	World Journal of Cardiology	Saadi A. and Kanmanthareddy A. and Anantha-Narayanan M. and Hardy K. and Williams M. and Alla V.M. AO - Williams, Mark; ORCID: https://orcid.org/0000-0002-1133-4765 AO - Saadi, Abdulghani; ORCID: https://orcid.org/0000-0003-4983-0040 AO - Kanmanthareddy, Arun; ORCID: https://orcid.org/0000-0003-0256-1146 AO - Hardy, Karen; ORCID: https://orcid.org/0000-0002-5937-8492 AO - Alla, Venkata M.; ORCID: https://orcid.org/0000-0002-9196-7238 AO - Anantha-Narayanan, Mahesh; ORCID:	https://dx.doi.org/10.4330/wjc.v12.i5.203 PT - Article	Newer models of cardiac rehabilitation (CR) delivery are promising but depend upon patient participation and ability to use technological media including Internet and smart devices. AIM To explore the availability of smart devices, current utilization and proficiency of use among older CR program attendees.	Quantitative	USA	older cardiac rehabilitation participants	Patient characteristics, n (%) Patient characteristics n = 169 Age mean (SD) 71.1 (10.3)< 65 yr 37 (21.9)≥ 65 yr 132 (78.1) Gender Males 124 (73.5)Females 45 (26.5) Race Caucasian 152 (89.7)African American 9 (5.4)Hispanic 3 (1.8)Others 5 (3.1) House-hold income1 < 20 K 11 (6.33)20-39 K 28 (16.46)40-89 K 53 (31.65)≥ 90 K 43 (25.32)Not reported 34 (20.25) Education High school or less 37 (21.82)Some college 44 (26.06)College degree 49 (29.09)Postgraduate degree 29 (16.97)Doctoral degree 10 (6.06)									Of all participants, 72% reported no barriers to using Internet. Education and income were associated positively with measures of utilization and with less perceived barriers.As for barriers to utilization, 71% of participants reported perceiving no barriers to using Internet, 5.9% did not have Internet, 4.2% could not afford Internet, and 16.9% either found Internet difficult to operate or preferred not to use it. Mean use time was higher in participants who were < 65 years (2.5 h/d) compared to those who were ≥ 65 years of age (1.8 h/d) though this was not statistically significant (NS, P = 0.12). Participants who had a college education or above had a higher mean use-time of 2.3 h/d vs 1.6 h/d in the "No College" group (P = 0.02) (Figure (Figure1).1). Of all participants without a college education, 39% perceived barriers to Internet use versus only 20% of participants with a college degree who perceived a barrier to Internet use (P = 0.028). Finally, a significantly greater proportion of participants (39%) with an income of < $40 K, perceived barriers to Internet use compared to 17% of those with an income ≥ $40 K (P = 0.007).				UNDECLARED
Exploring public attitude toward biofeedback technologies: Knowledge, preferences and personality tendencies	2020	Journal of Public Health Research	Russo G.A. and Oliveri S. and Cincidda C. and Guiddi P. and Pravettoni G.	https://dx.doi.org/10.4081/jphr.2020.1782 PT - Article	Biofeedback is increasingly used in the clinical area and in daily health monitoring through wearable devices (e.g. smart watches). Nevertheless, it remains rather unknown. This study aimed to assess, in a sample of Italian citizens, the level of knowledge, attitudes, perceived efficacy and personality tendencies which could affect the uptake of biofeedback technologies.	Quantitative	Italy	General public	They were basically young adults (ages 18-30 years), adults and middle-aged adults (ages 30-65 years; only a couple of participants had an age over 65). 68.6% of the total subjects enrolled (160) were females (109). Their mean age was 35 (SD = 10) (minimum = 21 and maximum = 72) and among them, 41.3% was married or cohabiting. 46.2% had a bachelor's degree, whereas 19.2 % had a higher instruction that ranged from master's degree to Ph.D. and/or specialization.	Biofeedback is increasingly used in the clinical area and in daily health monitoring through wearable devices (e.g. smart watches).	The overall knowledge of psychophysiology among the participants was very low (M= 2.95, DS= 2.75): only 29.4% of subjects declared to know the biofeedback and 21.5% were aware of the biofeedback application in healthcare.		There was a good attitude toward the biofeedback training (question "How much would you be willing to undergo a biofeedback training?" ranged on a scale from 0 to 10, the mean score was M=6.01; DS=2.82), with no difference based on gender, psychological (sleep disorders, anxiety, depression) or physical status (e.g., participants mentioned osteoarthritis, thyroiditis, hypertension, hypothyroidism, celiac disease, migraine, diabetes) and no correlation with age and with physical activity.	Results showed that subjects who claimed to know the biofeedback had higher scores in Health Locus of Control subscale (t (158) = 2.210; p=0.029) compared to people who did not know. Furthermore, the Health Locus of Control had positive correlation with interest in undergoing biofeedback training (r = 0.240; p<0.001) and perceived efficacy of the same (r = 0.218; p<0.001). There was no significant difference in health personality tendencies between people who knew about the application of biofeedback in healthcare and people who did not know. The Health Esteem negatively correlated with interest in biofeedback training (r = -0.258; p<0.001) and with perceived efficacy of these technique (r = -0.246; p<0.001).		The foremost reasons to get biofeedback training, selected by the participants, were "I think it would be helpful to learn behaviors able to improve my health status (breathing rate, relaxing, etc.) without the application of the device" (46.9% participants), " I think it could be useful to know my health status (42.5% participants), and "Curiosity" (34.4% participants).		The most recurrent motivation that guided the reject of biofeedback training was: "The results might cause worry about my health status" (15%) and "I am worried about the application of the sensors".				UNDECLARED
Factors influencing motivation and engagement in mobile health among patients with sickle cell disease in low-prevalence, high-income countries: Qualitative exploration of patient requirements	2020	JMIR Human Factors	Issom D.-Z. and Henriksen A. and Woldaregay A.Z. and Rochat J. and Lovis C. and Hartvigsen G.	https://dx.doi.org/10.2196/14599 PT - Article	Sickle cell disease (SCD) is a hematological genetic disease affecting over 25 million people worldwide. The main clinical manifestations of SCD, hemolytic anemia and vaso-occlusion, lead to chronic pain and organ damages. With recent advances in childhood care, high-income countries have seen SCD drift from a disease of early childhood mortality to a neglected chronic disease of adulthood. In particular, coordinated, preventive, and comprehensive care for adults with SCD is largely underresourced. Consequently, patients are left to self-manage. Mobile health (mHealth) apps for chronic disease self-management are now flooding app stores. This study aimed to identify factors that may influence the long-term engagement and user adoption of mHealth among the particularly isolated community of adult patients with SCD living in low-prevalence, high-income countries.	Qualitative	Switzerland and Norway	Patients With Sickle Cell Disease in Low-Prevalence the caregiver of a person with SCD.	A total of 11 participants (7 males and 4 females) agreed to join the study. This led to a total of 7 patients and 3 caregivers participating in the study. Patients were aged between 21 and 55 years. A total of 7 participants were residing in Switzerland, 3 in Norway, and 1 was partly residing in the Democratic Republic of the Congo and in Switzerland.	Mobile health (mHealth) apps for chronic disease self-management						The three main motivators identified were (1) strengthen social support, (2) prevent VOC, and (3) reduce the limitations to functioning and independent living. Regarding the first motivator, participants wished to be able to enjoy with their families and give back to their communities.	[illegible] self-care tasks. Most emphasized the difficulty of managing their own limits. Some wished to be warned before exceeding their physical limitations.Importantly, most of participants suggested that such warnings should be detected with wearable sensors, releasing their cognitive load. Several participants stated that information overload would demotivate them for long-term engagement, specifying that mHealth apps should be as discreet as possible. Furthermore, most participants recognized the potential usefulness of notifications, but only if discreet and not disturbing.Notwithstanding, the majority of participants preferred user interfaces that are simple to use and do not require a high learning curve. Some participants believed messaging software was the best inspiration because of its intuitive interface. Another point to consider is that all participants expressed a lack of specificity and adaptability to SCD particularities in the usual health apps (ie, normal values for people with SCD are the values indicating a disease in healthy people). Finally, most participants preferred				Privacy issues were also a fundamental concern for most interviewees.	N
Biomedical REAl-Time Health Evaluation (BREATHE): Toward an mHealth informatics platform	2020	JAMIA Open	Bui A.A.T. and Hosseini A. and Rocchio R. and Jacobs N. and Ross M.K. and Okelo S. and Lurmann F. and Eckel S. and Dzubur E. and Dunton G. and Gilliland F. and Sarrafzadeh M. and Habre R.	https://dx.doi.org/10.1093/jamiaopen/ooaa011 PT - Article	To describe a configurable mobile health (mHealth) framework for integration of physiologic and environmental sensors to be used in studies focusing on the domain of pediatric asthma.	Quantitative	USA	Pediatric asthma patient		The Biomedical REAl-Time Health Evaluation (BREATHE) platform connects different sensors and data streams, contextualizing an individual's symptoms and daily activities over time to understand pediatric asthma's presentation and its management.			Of the 19 completed exit surveys, most children reported being satisfied (12, 63%) or very satisfied (6, 32%) with		their overall experience using BREATHE and found it somewhat easy (10, 53%) or very easy (9, 47%) to use. Parents/caretakers were similarly very satisfied (9, 47%) or satisfied (8, 42%) with their child's overall experience, with only 2 (11%) reporting being dissatisfied. Most parents agreed (13, 68%) or strongly agreed (5, 26%) that their child's experience using BREATHE was enjoyable and easy to remember using as instructed (12 [63%] agree, 4 [21%] strongly agree, and 3 [15%] disagree). When asked if using BREATHE every day was burdensome or took too much time or effort for their child, most parents disagreed (7, 37%) or strongly disagreed (4, 21%), although 7 (37%) agreed and 1 (5%) strongly agreed. Most parents (14, 74%) also did not think that carrying the kit every day was uncomfortable for their child. With respect to the smartphone app specifically, of 17 respondents, 14 (82%) and 3 (18%) thought the smartphone app was very easy or somewhat easy to use, respectively.		[illegible] of use or more pleasant experience were smaller or less bulky devices (especially the air monitor); more options to bundle the devices together; and integrating more sensors into the smartwatch (if not using only a smartwatch) with more durable protection (ie, more "sports" friendly). When asked what they would like to see to make BREATHE more useful to learn about their child's asthma and triggers, the majority of parents mentioned seeing more information, especially about air pollutant exposures and how they relate to exacerbations specifically at school or during certain activities; more tracking of symptom responses; and reminders to use medication as scheduled. When asked about the longest duration of time the parent and child would consider acceptable if given the option of using BREATHE for an extended period to learn more about the child's environmental exposures, experiences, and asthma, 2 weeks (7, or 41% of n = 17), 4 weeks (5, 29%), and 1 week (5, 29%) were the most frequent responses. One participant responded "Spring" because of the seasonal nature of the child's asthma, another with an open-ended "any" duration of time, and one with 8 weeks as the maximum duration. Two participants mentioned in-school months as more challenging given limited device access while at school.					N
Acceptability of the FreeStyle Libre Flash Glucose Monitoring System: The Experience of Young Patients With Type 1 Diabetes	2020	Clinical Medicine Insights: Endocrinology and Diabetes	Al Hayek A.A. and Robert A.A. and Al Dawish M.A. AO - Al Hayek, Ayman A; ORCID: https://orcid.org/0000-0002-3483-3196	https://dx.doi.org/10.1177/1179551420910122 PT - Article	In the current era of modern technology, the development of smart devices such as the flash glucose monitoring (FGM) systems helps patients with diabetes to effortlessly monitor their glucose levels more frequently. In this study, we determine the user acceptability of FGM among young patients with type 1 diabetes (T1D).	Quantitative	Saudi Arabia	Young Patients With Type 1 Diabetes	Variable Frequency % Gender Male 37 55.2Female 30 44.8 Age <15 y 35 52.2≥15 y 32 47.8	the FreeStyle Libre Flash Glucose Monitoring System					[illegible] participants strongly agreed that it was less painful to get glucose readings from the freestyle sensor, and that it was a more discreet (83.6%), more comfortable (85.1%), easier (95.5%), faster (82.1%), simpler (79.1%), more private (88.1%), and less stressful (77.6%) method, with minimal hassle (74.6%). It is noteworthy here that 86.6% of the participants reported absolutely no pain when the freestyle sensor was applied; also, majority of the participant (91%) reported no pain symptoms when scanning the sensor. From their statements regarding sensor application, 95.5% of the study population strongly agreed that the sensor application caused less pain than the routine finger-stick. Similarly, 85% of the users strongly agreed that using the sensor was comfortable, while 94% strongly agreed that they found the small size of the FGM made it easy to wear, 47% strongly agreed that wearing the sensor did not attract attention, 70% reported no discomfort under the skin, 80.6% stated that the sensor could be scanned without anyone noticing it, 89.6% felt that the sensor did not affect their daily activities, 91% strongly agreed that the sensor was very compatible with their lifestyle, 79.1% reported ease with taking a glucose reading with the scan, 89.6% reported that taking glucose readings with this system would not disrupt their daily activities, and 76.1% participants were excited to share with other individuals their experiences with this system.						Among the participants screened, the duration of the incomplete sensor placement reported was identified as premature accidental sensor removal, which occurred in 27 patients (42.2%) who mentioned sensor application and robustness problems. Further, a total of 6 participants complained of pain, which caused Figure 1 indicates the total user acceptability score of the study population.	N

Patient Perception and Satisfaction With Insulin Pump System: Pilot User Experience Survey	2019	Journal of Diabetes Science and Technology	Grando M.A. and Bayuk M. and Karway G. and Corrette K. and Groat D. and Cook C.B. and Thompson B. AO - Grando, Maria Adela; ORCID: https://orcid.org/0000-0002-5769-8556	https://dx.doi.org/10.1177/1932296819843146 PT - Article	The goal of this study was to assess patient perspectives and satisfaction with the MiniMed 670G insulin pump. Those participants who used the pump as part of a hybrid closed loop were also asked to provide their views on the automatic feature (auto mode).	Quantitative	USA	Diabetes patients	Demographic Count (%), or mean (standard deviation)<br>Age (years) 44.3 (18)<br>HbA1c 6.7% (0.7%)<br>Length of diagnosis (years) 26 (19)<br>Insulin pump usage (years) 13 (10)<br>Hybrid closed loop system usage (days) 154 (122)<br># female 14 (74%)<br># Caucasian 18 (95%)	Insulin Pump System			Overall, participants indicated a high level of satisfaction with the pump (14/21) mostly because of improvements in blood glucose (BG) control (15/21).		As most liked features, better control and more consistent BG level (9) was followed by ease of use (5). Few participants indicated that they liked nothing about the auto mode (2). Satisfaction with insulin delivery was also mentioned (1). Between the least liked features, pump allowing BG to remain elevated was top rated (5), followed by complains about high alert frequency (4), dissatisfaction with insulin administration (3), and settings not flexible enough (2). Few participants indicated that there was nothing about the auto mode that they disliked (2). One participant indicating not using the auto mode feature.					Overall, participants indicated a high level of satisfaction with the pump (14/21) mostly because of improvements in blood glucose (BG) control (15/21).		N
Toward a precision behavioral medicine approach to addressing high-risk sun exposure: A qualitative analysis	2019	JAMIA Open	Stump T.K. and Spring B. and Marchese S.H. and Alshurafa N. and Robinson J.K.	https://dx.doi.org/10.1093/jamiaopen/ooz034 PT - Article	Precision behavioral medicine techniques integrating wearable ultraviolet radiation (UVR) sensors may help individuals avoid sun exposure that places them at-risk for skin cancer. As a preliminary step in our patient-centered process of developing a just-in-time adaptive intervention, this study evaluated reactions and preferences to UVR sensors among melanoma survivors.	Qualitative	USA	Early stage adult melanoma survivors	All study participants identified as White/Non-Hispanic. Among the eleven participants in two focus groups, 55% were female, and ages ranged from 28 to 65 (M = 46.82, SD = 13.08). Of the 39 observational study participants, 61.5% were female, and ages ranged from 28 to 84 (M = 58.59).	Precision behavioral medicine techniques integrating wearable ultraviolet radiation (UVR) sensors			In general, participants were not interested in logging their sun protection on an online or paper log. Likewise, participants were also not particularly interested in feedback on their use of sun protection.<br>When presented with printed education on sun protection, participants expressed that this information was too basic to be useful.<br>Participants indicated that this information could capture their attention and be something that they check daily, similar to a weather forecast.<br>Participants were enthusiastic about the use of UVR sensors, saying it was "very cool" (F10) and suggesting that it would be most useful if it were connected to a mobile application.		Participants questioned the appropriate body location for a UVR sensor. This participant also added that by placing the sensor on more than one body site.<br>Participants indicated that they would be more interested in UVR-sensing devices that are "waterproof and sunscreen-proof" . Appearance was also a concern, with several participants expressing a desire for a small device in attractive colors.<br>Several participants indicated interest in adding a UVR sensors to products they were already wearing or using, such as "adher[ing] it to the side of your sunglasses."<br><br>Participants provided mixed input on how long they would want to use a UVR sensor.		an opportunity to more fully understand the kind of information provided by the device. Likewise, another participant had concerns about understanding the data, indicating she would likely not wear the device.<br>Participants valued the real-time feedback that a UVR monitoring system could provide and believed it should ideally have the ability to log and provide reminders of sunscreen application times, ideally on the actual device—"Yeah, just like a button" (F11)—and suggested that the device could chime, speak, or change colors to indicate reapplication time.					N
A theoretical framework and conceptual design for engaging children in therapy at home-the design of a wearable breathing trainer	2019	Journal of Personalized Medicine	Siering L. and Ludden G.D.S. and Mader A. and Van Rees H. AO - Ludden, Geke D.S.; ORCID: https://orcid.org/0000-0003-4508-9865	https://dx.doi.org/10.3390/jpm9020027 PT - Article	Wearable technologies are being implemented in the health and medical context with increasing frequency. Such technologies offer valuable opportunities to stimulate self-management in these domains. In this context, engagement plays a crucial role. An engaged patient is a patient who is emotionally involved and committed to the therapy or care process. Particularly for children who have to follow some sort of therapy, engagement is important to ensure a successful outcome of the therapy. To design for engagement, a framework based on theories of motivation in child therapy was developed.	Qualitative	Netherlands	Children in Therapy at Home	(aged 6–13 years, 6 girls)	a Wearable Breathing Trainer			In general, the concept was received positively.		Nevertheless, for all children, the interaction between vest and application was clear and they were positive and even in awe about it.		more and brighter colors. Some children indicated that the squares should be hidden to make it look like a normal shirt. Only one girl explicitly mentioned that she likes the appearance of the squares. However, they wished for more interaction: "I would like to have something like a small game that I can play after my training (12-year old boy). Younger children mentioned that they would prefer a different story, or different characters: "Many children like animals, maybe we can make it a zoo story" (10-year old girl), "I like firemen, so I would like to help a fireman" (7-year old girl).			The children liked the application as a supporting tool.		N
Can Google Glass improve freezing of gait in parkinsonism? A pilot study	2019	Movement Disorder	Lee A. and Hellmers N. and Vo M. and Popa P. and Henchcliffe C. and Sarva H. and Campbell C.		To determine feasibility of Google Glass (GG) technology to improve freezing of gait (FOG) in parkinsonism	Quantitative	USA	parkinson patients who feel freezing of gait	Of the nine subjects, mean age was 70.7 years (range 54–82, SD 10.0), and 5 subjects were female.	google glass					Most (5/9) subjects felt very comfortable or comfortable using GG, 7/9 responded that they would be interested in owning a pair of smart glasses with a dance cueing app in the future, and 7/9 responded that they would recommend the device to other people. Problems reported by the subjects (Figure 3) included: difficult to use tap controls (N = 4); confusing screen instructions (N = 5); small screen size (N = 4); or low volume (N = 3). Some found the device distracting to use in public (N = 3).					On the question "Was there an improvement in freezing of gait," 3 subjects reported improvement.		N
Physical factors that influence patients' privacy perception toward a psychiatric behavioral monitoring system: A qualitative study	2018	Neuropsychiatric Disease and Treatment	Zakaria N. and Ramli R.	https://dx.doi.org/10.2147/NDT.S115261 PT - Article	Psychiatric patients have privacy concerns when it comes to technology intervention in the hospital setting. In this paper, we present scenarios for psychiatric behavioral monitoring systems to be placed in psychiatric wards to understand patients' perception regarding privacy.	Qualitative	Malaysia	Psychiatric patients	patients' age ranged from 14 to 54 years. Out of the 25 participants, 20 reported having an occupation varying from running a business to a government position such as police officer.	psychiatric behavioral monitoring system			Because of the nature of their illnesses, four patients (Olsen, Kathy, Felicia, and Murdoch) said they would feel very happy if there was a camera monitoring their behavior because they enjoy the attention.				Of these 16 patients, 2 (Michael and Abe) mentioned that it would be better if cameras were mounted only in certain places, even though the interviewers had not yet mentioned this. Michael and Abe suggested that cameras should be located near the beds and the meeting room, but not in the bathroom.				geneal no concerns for privacy due to illness<br><br>Five patients (Jamie, Jesse, Candice, Sarah, and Nancy) stated that they would feel unhappy and stressed if there was a camera monitoring their behavior in the ward.<br><br>Each participant had his or her own privacy preferences regarding whom they would allow to perform the monitoring.	N
The HOPE pilot study: Harnessing patient-reported outcomes and biometric data to enhance cancer care	2018	JCO Clinical Cancer Informatics	Wright A.A. and Raman N. and Staples P. and Schonholz S. and Cronin A. and Carlson K. and Keating N.L. and Onnela J.-P.	https://dx.doi.org/10.1200/CCI.17.00149 PT - Review	Purpose Integrating patient-reported outcomes (PROs) into clinical practice is an increasingly promising strategy for improving patients' symptoms, communication, and clinical outcomes. The objective of the current study was to assess the feasibility, acceptability, and perceived effectiveness of a mobile health intervention that was designed to collect PROs and activity data as a measure of health status.	Mixed	USA	patient with recurrent, incurable gynecologic cancer	Age mean 60; 8 married; 8 with children; 8 with more than 51000 income,; 6 white, 6 college education, 7 christian,	physical activity and the Beiwe research platform to collect PROs, stratify patient responses by risk, provide tailored symptom management, and notify patients and clinicians of high-risk symptoms.			Upon study completion, nine participants disagreed or strongly disagreed with the statement, "Participating in this study placed a substantial burden on me." Only one participant agreed with the statement, "I wish I had not agreed to participate in this study," citing her regret about her nonadherence to the study.							Both participants and clinicians indicated that participating in the intervention increased participants' physical activity, communication with the care team, and symptom management		N
User experience and clinical effectiveness with two wearable global positioning system devices in home dementia care	2018	Alzheimer's and Dementia: Translational Research and Clinical Interventions	Megges H. and Freiesleben S.D. and Rosch C. and Knoll N. and Wessel L. and Peters O.	https://dx.doi.org/10.1016/j.trci.2018.10.002 PT - Article	The user experience and clinical effectiveness with wearable global positioning system (GPS) devices for persons with dementia (PwDs) and caregivers (CGs) remain unclear although many are available. Using a crossover design, 20 dyads tested two similar commercial GPS watches (products A and B) at home for 4 weeks each. Usability, product functions, design features and product satisfaction at home and the clinic were investigated. Caregiver burden and quality of life assessed clinical effectiveness.	Quantitative	Germany	people with dementia	Characteristics PwDs (n = 17) CGs (n = 17)<br>Age, mean ± SD [range] 71.7 ± 6.9 [56–80] 67.7 ± 8.0 [51–77]<br>Gender ratio (% female) 8/9 (53) 9/8 (47)<br>Education<br>High school, n (%) 7 (41) 7 (41)College, n (%) 2 (12) 1 (6) University, n (%) 8 (47) 9 (53)	wearable global positioning system devices in home dementia care				Product satisfaction with both products was significantly lower at home.	The final 17 dyads rated the usability, telephone function, overall design features, font, buttons, and battery life of B significantly better than A. PwDs rated the overall design features and buttons of A significantly better than CGs.							UNDECLARED
"MigraineMonitor" - Towards a system for the prediction of migraine attacks using electrostimulation	2018	Biomedizinische Technik	Stefke A. and Wilm F. and Richer R. and Gradl S. and Eskofier B.M. and Forster C. and Namer B.		Within 24 h before a migraine attack unspecific premonitory symtpoms, like distortions of perception, and changes in sensitivity to external stimuli, such as heat pain, can occur. Recently, missing habituation of pain magnitude in migraine patients to a longer lasting heat stimulus and a 5 s suprathreshold electrical stimulation was described. Electrical stimulation in contrast to computer-driven heat stimulation has the advantage of robustness and home-usability of the system. Based on these results, this work presents a smartphone application to track influence factors, e.g. weather conditions and premonitory symptoms on migraine attacks and an Arduino-based control unit which replaces the traditional approach of manual and therefore error-prone and time-consuming electrical stimulation for an early prediction of migraine. The control unit consists of a microcontroller combined with a touchscreen and is designed to automatically control a stationary and clinical electrostimulator. The application implements a diary functionality for documenting migraine influence factors and tracking headache attacks. The usability of both components of the project was	Quantitative	Germany	migraine patients	The touchscreen-based control unit was evaluated with 15healthy subjects (44% female, age 32±13 years (M±SD)) The application's usability was evaluated independently fromthe control unit within a separate study of 21 subjects (71%female, age 31±13 (M±SD)), who tested the migraine diaryfunction over a period of two weeks	a System for thePrediction of Migraine Attacks usingElectrostimulation					Overall, the application reached a SUS score of 91.67±6.96(M±SD).							N
Ethical Implications of User Perceptions of Wearable Devices	2018	Science and engineering ethics	Segura Anaya L.H. and Alsadoon A. and Costadopoulos N. and Prasad P.W.C.	https://dx.doi.org/10.1007/s11948-017-9872-8 PT - Review	Health Wearable Devices enhance the quality of life, promote positive lifestyle changes and save time and money in medical appointments. However, Wearable Devices store large amounts of personal information that is accessed by third parties without user consent. This creates ethical issues regarding privacy, security and informed consent. This paper aims to demonstrate users' ethical perceptions of the use of Wearable Devices in the health sector.	Mixed	Australia	General public	60 respondents, 35 male and 25 female.	Health Wearable Devices				the relationship between Wearable Device Governance and the responses from males and females. Seventeen females out of twenty and twelve males out of twenty think that Wearable Devices should provide short policies easy to read, a more understandable informed consent form and involved regulatory authority from providers.<br><br>Table 6 shows the significance of informed consent for different age groups. 66% of individuals aged between 20 and 30 did not have concerns. In contrast, for respondents from the age group 60 to 70, informed consent was of high importance (60%). This was also the case for respondents aged between 50 and 60 (~50%) and those aged 40–50 (61%). Only three individuals between the age of 30 and 40 considered informed consent to be of high importance.							Most female participants answered that privacy and being aware of what the device is collecting as their main concern, while most males answered that privacy is an issue on Wearable Device risk perception.individuals with health issues might use such devices to monitor or control health related problems and that there is significant concern that those data will be shared with or misappropriated by third parties which and may be difficult to control where the internet is involved.	UNDECLARED
Multiple wearable sensors in parkinson and huntington disease individuals: A pilot study in clinic and at home	2017	Digital Biomarkers	Adams J.L. and Dinesh K. and Xiong M. and Tarolli C.G. and Sharma S. and Sheth N. and Aranyosi A.J. and Zhu W. and Goldenthal S. and Biglan K.M. and Dorsey E.R. and Sharma G.	https://dx.doi.org/10.1159/000479018 PT - Article	Clinician rating scales and patient-reported outcomes are the principal means of assessing motor symptoms in Parkinson disease and Huntington disease. However, these assessments are subjective and generally limited to episodic in-person visits. Wearable sensors can objectively and continuously measure motor features and could be valuable in clinical research and care.The study's aims were to assess the feasibility of use of wearable sensors, to determine the activity (lying, sitting, standing, walking) of participants, and to survey participants on their experience.	Quantitative	USA	Parkinson and Huntington Disease Individuals	Parkinson disease (n = 16) Huntington disease (n = 15) Prodromal Huntington disease (n = 5)<br>Demographics<br>Age, years 58±16.2 68±8.7 55±10.7 38±8.6<br>Sex (women), % 70 44 20 40<br>Ethnicity (white), % 90 94 100 100<br>Hispanic ethnicity, % 0 0 0 0<br>Education (4-year college degree or higher), % 50 100 53 40<br>Currently employed or student, % 45 19 13 60<br>Currently married or in a domestic partnership, % 85 100 53 0	Wearable Sensors			When asked if they would be willing to use these sensors if asked in the future, 86% (n = 48) of participants said they would be "willing" or "very willing."		the majority of participants found the sensors to be "comfortable" (n = 42) and "easy to remove" (n = 50). The most common complaint (n = 31) was regarding the adhesion of the sensors and participants reported sensors falling off or coming loose as a major drawback.							Ray Dorsey is a consultant to MC10 Inc., a wearable device company, that provided the sensors used in this study.
A New, Wireless-enabled Blood Glucose Monitoring System That Links to a Smart Mobile Device: Accuracy and User Performance Evaluation	2017	Journal of Diabetes Science and Technology	Christiansen M. and Greene C. and Pardo S. and Warchal-Windham M.E. and Harrison B. and Morin R. and Bailey T.S.	https://dx.doi.org/10.1177/1932296817691301 PT - Article	These studies investigated the accuracy of the new ContourNext ONE blood glucose monitoring system (BGMS) that is designed to sync with the ContourTM Diabetes app on a smartphone or tablet. A questionnaire was provided to assess user feedback on ease of use.	Quantitative	USA	Diabetes patients	Gender, n (%)<br>Female 171 (51)<br>Male 162 (49)<br>Type of diabetes, n (%)a<br>Type 1 116 (35)<br>Type 2 215 (65)<br>Type unknown 2 (1)<br>Do not have diabetes —<br>Age, years<br>Mean (range) 54 (18-81)<br>Race, n (%)a<br>White 258 (78)<br>Black/African American 46 (14)<br>Asian 20 (6)<br>American Indian/Alaska Native 6 (2)<br>Hawaiian/Pacific Islander 2 (1)<br>No answer 8 (2)	Blood Glucose Monitoring System			, and 92.1% of subjects responded "strongly agree," "agree," or "neutral" that they preferred the meter used in this study to their regular meters.		Ease-of-use questionnaire responses among subjects with diabetes (n = 332) demonstrated that most subjects "strongly agree," "agree," or are "neutral" that (1) it is easy to do a fingerstick blood test with this meter (97.9%), (2) the meter display is easy to see and read (98.2%), (3) it is easy to understand the test results (99.7%), (4) the instructions are easy to understand (97.6%), (5) the instructions clearly explain how to run a test (98.5%), and (6) the instructions clearly explain what to do if an error message is displayed by the meter (99.4%).				In addition, 85.6% of subjects responded "strongly agree," "agree," or "neutral" that they use their current meter because their insurance companies cover the strips	Diabetes management questionnaire responses among subjects with diabetes (n = 332) demonstrated that the majority of subjects "strongly agree," "agree," or are "neutral" that accuracy is important: (1) to help with their ability to manage their diabetes (99.4%), (2) to help with understanding how food or exercise affects low blood sugars (99.7%), (3) to help with preventing low blood sugars (99.7%), and (4) to help with using their results to gain better control of their diabetes (99.4%).	Safety There were 8 mild, anticipated, non–device-related adverse events; all were classified as hypoglycemia and all occurred in subjects with diabetes. All adverse events resolved prior to the subjects leaving the testing site.	The author(s) declared the following potential conflicts of interest with respect to the research, authorship, and/or publication of this article: MC has nothing to disclose. CG, SP, MEWW, and BH are employees of Ascensia Diabetes Care. RM was an employee of Ascensia Diabetes Care at the time of the study. TSB has received research support from Abbott, ACON, Ascensia Diabetes Care, Bristol-Myers Squibb, Dexcom, GlaxoSmithKline, Halozyme, Insulet, Janssen, Lexicon, LifeScan, Lilly, Medtronic, Merck, Novo Nordisk, Orexigen, and Sanofi; has received consulting honoraria from Ascensia Diabetes Care, AstraZeneca, BD, Lilly, Medtronic, Novo Nordisk, and Sanofi; and has received speaking honoraria from Abbott, Insulet, Novo Nordisk, and Sanofi.
Wirelessly observed therapy to optimize adherence and target interventions for oral hepatitis C treatment	2017	Journal of Managed Care and Specialty Pharmacy	Bonacini M. and Kim Y. and Pitney C. and McKoin L. and Landis C.		Real-world data on adherence with the new direct-acting agents (DAAs) to treat chronic hepatitis C virus (HCV) are limited, and suboptimal adherence can lead to unnecessary treatment failures. Refill methods to measure adherence are inaccurate and do not allow patients or providers the opportunity to respond quickly. A novel digital medicine offering (DMO, Proteus Discover) consists of digital medicines (medicines co-encapsulated with an ingestible sensor pill which activates when swallowed), wearable sensor patch (measures ingestion adherence, physical activity and heart rate), patient mobile app and provider web portal to allow for timely assessment and optimization of adherence. To evaluate real-world effectiveness of the DMO in patients with chronic HCV infection prescribed sofosbuvir and ledipasvir (SOF/LDV).	Quantitative	USA	patients diagnosed with chronic HCV infection	A total of 31 patients were screened, and of these patients, 28 met the inclusion criteria and were enrolled in the study (ITT population). All 28 patients (mean 59 years, SD 7; 61% [17/28] male; 61% [17/28] non-Caucasians; 93% [26/28] treatment naïve) had HCV GT-1.	a novel digital medicine program that directly measures drug ingestion adherence.			Overall satisfaction with the use of the device was high among study subjects (Figure 1).		Most (92%) patients completely or somewhat agreed that the DMP was easy to use in their daily routine and helped them feel more involved in managing their health care, 96% did not mind wearing the patch and sharing their data with their health care team					, and 85% agreed that the DMP helped them understand the importance of taking their medications regularly.		YK and MT are employees of Proteus Digital Health. MB has received research support from AbbVie, Allergan, Assembly Biosciences, Boehringer-Ingelheim, Intercept, Genfit, Gilead, Novartis, and Pfizer and has received speaker fees from AbbVie, Gilead, and Intercept. CL received research funding from Gilead, AbbVie, and Proteus. CP and LM report no disclosures.
Wearable devices and smartphones for activity tracking among people with serious mental illness	2016	Mental Health and Physical Activity	Naslund J.A. and Aschbrenner K.A. and Bartels S.J.	https://dx.doi.org/10.1016/j.mhpa.2016.02.001 PT - Article	People with serious mental illness, including schizophrenia spectrum and mood disorders, are more physically inactive than people from the general population. Emerging wearable devices and smartphone applications afford opportunities for promoting physical activity in this group. This exploratory mixed methods study obtained feedback from participants with serious mental illness to assess the acceptability of using wearable devices and smartphones to support a lifestyle intervention targeting weight loss.	Mixed	USA	People with Serious Mental Illness	N 11<br>Demographic Characteristics<br>Mean Age (SD) 48.2 (11.2)<br>Female (%) 8 (73%)<br>Non-Hispanic white (%) 11 (100%)<br>Education<br>Completed high school 5 (46%)Some college 3 (27%)College degree 3 (27%)<br>Living situation<br>Living independently 8 (73%)Living with family 3 (27%)<br>Marital status<br>Never married 3 (27%)Currently married 1 (9%)Previously married 7 (64%)<br>Currently Employed (part or full-time) 3 (27%)	activity tracker Fitbit			highly satisfied with the device.		Overall, participants were in agreement that the Fitbit was easy to use. Another participant described his experience using the Fitbit. Participants were also satisfied with the size of the device, and found it easy to understand<br><br>Not all features of the Fitbit mobile application were considered useful. For example, participants' considered the tracking of calories burned to be distracting and misleading. For some participants, the calories burned was the only real problem with the Fitbit in general	Three participants used the dietary tracking feature, 2 used the mobile application to track their weight, and 5 used the application to log how much water they consumed. Also, participants were enthusiastic about being able to see their own data on the mobile application.	Other participants mentioned practical concerns with operating the smartphone such as small screen size, short battery life, and the need for basic instructions for making calls or shutting off the phone.<br>For the few participants with limited prior exposure to mobile technologies, learning to use the smartphone became a source of distraction: "We spent a lot of time on just trying to figure out how to use the phone, which is unfortunate." (Participant #10). While another participant described her experiences learning to use the smartphone:<br><br>Some participants expressed the need for more instruction for using the smartphone and for accessing the companion mobile application for the Fitbit. Other participants recommended group training and tutorials for introducing the technology instead of individualized training.	There were only a few challenges with using the Fitbit, and these were mostly related to learning to use the smartphone and companion mobile application for the Fitbit.For one participant with limited prior experience using mobile technologies, the phone was a source of discouragement, and it made her feel like she could not understand it		motivating. Most participants (N=8) used the Fitbit for setting daily step goals, while others explained that the Fitbit helped create a sense of accomplishment from being more active and collecting more steps.<br>The Fitbit was also helpful for increasing awareness of physical activity and for setting daily step goals..<br>The Fitbit was even described as empowering by one participant, because it compelled her to work towards her daily step goal. Another participant found that the encouraging messages and prompts through the companion mobile application for the Fitbit helped her to reach her step goals. Most participants agreed that the Fitbit helped them to be more active, and that it was fun to use and that they would recommend it to a friend. Importantly, participants also indicated that the Fitbit was something that they would like to continue to use in the future.		N
Acceptability of an intelligent wireless sensor system for the rapid detection of health issues: Findings among home-dwelling older adults and their informal caregivers	2016	Patient Preference and Adherence	Cohen C. and Kampel T. and Verloo H.	https://dx.doi.org/10.2147/PPA.S113805 PT - Article	Aging at home rather than in an institution is now considered the gold standard. Public health figures document an important demographic transition to an increasingly elderly society. At the same time, this is accompanied by the emergence of significant numbers of innovative technologies to help and support home-dwelling older adults in declining health who wish to remain at home. To explore the acceptability of intelligent wireless sensor system (IWSS) among home-dwelling older adults in rapidly detecting their health issues.	Quantitative	Swtherland	home-dwelling older adults and their informal caregivers	Table 1 summarizes the sociodemographic characteristics of the participants receiving the IWSS intervention. Majority of the participants were female (67.6%), widowed, aged over 83 years, and lived alone (91.2%) in own home with nursing care.	an intelligent wireless sensor system for the rapid detection of health issues			Only 6/23 participants (26.1%) were satisfied with the IWSS, while more than half of their informal caregivers were satisfied (8/15; 53.3%).	Participants living long distances from their informal caregivers showed more interest in using IWSS.	every activity at homes. Two informal caregivers complained about the long time necessary to install the wireless sensors. Informal caregivers were dissatisfied with the need to acknowledge each alarm message with a telephone call to assess the pertinence of the alarm and whether any physical intervention was necessary. In a few families, these situations even went so far as to create conflicts between participants and their relations. More than half of the emergency alarm messages were sent out during the night, and participants in these situations often took an extended time to return to bed. Food burning on hotplates was another incident detected at two participants' homes and the IWSS sent emergency alarm messages to their nurses and informal caregivers. The IWSS failed to detect two falls, probably due to lack of suitably positioned sensors. Insufficient training for nurses on the use of IWSS caused several difficulties in the effective employment of its software in the study. almost one-sixth of the messages (13.8%) had technical errors.	During the clinical follow-up, patients stated that short distances from informal caregivers, their declining health status, and fear of falls or being found dead by family members made the IWSS worth using and encouraged them to do so. Participants and informal caregivers who stated that they had received clear, understandable information at the IWSS also felt that this facilitated the system's use.	Participants' ease of use was negatively influenced by mild or moderate cognitive impairment, resulting in a need for supplementary explanations about the IWSS. For several reasons, informal caregivers were mostly absent during the installation of the system and while giving explanations about the IWSS, resulting in inappropriate responses to emergency alarm messages. Finally, obstacles to the system's ease of use were also due to some of its components such as the failing mobile telephone connections in rural areas, which required the health care nurses to make adjustments and reduced the system's usefulness.	some rural areas were not always covered by the mobile phone network and some alarm messages did not reach the informal caregivers.		Regarding the usefulness of IWSS, one-third (34.8%) of the participants and three-quarters (76.5%) of the informal caregivers considered the IWSS useful for older adults who wished to remain in their homes, and believed that the IWSS was an appropriate means of ensuring safety in case of falls.	Participants also felt that the IWSS was intrusive and that they were being watched.	N
A technology-enabled adherence enhancement system for people with bipolar disorder: Results from a feasibility and patient acceptance analysis	2015	Patient Preference and Adherence	Sajatovic M. and Davis M.S. and Cassidy K.A. and Nestor J. and Sams J. and Fuentes-Casiano E.	https://dx.doi.org/10.2147/PPA.S81724 PT - Article	As poor medication adherence is common in bipolar disorder (BD), technology-assisted approaches may help to monitor and enhance adherence. This study evaluated preliminary feasibility, patient satisfaction and effects on adherence, BD knowledge, and BD symptoms associated with the use of a multicomponent technology-assisted adherence enhancement system.	Quantitative	USA	people with bipolar disorder	participants, mean age 62 years (range 49–71 years), 4/5 (80%) Caucasian, and 4/5 (80%) single/divorced or widowed. Mean years of education was 13 years (range 12–14 years). The majority resided in private homes with other people. Most participants (4/5, 80%) were on a single medication for treatment of BD. On average, participants had BD for 21 years (range 5–43 years) and were clinically stable, treated BD patients. These pilot study participants were mostly euthymic or mildly depressed. Possible ratings on the ISS for depressive severity range from 0 to 200 with higher scores representing more severe depression. The study sample ISS scores were reflective of their overall clinical stability with a mean of 40 (SD 56).	A technology-enabled adherence enhancement system					Usability scores on the 5-point scale were high overall with means ranging from 4.8 to 5.0 on all usability questions. When queried regarding what they liked best about the system, the most common response was the simplicity and ease of use, while the most common complaints or least liked components of the system were related to the difficulty with the temporary (tape) method used to affix the pill caps to the bottle.					Mean scores on the Morisky scale improved from baseline to follow-up (3.20–3.60). Means on all four subscales of the ISS were all in the direction of improvement. Scores on perceived conflict (baseline 60, endpoint 48), activation (baseline 64, endpoint 12), and depression index (baseline 40, endpoint 10) all decreased, while scores on well-being (baseline 222, endpoint 258) increased. Finally, mean TKT scores increased from baseline to follow-up (14–19.6) with all five participants demonstrating improved treatment knowledge relative to the baseline test administration.		Martha Sajatovic is a consultant for Prophase, Otsuka, Sunovion, Pfizer, Amgen, and Bracket, and has received grant support from Pfizer, Merck, Ortho-McNeil Janssen, and Janssen. Michael Davis is employed byMedicaSafe Inc. Joseph Nestor is a former employee of MedicaSafe Inc. The other authors report no conflicts of interest in this work.
Remote monitoring of cardiac implantable electronic devices-A single center experience	2015	Indian Heart Journal	Anitha and Aditya A. and Varatharaju J. and Ramkumar L. and Ajit U.		Follow up of patients with implantable cardiac electronic devices (ICED) - pacemaker, CRT and AICD is mandatory. The device interrogation is usually performed in the clinic periodically. The cost and the inconvenience of such an exercise have been shown to be significant. The newer technology of remote monitoring enables the patients to transmit the data from their devices constantly from a remote location. This study evaluates acceptability, feasibility, safety, cost effectiveness, benefits and technical difficulties with remote monitoring technology in India	Quantitative	India	patient with cardio diseases	Remote Monitoring of Cardiac Implantable Electronic Devices					The affordability of the patient and availability of cellular network influence the acceptability of the technology.								Dr. Zeitler was funded by National Institutes of Health (NIH) T-32 training grant #2 T32 HL 69749-11 A1, however, no relationships exist related to the analysis presented. Dr. Piccini receives grants for clinical research from ARCA biopharma, Boston Scientific, Gilead, Johnson & Johnson, ResMed, Spectranetics, and St Jude Medical, and serves as a consultant to BMS/Pfizer, GlaxoSmithKline, Janssen Pharmaceuticals, Medtronic, and Spectranetics.
Development of a smartphone application to enable remote monitoring in the outpatient management of cirrhotic ascites	2015	Journal of Hepatology	Ha P. and Warner S. and O'Neil P. and Anderson P. and Dev A. and Ratnam D. and Le S. and Sievert W.		Patients who develop hepatic decompensation with ascites have a poor prognosis and often experience other complications including spontaneous bacterial peritonitis, hepatic encephalopathy and variceal bleeding. We hypothesised that smartphone (SP)-enabled remote monitoring of patients with ascites may enable early detection of infection and acute decompensation, facilitate timely intervention and improve patient outcomes. This pilot study aimed to design, develop and implement a remote monitoring system (RMS) for outpatients with cirrhotic ascites.	Quantitative	Australia	Patients who develop hepatic decompensation with ascites	EthnicityCaucasian 21 (78.0%) Asian 5 (19.0%) African 1 (4.0%)<br>EmploymentEmployed 5 (18.5%)<br>Highest level of education achievedPrimary School 7 (25.9%) Secondary School 15 (55.6%) Tertiary 5 (18.5%)	A SMARTPHONE APPLICATION TO ENABLE REMOTE MONITORING IN THE OUTPATIENT MANAGEMENT OF CIRRHOTIC ASCITES			There were 19 (70%) patients who reported that they wanted a RMS for outpatient care.				There were 24 (89%) patients who requested information regarding diet and/or liver disease to be included in the service. All patients desired an outpatient appointment reminder service, however only one (5%) patient requested a medication reminder service.						N

Improving Self-Monitoring of Blood Glucose among Adults with Type 1 Diabetes: Results of the MobileTM Study	2014	Diabetes Therapy	Overland J. and Abousleiman J. and Chronopoulos A. and Leader N. and Molyneaux L. and Gilfillan C.	https://dx.doi.org/10.1007/s13300-014-0092-9 PT - Article	The objective of this study was to determine whether people with type 1 diabetes are more likely to self-monitor their blood glucose (SMBG) as recommended by their diabetes health care professional using the Accu-Chek MobileTM (F. Hoffmann-La Roche AG, Basel, Switzerland) monitoring system compared to the Freestyle OptiumTM (Abbott, North Chicago, IL, USA).,	Quantitative	Australia	Diabetes patients	Age (years)a 22.8 (1.8) 32.7 (10.3) Males (%) 60 43	Self-Monitoring of Blood Glucose among Adults with Type 1 Diabetes			After 3 months of using both monitoring systems, the majority of participants (77%) indicated a preference for the Accu-Chek Mobile meter.									Prof. Christopher Gilfillan was previously a member of the Roche Accu-Chek Advisory Board and received honorary for participation and support for transport to and from meetings. This period overlapped with the beginning of the study. Associate Professor Jane Overland remains a member of the Roche Accu-Chek Advisory Board and received honorary for participation and support for transport to and from meetings. Jessie Abousleiman, Andriana Chronopoulos, Natasha Leader, and Lynda Molyneaux declare no competing interests.
Usability testing of a monitoring and feedback tool to stimulate physical activity	2014	Patient Preference and Adherence	van der Weegen S. and Verwey R. and Tange H.J. and Spreeuwenberg M.D. and de Witte L.P.	https://dx.doi.org/10.2147/PPA.S57961 PT - Article	A monitoring and feedback tool to stimulate physical activity, consisting of an activity sensor, smartphone application (app), and website for patients and their practice nurses, has been developed: the 'It's LiFe!' tool. In this study the usability of the tool was evaluated by technology experts and end users (people with chronic obstructive pulmonary disease or type 2 diabetes, with ages from 40-70 years), to improve the user interfaces and content of the tool.	Mixed	Netherlands	people with chronic obstructive pulmonary disease or type 2 diabetes	Age mean (SD) 61.4 (8.5) 60.2 (9.0) 58.6 (7.8)<br>Sex number (%)<br>Male 4 (80%) 11 (55%) 3 (60%)<br>Female 1 (20%) 9 (45%) 2 (40%)<br>Disease number (%)<br>COPD 2 (40%) 10 (50%) 1 (20%)<br>Diabetes type 2 3 (60%) 10 (50%) 4 (80%)<br>Education level number (%)<br>≤ intermediate vocational education 3 (60%) – 4 (80%)<br>> intermediate vocational education 2 (40%) – 1 (20%)<br>Computer experience number (%)<br>≤5 hours per week 1 (20%) 2 (10%) 1 (20%)<br>>5 hours per week 4 (80%) 18 (90%) 4 (80%)<br>Smartphone experience number (%)<br>Yes 1 (20%) 6 (30%) 1 (20%)<br>No 4 (80%) 14 (70%) 4 (80%)	A monitoring and feedback tool to stimulate physical activity, consisting of an activity sensor, smartphone application (app), and website for patients and their practice nurses			The participants rated the desirability of the app and website positively.	Again, people with higher education levels tended to be more critical.	To describe the app, three participants chose the phrase "easy to use" and two participants chose the words "motivating", "usable", "understandable", "useful", "suitable", and "accessible". The only chosen word that could be interpreted as negative was "business-like". Concerning the website, "accessible" was chosen three times and "clear", "interesting", "understandable", and "stimulating" were chosen by two participants.<br><br>The participants rated "comprehensiveness" and "readability" of the manual with an average of 4.5 and 4.8, respectively, on a scale ranging from 1 to 7 (Table 1). Based on the suggestions for improvement, the manual was extended with more information about the general use of the smartphone. One participant doubted whether the activity sensor was robust enough.<br><br>Although most participants had no previous experience with smartphones, none of the eight tasks on the app were rated as difficult (Table 1). most participants were afraid of losing the sensor. This problem was solved in the new prototype by adding a security clip with a thread that can be attached to belt loops. almost all participants had the idea that the activity results were not consistent with their experienced activity.<br><br>Seven participants forgot to log in or did not manage to complete the task because they were not able to type in their correct user name and log-in on the phone. Seven patients complained that automatic data transfer from sensor to phone (which should occur every 15 minutes) did not work properly.		Participants were confused about the difference between the diary sessions and the "remarks of today" and, in their view, there were too many sessions. In response, the sessions were renamed, the session "remarks of today" was no longer announced by email, the diary sessions were offered less often, and some text fields were enlarged. Based upon these results, all errors were solved in a new prototype and the help documentation was extended, the manual and instruction movies were adapted, and all comments about ambiguities were merged in a "frequently asked questions" file.<br><br>Several suggestions for improvement were made during the thinking-aloud procedure. The most prominent suggestions included quicker response of the app on swiping, an always visible timescale in the hour view even when there is no activity, and better distinction between the bars in the month view. Those remarks were translated into improvements in the third prototype of the app.<br><br>The dialogue session "preparation for goal setting" was rated as the most difficult. Three out of four participants completed this task on the app. This task was a long session with different input methods per question. Based on the results of the lab tests, a recommendation was added that long and complex dialogue sessions should be completed via the website rather than via the app. These sessions were: "registration", "preparation for goal setting", and "set up an activity plan".					The companies involved in the development are Maastricht Instruments BV, the Netherlands, IDEE Maastricht UMC+, the Netherlands, and Sananet Care BV, the Netherlands. The authors report no other conflicts of interest in this work.
Usability Study of a wireless monitoring system among Alzheimer's disease elderly population	2014	International Journal of Telemedicine and Applications	Abbate S. and Avvenuti M. and Light J.	https://dx.doi.org/10.1155/2014/617495 PT - Article	Healthcare technologies are slowly entering into our daily lives, replacing old devices and techniques with newer intelligent ones. Although they are meant to help people, the reaction and willingness to use such new devices by the people can be unexpected, especially among the elderly. We conducted a usability study of a fall monitoring system in a long-term nursing home. The subjects were the elderly with advanced Alzheimer's disease. The study presented here highlights some of the challenges faced in the use of wearable devices and the lessons learned.	Qualitative	Italy	Alzheimer's Disease Elderly Population	The four subjects involved in the test were from 75 to 92 years old. All of them were at staggering stages of dementia progression and associated abnormal behaviors, thus limiting the usability study. In particular, AD subjects were at levels 5/6 of Reisberg stage and they resulted below a score of 12 out of 30 (severe cognitive impairment) of the MMSE (Folstein test). All the patients were in long-term care.	a Wireless Monitoring System among Alzheimer's Disease Elderly Population			Patient's unawareness of the system's benefits is a major concern. They did not understand about the sleep tests performed. Some of them thought that the hat was to keep them warm. While someone did not move at all in the bed, assuming a supine position for the entire night, others kept removing the hat. As a result, their sleep was interrupted by the testing, as the hat had to be put back to the correct position several times.				The study suggests that ergonomic and aesthetic modifications are necessary to improve the level of usability and acceptability, especially in an elderly user population. Analysis of the users' dress code was fundamental to figure out a comfortable and easily wearable solution. Typically, the elderly are attached to a specific aesthetic dress code, characteristic of their likes/dislikes. They prefer simple, loose, and comfortable dress and therefore the focus should be on a retro style. Unfortunately, such loose dresses make it difficult to put wearable devices close to the body in order to monitor accurately. At the same time, care must be taken such that these devices should not cause itching, rashes, or skin diseases if worn too tight.					N
Evaluation of a blood glucose monitoring system with automatic high- and low-pattern recognition software in insulin-using patients: Pattern detection and patient-reported insights	2013	Journal of Diabetes Science and Technology	Grady M. and Campbell D. and MacLeod K. and Srinivasan A.	https://dx.doi.org/10.1177/193229681300700419 PT - Article	This study aimed to evaluate the performance of a glucose pattern recognition tool incorporated in a blood glucose monitoring system (BGMS) and its association with clinical measures, and to assess user perception and understanding of the pattern messages they receive.	Quantitative	UK	diabetes patients	Male gender, n (%) 44 (43.6)<br>Mean age, years (range) 44.1 (16.1–76.3)	a Blood Glucose Monitoring System with Automatic High- and Low-Pattern Recognition Software			Of those participants who clicked on the pattern message every time (74.3%) or occasionally (21.8%) to see the extra details describing the pattern, 89.3% found the extra details about the pattern useful.		Responses to the user questionnaire showed that most participants found high (84.2%) and low (83.2%) pattern alert messages clear and easy to understand. Most participants considered the frequency of low (82.0%) and high (63.4%) pattern messages about right.In all, 71.3% of participants indicated they preferred to use a meter that provides high- and low-pattern messages, whereas only 9.9% preferred to use a meter without this option.		However, 92.1% of the participants indicated that they would prefer to set the high and low blood glucose thresholds themselves, or have them set by their HCP, rather than using the default thresholds.					Mike Grady, Denise Campbell, and Kirsty MacLeod are employees of LifeScan Scotland Ltd. Aparna Srinivasan is an employee of LifeScan, Inc.
Precision, accuracy, and user acceptance of the OneTouch SelectSimple blood glucose monitoring system	2011	Journal of diabetes science and technology	Philis-Tsimikas A. and Chang A. and Miller L.		The OneTouch SelectSimpleTM blood glucose monitoring system (BGMS) is a device for self-monitoring of blood glucose designed for ease of use. System accuracy was evaluated with blood samples from 100 diabetes patients tested on 3 test-strip lots, compared with a reference system (YSI 2300 STAT). To test user accuracy, patients (n = 156) and health care professionals (HCPs) tested subject blood with the SelectSimple twice. Health care professionals evaluated subject BGMS technique after a 3-5 day home-testing period. Users evaluated the instructions for use and responded to a user acceptance questionnaire.	Quantitative	USA	Diabetes patients	Gender, n (%)<br>Female 84 (53.8)Male 72 (46.2)<br>Age, y<br>Mean 57.4 Median 58.5 Range 20.5–84.8<br>Ethnicity, n (%)<br>American Indian/Native Alaskan 1 (0.6)Asian/Pacific Islander 14 (9.0)Black/African American 5 (3.2)Hispanic/Latino 22 (14.1)White 109 (69.9)Other 5 (3.2)<br>Education completed, n (%)<br>Did not complete high school 2 (1.3)Completed high school with or without additional education 154 (98.7)	the OneTouch SelectSimple Blood Glucose Monitoring System			Patient comprehension of the instructions for use as well as patient responses to the user acceptance questionnaire fell within acceptable limits for all parameters.									Athena Philis-Tsimikas is an employee of The Scripps Whittier Diabetes Institute, which receives grants for research and educational programs from LifeScan, sanofi-aventis, Novo Nordisk, Roche Diagnostics, Bayer Diagnostics, Lilly Pharmaceuticals, Amylin Pharmaceuticals, Bristol-Myers Squibb, Merck, and AstraZeneca. Lupe Miller is an employee of LifeScan, Inc.
Design and usability of a home telerehabilitation system to train hand recovery following stroke	2009	Journal of Medical Devices, Transactions of the ASME	Durfee W.K. and Weinstein S.A. and Bhatt E. and Nagpal A. and Carey J.R.	https://dx.doi.org/10.1115/1.4000451 PT - Article	Current theories of stroke rehabilitation point toward paradigms of intense concentrated use of the afflicted limb as a means for motor program reorganization and partial function restoration. A home-based system for stroke rehabilitation that trains recovery of hand function by a treatment of concentrated movement was developed and tested.	Quantitative	USA	stoke patients	The mean age (± SD) of the subjects was 66.7 ± 9.6 years. The mean stroke duration (time from stroke onset o entrance into study) was 39.1 ± 24.8 months.	a Home Telerehabilitation System to Train Hand Recovery Following Stroke			15 saw the automated feedback as being useful and		to use the system" and that "the computer was easy to use." Fourteen could independently don and doff the sensors. Fifteen did not feel the system interfered with their daily routine, 16 found the teleconferencing sessions easy to manage. In self reported comments associated with the survey, almost all of the subjects commented on the convenience of being able to work on the treatment at their leisure, fitting it around their normal daily routine. About one quarter of the subjects had difficulty donning and doffing the sensors by themselves, which was probably correlated to severity of the stroke. The most consistent complaint was putting on the electrogoniometers independently, particularly using the paretic hand to apply the electrogoniometer to the nonparetic hand, which took some subjects 10-15 minutes or more.							UNDECLARED
Utilizing activity sensors to identify the behavioural activity patterns of elderly home care clients	2022	Disability and rehabilitation. Assistive technology	Hyvari S. and Elo S. and Kukkohovi S. and Lotvonen S. AO - Hyvari, Sauli; ORCID: https://orcid.org/0000-0002-0666-5471	https://dx.doi.org/10.1080/17483107.2022.2110951 PT - Article	The behavioural activity pattern is a behavioural and biological 24-hour rhythm. Ageing, diseases and memory disorders can change this pattern. Home care staff can utilize knowledge about the behavioural activity pattern of elderly home care clients in many ways. The purpose of this study was to evaluate whether home care staff could identify the behavioural activity pattern of elderly home care clients using activity sensors, namely, actigraphs and motion sensors, could identify the behavioural activity rhythms.	Quantitative	Finland	elderly home care clients	Female 4 Age 85.6 (±8.1)	activity sensors					The behavioural activity pattern was easy to identify from the motion sensor data, whereas actigraph data were difficult to interpret. The home care staff members' answers to open-ended questions reinforced the reliability of motion sensor data.							N
Objective Assessment of Adherence and Inhaler Technique among Asthma and COPD Patients in London: A Study in Community Pharmacies Using an Electronic Monitoring Device	2023	Pharmacy	Hesso I. and Nabhani-Gebara S. and Kayyali R. AO - Nabhani-Gebara, Shereen; ORCID: https://orcid.org/0000-0002-5784-4779	https://dx.doi.org/10.3390/pharmacy11030094 PT - Article	The INhaler Compliance Assessment (INCATM) device is an electronic monitoring device (EMD) that assesses both patient's adherence and inhaler technique (IT). This study aimed, first, to assess the value of using the INCATM device as an objective measure during medicine use review (MUR) consultations provided by community pharmacists (CPs) on patients' adherence and IT. Second, we aimed to explore patients' perceptions about the INCATM device.	Mixed	UK	Asthma and COPD Patients	Eighteen out of the 48 patients who were recruited for the observational study participated in the service evaluation. The median age of participants was 64.5 years; Age (y) 64.5 (20.3)<br>Gender, n (%)<br>Female 10 (56) Male 8 (44)<br>Respiratory condition<br>Asthma 6 (33%) COPD 12 (67%)<br>Smoking history<br>Non-smoker 5 (28) Current smoker 5 (28) Ex-smoker 8 (44)<br>Education Level<br>Primary 1 (6) Secondary 7 (39) College 6 (33) Undergraduate 2 (11) Postgraduate 2 (11)<br>Marital status<br>Single 5 (28) Married 8 (44) Divorced 1 (6) Widow/Widower 4 (22)<br>Residential area<br>Kingston Upon Thames 2 (11) Richmond Upon Thames 6 (33) Wandsworth 1 (6) Merton 6 (33) Sutton 3 (17)	Electronic Monitoring Device			Patients' positive perceptions about the INCATM technology. As for the usefulness of the INCATM feedback, 61% (n = 11/18) strongly agreed and 39% (n = 7/18) agreed to its usefulness. Thus, all patients perceived the INCATM feedback to be useful.		More than two-thirds of patients (67%, n = 12/18) found the accuhaler with the mounted INCATM device very easy to use, 5 patients found it easy (28%), whereas only one patient (5%) found it neither easy nor difficult. As for the handling of the adapted accuhaler, 61% (n = 11/18) of patients responded favourably, rating it as very manageable, and 39% (n = 7/18) as "manageable. None rated it as cumbersome or very cumbersome.<br><br>With respect to the counselling information, all patients provided positive rating, with 72% (n = 13/18) rating it as very easy and 28% (n = 5/18) rating it as easy. Similar results were obtained regarding the INCATM feedback, since 61% (n = 11/18) rated the feedback as very easy and 39% (n = 7/18) rated the feedback to be easy. The consultation style of the CP was rated as very easy (72%, n = 13/18) or easy (28%, n = 5/18) by the patients. The language used during the consultation was found very easy by half of the patients (50%, n = 9/18), and easy by 8 patients (44%, n = 8/18). Only one patient rated this as neither easy nor difficult.<br>Regarding the usefulness of the counselling information, most patients (n = 17/18) had positive responses, either strongly agree (61%, n = 11/18) or agree (33%, n = 6/18). Only one patient was undecided about this aspect.			This participant went a step further by showing concern in terms of healthcare costs because, in their particular context/case, using more reliever medication and stepping up the preventer treatment meant more costs to the NHS.		The benefit of the INCATM feedback being visible objective evidence about adherence and IT over the current standard method, which is the IT checklist, was clearly demonstrated in one case where the patient was over relying/over using the reliever inhaler and not noticing any benefit, despite having their IT checked by HCPs at the pharmacy, respiratory unit, or pulmonary rehabilitation program. The patient highlighted that none of the HCPs who examined them were able to identify any problem in the technique on the spot, which subsequently led to a discussion about stepping up the treatment.The same participant then provided an additional comment about the benefit of the feedback as evidence about patients' performance, especially when there is a poor relationship between the patient and the HCP due to the objective nature of the feedback.the feedback was useful in terms of uncovering any gaps in their daily usage, whether related to adherence or IT, which would enable them to correct these gaps to ensure better usage in the future.In one case, the use of the feedback within the consultation identified the temporal patterns of inhaler use and uncovered that the patient was using it only for symptom relief rather		N
Addressing privacy concerns for mobile and wearable devices sensors: Small-group interviews with healthy adults and cancer survivors	2022	PEC Innovation	Brannon G.E. and Mitchell S. and Liao Y.	https://dx.doi.org/10.1016/j.pecinn.2022.100022 PT - Article	Mobile and wearable sensor technology is increasingly common and accessible. The aim of this study was to explore individuals' perceptions and acceptability of mobile and wearable sensors, as well as concerns.	Qualitative	USA	General public	insufficiently active, overweight/obese adults (22 non-patients [aged 25–52] and 17 cancer survivors [aged 50–74]) were recruited to participate in the study. Of the participants, the majority (n = 36) were females and three were males. The former group were recruited from participants in previous research studies and programs through which they were exposed to wearable sensors.<br><br>To be eligible for the study, all participants were required to be (1) adults over 18 years of age, (2) overweight or obese (i.e., body mass index ≥25 kg/m2 based on self-reported height and weight), (3) insufficiently active (i.e., engage in less than 150 min of moderate-intensity physical activity per week in the past month based on self-report), (4) able to walk one block without pain or discomfort, and (5) able to speak, read, and write in English and the individuals reporting that they were	mobile and wearable devices sensors			In general, most of the participants felt that privacy concerns, as minimal as they were, did not decrease their perceptions of acceptability regarding the mobile and wearable devices.				Knowing who has access to one's health information, and the implications for how they might share or use their health information, was of great importance for several participants, but the topic was more common for non-patients than cancer survivors. Knowing who has access wasn't enough for the participants, but also knowing what the receiver might do with the information, and how that might directly affect the participant (i.e., by access to health care coverage, cost of coverage, or coverage of service status). Perceiving both unwanted access to one's private health information, as well as potential dangers associated with that (i.e., the expenses from potential increases to cost of health insurance) were the few barriers participants discussed.	n fact, the survivors perceived the sensors positively despite the identification of other potential barriers to using the mobile and wearable devices (i.e., the subcutaneous device insertions)Ownership related to data sharing was another story entirely, however, and was considered a barrier for some participants.		Most of the participants indicated they were unconcerned about their information privacy, with the perceived benefits of having their information shared with others outweighing the perceived barriers.	Participants were asked to describe their general privacy concerns about using wearable sensors like Fitbit or CGMs. Themes regarding the key concerns encompassed information privacy, data sharing, data access, and ownership, with clear links to HBM constructs, including severity, susceptibility, barriers, benefits, and self-efficacy. The primary similarity between the cancer survivor and non-patient groups was that both groups indicating fear of their health data being shared with their health insurance companies. Differences between the groups included 1) cancer survivors being freer with their health information, with looser metaphorical boundaries around their information, and 2) cancer survivors engaging in greater levels of data access (interacting with their own data virtually and allowing their health care providers virtual access as well) compared with non-patients.	N
Need for personalized monitoring of Parkinson's disease: the perspectives of patients and specialized healthcare providers	2023	Frontiers in Neurology	Evers L.J.W. and Peeters J.M. and Bloem B.R. and Meinders M.J.	https://dx.doi.org/10.3389/fneur.2023.1150634 PT - Article	Digital tools such as wearable sensors may help to monitor Parkinson's disease (PD) in daily life. To optimally achieve the expected benefits, such as personized care and improved self-management, it is essential to understand the perspective of both patients and the healthcare providers. We identified the motivations for and barriers against monitoring PD symptoms among PD patients and healthcare providers. We also investigated which aspects of PD were considered most important to monitor in daily life, and which benefits and limitations of wearable sensors were expected.	Mixed	Netherlands	Parkinson patients	Age (years), mean (SD) 67.3 (8.6) 69.1 (8.1)<br>Gender (men), n (%) 146 (71%) 136 (61%)	personalized monitoring of Parkinson's disease			Some patients were also more enthusiastic about passive monitoring			To support the communication with healthcare providers was frequently mentioned by both early and late PD patients. Obtaining insights in the effect of medication and other treatments was another important motivation for many patients. More prevalent motivations in the early PD group were (1) following the disease progression over longer time periods ("To better interpret any decline over a longer time period, and use this to have a potential prognosis, to be able to anticipate on supportive measures"), and (2) dealing with the emotions associated with having PD ("To keep having control on the disease, and to deal with it as well as possible"). Motivations mentioned more often by the late PD group were (1) to better remember symptoms ("because you cannot remember the many complaints that you come across during the day"), and (2) to be able to undertake actions yourself to improve your well-being ("The goal was to split my day into energy blocks, so I can do the most difficult activities during the hours with the most energy," and "to limit the use of medications as much as possible").On the "motivations for self-tracking" scale, both early and late PD patients scored highest on the self-design	patients showed a larger interest in monitoring fatigue, problems with fine motor movements and tremor. Regarding factors that influence symptoms, patients showed more interest in the effects of stress. Patient expressed the desire that the schedule of tasks should be personalized and that sensors should sense when the patient is not available Nevertheless, some patients doubted whether they had the discipline to do repetitive tasks	The desire not to focus too much on having PD was an important barrier for many early and late PD patients. The most common barrier among early PD patients was the fact that their PD was relatively stable. A common barrier among late PD patients was a lack of discipline or motivation. Some patients thought that keeping track of their PD was too energy-consuming. Other patients missed an easy-to-use self-monitoring tool. The fact that some patients mainly experienced "practical" barriers or never thought of tracking their PD, aligns with the fact that two-third (68%) of patients who had not tracked their PD during the previous year indicated to be interested in self-monitoring.		they responded to the statement "I believe that wearable sensors have the potential to help me monitor my Parkinson patients" with a mean score of 5.5 (neurologists), 5.5 (physiotherapists), and 5.4 (nurses). Obtaining more objective measurements was the most frequently mentioned theme across all groups. Patients who were interested in symptom monitoring, agreed that wearable sensors could provide more objective information, which would be useful to share and discuss with healthcare providers.	Healthcare providers were generally more concerned about privacy than the patients themselves. Patients were generally very open to share information with their healthcare providers, and mainly emphasized the positive aspects of data being available. Some patients mentioned that data should not be shared with insurance companies.	[illegible] this work from the Michael J Fox Foundation, UCB Biopharma, Stichting ParkinsonFonds, the Netherlands Organization for Scientific Research, and by the Dutch Ministry of Economic Affairs by means of the PPP Allowance made available by the Top Sector Life Sciences & Health to stimulate public-private partnerships. BB serves as the co-Editor in Chief for the Journal of Parkinson's disease, serves on the editorial board of Practical Neurology and Digital Biomarkers, has received fees from serving on the scientific advisory board for Biogen and UCB (paid to the Institute, not to BB), has received fees for speaking at conferences from AbbVie, Biogen, UCB, Zambon, Roche, GE Healthcare, and Bial (paid to the Institute, not to BB), and has received research support from the Netherlands Organization for Health Research and Development, the Michael J Fox Foundation, UCB, AbbVie, the Stichting Parkinson Fonds, Hersenstichting Nederland, the Parkinson's Foundation, Verily Life Sciences, Horizon 2020, the Topsector Life Sciences and Health and the Parkinson Vereniging, outside the submitted work. BB does not hold
Generational Perspectives on Technology's Role in Mental Health Care: A Survey of Adults With Lived Mental Health Experience	2022	Frontiers in Digital Health	Woerner M. and Sams N. and Rivera Nales C. and Gorstein T. and Johnson M. and Mosser B.A. and Arean P.A.	https://dx.doi.org/10.3389/fdgth.2022.840169 PT - Article	Personal technology (e.g., smartphones, wearable health devices) has been leveraged extensively for mental health purposes, with upwards of 20,000 mobile applications on the market today and has been considered an important implementation strategy to overcome barriers many people face in accessing mental health care. The objective of this study is to better understand why digital mental health tools are not as broadly adopted as predicted, what role people with lived mental health experience feel technology should play in their care and how those results compare across age groups.	Mixed	USA	Adults With Lived Mental Health Experience	Most of the sample identified as female (651/995, 65.4%), were employed (610/981, 62.2%) and did not self-identify as an underrepresented population based on skin color, heritage, socio-economic status, gender-identity, sexual orientation, or other identified characteristics (654/989, 66.1%). The sample was predominately white (750/994, 75.5%) followed by Black or African American (74/994, 7.4%), multi-racial (69/994, 6.9%), and Hispanic/Latinx (55/994, 5.5%).	App, Message Based Care, and Telehealth Usage			one in three participants (334/988, 33.8%) said they had considered using an app for this purpose.	Compared to younger and middle-aged adults, significantly fewer older adults considered an app for their mental health. Although older adults were similar to younger and middle-aged adults in their general view of mental health apps, they were less likely to rank them (1) as playing an important role in managing mental health (χ22 = 11.1, p < 0.01), (2) to manage mental health (χ22 = 15.7, p < 0.01), (3) as an effective intervention for managing mental health conditions (χ22 = 14.6, p < 0.01), and (4) were less willing to use these tools (χ22 = 25.2, p < 0.01).		Our qualitative analysis of responses found that a majority of the total sample (56%) felt that technology should play a complementary role to traditional mental health care, with 19.8% recommending technology play a major role in mental health care. This finding was shared across age groups, with one exception: older adults were more likely to indicate that technology should play no role in mental health care (10.8%), whereas only 4.9% of younger adults and 7.5% of middle-aged adults felt technology should play no role in mental health care.	When individuals were asked their preference between message-based care with or without video-conferencing, all age groups ranked supplementing message-based care with video-conferencing more favorably than message-based care without video-conferencing.<br><br>Five major themes emerged regarding the specific role that technology should play in mental health care: paired with a mental health professional, for communication purposes, symptom monitoring, improving access to care, and specific recommendations for technology in mental health.<br><br>Finally, 26% of the sample who answered this question provided detailed recommendations for technology use. These recommendations were to use technology to promote and reinforce healthy behaviors, utilize specific applications to help mental health, and for disorders that would specifically benefit from technology, such anxiety.					N
The feasibility and acceptability of mobile health monitoring for real-time assessment of traumatic injury outcomes	2021	mHealth	Jacoby S.F. and Robinson A.J. and Webster J.L. and Morrison C.N. and Richmond T.S.	https://dx.doi.org/10.21037/mhealth-19-200 PT - Article	Traumatic injuries are a health event that can begin a trajectory towards chronic health and social challenges. Mobile technology-based prevention and treatment interventions have been used to monitor and transform outcomes across a myriad of health conditions, but their potential in long-term injury recovery is unexplored. The goal of this pilot study was to assess the acceptability and feasibility of mobile health monitoring for long-term outcomes in a population of trauma patients with known barriers to health and social care after injury.	Qualitative	USA	individuals with traumatic injury	Gender<br>Male 25 (100%)<br>Race<br>African American/Black 24 (96%)Native American 1 (4%)<br>Age<br>Years 40.2 (11.9)Range (years) 21–61<br>Marital Status<br>Single, never-married 20 (80%)Married 3 (12%)Divorced 2 (8%)<br>Income<br><$10,000 9 (36%)$10,000–29,999 8 (32%)$30,000–49,999 6 (24%)$50–79 k 1 (4%)Missing 1 (4%)<br>Employment Status<br>Employed full-time 9 (36%)Employed part-time 2 (8%)Unemployed, looking for work 12 (48%)Retired 2 (8%)<br>Education<br>Some high school 2 (8%)High school graduate or GED 15 (60%)Some college 5 (20%)College graduate 2 (8%)Trade/technical training 1 (4%)<br>Primary payer at time of injury	mobile health monitoring for real-time assessment of traumatic injury outcomes			Overall, the participants found mobile monitoring to be acceptable							the benefit of convenience and the flexibility to reflect and respond to survey items at their own pace. Participants also offered additional feedback on the perceived benefit of long-term engagement with health and social services after hospitalization and the opportunity for enhanced peer-group interactions. These findings were reinforced in post-implementation interviews which highlighted, in addition to the acceptability of mobile monitoring, the value of self-monitoring through use of the Fitbit device to track physical activity and sleep on its smartphone application.	The one disadvantage cited about mobile monitoring concerned information privacy and control over who would be able to surveil physical activity and sleep data.	Conflicts of Interest: All authors have completed the ICMJE uniform disclosure form (available at http://dx.doi.org/10.21037/mhealth-19-200). The series "Real-Time Detection and Management of Chronic Illnesses" was commissioned by the editorial office without any funding or sponsorship. SFJ reports grants from Alex & Rita Hillman Foundation and the University of Pennsylvania University Research Foundation, during the conduct of the study. AR reports grants from Alex & Rita Hillman Foundation and the University of Pennsylvania University Research Foundation, during the conduct of the study. JLW reports grants from Alex & Rita Hillman Foundation and the University of Pennsylvania University Research Foundation, during the conduct of the study. TSR reports grants from National Institutes of Health/NINR, during the conduct of the study. The authors have no other conflicts of interest to declare.

A cardiovascular clinic patients' survey to assess challenges and opportunities of digital health adoption during the COVID-19 pandemic	2022	Cardiovascular Digital Health Journal	Dagher L. and Nedunchezhian S. and El Hajjar A.H. and Zhang Y. and Deffer O. and Russell A. and Pottle C. and Marrouche N. AO - El Hajjar, Abdel Hadi; ORCID: https://orcid.org/0000-0003-4820-8102 AO - Zhang, Yichi; ORCID: https://orcid.org/0000-0002-1780-7156 AO - Marrouche, Nassir; ORCID: https://orcid.org/0000-0003-0454-0541	https://dx.doi.org/10.1016/j.cvdhj.2021.10.007 PT - Article	COVID-19 boosted healthcare digitalization and personalization in cardiology. However, understanding patient attitudes and engagement behaviors is essential to achieve successful acceptance and implementation of digital health technologies in personalized care. This study aims to understand current and future trends in wearable device and telemedicine use in the cardiology clinic patient population, recognize patients' attitude towards digital health before and after COVID-19, and identify potential socioeconomic and racial/ethnic differences in adoption of digital health tools in a New Orleans patient population.	Quantitative	USA	cardiovascular clinic patients	Age in years, mean ± SD [illegible]<br>Age breakdown, n (%)<br>18–30 42 (14.0%)31–50 51 (17.1%)50–65 75 (25.1%)65+ 81 (27.1%)Unspecified 53 (17.7%)<br>Sex<br>Female 152 (50.8%)Male 142 (47.5%)Unspecified 5 (1.7%)<br>Race<br>African American 73 (24.4%)White 197 (65.9%)Asian 16 (5.4%)Hispanic 4 (1.3%)Native American/Alaskan Native 1 (0.3%)Other 1 (0.3%)Decline to specify 2 (0.7%(Missing) 5 (1.7%)<br>Education level<br>No high school 4 (1.3%)Some high school 24 (8.0%)Graduated high school 55 (18.4%)Some college 61 (20.4%)Associate degree 23 (7.7%)Bachelor's degree 47 (15.7%Graduate degree 73 (24.4%)(Missing) 12 (4.0%)	wearable device and telemedicine use in the cardiology clinic patient population			Furthermore, most patients had a favorable view toward implementing wearable devices in clinical practice				However, among users, the primary data participants were interested in was information regarding health monitoring (64.9%) . Specifically, patients wished to seek information regarding heart rhythm (66.9%), physical activity (49.2%), vital signs (47.8%), sleeping patterns (37.8%), and diet (30.4%) (Figure 3C).	High levels of costs and lack of technical knowledge were 2 commonly noted reasons for decreased usage of wearable devices (17.1% and 10.0%, respectively) (Figure 3A).		, with 60.1% stating that wearable devices could supplement routine tests and 33% believing that they could replace routine tests (Figure 3D).		Dr Marrouche reports receiving grant support and consulting fees from Abbott, Wavelet Health, Medtronic, Biosense Webster, Boston Scientific, GE Health Care, and Siemens; receiving consulting fees from Preventice; and holding equity in Marrek and Cardiac Design. All other authors have no conflicts of interest associated with the content of this manuscript.
Reliability of Wearable Sensors for Assessing Gait and Chair Stand Function at Home in People With Knee Osteoarthritis	2023	Arthritis Care and Research	[illegible] and Friscia B. and Torabian K.A. and LaValley M.P. and Gheller M. and Adamowicz L. and Georgiev P. and Viktrup L. and Demanuele C. and Wacnik P.W. and Kumar D. AO - Rose, Michael J.; ORCID: https://orcid.org/0000-0002-5400-2461 AO - Neogi, Tuhina; ORCID: https://orcid.org/0000-0002-9515-1711 AO - Torabian, Kaveh A.; ORCID: https://orcid.org/0000-0003-4265-7345 AO - Kumar, Deepak; ORCID: https://orcid.org/0000-	https://dx.doi.org/10.1002/acr.25096 PT - Article	To assess the reliability of wearable sensors for at-home assessment of walking and chair stand activities in people with knee osteoarthritis (OA).	Quantitative	USA	People With Knee Osteoarthritis	Age, years70.5 ± 4.7Body mass index, kg/m230.6 ± 4.7KOOS pain, 0–10060.2 ± 10.6KOOS ADL, 0–10068.8 ± 14.2KOOS weight-bearing painMore painful knee, 0–125.15 ± 1.35Less painful knee, 0–123.15 ± 2.06Study leg = left leg, no. (%)9 (45)Unilateral knee OA, no. (%)10 (50)Had previous knee injury, no. (%)13 (65)Sex assigned at birth, no. (%)Female17 (85)Male3 (15)White, no. (%)†19 (95)Education, no. (%)Without a college degree2 (10)Undergraduate2 (10)Graduate13 (65)Doctorate3 (15)Annual income, no. (%)< $50,0004 (20)$50,000–$150,0006 (30)> $150,0004 (20)Did not report6 (30)Currently employed, no. (%)11 (55)	Wearable Sensors for Assessing Gait and Chair Stand Function at Home			In our study, feedback from the participants showed highacceptability of our approach.		Participants reported the sensors as very easy to apply andcompletely comfortable to wear.							Dr. Neogi reported serving as a consultant for Pfizer/Lilly, Regeneron, and Novartis outside the submitted work. Dr. Kumar reported receiving grants from the National Institutes of Health during the conduct of the study for unrelated projects outside the submitted work. Lukas Adamowicz, Pirinka Georgiev, Charmaine Demanuele, and Paul W. Wacnik are employees of Pfizer with stock and/or stock options. Lars Viktrup is an employee of Eli Lilly & Company with stocks and/or stock options.
Intention to use wearable health devices and its predictors among diabetes mellitus patients in Amhara region referral hospitals, Ethiopia: Using modified UTAUT-2 model	2023	Informatics in Medicine Unlocked	Walle A.D. and Jemere A.T. and Tilahun B. and Endehabtu B.F. and Wubante S.M. and Melaku M.S. and Tegegne M.D. and Gashu K.D.	https://dx.doi.org/10.1016/j.imu.2022.101157 PT - Article	The burden of diabetes mellitus is increasing in Africa. Wearables have a proven track record of combating chronic diseases. This study aimed to assess DM patients' intentions to use wearable health devices and its predictors in Ethiopia.	Quantitative	Ethiopia	Diabetes patients	Gender Male 533 60.4Female 350 39.6<br>Age (year) 18–24 76 8.6 25–34 242 27.4 35–44 229 25.9 44 336 38.1<br>Educational level Unable to read &write 88 9.9<br>Able to read &write 232 26.3<br>Primary education 51 5.8<br>Secondary education 191 21.6<br>Higher education &above 321 36.4<br>Marital status Single 137 15.5.<br>Married 561 63.5<br>Separated 103 11.7<br>Others* 82 9.3<br>Religion Orthodox 549 62.2<br>Muslim 147 16.6<br>Protestant 136 15.4<br>Catholic 51 5.8<br>Place of residence Urban 664 75.2<br>Rural 219 24.8<br>Occupation Housewife 89 10.0<br>Government employee 220 24.9<br>NGO employee 107 12.1<br>Farmer 74 8.4<br>Marchant 249 28.2	wearable health devices			The proportion of intention to use wearable health devices was 47.1%, 95% CI (43.7-50.5).	Effort expectancy (beta = 0.543, P < 0.01), performance expectancy (beta = 0.306, P < 0.01), facilitating condition (beta = 0.131, P < 0.05), and habit (beta = 0.093, P < 0.05) had a positive direct relationship with intention to use wearable health devices. Age (beta = 0.439, p < 0.001) was moderate performance expectancy, while gender (beta = 0.780, p < 0.001) had moderate effort expectancy to the intention to use wearable health devices.								N
Clinical Targets and Attitudes Toward Implementing Digital Health Tools for Remote Measurement in Treatment for Depression: Focus Groups With Patients and Clinicians	2022	JMIR Mental Health	de Angel V. and Lewis S. and White K.M. and Matcham F. and Hotopf M.	https://dx.doi.org/10.2196/38934 PT - Article	Remote measurement technologies, such as smartphones and wearable devices, can improve treatment outcomes for depression through enhanced illness characterization and monitoring. This study aims to identify clinically meaningful targets for digital health research in depression and explore attitudes toward their implementation in psychological services.	Qualitative	UK	depression patients	Age (years), mean (SD) 36.7 (9.3) 47.9 (15.7) 47.7 (11.6) 44.6 (13.3)<br>Gender, n (%)<br>Women 5 (83) 6 (67) 7 (100) 18 (82)<br>Men 1 (17) 3 (33) —a 4 (18)<br>Ethnicity, n (%)<br>Asian or Asian British 1 (16) — 1 (14) 2 (9)<br>Black African or Caribbean, or Black British 1 (16) 3 (33) — 4 (18)<br>White British 3 (50) 4 (44) 4 (57) 11 (50)<br>White other — 1 (11) 1 (14) 2 (9)<br>Mixed or multiple ethnic groups 1 (16) 1 (11) 1 (14) 3 (14)	Digital Health Tools for Remote Measurement in Treatment for Depression								Most participants were concerned about the price of the technology and the surrounding infrastructure, including access to reliable Wi-Fi.<br><br>Participants also agreed that technology and software would need to be simple to use and convenient; for example, requiring regular charging or software updates that took time and technological literacy would affect their engagement. Participants valued the option to personalize their experience and adapt the software to their needs by deciding what to measure and manipulating the screen display.		participants believed that digital tools could complement human interaction by creating another channel of communication, which was thought to help with in-treatment discussions. Both clinicians and patients discussed how RMTs could accommodate the provision of lower-intensity extra support after treatment, for example, by having posttreatment check-ins. This technology was also thought to provide an opportunity to create a community by sharing achievements and involving others in their goals, thus providing an additional source of support. Participants thought positively of RMTs as tools for memory aids in several ways, including helping manage practicalities such as taking medication and keeping track of what improves their mood. Another way in which it was perceived as a complement to treatment was through planning and goal setting.<br><br>Increased Self-awareness Subthemes within self-awareness revolved around having a reflexive tool to identify triggers and boosters of well-being, assess treatment trajectory, positive or negative reinforcements, and the potential to worsen rumination and health anxiety. Keeping a continuous	Concerns arose regarding the reliability of the technology, especially devices with physiological readings, and how making health decisions based on unreliable sensors could potentially be harmful. Discretion was also important, as wearable devices should not identify people with psychiatric disorders. Participants preferred mobile phone apps to wearable devices; however, commercially available fitness devices or smartwatches would avoid these concerns.Information and Data Data and information management emerged among the three focus groups and revolved around five main topics: security, privacy, control over data collection, feedback, and clinician data management and workload.Conversely, 2 further subthemes revolved around the fear of overreliance on technology and a decline in human interaction. Clinicians believed that relying on a wearable device during treatment could affect relapse rates if patients became reliant on these tools and they were then taken away after treatment. Patients	Conflicts of Interest: MH is the principal investigator of the Remote Assessment of Disease and Relapse–Central Nervous System program, a precompetitive public-private partnership funded by the Innovative Medicines Initiative and the European Federation of Pharmaceutical Industries and Associations.
Deploying the Behavioral and Environmental Sensing and Intervention for Cancer Smart Health System to Support Patients and Family Caregivers in Managing Pain: Feasibility and Acceptability Study	2022	JMIR Cancer	LeBaron V. and Alam R. and Bennett R. and Blackhall L. and Gordon K. and Hayes J. and Homdee N. and Jones R. and Lichti K. and Martinez Y. and Mohammadi S. and Ogunjirin E. and Patel N. and Lach J.	https://dx.doi.org/10.2196/36879 PT - Article	Distressing cancer pain remains a serious symptom management issue for patients and family caregivers, particularly within home settings. Technology can support home-based cancer symptom management but must consider the experience of patients and family caregivers, as well as the broader environmental context. This study aimed to test the feasibility and acceptability of a smart health sensing system-Behavioral and Environmental Sensing and Intervention for Cancer (BESI-C)-that was designed to support the monitoring and management of cancer pain in the home setting.	Mixed	USA	Patients and Family Caregivers	Age band (years)<br>45-54 1 (10) 1 (20) 0 (0)<br>55-64 4 (40) 2 (40) 2 (40)<br>65-74 4 (40) 2 (40) 2 (40)<br>75-84 1 (10) 0 (0) 1 (20)<br>Rurala 8 (80) 4 (80) 4 (80)<br>Sex<br>Female 6 (60) 2 (40) 4 (80)<br>Male 4 (40) 3 (60) 1 (20)<br>Race<br>Black or African American 4 (40) 2 (40) 2 (40)<br>White 6 (60) 3 (60) 3 (60)<br>Ethnicity<br>Latino or Hispanic 0 (0) 0 (0) 0 (0)<br>Non-Latino or non-Hispanic 10 (100) 5 (100) 5 (100)<br>Highest education level<br>Less than high school 1 (10) 1 (20) 0 (0)<br>High school graduate 2 (20) 0 (0) 2 (40)<br>Some college 5 (50) 3 (60) 2 (40)<br>Professional or graduate degree 2 (20) 1 (20) 1 (20)<br>Current employment	Cancer Smart Health System to Support Patients and Family Caregivers in Managing Pain					Postdeployment Likert surveys demonstrated that, overall, patients and caregivers perceived the BESI-C system to be helpful and low burden (Table 4).Both caregivers and patients acknowledged some frustration with the technical challenges with the smart watches, including battery life, occasional lag in the touch-to-wake screen tap feature, and inconsistency with EMA delivery.		A caregiver expressed the desire for greater flexibility in describing unusual events that may influence pain.<br>Participants also expressed a desire for clearer instructions about wearing the smart watch and an interest in having the smart watch capture distress from symptoms other than pain, such as nausea.				Overall, dyads expressed a strong interest in data sharing (4.7 out of 5), with patients and caregivers equally agreeing about their desire to see their own data (4.4 out of 5), and even more strongly agreeing on the importance of sharing data with their respective partners (4.8 out of 5) and health care providers (4.8 out of 5). Interestingly, caregivers disagreed more strongly about the unobtrusiveness of the environmental sensors (3.4 out of 5) than patients (4.8 out of 5). Dyads disagreed that the BESI-C changed pain medication use (overall and patients: 2.2 out of 5; caregivers: 2.3 out of 5). Caregivers (4.4 out of 5) agreed more strongly than patients (2.6 out of 5) that recording pain events increased their awareness of pain.	The first (VL) and last (JL) authors have a pending patent application through the University of Virginia Licensing & Ventures Group related to the Behavioral and Environmental Sensing and Intervention for Cancer technology.
User acceptance of wearable intelligent medical devices through a modified unified theory of acceptance and use of technology	2022	Annals of Translational Medicine	Yin Z. and Yan J. and Fang S. and Wang D. and Han D.	https://dx.doi.org/10.21037/atm-21-5510 PT - Article	The acceptance of wearable intelligent medical devices and the factors influencing behavioral intention to use them have been scarcely studied. This study aimed to increase the current understanding of wearable intelligent medical devices and investigate the factors influencing their acceptance.	Quantitative	China	General public	questionnaires were selected for data analysis. As shown in Table 1, 62.0% of the respondents were men. Respondents' median age was 26 (interquartile range, 22–31) years. Most respondents had less than a bachelor's degree (93.1%). Regarding regional background, 78.1% lived in Eastern China, and 76.7% lived in urban areas. Most respondents' monthly income was less than 20,000 yuan (67.0%). In terms of health conditions, 84.7% had no underlying disease, and 82.9% had never undergone surgery.	wearable intelligent medical devices	only 188 (8.6%) understood and used intelligent medical products, among which WIMDs were the most widely used (49.5%).			The findings suggested that facilitating conditions (path coefficient =0.942, P<0.001) were critical to the use of wearable intelligent medical devices. Behavioral intention significantly mediated the effects of perceived risk, perceived cost, health expectation, perceived ease of use, and social influence on user behavior (path coefficient =0.210, P<0.001). Health expectation (path coefficient =0.860, P<0.001), perceived ease of use (path coefficient =0.289, P<0.001), and social influence (path coefficient =0.153, P<0.001) were found to play essential roles in predicting behavioral intention.	More than half of the users (51.6%) believed that the current intelligent medical products are relatively easy to use and that their functions can meet their current needs.			Perceived cost (path coefficient =0.034, P<0.05) and perceived risk (path coefficient =−0.031, P<0.05) had no significant effect on behavioral intention.	Of the respondents who used intelligent medical products, 47.3% primarily acquired them on their initiative, and 27.7% acquired them based on friends' recommendations.			N
What patients and care partners want in a wearable dialysis device: a mixed-methods study	2022	IISE Transactions on Healthcare Systems Engineering	Jonsdottir A.A. and Kessler L.G. and Rim S.-Y. and Kim J.-E. AO - Jonsdottir, Auur Anna; ORCID: https://orcid.org/0000-0001-5627-2951 AO - Rim, Seung-Yeon; ORCID: https://orcid.org/0000-0003-2908-938X AO - Kim, Ji-Eun; ORCID: https://orcid.org/0000-0002-8767-2971 AO - Kessler, Larry G.; ORCID: https://orcid.org/0000-0002-8024-5424	https://dx.doi.org/10.1080/24725579.2021.1958273 PT - Article	Few studies have explored the similarities and differences between patients' and care partners' perspectives in terms of their ideal dialysis procedure and desired outcomes. As part of a project to improve the quality of life of patients with end-stage renal disease, we elicited unbiased feedback from both patients and care partners regarding the design of a wearable dialysis device tailored to meet users' clinical needs.	Qualitative	USA	dialysis patients and care partners	Characteristic Patients (n = 24) Care partners (n = 12)<br>Age, years<br>Mean (SD) 65.5 (8.67) 64.25 (9.44)<br>95% CI (59.01, 69.59) (62.03, 68.97)<br>Gender, no. (%)<br>Male 24 (100) 1 (8)<br>Female 0 (0) 11 (92)<br>Ethnicity, no. (%)<br>Black 11 (46) 7 (58)<br>White 9 (37) 2 (17)<br>Other (e.g. Asian, Hispanic) 4 (17) 3 (25)<br>Education (1 = high school, 2 = associate degree/some college, 3 = bachelor's degree, 4 = master's degree, 5 = doctoral degree)<br>Median 2 2	wearable dialysis device					Patients further described the device as being simple to operate, easy to hook up to the bloodstream, and easy to put on and take off as needed.		Our analysis shows that 83% of all participants preferred carrying the device on the upper part of the body, with the back being both the patients' and care partners' most preferred location. We noticed that some participants used the word "vest" to describe their preferred location for carrying the device on the body. To distinguish these responses from the responses that mentioned only the abdomen or the back, we use the word "vest" to represent the combined region of the abdomen and back. The device should be able to be hidden from the general public under a patient's clothing, and preferably lightweight, small, flexible, and ultimately comfortable to wear. The patients expressed their desire for a device that would not restrict their movements or interfere with their daily activities, and some patients even suggested a device with wheels for easy transportation.<br><br>Other design features mentioned by the patients include a long-lasting battery that could easily be recharged, similar to the battery of a cell phone, and features to accommodate a range of different body types such as adjustable straps or a belt.<br><br>While care partners similarly imagined a lightweight and compact device, they also envisioned a device that was designed in such a way that patients could easily reach and interact with the device with or without the assistance of their care partners. The care partners indicated that they would like a device that includes features for treatment					N
Exploring the Impact and Acceptance of Wearable Sensor Technology for Pre- and Postoperative Rehabilitation in Knee Replacement Patients: A U.K.-Based Pilot Study	2022	JBJS Open Access	Cooper D.M. and Bhuskute N. and Walsh G.	https://dx.doi.org/10.2106/JBJS.OA.21.00154 PT - Article	Knee replacement operations are common, highly successful procedures that are increasing in frequency. The COVID-19 pandemic has emphasized the need for innovative care pathways that reduce face-to-face appointments. We report on the impact of introducing a wearable sensor for pre- and postoperative rehabilitation of 21 knee replacement patients at 2 hospitals in the U.K.	Quantitative	UK	Knee Replacement Patients	Participants 21<br>Study site 1 (no. [%]) 8 (38%)<br>Study site 2 (no. [%]) 13 (62%)<br>Sex<br>Male (no. [%]) 12 (57%)<br>Age* (yr) 55.08 ± 7.7<br>Female (no. [%]) 9 (43%)<br>Age* (yr) 61.33 ± 9.5<br>Overall age* (yr) 57.76 ± 8.9<br>Height* (cm) 171.26 ± 10.9<br>Body weight* (kg) 91.1 ± 16.4	Wearable Sensor Technology for Pre- and Postoperative Rehabilitation in Knee Replacement Patients			I liked using the range-of-motion sensor" (81.25%),		"Downloading the app was easy" (93.75%), "I understood how to do my rehabilitation using the range-of-motion sensor and app" (82.35%),and "I was able to use the range-of-motion sensor by myself" (88.24%).					"The range-of-motion sensor motivated me to do my rehabilitation" (88.24%), "I felt secure with the remote monitoring as the physiotherapist could see my progress" (64.71%), "I felt secure contacting the rehabilitation team via the app" (64.71%),		B Braun was employed by B Braun as Lead medical science liaison
Consumer Adoption of Digital Technologies for Lifestyle Monitoring	2019	2019 IEEE 23rd International Symposium on Consumer Technologies (ISCT)	L. Gastaldi and E. Lettieri and M. Mandolfo	10.1109/ISCE.2019.8901023	Despite their potential, the adoption of wearable devices has been relatively slow when compared to other digital technologies. This paper investigates, grounding on the Theory of Planned Behavior, the adoption by end users of digital technologies for lifestyle monitoring. Data on consumers' perception and usage of wearable devices have been collected through a survey administered to 1,000 Italian citizens and further analyzed through a Structural Equation Model approach.	Quantitative	Italy	General public	48% male, 35% high school, 69% town, 55% small town, most over 35 years old	Digital Technologies for Lifestyle Monitoring									Results show that, above the functional value of the device, external influence, particularly doctor opinion, exerts an essential role in adoption. Online health literacy proves to be a relevant factor as well, showing the importance of cultural patterns in wearables diffusion.			UNDECLARED
Activity Tracker and Elderly	2015	Conference on Computer and Information Technology; Ubiquitous Computing and Communications; Dependable, Autonomic and Secure Computing; Pervasive Intelligence and Computing	P. Rasche and M. Wille and S. Theis and K. Sch鋘fer and C. M. Schlick and A. Mertens	10.1109/CIT/IUCC/DASC/PICOM.2015.211	Wearable devices like activity tracker, measuring motion and steps, enable users to monitor their behaviour and might support a healthier lifestyle. These wearables might also motivate elderly to be active and live healthy. Unfortunately they are mostly designed for younger users and it is unclear if they are usable by older adults. In order to answer this research question a four week lasting empirical study was conducted regarding individual motivation and activity trackers' usability with 15 participants older than 60 years.	Qualitative	USA	Older adults	15 participants (8 female, 7 male) having no experience with activity tracker took part in this study (see Table II). The average age was 68 years (SD = 5.29).	activity tracker			they were willing to apply this kind of product to their daily life.		Based on the interviews participants felt comfortable using an activity tracker.The activity tracker just had one button to interact with. Participants reported this interaction design to be difficult and annoying. They had problems feeling the button under the silicon wristband.		The semi structures interviews revealed that older users prefer to use the activity tracker instead of a wristwatch. Therefore the time should be displayed continuously on the display of the activity tracker if no other data is accessed by the user. Participants also reported that it would be useful if the product is waterproof, therefore it could be worn in the shower, during swimming or aqua-fitness. Participants weighted the functions "measuring pulse" and "counting steps" higher than the other available functions. Within the group of requirements "functional range" and "data accuracy" were rated highest. Requirements like "price" or "design" were rated lowest					UNDECLARED
How Older Adults?User Experiences of Smart Healthcare System are Affected by their Cultural Differences	2021	2021 IEEE 3rd Eurasia Conference on Biomedical Engineering, Healthcare and Sustainability (ECBIOS)	C. Li and S. Xu	10.1109/ECBIOS51820.2021.9510513	Population aging is a challenging and cross-culture problem throughout the world. At present, developing smart healthcare systems is a critical way to help realize healthy aging. On the other hand, the integration of data acquisition modules in multiple non-invasive sensors and wireless local area networks makes it possible to timely find and detect health problems in older adults. This study aims at exploring the influence of older adults' culture differences on user experience design for the smart healthcare system.	Qualitative	China	older adults	As shown by the demographic analysis of subjects, women make up a larger part of the samples, accounting for 48.3%, as compared with men who accounted for 51.7%, but the overall distribution of genders was even. Subjects were aged between 60 and 75, and all of them are middle- and young-aged elderly. Specifically, 61.1% of older adults were aged between 65 and 70. In terms of educational attainment, 80.0% of subjects held diplomas of vocational school and over. Concerning income, subjects with higher than 2000 yuan account for 73.3%. In terms of living conditions, 40.0% of subjects lived alone, while 60.0% of them with other family members.	Smart Healthcare System				These data show that cultural difference indeed affects the user experience of older adults. The user experience design of the smart healthcare system has a higher attractiveness for Active Users but mediocre attractiveness for Inactive Users. It infers that the active users tend to perceive higher levels of creative and pragmatic value in the "EldLink" system, while the inactive users see it as less pragmatic and instead it may further complicate their life and reduce their quality of life.								UNDECLARED
A Quantitative Research for Determining the User Requirements for Developing a System to Detect Depression	2021	2021 International Conference on e-Health and Bioengineering (EHB)	V. -G. Sasu and B. -I. Ciubotaru and R. Popovici and A. -F. Popovici and N. Goga and G. Datta	10.1109/EHB52898.2021.9657684	Smart apps and wearables devices are an increasingly used way in healthcare to monitor a range of functions associated with certain health conditions. Even if in the present there are some devices and applications developed, there is no sufficient evidence of the use of such wearables devices in the detection of some disorders such as depression. Thus, through this paper, we want to address this need and present a quantitative research to determine the user requirements for developing a smart device that can detect depression.	Qualitative	Romania	General public	The research involved 205 participants, 73 males (35.6%)<br>and 132 females (64.4%). The age of respondents was<br>distributed as follow: under 35 – 80.1%, between 35 and 50 –<br>10,7%, between 50 and 65 – 8.3% and between 65 and 76 –<br>1.0%.	Developing a System to Detect Depression	Also, to test the level of familiarity with health monitoring systems, respondents were asked if they were using an application or a smart bracelet. 42% stated that they do use a health monitoring application, and 58% responded that they do not.						When asked about the possible purchase of a smart bracelet, 52.2% of the respondents answered negative, and 47.8% positive.<br>Therefore, 16.6% were very unlikely to purchase it, 12.7% unlikely, 21.5% likely, 22.0% quite probably, and 27.3% very likely.	In relation to other factors that respondents considered important, 45.6% costs, 7.8% efficiency, and 46.1% ease of use.				UNDECLARED

A Multiple Stakeholder Perspective on the Drivers and Barriers for the Implementation of Lifestyle Monitoring Using Infrared Sensors to Record Movements for Vulnerable Older Adults Living Alone at Home: A Qualitative Study	2022	INTERNATIONAL JOURNAL OF ENVIRONMENTAL RESEARCH AND PUBLIC HEALTH	Braspenning, AM and Cranen, K and Snaphaan, LJAE and Wouters, EJM	10.3390/ijerph19010570	A variety of technologies classified as lifestyle monitoring (LM) allows, by unobtrusive monitoring, for supporting of living alone at home of vulnerable older adults, especially persons with neurocognitive disorders such as dementia. It can detect health deterioration, facilitate early intervention, and possibly help people avoid hospital admission. However, for LM to redeem its intended effects, it is important to be adopted by involved stakeholders such as informal and formal caregivers and care managers. Therefore, the aim of this qualitative study is to understand factors that drive or impede successful implementation of LM for vulnerable older adults, specifically using infrared sensors to record movements, studied from a multiple stakeholder perspective.	Qualitative	Netherlands	older adults and caregiver	The research sample consisted of 14 respondents, 11 of whom were female. We included five informal caregivers, five health care professionals, and four health care managers. The health care professionals consisted of nurses and professional caregivers who worked in the home support, e.g., providing aid in cleaning the house. Participants' age ranged from 32 to 69 years	Infrared Sensors to Record Movements for Vulnerable Older Adults Living Alone at Home				Age sometimes plays a role and the level of social control of family or neighbors. Overall, younger informal caregivers were more keen to use LM, and people with a larger social network were less interested to use LM.	Informal caregivers, however, did not experience the burden of LM. They needed to get used to the notifications, but it was not felt to be a burden of LM.	an increased insight into the health status and daily living pattern of older adults living alone without the use of a camera, an increased independence of older adults, ultimately facilitating living at home longer, and a sense of safety through fall detection and the possibility to monitor a person without intruding privacy.	Some of the participants described that a different design of the health care system is necessary in order for health care professionals to have the opportunity to anticipate the notifications of LM by provision of the required care according to these LM notifications. In order to be able to react on LM notifications, flexibility in care is needed, resulting in new tasks and responsibilities. As an example, an informal caregiver said that if LM is telling a client has had a bad night of sleeping, it would be helpful if the health care professional has the flexibility to visit the patient at a different time than planned.	A barrier for implementation among all stakeholders was found in terms of transition of care and responsibilities. Possibilities for a responsibility shift from HCP to IC, for example, in the desired follow up of LM, was lacking and was perceived to be important for LM to redeem its effects. A passive attitude to LM by health care professionals was found, caused by lack of awareness of LM by health care professionals and different perspectives about whether technology is part of the job of a health care professional. As such, a fit of the needed way of working for LM to redeem its intended effects was not present according to this construct.			A few suggestions for improvement were addressed after using LM relating to the appearance of errors. The reliability of the LM was lacking at a few points, which made it difficult to trust LM in its use.	N
A Scalable System for Passively Monitoring Oral Health Behaviors Using Electronic Toothbrushes in the Home Setting: Development and Feasibility Study	2020	JMIR MHEALTH AND UHEALTH	Shetty, V and Morrison, D and Belin, T and Hnat, T and Kumar, S	10.2196/17347	Dental disease (including dental caries and periodontal disease) is largely preventable and closely linked to inadequate oral health behaviors. Digital health technologies have great potential for unobtrusively monitoring brushing behaviors in home settings and promoting optimal oral self-care routines at scale. The aim of this study is to leverage the ubiquity of electronic toothbrushes and smartphones with the development of a Remote Oral Behaviors Assessment System (ROBAS) and evaluate its feasibility for passively tracking brushing behaviors in real-world settings.	Quantitative	USA	General public	Age (years) and gender Less tech-savvy More tech-savvy<br>18-29<br>Female 3 3<br>Male 3 2a<br>30-44<br>Female 3 3<br>Male 2b 3<br>≥45<br>Female 3 3<br>Male 2b 2a	Monitoring Oral Health Behaviors Using Electronic Toothbrushes in the Home Setting			From a quantitative standpoint, satisfaction with ROBAS was uniformly high (Figure 8).		Most participants (85%) found the system relatively easy to use and enjoyed the experience of the electronic toothbrush. A majority expressed an interest in receiving feedback about their brushing behaviors (91%) and indicated that they would recommend ROBAS to a family member or friend (86%). When the exit interview responses were stratified by each of the baseline characteristics, none of the questions showed evidence of differences in mean by gender, age group, or tech-savviness (a=.05). However, a sizeable segment (68%) reported that they found the additional dedicated study phone to be burdensome. Some participants (6.8%) reported technical issues involving connectivity (eg, participant was unable to connect the brush to the phone app, lag in connection) or were bothered by a short battery life.							N
Improving care model for congenital heart diseases in paediatric patients using home telemonitoring of vital signs via biomedical sensors	2020	2020 IEEE INTERNATIONAL SYMPOSIUM ON MEDICAL MEASUREMENTS AND APPLICATIONS (MEMEA)	Donati, M and Panicacci, S and Fanucci, L and Saponara, S and Ait-Ali, L and Dalmiani, S and Festa, P and Mastorci, F and Pingitore, A and Penne, W and Celli, A and IEEE		About 1% of newborns have congenital heart disease at birth. This generally does not imply death, but it leads to multiple hospitalizations and outpatient visits. In addition, it results in a negative impact on quality of life for the children themselves and for their families. Telemedicine platforms can be employed in this field, to reduce the number of hospital admissions and check-ups and to improve the communication between parents/caregivers and the hospital staff. The aim of the AIR CARDIO project is to evaluate the impact, efficiency and effectiveness of a home telemonitoring system for children with congenital heart disease.	Mixed	Italy	congenital heart diseases in paediatric patients children/The evaluation of the psychological profile of parents/caregivers	The inclusion criteria are the following:<br>1) Age 0-16;<br>2) Patient with CHD undergoing cardiac surgery and/or patient with CHD awaiting surgery and/or patient hospitalized for CHD and/or arrhythmia;<br>3) Signed informed consent from parents/parental responsibility holders;<br>4) Internet connectivity at the patient's home.<br>The exclusion criteria are the following:<br>1) Age > 16;<br>2) Patients living abroad;<br>3) Lack of parental consent	g home telemonitoring of vital signs via biomedical sensors										a low level of perceived psychological well-being and poor sleep quality, associated with the child's illness, in line with what highlighted in the literature. This profile, if in the short term is linked to a poor quality of life, in the long term could predispose to the development of chronic degenerative diseases.		UNDECLARED
Experience of Emergency Department Patients With Using the Talking Pole Device: Prospective Interventional Descriptive Study	2018	JMIR MHEALTH AND UHEALTH	Yoo, J and Soh, JY and Lee, WH and Chang, DK and Lee, SU and Cha, WC	10.2196/mhealth.9676	Patient engagement is important. However, it can be difficult in emergency departments (EDs).The aim of this study was to evaluate the satisfaction of ED patients using a patient-friendly health information technology (HIT) device, the "Talking Pole," and to assess the factors relevant to their satisfaction.	Quantitative	Korea	Emergency Department Patients and caregiver	Patient (n=28) Caregiver (n=24) Total (n=52)<br>Age (years), mean (SD) 57.7 (13.9) 50.9 (10.9) 54.6 (12.9)<br>Sex, n (%)<br>Male 16 (57) 4 (17) 20 (38)<br>Female 12 (43) 20 (83) 32 (62)<br>Diagnosis category, n (%)<br>Cardiology 1 (4) 0 (0) 1 (2)<br>Gastroenterology 2 (7) 1 (4) 3 (6)<br>Infectious 5 (18) 4 (17) 9 (17)<br>Neurology 1 (4) 1 (4) 2 (4)<br>Oncology 17 (61) 16 (67) 33 (63)<br>Nephrology 2 (7) 2 (8) 4 (8)<br>Severity (Korean Triage and Acuity Scale), n (%)<br>1 0 (0) 0 (0) 0 (0)<br>2 1 (4) 0 (0) 1 (2)<br>3 15 (54) 14 (58) 29 (56)<br>4 11 (39) 9 (38) 20 (38)<br>5 1 (4) 1 (4) 2 (4)	the Talking Pole Device			Overall satisfaction with the "Talking Pole" system was measured at 4.17 points.	The overall satisfaction score was higher for the male group (4.25 points) than for the female group (4.12 points), the caregiver group (4.25 points) than for the patient group (4.11 points), the moderate-severity group (4.23 points) than for the low-severity group (4.09 points), and the general patients group (4.37 points) than for the oncology patients group (4.06 points), but statistical significance of each subgroup was not verified	The mean of participant responses was at least 4.0 points for all items. Items that evaluated interactions with medical staff, such as "call button" and "input pain score," were rated higher than those that evaluated the information display, such as "display information of username," "medical staff," "fluid infusion," and "dietary prescription" Spearman correlation coefficient showed a strong correlation of "overall satisfaction" with "comparison to the previous visit" (ρ=.73), "perceived benefit" (ρ=.73), "information satisfaction" (ρ=.70), and "efficiency" (ρ=.70); on the other hand, items related to function were low (ρ=.29-.48; see Figure 3). All correlation coefficients were significant at P=.05.							WHL is employed by Samsung Electronics.
Multi-modal data collection for measuring health, behavior, and living environment of large-scale participant cohorts	2021	GIGASCIENCE	Wu, CY and Fritz, H and Bastami, S and Maestre, JP and Thomaz, E and Julien, C and Castelli, DM and de Barbaro, K and Bearman, SK and Harari, GM and Craddock, RC and Kinney, KA and Gosling, SD and Schnyer, DM and Nagy, Z	10.1093/gigascience/giab044	As mobile technologies become ever more sensor-rich, portable, and ubiquitous, data captured by smart devices are lending rich insights into users' daily lives with unprecedented comprehensiveness and ecological validity. We developed a home environment sensor kit for continuous indoor air quality tracking and deployed it in conjunction with smartphones, Fitbits, and ecological momentary assessments in a cohort study of up to 1,584 college student participants per data type for 3 weeks.	Quantitative	USA	General public		activity tracker					Most devices worked well, but a noticeable percentage did not.		Hurdles to unobtrusiveness include the size, weight, and power need of a device, requirement for human attention and maintenance, and many potential others. A general direction of evolution for human-centric design and technology is to become more portable, convenient, and user-friendly thus affording higher and higher unobtrusiveness and ecological validity for its capacity of understanding and assisting humans, until it is truly "woven into the fabric of everyday life	Roughly 3–4% of students cited data privacy as a reason they did not want to participate. A second issue encountered was the compatibility of the smartphone application Beiwe with various software versions of iOS and Android and models of Apple and Android-compatible phones.				N
A study of the receptivity to telecare technology in a community-based elderly minority population	2007	JOURNAL OF TELEMEDICINE AND TELECARE	Bertera, EM and Tran, BQ and Wuertz, EM and Bonner, A	10.1258/135763307782215325	We examined the readiness of an elderly minority population to use various technologies for telecare.	Quantitative	USA	older adults	The average age of the study population was 73 years and the range was 55–106 years. The majoritywere women (55%) and African American (79%) or other minority group members (7%). More than one-third (37%) did not have a high school diploma andmore than half had an annual income of less thanUS $10,000 (h7459). The majority lived alone (91%)and were either divorced or separated (29%) orwidowed (29%). The demographics of the studypopulation were similar to that of the 200 residents.In order to determine which health technologieswould be most relevant to them, respondents wereasked to list their chronic health conditions. The mostcommon were arthritis (57%), hypertension (55%),diabetes (29%), chronic pain (24%) and asthma (17%).Participants also reported the barriers they perceived tomanaging these conditions. The top ranked concernswere getting enough exercise (80%) and	telecare technology			Overall, the majority of respondents agreed that healthtechnologies related to telecare would be acceptable ina wide variety of specific situations. The only exceptionwas camera-related monitoring even while they wereunwell	Respondents overwhelmingly accepted the use ofhealth technology if it enabled them to call for medicalhelp when needed or to send home monitoringinformation to their doctor (range 75–100% for allsubgroups). However, there were a fewsignificant differences. Those with at least a highschool education were somewhat more likely to accepttechnologies in both of these situations compared tothose who had less education, although the differenceonly reached significance for the calls for medical helpvariable. Elderly residents and those needing help withat least one IADL were more likely to want technologythat sent medical information to their doctor com-pared to younger respondents and those who did notneed help with IADLs						Respondents were most likely to agree or strongly agreewith health technologies that enabled sending healthinformation to their doctor, or that enabled them tocall for medical help when needed (Table 3).	Respondents were least likely to agree or strongly agree(68%) that they would accept health technologies thatallowed a nurse to check on them with a camera whenthey were unwell.	UNDECLARED
Virtual respiratory therapy delivered through a smartphone app: a mixed-methods randomised usability study	2022	BMJ OPEN RESPIRATORY RESEARCH	Baxter, CA and Carroll, JA and Keogh, B and Vandelanotte, C	10.1136/bmjresp-2022-001221	A new smartphone app (QUT Inspire) has been developed to detect inspiratory sound and deliver virtual incentive spirometry (ISy), a respiratory therapy technique used in postoperative recuperation, management of some chronic conditions and with potential applications in SARS-CoV-2 rehabilitation. The aim of this study was to compare the usability of this new app with a clinical ISy device as measured by effectiveness, efficiency and satisfaction.	Mixed	Australia	people with some chronic conditions and with potential applications in SARS-CoV-2 rehabilitation	Participant demographics<br>Gender n (%)<br>Female 14 (58%)<br>Male 10 (42%)<br>Age and age distribution 39.2±12.2 years<br>Under 25 1 (4%)<br>25–34 8 (33%)<br>35–44 2 (8%)<br>45–54 11 (46%)<br>55–64 2 (8%)<br>65 and above 0 (0%)<br>Education n (%)<br>High school 14 (58%)<br>Undergraduate degree 6 (25%)<br>Postgraduate degree 4 (17%)<br>Phone type n (%)<br>Android 10 (42%)<br>Apple 14 (58%)<br>Internet browser used n (%)<br>Chrome 8 (33%)<br>Firefox 4 (17%)<br>Safari 12 (50%)	Virtual respiratory therapy delivered through a smartphone app					all agreed that the app was user friendly, that they liked the app layout and that they were easily able to complete three inhalations using the app.While most participants (83.3%, n=20) agreed that similar effort was required to inhale using the app and the clinical ISy device, four participants (16.7%) reported that less effort was required to use the app. Most participants (87.5%, n=21) reported that they understood the screen and video instructions. Three-quarters of participants (75.0%, n=18) agreed that the timer provided motivation to persist with inspirations, and that the attempt counter was useful for tracking inhalations (83.3%, n=20).		Most participants preferred the new virtual app as best for displaying inspirations. Responsiveness of the app screen display and compactness when using the app on a smartphone were reported as advantages of the virtual ISy device. Most users favoured the smartphone app for maintaining inspiratory effort with some noting more rapid responsiveness to inspiratory sound compared with the clinical ISy device					N
Feasibility study of portable technology for weight loss and HbA1c control in type 2 diabetes	2016	BMC MEDICAL INFORMATICS AND DECISION MAKING	Bentley, CL and Otesile, O and Bacigalupo, R and Elliott, J and Noble, H and Hawley, MS and Williams, EA and Cudd, P	10.1186/s12911-016-0331-2	The study investigated the feasibility of conducting a future Randomised Controlled Trial (RCT) of a mobile health (mHealth) intervention for weight loss and HbA1c reduction in Type 2 Diabetes Mellitus (T2DM).	Mixed	UK	Overweight and obese people with diabetes	Characteristic Group 1 (n = 9) Group 2 (n = 9) Group 3 (n = 9) Total (N = 27)<br>Age in years, Mean (SD) 55.7(9.9) 52.1(7.8) 50.8(8.1) 52.9(8.6)<br>Male 0(0) 5(55.6) 7(77.8) 12(44.4)<br>Female 9(100) 4(44.4) 2(22.2) 15(55.6)<br>Ethnicity<br>British 7(77.8) 8(88.9) 8(88.9) 23(85.2)<br>Irish 1(11.1) 0(0) 0(0) 1(3.7)<br>Asian 0(0) 1(11.1) 0(0) 1(3.7)<br>African 1(11.1) 0(0) 0(0) 1(3.7)<br>Caribbean 0(0) 0(0) 1(11.1) 1(3.7)<br>Living Circumstances<br>Living with others 7(77.8) 8(88.9) 6(66.7) 21(77.8)<br>Living alone 2(22.2) 1(11.1) 3(33.3) 6(22.2)<br>Socio-Economic Status<br>Professional employmentb 3(33.3) 2(22.2) 7(77.8) 11(40.7)<br>Home owned outright 4(44.4) 2(22.2) 3(33.3) 8(29.6)	portable technology for weight loss and HbA1c control in type 2 diabetes			Three participants did seek (and were given) help in instances where they wanted to correct for errors they had made when entering exact calorific values. The focus groups mainly suggested that the device was easy to incorporate into their everyday lives, although problems were reported:<br><br>In another case a participant adapted how they carried the device even though it was not in the preferred location for accuracy:		There were 3 instances of data being lost due to faulty or lost devices—creating some gaps in the data collected.<br><br>The focus group participants reported the device was easy to use, in some cases linking it directly to adhering to increased exercise or dieting :<br><br>There were also some comments implying summer holidays may have interfered with adhering to use of the device and in following their chosen diet and physical exercise programme.<br><br>The education/training sessions around diet and exercise, and use of the device, generated mainly positive comments but with a few negatives, e.g. :<br><br>Receiving a booklet with the training information in it, the signposting to low-GI diet guides, and practical knowledge of one of the trainers in making low-cost low-GI selections and baking, were appreciated. Two participants had minor problems with device training and required extra practice during the training session.<br><br>A few individuals disliked the NLP element of the training. It asked participants to visualise themselves at their ideal weight, a task which some participants found upsetting as they felt the exercise and its implied weight loss were unrealistic.	The exercise training, while mentioned in the introduction in the focus groups, was not a specific sub-topic—although there was an open opportunity to comment. There were no comments specifically about the exercise training, positive or negative, but there were comments indicating doing more exercise (most often with reference to motivation attributed to the device)				In addition to the quotations from P1/009 to P1/023 in the preceding Adherence section there were further indications of positive motivational value of the device:	But some reported disadvantages or negative experiences (e.g. in comparison with a face-to-face intervention):<br><br>"It has not really (changed my behaviour), I've been a bit stressed and moody…it is to do with other things". P1/005<br><br>"I might have been better with a trainer or a coach". P1/026<br><br>"I haven't religiously sent the emails …. If I didn't have anything positive to say I didn't really want to send the email (laughs)". P1/018	N
Are caregivers ready for digital? Caregiver preferences for health technology tools to monitor medication adherence among patients with serious mental illness	2022	DIGITAL HEALTH	Forma, F and Chiu, K and Shafrin, J and Boskovic, DH and Veeranki, SP	10.1177/20552076221084472	Adherence to antipsychotic medication is critical for bipolar disorder (BPD), major depression (MDD) and schizophrenia (SCZ) patients. Digital tools have emerged to monitor medication adherence along with tracking general health. Evidence on physician or patient preferences for such tools exists but is limited among caregivers. The study objective was to assess preferences and willingness-to-pay (WTP) for medication adherence monitoring tools among caregivers of SMI patients.	Quantitative	USA	patients with serious mental illness and caregiver	A majority of caregivers were female (59%), Caucasian (69%), married (69%), had some college education (72%), and reported >$75,000 mean annual household income (47%). One-third of caregivers were parents of the patients with SMI, 30% were spouses/partners, 14% were children, and 22% were other types of family members. It should be noted that we did not purposively exclude non-family members from participation in the survey, and potential participants could also include friends, neighbors, or other unpaid caregivers. However, the analytic sample ultimately included participants who reported being related to the individual with SMI. Among the 184 caregivers, 57 (31%), 61 (33%) and 66 (36%) provided care to patients diagnosed with BPD, MDD and SCZ, respectively. Sixty four percent of the caregivers reported using medication adherence tools, and among them, 85% used the tools daily. In addition, although	monitor medication adherence among patients with serious mental illness			Overall, caregivers highly preferred digital adherence monitoring tools as compared to non-digital. The disutility weight of the out-of-pocket cost was 0.0078 (95% confidence interval (CI): 0.011, 0.0038). Caregivers of patients with SMI highly preferred (OR 7.34, 95% CI: 5.00–10.79) a pill embedded with IEM sensor and were willing to pay $255.04 (95% CI $123.21–$386.86) per month more for this tool than a non-digital pill organizer. Similarly, caregivers had 2.64 times higher preference (95% CI: 1.90–3.70) with a WTP $124.50 (95% CI: $48.18–$200.81) per month more for a mobile app relative to a non-digital pill organizer. The smart pill organizer was found to be less preferred by caregivers (OR) 0.54 95% CI: 0.40–0.73) with a WTP of −$78.40 (95% CI: −$129.88, −$26.92) per month, which can be interpreted as needing to pay a caregiver $78.40 per month to use the smart pill dispenser	Further, subgroup analyses by relationship found that parents, spouses, or child caregivers of patients with SMI highly preferred a pill embedded with IEM sensor than a non-digital pill organizer. However, WTP was found to be significant only for children of the patient, where they were willing to pay $227.27 (95% CI: $15.78–$438.76) per month more for the pill with IEM than a non-digital pill organizer. Compared to a non-digital pill organizer, higher preferences and WTP for a pill embedded with IEM sensor was found among caregivers irrespective of income groups (Supplementary Table 1).<br><br>Based on these preferences, a plurality of caregivers preferred a pill embedded with IEM sensor. Specifically, 39.5% of caregivers preferred a pill embedded with IEM sensor followed by a mobile app (26.6%) and a non-digital pill organizer (23.8%) (Figure 2).								The author(s) declared the following potential conflicts of interest with respect to the research, authorship, and/or publication of this article: PRECISIONheor received funding from Otsuka Pharmaceutical Development & Commercialization Inc. to conduct this research. KC is an employee of PRECISIONheor, PV and JS are former employees of PRECISIONheor. DH is an employee of Otsuka Pharmaceutical Development & Commercialization, Inc., and FF is a former employee of Otsuka Pharmaceutical Development & Commercialization, Inc.
Feasibility, Usability, and Effectiveness of a Machine Learning-Based Physical Activity Chatbot: Quasi-Experimental Study	2021	JMIR MHEALTH AND UHEALTH	To, QG and Green, C and Vandelanotte, C	10.2196/28577	Behavioral eHealth and mobile health interventions have been moderately successful in increasing physical activity, although opportunities for further improvement remain to be discussed. This study aims to investigate the feasibility, usability, and effectiveness of a machine learning-based physical activity chatbot.	Quantitative	Australia	General public	(95/116, 81.9%), in a relationship (85/116, 73.3%), White (101/116, 87.1%), and full-time workers (82/116, 70.7%). The participants had an average age of 49.1 (SD 9.3) years, with an average BMI of 32.5 (SD 8.0) kg/m2, and 81.9% (95/116) of the participants were either overweight or obese. The average step count was <6000 (SD 2391) steps/day. Only 13.8% (16/116) of the participants met the physical activity guideline. There were no significant differences in these characteristics among those with different participation durations (P>.05).	a Machine Learning-Based Physical Activity Chatbot						The average usability score for the chatbot was 61.6 (9.7) with majority of the participants scoring the chatbot as OK (89/113, 78.8%) or good (12/113, 10.6%). About one-quarter liked very much the messages that the chatbot sent out. However, only 43.4% (49/113) thought that the chatbot understood their messages most of the time. Most participants experienced technical issues (93/113, 82.3%) and stopped receiving the chatbot messages at any time during the study (95/113, 84.1%).The average usability for Fitbit was 64.0 (SD 11.1) with majority scoring the Fitbit usability as OK (77/113, 68.1%) or Good (22/113, 19.5%).				Although less than half would recommend the chatbot to others (49/113, 43.4%) and about one-third (40/113, 35.4%) would continue to use the chatbot in the future, more than half (60/113, 53.1%) agreed that the chatbot helped them become more active. About one-third thought the chatbot was quite or very useful in helping them increase confidence for engaging in regular physical activity, and in helping them stay motivated to participate in physical activity. About one-quarter thought the chatbot was useful in helping them overcome barriers, increase support they receive, and plan for physical activity during the study period.		N
The comfort of adolescent patients and their parents with mobile sensing and digital phenotyping	2023	COMPUTERS IN HUMAN BEHAVIOR	Orr, M and MacLeod, L and Bagnell, A and McGrath, P and Wozney, L and Meier, S	10.1016/j.chb.2022.107603	To integrate mobile sensing and digital phenotyping into youth mental health care, the norms of comfort among adolescent patients and their parents with mobile sensing and digital phenotyping must be understood. The current study sought to elucidate the norms of comfort among adolescent patients and the parents of adolescent patients.	Quantitative	Canada	adolescents (i.e., ages 10–21 years) who were in contact with the healthcare system for a mental health disorder (i.e., adolescent patients) or parents of adolescents receiving care for a mental health disorder.	The total sample of the current study contained 191 participants, 109 of which were adolescent patients, with ages between 11 and 21 years (M = 18.13, SD = 2.43), and 82 of which were parents reporting on their children between the ages of 10 and 21 years (M = 15.08, SD = 3.51). The majority of the patients were female (n = 102, 93.58%) and currently in treatment for a mental disorder (n = 70, 64%). Of the parents, 95% (n = 78) were mothers and slightly more than half of the children they were reporting on were female (n = 43, 52.44%). Most patients (70%) and parents (60%) were open to themselves or their child being helped by digital tips from their clinician. Most patients (96%) and parents (75%) responded that they or their child owned a mobile phone and used it daily. Similarly, most patients (96%) and parents (84%) reported that they or their child used a mobile phone to connect to the internet through Wi-Fi.	mobile sensing and digital phenotyping			For health-related data, most questionnaire responses (n = 1052) were supportive of a clinician collecting data about an adolescent patient, with the lowest level of detail (i.e., Level 1) being the most frequently supported (n = 803).	Regarding the specific data sensors to collect their personal data, 46% of adolescents and 51% of parents were comfortable sharing the total amount of photos taken on their phones, 48% of adolescents and 52% of parents were comfortable sharing the total number of videos taken on their phones, 48% of adolescents and 51% of parents were comfortable sharing the number of calls, and 49% of adolescents and 55% of parents were comfortable with the number of messages being recorded (Fig. 3). Additionally, most adolescents and parents were comfortable with typing speed (69%/60%) and screen time (67%/68%) being recorded (Fig. 3). Importantly, when the "maybe" category was included comfort levels in most of these categories increased to over 80%. Focusing on health-related data, most adolescents and parents were comfortable with the number of steps (79%/74%), time spent walking/running (79%/75%), time spent sleeping (77%/75%), heart (84%/74%) and breathing rate (84%/75%) being recorded (Fig. 3). The inclusion of the "maybe" category increased comfort levels to over 90%. For global position system (GPS) data, 50% of								N

Perspectives of Patients With Orthopedic Trauma on Fully Automated Digital Physical Activity Measurement at Home: Cross-sectional Survey Study	2023	JMIR FORMATIVE RESEARCH	Scherer, J and Yogarasa, V and Rauer, T and Pape, HC and Heining, SM	10.2196/35312	The automated digital surveillance of physical activity at home after surgical procedures could facilitate the monitoring of postoperative follow-up, reduce costs, and enhance patients' satisfaction. The aims of this study were to assess whether patients with orthopedic trauma would be generally willing to use the proposed automated digital home surveillance system and determine what advantages and disadvantages the system could bring with it.	Quantitative	Switzerland	Patients With Orthopedic Trauma	In total, 201 patients (75 female patients and 2 patients of undefined gender) whose mean age was 46.9 (SD 18.6) years were included. Most of the participants (83/201, 41.3%) were aged 30 to 55 years. Most of the patients stated that they were working from 9 AM to 5 PM (ie, an office job; 62/199, 30.8%), 18.9% (38/199) were retired, and 16.9% (34/199) reported "other." Shift work (25/199, 12.4%), part-time work (25/199, 12.4%), and self-employment (15/199, 7.5%) were the least stated answers. Most of the patients (76/201, 37.8%) were living with 1 other person, and 24.9% (50/201) stated that they were living alone (mean 2.48, SD 1.35; range 0-5).	Fully Automated Digital Physical Activity Measurement at Home			Most patients (120/201, 59.7%) stated that they could imagine using the proposed measurement system, and 18.4% (37/201) were unsure.	[illegible] and female participants (P=.41; Figure 1). Patients aged younger than 30 years were insignificantly more likely to use the proposed home surveillance system (34/47, 72%) than patients aged between 30 and 55 years (49/83, 59%) and participants aged older than 55 years (37/71, 52%; P=.25; Figure 2). In terms of occupation, there was no statistical difference between the groups regarding their acceptance of the proposed home surveillance system (P=.43). The highest acceptance was seen in patients with part-time jobs (17/25, 68%) and patients with nine-to-five jobs (42/62, 68%). The least acceptance was seen in the group of self-employed patients (5/15, 33%; P=.43). With regard to the number of persons in participants' respective households, the highest acceptance was seen in patients living with 4 other people (11/14, 79%), followed by patients living with 1 person (49/76, 65%). The least acceptance was seen in patients living with 2 other people (15/30, 50%; P=.56).						The most stated reason for being in favor of the proposed digital home surveillance system was fewer physician visits (110/201, 54.7%), followed by less effort (102/201, 50.7%) and the belief that data analysis contributes to medical progress (94/201, 46.8%). With regard to the age groups, significantly more young patients stated that higher data quality was an advantage of the proposed system (patients aged <30 years: 22/47, 47%; patients aged 30 to 55 years: 24/83, 29%; patients aged >55 years: 18/71, 25%; P=.04; Figure 3B).Significantly more patients with a part-time job or a nine-to-five job than other surveyed patients stated that data analysis contributes to medical progress and believed that this was an advantage (P=.047).	The most stated disadvantage of the proposed digital surveillance system was the missing patient-physician contact (144/201, 71.6%), followed by the lack of incidental findings (87/201, 43.3%) and possible measurement errors (77/201, 38.3%). There were no significant differences between male and female participants (P=.07).	N
A sports and health application for patients with type 1 diabetes mellitus -An end-user survey on expectations and requirements	2022	JOURNAL OF DIABETES AND METABOLIC DISORDERS	Holzer, R and Werner, F and Behrens, M and Volkery, C and Brinkmann, C	10.1007/s40200-022-01024-0	The aim of this survey is to investigate type 1 diabetes mellitus patients' expectations for and requirements of an ideal mobile self-management app with a special focus on functions for sports and exercise.	Quantitative	Germany	Diabetes patients	A total of 251 subjects participated in the survey. After verifying the inclusion criteria and completing a plausibility check, 167 respondents were included in the analyses. Reasons for exclusion were either missing T1DM as well as incomplete or inconsistent responses. Among the included subjects, 65.9% were female and 34.1% were male. The mean age of the sample was 33 ± 12 years (min-max: 8–65 years). The majority of respondents were employees (61.7%), and 6.0% were self-employed. Among the participants, 16.2% were university students at the time of the survey. One respondent (0.6%) was retired. The remaining participants (15.6%) had other employment, such as school pupil or homemaker. The monthly net income of 58.1% of participants was between EUR 1,000 and EUR 3,000. 18.6% reported a lower and 10.2% a higher monthly net income. 13.2% did not provide any information.	mobile self-management app with a special focus on functions for sports and exercise.			Most of them (83.8%; 95% CI: 78.2 – 89.4%) strongly agreed or rather agreed with the statement that there is a general need for a sports health app specifically for T1DM patients.				The key features/aspects that were deemed "rather important"/"very important" by over 75% of respondents were: data security (96.4%), integration of further health data (e.g. heart rate, step count, calories) from other apps on their smartphone (92.2%), automatic import of glucose data from other apps (91.6%), individual target setting (87.4%), warnings about abnormal glucose levels (82.6%), warnings about other abnormal health data (81.4%), diary function (80.8%), information on the completed training session (80.8%) and displaying/processing of further fitness variables (such as heart rate, step count, etc.) from other health-related wearable systems (77.8%).	The maximum price the majority of respondents would pay for such a sports health application ranged from EUR 1–10 (1–5 EUR (35.3%; 95% CI: 28.1 – 42.6%), EUR 5–10 (35.3%; 95% CI: 28.1 – 42.6%)). 8.4% (95% CI: 4.2 – 12.6%) would want to spend less, and 9.6% (95% CI: 5.1 – 14.0%) would spend more money on it. The remaining 11.4% (95% CI: 6.6 – 16.2%) would not purchase such an app at all.				C.B. is a member of the Abbott Advisory Board. Abbott had no role in data analysis, interpretation or decision to publish the manuscript. All other authors declare that they have no conflict of interest.
A gamified app for supporting undergraduate students' mental health: A feasibility and usability study	2022	DIGITAL HEALTH	Nicolaidou, I and Aristeidis, L and Lambrinos, L	10.1177/20552076221109059	Resilience, a person's mental ability to deal with challenging situations adaptively, is an important life skill. Supporting students in building psychological resilience and coping during crises (with the COVID-19 pandemic being a prime example) is crucial. Very few mobile applications (apps) for mental health explicitly report behavioral change techniques. Moreover, only a handful of the apps that support resilience are gamified, or use smartphone sensors readily available in modern smartphones for health self-management, or were designed for use by a nonclinical population. This study describes the design of a prototype for a gamified, theory-based mobile app that utilizes the Internet of Things to provide personalized data and enhance undergraduate students' resilience.	Quantitative	Cyprus	college student	A total of 74 adult participants (44 male and 30 female) with an average age of 22 years old (M = 21.86, SD = 1.78, min = 18, max = 24) tested and then evaluated Student Stress Resilience online due to social distance measures and university closures imposed due to the COVID-19 pandemic. Participants came from five different universities with the majority of them coming from the university of the authors. Most of the participants (58.1%, 43/74) were users of similar applications for health in the past, while less than half of the participants (45.9%, 34/74) were current users of a health app. The majority of current users of such applications (56.4%, 22/34) did not use them often. Approximately one in four participants (28.2%, 11/39) used a health app once or twice per month, and the same percentage of participants (28.2%, 11/39) reported that they used their health app rarely. Only 36% of participants (35.9%, 14/39) used their	A gamified app for supporting undergraduate students' mental health			Positive responses (approximating four out of five, on average) were received for the perceived usefulness of all functions (Table 1).<br><br>The lowest score was reported for the use of IoT as an objective way to measure and monitor progress, specifically the functionality that refers to the use of a sound sensor (M = 3.34, SD = 1.11) as a proposed way to measure surrounding sound and subsequently receive points concerning achieving the goal of socializing with other people. This finding may indicate participants' potential lack of familiarity with or lack of interest in using sensor-based data for health purposes, even though the use of an accelerometer was seen more positively (M = 3.70, SD = 1.04) compared to the use of a sound-sensor. This may be attributed to the value that students place on the goal of increasing their physical exercise, which was evaluated with the second-highest score in reference to descriptive statistics (M = 3.92, SD = 0.90) and the highest score (71.6%, 53/74) in reference to NPA.		The app received a satisfactory usability score. 50 Females evaluated the usability of the app (M = 77.58, SD = 12.50) significantly higher (t72 = −2.19, p = 0.032) compared to males (M = 69.66, SD = 16.91). current users evaluated the app higher compared to non-users.							N
Assessing Older Adults' Perceptions of Sensor Data and Designing Visual Displays for Ambient Environments	2014	METHODS OF INFORMATION IN MEDICINE	Reeder, B and Chung, J and Le, T and Thompson, H and Demiris, G	10.3414/ME13-02-0009	Our objectives were to: 1) characterize older adult participant' perceived usefulness of in-home sensor data and 2) develop novel visual displays for sensor data from Ambient Assisted Living environments that can become part Of electronic health records.	Qualitative	USA	older adults	We enrolled 8 community-dwelling older adults between the ages of 79–86. All eight participants participated in the three-month interviews.	Sensor Data and Designing Visual Displays for Ambient Assisted Living Environments					perceived usefulness of visual displays	As mentioned above, participants' desires to understand personal activity patterns were related to perceived usefulness of sensor data. Similarly, participants mentioned that changes in health status could affect perceived usefulness. Living situation was cited as a factor that influenced perceived usefulness. Other identified factors that affected perceived usefulness were age, the time when health monitoring begins, perceived interest level of others in accessing data and accessibility of data to others.				Participants identified specific potential uses of personal activity data for monitoring health status and capturing early signs of illness.Another participant cited the potential usefulness of personal activity data when describing those who suffer from depression. Other ideas discussed by participants about potential health applications for personal activity data included monitoring sleep interruptions due to sleep apnea, changes in bathroom activity due to administration of diuretics, changes in eating behavior and detection of social isolation.	Participants also voiced concerns about the availability of contextual information necessary to make sense of sensor data. Several brought up the point that the system would need external information to detect patterns and wondered how this would be possible. An example of an external information source is scheduling information that could enable alerts based on whether a person was away from home or motionless and in need of assistance. One participant in particular was against extra personal effort to notify the system that might interrupt his day.	N
Effects of Wearable Fitness Trackers and Activity Adequacy Mindsets on Affect, Behavior, and Health: Longitudinal Randomized Controlled Trial	2023	JOURNAL OF MEDICAL INTERNET RESEARCH	Zahrt, OH and Evans, K and Murnane, E and Santoro, E and Baiocchi, M and Landay, J and Delp, S and Crum, A	10.2196/40529	There is some initial evidence suggesting that mindsets about the adequacy and health consequences of one's physical activity (activity adequacy mindsets [AAMs]) can shape physical activity behavior, health, and well-being. However, it is unknown how to leverage these mindsets using wearable technology and other interventions. This research examined how wearable fitness trackers and meta-mindset interventions influence AAMs, affect, behavior, and health.	Quantitative	USA	General public		Wearable Fitness Trackers										. Participants exposed to deflated step counts perceived their activity as more inadequate; ate more unhealthily; and experienced more negative affect, reduced self-esteem and mental health, and increased blood pressure and heart rate (compared with participants receiving accurate step counts). Inflated step counts did not change AAM or most other outcomes (compared with accurate step counts). Participants receiving the meta-mindset intervention experienced improved AAM, affect, functional health, and self-reported physical activity (compared with participants receiving accurate step counts only).		N
A Usability Study of a Mobile Monitoring System for Congestive Heart Failure Patients	2014	E-HEALTH - FOR CONTINUITY OF CARE	Svagard, I and Austad, HO and Seeberg, T and Vedum, J and Liverud, A and Mathiesen, BM and Bendixen, OC and Keller, B and Osborne, P and Strisland, F	10.3233/978-1-61499-432-9-528	Sensor-based monitoring of congestive heart-failure (CHF) patients living at home can improve quality of care, detect exacerbations of disease at an earlier stage and motivate the patient for better self care. This paper reports on a usability study of the ESUMS system that provides continuous measurements of heart rate, activity, upper body posture and skin temperature via a sensor belt and a smartphone as patient terminal.	Qualitative	USA	Congestive Heart Failure Patients		a Mobile Monitoring System for Congestive Heart Failure Patients			The patients seemed generally happy with using the sensor belt		Reported few problems in putting it on or taking it off. The general impression from the nurses following up on the patients was that they "put the belt on and then forgot about it". The patients' actual use of the application as a source of information on own health situation was variable. Two patients only turned on the smartphone at the end of the day to transmit data stored on the belt. However, the other three patients did use the phone to view the data. Technical problems with the phones complicated the matter. The phones "froze" quite frequently, which required phone reset. The battery drained quickly, resulting in unstable operation when on low battery. Another common problem was a Bluetooth pairing instability between smartphone and the blood oxygen saturation sensor	Patient 2 was clearly inspired by it and also by the motivation heart : "I like that I can see my heart rate. Once I felt my heart beat fast I checked my phone and it was up to a 120." The following quote is from the log-book of the head study nurse, regarding the motivation heart:, after a phone call with Patient 2. "She said: I met my goal! I got my heart! She was very excited and encouraged."						UNDECLARED
Exploring User Needs and Preferences for Mobile Apps for Sleep Disturbance: Mixed Methods Study	2019	JMIR MENTAL HEALTH	Aji, M and Gordon, C and Peters, D and Bartlett, D and Calvo, RA and Naqshbandi, K and Glozier, N	10.2196/13895	Mobile health (mHealth) apps demonstrate promise for improving sleep at scale. End-user engagement is a prerequisite for sustained use and effectiveness. We assessed the needs and preferences of those with poor sleep and insomnia to inform the development of an engaging sleep app.	Mixed	Worldwide	those with poor sleep and insomnia	A total of 9 participants (6 males and 3 females, aged 21 to 70 years) were recruited.Participants were aged between 18 and 79 years (mean 38.8, SD 11.9) and predominately female (88%;)<br>Education<br>Secondary school 46 (27.5)<br>Diploma 25 (15.0)<br>Trade certificate 29 (17.4)<br>Bachelor's degree 45 (26.9)<br>Postgraduate degree 22 (13.2)<br>Employmenta<br>Full-time 53 (31.7)<br>Part-time 58 (34.7)<br>Student 17 (10.2)<br>Unemployed 20 (12.0)<br>Retired 11 (6.6)<br>Other 16 (9.6)	Mobile Apps for Sleep Disturbance			In total, 93% (156/167) of individuals indicated willingness to try an app proven to improve sleep.		User Experience Apps' effectiveness received the highest proportion of user experience mentions (29/51, 57%). The general design or esthetics received the second highest proportion of mentions (13/51, 25%).<br>Other App cost received over half of the other mentions (32/53, 60%), which were mostly critiques. In-app purchases were the second most frequently critiqued (11/53, 21%).Functionality Data synchronization was the most commonly mentioned functionality (40/135, 30%), the majority of which were critiques (29/40, 73%). Bugs were the second most frequently mentioned (41/135, 30%). Mentions of data accuracy followed (29/135, 21%), which included a high proportion of critiques (24/29, 83%; Figure 4).		App Content<br>App Features Participants generated feature ideas including:<br>Alarms for waking up and staying awake when tired, challenging alarm turn-off sequences to prevent oversleeping and a selection of alarm sounds<br>Audio features (meditation, podcasts, and relaxing sounds)<br>Encouragement (signs of progress and rewards)<br>Frequently Asked Questions were preferred to chatbots as the latter requires keyboard input<br>Recommendations for helpful apps and websites<br>Social features, in particular, the ability to share information among a community of poor sleepers. There was interest in tips from others who have successfully completed the program.<br>Unsurprisingly, there was a strong interest in sleep tracking and data visualization. As one participant [P3, FG2] stated, "Graphs appeal to me...Graphs, I like graphs." Participants expressed interest in objective data (eg, "How much you sleep per night, and how much is REM, and how much is deep sleep"[P3, FG1]) but also stressed the importance of subjective measures such as sleep quality or satisfaction explaining that duration alone can be misleading:<br><br>Tracking daily behaviors such as exercise or usage of treatments, for example, sleeping pills against sleep was important:	Cost Can Be a Motivator Making a sleep app free may undermine long-term commitment.			Only those participants with increased insomnia severity wanted to share sleep data with their doctor.	N
mHealth and patient generated health data: stakeholder perspectives on opportunities and barriers for transforming healthcare	2020	MHEALTH	Lavallee, DC and Lee, JR and Austin, E and Bloch, R and Lawrence, SO and McCall, D and Munson, SA and Nery-Hurwit, MB and Amtmann, D	10.21037/mhealth.2019.09.17	Wearable devices, mobile health apps, and geolocation technologies place the ability to track, monitor and report data in the individuals' hands - or on their bodies. These innovations create an opportunity for "connected health," where individuals collect data outside of the healthcare encounter and report it to care providers. Collection of such patient-generated health data (PGHD) has the potential to impact the delivery of healthcare through remote monitoring, and by allowing patients and healthcare teams to provide targeted and efficient care that aligns with the health status of individual patients. To understand the value and barriers associated with clinical integration of PGHD we engaged a range of stakeholders, examining their perspectives and experiences of PGHD use.	Qualitative	USA	patients and care partners	We conducted interviews with healthcare consumers, healthcare providers, and healthcare administrators, a total of 41 interviews.	Wearable devices, mobile health apps, and geolocation technologies place the ability to track, monitor and report data in the individuals' hands – or on their bodies.						Individuals decided whether to share PGHD with their providers based on whether the individual believed sharing it would further their goals for the healthcare visit. PGHD tracked solely for the purpose of wellness or advancing personal health goals were typically not shared with providers.Goals expressed by healthcare consumers for sharing PGHD with providers included improving efficiency of communication about health experience, enhancing the ability to convey health status and concerns, and providing data to inform treatment decisions. Healthcare consumers recognize that time with providers is limited and they view sharing PGHD as an opportunity to maximize the time available by preparing for healthcare discussions ahead of time and presenting data about their health in an efficient way.		Importantly, the extent to which people believe providers find tracking data useful influences the extent to which people share PGHD in the healthcare setting. Healthcare consumers report that lack of buy-in or dismissive attitudes toward PGHD by health care providers or systems can result in selecting not to continue sharing data with their providers. In some instances, providers not valuing PGHD leads individuals to find new providers who support engaged discussions of PGHD.Capturing and utilizing PGHD requires effort, time, and resources on behalf of both the healthcare consumer and, when shared, healthcare teams. While diverse modes of tracking data allow healthcare consumers to test and select methods that work best for their needs, the diversity of data types and modes presents a challenge when managing data across multiple platforms. Interoperability between devices and platforms, or single platforms that support tracking across multiple dimensions of health, would reduce this complexity and facilitate sustained use.Providers and patients also face dilemmas in how much to tailor PGHD tracking and reporting tools to patient needs and preferences. Greater personalization may reduce the burden of collecting this data for patients, but then providers must respond to more types of data from different tracking modalities presented in different formats (or using different measurement standards), thus increasing provider burden. The lack of standards around how data is tracked using different technologies, interoperability across devices and platforms, and a lack of evidence-based interpretation of data further		Healthcare consumers who track PGHD solely for self-management find it an effective tool for tracking goals and goal attainment when captured in a manner that can inform and guide behavior. PGHD also offers an effective strategy for managing multiple dimensions of health, such as medication management, monitoring symptoms, and documenting biometric data. Tracking PGHD provides healthcare consumers with an increased awareness of their health, and the ability to better engage in care with their healthcare providers.Healthcare consumers tracking PGHD report increased awareness of health, providing a sense of confidence in their ability to accurately recall past events and communicate with providers about health trends and decisions around treatment regimens. PGHD can also inform the decision to seek out care when trends in data become concerning. Healthcare consumers also note that data provides tangible evidence of health experiences that they perceive as being taken more seriously by providers than verbal or descriptions of symptoms.Positive unintended consequences of PGHD tracking included triggering the decision to pursue medical care to address changing symptoms,	Most notable is the potential for increased anxiety or emotional stress for healthcare consumers who track PGHD. This may be caused by increased awareness of declining health, hyper-focus on data, the burden associated with tracking data, and feelings of failure or inadequacy if unable to track data. Healthcare consumers also expressed that tracking PGHD can alter relationships with family members, friends, and healthcare providers. Individuals serving as care partners noted that both the process of tracking as well as discussions about PGHD could negatively impact relationships with family members or friends through perceived changes to the power balance and by creating a heightened focus on health.	N
Detecting physical abilities through smartphone sensors: an assistive technology application	2022	DISABILITY AND REHABILITATION-ASSISTIVE TECHNOLOGY	Whittington, P and Dogan, H and Phalp, K and Jiang, N	10.1080/17483107.2020.1839135	It is important to promote assistive technologies to improve quality of life. The proposed SmartAbility Android Application recommends assistive technologies for people. The Application was evaluated by pupils (n = 18) at special educational needs schools with physical conditions, including cerebral palsy, autism and Noonan syndrome, and assessed through the NASA Task Load Index (TLX) and System Usability Scale (SUS).	Quantitative	UK	pupils (n = 18) at special educational needs schools with physical conditions, including cerebral palsy, autism and Noonan syndrome	https://www.tandfonline.com/doi/full/10.1080/17483107.2020.1839135?needAccess=true#	Detecting physical abilities through smartphone sensors					Through analysis of the SUS questionnaire, 89% of participants stated that the Application was easy to use and they were not required to learn a great number of skills before using the Application. However, some participants found the buttons difficult to press and certain abilities challenging. Overall, all participants using the Application were very satisfied.							N
Validation and User Evaluation of a Sensor-Based Method for Detecting Mobility-Related Activities in Older Adults	2015	PLOS ONE	Geraedts, HAE and Zijlstra, W and Van Keeken, HG and Zhang, W and Stevens, M	10.1371/journal.pone.0137668	Regular physical activity is essential for older adults to stay healthy and independent. However, daily physical activity is generally low among older adults and mainly consists of activities such as standing and shuffling around indoors. Accurate measurement of this low-energy expenditure daily physical activity is crucial for stimulation of activity. The objective of this study was to assess the validity of a necklace-worn sensor-based method for detecting time-on-legs and daily life mobility related postures in older adults. In addition user opinion about the practical use of the sensor was evaluated.	Quantitative	Netherlands	older adults	Characteristics Total group (n = 20) Frail subjects (n = 7) Non-frail subjects (n = 13)<br>Age (year) 79.7 ± 5.7 84.1 ± 2.6 77.3 ± 5.5<br>Gender (male/female) 5/15 0/7 5/8<br>Height (cm) 165.0 ± 0.1 161.1 ± 0.1 166.5 ± 0.1<br>Weight (kg) 79.6 ± 11.2 79.7 ± 11.3 79.6 ± 11.5<br>GFI score Living conditions (independent/dependent) User of cane or walker(yes/no) 2.7 ± 2.1 16/4 9/11 6.0 ± 2.6 4/3 7/0 1.4 ± 1.1 13/0 2/11	a Sensor-Based Method for Detecting Mobility					The average score on the user evaluation questionnaire was 4.4 (SD ± 0.6; range 2.4–5.0) on a scale of 1 to 5.		Recommendations regarding improvements mainly concerned the sensor's shape: five subjects indicated that it would be a possible amelioration for the sensor to be thinner.					The authors of this manuscript have read the journal's policy and have the following competing interests: Philips Research Europe had a commercial affiliation in performing this study. Philips Research Europe provided the technology and expertise regarding the sensor equipment validated in this study, as well as support in the form of salary for W. Zhang. All technology and patents remain at Philips Research Europe. Relevant patent details are as follows: "Sit-to-stand transfer detection" (WO/2013/001411): https://patentscope.wipo.int/search/en/detail.jsf?docId=WO2013001411&recNum=100&docAn=IB2012053083&queryString=%22koninklijke%20philips%22&maxRec=26076; "Method and apparatus for identifying transitions between sitting and standing postures" (WO2014083538 A1): http://www.google.com/patents/WO2014083538A1?cl=". This does not alter the authors' adherence to PLOS ONE policies on sharing data and materials.
Usability evaluation of mobile phone technologies for capturing cancer patient-reported outcomes and physical functions	2023	DIGITAL HEALTH	Oakley-Girvan, I and Yunis, R and Fonda, SJ and Neeman, E and Liu, RY and Aghaee, S and Ramsey, ME and Kubo, A and Davis, SW	10.1177/20552076231186515	By eliminating the requirement for participants to make frequent visits to research sites, mobile phone applications ("apps") may help to decentralize clinical trials. Apps may also be an effective mechanism for capturing patient-reported outcomes and other endpoints, helping to optimize patient care during and outside of clinical trials. We report on the usability of Digital BioMarkers for Clinical Impact (DigiBioMarC & TRADE; (DBM)), a novel smartphone-based app used by cancer patients in conjunction with a wearable device (Apple Watch & REG;).	Mixed	USA	cancer patient	[illegible] of consent was 59.9, with a standard deviation (SD) of 11.6, range 31–83. A majority (86%) of participants were not of Hispanic or Latino descent, and 68% of patients identified as White (Table 2). Most (78%) participants were female. Almost all (92%) participants had completed at least some college. About half (52%) were retired or unemployed, with 32% self-employed or working full- or part-time. Almost all (98%) had adequate cancer health literacy as indicated by scores of 5 or 6 on the Cancer Health Literacy Test (CHLT-6). 15 Breast cancer patients represented about one-third of the study participants (34%), with gynecological (24%), gastrointestinal (24%), thoracic (12%), and other (6%) making up the remainder.	Digital BioMarkers for Clinical Impact (DigiBioMarC™ (DBM)), a novel smartphone-based app used by cancer patients in conjunction with a wearable device (Apple Watch®). DBM is designed to collect patient-reported outcomes and record physical functions.					The total SUS (76.6 ± 18.5), usability (74.0 ± 19.7), and learnability (86.8 ± 21.5) sub-scores were above average. 30 For the last in-app NPS, the number of "detractors" (42.9%) outnumbered "promoters" (24.5%). However, the NPS score from the last qualitative interviews was substantially higher at 24		Patients indicated that they wanted to receive more feedback from the app, and had suggestions for enhancing the content, frequency, and timing of the survey questions. While patients appreciated the simplicity and clinical appearance of the app, some suggested enhanced visuals and features. They also suggested notifications for when tasks were scheduled in the app, and suggested clarifying instructions for specific tasks.			They appreciated the prompts to increase their activity levels, which were a feature of the Apple Watch.		IOG, SWD, and RY are employed by Medable Inc., which developed the DBM app with funding from the National Institutes of Health through the Small Business Innovation Research program with the National Cancer Institute. SJF is subcontracted to work with Medable to analyze the study data.

Personalizing Sensor-Controlled Digital Gaming to Self-Management Needs of Older Adults with Heart Failure: A Qualitative Study	2020	GAMES FOR HEALTH JOURNAL	Radhakrishnan, K and Baranowski, T and O'Hair, M and Fournier, CA and Spranger, CB and Kim, MT	10.1089/g4h.2019.0222	In older persons with heart failure (HF), an inability to self-manage their disease condition can result in poor health outcomes and quality of life. With the rise in smartphone use and digital game playing among older adults, digital tools such as sensor-controlled digital games (SCDGs) can offer accessible health-promoting tools that are enjoyable and easy to use. However, designing SCDGs that are compelling and aligned with their life values and self-management needs can be challenging. This article describes a qualitative study with older adults with HF who were recruited from a cardiac rehabilitation laboratory in central Texas to identify their perceptions and expectations regarding a SCDG for HF self-management.	Qualitative	USA	Older Adults with Heart Failure	https://www.ncbi.nlm.nih.gov/pmc/articles/PMC7457967/table/tb1/	Sensor-Controlled Digital Gaming to Self-Management Needs of Older Adults with Heart Failure			Majority of the participants were receptive to the idea of using an SCDG.	Stimulating games (e.g., word or numbers games such as Sudoku) were preferred by nearly half of the participants, of whom majority had a higher educational degree; more casual puzzle game genres (e.g., casino slots or matching objects by color) were preferred by others, of whom few had a higher educational degree.	The option to earn game rewards and incentives was preferred by nearly half of the participants. Preferences for game rewards included earning higher points, levels, or trophies; receiving recipes for low-salt food items; suggestions for local restaurants offering low-salt food items; or receiving real-life rewards such as Amazon gift cards, coupons to grocery stores, or gym membership fees. Providing information and tips on managing HF was found valuable by a majority of the participants, and almost all participants favored mini-quizzes to test their knowledge and provide subsequent answers.	Setting daily goals for physical activity and tracking the progress of those goals within the game were perceived by some of the participants as a valuable motivational feature for engaging in that behavior. Avoiding negative consequences such as hospitalization was preferred as a game objective by majority of the participants. Such a goal might motivate participants to maintain their behaviors, because these participants detested hospitalizations.	Majority of the participants preferred the use of human-like avatars to elicit empathy with the character in the game, although few did not care about game characters. Human game characters that matched the older age of the participants, appeared cheerful and active, and dressed in contemporary clothing were preferred by the participants. However, some participants wanted fun animals to be offered as game characters, such as the Pink Panther, cats, or dogs.	Participants indicated that finding ways to integrate game play into their current routines would support their playing the HF game; however, falling sick, getting discouraged during game play, busy schedules, the game being too demanding, or the game being boring would negate their playing the game. However, these older adults emphasized that such a game should be simple, and that the syncing of data between the sensors and the game app should be automatic, with little effort on the player's part. Some participants were concerned about the compatibility of the SCDG with their existing tracking devices, such as a Fitbit tracker or the Apple Watch.<br><br>Some participants, however, thought that the game's goal should reflect aspects of wellness, because hospitalization might be too limiting or depressing. One participant suggested that progression in the game could be displayed visually through gradual change in the avatar's health status, so that negative consequences could be unveiled gradually rather than abruptly by a hospitalization.<br><br>Some participants preferred a dashboard to see progress in their behaviors, including the use of brightly colored stars in a calendar. Preferences for notifications included reminders for both medications, especially at nighttime, and exercising, as well as rating for completion of daily behavior goals including tips to improve the rating the next day. To enhance effectiveness, participants preferred receiving one to two	Since majority of the participants said that they would like their family members to become involved in the game or receive notifications related to the game, perhaps this could be provided as an option. Participants also reported that the games being recommended by health practitioners such as a cardiologist would enhance their belief that playing the game would be helpful.	Majority of the participants reported that SCDGs could impact their HF and quality of life by helping them become more aware of their behaviors, stay on track, establish routines, obtain education and information that would help alleviate anxiety, receive reminders for behaviors, and take charge of their health.		M.O., the owner of Good Life Games, Inc. company, which develops health games for hire, provided the prototype game images for this study. The other authors do not report any conflict of interest in conducting this study.
User preferences and usability of iVitality: optimizing an innovative online research platform for home-based health monitoring	2015	PATIENT PREFERENCE AND ADHERENCE	van Osch, M and Rovekamp, AJM and Bergman-Agteres, SN and Wijsman, LW and Ooms, SJ and Mooijaart, SP and Vermeulen, J	10.2147/PPA.S82510	The iVitality online research platform has been developed to gain insight into the relationship between early risk factors (ie, poorly controlled hypertension, physical or mental inactivity) and onset and possibly prevention of dementia. iVitality consists of a website, a smartphone application, and sensors that can monitor these indicators at home. Before iVitality can be implemented, it should fit the needs and preferences of users, ie, offspring of patients with dementia. This study aimed to explore users' motivation to participate in home-based health monitoring research, to formulate requirements based on users' preferences to optimize iVitality, and to test usability of the smartphone application of iVitality.	Qualitative	Netherlands	patients with dementia.	Variable Phase 1<br>Phase 2<br>Phase 3<br>Phase 4<br>Interview 1 (n=3) Focus group (n=6)<br>Interview 2 (n=2) Usability evaluation (n=4)<br>Sex (male/female) 1/2 1/5 0/2 1/3<br>Age, years (male/female) 53/55, 62 53/42, 55, 58, 63, 64 50, 51 53/43, 61, 62<br>Highest education<br>Middle 1 of 3 4 of 6 – 3 of 4<br>High 2 of 3 2 of 6 2 of 2 1 of 4<br>Experience in using a 2 of 3 3 of 6 2 of 2 2 of 4<br>smartphone (n)<br>Experience in using 3 of 3 6 of 6 2 of 2 4 of 4	iVitality consists of a website, a smartphone application, and sensors that can monitor these indicators at home.			They perceived the smartphone application as an important, user-friendly, and useful research tool.		Participants experienced problems related to, for example, unclear notifications, menu navigations, and unclear graphical presentations. Although feedback was appreciated by all participants with mild or severe hypertension, only one participant would intend to visit his/her general practitioner to discuss this. All participants indicated a willingness to wear their phone in a pocket for monitoring of physical activity. At the end of the usability test, participants were more experienced in using the smartphone application.<br><br>Participants perceived the smartphone application as useful (mean score 5.50±1.71 on a scale of 1–7, with a higher score indicating better usability) and of good information quality (mean score 5.96±63) and interface quality (mean score 5.68±1.83).	Participants scored high on autonomous regulation (ie, intrinsic motivation) to use the smartphone application (mean score 5.44±2.42) and low on controlled regulation (ie, extrinsic motivation, mean score 1.32±0.67).<br><br>Results of the interviews (n=3) revealed that the participants were highly motivated to contribute to research aimed at increasing knowledge about dementia.	lengthy period of time (eg, 6 months) due to work or exceptional circumstances such as vacations. In addition, not all participants preferred receiving daily questions about their lifestyle. This would remind them and keep them too preoccupied with the idea of being at risk for dementia. Further, participants preferred some adjustments in the graphical representation of their measured data. A graphical output of the first version of the smartphone application is shown in Figure 2A. Some participants mentioned preferring a reference line in the graph reflecting blood pressure values indicating the accepted range of blood pressure according to their age group. Therefore, during phase 4, the usefulness of a new graphical design was tested, representing the measured blood pressure values along with a reference line as shown in Figure 2B. In addition, participants indicated that they would like to receive information about blood pressure and lifestyle regarding dementia alongside their data. This could increase their awareness of the relationship between health, lifestyle, and dementia.In summary, although participants stated to have the intention to use the iVitality smartphone application (as both phases 1 and 2 revealed), its perceived usefulness and ease of use depends on the measurement protocol (ie, time interval, type of questions) and its visual abilities/features.						N
User preference for an IoT healthcare application for lifestyle disease management	2018	TELECOMMUNICATIONS POLICY	Kim, S and Kim, S	10.1016/j.telpol.2017.03.006	The current convergence between information and communications technology (ICT) and medical fields reflects a shared vision of seamlessly connected sensors and devices that can improve healthcare services, and an expectation of the emergence of Internet of Things (IoT) healthcare. This study provides a relevant guide for IoT healthcare service developers, from the perspective of the technology user.	Quantitative	Korea	patients		an IoT healthcare application for lifestyle disease management				, while medical history has a considerable influence on the perceived importance of the proposed attributes.			The results showed that potential users, in general, require a safe and trustworthy healthcare service, rather than greater functionality					UNDECLARED
Patients' Perception of the Use of the EasyPod (TM) Growth Hormone Injector Device and Impact on Injection Adherence: A Multi-Center Regional Study	2022	FRONTIERS IN PEDIATRICS	Deeb, A and Al Yaarubi, S and Bin Abbas, B and Al Jubeh, J and Chaturvedi, D and Al Hassani, N and Mutair, A and Al Masri, N and Al Sanad, Y and Al Shidhani, A and Mahmoud, NS and Alherbish, A and Savage, MO	10.3389/fped.2022.839278	This study aimed to assess patient perceptions of the use of the EasyPod (TM) growth hormone delivery device and its association with compliance.	Mixed	United Arab Emirates, Oman, and Saudi Arabia,	Children and adolescents aged 3–18 years, diagnosed with growth disorders and caregivers	[illegible] from six centers in three countries (Center 1: 32, Center 2: 12, Center 3: 17, Center 4: 65, Center 5: 40, Center 6: 20). The mean age ± SD of the enrolled patients was 11.8 ± 2.76 years. There were 117 males and 65 females in the study. Gender information was missing for four patients. The mean ± SD duration of treatment with growth hormone was 3.74 ± 2.9 years. The mean percentage of compliance recorded was 87% (range: 50–100%). A hundred patients (53.76%) had injection compliance of ≥90%; mean compliance in this group was 95% (range: 90–100%). The average duration of use of the device in these patients was 3.49 ± 2.98 years compared to 3.56 ± 2.33 in the 86 patients with <90% compliance (mean compliance of 79%; range: 50–89%). Twenty-nine patients had 100% compliance and the average duration of use in these patients was 2.57 ± 2.76	the Use of the EasyPod™ Growth Hormone Injector Device and Impact on Injection Adherence					Among the tracking features, a statistically significant higher proportion of patients (78%) in the ≥90% compliance group reported the feature of the history of injected and missed doses to be "very useful" as compared to 47.7% in the <90% compliance group (<90% vs. ≥90%, [χ2 (1) = 23.266, p < 0.001]) (Table 2), with a mean rank of 75.92 in the <90% compliance group and 108.62 in the ≥90% compliance group.<br><br>The feature of medicine left in cartridge reminder and the battery power indicator was rated to be "very useful" by 46 and 48%, respectively, by patients in the higher compliance group (Table 2). This was significantly higher than the patients in the lower compliance group (23.3 and 20.9%) (p < 0.001).<br><br>Among highly compliant patients, 80% found the tracking features of the device to be useful in tracking missed doses and in encouraging their child to be more compliant. In the higher compliance group, 48% of patients reported no pain experience on using the device (score of 1/5) and none reported a score of 5/5 (indicative of severe pain). The requirement of keeping the device in the fridge was reported as the most inconvenient feature in the higher compliance group by 56% of patients as compared to 39.5% patients in the lower compliance group (p-value <0.05).							This publication received funding from Merck Serono Middle East FZ-LTD. The funder was not involved in the study design, collection, analysis, interpretation of data, the writing of this article, or the decision to submit it for publication. This publication is done via an unrestricted medical writing grant by Merck Serono Middle East FZ-LTD, an affiliate of Merck KGaA, Darmstadt, Germany. The authors declare that the research was conducted in the absence of any commercial or financial relationships that could be construed as a potential conflict of interest.
Smart textiles: transforming the practice of medicalisation and health care	2019	SOCIOLOGY OF HEALTH & ILLNESS	Joyce, K	10.1111/1467-9566.12871	Smart textile medical devices are forms of clothing that use sensors and fabrics to monitor bodily processes and communicate with data systems through wireless transmission. To investigate the co-evolution of digital technologies and health care practices, this study draws on focus group and fieldwork data to analyse the sociological implications of the creation of two smart textile devices: one - the bellyband - will replace the tocodynamometer and foetal heart rate monitor during labour and birth in hospitals and the other - the babyband - will replace the cardiopulmonary monitor in neonatal intensive care units.	Qualitative	USA	women who had given birth in the last 5 years and birth partners who had supported a woman during birth in the last 5 years.		Smart textile medical devices are forms of clothing that use sensors and fabrics to monitor bodily processes and communicate with data systems through wireless transmission.			Across all user groups, participants fully supported the use of bellyband and babyband in hospitals and clinics.		Across participants, a soft fabric that allowed people to move evoked ease and comfort.	Participants' enthusiasm for the bellyband was generated in part by comparing it to their experiences with existing machines. the devices "tether" labouring women.<br>In addition to the language of tethering, participants used language like "tied down" or "restrictive" to describe current systems of monitoring. A midwife stated, for example, "If the mom is stuck in a bed, stuck to a machine, you feel like you're tied down." Participants imagined the baby being able to move freely and to be touched more easily without the encumbrance of cords.	A majority of participants in all stakeholder groups agreed that the more functions that could be included in the bellyband, the better. Although recognising the scientific and design challenges, such an aim may represent, most participants spoke of the need to include maternal heart rate, foetal heart rate, foetal contractions and baby movement in the bellyband. The key reason for this was to decrease the number of machines attached to women in labour.<br><br>Although participants were enthusiastic about the devices' use in hospitals, they overwhelmingly rejected the proposed expansion of monitoring into the home during routine pregnancies or a baby's first year or two of life. Participants highlighted three main reasons for this position. First, across all groups, participants thought using the device at home without clinician oversight would increase prospective and current parents' anxiety. Another mother highlighted how the OTC babyband would increase the anxiety or what she called "the crazy." Second, participants who worked in the health sector were concerned that OTC sales would create more work for clinicians who would get called when people thought there was an unusual reading.Finally, doctors, doulas, midwives and nurses emphasised that there is no medical evidence that monitoring would have any impact on pregnancy or infant outcomes.<br><br>Although participants wholeheartedly rejected at home, OTC use,			For users, the wireless feature of the bellyband created a patient who could move, which in turn supported women as they laboured to give birth.		UNDECLARED
iTex Gloves: Design and In-Home Evaluation of an E-Textile Glove System for Tele-Assessment of Parkinson's Disease	2023	SENSORS	Ravichandran, V and Sadhu, S and Convey, D and Guerrier, S and Chomal, S and Dupre, AM and Akbar, U and Solanki, D and Mankodiya, K	10.3390/s23062877	Parkinson's disease (PD) is a neurological progressive movement disorder, affecting more than 10 million people globally. In this work, we present an in-home telemedicine kit, named iTex (intelligent Textile), which is a patient-centered design to carry out accessible tele-assessments of movement symptoms in people with PD.	Quantitative	USA	patients with Parkinson's Disease	Four individuals (two male, two female) were recruited to the study with ages ranging from 54 to 86 years. The participants had been diagnosed with PD between 2 and 19 years.	Design and In-Home Evaluation of an E-Textile Glove System for Tele					Although all participants found the application to be straightforward to use, some participants felt the questionnaire page was moderately difficult to use with their tremors. All participants found the magnetic charging cable to be helpful to charge the gloves system. Participant 3 reported that he found the number of cables for powering tablets and the gloves to induce more stress and wondered if better cable management of wireless power could be used in the future. From the self-reported measures in Figure 13, we can see that participants primarily reported slight and mild tremor and dyskinesia symptoms. Multiple participants had reported that they found the questionnaire response selection drop-down to be difficult to use particularly while off medication. The glove-wearing and removal process was reported to be tedious by certain participants particularly when they were off-medications. Fit issues were also present with participants' larger hand sizes. These highlight the need for personalized gloves and incorporating usability-enhancing design mechanisms.							N
Smart homes for older people involved in rehabilitation activities - reality or dream, acceptance or rejection?	2018	BALNEO RESEARCH JOURNAL	Sitar-Taut, AV and Sitar-Taut, DA and Cramariuc, O and Negrean, V and Sampelean, D and Rusu, L and Orasan, O and Fodor, A and Dogaru, G and Cozma, A	10.12680/balneo.2018.199	Recent statistics show an increase in the prevalence of the elderly population. The year 2012 was declared European Year for Active Ageing and Solidarity between Generations, and the European Commission launched campaigns like The Active and Assisted Living Joint Program (AAL JP). Rehabilitation in the elderly is a desideratum, but the problems of rehabilitation in the elderly are numerous. The aim of the study was to evaluate degree of acceptance/implementation of different technologies in Romania, of monitoring in the rehabilitation activity conducted at home.	Quantitative	Romania	older adults	The study comprised 154 persons with a mean age of<br>73.37 ± 7.33 years, of which 64 (41.6%) male and 90<br>(58.4%) female.	Smart homes for older people involved in rehabilitation activities			71.9 % of patients agreed to wear a portable sensor,;33.1% accepted videocam with no significant<br>differences between sexes (p=0.8);47.4% accepted a screen with no significantdifferences between sexes (p=0.8);41.3% accepted living room monitoring with nosignificant differences between sexes (p=0.9);<br>68% accepted sensor in the room, on the wall with no significant differences between sexes (p=0.3);<br>69.1% accepted fall detection sensor with no significant differences between sexes (p=0.4).	No significant differences were found regarding the acceptance vs<br>rejection of personal sensors, living room monitoring,<br>sensors in the room, fall detection sensors.	Less than 50% of the<br>patients considered the mobile phone, notebook or<br>tablet easy to use, pleasant.			The main reasons for system rejection were<br>represented by fear of losing the device, perception of<br>no need, and privacy concerns.				UNDECLARED
Engaging across dimensions of diversity: A cross-national perspective on mHealth tools for managing relapsing remitting and progressive multiple sclerosis	2019	MULTIPLE SCLEROSIS AND RELATED DISORDERS	Simblett, SK and Evans, J and Greer, B and Curtis, H and Matcham, F and Radaelli, M and Mulero, P and Arevalo, MJ and Polhemus, A and Ferrao, J and Gamble, P and Comi, G and Wykes, T and RADAR-CNS Consortium	10.1016/j.msard.2019.04.020	Smartphone apps and wearable devices could augment clinical practice by detecting changes in health status for multiple sclerosis (MS). This study sought to investigate potential barriers and facilitators for uptake and sustained use in (i) people with both relapsing remitting MS (RAMS) and progressive MS (PMS) and (ii) across different countries.	Qualitative	UK, Spain, Italy	Participants received a diagnosis of MS.	[illegible] 24 people across three countries (UK Group 1a N9; UK Group 1b N5; Spain N9; Italy N6). Participants in Italy had been living with MS for longer, in Italy and Spain all were white, and in Italy only one participant was a woman. Diagnosis (RRMS or PMS) was evenly distributed across countries (see Table 1) and there were no differences between diagnoses in age (t (1, 21) = 0.13, P = .99) or time since diagnosis (t (1, 21) = −0.04, P = .97). Age was also similar across countries (see Table 1).Empty Cell UK (n = 9) Spain (n = 9) Italy (n = 6)<br>Gender: n female (%) 6 (66.7) 4 (44.4) 1 (16.7)<br>Age: mean (SD) 43.4 (9.5) 54.2 (9.8)	mHealth tools	Two participants reported that they had no previous experience of mHealth technologies. Other participants reported using activity trackers and smartwatches as well as smartphone apps for health. Common uses had included managing diet and weight and fitness/exercise, including tracking heartrate.						The majority of the evidence falling under the themes of convenience, accessibility, usability and intrusiveness of technology were unique to MS. This highlights the necessity of considering condition-specific requirements in the design of mHealth technologies. Choice and control was a large category emerging. Co-production and careful user-testing are likely to be key determiners, which can accommodate fluctuating or progressive disabilities. This echoes the conclusions of previous studies that tools need to be designed with specific disabilities in mind (Engelhard et al., 2017).	articipants spoke about the potential impact on the validity and reliability of results if these needs are not taken into account, regardless of whether symptoms are episodic (e.g. RRMS) or enduring (e.g. PMS). Other factors such as the impact of poor motivation, insight and forgetfulness were also important to consider as part of the design of mHealth technologies but may not be specific to MS (Simblett et al., 2018).		Perceived utility of mHealth for providing information, raising awareness and enabling a greater level of accuracy than human assessment	Perceived costs associated with negative impact on mood, concerns about privacy and potential inaccuracy, which needed to be balanced against perceived utility	N
The Use of Wearable Activity Trackers Among Older Adults: Focus Group Study of Tracker Perceptions, Motivators, and Barriers in the Maintenance Stage of Behavior Change	2019	JMIR MHEALTH AND UHEALTH	Kononova, A and Li, L and Kamp, K and Bowen, M and Rikard, RV and Cotten, S and Peng, W	10.2196/mhealth.9832	Wearable activity trackers offer the opportunity to increase physical activity through continuous monitoring. Viewing tracker use as a beneficial health behavior, we explored the factors that facilitate and hinder long-term activity tracker use, applying the transtheoretical model of behavior change with the focus on the maintenance stage and relapse.The aim of this study was to investigate older adults' perceptions and uses of activity trackers at different points of use: from nonuse and short-term use to long-term use and abandoned use to determine the factors to maintain tracker use and prevent users from discontinuing tracker usage.	Qualitative	USA	older adults	white, female, college/graduate	Wearable Activity Trackers	Many participants occasionally referred to themselves as being laggards and luddites, that is, not being technologically savvy.	Short-term users described themselves as having health issues and being active. Long-term users also mentioned having health issues but were more likely to describe themselves as being technologically savvy and early technology adopters.				Several nonuser participants named long-term benefits, such as staying away from medications, feeling better, losing weight, and improving physical indicators of health (heart rate and blood pressure), as motivations that could drive tracker use.<br><br>Participants agreed that tracker use motivated them to walk more, driven by quantifying activity (counting steps) and continuously making users "more conscious of extra walking" (female participant, nonuser):<br><br>Short-term and former users talked about the same tactics that nonusers did, with an addition of 2 new tricks: using a red-line activity tracker alert of inactivity and shopping.<br><br>Short-term and former users were also motivated by the presence of others: either participants competed with their friends and family members in numbers of steps taken, were supported by them (eg, being given a tracker as a gift), or simply observed them using activity trackers:	After trying activity trackers, nonusers found 4 features and functions to be useful: it could be used as a watch to show date and time, it had a comfortable band, it tracked sleep, and it was waterproof. Interestingly, the step count feature was not a favorite feature among the nonusers as most perceived step quantification to be inaccurate.<br><br>Although they continued to suggest that activity trackers should be aesthetically appealing and not ugly, they focused on activity tracker band comfort, which, according to many participants, Garmin VivoFit 2 lacked. Nonusers described the band as plastic (ie, cheap and of bad quality), clunky, annoying, rigid, and uncomfortable:<br><br>Short-term and former activity tracker users identified simple usage patterns by focusing on the importance of step count. They also wished that activity trackers were more user friendly for older adults when they discussed device maintenance:Short-term users expressed an appreciation of heart rate monitoring feature and wished to have features for better vision and a stopwatch function on their ideal activity trackers. Former users liked the sleep-tracking feature and wanted an activity tracker that measures blood pressure and provides accurate information.<br><br>long-term users identified motivational messages and reminders, pace tracking, and calories burned as being important:Time feature, in	Participants in the former and shot-term user focus groups appeared to like their routines and established exercise schedule, to which activity trackers did not add.<br><br>Tracker nonusers mostly generated questions and speculations about how trackers worked。Participants explained that physical activities were very diverse and had different levels of intensity that no technology could grasp. Their skepticism toward and disappointment with the technology increased because they found that activity trackers had given inaccurate information.<br><br>Other reasons for nonadoption included high price, physical limitations that older adults face as well as inactive lifestyle, little interest and curiosity in trying activity trackers, band discomfort, and technology use difficulties.<br><br>Short-term and former users added that defects in activity trackers could or did make them stop using the activity tracker. One short-term user briefly discussed the issue of keeping personal data private when information from the activity tracker is synchronized with a mobile phone or computer and shared on social networks.<br><br>Former users indicated that long-term use of activity trackers was not necessary as one gets an impression of numbers related to certain				N
Sharing patient-generated data with healthcare providers: findings from a 2019 national survey	2021	JOURNAL OF THE AMERICAN MEDICAL INFORMATICS ASSOCIATION	Turner, K and Jo, A and Wei, G and Tabriz, AA and Clary, A and Jim, HSL	10.1093/jamia/ocaa272	Our study estimates the prevalence and predictors of wearable device adoption and data sharing with healthcare providers in a nationally representative sample. Materials and Methods: Data were obtained from the 2019 Health Information National Trend Survey. We conducted multivariable logistic regression to examine predictors of device adoption and data sharing.	Quantitative	USA	Patient	Age, mean (SD), y 55.75 (16.61) 61.16 (14.67) <.0001 61.10 (14.13) .829<br>Gender, no. (%)<br>Male 1,803 (43.35) 570 (45.82) .008 259 (44.97) .290<br>Female 2356 (56.65) 674 (54.18) 317 (55.03)<br>Race, no. (%)<br>Black 698 (16.78) 236 (18.97) .017 113 (19.62) .693<br>White 3461 (83.22) 1008 (81.03) 463 (80.38)<br>Ethnicity, no. (%)<br>Hispanic 613 (14.74) 196 (15.76) .998 78 (13.54) .050<br>Non-Hispanic 3546 (85.26) 1048 (84.24) 498 (86.46)<br>Education, No. (%)<br>High school diploma or less 2105 (50.61) 680 (54.66) .001 295 (51.22) .126<br>College degree 1184 (28.47) 305 (24.52) 152 (26.39)<br>Postgraduate 870 (20.92) 259 (20.82)	wearable device			The sample contained 4159 individuals, of whom 29.9% had adopted a wearable medical device in 2019 (Table 1). Among adopters, 46.3% had shared PGD with their healthcare provider.	In the multivariable analyses, individuals with Hispanic ethnicity, a usual source of care, diabetes, hypertension , and multiple chronic conditions had significantly higher odds of device adoption holding all else constant. Individuals with household income of $50 000–$74 999 and $75 000+ had significantly higher odds of device adoption compared with individuals who had a household income less than $20 000 annually.Among adopters, individuals with Black race, insurance, a usual source of care, diabetes, and hypertension had significantly higher odds of sharing PGD with a provider holding all else constant. Individuals with household income of $35 000–$49 999, $50 000–$74 999 and $75 000+ had significantly higher odds of sharing PGD with a provider compared with individuals with household income less than $20 000 annually.								Dr. Jim has consulted for RedHill BioPharma, Janssen Scientific Affairs, and Merck.
Usability of a wearable device for home-based upper limb telerehabilitation in persons with stroke: A mixed-methods study	2023	DIGITAL HEALTH	Toh, SFM and Gonzalez, PC and Fong, KNK	10.1177/20552076231153737	The use of wearable technology offers a promising home-based self-directed option for upper limb training. Although product usability is a crucial aspect of users' acceptance of a wearable device, usability studies in wearable devices are rare, with most studies focusing primarily on clinical validity. This study aimed to explore the usability of a wristwatch device called "Smart reminder" for home-based upper limb telerehabilitation for persons with stroke.	Mixed	Hong Kong, China	persons with stroke	Characteristics of the participants (n = 11).<br><br>Demographics<br>Gender (n)<br>Female 7<br>Male 4<br>Age (years)<br>Years (mean ± SD) 55.9 (11.1)<br>Young (<48 years old) 2<br>Middle aged (48–63 years old) 5<br>Older (>63 years old) 4<br>Time of stroke onset (years)<br>Years (mean ± SD) 6.3 (3.1)<br>>1 year to 5 years 4<br>>5 years 7<br>Upper limb severity, FTHUE-HK (levels)<br>FTHUE-HK: 3 to 5 (low functioning) 7<br>FTHUE-HK: 6 to 7 (high functioning) 4	a wearable device for home-based upper limb telerehabilitation in persons with stroke	Experience with wearable device(s)<br>Yes 1<br>No 10		an "excellent" acceptability level.		an "above average" usability score. The effort expectancy theme/construct is defined as the degree of ease the user associates with using the device.44,47 All participants unanimously shared that the device was user-friendly and easy to use. In addition, some participants found it convenient to use because it allowed them flexibility in the location and time when they used it. Nevertheless, for this construct, the participants highlighted two main challenges: technical issues and the wearability of the device.Donning the watch was difficult for most participants, especially one-handedly, and two participants required assistance from their caregivers.<br><br>Technical issues. Nearly all participants reported facing minor technical issues when using the proposed watch. Common technical problems included system hangs, short battery lifespan, and slow response time from the system.	With that ability, they could feel even more motivated to pursue continuous improvement.	During the interviews, the participants were asked to suggest any changes they would like to recommend for the device. The top five changes proposed by the participants are: (a) changing to the strap, (b) reducing the size of the device, (c) more exercise variety, (d) reducing the weight of the device, and (e) resolving technical issues.All participants preferred to use the proposed wristwatch at home, which they perceived as a conducive environment because it provided them with privacy and physical space to perform their exercises. Some expressed hesitancy to wear the device outdoors, as they were concerned about others' perceptions, primarily due to its obtrusiveness and aesthetics and the lack of a suitable place.<br><br>Although the proposed device allowed such flexibility, the device's reminder system created some restrictions because the alarm would ring at the previously scheduled time set by the therapist<br><br>Some participants requested more variety of exercise content in the telerehabilitation system in order to sustain their long-term engagement.	Intention to use. When asked about their intention to use the device in the future, all participants unanimously indicated their keenness to use it. Nevertheless, to facilitate long-term usage, some participants requested an interim review with the therapist on their progress and the opportunity to adapt the prescribed exercises throughout the intervention. In their reviews with the therapist, they wanted to discuss adjusting the device's external prompts and adapting the exercises' difficulty level according to their upper limb's progress. In addition, some wished to see their progress and self-monitor it from the telerehabilitation system.	Slightly more than half of the participants did not consider the opinions of their families and friends to be significant in affecting their intention to use the proposed wristwatch.	Participants found the wristwatch useful for creating health benefits and establishing an exercise routine.Most participants reported physical improvements in their hemiplegic limbs after using the wristwatch, such as increased upper and lower limb strength and reduced upper limb pain.		This research received the grant from Caspar Health Limited (No. ZDBD). I had full access to all of the data in this study and I take complete responsibility for the integrity of the data and the accuracy of the data analysis.

Deployment of assistive living technology in a nursing home environment: methods and lessons learned	2013	BMC MEDICAL INFORMATICS AND DECISION MAKING	Aloulou, H and Mokhtari, M and Tiberghien, T and Biswas, J and Phua, C and Lin, JHK and Yap, P	10.1186/1472-6947-13-42	With an ever-growing ageing population, dementia is fast becoming the chronic disease of the 21st century. Elderly people affected with dementia progressively lose their autonomy as they encounter problems in their Activities of Daily Living (ADLs). Hence, they need supervision and assistance from their family members or professional caregivers, which can often lead to underestimated psychological and financial stress for all parties. The use of Ambient Assistive Living (AAL) technologies aims to empower people with dementia and relieve the burden of their caregivers. The aim of this paper is to present the approach we have adopted to develop and deploy a system for ambient assistive living in an operating nursing home, and evaluate its performance and usability in real conditions.	Qualitative	Singapore	dementia patients and professional caregivers	The eight patients are women living on the second floor of the nursing home and aged between 78 and 92 years. Two of them need minimal assistance (help to walk or to lie on the bed) while the six others need moderate assistance (help to take the shower or to eat).	assistive living technology in a nursing home environment					We have also met problems related to the use of sensors, being battery issues or simply a curious user pulling off the new "object" in his environment		[illegible] for them to be informed on how patients are performing their ADLs, and to provide support as and when appropriate. In order not to increase the attention needed from caregivers, it has been decided that the system should remain silent when no issues are met by patients. Notifications are to be raised only when a reaction is necessary.<br><br>In order to assist the patients in these activities, caregivers emphasised that reminders should first be sent to patients to encourage them to think and retain some level of independence. Caregivers should interfere only when the residents lose their way and are not able to solve their problems independently.<br><br>Using our system, caregivers should be able to register an interest that keep track of which services are useful for which residents. Services should also be personalized to the profile and the evolution of the disease of each patient.				Guaranteeing the respect of patients' and caregivers' privacy and the confidentiality of the research records is a major requirement.	N
Accuracy and Adoption of Wearable Technology Used by Active Citizens: A Marathon Event Field Study	2017	JMIR MHEALTH AND UHEALTH	Pobiruchin, M and Suleder, J and Zowalla, R and Wiesner, M	10.2196/mhealth.6395	Today, runners use wearable technology such as global positioning system (GPS)-enabled sport watches to track and optimize their training activities, for example, when participating in a road race event. The aim of this study was 2-fold: (1) to determine the adoption of wearable technology by runners, especially "smart" devices and (2) to investigate on the accuracy of tracked distances as recorded by such devices.	Quantitative	Germany	Active citizens	The cohort of the pre-race survey comprised 78.7% (133/169) male and 21.3% (36/169) female full marathon runners. For the half-marathon, 61.9% (396/635) males and 37.3% (239/635) females were recorded. According to the organizer's starting list, 82.4% (593/720) of the marathon runners were males and 17.6% (127/720) female.<br><br>For the half-marathon course, a higher percentage of female runners (27.01%, 1492/5524) had themselves registered (male: 72.99%, 4032/5524). Table 1 shows the distribution of sex and age for the full and half-marathon.	Wearable Technology Used by Active Citizens			Results of the pre-race survey obtained by Q1 showed that 26.1% (234/898) of the runners did not use any device for their exercises or during a running event. In contrast, 8.8% (79/898) of the athletes stated that they used more than 1 device.	Regression analysis showed that the relation between females and higher age groups and no usage of additional devices for exercise was statistically significant (Table 3).			An analysis of device records for the full and half-marathon participants revealed that, in both groups, the majority of runners preferred GPS-enabled sport watches (full: 57.5%, 104/181; half: 42.6%, 297/698). Interestingly, the usage of mobile phones in combination with running apps was more prevalent for half-marathon participants (full: 12.2%, 22/181; half: 19.5%, 136/698).					N
Living with Sensor-Augmented Pump Therapy in Type 1 Diabetes: Adolescents' and Parents' Search for Harmony	2014	CANADIAN JOURNAL OF DIABETES	Rashotte, J and Tousignant, K and Richardson, C and Fothergill-Bourbonnais, F and Nakhla, MM and Olivier, P and Lawson, ML	10.1016/j.jcjd.2014.02.002	Adolescents have difficulty successfully sustaining use of continuous glucose monitoring even when it is introduced to experienced pump users. However, little is known about how adolescents and parents perceive and manage sensor-augmented pump therapy (SAPT) in daily life. The purpose of this study was to explore adolescents' and parents' daily experience of living with SAPT.	Qualitative	Canada	Diabetes adolescents and parents	Female/male, n 4/3<br>Age, years, median (range) 15 (13–17)†<br>Diabetes duration, years, median (range) 11 (3–14)<br>Age at diabetes onset, years, median (range) 4 (1.5–10)<br>Duration of pump use, years, median (range) 4 (0–7)<br>Duration of CGM use,* months, median (range) 18 (1.5–24)<br>Introduction to CGM 3 simultaneous pump+CGM<br><br>4 delayed: pump, then CGM‡	Sensor-Augmented Pump Therapy in Type 1 Diabetes					A positive partnership with CGM technology was rarely easily developed, and unlike their partnership with the pump, no adolescent expressed that CGM felt like it was part of their being.					In due course, adolescents who experienced harmony living with CGM technology learned to use its data to become more attuned to their body's early warning signs of hypoglycemia and hyperglycemia and, for some, to identify trends that aided in peak physical performance. Similarly, they learned to use knowledge of their body to inform them when CGM readings were inaccurate. Trusting CGM technology was difficult as the readings were often inaccurate and the monitor was unreliable owing to various malfunctions.Adolescents who trusted the technology had learned to limit their expectations about the accuracy of glucose readings, appreciating that a partnership with CGM would require their time and attention in the form of recalibrations.	System mistrust resulted in frustration or anger at being inconvenienced and a sense that a commitment to SAPT was "just wasted time." The sensor did not decrease the need for self-monitoring of blood glucose; in fact, it was often required more frequently because stable glucose readings were necessary for system calibration and verification of data and alarms.	UNDECLARED
Experiences of remote mood and activity monitoring in bipolar disorder: A qualitative study	2017	EUROPEAN PSYCHIATRY	Saunders, KEA and Bilderbeck, AC and Panchal, P and Atkinson, LZ and Geddes, JR and Goodwin, GM	10.1016/j.eurpsy.2016.11.005	Mobile technology enables high frequency mood monitoring and automated passive collection of data (e. g. actigraphy) from patients more efficiently and less intrusively than has previously been possible. Such techniques are increasingly being deployed in research and clinical settings however little is known about how such approaches are experienced by patients. Here, we explored the experiences of individuals with bipolar disorder engaging in a study involving mood and activity monitoring with a range of portable and wearable technologies.	Mixed	UK	People in bipolar disorder	Mean age at study start (SEM, range), years 43.43 (3.2, 26–63) 46.29 (4.08, 33–63) 44.38 (2.49, 26–63)<br>Female gender, n (%) 9 (64.3) 5 (71.4) 14 (66.7) Employment status<br>Employed full-time, n (%) 5 (35.7) 5 (71.4) 10 (47.6)<br>Employed part-time, n (%) 5 (35.7) 2 (28.6) 7 (33.3)<br>Student, n (%) 1 (7.1) 0 (0.0) 1 (4.8)<br>Unemployed, n (%) 3 (21.4) 0 (0.0) 3 (14.3)<br>Education level<br>O-level/GCSE, n (%) 1 (7.1) 0 (0.0) 1 (4.8)<br>AS/A-level/HND/BTEC, n (%) 2 (14.3) 1 (14.3) 3 (14.3)<br>Degree (includes NVQ level 5), n (%) 7 (50.0) 3 (42.9) 10 (47.6)<br>Post-graduate degree, n (%) 3 (21.4) 3 (42.9) 6 (28.6)<br>Missing data, n (%) 1 (7.1) 0 (0.0) 1 (4.8)	remote mood and activity monitoring in bipolar disorder			A majority (n = 14, 66%) of participants spoke about the importance of context when monitoring mood.		Questionnaire responses revealed that once daily mood monitoring via the daily Mood Zoom questionnaires was found to be of little or no inconvenience (Mdn 1.5; Table 2).In addition, the majority of participants tolerated the study devices well. some participants commented on problems remembering to wear their technology or apply appropriate button-presses to activate certain functions.	They emphasised that mood changes were to be expected in many instances. In some cases, participants expressed the desire to be able to record context in order to be able to reflect on possible patterns and triggers to their mood episodes.				The majority (n = 16, 76%) of participants described how the use of mood and activity monitoring enhanced their understanding of their illness.The relationship between mood states and sleep was noted by a number of participants (n = 6, 29%).In over half of participants, mood and activity monitoring was associated with a change in behaviour. The majority of change related to activity, with most finding that they had increased their exercise levels as a result of taking part in the study.Others commented on improving their sleep hygiene.Eighteen participants (86%) thought that prospective mood and activity monitoring would be beneficial to enhance clinical decision making during routine clinical care, and make time spent with psychiatrists more efficient.Comments were made about how difficult it is to recall mood states and how monitoring can assist with this.	Four participants spoke about the potential for monitoring to become preoccupying and thus unhelpful.A few (n = 3, 14%) expressed a desire to receive feedback or interpretation of their data.Two participants raised concerns as to how they might misinterpret monitoring when unwell and many others highlighted the need for such devices to look normal and not draw attention from the general public. Concerns were raised that reactive mood changes might be misinterpreted as representing illness relapse. Three participants raised concerns about the sensitivity of the daily Mood Zoom questions having noticed that their responses rarely changed.One participant acknowledged that this might simply have reflected their current mood state.Some queried the mood descriptors we had used in the questionnaires as they felt that others may have been more relevant to them.Others (n = 6, 29%) raised semantic concerns stating that they would have preferred to have had greater explanation as to the meaning of the mood	GMG and JRG are NIHR Senior Investigators. GMG holds a grant from Wellcome Trust, shares in P1vital and has served as consultant, advisor or CME speaker for AstraZeneca, MSD, Eli Lilly, Lundbeck (/Otsuka or /Takeda), Medscape, P1Vital, Pfizer, Servier and Sunovion. The views expressed are those of the authors and not necessarily those of the NHS, the NIHR or the Department of Health. The other authors have no disclosures to make.
A Smoking Cessation Mobile App for Persons Living With HIV: Preliminary Efficacy and Feasibility Study	2022	JMIR FORMATIVE RESEARCH	Schnall, R and Liu, JF and Alvarez, G and Porras, T and Ganzhorn, S and Boerner, S and Huang, MC and Trujillo, P and Cioe, P	10.2196/28626	The prevalence of smoking in the United States general population has gradually declined to the lowest rate ever recorded; however, this has not been true for persons with HIV. We conducted a pilot test to assess the feasibility and efficacy of the Lumme Quit Smoking mobile app and smartwatch combination with sensing capabilities to improve smoking cessation in persons with HIV.	Mixed	USA	persons with HIV who smoke .	Characteristics Total (n=40)a Intervention (n=20) Control (n=20) P value<br>Age (years), mean (SD) 53.7 (9.2) 53.4 (10.2) 54.0 (8.5) .84<br>Gender identity, n (%) —b — — >.99<br>Male 20 (50) 10 (50) 10 (50) —<br>Female 18 (45) 9 (45) 9 (45) —<br>Transgender female 1 (3) 0 (0) 1 (5) —<br>Genderqueer 1 (3) 1 (5) 0 (0) —<br>Sex at birth, n (%) — — — >.99<br>Male 22 (55) 11 (55) 11 (55) —<br>Female 18 (45) 9 (45) 9 (45) —<br>Race, n (%) — — — .06<br>Black/African American 31 (78) 13 (65) 18 (90) —<br>White 1 (3) 0 (0) 1 (5) —<br>Multiracial 1 (3) 1 (5) 0 (0) —<br>Unknown 7 (18) 6 (30) 1 (5) —<br>Hispanic/Latinx ethnicity, n (%) — — — .13<br>Yes 9 (23) 7 (35) 2 (10) —<br>No 31 (78) 13 (65) 18 (90) —<br>Education, n (%) — — — .11	A Smoking Cessation Mobile App for Persons Living With HIV			Very good							"And it does really help you. It tells you when your day is over. It goes, 'You have such-and-such more days before you quit.' That was a good reminder..."<br>"I looked at it a couple times a day. In the beginning when I was smoking, I looked at it almost every time I smoked just to see if it recorded. But after I stopped smoking, I would look at it still..."<br>"...this watch is going to monitor my movement of my hand... I'm not going to be smoking all the time and moving my hand... I already knew that... that I would have to check in and be honest. Honesty was one of my biggest, like I expected to be honest. And that's what I've been..""[The app was] 100% helpful for an individual that's really focused on not smoking, on quitting, being focused on quitting and wanting to... I mean that was amazing... to have a watch on and when you're going like this or you know... they always give you the option to say, well, no, I wasn't smoking at that time; I was eating or something like that... the individual has to be honest, with themselves...		N
Development and Feasibility of a Digital Acceptance and Commitment Therapy-Based Intervention for Generalized Anxiety Disorder: Pilot Acceptability Study	2021	JMIR FORMATIVE RESEARCH	Hemmings, NR and Kawadler, JM and Whatmough, R and Ponzo, S and Rossi, A and Morelli, D and Bird, G and Plans, D	10.2196/21737	Generalized anxiety disorder (GAD) is characterized by excessive worry that is difficult to control and has high comorbidity with mood disorders including depression. Individuals experience long wait times for diagnosis and often face accessibility barriers to treatment. There is a need for a digital solution that is accessible and acceptable to those with GAD. This paper aims to describe the development of a digital intervention prototype of acceptance and commitment therapy (ACT) for GAD that sits within an existing well-being app platform, BioBase.	Mixed	UK	people with Generalized Anxiety Disorder	Variable Value<br>Age (years), mean (range) 25.2 (19-48)<br>Gender, n<br>Female 10<br>Male 0<br>Education, n (%)<br>School to age 16 2 (20)<br>College/A-levels to age 18 7 (70)<br>Undergraduate degree 1 (10)<br>Undertaking therapy/counseling, n (%)<br>Yes 2 (20)<br>No 8 (80)<br>On medication for anxiety, n (%)<br>Yes 8 (80)<br>No 2 (20)	a Digital Acceptance and Commitment Therapy–Based Intervention for Generalized Anxiety Disorder BioBase is a smartphone app that contains several features that cover the core processes of ACT (aware, open, and active).			useful		Results from the SUS indicate an average A rating (mean 83.4, median 88.7, IQR 75-96.9). All 7 participants that had an exit interview stated downloading the app and going through the app onboarding process including pairing the wearable were simple and none reported problems. Three participants stated they felt they needed to wear the wearable a bit too tight or that it was uncomfortable at times.					[illegible] helpful, and useful for their anxiety.<br><br>Participants stated the tools were also relevant, useful, and helpful to their anxiety. Three participants stated the deep breathing exercise was therapeutic, especially before bed. Two participants specifically found the mood tracker helpful for their anxiety, stating "it made me stop and think a bit more about how I'm feeling." One participant mentioned the body scan as the most useful tool as she preferred body awareness mindfulness techniques over breathing-related relaxation exercises.<br><br>Overall, 2 participants stated that by "using an app every day to check in with myself, it's much easier to control my anxiety" and that "having goals set by the app and a module to complete each day was helping my mental state ... makes me look at [a feeling], acknowledge it and move on from it easier."		DP and DM were the CEO and CTO, respectively, and cofounders of BioBeats, the provider of the BioBase program. Both DP and DM contributed to interpretation of the data and revising the manuscript, but were not involved in study design, recruitment, analysis, or initial drafting of the manuscript. SP, NH, JK, and GB were employees of BioBeats. The analysis was conducted by NH, JK, and SP. Manuscript was drafted by NH and JK. GB contributed to revision of the manuscript and interpretation of data. RW was a paid consultant for BioBeats, the provider of the BioBase program, and contributed to program design and revision of the manuscript.
Use of wireless respiratory rate sensor monitoring during opioid patient-controlled analgesia after gynaecological surgery: A prospective cohort study	2021	INDIAN JOURNAL OF ANAESTHESIA	Cheng, SM and Chan, JJI and Tan, CW and Lu, EH and Sultana, R and Sng, BL	10.4103/ija.IJA_1262_20	Respiratory depression is a rare but serious complication during opioid administration. Therefore, early detection of signs of deterioration is paramount. The current standard of care of using manual intermittent respiratory rate (RR) measurement is labour intensive and inefficient. We evaluated a wireless sensor monitor, Aingeal (Renew Health Ltd, Ireland), to continuously monitor RR, heart rate (HR) and temperature compared to standard clinical measurements.	Quantitative	Singapore	Female patients undergoing gynaecological surgery and requiring postoperative analgesia via intravenous PCA	Age (years) 46.7±9.0<br>Race<br>Chinese 24 (68.6)<br>Malay 8 (22.9)<br>Indian 0 (0.0)<br>Others 3 (8.5)<br>Weight (kg) 69.0±16.8<br>Height (cm) 156.5±7.0<br>BMI (kg/m2) 28.1±6.3<br>ASA status<br>I 12 (34.3)<br>II 19 (54.3)<br>III 4 (11.4)	wireless respiratory rate sensor monitoring during opioid patient-controlled analgesia after gynaecological surgery					Patients expressed high satisfaction regarding the use of sensor device, although a few patients reported discomfort or skin itchiness upon application [Table 2].Some of them also had difficulties with self-application especially after showering.							N
A Fitness App for Monitoring Walking Behavior and Perception (Runkeeper): Mixed Methods Pilot Study	2021	JMIR FORMATIVE RESEARCH	Hollander, JB and Folta, SC and Graves, EM and Allen, JD and Situ, M	10.2196/22571	Physical activity has a strong positive impact on both physical and mental health, and public health interventions often encourage walking as a means to promote physical activity. Social connectivity, such as that among spouses, families, friends, and colleagues, highly influences physical activity. Although technology-based interventions have some influence on human behavior, they have not been fully implemented and evaluated for their influence on walking through social connectivity. We aimed to pilot-test the organization of neighborhood walking clubs and use of a mobile app (Runkeeper) to encourage social connectedness and neighborhood cohesion, as well as to increase physical activity.	Mixed	USA	General public	In total, we recruited 46 participants: 34 from the Facebook advertisement, 5 from door-to-door recruitment, and 7 from friends' recommendations. A total of 7 were identified as males and 39 as females (Multimedia Appendix 1). The participation rate of the baseline survey was 100% (43/43), and the completion rate of the exit survey was 100% (43/43	A Fitness App for Monitoring Walking Behavior and Perception (Runkeeper)					Although many participants appreciated the tracking features of the app, most were frustrated by the manual operations of it.Some said they preferred wearable PA monitors that did not require this.	[illegible] suggests that participants were motivated by the competitive aspect of the walk ranking on the app. Participants also reported that being organized in groups to walk enhanced feelings of accountability, competitiveness, and peer pressure.A theme from the interviews supports and provides context for the change in psychological condition as a form of motivation. Many considered walking to be the best way of releasing stress, especially after their daily work.Themes from the interviews further suggest that participants were avid walkers but not avid app users. Many said that they walked more frequently than suggested by the app and often forgot to manually start it.		nonadherent participants described more barriers to PA, such as work and family issues.		participants mostly expressed that walking enhanced their appreciation for the physical and natural features of their neighborhood.<br><br>Some participants also felt more virtually connected with their neighbors because of the chat platform on the app.<br><br>These participants also revealed a lack of belongingness to their physically existing community.		N
Formative Evaluation of a Smartphone App for Monitoring Daily Meal Distribution and Food Selection in Adolescents: Acceptability and Usability Study	2020	JMIR MHEALTH AND UHEALTH	Langlet, B and Maramis, C and Diou, C and Maglaveras, N and Fagerberg, P and Heimeier, R and Lekka, I and Delopoulos, A and Ioakimidis, I	10.2196/14778	Obesity interventions face the problem of weight regain after treatment as a result of low compliance. Mobile health (mHealth) technologies could potentially increase compliance and aid both health care providers and patients.This study aimed to evaluate the acceptability and usability and define system constraints of an mHealth system used to monitor dietary habits of adolescents in real life, as a first step in the development of a self-monitoring and lifestyle management system against adolescent obesity.	Quantitative	Sweden	Adolescents	a mean age of 16.8 years (SD 0.7 years) and BMI of 21.9 kg/m2 (SD 4.1 kg/m2). Characteristics Total (n=24), mean (SD) Men (n=7), mean (SD) Women (n=17), mean (SD)<br>Age (years) 16.8 (0.7) 17.2 (0.5) 16.6 (0.7)<br>Weight (kg) 62.2 (14.4) 75.7 (10.1) 56.6 (12.1)<br>Height (cm) 168.1 (10.3) 181.3 (6.8) 162.7 (5.3)<br>BMI (kg/m2) 21.9 (4.1) 23.3 (4.7) 21.3 (3.8)	Formative Evaluation of a Smartphone App for Monitoring Daily Meal Distribution and Food Selection in Adolescents					Participants rated the system usability between 47.5 and 97.5, with a mean value of 77.1, which corresponds to a grade B 'Good' rating, on the SUS. No adverse events were reported to researchers during the experiments or to school personnel after the experiment. The smartphone app and chewing sensor received the highest and lowest ratings for comfort, respectively. For perceived usability, all system components (smartphone app, food scale, and chewing sensor) scored highest at home and lowest outside home (Table 2).		Suggestions for improvements of the smartphone app fell into 4 categories: (1) 5 individuals suggested making it more responsive or faster, (2) 4 wanted a brighter color theme, (3) 3 wanted the function to retrospectively add meals, and (4) 3 requested better app interfaces, without specifying what was lacking.					During data collection, BL and II received part of their salaries from Mandometer AB (Stockholm, Sweden), owner of the IPR for the Mandometer®. This does not alter the authors' adherence to journal policies
Smart Patch for Skin Temperature: Preliminary Study to Evaluate Psychometrics and Feasibility	2021	SENSORS	Kim, H and Kim, S and Lee, M and Rhee, Y and Lee, S and Jeong, YR and Kang, S and Naqi, M and Hong, S	10.3390/s21051855	There is a need for continuous, non-invasive monitoring of biological data to assess health and wellbeing. Currently, many types of smart patches have been developed to continuously monitor body temperature, but few trials have been completed to evaluate psychometrics and feasibility for human subjects in real-life scenarios. The aim of this feasibility study was to evaluate the reliability, validity and usability of a smart patch measuring body temperature in healthy adults.	Quantitative	Korea	General public	The average age of the 35 participants was 30.29 (SD = 9.63) years; they were mostly female (20/35, 57.1%), had a bachelor's or higher degree (23/35, 65.7%) and most were college students (9/35, 25.7%) or graduate students (4/35, 11.4%). Six participants (dropout rate = 17.14%) were excluded due to incomplete data secondary to: (1) device errors of data storage or (2) partially missing information in spite of 24-h attachment of the smart patch.	Smart Patch for Skin Temperature					[illegible] (M = 3.37, SD = 0.81) and ease to maintain (M = 3.49, SD = 0.74).<br><br>Few participants reported some discomfort due to limited daily activity (n = 2), itchy skin (n = 7) or detaching concerns (n = 2). In some situations, especially when exercise was prolonged, high-intensity and in a hot environment, extra sweat led to increased showering. We provided protective film to the participant to prevent frequent showers from damaging the smart patch and falling off the participant's body. Participants attached the protective film to the smart patch during the shower and removed it after the shower. However, smart patches also fell off in the process of removing protective films, and, frequent hot water showers have damaged the appearance of the smart patches. Other problems include itching of the attachment site due to skin dryness and discomfort caused by the smart patch when moving during sleep (see Appendix B).					They mentioned satisfaction of "being self-monitored" that the study participants were able to monitor their own body temperature using the supplied mobile app. The study participants perceived how their daily activities and surrounding environment affect the body temperature changes. Thus, they stated that they had some insight to maintain the normal body temperature for the health and safety.		The authors declare no conflict of interest. The funders had no role in the design of the study; in the collection, analyses, or interpretation of data; in the writing of the manuscript, or in the decision to publish the results.
USER REQUIREMENTS AND ANALYSIS OF PREECLAMPSIA DETECTION DONE THROUGH A SMART BRACELET	2018	12TH INTERNATIONAL TECHNOLOGY, EDUCATION AND DEVELOPMENT CONFERENCE (INTED)	Marin, I and Vasilateanu, A and Pavaloiu, B and Goga, N		This paper is based on a survey completed by persons of different ages and having various specialization domains regarding the use of smart bracelets for detecting preeclampsia. The aim is to decide upon its popularity among people and to determine the user requirements.	Quantitative	Unspecified	preeclampsia patients	Out of the 100 participants, 64 were women and 36 were men, all having different ages, occupations and coming from different countries. Their average age is of 28 years. 71% of the interviewed persons own or intend to buy a smart watch or a smart bracelet. More than half of them already use a health care application on their smart device.	PREECLAMPSIA DETECTION DONE THROUGH A SMART BRACELET	89% of them said that they do not know any wearable device which monitors preeclampsia.		89% of the participants attested the fact that they would like the pregnant women to be monitored during gestation to determine the occurrence of preeclampsia. An encouraging number of 90.63% participants are willing to have their blood pressure monitored during the pregnancy. 88.89% of them would recommend such a product to a relative or a friend, proving that such a product is welcomed and appreciated on the market.				The most wanted features which the participants want are health statistics (71%), along with heart rate monitor (71%), followed by sports track (41%), message reminder (33%) and call reminder (24%). women, having an average age of 29 years. The features that they would like the bracelet to have are heart rate monitor (75%), health statistics (75%), followed by sports track (39.06%), message reminder (32.81%) and call reminder (20.31%).					UNDECLARED
Patient and practitioner experience with clinical lumbar motion monitor wearable technology	2019	HEALTH AND TECHNOLOGY	Ferguson, SA and Berner, RS and Bridger, MA and Khan, SN and Weaver, TE and Yu, EM and Marras, WS	10.1007/s12553-019-00330-7	Low back pain (LBP) is the leading cause of disability worldwide. Unfortunately, there is no gold standard for objectively quantifying low back function. The clinical lumbar motion monitor (CLMM), a wearable technology, has been developed to provide an objective measure of low back function. The evaluation for the patient is like playing a video game with their back. For health care practitioners the CLMM provides three metrics including overall impairment, structural/muscular and test reliability (do we have good data). This study had two goals. 1) To evaluate the ease of use for the patients. 2) To evaluate how the health care practitioners were able to use the results.	Quantitative	USA	the low back pain patients	Sixty-eight patients participated in the research. The average age of participants was 44 years with a standard deviation of 11 years. Fifty-four percent of the population was male. However, two patients left the clinic before completing their questionnaire.	clinical lumbar motion monitor wearable technology											The biggest concern for further development may be the comfort of the monitor with 8% responding neutral.	N
Patients' attitudes toward the use of IoT medical devices: empirical evidence from Romania	2019	PROCEEDINGS OF THE INTERNATIONAL CONFERENCE ON BUSINESS EXCELLENCE	Costea-Marcu, IC and Militaru, G	10.2478/picbe-2019-0050	The Internet of Things (IoT) in healthcare sector is the collection of medical devices and applications that connect through online computer networks. The purpose of this study is to analyse the acceptance of medical devices that are based on IoT technology and monitor the health of individuals/patients.	Quantitative	Romania	patient		IoT medical devices										For the elderly and people with disabilities, these IoT provisions are veryuseful in providing autonomy, a personalized approach and permanent monitoring.	face the adoption of the IoT.In addition, privacy requirements are another major challenge in using these technologies. Risks arisewhere devices and applications are untested, resulting in issues with interoperability, stability,compatibility and data security.	UNDECLARED

Feasibility of Using a Wearable Biosensor Device in Patients at Risk for Alzheimer's Disease Dementia	2020	JPAD-JOURNAL OF PREVENTION OF ALZHEIMERS DISEASE	Saif, N and Yan, P and Niotis, K and Scheyer, O and Rahman, A and Berkowitz, M and Krikorian, R and Hristov, H and Sadek, G and Bellara, S and Isaacson, RS	10.14283/jpad.2019.39	Alzheimer's disease (AD) is the most common and most costly chronic neurodegenerative disease globally. The goal of this prospective observational study was to determine the feasibility of using a wrist-worn biosensor device in patients at-risk for AD dementia.	Quantitative	USA	Patients at Risk for Alzheimer's Disease Dementia	Forty consecutive APC patients were asked to participate in the study. Thirty-four patients (85%) agreed to wear the WHOOP device. However, one patient (2.5%) lost the device prior to data collection. All 33 patients had a family history of AD and were given an extensive clinical evaluation which included neuropsychological testing, anthropometric measurements, serum biomarkers, and AD biomarker assessment (via amyloid positron emission tomography [PET] or cerebrospinal fluid). Upon clinical assessment, 24 patients (72.7%) were classified as normal cognition and were asymptomatic, 6 patients (18.2%) had subjective cognitive decline (SCD), and 3 patients (9.1%) were found to be amyloid-positive (one with pre-clinical AD, one with pre-clinical Lewy-Body Dementia [LBD], and one diagnosed with MCI due to AD). Five patients took prescription medications for sleep	a Wearable Biosensor Device in Patients at Risk for Alzheimer's Disease Dementia			Of these 27 participants, 22 (81.5%) were satisfied with WHOOP after one month and 24 (88.9%) were satisfied after six months (satisfied = Strongly Agree + Agree). At six months, 23 participants (85.2%) responded that they wanted to continue wearing the WHOOP while 2 participants (8.4%) responded that they were not sure.									Dr. Isaacson has served as a scientific advisor for Eisai. The remaining authors report no conflicts of interest or other relevant disclosures.
Investigating Adoption Factors of Wearable Technology in Health and Fitness	2018	2018 OPEN INNOVATIONS CONFERENCE (OI)	Rubin, A and Ophoff, J		This research study uses the second generation of the Unified Theory of Acceptance and Use of Technology (UTAUT2) model to investigate the factors of adoption which would influence an individual's behavioural intention towards using a wearable device, focusing on health and fitness purposes.	Quantitative	USA	General public	The results show that most of the data was collected from younger individuals as the age category of 18–24 had the highest responses with 40.32%. A large portion of the data collected came from the middle-aged working-class age group categories (i.e. 30–49) with a total of 34.68%. The lowest number of participants came from the over 60 age group category with 2.42%. The remaining 22.58% of participants came from the 25–29 age group category (9.68%) and the 50–59 age group category (12.9%).Gender consisted of three categories; “Male”, “Female” and “Prefer Not to Answer”. Most of the participants (58.06%) consisted of females while the remaining participants were male as there were no participants who did not wish to withhold their gender. The highest income level category was “More than R50 000” with 22.58%. This	Wearable Technology in Health and Fitness						The most noteworthy outcome from the significance test is that “Effort Expectancy”, “Social Influence”, “Facilitating Conditions”, “Hedonic Motivation” and “Price” are not considered to have a positive relationship with “Behavioural Intention” as hypothesised. The second most noteworthy outcome from the significance test is that neither “Facilitating Conditions” nor “Habit” had a positive relationship with “Usage Behaviour”						UNDECLARED
Experiences of Using Wearable Continuous Glucose Monitors in Adults With Diabetes: A Qualitative Descriptive Study	2022	SCIENCE OF DIABETES SELF-MANAGEMENT AND CARE	Kang, HS and Park, HR and Kim, CJ and Singh-Carlson, S	10.1177/26350106221116899	The purpose of the study was to understand the experiences of adults with diabetes wearing a continuous glucose monitor (CGM).	Qualitative	Korea	Diabetes adults	The participants ranged in age from 22 to 65 years (M = 44.5 years, SD = 13.67), with 21.5% being under age 30, 21.05% 30 to 39 years, 10.53% 40 to 49 years, 36.84% 50 to 59 years, and 10.53% over age 60. Twelve (63.2%) were women, and 7 (36.8%) were men. Eleven participants had T1DM (57.9%), and 42.1% had T2DM. Regarding the length of time using a CGM, 85.5% of participants reported using it for 1 month to 1 year. Most participants (84.2%) used insulin.	Wearable Continuous Glucose Monitors in Adults With Diabetes	Most participants stated that they were unaware of continuous glucose monitoring until their clinicians recommended it. Thus, they indicated that it was helpful when the clinician explained in detail their status, such as “it is within normal range,” “it is much higher right now, so it is better to increase the insulin dose.”				Participants responded that their first impression of the CGM was that it was an “easy to use” and “convenient” device. Moreover, visualizing their glucose status was easier through graphs, numbers (eg, glucose, A1C), and color-coding with red, green, or orange, which indicated low, normal, or high glucose levels, respectively, compared with written glucose record sheets.		Regarding the need for improved service quality, participants responded that they hoped services would improve and better insurance coverage would be provided. Several participants contacted the service center to exchange their devices for errors or detachment issues. Some said they were satisfied with the service, while others wished they could exchange sensors more efficiently from where they were purchased. Participants noted that it was simple to buy the device, but the exchange process was difficult because they could not exchange them at the site where it was purchased or at the pharmacy. In addition, participants had difficulty confirming whether the device could be exchanged by contacting someone in the service department. One participant said, “It was not easy to communicate through the computer and send photos of the sensor when problems such as a malfunction occurred.”			Participants reported that glucose levels using CGM could be checked quickly when they experienced physical symptoms, such as shakiness or lethargy, to determine if they had hypoglycemia. They were motivated by this feedback and empowered to engage in self-care. Moreover, one change that participants noticed was the clinician's use of their glucose data during an appointment. They reported feeling that they were receiving better care than before and received more accurate treatment because clinicians could carefully check and explain the data in greater detail based on the graphs and blood glucose patterns. Participants responded that their quality of life improved since using the CGM. Most importantly, participants did not have to complete fingerstick tests before or after meals, which they found stressful. They described that their social and physical activities were less restricted and more accessible than before, and participants felt freer than before continuous glucose monitoring. They could also enjoy sports that they were previously afraid to participate in because of their fears of hypoglycemia. Participants responded that their glucose and A1C levels decreased. They liked	Common issues mentioned by participants were the device becoming loose, the sensor detaching, and differences in glucose levels between the fingersticks and the CGM. Participants also reported that the area around the sensor was itchy, insertion site pain, minor skin discoloration at the adhesive sites, and difficulty placing the sensor correctly. Participants said that the sensor became loose when they showered or bumped the corner of a wall, door, or refrigerator while working or walking. Participants said they felt disappointed when the CGM detached because of its cost. Participants stated that the adhesive tape should have been present in the package rather than having to purchase them separately, the size of the sensor should have been smaller, or that the sensor could have been placed in areas other than the upper arms, such as the abdomen or thigh, to reduce the risk of detachment and to make it	N
Better understanding about what's going on': young Australians' use of digital technologies for health and fitness	2020	SPORT EDUCATION AND SOCIETY	Lupton, D	10.1080/13573322.2018.1555661	Digital technologies such as websites, online discussion forums, social media, content-sharing platforms, mobile apps and wearable devices are now available as a means for young people to learn about and promote their health, physical fitness and wellbeing. This article provides findings from a qualitative interview-based study of young Australians (aged 16-25 years) which explored their practices and understandings related to digital and non-digital sources of health and fitness information, advice and support.	Qualitative	Australia	young people	Seventeen participants were aged 16–20 years and 13 were aged 21–25 years. Most of the participants (22) had completed at least some university education, while five were still at school and three had finished or were currently in post-school technical training. In terms of ethnic background, this was quite a diverse group of young Australians, reflecting the multi-ethnic nature of the Australian population more generally (Australian Bureau of Statistics, Citation2018). Almost half (14) the participants identified as Anglo-Celtic, eight as Asian, two as European and six as other (Anglo-Indigenous, Middle-Eastern, Anglo/Armenian, Asian/Pacific-Islander and two as Eurasian). Ten of the participants lived in New South Wales, ten in Victoria, five in Queensland, two in South Australia and one each in Western Australia, Tasmania and the Australian Capital Territory. Twenty-one participants lived in cities	digital technologies for health and fitness					While the affordances of convenience, accessibility and detail and diversity of information offered by digital media and devices were valued by the participants, their accounts also highlighted the importance of face-to-face as well as online relationships and personal connections with other people for providing information and support, including family members and friends as well as medical professionals.					The agential capacities generated by the participants' enactments of digital health included gaining a better knowledge of bodies, illness and healthcare and feeling more in control of health and wellbeing states. The participants highly valued the agential capacity of digital technologies to generate detailed information about their bodies and health states and imagined new technologies that would be able to achieve even more detailed personalisation and customisation.	However, they expressed little knowledge or concern about how their personal health data may be exploited by other actors or agencies.	N
Do Consumers Care About Aesthetics and Compatibility? The Intention to Use Wearable Devices in Health Care	2021	SAGE OPEN	Jeong, J and Kim, Y and Roh, T	10.1177/21582440211040070	This study examined the influencing factors on consumers' intention to use wearable devices in health care (WDH).	Quantitative	Korea	General public	Among the total 303 respondents, 163 (53.8%) were female and 140 (46.2%) were male. Most respondents were in their 20s (n = 251, 82.84%), 30s (n = 46, 5.18%), 40s (n = 4, 1.32%), and 50s (n = 2, 0.66%). Education degree of most participants were high school (n = 156, 51.49%) and undergraduate students (n = 101, 33.33 %) and some participants had higher education level over graduate (n = 46, 15.18%).	Wearable Devices in Health Care				Results show no difference between females and males in all paths when they intend to use WDH.		The reason to purchase indicated that convenience showed the highest percentage (n = 192, 63.37%) and followed by brand image (n= 51, 16.83%), commercial (n = 37, 12.21%), and friends' influence (n = 23, 7.59%). Regarding the purpose of purchase, the curiosity and functional advantage marked the same highest responses (n = 91, 30.03%), followed by brand image (n = 67, 22.11%), and friends (n = 54, 17.82%).						N
Health Literacy and Health Information Technology Adoption: The Potential for a New Digital Divide	2016	JOURNAL OF MEDICAL INTERNET RESEARCH	Mackert, M and Mabry-Flynn, A and Champlin, S and Donovan, EE and Pounders, K	10.2196/jmir.6349	Approximately one-half of American adults exhibit low health literacy and thus struggle to find and use health information. Low health literacy is associated with negative outcomes including overall poorer health. Health information technology (HIT) makes health information available directly to patients through electronic tools including patient portals, wearable technology, and mobile apps. The direct availability of this information to patients, however, may be complicated by misunderstanding of HIT privacy and information sharing. The purpose of this study was to determine whether health literacy is associated with patients' use of four types of HIT tools: fitness and nutrition apps, activity trackers, and patient portals. Additionally, we sought to explore whether health literacy is associated with patients' perceived ease of use and usefulness of these HIT tools, as well as patients' perceptions of privacy offered by HIT tools and trust in government, media, technology companies, and health care.	Quantitative	USA	General public	Characteristic Mean (SD) or n (%) Age in years, mean (SD) 16.7 (43.5) Work in health care, n (%) 603 (12.1) Male, n (%) 2102 (42.3) Race, n (%) White 3146 (63.2) Hispanic 671 (13.5) African American 794 (16.0) Asian 218 (4.4) Other 121 (2.4) 2-Year college degree or higher, n (%) 2980 (59.9) Household income in US $, n (%) <10,000 230 (4.6) $10,000–49,999 1908 (38.3) $50,000–99,000 1764 (35.5) ≥$100,000 1068 (21.5)	Health information technology (HIT) makes health information available directly to patients through electronic tools including patient portals, wearable technology, and mobile apps.				Across all HIT tools, fewer participants with less than adequate health literacy indicated technology use than those with adequate health literacy	Of most relevance to our study, NVS score was significantly associated with perceived ease of use and perceived usefulness across all HIT after controlling for demographics						health literacy might influence perceptions of privacy associated with HIT.	N
Socio-technical barriers affecting large-scale deployment of AI-enabled wearable medical devices among the ageing population in China	2021	TECHNOLOGICAL FORECASTING AND SOCIAL CHANGE	Xing, F and Peng, GC and Zhang, BQ and Li, SY and Liang, XT	10.1016/j.techfore.2021.120609	In order to provide better health and caring services to its rapidly growing ageing population, China has turned to new digital innovations empowered by Internet of Things (IoT) and artificial intelligence (AI) technologies, e.g. wearable medical devices (WMDs). This paper reports on an exploratory study that investigated socio-technical barriers and challenges affecting large-scale deployment of AI-enabled WMDs amongst Chinese old people.	Qualitative	China	older adults	plus 2-3 family members were involved in each of the five focus groups with Chinese families. For each of the two WMD providers, 2-3 senior/middle managers plus 2 engineers participated in the study. The last two focus groups involved 2 managerial staff and 2-3 clinicians/caregivers from each public health organisation.	AIenabled wearable medical devices among the ageing population in China.								The results showed a set of 16 crucial barriers related to diverse technological, managerial, clinical, financial, legal and personal aspects. amongst these, lack of collaboration between WMD providers and public health organizations was identified to be the most critical barrier. This challenge was triggered by a range of clinical, financial, legal, managerial, and technical reasons, and would substantially prevent large-scale deployment and usage of AI-enabled wearable medical devices in the Chinese context.				UNDECLARED
Technology Adoption, Motivational Aspects, and Privacy Concerns of Wearables in the German Running Community: Field Study	2018	JMIR MHEALTH AND UHEALTH	Wiesner, M and Zowalla, R and Suleder, J and Westers, M and Pobiruchin, M	10.2196/mhealth.9623	Despite the availability of a great variety of consumer-oriented wearable devices, perceived usefulness, user satisfaction, and privacy concerns have not been fully investigated in the field of wearable applications. It is not clear why healthy, active citizens equip themselves with wearable technology for running activities, and what privacy and data sharing features might influence their individual decisions.The primary aim of the study was to shed light on motivational and privacy aspects of wearable technology used by healthy, active citizens. A secondary aim was to reevaluate smart technology adoption within the running community in Germany in 2017 and to compare it with the results of other studies and our own study from 2016.	Quantitative	Germany	Runner/ active citizen	A total of 845 questionnaires were collected. Use of technology for activity monitoring during events or training was prevalent (73.0%, 617/845) in this group. Male long-distance runners and runners in younger age groups (30-39 years: odds ratio [OR] 2.357, 95% CI 1.378-4.115; 40-49 years: OR 1.485, 95% CI 0.920-2.403) were more likely to use tracking devices, with ages 16 to 29 years as the reference group (OR 1).	Wearables				devices was associated with runners of younger age groups, see Table 6. This finding was statistically significant for 30 to 39 years age group (P=.002). Older age groups were less likely to use such devices. However, this finding was only significant for the age group of 60 to 69 years (P=.005). Being a participant of the walking/Nordic walking course was predictive of using no technology when compared with the reference group of half-marathon runners (P=.005). Marathon and relay runners were more likely to use wearable devices (odds ratio [OR] 1.368 and OR 1.458). Moreover, female runners seemed not to rely on technology (OR 0.745), although these findings were not statistically		As presented in Table 3, nearly 9 out of 10 runners (89.8%, 554/617) used wearables as a tool for exercise control. For at least a third of the participants (34.0%, 210/617), technology was used for self-motivational reasons and as an enabler to get more active in general. Only a very small percentage (1.0%, 6/617) of the runners mentioned that they used a wearable device sponsored by their health insurance company/sickness fund or as recommended by their physician. The most common reason for technology use was to monitor exercise. This was consistent for runners across all age groups: 87.3% to 100%		More than two-thirds (68.0%, 155/228) answered that they listen to their own body' instead of technology. Technical barriers with wearables were reported by 12.7% (29/228); and 3.0% (7/228) had encountered bad experiences in the past.				N
Acceptance and Preferences of Using Ambient Sensor-Based Lifelogging Technologies in Home Environments	2021	SENSORS	Offermann, J and Wilkowska, W and Poli, A and Spinsante, S and Ziefle, M	10.3390/s21248297	Diverse sensor-based technologies can be used to track (older and frail) people's movements and behaviors in order to detect anomalies and emergencies. The present study analyzed the perception of benefits and barriers as well as acceptance of these specific sensor-based technologies using an online survey (reaching N=312 German participants).	Quantitative	Germany	(older and frail) people	On average, the participants were rather young (M = 32.9; SD = 18.7; Min = 18; Max = 91; median = 23) and the sample contained a higher proportion of females (62.8%; n = 196). compared to males (36.9%;n=115), while one participant indicated a diverse gender (0.3%). The sample was educated comparably high with 55.8% (n=174) of the participants holding a university entrance qualification and 28.2% (n=88) holding a university degree. Only small parts of the participants reported lower educational levels, such as secondary or elementary school certificates(16.0%;n=50). Asked for their living situation, most of the participants indicated to live in a partnership or be married (56.4%;n=176), while a third (33.3%;n=104) reported to be single, and small parts to be divorced (1.9%;n=6) or widowed (4.2%;n=13). 4.2% (n=13) did not indicate their living situation. Addressing the context of support in	Ambient Sensor-Based Lifelogging Technologies in Home Environments			AI find the use of such sensors useful. * 1 6 4.2 1.2 4.3 1.2 4.3 1.2 I would like to use such sensors in my home. ** 1 6 3.3 1.4 3.5 1.4 3.5 1.4 I can well imagine the use of the sensors. *The results show technology-related differences, especially regarding the perception of benefits. Furthermore, the participants estimated the costs of these sensors to be higher than they are, but at the same time showed a relatively high willingness to pay for the implementation of sensor-based technologies in their home environment.							Recognizing deviations from normal behavior Triggering alarms (e.g., when objects are not closed properly, in emergency situations) Automatic reminders (e.g., to ventilate, close doors) Increased security (e.g., forgotten pot on the stove) Recognition of changes in movement Recognition of emergency situations Recognition of emergencies (e.g., falls) **	Feeling of dependence Perceived violation of own privacy Need for a permanent receiver (=collector) Perceived harmfulness of signals to health Doubts about reliability Unintentionally triggered alarms Unintentional damage/defects of the sensor (e.g., by dropping). Fears of emissions (e.g., noise, light, radio) Feeling of surveillance **	N
Consumers' perceived attitudes to wearable devices in health monitoring in China: A survey study	2017	COMPUTER METHODS AND PROGRAMS IN BIOMEDICINE	Wen, D and Zhang, X and Lei, J	10.1016/j.cmpb.2016.12.009	Capital market and consumer market interest in wearable devices has surged in recent years, however, their actual acceptance in the field of health monitoring is somewhat not as expected. This study aims to understand the perceptions of wearable devices of general consumers, analyze the review of the devices by users, and find existing problems associated with current wearable devices.	Quantitative	China	General public	Item Choices Frequency Percentage Sex Male 994 48.3% Female 1064 51.7% Age <18 15 0.7% 18–25 593 28.8% 26–30 447 21.7% 31–40 584 28.4% 41–50 355 17.2% 51–60 57 2.8% >60 7 0.3% Education Junior high school and below 25 1.2% Vocational and high school 117 5.7% Junior college 311 15.1% Undergraduate 1183 57.5% Master's degree 340 16.5% PhD 82 4.0% Occupation IT 328 15.94% Healthcare 289 14.04% Manufacturing 257 12.49% Education 255 12.39%Financial 151 7.34%Commerce and sales 107 5.20%Others 671 32.60% Monthly income <1000 226 11.0% 1000–2000 143 6.9% 2001–4000 475 23.1% 4001–7000 647 31.4% >7000 567 27.6%	wearable devices in health monitoring	The respondents had a low level of knowledge about wearable devices		were optimistic with regard to the devices' future (3.86/5), and 84% recognized an acceptable price of less than 2000 RMB. Nearly half of the respondents were unwilling to continuously wear the device (47.1%) and share their health data (44.7%).		The respondents had different levels of emphasis regarding the existing problems of wearable devices at different use stages. Being easily damaged or lost (49.7%), being incapable of providing health recommendations based on data analysis (46.7%), and being uncomfortable to wear (45.8%) likely lead consumers to abandon the use of wearable devices.		The functions of wearable devices that the respondents expected were mainly health management (63.5%), mobile phone accessories (61.9%), and location tracking (61.2%), and the promising hot future functions were mainly data analysis (74.2%), exercise coaching (60.5%), and child tracking (58.8%). Regarding the health monitoring functions, the respondents were most interested in heart health monitoring. (4)					N
Physiolytics at the workplace: Affordances and constraints of wearables use from an employee's perspective	2019	INFORMATION SYSTEMS JOURNAL	Mettler, T and Wulf, J	10.1111/isj.12205	Wearables paired with data analytics and machine learning algorithms that measure physiological (and other) parameters are slowly finding their way into our workplace.Using an affordance theory lens, we, therefore, explore the mental models of employees who are faced with the introduction of physiolytics as part of corporate	Quantitative	Swtherland	Employee		wearables						We identify five distinct user types eachof which characterizes a specific viewpoint on physiolytics atthe workplace: the freedom loving, the individualist, the cynical,the tech independent, and the balancer						UNDECLARED
Development of a Wearable Cardiac Monitoring System for Behavioral Neurocardiac		JMIR MHEALTH AND	Uddin, AA and Morita, PP and Tallevi, K and Armour, K and Li, J and	10.2196/mhealth.52	Elevated blood pressure is one of the main risk factors for death globally. Behavioral neurocardiac training (BNT) is a complementary approach to blood pressure and stress management that is intended to exercise the autonomic reflexes, improve stress recovery, and lower blood pressure. BNT involves cognitive-behavioral therapy with a paced breathing technique and heart rate variability biofeedback. BNT is limited to in-clinic delivery and faces an accessibility barrier because of the need for clinical oversight and the use of complex monitoring tools. The objective of this project was to design, develop, and evaluate a wearable electrocardiographic (ECG) sensor system for the delivery of BNT in a home setting.			people with Elevated blood	A total of 6 participants completed the usability test with the Beatsensor system. There were 2 male and 4 female participants. The study had 1 participant in the 25-34 years age range, 2 in the 45-54 years range, 2 in the 55-64 years range, and 1 on the 65-74 years range. Participants' mobile phone use varied from no use at all (2/6) to using a mobile phone more than once a week (3/6), with the majority (4/6) finding mobile phones	a Wearable Cardiac Monitoring System for Behavioral			Participants also indicated that they would not hesitate to wear the sensor		positive. Participants found the sensor to be unobtrusive, reporting that it was compact and small and comfortable to wear and that it did not negatively affect day-to-day tasks. However, a few participants indicated that the sensor might attract attention.The testing identified no major issues with the Beatsensor, only minor issues that can easily be mitigated with simple adjustments to the design or training. Some of the participants were unsure what orientation to wear the sensor and where to place it. Participants also found it difficult to reset or power cycle the sensor. Finally, participants experienced some discomfort removing the sensor due to the adhesive electrodes pulling on hair. The primary issue identified with the BNT app was an inadequate level of training and education on core concepts of BNT. As a result of poor organization and presentation of information in these sections, users demonstrated a lack of understanding of those concepts. This resulted in several participants performing the exercises but being unable to fully interpret the results. Thus, they could not benefit from operant feedback and were sometimes unsure if they met their goal. Another issue was information overload on certain pages. This was a result of the user being asked to pace their breathing, view the biofeedback,					In the postsession debrief interview conducted by the study coordinator, all participants stated that the system had a positive effect on their stress		

Research on elderly users' intentions to accept wearable devices based on the improved UTAUT model	2023	FRONTIERS IN PUBLIC HEALTH	Chen, JX and Wang, T and Fang, ZY and Wang, HT	10.3389/fpubh.2022.1035398	As the proportion of the world's elderly population continues to increase, wearable devices can provide ideas for solving a series of problems caused by population aging. Therefore, it is of great significance for the development of intelligent elderly care and the improvement of the quality of elderly care services to explore the factors that influence the intention of elderly users to accept wearable devices.An improved unified theory of acceptance and use of technology (UTAUT) model is constructed from the perspective of elderly individuals, and new parameters are added, including four factors related to wearable devices, including performance expectancy, perceived cost, hedonic value and aesthetic appeal, and three factors related to elderly individuals, including personal physiological conditions, health anxiety and personal innovativeness in	Qualitative	China	Older adults	The respondents were all over 60 years old.	wearable devices						The findings of this study revealed that performance expectancy, perceived cost, hedonic value and aesthetic appeal all have significant impact on elderly users' intention to use wearable devices. Furthermore, personal innovativeness in information technology, personal physiological condition, and intention to use all have significant impact on elderly users' actual usage behavior of wearable devices. However, there is no obvious relationship between health anxiety and actual usage behavior.						N
Comparative study on treatment satisfaction and health perception in children and adolescents with type I diabetes mellitus on multiple daily injection of insulin, insulin pump and sensor-augmented pump therapy	2017	SAGE OPEN MEDICINE	Hussain, T and Akle, M and Nagelkerke, N and Deeb, A	10.1177/2050312117694938	Diabetes management imposes considerable demands on patients. Treatment method used has an impact on treatment satisfaction. We aim to examine the relationship between treatment satisfaction and health perception with the method used for treatment of type I diabetes mellitus in children and adolescents.	Quantitative	Abu Dhabi, United Arab Emirates	children and adolescents with type 1 diabetes mellitus	In all, 72 patients were enrolled in the study. Male-to-female ratio was 1:1. Mean age (SD) for all participants was 11.4 (4.4) years with a mean (SD) duration of diabetes of 4.9 (3.5) years. Mean (SD) HbA1c was 8.1 (1.2). Breakdown of age, gender, duration of diabetes and HbA1c for each study group is illustrated in Table 3. Median (range) duration of sensor use in the sensor-augmented therapy group was 17.7 (3–30) days/month.	sensor-augmented pump therapy			Overall mean of scale for treatment satisfaction questions was 25.3, 29.7 and 31.7 for the MDI, pump and sensor-augmented pump, respectively.	The difference was statistically significant with a p value of 0.00. On post hoc Bonferroni analysis, the difference shown was statistically significant between the MDI and both the pump and the pump and sensor (p = 0.004 and 0.00, respectively). However, the difference was not significant between the pump and the sensor-augmented pump groups (p = 0.36).	A total of 19 patients used continuous glucose monitoring system (CGMS). This group scored highest among the three studied groups in majority of the treatment satisfaction questions as well as the health perception.					Q1. I feel generally good with my diabetes treatment Mean scale for this question was 3.56, 4.26 and 4.4 for the MDI, pump and sensor-augmented pump groups, respectively. ANOVA testing showed a statistically significant difference in the answers between the study groups (p = 0.003).Q3. I feel confident with dealing with my diabetes<br>The overall feeling on the confidence of diabetes management was assessed in this question, which showed a higher confidence in the pump and the sensor-augmented pump groups compared to the MDI. The difference was statistically significant with a p value of 0.012.<br>Q4. I am not worried about hypoglycemia<br>In responding to the question related to concerns about hypoglycemia, the sensor-augmented pump group scored the highest with a statistically significant difference (p = 0.049).<br>Q5. I am not worried about hyperglycemia<br>Similar to Q3, the sensor-augmented pump therapy group scored higher in response to this question. However, there was no statistical significance in the difference between groups.<br>Q6. I use correction doses when my blood sugar is		N
Veterans' Perspectives on Fitbit Use in Treatment for Post-Traumatic Stress Disorder: An Interview Study	2018	JMIR MENTAL HEALTH	Ng, A and Reddy, M and Zalta, AK and Schueller, SM	10.2196/10415	The increase in availability of patient data through consumer health wearable devices and mobile phone sensors provides opportunities for mental health treatment beyond traditional self-report measurements. The aim of this study was to gain an understanding of patients' motivations to use or not to use wearables devices during an intensive treatment program for post-traumatic stress disorder (PTSD).	Qualitative	USA	Veterans	Our participant sample represents approximately 13% (13/96) of all veterans that completed the IOP in the 9 months before the interviews. Age between 30-61, 11 male and 2 female, 4 african american and 8 white	Fitbit Use in Treatment for Post-Traumatic Stress Disorder						use the Fitbit during the IOP: increased self-awareness, its contribution to supporting social interactions, and a desire to give back. Several participants stated that they hoped their Fitbit data would benefit future veterans, move research forward in the treatment of PTSD, and support the development of the IOP.		Lack of Clarity About Purpose Lack of Meaningful Data Challenges in the Veteran-Provider Relationship The rapport between a therapist and patient is critical for effective therapy [53], yet several participants mentioned that veterans have an inherent distrust of HCPs, which made it difficult for them to share information including Fitbit data.				N
Analyzing Older Adults' Perceived Values of Using Smart Bracelets by Means-End Chain	2020	HEALTHCARE	Jeng, MY and Yeh, TM and Pai, FY	10.3390/healthcare8040494	To cope with the demands for medical care in an aging society, smart healthcare wearable devices that can measure physiological signals are being regarded as the primary tools in medical care programs, allowing the users to acquire basic health data. Although the smart healthcare wearable devices could be applied to disease management and prevention that could help older adults control their health, older adults must be willing and able to use and continue to use them. In this research, interviews conducted through means-end chain (MEC) and laddering were used to guide the older adults step-by-step by explaining abstract ideas and emphasizing value in their perceptions of specific attributes.	Mixed	Taiwan	Older adults	older adults, including 24 males (60%) and 16 females (40%). In terms of age, the 66–70-year-old group was the largest, with 24 respondents, accounting for 60% of respondents. In terms of occupation, retirees made up the largest group, with 18 respondents, accounting for 45% of the total. This was followed by people from the service industry, with 10 respondents, accounting for 25% of the total. In terms of level of education, senior high school and vocational school made up the largest group, with 32 respondents, accounting for 80% of the total. Finally, 24 respondents (60%) lived with family members, while 12 respondents lived with spouses.	Smart Bracelets						In terms of value goals, older adults want to achieve the value goals of a sense of social belonging, improved quality of life, and healthier bodies.	seniors believed that smart bracelets in mobile health devices should have the attributes of safe use, real-time information feedback, correct data, comfortable wear, and clear screen.			The device can provide the ultimate value of long-term health promotion for older adults.In terms of consequent benefits, older adults can use smart bracelets in mobile health devices to gain benefits in learning about smart products, understanding technology applications, increasing health awareness and relaxation, and satisfying curiosity.		N
Patients' Perspective on Digital Technologies in Advanced Genitourinary Cancers	2021	CLINICAL GENITOURINARY CANCER	Rodler, S and Buchner, A and Stief, CG and Heinemann, V and Staehler, M and Casuscelli, J	10.1016/j.clgc.2020.03.018	Digital technologies allow for the remote monitoring of cancer patients and thereby close an important care gap. Despite a variety of upcoming digital health-tech solutions, there is little knowledge on uro-oncologic patients' perception of digital technologies in clinical care and cancer trials. A questionnaire was developed to evaluate patients' current use, preferences, and expectations of digital health technology.	Quantitative	Germany	patients with Advanced Genitourinary Cancers	Ninety-seven patients undergoing systemic therapy for metastatic renal-cell, urothelial, or prostate cancer were included in the final analysis. Internet, smartphone, and wearable user rates were significantly higher in younger patients (100% user rate in age group 40-49 years vs. 38% in age group 80-89 years).	Digital Technologies in Advanced Genitourinary Cancers The remote monitoring of cancer patients is thus a promising field; digital follow-up of patient-reported outcomes and digital biomarkers may allow clinicians to predict, detect, and potentially prevent adverse events			Interest in activity data was higher (3.7/5) than sleeping data (2.7/5, P < .0001; Figure 3A).	Younger age was significantly correlated with more interest in sleeping data (P = .048) and was not correlated with higher interest in activity data (P = .05; Figure 3B). Whether a patient receives chemotherapy or immunotherapy had no significant impact on patient interest in activity or sleeping data in our cohort (Figure 3C). Activity data become more interesting with advancement of treatment line (3.5/5 in the first-line setting, 4.1/5 in the third-line or more advanced setting, P = .027; Figure 3D). This increment in interest was stronger in sleeping data.		was on average 2.3/5, with 18% of patients at least likely to use a wearable (Figure 2A). When patients were offered the opportunity to receive data from a wearable, the motivation was significantly higher (P = .0001), on average 2.9/5. Motivation to wear wearables showed a tendency toward higher motivation in the younger age groups, but there was no significant difference between all age groups (Figure 2B). Male gender had a significant impact on the motivation to use wearables when the generated data are received (3.0/5 vs. 2.0/5, P = .0187; Figure 2C), whereas male gender had no significant impact on willingness to use wearables when data are not received (2.3/5 vs. 1.9/5, P = .288).			An intelligent integration of digital devices into patients' everyday life is critical in order to achieve adherence.			N
Identifying critical success factors for wearable medical devices: a comprehensive exploration	2022	UNIVERSAL ACCESS IN THE INFORMATION SOCIETY	Degerli, M and Yildirim, SO	10.1007/s10209-020-00763-2	For healthy living, the successful use of wearable medical devices such as smartwatches, smart clothes, smart glasses, sports/activity trackers, and various sensors placed on a body is getting more important as benefits of these devices become apparent. Yet, the existing knowledge about the critical success factors for wearable medical devices needs to evolve and develop further. The main objective of this research is to distill salient constructs to enhance the successful use of wearable medical devices. Specifically, the study aims to identify factors, associated items, and interactions of the relevant factors.	Mixed	Turkey	patient	Dimension N %<br>Gender Female<br>536 50.7<br>Male 521 49.3 Income1<br>Education<br>Low 539 51.0<br>Mid 380 36.0<br>High 138 13.1<br>Generation<br>Gen Z 202 19.1<br>Millennials 439 41.5<br>Gen X 207 19.6<br>Boomers 209 19.8 Education<br>Education<br>Prim. Edu 61 5.8<br>High Sch 193 18.3<br>Bachelor 501 47.4<br>Master's 238 22.5<br>Doctorate 64 6.1	wearable medical devices	Device known4<br><br>Smart clothes<br>195<br>18.4<br><br>Body sensor(s)<br>258<br>24.4<br><br>Smart glass<br>288<br>27.2<br><br>Activity tracker<br>557<br>52.7<br><br>Smartwatch<br>806<br>76.3			the user characteristics factor is important in determining attitude & behavioral intention.	Compatibility, Design, Dependability, Privacy, confidentiality, and security factor Perceived ease of use Promotion		Promotion, worthiness, design, dependability, and perceived usefulness constructs for the success of wearable medical devices were found to be the top five constructs. For the promotion construct, the participants asked for more information and demonstration to enhance appropriate awareness. Intended for worthiness, the participants reiterated the importance of the affordable cost of the wearable device. For design, the participants highlighted the need for convenience and easy interaction. In the case of dependability, the participants underlined the reliability and correctness. Regarding perceived usefulness, people restated the absolute need for useful functionalities.					N
Patient and Healthcare Professional Priorities for a Mobile Phone Application for Patients With Peripheral Arterial Disease	2023	CUREUS JOURNAL OF MEDICAL SCIENCE	Sivagangan, P and Harwood, AE and Stather, PW	10.7759/cureus.33993	Supervised exercise therapy (SET) is the first-line treatment for the peripheral arterial disease (PAD), however, access and compliance are low. An alternative method of delivering this therapy is through mobile health applications, which can be more accessible and convenient for patients. The aim of this study is to evaluate patient, public and healthcare professional (HCP) priorities with regard to a dedicated mobile phone application to deliver remote SET.	Quantitative	UK	patient		a Mobile Phone Application for Patients With Peripheral Arterial Disease			Eighty-four per cent wanted their therapy to be monitored by their vascular team, with no patients selecting that they were not at all interested in this notion (Figure (Figure1).1). The majority of patients (78%) were also interested in being able to measure their walking distances with 79% also wanting their vascular team to be able to view their walks. Most patients (76%) were interested in being able to watch exercise videos and 62% were interested in being able to copy these exercise videos (Figure (Figure1)1).		A communication platform was also valuable to patients with 74% being very interested in being able to send messages to the team (Figure (Figure1)1) and 68% interested in sending pictures of their legs. Most patients (74%) were also keen on having the vascular team contact them through the application.<br><br>Documenting other forms of physical activity and the use of wearable technology was less valuable to patients (Figure (Figure1).1). The ability to document medications and operations/procedures and carry out dedicated tests required by the vascular team was also of high interest to patients.<br><br>Interacting with other PAD users and carrying out group activities was less desirable with only 35% being very interested in conversing with other users (Figure (Figure1).1). Additionally, 29% of patients preferred not to locate local people using the application and 41% and 38% were not interested in joining a group or setting group challenges, respectively. Also, setting individual challenges was desired by less than half of the patient respondents (45%).							The authors have declared financial relationships, which are detailed in the next section.
The SPLENDID Eating Detection Sensor: Development and Feasibility Study	2018	JMIR MHEALTH AND UHEALTH	van den Boer, J and van der Lee, A and Zhou, LC and Papapanagiotou, V and Diou, C and Delopoulos, A and Mars, M	10.2196/mhealth.9781	The SPLENDID eating detection sensor (an ear-worn device with an air microphone and a photoplethysmogram [PPG] sensor) was developed to enable complete and objective measurements of eating events. The objective of our study was to explore how potential users perceive and experience the SPLENDID eating detection sensor.	Mixed	Netherlands	people with overweight or obesity	women (mean age 22 [SD 2] years; mean body mass index [BMI] 22.5 [SD 1.9] kg/m2) interested in weight management.The prototypes were tested by 23 healthy, young adults (13 men and 10 women; age 23 [SD 3] years; mean BMI 22.6 [SD 3] kg/m2). This included both closed and open-ended questions.Twenty-two overweight, young adults (3 men and 19 women; mean age 23 [SD 2] years; mean BMI 28.0 [SD 2.3] kg/m2) tested the wearable eating detection sensor. They participated for 2 testing days.	The SPLENDID Eating Detection Sensor			Across the different stages of development, the potential users were enthusiastic about the concept. They especially liked that it provided objective information on their eating patterns.		However, they stressed that it needed to be comfortable to wear and discreet. The latest version of the eating detection sensor did not yet meet these requirements.		In particular, the wearer comfort of the sensor requires attention.<br><br>One option would be to offer different shapes and sizes of the eating detection sensor so that the users can find a sensor with a good fit.<br><br>By resolving the issues related to wearer comfort, the visibility of the eating detection sensor is likely to be reduced as well. Furthermore, the visibility of the current version of the eating detection sensor was already acceptable to some of the participants.					N
Forensic Psychiatric Outpatients' and Therapists' Perspectives on a Wearable Biocueing App (Sense-IT) as an Addition to Aggression Regulation Therapy: Qualitative Focus Group and Interview Study	2023	JMIR FORMATIVE RESEARCH	ter Harmsel, JF and Smulders, LM and Noordzij, ML and Swinkels, LTA and Goudriaan, AE and Popma, A and van der Pol, TM	10.2196/40237	Given the increased use of smart devices and the advantages of individual behavioral monitoring and assessment over time, wearable sensor-based mobile health apps are expected to become an important part of future (forensic) mental health care. For successful implementation in clinical practice, consideration of barriers and facilitators is of utmost importance.The aim of this study was to provide insight into the perspectives of both psychiatric outpatients and therapists in a forensic setting on the use and implementation of the Sense-IT biocueing app in aggression regulation therapy	Qualitative	Netherlands	Forensic Psychiatric Outpatients	Variable Values<br>Age (years), mean (SD) 29.76 (10.60)<br>Male participants, n (%) 19 (90)<br>Cultural background, n (%)<br>Western 7 (33) Non-Western 7 (33)<br>Mixed 7 (33)<br>Educational background, n (%)<br>None 1 (5) Primary education 3 (14)<br>Junior secondary education 12 (57)<br>Senior secondary education 5 (24)<br>Attitude toward new technologies, mean (SD)a 4.33 (0.86)<br>Perceived proficiency in using new technologies, mean (SD)b 8.00 (1.05)<br>Age (years), mean (SD) 35.00 (5.55) 35.11 (7.99)<br>Female participants, n (%) 5 (83) 8 (89)<br>Position, n (%)<br>Master psychologist or pedagogue 4 (67) 3 (33) Health care psychologist (in training) 0 (0) 3 (33) Clinical psychologist (in training) 1 (17) 3 (33)<br>Systemic therapist 1 (17) 0 (0)<br>Work experience (years), n (%)	a Wearable Biocueing App (Sense-IT) as an Addition to Aggression Regulation Therapy					At posttest measurement, forensic outpatients evaluated the usability of the Sense-IT biocueing app as acceptable (mean 73.13, SD 13.35). System usability was not significantly correlated with age, attitude toward new technology, or perceived proficiency in using new technologies. Notably, all patients returned the borrowed materials except for 10% (2/21), who reported that they had lost their smartwatch because of robbery. Notification issues 8 (38) Problems related to the amount (too many) or the timing (too soon or too late) of the notifications received<br><br>Connectivity issues 8 (38) Problems related to instability of the connection between smartwatch and smartphone<br><br>Use of a study-provided phone 5 (24) Problems related to the use of the app on a study-provided smartphone<br><br>Limited adaptive functionalities 6 (29) Limitations in the ability to personalize settings during the study<br><br>Design-related issues 6 (29) Problems related to personal design-related preferences<br><br>Other software issues 4 (19) Other problems related to the functions of the app<br><br>Limited battery life 4 (19) Limitations in the battery life of the smartwatch<br><br>Other hardware issues 4 (19) Other problems related to the smartwatch		At the patient level, increasing interoceptive and emotional awareness was mentioned the most. Therapists supposed that the app could help patients learn and experience how their body and mental state are connected and that reminders could help them be aware of the signals the body gives in everyday life. Furthermore, a therapist stressed the importance of these basal skills in the first stages of treatment:	The limited ease of use of the app on a research-owned smartphone was discussed as a barrier in both focus groups.<br>In the focus group of therapists working with adults, limitations in the perceived accuracy of the feedback were mentioned as barriers.<br>Limited proficiency in using technological interventions was mentioned as a barrier in both groups.<br>Both focus groups mentioned a certain amount of motivation (or at least ambivalence) to change as a facilitator, and the lack thereof was mentioned as a barrier.<br>In both groups, the feeling of being controlled by others was seen as a contraindication for the use of the app.In addition to delusional and other psychotic disorders, disorders that involve an excessive focus on physiological sensations, for instance, hypochondria, were also mentioned as barriers.	The use of the app on the patients' own smartphones was explicitly mentioned as a facilitator by the therapists in the young adult group. Furthermore, the simplicity of the rationale of the app—delivering concrete, real-time physiological feedback—was mentioned as a facilitator.<br>Perceived accuracy of the app was also discussed as an essential factor.<br>Integration into treatment was mentioned as a facilitator of implementation in both focus groups.<br>Knowledge and skills of therapists were also identified as important factors.	Advantages<br><br>Rationale 4 (19) The idea or rationale behind the app; its functionality<br><br>Simplicity 7 (33) The clarity and simplicity of the app and its functions<br><br>Awareness 8 (38) The helpfulness of the app to increase both interoceptive and emotional awareness<br><br>Behavioral support 2 (10) The helpfulness of the behavioral support messages	Disadvantages<br><br>Perceived inaccuracy 7 (33) The perceived inaccuracy of the HRa measurements or the recognized activity profiles or the perceived limitations of the app to detect subjectively experienced stress	JFtH and MLN (and, more remotely, TMVdP and AP) were involved in the development process of the Sense-IT biocueing app. They have no commercial or financial relationships that could be construed as potential conflicts of interest. LMS, LTAS, and AEG also have declared that they have no competing or potential conflicts of interest.
Cancer Patients' Perception of Usefulness of Wearable Exercise Trackers	2021	PM&R	Ng, A and Gupta, E and Bansal, S and Fontillas, RC and Amos, CE and Williams, JL and Dibaj, S and Bruera, E	10.1002/pmrj.12475	To determine the feasibility and perception of usefulness of wearable trackers in inpatient and outpatient cancer rehabilitation patients.	Quantitative	USA	Cancer Patients	A majority of the inpatient partici-pants were male (54%), white (72%), married (72%), andwith a median age of 61 years. The majority of outpatientparticipants were female (62%), white (72%), married(68%), and with a median age of 63. There was no differ-ence between inpatients and outpatients in regard to846Cancer Patients'Perception of Wearable Trackers	Wearable ExerciseTrackers			we found that 48/50 (96%) outpatients and47/50 (94%) inpatients reported wearing the wearabletracker daily. Inpatients(30/50, 60%) and outpatients (42/49, 86%) found thefeedback from the wearable tracker to be helpfu	between age, gen-der, ethnicity, marital status, or education and the per-ception of usefulness of the wearable tracker. Therewere no significant associations between patients'per-ception of wearable tracker usefulness and demographicsor symptoms reported by ESAS at baseline. More outpatient participants 37/49 (76%)than inpatient participants 29/50 (58%) reported theywould continue to wear the wearable tracker after thisstudy (Figure 2). Perception of wearable tracker'susefulness was significantly higher in the outpatients41/49 (84%) versus the inpatient participants 33/50(66%) (P=.04). More outpatients (31/49, 63%) versusinpatients (27/50, 54%) perceived the wearable trackeras helping to increase physical activity.	Both outpatientsand inpatients equally reported that the wearabletracker was comfortable to wear (92%).							UNDECLARED
Can a Free Wearable Activity Tracker Change Behavior? The Impact of Trackers on Adults in a Physician-Led Wellness Group	2016	JMIR RESEARCH PROTOCOLS	Gualtieri, L and Rosenbluth, S and Phillips, J	10.2196/resprot.6534	Wearable activity trackers (trackers) are increasingly popular devices used to track step count and other health indicators. The aim of this pilot research is to investigate the use of trackers by adults with chronic medical conditions who have never used trackers previously. Specifically, we aim to determine (1) if participants would accept and use trackers to increase their physical activity; (2) if there were barriers to use besides cost and training; (3) if trackers would educate participants on their baseline and ongoing activity levels and support behavior change; and (4) if clinical outcomes would show improvements in participants' health.	Mixed	USA	adults with chronic medical conditions	Of the 11 patients who participated in the wellness group from the start, 1 left after eight weeks for personal reasons unrelated to health and is not included in any reported results. For the 10 who completed the program, ages ranged from 39 to 77 years old with a mean age of 61 and a median age of 63. Patients included 2 (20%, 2/10) males and 8 (80%, 8/10) females. Of the 10 primarily lower-income patients, 5 (50%, 5/10) worked full-time, 1 (10%, 1/10) worked part-time, and 4 (40%, 4/10) were retired. All patients suffered from at least one of the following chronic medical problems: overweight and obesity, hypertension, type 2 diabetes, hyperlipidemia, and joint pain. All but 1 of the patients was overweight or obese. Baseline levels of physical activity, as assessed by JP through patient interviews and group counseling, ranged from almost entirely sedentary to moderately active. All patients stated that they were	Wearable Activity Tracker			Half of the participants, many of whom were individuals who had previously considered purchasing a tracker, expressed excitement and hopeful feelings, such as "grateful" and "thrilled." In contrast, the other half of the participants expressed negative or neutral feelings, including that they viewed trackers as something for athletes, "a fashion trend," "a gimmick," or what "snobby people wore" to maintain "an air of superiority." Words like "skeptical," "overwhelmed," and "unsure" were also used.<br>Comparatively, when participants described how they felt about their tracker at the time of the interview, all responses were positive. Specific words used include "fantastic," "helpful," "optimistic," "very happy," "healthy," "very satisfied," and "elation."<br><br>A further indication of attitudes towards trackers was that all participants said they would recommend a wearable activity tracker to others. In addition, 8 (80%, 8/10) said they would purchase one as gift for someone else, although 2 (20%, 2/10) expressed concern about the cost. The remaining 2 (20%, 2/10) participants were hesitant about giving a tracker as a gift since they thought it could convey a negative message to the receiver.		Use of the tracker itself varied as well. Only 50% (5/10) of the participants used the wristband with the Withings Pulse. Of the others, 1 (10%, 1/10) alternated between wearing it with the wristband and clipping it to clothing, 2 (20%, 2/10) wore it solely clipped, and 1 (10%, 1/10) kept the device in a pocket. The reasons for solely clipping the device included poor fit and discomfort: in one case due to the circumference of the band and in the other the feel of the band when sweating during menopause. In addition, 1 (10%, 1/10) participant was unable to use the device due to incompatibility with her mobile phone operating system, but used the corresponding Withings app to track step count and thus answered interview questions referring to the impact of tracking on physical activity.	Participants noted external factors that influenced their tracker use or their ability to incorporate fitness in their lives. Weather was cited by a number of participants, and the constraints of jobs and retirement were also mentioned. Many participants mentioned fluctuations in routine between weekdays and weekends and how the tracker helped them identify and change that.<br>In the time between receiving the tracker and being interviewed, 40% (4/10) of the participants' use of the trackers or adherence to their fitness routine was temporarily derailed due to external circumstances. One individual stopped walking due to a fall. Another brought the tracker on a trip but didn't immediately unpack it after returning. A third had trouble fitting exercise into her schedule when a family member was hospitalized. A fourth participant's tracker stopped working. In all these cases, despite cessation, the intention to continue physical activity remained and was emphasized during the interview process.		For many, the cost of the trackers was an impediment to purchase, but for some the cost was coupled with the lack of perceived value of trackers.Many participants expressed choice confusion due to the number of brands, models, and features on the market. However, all participants were willing to try a tracker, since being given a tracker removed the barriers of cost, perceived value, and choice confusion.Some participants expressed frustration that the tracker didn't register activities such as yoga, standing, or the use of some exercise machines.	70% (7/10) strongly believed their tracker use would have been less positive without the informal discussion about the tracker and the support from other members during each session. One participant found inspiration from another participant who had a similar work situation, who "helped me figure out how to work on getting out and getting my walks in, especially on days where I felt I couldn't make the time."<br>All participants expressed positive sentiments about the wellness group, noting the lack of competition in the group, and that the weekly meetings were "supportive" and provided a sense of "camaraderie."	Trackers as Educational Tools tracker served as a trigger to increase their physical activity by adding a walk or increasing the duration of a walk.<br><br>When describing their tracker use, participants talked about how they felt accountable to it or were kept honest by it.<br><br>Participants said they were less likely to make excuses for not being physically active and a few mentioned that they competed with themselves by trying to beat the previous day's step count, or, if they had a low step count one day, making sure the next day was better. As one said,<br><br>Participants noted the satisfaction of reaching a goal, exceeding a goal, or setting a new goal.<br><br>Receiving trackers through the wellness group appeared to amplify participants' positive experiences.		N
The Acceptance of Smart Wearable Devices through Health Cognitive	2016	INTERNATIONAL CONFERENCE ON COMPUTING AND PRECISION ENGINEERING (ICCPE 2015)	Huang, FF and Lai, YH	10.1051/matecconf/20167105005	Many people have become "health consumers" and "health conscious" by seeking products to more effectively manage health-related lifestyle aspects such as fitness activities, healthcare, sports, and rehabilitation. This study explores factors influencing people's attitudes to use lifestyle management devices.	Quantitative	Taiwan	General public	Variables N % Gender Male 96 58% Female 67 41% Education University 127 77% High school 36 22% Marriage Yes 24 14% No 139 85% Employment Yes 42 25% No 121 74% Increasing health Information Yes 151 92% No 12 7%	Smart Wearable Devices			As to the demand for smart wearable device, 82.8% (135) were willing to purchase the products at present. When asked about an acceptable payment for buying the smart wearable devices, 73.6% (120) of the respondents chose NTD$1,000 or less.		The results revealed that perceived ease of use	There are significant relationships between health anxiety and the use frequency, devices' number of health information searching. People who are more anxious about their health are more likely to seeking health information frequently and using the smart wearable device.				illness in order to understand their diagnosis and treatment options as well as by healthy people for risk evaluation and disease prevention. In this study, most of the respondents are healthy persons. And the purpose of using the smart wearable device is to increase their health information. It is supposed that the smart wearable device is helpful for disease prevention and health promotion.		N

User Preferences and Design Recommendations for an mHealth App to Promote Cystic Fibrosis Self-Management	2014	JMIR MHEALTH AND UHEALTH	Hilliard, ME and Hahn, A and Ridge, AK and Eakin, MN and Riekert, KA	10.2196/mhealth.3599	mHealth apps hold potential to provide automated, tailored support for treatment adherence among individuals with chronic medical conditions. Yet relatively little empirical research has guided app development and end users are infrequently involved in designing the app features or functions that would best suit their needs. Self-management apps may be particularly useful for people with chronic conditions like cystic fibrosis (CF) that have complex, demanding regimens. The aim of this mixed-methods study was to involve individuals with CF in guiding the development of engaging, effective, user-friendly adherence promotion apps that meet their preferences and self-management needs.	Mixed	USA	individuals with CF	Demographic characteristics n (%)<br>Age, years, mean (SD) (range) 30.2 (5.9) (21-43)<br>Race, % Caucasian 15 (100%)<br>Gender, % female 7 (47%)<br>Marital status, % married/partnered 11 (73%)<br>Education, % college degree or beyond 11 (77%)<br>School, % currently attending 2 (13%)<br>Work, % full or part time 11 (73%)<br>Annual income, % ≥US $100,000 9 (60%)<br>Insurance status, % private 12 (80%)	an mHealth App to Promote Cystic Fibrosis Self-Management			The use of prizes to motivate health behavior was also controversial. Some respondents were eager to earn rewards for completing CF treatments, especially those that were tailored to their interests or that benefitted the CF Foundation, while others did not feel that material prizes would sufficiently motivate them.		Participants raised several points about the app design and usability. A central issue for many was ease of use, with minimal burden or effort to input medical data or set up scheduled alarms. Participants described frustration with other apps having "tedious" data entry and access processes [age 33, female] that limited the app's usefulness.		(1) information at one's fingertips, (2) automation of functions and integration with other technologies, (3) communication with health care providers, and (4) socialization within the CF community (described below, with representative quotes in Tables 2- -5).5).<br><br>One point of agreement was the importance of comparing adherence behaviors rather than clinical indicators (eg, lung function, weight), because individuals have more control over their behaviors than their health outcomes.<br><br>Clear, intuitive navigation was important, with the ability to easily search data within the app. Participants wanted graphical displays to be presented in digestible, easily understood, and mobile-friendly visual formats. Simple features, such as a basic alarm or medication reminder system, were generally not of interest; rather, participants favored app designs that integrated multiple functions within a single, cohesive program.<br><br>with so many possible features within a single app, several participants also emphasized the need for customization options, such as selecting which features to use and privacy settings.<br><br>Finally, a common recommendation was to sync the app across multiple devices so that the data and functionality could be updated and accessed				One of the clearest overarching messages was the desire for an app tailored to the unique, complicated experience of having and managing CF. A common challenge of using available health-related apps is that they may or may not fit with CF treatment goals. For instance, nutrition or diet apps are typically designed for weight loss, which is in stark contrast to CF nutrition guidelines that call for high calorie, fat, and sodium intake and often weight gain.	N
Acceptance of Commercially Available Wearable Activity Trackers Among Adults Aged Over 50 and With Chronic Illness: A Mixed-Methods Evaluation	2016	JMIR MHEALTH AND UHEALTH	Mercer, K and Giangregorio, L and Schneider, E and Chilana, P and Li, M and Grindrod, K	10.2196/mhealth.4225	Physical inactivity and sedentary behavior increase the risk of chronic illness and death. The newest generation of "wearable" activity trackers offers potential as a multifaceted intervention to help people become more active. To examine the usability and usefulness of wearable activity trackers for older adults living with chronic illness.	Mixed	Canada	Adults Aged Over 50 and With Chronic Illness	Age<br>64 (52-84)<br>Gender<br><br>Male 9 (28)<br>Female 23 (72)	Wearable Activity Trackers			All the wearable activity trackers tested had moderate acceptability, with the standard pedometers having the lowest acceptability to users		App Positive statement<br>Pedometer "I just want a step counter, I don't care about the rest of the stuff"<br>Fitbit Zip "The Fitbit I still say is the easiest. I actually got an email that tells you your weekly progress, and I really like that."<br>Jawbone Up 24 "I liked the sleep data a lot, and I found the Jawbone easy to use. I found it really easy, it was light."<br>Misfit Shine "I really liked that I could wear it in the water An issue brought up by each group was that there were no instruction manuals, which prevent them from feeling comfortable with the various devices. App Negative statement<br>Pedometer "The very first one, the pedometer was unbelievably off. I would take one step, and it would count 10."<br>Fitbit Zip "It was too small, I was scared I would lose it"<br>Jawbone Up 24 "Jawbone, see look, this is by far the easiest to put on and off but once it's on it can get a little annoying"<br>Misfit Shine "The Shine I thought was just the most unintuitive, poorly constructed system"<br>Withings Pulse "The reason I didn't choose it is because I was afraid that over time it would all break."	TAM identifies behavioral intention to use as one of the key attitudes to adoption. If a participant wanted to make lifestyle choices, he/she is more likely to identify a perceived usefulness of a device, which increased their intention to use it and eventually adopt the technology. In all groups, participants had been asked by a physician to exercise more, and agreed that they needed to be more active. Tracking Participants were less interested in being motivated by the activity tracker than in being motivated by the self-awareness gained from data collected by the tracker. The perceived usefulness and behavioral intention to use help the users to find clarity around real and perceived activity levels. This clarity was a step toward finding an intrinsic motivation to become more active.	There is a perception that the navigation of devices and apps requires technological know-how that is often absent in the older adult and elderly population. Modifications of the TAM have identified technical know-how as a form of self-efficacy that influences a user's perceived ease of use and attitude toward using [69,71,72]. It was clear that improvements could be made to the devices and associated apps to make them more accessible for older adults.	Those who felt they would not purchase a tracker included reasons of cost, lack of interest, and complexity of the devices. Tables 5 and and66 present the positive and negative associated with individual fitness trackers, respectively.TAM additionally identifies the lack of instructions as a barrier to a person adopting, because it is a barrier to actual system use and can imply to the user that they should be able to use the device and that difficulties are personal failing. In addition to learning to use the device, users must also learn to speak the language of the device, including terms such as "active time," "1000 points," and "link with Bluetooth." Several times a barrier to learning was that the participant asked older/adult children for help using the device, and was met with impatience and frustration.	The common reasons for liking or disliking a device are how they looked, be it for subtle or fashion-based factors, and ease of use. The participants' perceived comfort level with the devices was another notable aspect of this study that drove how participants ranked the device. In Canada, physical activity trackers are not taxed if bought with a prescription. Several of our participants also stated they wished the devices were available in pharmacies because they did not go into the electronics stores where the devices are traditionally sold. There was also a noted desire to learn about the devices from someone in health care. The participants were interested if their doctors or other health care professionals would be interested in the data provided from the devices. There was also a noticed interest in pharmacies carrying the devices, and having pharmacists able to explain how to use them, similar to how health-monitoring systems such as			N
Feasibility and Acceptability of Wearable Cameras to Assess Self-care in People With Heart Failure: Pilot Study	2023	JMIR FORMATIVE RESEARCH	Askman, S and Lof, M and Maddison, R and Nourse, R	10.2196/40536	Heart failure (HF) is a common chronic condition that affects over 26 million people worldwide. It is a progressive and debilitating disease with a broad symptom profile, intermittently marked by periods of acute decompensation. People with HF generally do not self-manage their condition well (eg, monitoring symptoms, taking medications regularly, physical activity, etc). A better understanding of self-care activities and what factors may indicate deterioration is warranted.The aim of this study was to determine the feasibility and acceptability of using wearable cameras to assess self-care activities in people with HF. The study objectives were to (1) explore whether changes in self-care activities could be identified prior to hospitalization and (2) determine the acceptability of wearable cameras to people with HF.	Quantitative	Australia	people with HF	Characteristics All participants (N=30)<br>Not hospitalized (n=20, 66%)<br>Hospitalized (n=10, 33%)<br>Age (years), median (IQR) 77 (67-83) 73.0 (66-82) 80.5 (73-87)<br>Sex, n (%)<br>Male 18 (60) 12 (60) 6 (60)<br>Female 12 (40) 8 (40) 4 (40)<br>NYHAa class, n (%)<br>II 10 (33) 7 (35) 3 (30)<br>III 20 (66) 13 (65) 7 (70)<br>Years since HFb diagnosis, n (%)<br>≤5 18 (60) 12 (60) 5 (50)<br>5-10 3 (10) 2 (10) 1 (10)<br>10-20 7 (23) 5 (30) 2 (20)<br>>20 2 (7) 0 (0) 2 (20)	Wearable Cameras to Assess Self-care in People With Heart Failure			All participants (N=30) were presented with 8 statements of acceptability, scored on a 5-point Likert scale. Overall, participants reported high acceptability for wearing the cameras										N
Older Adults' and Clinicians' Perspectives on a Smart Health Platform for the Aging Population: Design and Evaluation Study	2022	JMIR AGING	Cristiano, A and Musteata, S and De Silvestri, S and Bellandi, V and Ceravolo, P and Cesari, M and Azzolino, D and Sanna, A and Trojaniello, D	10.2196/29623	The objective of this study is 2-fold; we aimed to design the functionalities of a smart health platform addressing 5 chronic conditions prevalent in the older population (ie, hearing loss, cardiovascular diseases, cognitive impairments, mental health problems, and balance disorders) by considering both older adults'and clinicians' perspectives and to evaluate the identified smart health platform functionalities with a small group of older adults.	Quantitative	Italy	older adults	A total of 24 participants ([illegible] women) aged >65 years completed the web-based questionnaire for older adults. The sample was distributed as follows: 67% (16/24) in the 65 to 70 age range group, 17% (4/24) in the 71 to 75 age range group, 8% (2/24) in the 76 to 80 age range group, and 8% (2/24) in the >81 years group. Most respondents (16/24, 67%) declared that they lived with someone (all of them claimed to share their home with their spouse and 5/24, 21% with their progeny as well). The medical conditions prevalent among participants were hypertension (9/24, 38%), cardiovascular disease (6/24, 25%), anxiety (4/24, 17%), hearing difficulties (3/24, 13%), arrhythmias (4/24, 17%), balance disorders (2/24, 8%), and depression (1/24, 4%). Instead, 21% (5/24) of participants claimed to have experienced none of the abovementioned disorders.	a Smart Health Platform for the Aging Population						Even older adults (18/24, 75%) expressed interest in receiving periodic reports about their own health status (weekly reports were preferred by 4/24, 17%, whereas monthly reports were favored by 20/24, 83%). In addition, they would like their own clinician (8/24, 33%), wife or husband (9/24, 38%), son or daughter (7/24, 29%), or none (4/24, 17%) to have access to the content of those reports. Approximately 63% (15/24) of the older adults showed an interest in notifications and suggestions that the platform could generate based on the collected data. This information was expected to be shared with the spouse (11/24, 46%), son or daughter (6/24, 25%), physician (2/24, 8%), and friend (2/24, 8%), whereas some (3/24, 13%) of participants preferred that no one had access to such notifications and suggestions.				Less unnecessary visits 40 (33.9) 3 (12.5)<br>Enhanced patient's safety 39 (33.1) 9 (37.5)<br>Better self-management of patients' health status 77 (65.3) 10 (41.7)<br>Better patient's social interactions 37 (31.4) 4 (16.7)<br>Enhanced patient's autonomy — 3 (12.5)<br>Enhanced patient's confidence 55 (46.6) 4 (16.7<br>Improved patient's health status 12 (10.2) —<br>Improved patient's diet habits 28 (23.7) 2 (8.3)<br>Improved patient-physician communication 9 (7.6) —<br>Time saving 23 (19.5) 6 (25)<br>Money saving 19 (16.1) 2 (8.3)	Privacy 19 (16.1) 5 (20.8)<br>Change of routine 28 (23.7) 3 (12.5)<br>Erroneous measurements 37 (31.4) 4 (16.7)<br>Erroneous notifications (suggestions by platform) 31 (26.3) 3 (12.5)<br>Technical issues of the devices 60 (50.8) 7 (29.2)<br>Education on devices and platform use 75 (63.6) 2 (8.3)<br>Increased stress for the user 25 (21.2) 5 (20.8)<br>Other: decreased patient's referral to private practice 2 (1.7) —	N
Impact of Actual Use Behavior of HealthcareWearable Devices on Quality of Life: A Cross-Sectional Survey of People with Dementia and Their Caregivers in Ghana	2022	HEALTHCARE	Lamyo, E and Dai, BZ and Lamyo, A and Nutakor, JA and Ampon-Wireko, S and Nkrumah, ENK and Appiah, R	10.3390/healthcare10020275	The increasing prevalence of dementia has necessitated the introduction and use of healthcare wearable devices to augment the healthcare services delivered to people with dementia (PWDs). Unfortunately, evidence on user feedback and the real impact of the actual use of these devices on the quality of life of PWDs has not been fully explored, especially in the context of developing countries. This study, therefore, seeks to evaluate the impact of actual use behavior (AUB) of healthcare wearable devices on the quality of life (QoL) of PWDs in Ghana by using constructs from the extended Unified Theory of Acceptance and Use of Technology (UTAUT2) model and the SF-36 quality of life instrument.	Quantitative	Ghana	People with Dementia and Their Caregivers in Ghana	Age (years)<br>18 years 4 (7%) 0 (0%)19 to 31 13 (22%) 0 (0%)32 to 44 19 (33%) 0 (0%)45 to 54 14 (24%) 75 (28%)55 to 64 6 (10%) 120 (46%)65 to 74 2 (3%) 54 (21%)75 and over 0 (0%) 13 (5%)<br>Gender, n (%)<br>Female 32 (55%) 143 (55%)Male 26 (45%) 119 (45%)<br>Marital Status<br>Single 33 (57%) 44 (17%)Married 12 (21%) 121 (46%)Widowed 0 (0%) 25 (10%)Widower 0 (0%) 23 (9%)Divorced 13 (22%) 49 (19%)<br>Level of Education<br>No Formal Education 3 (5%) 19 (7%)Primary School 4 (7%) 56 (21%)Less Than JHS 1 (2%) 2 (1%)Junior High School (JHS) 7 (12%) 82 (31%)Less Than SHS 15 (26%) 15 (6%)Senior High School (SHS) 3 (5%) 37 (14%)Nursing Diploma 5 (9%) 17 (6%)Teacher College Diploma 7 (12%) 16 (6%)Higher National Diploma (HND)	healthcare Wearable Devices on Quality of Life					The results showed a significant positive relationship between AUB and QoL (p < 0.001). Other predictors such as behavioral intention, facilitating conditions, effort expectancy and social influence were also found to significantly impact the actual use behavior of healthcare wearable devices among PWDs.					The study also revealed that 4% (14) of patients rated their reported health transition associated with the use of healthcare wearable devices as excellent, 42.5% (136) as very good, and 39.4% (126) as good, respectively at the time of the study compared to a year ago.		N
Users' experiences of wearable activity trackers: a cross-sectional study	2017	BMC PUBLIC HEALTH	Maher, C and Ryan, J and Ambrosi, C and Edney, S	10.1186/s12889-017-4888-1	Wearable activity trackers offer considerable promise for helping users to adopt healthier lifestyles. This study aimed to explore users' experience of activity trackers, including usage patterns, sharing of data to social media, perceived behaviour change (physical activity, diet and sleep), and technical issues/barriers to use.	Quantitative	Australia	General public	On average, participants were aged 33.1 years (SD 12.4, range 18–70 years), and approximately two thirds were female. There were no sociodemographic differences between current and former users with the exception that current users were more likely to be in a relationship than former users (X 2 (2) = 8.98, p = .01).<br><br>The most commonly used brand of activity tracker was Fitbit (67.5%, n = 160), followed by Garmin (16.5%, n = 39), Apple (3.4%, n = 8), Jawbone (2.5%, n = 6), Samsung (1.7%, n = 4), Polar (1.3%, n = 3), and other (7.1%, n = 17). Overall, users were most likely to obtain their activity trackers by purchasing it themselves (56.58%) or receiving it as a gift from their family (43.5%).<br><br>Participants were asked how long they had been using their activity trackers.	wearable activity trackers			Overall, 94% of current users and 65% of former users agreed that they had had a positive overall experience using their activity tracker. The majority of current users either strongly or somewhat agreed that various features on their trackers were useful, including: steps (95%), active minutes (76%), sleep (66%), heart rate (63%), stairs climbed (58%), energy burned (57%). Fewer agreed that the food intake feature was useful (36%). Participants were also asked their opinions on the usefulness of real time monitoring and long term monitoring features. The majority of current (89%) and former (54%) users agreed that the real time monitoring was useful, while slightly fewer current users (78%) and former users (41%) agreed that long term monitoring was useful.		However, some aspects of ease of use did vary; specifically, Fitbit users rated the stair climbing, heart rate and dietary intake features as being significantly easier to use than Garmin users did (p = 0.01–0.02).<br><br>There was no significant difference between the total number of complaints reported by Fitbit users (Mdn = 2.00) and Garmin (Mdn = 1.50) users (U = 1419.00, z = −1.36, p = .17, r = .11). However, Fitbit users were more likely to report complaints relating to their activity trackers' battery life (X 2 (1) = 12.59, p < .01), whilst Garmin users were more likely to report problems with their activity trackers' screens. most commonly relating to the tracker not suiting their outfit (19%), low battery life (19%), difficulties with the support software (17%) or perceived inaccuracy of data collected (17%). Former users reported more issues than current users overall (U = 1648.5, z = − 2.36, p = .02, r = .18), with complaints typically relating to battery life (30%), difficulties with the support software (30%), the device falling off (22%) and wear and tear issues (22%)	Overall, activity trackers mainly motivated users to monitor activity patterns (35.9%, n = 85), improve fitness (27.4%, n = 65) and improve health (18.1%, n = 43) (see Additional file 2: Table S1 for full details).		.Former users were asked to identify why they no longer use their activity tracker, and the main reasons given were that they felt they had learnt everything they could from their tracker (30%), their tracker was broken (22%), and/or their tracker was not helping them achieve their goals (14%; Table 4).		The majority of current (81.4%) and former (51.3%) users believed that they incorporated more physical activity into their day whilst wearing their activity tracker. In contrast, only 40.2% of current users and 13.5% of former users reported they had improved their eating patterns as a results of using their tracker, and even fewer reported they had changed their sleeping pattern (24.1% of current users and 10.8% of former users).		N
Exploring factors affecting consumers' adoption of wearable devices to track health data	2023	UNIVERSAL ACCESS IN THE INFORMATION SOCIETY	Pancar, T and Yildirim, SO	10.1007/s10209-021-00848-6	In this research, the factors affecting the adoption of wearable devices to track health information are investigated.	Mixed	Turkey	General public	All Male 18–34 35–44 Over 45 Up to 1 year 3 or more years<br>1263 834 439 827 216 220 699 549	wearable devices to track health data						However, interviews showed that there is a significant social aspect driving wearable device use.	That means the visual appearance of the wearable devices is somewhat crucial for them. the importance of the wearable device's compatibility with the user's existing technology stack or technology ecosystem. Users are preferring devices fitting their existing smartphones, computers, etc.	[illegible] price is a significant factor for adopting wearable devices, and interviews strongly confirmed the importance of price. Performance expectancy was identified as a very important factor in the survey-based analysis. Quantitative analysis of the survey highlighted habit as a very important factor.<br><br>According to the quantitative analysis of the survey, social influence is not a significant factor for the use of wearable devices.			Privacy was not mentioned as an important factor for most of the interviewees. They stated that other devices such as smart assistants they are using at home have access to more sensitive information, and they do not see a higher risk to privacy related to their wearable device usage, or it is an acceptable level of risk.	N
Patient satisfaction in emergency department: Unveiling complex interactions by wearable sensors	2021	JOURNAL OF BUSINESS RESEARCH	Stefanini, A and Aloini, D and Gloor, P and Pochiero, F	10.1016/j.jbusres.2019.12.038	We analyzed the interaction between patients and providers in the emergency department of a large university hospital using Sociometric Badges.	Mixed	Italy	patient		wearable sensors						Results show that patient satisfaction and service perceptions are greatly influenced by behavioral and network factors. Patients appreciate the physical closeness of the doctors and the providers' continuous monitoring of their health conditions. They also desire to be actively involved into the communication network with practitioners. In addition, patients perceive positively teams where doctors take the leadership of the communication network and ensure an effective team conversation.						UNDECLARED
Too old for technology? Use of wearable healthcare devices by older adults and their willingness to share health data with providers	2021	HEALTH INFORMATICS JOURNAL	Chandrasekaran, R and Katthula, V and Moustakas, E	10.1177/14604582211058073	Drawing upon a national survey in US with 1481 older adults, we examine the use of wearable healthcare devices and the key predictors of use viz. sociodemographic factors, health conditions, and technology self-efficacy.	Quantitative	USA	older adults	Significant differences were observed between users and non-users of healthcare wearable devices across gender, education, and household income. Seniors who were women (10.4%), white (13.93%), those with some level of college education or college graduates (11.91%), and annual household incomes above $75K (9.71%) were most likely to report using wearable healthcare devices. There were no significant differences based on marital status.	wearable healthcare devices by older adults				Our results indicate that older men are less likely to use wearable healthcare devices, as compared to older women (OR = 0.62, 95% CI 0.36–1.04). Compared to seniors whose educational qualifications were less than high school, others with better education were five to nine times more likely to use wearables. Asian seniors were twice more likely to use wearables as compared to whites (OR = 2.60, 95% CI 0.89–7.64). We also found a strong positive association between annual household income and the use of wearable devices. The likelihood of wearable use steadily increased for seniors as their annual household income levels increased.<br>Only one out of the three health-care–related variables exhibited a significant association with wearable use. We found that healthy seniors are almost twice more likely to use wearables as compared to those with self-reported poor health (OR = 1.98, 95% CI 1.37–2.87). The number of chronic conditions and the frequency of visits to the healthcare provider did not emerge as significant predictors.<br>Technology self-efficacy turned out a significant									N
Using a Mobile Social Networking App to Promote Physical Activity: A Qualitative Study of Users' Perspectives	2018	JOURNAL OF MEDICAL INTERNET RESEARCH	Tong, HL and Coiera, E and Laranjo, L	10.2196/11439	Despite many health benefits of physical activity, nearly a third of the world's adult population is insufficiently active. Technological interventions, such as mobile apps, wearable trackers, and Web-based social networks, offer great promise in promoting physical activity, but little is known about users' acceptability and long-term engagement with these interventions. The aim of this study was to understand users' perspectives regarding a mobile social networking intervention to promote physical activity.	Qualitative	Australia	General public	Characteristics Value<br>Age, mean (SD) 23.6 (4.6)<br>Female gender, n (%) 28 (51)<br>Weight, mean (SD) 78.1 (22.3)<br>BMIa (kg/m2), mean (SD) 26.5 (6.8)<br>BMI categoriesb, n (%)<br>17-18.49 3 (6)<br>18.5-24.9 24 (44)<br>25-29.9 15 (27)<br>≥30 13 (24)<br>Steps/day, mean (SD) 9937 (3527)<br>Marital status, n (%)<br>Single 27 (49)<br>In a relationship 22 (40)<br>Married or de facto 6 (11)<br>Daily smartphone use (hours), mean (SD) 5.6 (3.4)<br>Most used appsc, n (%)<br>Social media 49 (89)<br>Fitness apps 6 (10)<br>Occupation, n (%)<br>Student 42 (76)<br>Other 13 (24)<br>Smartphone, n (%)	Mobile Social Networking App to Promote Physical Activity						At the individual level, participants mentioned that goal setting, self-monitoring, and feedback were important for their physical activity. At the social level, social comparison and the connection with other users in terms of familiarity and similarity were considered motivating. Finally, at the technological level, automation and personalization were considered to be facilitators		technological limitations were observed as reducing user engagement. As time went on, many users described the feeling that the novelty of the technology had worn off, and they started to think of it as a chore. Even apparently simple tasks like charging the devices were seen by participants as an extra burden in their already busy daily routines Technical Problems and User Experience Technical problems were often described as a common cause for attrition (Textbox 3, quote 8). In addition, user experience factors, such as the design aspects of the interface and its usability, were reported as important aspects of engagement and continued use.		Specifically, wireless devices provided an automatic way for users to collect and self-monitor personal measurements, and their integration with the mobile app provided a user interface platform for participants to visualize those data and to review progress .<br>Many users mentioned that having personalized information and services would also support long-term engagement, as they could offer the advantage of providing relevant information tailored to each specific user, thus eliminating the cognitive burden of dealing with information overload. Many users described that personalization should go beyond the content generated by the system and extend to the provision of relevant services .		EC could benefit from commercialization of fit.healthy.me.

Attitudes and perceptions of adults of 60 years and older towards in-home monitoring of the activities of daily living with contactless sensors: An explorative study	2015	INTERNATIONAL JOURNAL OF NURSING STUDIES	Claes, V and Devriendt, E and Tournoy, J and Milisen, K	10.1016/j.ijnurstu.2014.05.010	Contactless monitoring is increasingly used to enhance qualitative and cost-effective care for older persons. Succesful integration of this technology in older peoples' daily lives, depends on their acceptance of these systems. The primary purpose was to explore attitudes and perceptions of adults of 60 years and older towards contactless monitoring of the activities of daily living.	Quantitative	Belgium	Older adults	The average age was 72.4 years (range = 60–90, standard deviation SD = 6.39) and most respondents were women (n = 162, 67.8%). Educational levels varied considerably, but most respondents (n = 215, 90.4%) at least completed secondary education and were currently retired (n = 232, 97.1%). The majority did not provide informal care (n = 139, 67.8%) neither received informal or professional care at home (n = 157, 68.3%). At least half of the sample had experience with using technologies such as a computer (n = 146, 61.6%) or the Internet (n = 141, 59.5%), but only eight respondents (3.4%) indicated using a personal alarm system. Most respondents perceived themselves as being in very good or good health (n = 190, 77.5%), being as active as peers (n = 127, 51.8%) and as having only occasional memory problems (n = 143, 58.4%)	in-home monitoring of the activities of daily living with contactless sensors							Respondents found it very important that professional caregivers or installers would be able to answer their questions about the monitoring (96.7%) and that they could temporarily switch off the technology if desired (93.0%). Most respondents found it important that the monitoring should function automatically (92.1%) and that it should still be operable by the older end-user (81.8%).			The technology was found most useful to remain living at home longer (94.3%), both safely (93.7%) and independently (94.8%). Timely detecting and receiving assistance for both emergency situations (93.6%) and gradually emerging health problems (86.5%) was also found valuable and monitoring did not seem redundant to the use of other aids (90.7%). In general, contactless monitoring was embraced for its usefullnes in later life (95.5%), only a minority of respondents (25.3%) indicated it could already be useful at this moment in their life (Table 3).Most respondents indicated the information collected through contactless monitoring could be useful for both professional (94.7%) and informal (86.9%) caregivers and were willing to share the information with these third parties (94.2% and 85.7% respectively). In contrast, more than half of the respondents indicated that avoiding unnecessary worries (56.8%) and control of daily activities (54.4%) could be reasons to forbid access to informal caregivers. The possibility to have access to the data themselves was also highly valued (90.4%) (Table 3). Furthermore, respondents cleary indicated the	Most concerns were expressed regarding the possibility that the technology would give unintelligible error messages (71.7%), would be maladjusted to limitations that may arise with ageing, such as problems with vision, hearing, manipulating small buttons (70.3%) or would infringe on the own privacy (64.4%). A possible feeling of stigmatization (35.1%) or visibility for visitors (30.6%) were seen as minor concerns.However false alarms were a cause of concern for (60.3%); of the repondents there was no unanimity whether they would be experienced as a burden (68.3%) or as a positive sign that the monitoring works (56.3%). Mixed attitudes and perceptions were also associated with the use of video cameras in a system for contactless monitoring. Most respondents (82.3%) would find video cameras useful. On the contrary, 41.1% would not accept this type of sensors due to privacy infringement. The acceptation rate rised considerably when	N
Motivation and User Engagement in Fitness Tracking: Heuristics for Mobile Healthcare Wearables	2017	INFORMATICS-BASEL	Asimakopoulos, S and Asimakopoulos, G and Spillers, F	10.3390/informatics4010005	We report on a 4-week situated diary study and Healthcare Technology Self-efficacy (HTSE) questionnaire assessment of 34 users of two popular American fitness trackers: JawBone and FitBit. The study results illustrate design implications and requirements for fitness trackers and other self-efficacy mobile healthcare applications.	Mixed	USA	General public	Participants' ages ranged from 18–60. The majority (n = 31) had bachelor's and/or master's degree, and 3 a doctorate degree. Over half (n = 28) reported using their tracker for more than 6 months. The majority of participants reported using other personal wellness tools in addition to activity trackers, including food journaling tools like MyFitnessPal and sleep tracking tools, such as Sleep Cycle.	Fitness Tracking						Specific factors which impacted user motivation were as follows (a) Seeing if I met my goal (movement/sleep/calories count); (b) Look and feel good, improve mood and avoid sitting; and (c) Getting tips and recommendations from the app (Fitbit offers self-interpreted data, while Jawbone offers data interpreted by a SmartCoach).<br>From a UX point of view, using the application to work on a personal health or wellness issue (e.g., sleep, weight, mobility) is a driver for believing in the motivational power of the device	Many users suggested a need to build trusting relationships with and between users, giving regular recognition and high-fives. Fitness tracker data is considered fun and often entertaining (JawBone SmartCoach uses some humor to cause self-reflection). Fitbit and Jawbone users expressed the need for relatedness with family and/or friends as they were interacting with the applications. In addition, participants were constantly checking the weeks' data to gain insight into the correlation between their fitness activities and wellness. However, both Jawbone and Fitbit did not provide an easy way to see trending or patterns to users, with a UX strategy that seems to focus on current fitness status data.<br>Although users generally felt motivated that they should be improving, FitBit did not provide direction as to what users should focus and improve upon. JawBone provided users with tips and hints as to how to respond to their activity tracking, but this caused more variability in their behavior over time (see Figure 2). FitBit users in comparison had more consistent motivation patterns over the 4-week reporting period. Generally, users enjoyed the level of autonomy they were granted by the apps. However, some stated a need for apps to balance autonomy with more self-directed goal creation to support their engagement.		Users reported using numerous competence-supportive strategies including affording users with the required skills, differentiation of exercise sequences, encouraging self-reflection, giving opportunities for receiving constructive feedback. These features include notes and comments, or competitions with other users, offering reflection on their own progress. Apps that can map users' awareness and their achievements allow them to provide targeted competence support. Fitbit users reported increased confidence and competence with using the app; however, they complained that they could not control the speed at which levels/sessions progressed. Notifications in the Fitbit app, and in Jawbone to a lesser extent - offers a component that allowed participants to track their progress and plan alternate or sustained activities, while reflecting on the impact of their actions. It can also be used for	Users reflected positively on the use of the apps, with one user felt that the autonomy-supportive style was evident in terminology used. Users felt motivational value from seeing steps, styles and advice (Jawbone). User attitudes reinforced autonomy stating it made use of the device more engaging and positively influenced sustained or repeat use.The majority of participants reported fitness tracking applications and devices to be useful for their motivation and behavior: enhance their motivation and willingness to undergo change in their fitness activities.		N
Monitoring Patients Reported Outcomes after Valve Replacement Using Wearable Devices: Insights on Feasibility and Capability Study: Feasibility Results	2021	INTERNATIONAL JOURNAL OF ENVIRONMENTAL RESEARCH AND PUBLIC HEALTH	Ocagli, H and Lorenzoni, G and Lanera, C and Schiavo, A and D'Angelo, L and Di Liberti, A and Besola, L and Cibin, G and Martinato, M and Azzolina, D and D'Onofrio, A and Tarantini, G and Gerosa, G and Cabianca, E and Gregori, D	10.3390/ijerph18137171	Wearable devices (WDs) can objectively assess patient-reported outcomes (PROMs) in clinical trials. In this study, the feasibility and acceptability of using commercial WDs in elderly patients undergoing transcatheter aortic valve replacement (TAVR) or surgical aortic valve replacement (SAVR) will be explored.	Quantitative	Italy	patients undergoing SAVR and TAVR	(10) 0.31<br>Male 60% (3) 33% (4) 41% (7)<br>Age 17 76/78/79 79/82/85 78/79/83 0.046<br>Marital status Married cohabitant 17 80% (4) 67% (8) 71% (12) 0.58<br>Widowed unmarried 20% (1) 33% (4) 29% (5)<br>Educational level Primary 16 40% (2) 64% (7) 56% (9) 0.38<br>Secondary 60% (3) 36% (4) 44% (7)	wearable device			The perceived risk dimensions score was higher in patients that underwent SAVR. Four patients reported that they would wish to continue to use the device (3 TAVR, 1 SAVR).		The device characteristics satisfied the patients, the equipment characteristics reached for both group the maximum score, the same for perceived ease of use, also perceived usefulness showed good levels.						Conversely, privacy concern (median 8 vs. 9, SAVR vs. TAVR), perceived risk (10 vs. 5 SAVR vs. TAVR) and subjective norm (10 vs. 9, SAVR vs. TAVR) showed lower level of satisfaction.	N
WILL YOU CARRY THAT WATCH? INVESTIGATING FACTORS AFFECTING CONTINUANCE INTENTION OF SMARTWATCHES	2021	INTERNATIONAL JOURNAL OF CONTEMPORARY ECONOMICS AND ADMINISTRATIVE SCIENCES	Enginkaya Erkent, E and Kose, SG and Ozer Cizer, E	10.5281/zenodo.5831643	The interest in wearable technologies, especially smartwatches rise day by day parallel with technological developments and an increasing need to monitor health. In line with those developments, this study aims to investigate the role of perceived ease of use, perceived usefulness, user satisfaction, healthology in explaining smartwatch continuance intention. In addition, this study investigates the relationships between perceived ease of use, perceived usefulness, healthology and user satisfaction.	Quantitative	Turkey	General public	females (52,3%) and 218 males (47,7%). It was observed that most participants were in the second category of age (%45,5%). The education level of participants showed 63,2% at master's degree and doctorate levels, %27,6 at the bachelor's degree and associate's degree levels, and 9,2% at primary and secondary education levels. The majority of the participants' household income is 10.001 TL and above (40,5%)	SMARTWATCHES				Findings demonstrate that the most powerful variable to explain smartwatch continuance intention is perceived usefulness	, whereas perceived ease of use contributes to user satisfaction the most.	Also, healthology is positively related to both user satisfaction and continuance intention.						UNDECLARED
"I shy away from them because they are very identifiable": A qualitative study exploring user and non-user's perceptions of wearable activity trackers	2021	DIGITAL HEALTH	Burford, K and Golaszewski, NM and Bartholomew, J	10.1177/20552076211054922	The purpose of this study was to qualitatively explore the perceptions of wearables among active adult users and non-users.	Qualitative	USA	General public	Characteristic Total sample (n = 20) Non-users (n = 10) Users (n = 10)<br>Age (years), M (SD) 33.0 (9.2) 32.5 (8.1) 35.0 (10.4)<br>Female, n (%) 11 (55) 3 (30) 8 (80)<br>Race/ethnicity, n (%)<br>White 17 (85) 9 (90) 8 (80)<br>African–American 0 (0) 0 (0) 0 (0)<br>Asian/Pacific Islander 3 (15) 1 (10) 2 (20)<br>Hispanic 1 (5) 0 (0) 1 (10)<br>Physical activity per week, n (%)<br>0 days 0 (0) 0 (0) 0 (0)<br>1–2 days 0 (0) 0 (0) 0 (0)<br>3–4 days 6 (30) 4 (40) 2 (20)<br>5–6 days 9 (45) 3 (30) 6 (60)<br>7 days 5 (25) 3 (30) 2 (20)<br>Wearable activity tracker, n (%)<br>Fitbit 4 (40) 0 (0) 4 (40)<br>Apple watch 3 (30) 0 (0) 3 (30)<br>Garmin 2 (20) 0 (0) 2 (20)<br>Fitbit and Garmin 1 1 (10) 0 (0) 1 (10)	wearable activity trackers			The majority of users expressed positive emotional reactions towards their devices.		Interestingly, there were three non-users that reported former usage of a wearable. For these non-users, there were certain flaws, such as charging the device, ease of use, and even skin irritation that that led non-users to stop using a device:	Wearable users typically held positive perceptions towards their wearables by discussing the accountability and validation provided by the devices. More specifically, the objective feedback (e.g. steps, heart rate, distance, calories, sleep) seemed to motivate participants to maintain consistency in their activity. Users also mentioned that they purchased their wearables for these performance indicators. The objective data made many users feel confident about their physical activity experience because they could see the results and progress: In addition, non-users discussed differences in motivation as a major reason for owning or not owning a device. Many of these participants were avid exercisers who were intrinsically motivated to be active each day as reflected in their reported physical activity. They did not perceive a need for an outside source of motivation because of their long-lasting relationship and existing goals with physical activity:	Non-users thought that the data needs to be more user-friendly and suggestive of their exercise performance. In addition, more insightful information about their effort during exercise might increase motivation for activity than just step count, distance, and pace:	Despite these positive feelings, several wearable users expressed a cyclical relationship between using their monitors to help achieve goals and reducing use after goals were met. Users suggested that perhaps the goal setting features are not enough to encourage long-term usage of these devices. The abandonment of devices overtime was recognized by one user who mentioned. Participants in both groups mentioned the cost may not be worth the benefits. Interestingly, five of the users reported that they were gifted or received their device on discount, which may have prevented cost from posing as a barrier to adoption in comparison to non-users.<br><br>Non-wearable users held perceptions that wearables were overly responsive to popular trends, materialistic, and even inequitable as the paramount reason for resisting adoption of the technology:<br><br>They even mentioned that people who wear a device might not necessarily be physically active, but want to identify as someone who uses this type of product Several mentioned that exercise habits cannot be sustained by technology. They did not perceive the devices as promoting a lasting, beneficial relationship with health behaviors.		Overall, wearable users described behavior change following use of the device. Specifically, in terms of health behavior change and sedentary change. Much of this seemed to derive from the notifications and data that led to a new awareness for users' sleep patterns, physical activity, and sedentary time:<br><br>Likewise, non-users recognized the potential of the devices to encourage physical activity and prevent disease:<br><br>Both users and non-users supported the use of devices in the medical field as they would be helpful for providers and patients—if certain aspects were changed, including: cost, appearance, accuracy. This might be the solution to the sustainability for users: Most users felt like the devices created a sense of achievement in their lives by adding to their performance, routines, and overall wellness: Wearable users did mention feelings of codependence as their device elicited added stress and a sense of guilt. The wearable became so profound in some participants' daily routines that their entire day would revolve around meeting their exercise goals. The	In addition, some wearable users were concerned that the devices were not accurate based upon their own experiences. Further, both users and non-users expressed skepticism towards the accuracy of the tracking features based upon their own experience. There was a particular concern with the devices overestimating the amount of activity:	N
AutoDietary: A Wearable Acoustic Sensor System for Food Intake Recognition in Daily Life	2016	IEEE SENSORS JOURNAL	Bi, Y and Lv, MS and Song, C and Xu, WY and Guan, N and Yi, W	10.1109/JSEN.2015.2469095	Nutrition-related diseases are nowadays a main threat to human health and pose great challenges to medical care. A crucial step to solve the problems is to monitor the daily food intake of a person precisely and conveniently. For this purpose, we present AutoDietary, a wearable system to monitor and recognize food intakes in daily life. To evaluate real-life user experience, we conducted a survey, which collects rating from 53 participants on wear comfort and functionalities of AutoDietary.	Quantitative	China	people with overweight or obesity		A Wearable Acoustic Sensor System<br>for Food Intake Recognition in Daily Life			Results show<br>that the current design is acceptable to most of the users.									UNDECLARED
Patterns of Use and Key Predictors for the Use of Wearable Health Care Devices by US Adults: Insights from a National Survey	2020	JOURNAL OF MEDICAL INTERNET RESEARCH	Chandrasekaran, R and Katthula, V and Moustakas, E	10.2196/22443	The main objective of this study was to examine the use of wearable health care devices and the key predictors of wearable use by US adults.	Quantitative	USA	Wearable Health Care Devices	Women (16.41%), White individuals (19.74%), adults aged 18-50 years (19.52%), those with some level of college education or who were college graduates (25.60%), and those with annual household incomes greater than US $75,000 (17.66%) were most likely to report using wearable health care devices. There were no significant differences based on marital status or race/ethnicity.	Wearable Health Care Devices				The logistic regression results revealed significant associations between demographic characteristics and the use of wearable health care devices (Table 2). Individuals aged 50-64 years (OR 0.57), 65-74 years (OR 0.46), or ≥75 years (OR 0.47) were less likely to use wearable health care devices than those aged 18-34 years. Women were 1.26 times more likely to use wearable health care devices than men (OR 1.26, 95% CI 0.96-1.65). As compared to non-Hispanic Asian individuals, White individuals (OR 1.65, 95% CI 0.97-2.79) were more likely to use wearables. Individuals with higher levels of education (ie, some college education; OR 1.06, 95% CI 0.30-3.69) or who were at least college graduates (OR 1.05, 95% CI 0.31-3.51) exhibited greater likelihood to use wearables. Individuals whose annual household income was at least US $75,000 were 2.6 times more likely to use wearables (OR 2.6, 95% CI 1.39-4.86) than those with incomes less than US $20,000.Individuals who felt their general health was better (OR 1.17, 95% CI 0.98-1.39) and those who perceived themselves to be overweight (OR 1.16, 95% CI 1.06-1.27) were more likely to adopt								N
Perspectives on the Gamification of an Interactive Health Technology for Postoperative Rehabilitation of Pediatric Anterior Cruciate Ligament Reconstruction: User-Centered Design Approach	2021	JMIR SERIOUS GAMES	McClincy, M and Seabol, LG and Riffitts, M and Ruh, E and Novak, NE and Wasilko, R and Hamm, ME and Bell, KM	10.2196/27195	iA is a web-based platform that incorporates wearable motion sensors and a mobile app that provides feedback and allows remote monitoring. The Wheel of Sukr is a gamification mechanism that includes numerous behavioral elements.This study aims to use a user-centered design process to incorporate behavioral change strategies derived from self-management theory into iA using the Wheel of Sukr, with the aim of influencing patient behavior.	Qualitative	USA	Young athletes aged 10-18 years with a history of ACL injury and surgical reconstruction	Demographics Values<br>Gender, n (%)<br><br>Male 3 (30)<br><br>Female 7 (70)<br>Age (years), mean (SD) 12.8 (1.32)	an Interactive Health Technology for Postoperative Rehabilitation of Pediatric Anterior Cruciate Ligament Reconstruction			Participant responses were positive when asked about using a mobile app for home exercises. They thought that an instructional component on the app would be helpfu		Overall, all participants liked the device or thought it was cool. Participants also cited convenience as it would be accessible by their phone<br><br>Participants were prompted to list features from their favorite games that they would include in a mobile app for their physical therapy. Sports specificity was commonly cited<br>Other features from the participants' favorite games that they endorsed included competition, notifications or reminders, rewards, and social aspects of gameplay:	Motivation<br>Position on a leaderboard<br>Reward for playing each day<br>Points for completing tasks<br>Leveling up<br>Self-management<br>Seeing daily results<br>Setting goals<br>Socializing<br>Connecting to friends in the game<br>Self-representation<br>Creating own character<br>Fun<br>Challenges within the game<br>Esteem<br>Congratulations<br>Growth<br>Complete goals<br>Increased challenge progression<br>Sustainability	Suggestions for Future Improvement The participants verbalized various suggestions or improvements for the device and/or app related to understanding the exercises, creating goal-motivated incentives to complete exercises, and visual additions or changes. From a usability standpoint, some suggested improved descriptions of the individual exercises, whereas others focused on improving the method of attaching the sensors to their knees. One individual suggested the inclusion of a general surgical recovery guideline:<br><br>The features of gameplay that participants wished to incorporate closely followed the themes from the Wheel of Sukr card sort activity.<br>Suggestions for improvements centered more on mobile apps and games as opposed to sports and may have been influenced by phase 2's focus on these topics. Some participants suggested the incorporation of a sport into the iA app, but none went further to elaborate specific ways to incorporate sports. Participants also did not specifically cite social networking capabilities as essential additions to iA, but they noted this capability to be of interest or importance in general mobile apps or games. Textbox 2 shows the suggested iA improvements separated into their respective Wheel of Sukr categories.			Participants liked that the device would be able to show them how they are moving during their physical therapy exercises<br>Other participants elaborated that it would help them because they would not have to wait until their next physical therapy session to get feedback about their exercises:<br>Some participants noted that they appreciated having the ability of a physical therapist to monitor their progress remotely:<br>they commented positively about the device and app. One parent said that it would help with motivation		MM receives royalties from Elizur, LLC, for a patent related to this manuscript. KMB receives royalties (<US $5000 per year) from the license of iA (University of Pittsburgh Reference #04275) being further developed in this research study.
Why Users Comply with Wearables: The Role of Contextual Self-Efficacy in Behavioral Change	2021	INTERNATIONAL JOURNAL OF HUMAN-COMPUTER INTERACTION	Rieder, A and Eseryel, UY and Lehrer, C and Jung, R	10.1080/10447318.2020.1819669	Wearables provide great opportunities for improving personal health, but research challenges their capacity to evoke behavioral change effectively. Realizing the full potential of wearables requires a better understanding of users' behavior change processes. Based on self-efficacy theory, we investigate how wearables influence users' perceptions of their self-efficacy and subsequent health behavior.	Qualitative	Switzerland	General public	age: 18-63; 9F, 16M; occupation: student, retired, professional, etc.	wearable activity trackers. Leading providers include Apple, Xiaomi, Fitbit, and Garmin (Statista, Citation2019b).				Our data showed that internal and external contexts can influence individuals' self-efficacy negatively or positively. While interviewees referred to certain internal contexts as contributing positively to their self-efficacy and influencing their compliance with the wearable, they identified other internal contexts as reducing their self-efficacy and compliance with the wearable.<br><br>The only external context that the interviewees mentioned as having a positive influence was household chores. Since these chores usually involved physically intense activities, interviewees felt that they facilitated attaining their activity goals. The external context, unlike the internal context, was typically listed as a factor that negatively affected self-efficacy (e.g., bad weather did not allow more steps, their schedule did not allow time to go running). Contexts could be short-term or long-term. Permanent external contexts like chronic medical conditions and permanent internal contexts like strong, ingrained habits imposed special hardship on users in terms of staying committed to the actions the wearable		Our participants reported that wearables provided information on the extent to which they accomplished their goals. Verbal persuasion refers to how verbal encouragement increases a person's self-efficacy and behavioral compliance. Wearables provided many elements of verbal persuasion for our participants. Most common were activity reminders, such as when the wearables say, "Walk another thousand steps to reach your goal today," or "You're on a five-day streak! Keep it up today." Besides activity reminders, verbal persuasion was also conveyed through real-time training parameters, bedtime reminders, or inactivity alerts.<br><br>Vicarious experience refers to how seeing another person do a task or knowing that another person is able to do a task causes individuals to believe that they can do it too. Vicarious experience is manifested mostly through wearables' social features that connect users to each other and allow them to build a community to follow and track each other's performance. Social features also enabled them either to compete against each other or to work jointly toward the same goals (i.e., challenges).				Fourteen of the twenty-five interviewees indicated that the elements related to verbal persuasion helped them feel more capable, increasing their task self-efficacy, and caused them to continue using the wearable and/or to comply with its suggestions. Our interviewees reported that such connections and competition increased their self-efficacy to do as much as or better than the others and to change their behavior to comply with the wearable.		UNDECLARED
There's nothing really they can do with this information': unpacking how users manage privacy boundaries for personal fitness information	2020	INFORMATION COMMUNICATION & SOCIETY	Zimmer, M and Kumar, P and Vitak, J and Liao, YT and Kritikos, KC	10.1080/1369118X.2018.1543442	Fitness trackers are an increasingly popular tool for tracking one's health and physical activity. While research has evaluated how these mobile devices can improve health and well-being, few studies have empirically evaluated users' privacy concerns that stem from the collection, aggregation, and sharing of personal fitness information (PFI). In this paper, we endeavor to gain a more complete picture of users' experiences with fitness trackers and how they manage the privacy of personal fitness information.	Mixed	USA	General public	F (80%)M (20%) Mean (SD) = 41 (12.7)	Fitness trackers					Using a five-point scale ranging from not important at all to very important, respondents said the most important features were the step counter (M = 4.81, SD = 0.53), calorie tracker (M = 4.34, SD = 1.01), and workout/activity tracker (M = 4.02, SD = 1.14), while the least important features were the ability to compete with others (M = 2.90, SD = 1.41) and engage with social features (e.g., messaging, groups, chat) (M = 2.10, SD = 1.16). While some of our participants expressed minor technological frustrations with their fitness tracker – including perceptions of imprecise step counts, skin irritations, lack of waterproofing, battery issues, or that their tracker lacked features available in more current models – few mentioned any significant drawbacks from owning a device. a few complained about the small size of their social network within the app. Others were wary of the shaming and over-competitive aspects of sharing personal fitness information across social networks.					Many participants noted how the devices made them more aware and more accountable regarding their level of activity, particularly when the devices 'nudged' them into action – some, but not all devices come equipped with reminder functions to prompt regular physical activity throughout the day.<br><br>Many fitness trackers collect a variety of fitness data beyond steps, and some interview participants cited sleep tracking as a major benefit of owning these devices.<br><br>Other benefits interviewees expressed included weight loss, increased endurance, feeling healthier, and a sense of making positive life choices. And while survey respondents rated the social aspects of fitness trackers of lowest importance (M = 2.10, SD = 1.16), numerous interview participants said the social functions help motivate them to exercise more.	Most participants expressed only minimal privacy concerns related to their use of a fitness tracker. A few participants admitted they were largely unaware of any broader privacy issues related to using a fitness tracker. Others expressed general ambivalence.<br>Regarding the relative sensitivity of personal fitness information, survey respondents expected that they would feel only average levels of concern (evaluated on a scale of 0–100) if their fitness tracker data were compromised in something like a security breach (M = 54.44, SD = 29.26).<br><br>Numerous interviewees noted, however, that privacy concerns would emerge if their fitness tracker was collecting or sharing personal information beyond just steps taken, such as personal identifiers or location data.<br><br>Other interviewees clearly stated that it would be a privacy concern if the tracker collected specific, GPS-based, location data.	N

Combining actigraphy and experience sampling to assess physical activity and sleep in patients with psychosis: A feasibility study	2023	FRONTIERS IN PSYCHIATRY	Pieters, LE and Deenik, J and de Vet, S and Delespaul, P and van Harten, PN	10.3389/fpsyt.2023.1107812	Sleep disorders and reduced physical activity are common in patients with psychosis and can be related to health-related outcomes such as symptomatology and functioning. Mobile health technologies and wearable sensor methods enable continuous and simultaneous monitoring of physical activity, sleep, and symptoms in one's day-to-day environment. Only a few studies have applied simultaneous assessment of these parameters. Therefore, we aimed to examine the feasibility of the simultaneous monitoring of physical activity, sleep, and symptoms and functioning in psychosis.	Quantitative	Netherlands	patients with psychosis	Age (years), mean (SD) 37.6 (7.8)<br>Sex (male), n (%) 25 (75.8)<br>DSM-5 diagnosis, n (%)<br>Schizophrenia 16 (48.5)<br>Schizoaffective disorder 4 (12.1)<br>Other psychotic disorder* 13 (39.4)<br>Symptom severity<br>BPRS total score (range 28–196), mean (SD) 48.4 (11.7)<br>BPRS positive symptoms (range 7–49), mean (SD) 12.9 (4.5)<br>BPRS negative symptoms (range 7–49), mean (SD) 12.2 (3.1)<br>Employment status, n (%)<br>Employed 5 (15.2)<br>Unemployed 28 (84.9)<br>Living situation, n (%)<br>Alone 16 (48.5)<br>Sheltered housing 7 (21.2)<br>With partner/family 10 (30.3)<br>Functioning<br>GAF symptom score (range 0–100), mean (SD) 48.4 (15.0)<br>GAF handicap score (range 0–100),	Mobile health technologies and wearable sensor methods enable continuous and simultaneous monitoring of physical activity, sleep, and symptoms in one's day-to-day environment.					Answers from the evaluation questionnaire (ranged 1–7) showed that the wrist actigraphy watch was mostly comfortable to wear during day (mean = 5.1, SD = 1.6, range 1–7) and night (mean = 5.1, SD = 1.5, range 2–7) and did not impair daily activities (mean = 1.9, SD = 1.2, range 1–5). In general, patients were not bothered by wearing the actigraphy watch visible to others (mean = 1.6, SD = 0.7, range 1–3). However, participants were moderately motivated to wear the actigraphy device for a longer period (several months; mean = 3.9, SD = 2.1, range 1–7).<br><br>The mean completion rates of the ESM were 64.0% (SD = 17.0, range 30.4–91.1) for the continuous questionnaires, 90.6% (SD = 13.9, range 42.9–100.0) for the morning questionnaires and 82.6% (SD = 20.1, range 28.6–100.0) for the evening questionnaires. The ESM item "this beep disturbed me" was rated with a mean of 2.3 (SD = 1.4, range 1.0–5.9) on a scale from 1 to 7. Answers from the evaluation questionnaires showed that participants did not experience difficulties using the ESM application (mean = 1.8, SD = 1.3, range 1–6) or understanding the ESM questions (mean = 2.0, SD = 1.2, range 1–5). Overall, participants found the number (mean = 3.3, SD = 1.8, range 1–7) and duration (mean = 2.5, SD = 1.3, range 1–5) of beep questionnaires not too burdensome.							N
Parents' views about healthcare professionals having real-time remote access to their young child's diabetes data: Qualitative study	2022	PEDIATRIC DIABETES	Kimbell, B and Rankin, D and Hart, RI and Allen, JM and Boughton, CK and Campbell, F and Frohlich-Reiterer, E and Hofer, SE and Kapellen, TM and Rami-Merhar, B and Schierloh, U and Thankamony, A and Ware, J and Hovorka, R and Lawton, J and KidsAP Consortium	10.1111/pedi.13363	We explored parents' views about healthcare professionals having remote access to their young child's insulin and glucose data during a clinical trial to inform use of data sharing in routine pediatric diabetes care.	Qualitative	Austria, Germany, Luxembourg, and the UK.	parents and young diabetes children		hybrid closed-loop system use with sensor-augmented pump therapy					Despite this, some parents noted having "initially felt a bit funny" (030_CLS) about these individuals being able to access their child's insulin and glucose data, because these data gave insight into their routines and private life more generally. However, all of these parents notes how any initial discomfort about "being watched" had quickly abated:		Indeed, some suggested that, were more regular remote support to be made available to closed-loop system users in routine care, they would favor a model of care whereby regular, scheduled review appointments could be replaced with ad-hoc appointments as and when needed:<br><br>"'Cause…I don't know what we're gonna talk about this afternoon [during review appointment], 'cause there's nothing to say. Like, everything's just going really, really well…So maybe if we just had it on a basis of: 'Oh, we'll have a look, you know, we'll definitely have a look at her data every, you know, couple of months, and if we feel like there's a problem we'll call at that point,' and we don't need like a particular scheduled appointment in." (018_CLS)<br><br>As parents recognized that HCPs working in routine clinical care would have less time to check data than HCPs during the trial, some also endorsed use of specialist software alerting HCPs to potentially problematic events or patterns in the data:<br><br>"I would hope on a closed-loop system there'd be a software that would be looking at what your closed-loop system is reporting back to them,			Better understanding and more tailored advice Parents also noted how these concerns had been substantially outweighed by the benefits gained by their child. Specifically, parents noted how, by virtue of being able to remotely access their child's data to inform pre-scheduled and unplanned study contacts/visits, HCPs had had much better understanding and awareness of activity patterns, everyday life and insulin requirements than was possible in routine clinical care, when they had generally only reviewed "snapshots" (typically the last 1–2 weeks) of data at quarterly review appointments. It was also noted how, because of this enhanced awareness, staff could offer clinical input better tailored to their child's needs and circumstances.Lessened workload Parents also highlighted multiple logistical benefits to remote data access/availability. Some, like this mother, welcomed no longer needing to remove the child's pump to access readings and email or upload these in advance of a consultation. Others observed how HCPs no longer needed to spend time during consultations downloading directly from the child's pump or glucose meter. HCPs' ability to "simply push a button and…look how it has been" (017_CLS), as some	oncerns/caveats about HCPs having continuous data access While most parents reported having no concerns regarding potential data protection or privacy breaches when granting HCPs continuous data access, several emphasized how their consent would be contingent on this being restricted to a select group of trusted individuals:<br>"…because I really trust those people there. I think they are really nice and we get on well. I think if you had a huge team there, it would be different." (013_CLS, father)<br><br>Similarly, some parents described specific scenarios in which they would likely refuse HCP access to their child's data, for example, if the data were stored on an unsafe internet-based platform or shared with third-party organizations, or if HCPs were able to remotely intervene in insulin delivery. Others observed how, while they had few anxieties about granting data access while their child was still young, it would be important to take into consideration the	R.H.reportshavingreceivedspeakerhonorariafromEliLillyandNovo Nordisk,servingonadvisorypanelsforEliLillyandNovoNordisk,and receivinglicensefeesfromBBraunandMedtronic.R.H.alsoreports patents,patentapplications,shareholdingsanddirectorshipat CamDiab.C.K.B.hasreceivedconsultancyfeesfromCamDiab. F.C.hasattendedadvisoryboards,obtainedspeakingfeesandeducationalsupportfromAbbott,Dexcom,Medtronic,Lilly,Insulet,and NovoNordisk.E.F.R.reportshavingreceivedspeakerhonorariafrom EliLillyandNovoNordisk,andservingonadvisoryboardsforEliLilly andSanofi.TheauthorsB.K.,D.R.,R.I.H.,J.M.A.,S.E.H.,T.M.K.,B.R.M.,U.S.,A.T.,J.W.,andJ.L.reportnoconflictsofinterestrelevanttothisarticle.
Adoption of mobile health services using the unified theory of acceptance and use of technology model: Self-efficacy and privacy concerns	2022	FRONTIERS IN PSYCHOLOGY	Liu, YZ and Lu, X and Zhao, G and Li, CJ and Shi, JY	10.3389/fpsyg.2022.944976	Mobile health (mHealth) services have been widely used in medical services and health management through mobile devices and multiple channels, such as smartphones, wearable equipment, healthcare applications (Apps), and medical platforms. However, the number of the users who are currently receiving the mHealth services is small. In China, more than 70% of internet users have never used mHealth services. Such imbalanced situation could be attributed to users' traditional concept of medical treatment, psychological factors (such as low self-efficacy) and privacy concerns. The purpose of this study is to explore the direct and indirect effects of mHealth users' self-efficacy and privacy concerns on their intention to adopt mHealth services, providing guidelines for mHealth service providers to enhance users' intention of adoption.	Quantitative	China	General public	A total of 386 valid questionnaires were retained, with a valid rate of 70.6%. Among the participants in this survey, there were 204 females, accounting for 52.8%, and 182 males, accounting for 47.2%, with a reasonable gender ratio. In terms of education background, the majority of participants were undergraduates, accounting for about 65%, and nearly 20% of participants had a master's degree or above. It can be seen that more than 80% of participants have higher education background	Adoption of mobile health services using the unified theory of acceptance and use of technology model			participants have used mHealth services, and nearly 80% of participants have used mHealth services more than twice, indicating that most of the participants have the experience of using mHealth services. They can better fill out the questionnaire based on their personal experience, thus ensuring the accuracy and reliability of the data. The channels through which the participants mainly use mHealth services include the hospital's official website, WeChat public account and mini programs, accounting for 89.6%. The mHealth services that the participants mainly engage in are making appointment, viewing department and doctor information, and checking about queuing and calling information. In addition, 10.4% of participants used mHealth services before but no longer use it now. The important reasons for participants to stop using mHealth are "No habit of using mHealth services" and "Unguaranteed professionalism and reliability of information,"	A questionnaire was designed by the research team and 386 valid responses were collected from domestic participants in China. Based on the unified theory of acceptance and use of technology (UTAUT) model, a research model integrated self-efficacy and privacy concerns was constructed to investigate their effects on users' intention to adopt mobile mHealth services. The results show that self-efficacy could facilitate users' intention to adopt mHealth services, and had a significantly positive effect on perceived ubiquity, effort expectancy, performance expectancy and subjective norm.								N
Evaluation of flash glucose monitoring after long-term use: A pediatric survey	2019	PRIMARY CARE DIABETES	Vergier, J and Samper, M and Dalla-Vale, F and Ventura, V and Baucher, F and Joubert, F and Pons, C and De Oliveira, M and Salet, R and Faure-Galon, N and Adiceam, P and Baechler-Sadoul, E and Epstein, S and Simonin, G and Reynaud, R	10.1016/j.pcd.2018.08.004	To understand the opinions of children with type 1 diabetes about their everyday use of flash glucose monitoring. (FGM).	Quantitative	France	Children with type 1 diabetes	https://www.sciencedirect.com/science/article/pii/S1751991818301190#tbl0005	Evaluation of flash glucose monitoring after long-term use			Overall, 233 (67.1%) of the participants were satisfied with the system and 39 (11.2%) were dissatisfied. The satisfaction rate was higher for the youngest patients (84.4% for the 0–5 years old vs. 64.6% for the 6–18 years old), but with borderline significance (p = 0.08). The motive for dissatisfaction frequently given on the free-comments page was the fact that the device did not provide real-time alerts.			The main motivation, reported by 85.9% of children, was to avoid the painful finger-pricking associated with SMBG. The next most frequent motivations were to facilitate nocturnal parental glucose control and to make measurements faster (for 60.8% and 50.7% of participants, respectively), while improving glycemic control was an incentive for 22.5% of patients. Finally, 36 participants reported that one of their motivations for taking up FGM was to begin self-monitoring again; 22 (61%) of these patients had an HbA1c level above 8.0%.				The use of FGM led most patients to change their habits: 70.6% reported that they checked their glucose levels more frequently, younger children and those treated with CSII performed significantly more checks than the rest of the cohort (p < 0.05 for both). However, only 37.5% used the directional arrows to adapt insulin dosage, and 37.2% to correct hyperglycemia sooner. Of the 276 participants who had used the sensor for more than 3 months, 97 (35.1%) declared that their HbA1c levels had decreased. Among the 71 patients who wanted to use FGM to improve glycemic control, 41 (57.5%) declared that they had achieved this. Among the 36 respondents who were not performing SMBG before using FGM, 12 (33.3%) reported that their metabolic control had improved (average time of use for these 12 children: 365 days [94–734]). However, there was no significant correlation between the declared number of daily scans or the fact that this number increased after switching to FGM and a reduction in HbA1c levels (p = 0.14 and p = 0.79 respectively).	Various barriers to prolonged use were reported by participants (Table 3, Table 4). The main reason given for interrupting or permanently stopping FGM was the cost of the device. This was reported by 68 children (19.6% of the cohort). Skin irritations ranging from erythema to pruritus and allergies were reported by 22.2% of respondents. The difference in the prevalence of skin reactions between patients treated with MDI (25.5%) and CSII (16.8%) was of borderline significance (p = 0.07). Seven children (2% of the cohort) interrupted or permanently stopped FGM because of cutaneous reactions and 11 more (3.2%) because the sensor tended to fall off. Poor sensor adhesion was reported by 165 (47.6%) patients and 179 (51.6%) used extra adhesives.Twenty-seven children (7.8% of the cohort), of which 23 (85%) were teenagers, interrupted or stopped FGM because they could not tolerate wearing it continuously. The proportions of patients treated with MDI and CSII who gave up for	N
The Motivational Impact of Wearable Healthy Lifestyle Technologies: A Self-determination Perspective on Fitbits With Adolescents	2017	AMERICAN JOURNAL OF HEALTH EDUCATION	Kerner, C and Goodyear, VA	10.1080/19325037.2017.1343161	Considerable numbers of young people are not meeting physical activity guidelines. Wearable fitness devices can provide opportunities for physical activity promotion. The aim of the study was to explore whether wearable healthy lifestyle technologies impacted on adolescents' (13- to 14-year-olds) motivation for physical activity.	Mixed	United Kingdom.	Adolescents	One hundred 13- to 14-year-olds (53 females, 47 males)	Fitbits												Qualitative evidence suggested short-term increases in motivation through feelings of competition, guilt, and internal pressure. Discussion: Findings suggest that healthy lifestyle technology may have negative motivational consequences	UNDECLARED
Wearable fitness tracker use in federally qualified health center patients: strategies to improve the health of all of us using digital health devices	2022	NPJ DIGITAL MEDICINE	Holko, M and Litwin, TR and Munoz, F and Theisz, KI and Salgin, L and Jenks, NP and Holmes, BW and Watson-McGee, P and Winford, E and Sharma, Y	10.1038/s41746-022-00593-x	As the use of connected devices rises, an understanding of how digital health technologies can be used for equitable healthcare across diverse communities is needed.	Qualitative	USA	patients	Of the 1007 adults surveyed, 39% identified as Hispanic, 36% as non-Hispanic Black or African American, and 15% as non-Hispanic White (Fig. 1). Almost three-quarters identified as cis-gender women (71%), 14% had less than a 9th-grade education while 45% had completed high school, and participants were evenly divided across age groups. The surveys were administered in English (68%) and Spanish (32%). The primary outcome was whether participants would like a fitness tracker, and overall 58% responded "yes," 20% "no," and 23% did not answer	Wearable fitness tracker								the following reasons for not currently owning a fitness tracker: "they are not helpful," "I do not know how to use it,"<br>"they are too expensive," "I do not understand how it can help me but want to learn," "I do not have access to the internet," and "I do not want to commit to using it every day."				N
It's Cool to be Healthy! The Effect of Perceived Coolness on the Adoption of Fitness Bands and Health Behaviour	2023	JOURNAL OF INFORMATION AND COMMUNICATION TECHNOLOGY-MALAYSIA	Noor, NM and Zolkepli, IA and Omar, B	10.32890/jict2023.22.1.5	The current study aims to examine the perception of coolness as a higher-order construct with multiple dimensions, namely originality, attractiveness, and sub-cultural appeals, by investigating the direct effect on fitness band adoption and indirect influence on users' health behaviour.	Quantitative	Malaysia	General public	GenderFemaleMale1281524654Age18 – 2526 – 35 36 – 4546 – 6061 and above 341508014212542951EducationPrimary schoolSecondary schoolDiploma or equivalent degreeBachelor's degreeMaster's degreeDoctorate's degree315511544611151855164LocationUrban 8530Suburban 17864Rural 176	Fitness Bands			This study's findings validated the positive impact of fitness band adoption on health behaviour, therefore supporting the second hypothesis (H2).		a significant effect of perceived coolness on fitness band adoption(H1).	The current study discovered coolness as a driving factor with the potential to be harnessed in promoting health awareness and optimal health practice, which supported the hypothesis (H3a).				n this study, the present hypothesis (H3b) was supported by the findings, thus posting that fitness band adoption possessed a significant influence on health behaviour.		UNDECLARED
A Remote Health Coaching, Text-Based Walking Program in Ethnic Minority Primary Care Patients With Overweight and Obesity: Feasibility and Acceptability Pilot Study	2022	JMIR FORMATIVE RESEARCH	Smart, MH and Nabulsi, NA and Gerber, B and Gupta, I and Di Eugenio, B and Ziebart, B and Sharp, LK	10.2196/31989	This study aims to assess the feasibility, acceptability, and preliminary outcomes of an 8-week text-based coaching and Fitbit program aimed at increasing the number of steps in a predominantly overweight ethnic minority population.	Mixed	USA	people with overweight or obesity	participants were women and were aged on average 47 years (SD 9.6 years). A total of 80% (24/30) of participants were African American, and 17% (5/30) were Hispanic or Latino. A total of 20% (6/30) of participants reported a yearly household income <US $20,000, and 37% (11/30) of participants reported high school as their highest level of education. At baseline, the average BMI was 39.3 kg/m2 (SD 9.3 kg/m2) and 77% (23/30) of the participants were considered obese (BMI≥30 kg/m2) [27]. A total of 30% (9/30) of participants self-rated their health as either fair or poor.	A Remote Health Coaching, Text-Based Walking Program						health, increase their physical activity, or lose weight. Some were curious about their daily step count.				participants noted additional behavior changes related to focusing on their step goals. Examples include self-reported weight loss, perceived improvement in chronic health conditions, and overall increased health consciousness.		N
A Wearable Sensor-Based Exercise Biofeedback System: Mixed Methods Evaluation of Formulift	2018	JMIR MHEALTH AND UHEALTH	O'Reilly, MA and Slevin, P and Ward, T and Caulfield, B	10.2196/mhealth.8115	The aim of this study was to assess the Formulift system with three different and realistic types of potential users (beginner gym-goers, experienced gym-goers, and qualified strength and conditioning [S&C] coaches) under a number of categories: (1) usability, (2) functionality, (3) the perceived impact of the system, and (4) the subjective quality of the system. It was also desired to discover suggestions for future improvements to the system.	Mixed	UK	gym-goers and conditioning [S&C] coaches	A total of 15 healthy volunteers participated (12 males, 3 females, age: 23.8 years [standard deviation, SD 1.8], height: 1.79 m [SD 0.07], body mass: 78.4 kg [SD 9.6]).	Formulift is a newly developed mobile health (mHealth) app that connects to a single inertial measurement unit (IMU) worn on the left thigh.			All participants said they "liked" the system or thought it was "good."		The system achieved a mean SUS usability score of 79.2 (SD 8.8). Beginners deemed the system most usable with a score of 86.25 (SD 1.9), whereas the experienced gym-goers and S&C coaches scored the system at 75.5 (SD 9.1) and 74.5 (SD 8.0), respectively.<br><br>For instance, the layout of the app was acknowledged to be very easy to follow with large icons, large buttons and a minimal number of menus being cited as the reasons for this. None of the users reported any difficulties interpreting the language used within the app. The color used within the app was also referred to in a positive manner. It was considered to "make things stand out great" during a session, make the app "attractive," and the color of the repetition number (red, orange, or green) following a set was said to be very useful. Participants also offered positive feedback regarding the "How to wear the sensor" section. They found the instructions were very clear and easy to follow. In addition to this, a participant spoke positively about wearing the IMU.<br><br>Participants reported a number of usability issues. The most reported usability issue related to app navigation, in particular, to going back a step within the app. Four participants, who are usually iPhone users, struggled initially to know how to navigate backwards in the app. Some participants also highlighted the need for more status indicators as a usability issue. Particularly, they highlighted that within the "How to use the App" instructions, there was no on-screen indicator when one reached the last instruction, which meant they did not know that the final instruction had been reached. More importantly, the need for a loading indicator was highlighted		The most popular suggestion for future changes to the system was to add more exercises that can be tracked. Experienced gym-goers and S&C coaches regularly suggested the need for more exercises to be tracked by the system. However, because of their experience and knowledge, they also suggested the types of exercises that would require technique classification and suggested that for many exercises the system would only need to automatically count repetition and sets. There was a general consensus among the experienced gym-goers and S&C coaches that upper body compound exercises (eg, bench press, overhead press, and barbell row) were the additional exercises that should be incorporated to the system, including technique classification. There was also a shared opinion that users should be able to add any exercise they complete to the system to be logged automatically. However, it was suggested that noncompound, secondary exercises may not require technique classification, as they are associated with a lower injury risk. It was also said that Olympic weightlifting moves should not be added to the battery of assessed movements, as they would be too dangerous to learn via an app. Feedback was another prominent subtheme that emerged in the area of future changes. Two key things were suggested recurrently: (1) providing longitudinal feedback or progress over time and (2) more detailed feedback on the completed exercise repetitions. This type of longitudinal feedback reflected what most users of the system would also desire. When participants mentioned this, they were informed of other work from the authors that			The beginners and experienced athletes found the system's feedback useful. The S&C coaches, whose experience and knowledge allowed them to gain more insight to the accuracy of the system, were predominantly content with the system's accuracy. The quantitative analysis of the perceived impact of the system, through the uMARS, demonstrated that the system was very beneficial to gym-goers in heightening their awareness of, advancing their knowledge of, increasing their motivation to, and their likelihood to seek help with "exercising with best possible technique".<br><br>All users reported that using the system would aid their technique while exercising. Beginners often mentioned that the system would enable them to learn proper technique, whereas experienced gym-goers stated that the system would be useful particularly when they lose focus or increase the weight they are lifting, and S&C coaches thought the system would help people correct their technique and avoid injury while exercising.Eight users also suggested that use of the system would have a positive effect on their focus and motivation to exercise with acceptable	There were no subthemes that emerged perceiving negative aspects to system use. However, one experienced gym-goer did suggest that use of the system could distract from focusing on their exercise technique.	N
Qualitative investigation into a wearable system for chronic obstructive pulmonary disease: the stakeholders' perspective	2016	BMJ OPEN	Kayyali, R and Savickas, V and Spruit, MA and Kaimakamis, E and Siva, R and Costello, RW and Chang, J and Pierscionek, B and Davies, N and Vaes, AW and Paradiso, R and Philip, N and Perantoni, E and D'Arcy, S and Raptopoulos, A and Nabhani-Gebara, S	10.1136/bmjopen-2016-011657	To ascertain the stakeholders' views and devise recommendations for further stages of the Wearable Sensing and Smart Cloud Computing for Integrated Care to Chronic Obstructive Pulmonary Disease (COPD) Patients with Co-morbidities (WELCOME) system development. This system aims to create a wearable vest to monitor physiological signals for patients concerned incorporating an inhaler adherence monitoring, weight, temperature, blood pressure and glucose metres, and a mobile health application for communication with healthcare professionals (HCPs).	Qualitative	Greece,UK, Ireland and the Netherlands	people chronic obstructive pulmonary disease	Demographicsofthepatientsfromfourcountrieswhotookpartinthefocusgroups Nationality Numberof patients SexAgegroupComorbidities MF51–6061–7071–8081–90CHFT2DM AsthmaHTNDep.Anx.Ins. British107332413233521 Greek76113303202321 Dutch81762001110010 Irish73422211251114 Total 32 1715129928796966 Percentageof total NA53.146.937.528.128.16.325.021.928.118.828.118.818.8 Anx.,anxiety;CHF,chronicheartfailure;Dep.,depression;F,female;HTN,hypertension;Ins.,insomnia;M,male;NA,notapplicable;T2DM, type2diabetesmellitus.	a wearable system for chronic obstructive pulmonary disease			MostDutchpatientsweresceptical abouttelehealth,had mixedviewsabouttheWELCOMEprojectorthe inhalermonitoringdeviceandwerecautiousaboutthe vest(table3).Afewcarerswerealsomorecautiousas theythoughtthesystemwouldtargetpeoplefromspecificagegroupsthatweretechnology-naive. Othercohortsofpatients/carerswerereceptivetotelehealthandthoughtitwascreativeandgaveafeelingof safety.Theideaofthevestwaswellreceived.		Most respondents disagreed with continuous wear,as it may be too stressful for the patient. Thedevelopmentofmale/femaleversionsofthevest wasfavouredbyUKandGreekpatients		Arangeoffunctionstobeincluded,asparttothe mobilephoneapplication,wereproposedbyHCPs, patientsandcarers(figure6).Patients/carersandHCPs fromGreeceandtheUKfeltthatitshouldincorporate educationalvideosfromreputablesourcespersonalised dependingonpatients'comorbidities. Patients/carersfromallcountrieswereinterestedin thediet/nutritionfunctionoftheapplication.Allbut theDutchpatientsalsowishedforthephysicalactivity andstressmanagementfunctionswhereasthemood andsocialnetworkfunctionswerepositivelyappraised bypatients/carersfromtwocountrieseach,namelythe UK/IrelandandtheNetherlands/Ireland,respectively. UKandGreekpatients,andIrishpatients/carers wantedanadditionalfeaturetoenablequestionand answersessionswithHCPs,forexample,GPs.TheGreek andDutchpatients/carersproposedreminderanddiary functionsthatcorrelatedwiththeproposalsfromHCPs. TheGreekcarerssuggestedanintegratedaccelerometer toautomaticallyrecordthepatient'sphysicalactivity. Themajorityoffunctionswerewelcomedby HCPs fromallcountrieswhowishedtobeinvolveddepending ontheirspecialty.Clinicalguidelinesshouldbeused andextracautionexercisedwhendevelopingcertain			WELCOMEwasseenasasystemthatcouldhelp patientsdecidewhentoseekmedicalassistance,thus reducingthenecessityofpatientshavingtotraveltoand fromthedoctors.	However,Dutchpatientshadconcerns aboutbeingconstantlymonitored. Themajorityofnon-Dutchrespondentsfeltthatthe inhalermonitoringdevicewouldbehelpfulinconfirmingcorrectuse.TheDutchpostulatedthatsuchadevice mightbea 'superfluousaddition' andmayonlyb helpfulatthestartoftreatment.Themobileapplication waswelcomedbyallbutUKpatients.	N
Ambient Intelligence from Senior Citizens' Perspectives: Understanding Privacy Concerns, Technology Acceptance, and Expectations	2015	AMBIENT INTELLIGENCE, AMI 2015	Kirchbuchner, F and Grosse-Puppendahl, T and Hastall, MR and Distler, M and Kuijper, A	10.1007/978-3-319-26005-1_4	Especially for seniors, Ambient Intelligence can provide assistance in daily living and emergency situations, for example by automatically recognizing critical situations. The use of such systems may involve trade-offs with regard to privacy, social stigmatization, and changes of the well-known living environment. This raises the question of how older adults perceive restrictions of privacy, accept technology, and which requirements are placed on Ambient Intelligent systems. In order to better understand the related concerns and expectations, we surveyed 60 senior citizens.	Qualitative	Unspecified	Older adults		Ambient Intelligence			The comparison between different features indicates that safety functionalities (emergency detection and burglary detection) are perceived as considerably more useful than disease detection and living comfort features. Fall prevention and energy savings features fell in between (see Table 4 for details). Particularly for burglary detection extra costs are accepted, while the acceptance is much less for living comfort features and disease detection functionality. The comfort functionality is also rated as significantly less likely to be used than the burglary detection feature.Interestingly, no differences emerged between these features for five items relating to the recording of information. The only difference in this regard is that respondents are more likely to accept the recording of information while being in the living room or in the kitchen if the system serves for emergency detection in contrast to living comfort features. Despite, several differences between features were found regarding the transmission of information to external recipients. This was considered least acceptable for energy-saving functionality, but very acceptable for emergency and fall prevention functionality.The wearable one-button emergency call system consistently elicited the lowest levels of concern, was perceived as the most useful, and respondents could also most likely imagine themselves using this system. The sensor-based indoor floor emergency detection system scored second on most dimensions. The camera-based behavior and emergency detection system, in contrast, was perceived as significantly more worrisome and less useful than the other two systems. The combined comprehensive emergency detection solution		Ease of use, particularly in case of emergency, emerged as the most important feature, and the safety of data as the second important priority. Places three to five referred to the affordability of the system (low running and maintenance costs, energy-saving design, and affordability of the system). The lowest priority was given to a constant visibility of the system and a high number of features. No differences were found between senior citizens with and without previous experience with assistive technologies. Ease of use, however, was significantly more important for female than for male respondents.	The necessity to change one's habits was deemed least acceptable, followed by the functionality of the system to identify which and how many persons are currently in the apartment (see Table 2). Whether other people could notice the use of assistive technologies was considered as least problematic. No significant differences were found between male and female respondents or between senior citizens with experience in using assistive technologies and those with no experience.					The greatest fear associated with the use of Ambient Intelligence technologies was the concern that criminals might misuse the collected data, followed by the fear that the system would not be sufficiently reliable (see Table 3). Moderate levels of fears were related to the possible transmission of inaccurate data, the monitoring of social interactions, the continuous monitoring, and the transmission of information to the wrong persons. The lowest causes for concern were the possibility that using the system would be too demanding or that its use would lead to a loss of independence. While no significant differences were found between male and female senior citizens in this regard, inexperienced respondents reported greater levels of concern than experienced senior citizens in four cases (fears of an unreliable system, of transmitting inaccurate information, of social interactions being monitored, and of constantly being monitored). This also resulted in a significantly greater overall fear score for this	UNDECLARED

What drives the use of wearable healthcare devices? A cross-country comparison between the US and Korea	2022	DIGITAL HEALTH	Rha, JY and Nam, Y and Ahn, SY and Kim, J and Chang, Y and Jang, J and Kurita, K and Park, JY and Eom, K and Moon, H and Jung, MH and Kim, YJ and Hwang, JE and Choo, H	10.1177/20552076221120319	Given the rapid growth of the wearable healthcare device market, we examined the associations among health-related and technology-related characteristics of using wearable healthcare devices and demonstrated how the associations differ between the US and Korean users.	Quantitative	USA and Korea	General public	Covariates Gender 0.03 Female 2075 (50.6%) 1539 (50.7%) 536 (50.4%) Male 2023 (49.4%) 1496 (49.3%) 527 (49.6%) Age 0.49 20–29 years old 827 (20.1%) 616 (20.2%) 211 (19.8%) 30–39 years old 818 (19.9%) 609 (20.0%) 209 (19.7%) 40–49 years old 815 (19.8%) 608 (19.9%) 207 (19.5%) 50–59 years old 826 (20.1%) 606 (19.9%) 220 (20.7%) 60 years old or over 825 (20.1%) 609 (20.0%) 216 (20.3%) Annual household income 61.50*** $0–$34,999 1257 (30.7%) 915 (30.0%) 342 (32.2%) $35,000–$74,999 1487 (36.3%) 1021 (33.5%) 466 (43.8%) $75,000 or more 1367 (33.4%) 1112	wearable healthcare devices			Younger, higher earner, and individuals with a child were more likely to use wearable healthcare devices. However, for Korean users, several associations disappeared including health information seeking, hedonic and social innovativeness, age, and household income.		The health-related characteristics that were significantly associated with using wearable healthcare devices included disease-related worries (β = 0.11**), health information seeking (β = 0.26***), physical activity (β = 0.62***), and health-related expenditures ($50–$199, β = 0.38***; $200 or more, β = 0.56***). Hedonic (β = 0.33***), social (β = 0.31***), and cognitive innovativeness (β = 0.14*) also exhibited positive relationships. Key drivers of using wearable healthcare devices include greater concern about a specific illness, active engagement in health-promoting behaviors, and hedonic and social motivation to adopt new technologies. However, more country-specific considerations are needed in future studies to identify the main benefits for target markets.						N
Understanding 'passivity' in digital health through imaginaries and experiences of coronavirus disease 2019 contact tracing apps	2022	BIG DATA & SOCIETY	Costa, A and Milne, R	10.1177/20539517221091138	Growing interest is being directed to the health applications of so-called 'passive data' collected through wearables and sensors without active input by users. To explore how these tensions are negotiated in practice, we present and discuss findings from an interview study with 30 members of the public in the UK and Italy, which examined their views and experiences of the coronavirus disease 2019 contact tracing apps as a large-scale, high-impact example of digital health technology using passive data.	Qualitative	UK and Italy	General public		coronavirus disease 2019 contact tracing apps		Overall, participants recognised the value of passive monitoring to help detect health risks.							Participants who had downloaded the app, or said they would, justified their decision by comparing it to other ways they were acting to manage the risk of infection, such as respecting social distance, wearing face coverings and paying attention to hands hygiene.	The participant commented that the social value of the app provided a strong justification for the potential exposure of her personal life, pre-empting concerns about privacy or the uncertain benefits for herself and her family	N
Direct Adherence Measurement Using an Ingestible Sensor Compared With Self-Reporting in High-Risk Cardiovascular Disease Patients Who Knew They Were Being Measured: A Prospective Intervention	2017	JMIR MHEALTH AND UHEALTH	Thompson, D and Mackay, T and Matthews, M and Edwards, J and Peters, N and Connolly, SB	10.2196/mhealth.6998	The objective of our study was to test a system comprising a biodegradable, ingestible sensor for direct measurement of medication ingestion in a group of patients at elevated cardiovascular risk attending a cardiac prevention and rehabilitation program.	Quantitative	UK	Cardiovascular Disease Patients	Characteristic Mean (SD) or n (%) Age in years, mean (SD) 62 (12) Male, n (%) 15 (71) Body mass index in kg/m2, mean (SD) 30.7 (5.0) Race/ethnicity, n (%) White 15 (71) Other 4 (19) Asian 1 (5) Black 1 (5) Diagnosis, n (%) Primary prevention 14 (67) Secondary prevention 7 (33) Cardiovascular drugs prescribed per patients, n (%) 0 2 (10) 1 3 (14) 2 9 (43) 3 3 (14) 4 3 (14) 5 1 (5)	an Ingestible Sensor in High-Risk Cardiovascular Disease Patients		approximately two-thirds responding that they would continue to use the system in the future, although one-third indicated that they would require amendments before doing so		A total of 8 (38%) patients agreed or strongly agreed with the statement that "the patch was comfortable," while 5 (24%) were neutral and a minority disagreed (Table 2). The majority of the patients 14 (67%) found the system easy to use.					elt that they were less likely to miss doses using the system	A single participant experienced an adverse event due to patch-related contact dermatitis. Overall, 11 (52%) f	N
Pain self-management plus activity tracking and nurse-led support in adults with chronic low back pain: feasibility and acceptability of the problem-solving pain to enhance living well (PROPEL) intervention	2023	BMC NURSING	Xu, WL and Zhang, YM and Wang, ZQ and Dorsey, SG and Starkweather, A and Kim, K	10.1186/s12912-023-01365-y	This study aimed to assess the feasibility and acceptability of a pain self-management intervention, Problem-Solving Pain to Enhance Living Well, which employs wearable activity tracking technology and nurse consultations for people with chronic low back pain.	Quantitative	UK	adults with chronic low back pain	[illegible] demographic and clinical characteristics are presented in Table 1. The participants were predominantly female (62.5%), white (67.5%), non-Hispanic or Latino (87.5%), never married (67.5%), and had college or undergraduate education (67.5%). The average age was 29.8 (SD = 11.7) years, and the average BMI was 26.8 (SD = 7.0). Individuals with less than 150 min of moderate physical activity were eligible for this study, and 62.5% of the participants reported engaging in some form of physical activity 1–3 days per week. Approximately 45% of the participants had low back pain for 1–5 years, and 55% reported pain frequency on at least half of the days over the past six months. Nearly 10% of the participants used opioid analgesics, and 32.5% used	Pain self-management plus activity tracking and nurse-led support in adults with chronic low back pain		Satisfaction with the overall quality of the videos (audio, pace, and organization) was rated as satisfied or very satisfied by 92.5% of the sample.		Among the 10 videos, guided imagery and stretching were rated as the most favorable.				With sufficient and understandable nurse consultation, participants could utilize self-management strategies to manage their pain over the course of this study. Satisfaction with nurse consultations was rated as satisfied to very satisfied by 89.6% of the sample, who reported that nurses were willing to listen to them, and participants were satisfied with respectful, sufficient, and understandable information. Participants reported that the nurse consultations provided a better understanding of decision-making (89.3% were satisfied to very satisfied) and pain self-management (92.9% were satisfied to very satisfied).	Participants found that the video modules were beneficial in providing information on their pain self-management (86.2%) and motivated them to engage in better pain self-management efforts (82.7%). Thirty participants (75%) completed six nurse consultation sessions, with consultation durations ranging from 8 to 20 min. Of the total sample, 90% successfully provided goals to better manage their cLBP symptoms and learned problem-solving skills with nurses during the consultation, including aerobic and resistant physical activities, symptom management, medication management, diet/weight control, and stress management.		N
Toward a Behavior Theory-Informed and User-Centered Mobile App for Parents to Prevent Infant Falls: Development and Usability Study	2021	JMIR PEDIATRICS AND PARENTING	Cooray, N and Sun, SL and Ho, C and Adams, S and Keay, L and Nassar, N and Brown, J	10.2196/29731	This study aims to develop a behavior theory- and evidence-based as well as user-centered digital intervention as a mobile app for parents to prevent infant falls following agile development practices.	Qualitative	Australia	parents and infants	aged 26-35 years, 14 (70%) were nulliparous (70%), 10 (50%) were Australian-born, 12 (60%) were in de facto relationships, 13 (65%) were employed full time, and 15 (75%) were living in apartment buildings. Of the 20 participants, 16 (80%) had attained either a university or Technical and Further Education graduate degree or a postgraduate degree and 9 (45%) had high household income (earning more than Aus $150,000 [US $109,500]; Table	User-Centered Mobile App for Parents to Prevent Infant Falls		Overall, the participants reported that they found the information useful and easy to understand.		The participants particularly liked that the intervention targeted social and environmental aspects. Some participants felt that the information is targeted more toward new mothers with 1 baby and pointed out the importance of information being suitable for the broader audience. In addition, they pointed out some information that they believed may not be practical and requested didactic information. In general, the participants liked the concept of the app but felt that the delivery of information could be more graphical. They commonly liked the self-tracking actions and the idea of receiving reminders and felt that this made the intervention more app-like. However, some were confused with the expected use of tracking, that is, as a checklist rather than using it while attending to the baby		The participants commonly requested additional information related to childcare, which was beyond the scope of the intervention, and some felt that the app scope may be too narrow. They also expressed the importance of the delivery channel if they are to use such an app. Mixed feedback was received about the chat feature, with some having concerns about moderation, bullying, and unsound advice being provided on social media platforms. Others felt that it might be a good place to open up about issues that they cannot raise with their immediate family and requested a professional moderator for the chat. Overall, it was clear that they saw this feature as a place to raise all infant-related questions rather than questions relevant to the intervention					N
A Study on Technology Acceptance of Digital Healthcare among Older Korean Adults Using Extended Tam (Extended Technology Acceptance Model)	2023	ADMINISTRATIVE SCIENCES	Zin, KSLT and Kim, S and Kim, HS and Feyissa, IF	10.3390/admsci13020042	The purpose of this study is to investigate the acceptability of digital health wearable technology in healthcare by the Korean older adults and their attitude towards the use of smart health watches by using an extended Technology Acceptance Model while considering the context of the COVID-19 pandemic.	Quantitative	Korea	Older adults	Over half of the respondents (52.4%) are between the ages of 66–75, and 32.4% of the respondents are aged 56–65 years old, followed by respondents aged 76 to 85 years old (14.7%), while those aged 86–95 years old represented 0.6% of the total sample. As for the occupation of the respondents, both office workers and professionals each represented (10.6%) of the total respondents, whereas 15.3% and 5.3% of the sample were self-employed and public officials, respectively. The majority of the respondents (34.7%) were living in a two-person household, 32.4% in household living four person households or above, 25.9% in three persons households, followed by those (7.1%) living in one person households. As for the income level, 27.6% of respondents have monthly income of 1–2 million won, 23.5% higher than 3 million won, 21.2% with 2–3 million won, followed by 20% with monthly income	smartwatch		Four variables (perceived usefulness, perceived ease of use, social influence, and facilitating condition) predict 67.6% of the attitude (R2 = 0.676), which predicts 51.3% of the behavior intention (R2= 0.513). The relationship between variables was tested by using path coefficient (β) and t statistics. Both the perceived usefulness and the perceived ease of use demonstrated the positive significant effect on attitude toward the use of a smart healthcare watch (H1: β = 0.425, p < 0.05; H2: β = 0.204, p < 0.001). Meanwhile, social influence had no significant effect on attitude towards the use of a healthcare smart watch (β = 0.095, p > 0.05) and thus H3 was rejected. Facilitating condition had a significant positive impact on the attitude towards use (β = 0.204, p < 0.05), thus H4 was supported. And lastly, the attitude toward telehealth had a significantly positive influence on the intention to use telehealth (β = 0.716, p < 0.001).									N
Experiences of using a wearable camera to record activity, participation and health-related behaviours: Qualitative reflections of using the Sensecam	2016	DIGITAL HEALTH	Wilson, G and Jones, D and Schofield, P and Martin, DJ	10.1177/2055207616682628	Upcoming technology is changing the way that we are able to collect data looking into activity, social participation and health behaviours. Wearable cameras are one form of technology that allows us to automatically record a collection of passive images, building a visual diary of the user's day. Whilst acknowledging the usefulness of wearable cameras in research, it is also important to understand individuals' experiences whilst using them. The aim of this study was to explore the acceptance, experience and usability of a wearable camera (Microsoft (R) Sensecam) to record the day-to-day activity and social participation of older people.	Qualitative	UK	Older adults	A purposive sample of 18 participants took part in this study (12 females, 6 males; aged 52–81 years).	using a wearable camera to record activity, participation and health-related behaviours	None of the 18 participants had any previous knowledge or experience of wearable cameras prior to taking part in this study.			Individuals' expectations and experiences of using the wearable camera differed considerably. Participants believed that the wearable camera would be intrusive, difficult to use and would evoke public reaction; however, these worries were not borne out in experience. Individuals typically forgot about the presence of the wearable camera during use, remembering it only sporadically. Design issues of the Sensecam were also a problem for the older adults in the study and affected their interaction with the technology.						One drawback to its use is that some participants were cautious of using the camera when around others, which impacted the amount of time the camera was worn, and, therefore, the nature of the data recorded.	N
Safety First? Users' Perception of Wearable Sensor Networks for Aging	2020	MOBILE NETWORKS & APPLICATIONS	Gaggi, O and Kolasinska, A and Palazzi, CE and Quadrio, G	10.1007/s11036-019-01234-6	Population aging is considered to be one of the most significant social transformations of the twenty-first century. In this paper, we evaluate the acceptability and usability of a system designed to silently monitor the activity and condition of older adults, with a particular focus on the elderly affected by cognitive decline. The system is composed of sensors that can be hidden in various wearable objects to collect data about a user and his/her surrounding environment and transmit them to a server for further elaboration. The aim is to improve safety and independence of older adults.	Quantitative	Italy	elderly people, their relatives	Elderly Relatives Medical Staff Participants 44 51 51 Males 25% 29,4% 31,4% Females 75% 70,6% 68,6% Age range 60 to 97 24 to 67 26 to 64 AVG age 76 44 40 SD age 8,53 6,69 11,73	Wearable Sensor Networks for Aging		group: 70% of the elderly respondents evaluate the proposed technology as highly useful, whereas 82% of health professionals admitted it would be relatively (35%) or very useful (47%) for the family members or caregivers of older adults and patients. A subjective estimation of the utility of the system for their own purposes as healthcare workers is reported in Fig. 5. The majority of the relatives consider WSNs as highly useful both for their loved ones requiring assistance (73%) and for themselves as caregivers (67%, see Fig. 6). Most importantly, 91% of older adults responded that if they had a chance, they would use the proposed devices and 89% agreed to wear them every day.		both the elderly and relatives believe that sensors sewn into the clothes would be the most practical solution. For the health practitioners, on the other hand, inserting the sensors inside a wristwatch appears to be even more functional. For the majority of participants (80%) the size of an item is the most important characteristic. Other features to be considered are color (indicated by 46% of participants) and shape (32%) of the device (Fig. 4).					The elderly, asked to evaluate predicted changes in their sense of safety due to the usage of our technology, admitted that it would increase significantly (75%).	Only 4% of the respondents do not expect to feel more secure as a result of applying the WSN to their everyday lives (see Fig. 7).	UNDECLARED
StraightenUp plus : Monitoring of Posture during Daily Activities for Older Persons Using Wearable Sensors	2018	SENSORS	Cajamarca, G and Rodriguez, I and Herskovic, V and Campos, M and Riofrio, JC	10.3390/s18103409	Monitoring the posture of older persons using portable sensors while they carry out daily activities can facilitate the process of generating indicators with which to evaluate their health and quality of life. The majority of current research into such sensors focuses primarily on their functionality and accuracy, and minimal effort is dedicated to understanding the experience of older persons who interact with the devices. This study proposes a wearable device to identify the bodily postures of older persons, while also looking into the perceptions of the users.	Mixed	Chile	Older adults	Our participants were 30 older persons (15 women and 15 men) aged between 60 and 83 years (average age: 77.8; standard deviation: 6.13) and living in a residential home. Regarding their digital skills, 28 participants have none and 2 have high or medium skill levels. The inclusion criteria were as follows: over 60 years old, and an absence of moderate or severe cognitive problems. According to the FEFA scale, on a scale from 0 to 55, participants had an average score of 24.3, with a minimum score of 15 and a maximum score of 38, denoting some functional deterioration.	Wearable Sensors				participants, and greater than or equal to 1.5 for 18 participants (60%), i.e., general user experience of StraightenUp+ was classified as good. The highest scoring characteristic was the attractiveness quality which scored 2.11, followed by hedonic identity with a score of 1.87. Then came the pragmatic quality characteristic, which scored 1.24. Finally, the lowest scoring quality was hedonic identify stimulation with a score of 1.05. The average values recorded are shown in Figure 5. Consequently, users view the device as having a good appearance, they are able to identify with it, and they find it both stimulating and motivating. Participants classified the device in a highly positive way in terms of prospective use (average scores for attractive, manageable, presentable, and creative were 2.3, 2.6, 2.8, and 2.6, respectively). However, the technical-human concept was classified as primarily a technical one (−2.3 average score), and likewise the demanding-non-demanding concept received negative scores (−0.8 average score), i.e., StraightenUp+ was classified as a non-demanding device, the use of which requires no prior knowledge.Perception of device: Twenty-eight of the participants felt that StraightenUp is comfortable, and two felt that it was uncomfortable. One participant indicated the need to include instructions or indicators to improve user experience in putting it on and taking it off. "You could improve it to make it so that I can't make a mistake, something that prevents mistakes when putting it on" (P18).	Sixteen participants would like to use StraightenUp+ permanently to gain greater understanding of the system and to become more accustomed to how it works. "I've never seen anything like it, it helps you to ask yourself things and then they become second nature… I've never used it, I asked how I was going to get around with this thing on, if I used it at all that is, everything you get used to using… I have got used to using so many things over the years but I never thought I'd use this, but if it can help you, you should use it" (P23). Ten participants would use it when required or when they felt pain or had a health problem.	urthermore, three participants commented that the device should be used under the clothes rather than over them. "It should be used under your clothes because nobody would notice, nobody would criticise me, nobody would be pointing out that I use it or not" (P4).	Finally, four participants were not interested in using the device at all, or unsure about wanting to use it. "I wouldn't use it, for one. I'm not used to it, and second, it would mean being constantly concerned with walking perfectly, like a machine" (P1). F		Participants valued the device as an article that can have a positive health impact. Five participants stated that it could prevent them from adopting bad postures, three participants valued that it could help them understand or diagnose their posture, and two participants that it could improve their health. One participant views the device as a support tool.		N
Use of wearable sensors for pregnancy health and environmental monitoring: Descriptive findings from the perspective of patients and providers	2019	DIGITAL HEALTH	Runkle, J and Sugg, M and Boase, D and Galvin, SL and Coulson, CC	10.1177/2055207619828220	The purpose of this study was to examine the perceptions of pregnant women and their providers at a rural health clinic on the use of wearable technology to monitor health and environmental exposures during pregnancy.	Quantitative	USA	pregnant women	completed the patient survey and 28 health care providers (n=13 OB/GYN and n=15 family medicine) completed the provider survey. Tables 1 and and22 show the demographic characteristics of pregnant patients and providers who responded to the survey. The mean age of patients was 27 years (standard deviation (SD) 6.4) and 86% were white (n=89). About half of the patient sample (n=51) had a high school degree and 31% (n=32) had a college or professional degree. Roughly one in four patients were either single or in an unmarried relationship. Most patients spoke English as their primary language (n=99, 96%) and only 6% of the sample (n=6) identified as Hispanic/Latina origin.	Use of wearable sensors for pregnancy health and environmental monitoring:	Roughly half of all pregnant respondents reported having downloaded a pregnancy-related health app (n=48, 47% yes versus n=55, 53% no).	Roughly half of participants (n=50, 49%) answered "yes" when asked if they would consider wearing a mobile sensor during pregnancy and 45% of respondents (n=46) would consider wearing a sensor embedded in their maternity clothes to monitor health indicators while pregnant.Duration of monitoring Patient response to the question "How long would you be willing to wear a GPS tracker while pregnant?" were mixed, but 22% of women (n=23) reported a willingness to wear a GPS tracker for the entire pregnancy.			When patients were asked which of the following scenarios they would consider wearing sensors to monitor health and the environment, they ranked pregnancy health (n=79, 76%) first followed by chronic disease management (n=73, 71%), personal health and diet monitoring (n=69, 67%), fitness tracking (n=69, 67%), and workplace health and safety (n=59, 57%).An overwhelming majority of patients responded with a willingness (n=78, 75%) to change their behavior during pregnancy in response to receiving personalized recommendations (based on height, weight, physiological monitoring) from their smartphone.					A total of 42% of patients (n=43) reported "no" when asked if they had privacy concerns about data stored on their smartphone or personal monitoring device. Roughly three out of 10 patient respondents went on to report that they would have privacy concerns about data stored on their smartphone or personal monitoring device being moved to a companion website or smartphone app. A large majority of pregnant women in this sample (n=95, 93%) would feel comfortable sharing information from personal monitoring devices with their doctor. Most women (n=91, 88%) believed that this type of information would improve their health and decrease the risk of disease, injury, or an adverse pregnancy health event.	N
"I Must Try Harder": Design Implications for Mobile Apps and Wearables Contributing to Self-Efficacy of Patients With Chronic Conditions	2019	FRONTIERS IN PSYCHOLOGY	Wulfovich, S and Fiordelli, M and Rivas, H and Concepcion, W and Wac, K	10.3389/fpsyg.2019.02388	By presenting results from an explorative study, this paper aims to identify implications for the design of mobile apps and wearables to effectively support patients' efforts in self-management of health with a special emphasis on support for self-efficacy of activities contributing to health.	Mixed	USA	Patients With Chronic Conditions	age: 18-65; 7M, 13f; Student, professional class, etc.	Mobile Apps and Wearables Contributing to Self-Efficacy of Patients With Chronic Conditions				The issue of preferring an input of long text or manual data on laptop (larger screen) is still present. Overall, the application interface in terms of application/wearable interaction was mentioned very often, yet not in negative terms of bad interface design, but in positive terms of application/wearables notifications keeping the user informed and content. Notifications provided by applications where discussed much more often than the application interface itself. Wearables' notifications are there to fit in the routine and unobtrusively support the user's goal matching along a day. For non-routine days, e.g., with less physical activity – the lack of notifications from wearable will be the first indicator of "bad" day in terms of matching the user's goal: "If I do not get notifications along my afternoon, I know I am in trouble" (S12). Additionally, notifications had to be found to be informative. Notifications that did not serve any purpose resulted in participants abandoning the device (e.g., Fitbit) or turning off the notifications completely from these specific applications. An example of a helpful notification was, e.g., "Each 1000 steps I get reminded to drink water" (S10). In our study, some individuals complained about applications not providing expected services or about the poor performance of their underlying network that influenced the provision of these services. A participant said, the "Internet can be touchy" (S7), to express their concern with the underlying network connectivity. Additionally, the participants complained about the lack or erroneous apps sync, low accuracy of Chinese input recognition, or inaccurate GPS. The lack of constant reliability can be a source of irritation: "When and app doesn't work as it should, my blood pressure skyrockets with the frustration" (S5). Ultimately, participants expressed that if an application is constantly malfunctioning or not refreshing content, they will stop using the application all together.Battery efficiency is	Overall, the experience of using smartphone, apps and wearables was reported as a positive one, as even one of the patients admitted – smartphone apps "Keep me sane" (S5). Along this line, there are multitude of factors influencing the nuances of the user's experience with mobile apps and wearables. We have used 100+ expressions from our 20 patient's weekly DRM interviews, additional 180 patients' interviews in the clinic. Figure 3 represents all the expressions used by study participants. We have grouped these words into clusters by using affinity clustering method, which we then labeled along the identified factor. The coding and grouping of words into clusters have been done by two independent coders, and their measure of agreement was 87%. The most disagreements were related to person's routines and lifestyle choices, because sometimes it was about framing of e.g., notifications (interface design) or charging patterns (the battery aspect) fitting into the lifestyle choices and user routines.	Self-Efficacy This factor has not been mentioned in 2012 paper (Ickin et al., 2012) and constitutes new finding, and since is related to the core aim of this paper is presented in a separate section and in great detail. Self-efficacy was a frequent theme of conversation with the participants; discussing their belief that they can manage their own physical activities, nutrition, sleep, medication adherence and other activities contributing to their health self-management with (or without) help of the mobile apps and wearables. There are four main sources of self-efficacy: (i) own past experiences, (ii) experiences of people similar to ourselves, (iii) verbal encouragement from others, and (iv) intrinsic state (Bandura, 1977, 1997). We analyzed the participant data for these factors and present the design implications for mobile apps and wearables contributing or hindering self-efficacy. Past Experiences Wearables and mobile device applications are designed to allow users to easily visualize trends. It allows users to see their previous successes as well as previous failures. Although wearables and apps enable users to see the trends, the target levels are predefined. For example, the target step levels are usually at 10,000 steps and some participants admitted that even though that set target was too high for them and they had never been able to achieve this predefined goal, they were not "tech savvy" enough to know how to change the goal. The self-defined	Some participants (n = 10) do not use technologies for self-management of health and admit it, ranging from asking for advice on how it can be used (e.g., they never heard of Fitbit) to denial of its use for their own health. The input for these participants we got is that "Privacy is an issue," "It's complicated," "I do not know how to use it," and "I am not a techie." These types of statements apply equally to older male and female patients.				N
Body-Worn Sensors for Parkinson's disease: A qualitative approach with patients and healthcare professionals	2022	PLOS ONE	Virbel-Fleischman, C and Retory, Y and Hardy, S and Huiban, C and Corvol, JC and Grabli, D	10.1371/journal.pone.0265438	Body-Worn Sensors (BWS) provide reliable objective and continuous assessment of Parkinson's disease (PD) motor symptoms, but their implementation in clinical routine has not yet become widespread. Users' perceptions of BWS have not been explored. This study intended to evaluate the usability, user experience (UX), patients' perceptions of BWS, and health professionals' (HP) opinions on BWS monitoring.	Mixed	France	people with parkinson disease	Patients' characteristics in each group. Group BWS a (n = 7) BWS b (n = 8) BWS c (n = 7) All (n = 22) Gender ratio (male/female) 2/5 6/2 1/6 9/13 Age (years) median [range] 71 [63; 79] 59 [41; 71] 68 [64; 75] 65,5 [41; 79] Disease duration (years) median [range] 1 [1; 17] 8,5 [2; 17] 10 [1.5; 14] 7,5 [1; 17] Number of techies* 5 7 5 17 Living alone 0 2 0 2	Body-Worn Sensors for Parkinson's disease	Before this study, 20 out of the 22 patients had not heard of BWS.	All 22 patients expressed interest in BWS monitoring. All patients said the device would not be a problem in their daily life before and after the use ("normal", "trouble-free", "without any issue", the device was "simple", "easy"). The physical characteristics and handling of BWS were trivialized ("aesthetically simple", "no problem").		According to the SUS results, the global usability score for BWS is 87.5 [72.5; 100] (median [range]). The three BWS were assessed as "excellent" to "best imaginable" usability (all scores were above 72.5 on the 100 point scale). SUS scores of BWS a, b, and c were comparable: 90 [77.5; 97.5], 87.5 [72.5; 92.5] and 95 [82.5; 100], respectively. The four categories of the AttrakDiff questionnaire were assessed positively. Global scores for the three BWS regarding attractiveness, PQ, identification of the user to the BWS and stimulation by the BWS to the user were respectively (median [range]): 1 [-3; 3], 2 [-3; 3], 0 [-3; 3] and 2 [-3; 3]. The rating of the three BWS was positive for 84% of all AttrakDiff items, all categories combined. The three BWS yielded independently very similar results for each AttrakDiff dimensio For 18 patients, the general exchange during consultations was incomplete because they could not have the time to discuss everything they wanted. In parallel, respectively eleven and nine patients were not satisfied with their treatment and PD follow-up at the time of the study.	The patients' enthusiasm increased their interest in attaining a better life with the disease (i.e., decrease the symptoms). Eighteen patients felt the need to know more about their symptoms and 19 expressed the interest to get the results, either to justify the monitoring or their feelings to the physician and then improve the discussion (for 17 patients), or to better understand their disease (for 16 patients).	suggestions for use were based on the patients' hopes that BWS monitoring will answer their demands and needs for their PD management. For instance, according to their experiences with the device, patients were able to suggest specific schemes for using the device during their PD follow-up (timing and frequency of monitoring, additional monitoring, extra involvement of the patient, etc.) or new ways of using it (other types of measurements, such as cardiac rhythm or other pathologies, for example).	The patients expressed little anxiety in using the BWS, only two patients manifested apprehension before having seen the device, ten patients after seeing it ("will I be able to use it correctly?", "how should it be done?"), and eight patients after using it. Eleven patients mentioned imagined or lived constraints before use and 12 patients after the one-week use. Few negative a priori conveyed during the first interview: 13 patients had negative preconceptions and four patients considered social embarrassment before use. The device outlook unveiled slight disappointment for four patients and six patients were disillusioned by the device use. Nine patients expressed the wish that the device should not be visible. For example, the ankle bracelet of BWS b regularly reminded people of the electronic parole bracelets. Eight patients pointed out the feeling of being observed or even spied on, wondering about privacy: "We ask ourselves the question, it's a bit embarrassing, then we move on and we keep on wearing, we forget", "I removed it to go to the toilet even if I suspected that it was not important for you". Those patients declared that they did not change their behavior. Six other patients changed their behavior at some point for a short time (extra use of the arm wearing the device, additional time awake, etc.). Patients justified the behavior change to provide the necessary information, which was not bothersome considering the possible advantages of a correct monitoring. In rare cases, patients required help with body placement. Finally, despite specific and individual concerns (16 patients expressed slight discomfort or device-		The usefulness of the tool triggered expectations and hopes for disease follow-up: "I think that it could replace the diaries which are complicated to fill, I could show that to the doctor instead of the sheets", "it would help manage everyday activities".According to 19 patients, clinicians would benefit from patients wearing the devices, as they would be able to adjust the treatment according to the results of BWS monitoring. Finally, twelve patients also thought about research ("we could compare the benefits of different treatments, for example") and the advantages of BWS monitoring for PD knowledge ("when we understand what is happening, we can act").	After the one-week experiment, 17 patients had a positive, and five had a neutral experience with the BWS use. In addition, 17 patients felt concerned about BWS monitoring other than for this study (S1 Table shows quotations from interviews illustrating those results).	I have read the journal's policy and the authors of this manuscript have the following competing interests: CVF received a grant from Association Nationale de la Recherche et de la Technologie (A.N.R.T.). CVF, YR and SH disclose full-time employment by Air Liquide Healthcare. CH declares no conflict of interest. JCC served on advisory boards for Air Liquide Healthcare, Biogen, BrainEver, Denali, EverPharma, Prevail Therapeutic, UCB, and received grants from Sanofi outside of this work. DG received received research funding from Air Liquide Healthcare; received grants from AP-HP (DRCPHRC) and France Parkinson, served on scientific advisory boards for AbbVie and Zambon; received speech honorarium from Medtronic, Abbvie and Merz; received travel funding from Abbvie and Merz. This does not alter our adherence to PLOS ONE policies on sharing data and materials.

The challenges of using medication event monitoring technology with pediatric transplant patients	2007	PEDIATRIC TRANSPLANTATION	Shellmer, DA and Zelikovsky, N	10.1111/j.1399-3046.2007.00681.x	This study investigated the advantages and challenges of using Medication Electronic Monitoring System (MEMS) technology to examine adherence among pediatric kidney transplant patients.	Quantitative	USA	pediatric transplant patients	A final sample of 29 participants met all inclusion criteria, gave informed consent to participate in the study, and completed at least the first phase of the larger study. The sample included 21 males and eight females, with a mean age of 14.03 yr (s.d. = 3.34, range eight to 19 yr). Participants were an average of 35.51 (±10.8) months post-transplant. Patients in this sample missed an average of 23% of their prescribed doses over the three-month duration of the study, with about a third of the patients (n = 10) missing more than 10% of their prescribed doses.<br><br>Although the majority of participants (69%) were Caucasian, there was a good representation of patients from minority backgrounds (13.8% African-American, 3.4% Asian, 6.9% Latino, and 6.9% were multi-ethnic). The median annual household income was approximately $61 000–70 000 (range <$12 000 to >$80 000).	using medication event monitoring technology with pediatric transplant patients			Acceptance of this technology proved difficult, as many patients either declined upfront or dropped out because they did not want to use the MEMS.		Eleven participants found transferring medications into the MEMS bottle difficult, describing several challenges. Some reported forgetting to transfer the medication into the MEMS bottle or keeping up with the medications in the bottle. Others had trouble getting used to the bottle, thought the bottle was too 'top heavy' and bulky to carry around in a pocket. Some patients complained that putting the medications in the MEMS cap added an extra task to or altered the family's regular medication regimen routine. Nineteen participants agreed that using the MEMS cap and bottle had been different than their typical routine, while the other eight participants reported that utilizing the MEMS cap had been similar to their medication taking routine. Seven of the participants reported that the MEMS cap constituted an extra step/burden and/or was difficult to transport making utilization of this technology unappealing. The technology was physically resistant to damage, withstanding four inadvertent drops and trips through airport metal detectors without any loss of information.					Five participants reported that the MEMS cap made it less likely to miss medications. Four participants reported that seeing the bottle prompted them or helped them remember to take their medication, while one participant reported that the bottle's distinguishable shape and size assisted in adherence efforts.<br><br>Beyond the effects reported by participants, it is important to note that the MEMS caps were extremely useful in the provision of individual adherence data. The data that were collected and downloaded were in excellent condition providing detailed information regarding daily dosages, inter-dose intervals and overall pattern of adherence for this sample of patients.	Six participants reported that the MEMS cap changed their usual routine (e.g., using a pillbox), which they did not like, increasing the chance that they would not use it accurately. Lastly, three participants worried about forgetting to take their medications when using the MEMS bottle.	N
The Use of Telemonitoring in Managing the COVID-19 Pandemic: Pilot Implementation Study	2021	JMIR FORMATIVE RESEARCH	McKinstry, B and Alexander, H and Maxwell, G and Blaikie, L and Patel, S and Guthrie, B and Technology Enabled Care TeleCOVID	10.2196/20131	Most people with COVID-19 self-manage at home. However, the condition can deteriorate quickly, and some people may develop serious hypoxia with relatively few symptoms. Early identification of deterioration allows effective management with oxygen and steroids. Telemonitoring of symptoms and physiological signs may facilitate this. The aim of this study was to design, implement, and evaluate a telemonitoring system for people with COVID-19 who are self-managing at home and are considered at significant risk of deterioration.	Mixed	UK	people with COVID-19	[illegible] patients; 111 patients were from Lanarkshire and 5 were from Highland (N=116).<br><br>Characteristic Patients who submitted readings (n=71) Patients who did not submit readings (n=45)<br>Sex, n (%)<br><br>Women 40 (56) 20 (44)<br><br>Men 31 (44) 25 (56)<br>Age in years<br><br>Mean (SD) 51.3 (15.8) 54.0 (13.2)<br><br>Range 24-94 25-87	a pulse oximeter and the monitoring system			All patients found self-monitoring to be reassuring.				All 11 people interviewed who had used the remote monitoring system described it as "easy" or "straightforward."			All 14 people interviewed said remote monitoring provided reassurance or "stopped you worrying."		BM has a paid consultancy with the Scottish Government to advise on the implementation of remote monitoring. He has also in the last 2 years provided consultancy services to the company Pharmatics to design an app to manage chronic obstructive pulmonary disease.
Challenges for Deploying IoT Wearable Medical Devices Among the Ageing Population	2018	DISTRIBUTED, AMBIENT AND PERVASIVE INTERACTIONS: UNDERSTANDING HUMANS, DAPI 2018, PT I	Xing, F and Peng, GC and Liang, T and Jiang, JY	10.1007/978-3-319-91125-0_25	The phenomenon of ageing population is raising substantial pressure to the national health and welfare systems of many countries in the world, and can be a potential threat to social stability and economic development. IoT wearable medical technologies have the potential to aid this struggle, but anecdotal evidence showed that large-scale deployment of IoT wearable devices among the ageing population could be fraught with challenges, which have not been well reported in academic literature. Therefore, this paper aims to address this knowledge gap by reporting on an exploratory study that firstly investigated older people's user requirements towards wearable medical devices and secondly explored potential challenges and difficulties for large-scale deployment of such devices.	Qualitative	China	Older adults	Family ID Number of 60 + people Number of family members Total<br>Focus group/family 1 1 2 3<br>Focus group/family 2 1 2 3<br>Focus group/family 3 1 2 3<br>Focus group/family 4 2 4 6<br>Focus group/family 5 2 2 4	IoT Wearable Medical Devices Among the Ageing Population					3.2 Issues About Device Design and Development<br>The complexity and variety of user requirements as discussed above will inevitably raise issues and difficulties when designing and developing wearable medical devices.<br><br>In particular, in order to monitor physical variables (e.g. movements, steps, and motion) and various vital signs (e.g. blood pressure, pulse, heart rate, blood oxygen, etc.) of elderly people, different types of sensors will need to be used [16,17,18]. The ideal solution will be to integrate all the needed sensors into one single device. This was supported by the focus group participants, who stated that they "want an integrated piece of tool rather than wearing too many different devices" (Focus Group 3). This however can lead to severe technical challenges regarding accuracy, stability, battery lifetime, size, and weight of the wearable device. Specifically, when too many sensors need to be embedded into one single device, its internal circuit and structure will become very complicated. When device manufacturers try to reduce and compress the size of the device, its accuracy and stability can often be affected [19, 20]. Faced with these problems, some manufacturers may then try to reduce the size of the device battery in order to leave more space for other crucial components, but this solution can in turn reduce battery lifetime and so lead to inconvenience to the users [16, 19]. Owing to these technical issues, the weight and size of an integrated wearable medical device may not be reduced easily, and so can cause discomfort to elderly people when they wear a relatively large and heavy piece of device on a daily basis [21].		3.1 Complicated and Diverse User Needs<br>It emerged from the focus group data that user requirements of elderly people for wearable technologies contain three main categories, namely healthcare, data privacy, and commodity requirements. In terms of healthcare needs, the participated elderly people and their families raised a number of essential hardware and software functions to be ideally included in wearable medical devices, such as:<br>Having a panic button exclusively for medical emergencies;<br>Having embedded sensors that automatically monitor vital signs (e.g. blood pressure, pulse, heart rate, blood oxygen), measure steps and sleep duration;<br>Having additional sensors that can accurately measure and support pulse diagnosis (i.e. a disease measurement method used commonly in traditional Chinese medicine);<br>Allowing creation of electronic medical record, booking of hospital beds, booking of medical appointments, interpretation of their vital signs, and medical recommendations and medicine reminders through mobile app.<br>It is apparent that these types of functions could be particularly useful to elder people with chronic diseases (e.g. Hypertension, High Cholesterol, Diabetes, Ischemic Heart Disease, Dementia, Parkinson's disease, Heart Failure), which do not necessarily require hospitalization but need long-term monitoring and treatment [13, 14].	From a customer perspective, the focus group participants reinforced that for most wearable medical devices available in the market, "the affordable ones often did not contain all the functions they need, and the ones that can satisfy their requirements are always far too expensive for them to buy" (Focus Group 2). This dilemma will inevitably affect large-scale deployment of IoT wearable medical devices. However, the focus group participants worried that it might not be easy to get involved a large number of doctors and medical caregivers in public hospitals in the caring end of wearable devices.			Last but not least, the focus group results showed that elderly people and their family members had serious doubts about current legal regulations associated with the usage of wearable medical devices. For instance, the two elderly people in focus group 5 questioned that "if we use these wearable devices on a regular basis, but things did not go well, such as, the device did not measure my heart rate accurately and so wrong treatment is provided to me, who should be legally responsible for it". In fact, the failure of wearable devices and related services can be caused by a mixture of potential reasons, including hardware issues (e.g. inaccurate and unstable measurements), software flaws (e.g. inadequate data processing and analysis), irresponsible caring professionals (e.g. wrong diagnosis and decisions made by doctors), and even inappropriate user behaviors (e.g. the elderly person does not charge and/or wear the device properly). Consequently, when medical accidents happened to the device users, it could be	UNDECLARED
Accuracy and Acceptability of Wearable Motion Tracking for Inpatient Monitoring Using Smartwatches	2020	SENSORS	Auepanwiriyakul, C and Waibel, S and Songa, J and Bentley, P and Faisal, AA	10.3390/s20247313	Inertial Measurement Units (IMUs) within an everyday consumer smartwatch offer a convenient and low-cost method to monitor the natural behaviour of hospital patients. However, their accuracy at quantifying limb motion, and clinical acceptability, have not yet been demonstrated. To this end we conducted a two-stage study: First, we compared the inertial accuracy of wrist-worn IMUs, both research-grade (Xsens MTw Awinda, and Axivity AX3) and consumer-grade (Apple Watch Series 3 and 5), and optical motion tracking (OptiTrack).	Quantitative	UK	in-patients	average age: 64 years; interquartile age range: 24–92 years) completed the acceptability questionnaire. A further 15 healthcare professionals (66% female; doctors = 5, nurses = 4, therapists = 3, and healthcare assistants = 3) working directly with these patients were also recruited. In-Patient Characteristics n (Weights)<br>Age (years)—Avg (IQR) 64 (24–92)<br>Female sex—n (%) 22 (50)<br>Male sex—n (%) 22 (50)	Wearable Motion Tracking for Inpatient			sensors were easy to operate and learn to use, comfortable, did not limit daily activities, did not cause anxiety and unobtrusive in appearance as seen in Table 2. A significant proportion of in-patients reported that they felt neutral towards the sensors or had nothing in particular to comment when asked open questions about system likes (n = 18), dislikes (n = 34), and expected functions and characteristics (n = 31) (ioQ1–3).									N
Understanding the Adoption of Smart Wearable Devices to Assist Healthcare in China	2016	SOCIAL MEDIA: THE GOOD, THE BAD, AND THE UGLY	Gao, S and Zhang, XM and Peng, SQ	10.1007/978-3-319-45234-0_26	With the development and advancement of information communication technology, smart wearable devices are playing a more and more important role in peoples' daily lives. This study aims to investigate the adoption of smart wearable devices to assist healthcare in China. Based on the previous technology diffusion theories (e.g., TAM, IDT), a research model with ten research hypotheses was proposed in this research.	Quantitative	China	General public	180 completed questionnaires were collected, among which 145 of them were valid questionnaires (i.e., valid respondent rate 80.6 %). Among the participants, 67 of the participants were male, and 78 were female. In terms of age, 68 participants were 25 years old and under 25 years old, while 77 participants were over 25 years old.	Smart Wearable Devices to Assist Healthcare					According to the results, perceived ease of use, perceived usefulness, trust and compatibility were found to have a statistically significant effect on users' attitude towards SWD, while perceived risk and personal characteristics did not have significant impact on users' behavior of using SWD.							UNDECLARED
Understanding the Experience of Cancer Pain From the Perspective of Patients and Family Caregivers to Inform Design of an In-Home Smart Health System: Multimethod Approach	2020	JMIR FORMATIVE RESEARCH	LeBaron, V and Bennett, R and Alam, R and Blackhall, L and Gordon, K and Hayes, J and Homdee, N and Jones, R and Martinez, Y and Ogunjirin, E and Thomas, T and Lach, J	10.2196/20836	This study aims to understand the experience of managing cancer pain at home from the perspective of both patients and family caregivers to inform design of the Behavioral and Environmental Sensing and Intervention for Cancer (BESI-C) smart health system.	Mixed	USA	cancer Patients and Family Caregivers	Overall, almost half of the total sample (10/22, 46%) was aged between 50 and 59 years, with an equal number of females and males (11/22, 50%). The participants were primarily White (20/22, 91%) and non-Hispanic/Latino (21/22, 96%). The majority of patients (11/12, 92%) had a primary residence classified by the Centers for Medicare and Medicaid Services as rural [47]. Caregivers were predominantly female (7/10, 70%), lived full time with the patient (9/10, 90%), and were the significant other or spouse (5/10, 50%). The average patient-reported NIH PROMIS pain interference score was 7.16 (0, lowest interference to 10, highest interference), and half of the patients (6/12, 50%) self-reported their performance status as symptomatic, but ambulatory and able to complete their basic needs independently (Eastern Cooperative Oncology Group [48] score of 1). The most common malignancy was	an In-Home Smart Health System			Overall, patients and family caregivers expressed interest and receptivity to the concept of BESI-C, validated the importance of monitoring cancer pain at home, were eager for innovative ways in which to do so, and provided constructive feedback regarding the system components.		Participants expressed a strong preference for technology that is unobtrusive, simple, convenient, durable, and aesthetically pleasing and that involves minimal interference with daily activities, such as sleep. Some participants expressed concerns about having multiple watches and having to charge them and the potential for them being misplaced.		When patients and caregivers were interviewed together, caregivers often helped encourage an initially skeptical or reluctant patient to try the technology or reassured them about practical aspects, such as where environmental sensors could be placed in the home or that they actually had enough electrical outlets. a desire for multifunctionality (eg, could environmental sensors measure variables beyond those focused on cancer pain, such as general air quality in the home, or other symptom management issues, such as sleep apnea, and could wearables measure additional factors such as blood pressure or blood glucose levels).	Regarding wearable sensors, participants were intrigued by the smart watch platform but wondered if (1) it would be difficult or complicated to answer questions on the watch owing to the relatively small touch screen, (2) they would have trouble using the technology, and (3) EMAs would become annoying. Participants also discussed practical issues related to internet connectivity and power outages (particularly in rural areas); the number of electrical outlets in the home needed to plug in environmental sensors; how much space the sensors would occupy; and placing sensors so that they are discrete, out of the way of small children, and any cords are safely secured to prevent trip hazards or falls.		There was also the acknowledgment that a system such as BESI-C could be particularly helpful in assisting caregivers to tune in to variables that may influence a patient's pain, but that may not be readily obvious. For example, when patients were asked about specific aspects of the technology, it often prompted comments acknowledging the difficulty of their illness on their caregiver and their concern for the impact it has had on them. Patients expressed that the BESI-C system would be helpful in providing an objective picture of how their caregiver was actually coping and what support their caregiver needed.	For both environmental and wearable sensors, participants expressed concerns regarding privacy. Specific to the environmental sensors, patients discussed the importance of having everyone in the home consent to sensor placement. Primary privacy concerns related to possible audio and video data collection as well as data sharing (eg, where are the data going and who has access to them).	N
Factors Influencing Continued Wearable Device Use in Older Adult Populations: Quantitative Study	2023	JMIR AGING	Esquivel, KM and Gillespie, J and Kelly, D and Condell, J and Davies, R and McHugh, C and Duffy, W and Nevala, E and Alamaki, A and Jalovaara, J and Tedesco, S and Barton, J and Timmons, S and Nordstrom, A	10.2196/36807	This study aimed to understand the usability factors that most influence whether an older adult will decide to continue using a wearable device.	Quantitative	Northern Ireland, Ireland, Sweden, and Finland	Older adults		Continued Wearable Device Use in Older Adult Populations				In addition to comparing usability with the intention to continue using the device, we evaluated what effect previous activity tracker experience (usability question 2) had on usability. In total, 20% (13/65) of the participants said that they had previously used a wrist-worn activity tracker, whereas 80% (52/65) of the participants said that they had never used a wrist-worn activity tracker before the trial. Statistical significance was evaluated for the SUS score of participants who had previous experience versus those who did not have previous experience. The results from an independent t test (Table 5) showed that there was no significant difference between a user's SUS score and whether they had previous experience with a wrist-worn activity tracker (P=.28).	[illegible] 67.15 (SD 18.27). Analysis of SUS scores by region showed that Finland had both the largest (11/23, 48%) percentage in the marginal category and the lowest (2/23, 9%) percentage in the unacceptable category, making it the best-performing region given that scores in the acceptable category were similar across regions. Northern Ireland and Ireland performed equally well in both the marginal and acceptable categories, with 41% (9/22) of people. Sweden had the largest (9/20, 45%) percentage in the acceptable category, but conversely, scored the worst in the unacceptable category (6/20, 30%). Across all regions, a total of 43% (28/65) of people thought that the device had an acceptable SUS score of >70. The participants who indicated that they would continue using the device averaged an SUS score of 71.8; thus, the "continue using" group on average considered the usability of the device to be within the "acceptable" category (range >70). In comparison, those who indicated that they would not be interested in continuing to use the device averaged an SUS score of 51.7 and, thus, the "not continue using" group on average ranked the usability of the device within the "marginally low acceptability" category (range >50 and <65). Results from an independent t test showed that the SUS scores of the 2 "continued use" groups were statistically significant. both comfort at night and becoming more active are key early indicators of whether a user will continue using and wearing a device. The results showed that none of the 3 physical function measures—WT10M1, WT10M2, and STS5—correlated with continued device use. This indicates that physical function before using the device is not likely to influence whether the participant will continue using the device in the future.							N
Home health monitoring around the time of surgery: qualitative study of patients' experiences before and after joint replacement	2019	BMJ OPEN	Grant, S and Blom, AW and Craddock, I and Whitehouse, M and Gooberman-Hill, R	10.1136/bmjopen-2019-032205	Hip and knee replacements are common major elective surgical interventions with over 200 000 performed annually in the UK. Not all patients achieve optimal outcomes or experience problems or delays in recovery. The number of patients needing these operations is set to increase, and routine clinical monitoring is time-consuming and resource-consuming for patients and healthcare providers; therefore, innovative evaluation of surgical outcomes is needed. The aim of this qualitative study was to capture the patient experience of living with a novel home monitoring sensing system during the period around joint replacement.	Qualitative	UK	patients before and after joint replacement	[illegible] Gender THR/TKR Cohabitation status Mr Hayes 70 Male THR Lives with spouse Mrs Henry 67 Female THR Lives with spouse Mr Price 85 Male THR Lives with spouse Mrs Evans 85 Female THR Lives alone Mr Connell 67 Male TKR Lives with spouse Mrs Griffiths 63 Female THR Lives with son and daughter in law* Mrs Wilson 89 Female TKR Lives alone Mrs Murray 71 Female THR Lives alone Mrs Bailey 73 Female THR Lives with partner Mrs Harrison 65 Female THR Lives with spouse and son* Mrs Thompson75 Female THR Lives with spouse Mr Baker 71 Male TKR Lives with spouse *Adult household member.	Home health monitoring around the time of surgery			Patients who agreed to the technology found living with it acceptable.							A home- sensing system that monitors the environment and activity of the people in the home could provide an innovative way of assessing patients' surgical outcomes. At a time characterised by reduced mobility, functional limitations and increased pain, patients in this study relied on informal and formal supportive networks to help maintain the system through the busy trajectory of the perioperative period.		N
Self-efficacy and trust in consumers' use of health-technologies devices for sports	2021	HELIYON	Chamorro-Koc, M and Peake, J and Meek, A and Manimont, G	10.1016/j.heliyon.2021.e07794	The commercial market for wearable health technology is growing but the value these technologies provide for consumers is debatable, as many technologies lack formal validation and are being abandoned at a high rate. The enabling of self-efficacy mechanisms in the design of health technologies, through the factors identified by self-determination theory and the Technology Acceptance Model, could increase the uptake and continued use of these devices. The aim of this research was to investigate how and why people use wearable health technology, and to evaluate their experiences from the perspective of perceptions of autonomy, safety, information security, information accuracy and willingness for continued use.	Quantitative	Australia	General public	Demographic data of participants were collected, which allowed for the development of a profile of survey respondents with a special interest in wearable sports technology (see Table 3). Of the 47 useable responses from respondents who considered themselves "sport enthusiasts or athlete", females (70.2%) represented a larger proportion than males (29.8%). Most respondents were younger than 25 years (44.7%). Almost half of the participants (42.6%) practiced sports three times a week and had been participating seriously in sports for more than 10 years (44.7%). Of those, 87.6% participated in one to two sports. Considering sports participation settings, most participants revealed that they practiced their sport as individuals (87.2%). Sports practice was mainly for competitions (68.1%), rather than for health interest (31.9%). Results also indicated that most participants had their own coach when participating in sports	health-technologies devices for sports			As can be seen in Table 7, most respondents (83%) intended to continue to use their wearable technology device in the future. However, more than half of them admitted that their future training would not be determined by metrics collected by their wearable technology device (53.2%). Similarly, more than half (57.4%) of the respondents indicated that they would not modify their training workload based on the metrics provided by their wearable technology.Nevertheless, their perceived willingness for continued use (PWCU) particularly in relation to the wearable technology was low across all three different usage situations (mean 2.12). This means that respondents seemed unwilling to voluntarily use wearable technology during sport practice or in their daily lives or both, even if wearable technology provided information concerning respondents' health and their sports practice.		For perceived quality of previous usage experience (PQ), overall results showed that respondents tended to have good experiences when using wearable technology devices (mean 4.06).	Among six different intended uses, results showed that over half of the participants had adopted wearable technologies to promote their health through fitness and activity monitoring (53.2%), while another quarter (25.5%) were monitoring their activity levels. Only a small proportion (2.1%) used their wearable technologies to receive instructions on how to perform their sports workout. Around 10% of participants stated other intended uses for the technology, such as to remind them about, and/or to monitor data regarding, 'food consumption', 'heart rate', 'calories', 'sleep' and 'training-session intensity'. When using wearable technologies, most participants (93.6%) reported that their use was voluntary. Most participants (97.9%) also said that they did not receive any incentive or bonus to use wearable technology (see Table 5). While wearable sensor technology was commonly used for monitoring training load and health, respondents (61.7%) were less interested in using their wearable technology devices for tracking their sleep patterns (see Table 6).				Based on the descriptive statistics presented in Table 8, respondents seemed to gain some sense of autonomy, or perceived autonomy (PA), because they were substantially involved in choosing the wearable technology that they used (mean 4.26). Respondents seemed to be aware that wearable technology devices captured information by monitoring their activities. However, the overall responses of perceived safety (PS) were neutral (mean 3.30), indicating that respondents neither disagreed nor agreed that they were provided adequate information about 'who would see the data collected' from the wearable technology.	For perceived information security (PIS), the results showed that respondents felt little concern about how their information and data were kept secure and private from people without access permission (mean 2.00). With regards to perceived information accuracy (PIA), respondents agreed that they were provided adequate information about what metrics would be measured by their wearable technology device (mean 4.13).	N
Privacy Perceptions in Ambient Assisted Living	2019	ICT4AWE 2019: PROCEEDINGS OF THE 5TH INTERNATIONAL CONFERENCE ON INFORMATION AND COMMUNICATION TECHNOLOGIES FOR AGEING WELL AND E-HEALTH	Schomakers, EM and Ziefle, M	10.5220/0007719802050212	Ambient Assisted Living (AAL) Technologies may help address the challenges that the ageing populations pose on the health care systems by supporting older adults in ageing-in-place, improving independence, and quality of care. Technology acceptance by the potential users and particularly privacy concerns are decisive obstacles to the widespread use of AAL. In order to examine privacy perceptions in detail, 86 participants (50% older than 50 years) evaluated AAL technologies and privacy concerns in a questionnaire approach.	Quantitative	Germany	older adults	Ascenario-basedapproachwasusedtoaccountforthesampleofusersnotlivingwithdementia. 125participantsstartedthequestionnaire,of whichn =86(68.8%)completedit.Theparticipants werebetween19and88yearsold(M=46.75, SD=20.3), with50%olderthan50years,and58.8%women.All levelsofeducationwerepresentinthesample(compulsorybasicsecondaryschooling:4%,secondary education:13%,apprenticeship:26%,universityentrancediploma:50%,universitydegree:6%;no diploma:1%).AllparticipantsarefromGermanyor Austria.Regardingtheparticipantsexperienceswith elderlycare,20.9%havealreadynursedanelderly relative,92%oftheparticipantshaveexperiencedthat	AmbientAssistedLiving							Reliability,monitoredactivities,datagranularity, automaticdecisions,andcontrollabilityareonaveragequitesimilarlyimportanttotheparticipants.				Theparticipantsratedfiveconcernsregardingtheir privacy,thathavebeenmentionedinpreliminary focusgroupsessions.Misuseofdataisthemostprevalentconcernfortheparticipants3,butisonlyrather greedon(M=3.76,SD=1.4,min=1,max=6). Feelingsofsurveillance(M=3.7,SD=1.3)andobtrusivenessofthetechnology(M=3.64,SD=1.3) areadditionalconcerns,thattheparticipantsrather agreeon.Thattheyareworriedaboutreactionof familyandfriendsisslightlydenied(M=3.43,SD= 1.3).Concernsaboutautomaticdecisionsthatthe systemmakesabouttheusersarerejected(M= 2.51,SD=1.2).Theperceptionofprivacyconcerns ismoderatelycorrelatedwiththedispositiontovalue privacy(r=.57,p<.01),butnottotheotheruser factors.	UNDECLARED
Efficacy and acceptability of using wearable activity trackers in older adults living in retirement communities: a mixed method study	2022	BMC GERIATRICS	Zhang, ZJ and Giordani, B and Margulis, A and Chen, WY	10.1186/s12877-022-02931-w	Wearable activity trackers hold the potential for enhancing health and fitness, but the use of wearable activity trackers has remained largely unexplored in older adults. The purpose of the current study was to examine the effectiveness and acceptability of wearable activity trackers for promoting physical activity (PA) in older adults living in retirement communities.	Mixed	USA	Older adults	The remaining 40 participants completed the endpoint assessment and were included in the final analysis. The average age of participants was 85.4 years (SD = 5.2) and more than half (55%) of them were 85 years or older. The majority of participants were female (80%), had graduated from high school or above (95%), and were not married (67.5%). All of the participants were Whites.	wearable activity trackers			The average score was 4.00 for item 1 measuring the overall satisfaction with the wearable activity tracker. The mean score of all items was 4.00. The results of the Acceptance questionnaire indicated that participants considered the wearable activity trackers to be helpful and acceptable.One participant dropped out of the study in the first week of intervention, reporting dissatisfaction on the accuracy of the activity tracker in recording step counts.		The average score of each item of the Acceptance questionnaire ranged from 3.83 for the item "I found the Fitbit clear and understandable to use" Theme 2: disliked features<br>When asked about the disliked features of the activity tracker, eight participants (20%) reported that wearing the activity tracker was sometimes uncomfortable. The uncomfortable experience was mainly because the watchband of activity tracker was too tight or the skin touch with the metal part of the activity tracker was annoying. Seven participants (17.5%) reported that they found it hard to read the visual feedback mainly because the numbers displayed on the activity tracker were too small.		Theme 3: additional suggestions<br>When asked about the possible improvement that could make the activity tracker more useful to them, 12 participants (30%) expressed their interest in exploring other functions of the activity tracker, such as tracking water intake and sleep. The activity tracker showed the potential for long-term use based on the interviews. The majority of participants (95%) expected that they would continue using the activity tracker. Only two participants (5%) reported they would not continue using the activity tracker. One indicated that the activity tracker was perceived inaccurate and the other indicated that he was not interested in the feedback the activity tracker provided.			[illegible] tracker, 20 participants (50%) indicated that the activity tracker made them more aware of their walking/daily steps. Nine participants (22.5%) also mentioned that the activity tracker helped them monitor their heart rate.<br><br>Checking the visual feedback from the activity tracker facilitated their PA behavior change through goal achievement. Eleven participants (27.5%) reported that being aware of their PA helped them achieve their activity goals more effectively. Note that this was different from awareness in that it evidently involved activity goals, although the goal achievement could be a consequence of self-		N

A Biofeedback App for Migraine: Development and Usability Study	2021	JMIR FORMATIVE RESEARCH	Ingvaldsen, SH and Tronvik, E and Brenner, E and Winnberg, I and Olsen, A and Gravdahl, GB and Stubberud, A	10.2196/23229	The objective of this study was to evaluate and improve the feasibility and usability of an mHealth biofeedback treatment app for adults with migraine.	Mixed	Norway	adults with migraine	Patient baseline demographics.<br><br>Characteristic Value (n=18)<br>Age (years), mean (SD) 40.6 (9.8)<br>Gender, female, n (%) 17 (95	A Biofeedback App for Migraine				[illegible]		[illegible]				One case of prespecified adverse events was recorded, in which a patient developed a skin rash from the sticky SEMG electrodes. The rash was mildly painful and lasted for a week. No other adverse events were detected.	NTNU and St. Olavs Hospital, Trondheim University Hospital, may benefit financially from the commercialization of the proposed treatment through future possible intellectual properties. This may include financial benefits to the authors of this article. ET, AO, AS are cofounders of the Nordic Brain Tech AS, a spin-off company that was established based on the proposed treatment in this and previous studies at NTNU. In addition, ET, AO, AS are coinventors of the proposed treatment in this study and may benefit financially from a license agreement between Nordic Brain Tech AS and NTNU. No conflicts were declared for SHI, EB, IW, and GBG.
Questionnaire study of electronic wear-time tracking as experienced by patients and parents during treatment with removable orthodontic appliances	2013	JOURNAL OF OROFACIAL ORTHOPEDICS-FORTSCHRITTE DER KIEFERORTHOPADIE	Schott, TC and Schrey, S and Walter, J and Glasl, BA and Ludwig, B	10.1007/s00056-013-0143-2	To survey how patients and parents rate microelectronic wear-time tracking (TheraMonA (R)) during treatment with removable orthodontic appliances.	Quantitative	Germany	patients and their parents.	The ensuing questionnaire study gave 128 consecutive patients (64 boys and 64 girls) and 128 parents an opportunity to rate their experience with electronic wear-time tracking during treatment. A total of 125 questionnaires were completed and returned yielding a response rate of 98%. The mean age of these patients was 11.99±2.7 years (range 6–20 years), although no consideration was given to age-related factors in analyzing the questionnaires. Treatment venues were the Department of Orthodontics at Eberhard Karls University in Tübingen (27% of patients) and three orthodontic offices – two in Rhineland–Palatinate and one in North Rhine–Westphalia (73% of patients).	electronic wear-time tracking as experienced by patients and parents during treatment with removable orthodontic appliances		A majority of respondents had a favorable impression of wear-time tracking.		[illegible]					Printed wear-time documents from the clinician's computer were considered a "nice certificate of compliance" by 46% of patients, and 38% of them stated that they intended to improve their compliance when faced with a poor record. Indeed, 48% of parents believe that wear-time tracking can improve the therapeutic success, while 32% believe that it can reduce the duration of treatment.<br>Feelings about effects on treatment (Tab. 4)<br>The view that wear-time tracking contributed to the success of their child's orthodontic treatment prevailed in 47.5% of parent respondents, compared to 5.7% of those who claimed that treatment success was not influenced by the monitoring. Of the parents, 32.4% believed that the monitoring would reduce the duration of treatment with the removable orthodontic appliance. Only 1 parent believed that the monitoring detracted from the quality of treatment (0.7%), and 4 parents claimed it worsened treatment-related discomfort (2.9%). Fifteen parents (11%) expressed no opinion about the effects of wear-time tracking on therapeutic success.<br>These favorable ratings by patients and their parents may help future patients and users to decide for or	Worries and concerns<br>The majority of parents (74.2%) had no worries or concerns about the use of a microsensor, although 9.7% were concerned about the possibility of "electrosmog" being released, and 6.5% were apprehensive about arguments back home should they not be compliant enough. An equal percentage questioned the accuracy of monitoring or were concerned about sharing responsibility for the success of treatment.	N
"Listen to Your Immune System When It's Calling for You": Monitoring Autoimmune Diseases Using the iShU App	2022	SENSORS	Ortet, C and Costa, LV	10.3390/s22103834	This study reports on the use of wearable sensors for inflammation surveillance and autoimmune disease management based on a literature search and evaluation of an app prototype with fifteen stakeholders, in which eight participants were diagnosed with autoimmune or inflammatory diseases and four were healthcare professionals.	Mixed	Portugal	participants were diagnosed with autoimmune or inflammatory diseases	age: 23-58; 9F, 6M; various occupations	Monitoring Autoimmune Diseases Using the iShU App		According to the evaluation of iShU (Table 5), it had an overall median of 4.5 from the 5-point Likert scale.		[illegible]		[illegible] was also mentioned that there is an interconnect failure between mHealth with healthcare professionals; a shortcoming with urgency mechanisms and customization; a lack of support in post-diagnosis and stress management throughout the course of the disease; missing previous evaluation and validation by experts before its launch on the market; and the tendency to not be accessible for all users due to being expensive and not inclusive.<br><br>Participants also shared their desires in a mHealth app, declaring they would like to have the possibility of (i) quickly monitoring all vital signs; (ii) having patient–doctor and patient–patient channels; (iii) having alerts and outreach for app usage, health monitoring, and altered values; (iv) pairing with existing smartwatches; (v) being provided self-care tips and updated information; (vi) having motivators that trigger continued use; (vii) having a repository of symptomatology; and (viii) having an accessible app, both in price and digital inclusion.			Several opportunities were emphasized: (i) the possibility of exporting all the health data and providing it to a doctor; (ii) the connection between patient and healthcare services; (iii) medication management and reminder; (iv) geolocation to health facilities; (v) being an official app recommended by the health care community; (vi) have a social media/community and forum; (vii) be translated to several languages; and (viii) add features such as the possibility of taking photos of visible symptoms to complete the diagnosis. However, threats of indirect competition with an already strong presence in the mHealth market, as well as ethical issues in data protection and sharing were also pointed out.<br><br>Given that the most monitored autoimmune diseases are diabetes and rheumatoid arthritis, there is a gap, as well as an opportunity, in support for other autoimmune diseases, with a special focus on systemic ones that have greater representation of inflammation signs. As such, the authors developed a concept based on the 5W2H framework, since they fit well for the starting point of brainstorming and initial development.		N
Research on a Community-based Platform for Promoting Health and Physical Fitness in the Elderly Community	2013	PLOS ONE	Tsai, TH and Wong, AMK and Hsu, CL and Tseng, KC	10.1371/journal.pone.0057452	In this study, we specifically evaluated the users' intention of using the iFit using a technology acceptance model (TAM). A total of 101 elderly subjects (27 males and 74 females) were recruited.	Quantitative	Taiwan	Older adults	were male and 73.3% were female, and with an average age of 79 (see Table 1). Of the total respondents, 52.5% had previously undergone fitness tests, while 30.7% had previously played interactive games. While many of the elderly respondents had a certain degree of experience with fitness tests, they were less familiar with the use of interactive games for testing or training.	a Community-based Platform for Promoting Health and Physical Fitness in the Elderly Community		Results indicate that perceived usefulness, perceived ease of use and usage attitude positively impact behavioral intention to use the platform.			risk factors among the frail elderly, mechanism for slowing the advance frailty, and technology acceptance and support for promoting physical fitness.				The iFit platform can offer user-friendly solutions for a community-based fitness care and monitoring of elderly subjects.		N
Assessing the factors that influence the adoption of healthcare wearables by the older population using an extended PMT model	2022	TECHNOLOGY IN SOCIETY	Singh, N and Misra, R and Singh, S and Rana, NP and Khorana, S	10.1016/j.techsoc.2022.102126	The present study aims to assess the intention of the older population to use healthcare wearable devices (HWDs) for wellness during life-threatening situations like COVID-19.	Quantitative	India	Older adults	https://www.sciencedirect.com/science/article/pii/S0160791X22002676#tbl1	healthcare wearables by the older population		he findings indicate that there is a significant impact of both the protective and utilitarian dimensions of HWDs. The study assesses the perceived vulnerability and severity of the older population in COVID 19 and the intention to use HWDs to handle such health crises. The study confirms that perceived usefulness and information accuracy of HWDs, as well as self-efficacy, perceived severity, and perceived vulnerability of senior citizens are high during the COVID-19 pandemic, which significantly influences their intention to use HWDs.									UNDECLARED
Women's Attitudes Toward Self-Monitoring of Their Pregnancy Using Noninvasive Electronic Devices: Cross-Sectional Multicenter Study	2019	JMIR MHEALTH AND UHEALTH	Schramm, K and Grassl, N and Nees, J and Hoffmann, J and Stepan, H and Bruckner, T and Haun, MW and Maatouk, I and Haist, M and Schott, TC and Sohn, C and Schott, S	10.2196/11458	This survey aimed to elucidate the attitudes of women toward self-monitoring of their pregnancy using noninvasive electronic devices. The technical details of the proposed devices were not specified.	Quantitative	Germany	Pregnant women	Characteristic Responses, n (%)<br>Age (years)<br><br>18-20 12 (2.4)<br><br>21-25 47 (9.3)<br><br>26-30 168 (33.2)<br><br>31-35 178 (35.2)<br><br>36-40 84 (16.6)<br><br>>41 17 (3.4)<br>Highest education degree<br><br>Dropped out of school 11 (2.2)<br><br>Secondary education ending with ninth grade 41 (8.1)<br><br>Secondary education ending with tenth grade 147 (29.0)	Women's Attitudes Toward Self-Monitoring of Their Pregnancy Using Noninvasive Electronic Devices		In the first step, the questionnaire explored if devices for self-monitoring could reduce physician consultations. When asked whether they regarded self-monitoring of their pregnancy as an alternative to consulting a physician, only 5.9% (29/493) of our study participants strongly agreed, 36.7% (181/493) of participants strongly disagreed, and the remaining 57.4% (283/493) of women favored an intermediate to skeptical standpoint toward this statement (Table 2). However, significantly more women regarded it as an alternative prior to seeing a doctor when they felt fewer baby movements (P<.001). Still, only 7.7% (38/496) strongly believed they would visit the ED less often during pregnancy if such devices were in place.<br><br>[illegible]	The attitudes toward pregnancy monitoring with eHealth devices differed substantially depending on socioeconomic status. Among patients with a university degree, 37.1% (77/207) would have consulted their obstetrician less often if they had the chance to monitor their fetus at home compared with 23.1% (66/286) without a university degree (P=.001). At the same time, 75.8% (157/207) of academics preferred a more detailed readout over a binary readout of monitoring devices compared with 66.3% (161/243) of nonacademics (P=.03). Both the attitude toward pregnancy monitoring with wearable devices and preferences for features of mobile devices did not depend on age, the number [illegible]			Preferences for the readout of mobile devices for pregnancy monitoring varied. While 29.8% (134/450) of the participants preferred a simple binary reading stating either that everything is normal or that consulting a physician is recommended, a majority of 39.6% (178/450) were in favor of more detailed information allowing graduating fetal well-being and providing information on fetal status. The remaining 30.7% (138/450) even wanted such devices to display as much information as possible.<br><br>[illegible]					N
Patient and Provider Perspectives on Symptom Monitoring During Outpatient Chemotherapy: Interview Study	2023	JMIR FORMATIVE RESEARCH	Chen, L and Bartel, C and Cai, XL and Cheng, YHD and Perer, A and McClaine, S and Kairis, E and Durica, K and Huang, WY and Low, CA	10.2196/46001	The goals of this study were to learn about patient and provider perspectives on monitoring symptoms during chemotherapy, understand barriers and challenges to effective symptom monitoring at one institution, and explore the potential value of remote symptom monitoring between provider visits.	Qualitative	USA	cancer patient During Outpatient Chemotherapy	Patients (mean 51, SD 19.2, range 19-82 years; 10/15, 67% White; 6/15, 40% female) had a variety of primary diagnoses, including pancreatic cancer (n=3), colorectal cancer (n=3), breast cancer (n=2), other solid tumors (n=2), and leukemia or lymphoma (n=5). Most patients (10/15, 67%) had attended at least some college.	Patient and Provider Perspectives on Symptom Monitoring During Outpatient Chemotherapy				[illegible]							N
Colombian Stakeholder Perceptions and Recommendations Regarding Fall Detection Systems for Older Adults	2023	GERIATRICS	Avella-Rodriguez, E and Gomez, L and Ramirez-Scarpetta, J and Rosero, E	10.3390/geriatrics8030051	This study aimed to analyze perceptions and recommendations from stakeholders on the effectiveness of fall detection systems for older adults, aside from any additional technological solutions they may use within their activities of daily living (ADLs)	Mixed	Colombia	older adults, informal caregivers	This study included 25 adults from Colombia, comprising six (24%) older adults (n = 2, 33.33%, women and n = 4, 66.67%, men) aged 65 years and over, and 19 (76%) adults (n = 10, 52.63%, women and n = 9, 47.37%, men) aged 25 to 60 years. From the aforementioned study population, the educational level of surveyed stakeholders (n = 14) is highly educated, the vast majority holding a postgraduate degree (92.86%), and the remainder being graduate professionals (7.14%), while the educational level of the interviewed stakeholders (n = 11) ranges from high school (9.09%), to graduate professional (36.36%), and postgraduate (54.55%). Survey participants were based in Bogotá (n = 4) 16.67%, Popayán (n = 1) 4%, Medellín (n = 2) 8%, and Tunja (n = 1) 4%, while the interviews were conducted in Cali (n = 13) 52%, Firavitoba (n = 2) 8% and Tuluá (n = 1) 4%, overwhelmingly in urban areas (n = 23) 92%, with those in	Fall Detection Systems for Older Adults	. The response from the informal caregivers and older adults is that they are unaware of FDSs. In fact, informal caregivers typically described the functions of FDSs based on their conjectures upon hearing the device name.	When asked if the older adult would like to use an WFDS, both informal caregivers and older adults responded in the affirmative. One reason given was the experience during a fall. For example, an older adult reported that he fell in their house, fell unconscious, and no one arrived to help them until he recovered consciousness. However, informal caregivers expressed obstacles such as the need to explain and justify the use of the system to a significant extent because older adults may feel that they are losing their independence.				[illegible]					N
Perceptions of patients and nurses regarding the use of wearables in inpatient settings: a mixed methods study	2022	INFORMATICS FOR HEALTH & SOCIAL CARE	Patel, V and Moosa, S and Sundaram, S and Langer, L and MacMillan, TE and Cavalcanti, R and Cram, P and Gunaratne, K and Bayley, M and Wu, R	10.1080/17538157.2022.2042304	Wearable devices for hospitalized patients could help improve care. The purpose of this study was to highlight key barriers and facilitators involved in adopting wearable technology in acute care settings using patient and clinician feedback.	Mixed	Canada	in-patients	Sex (women) 23 (59%)<br>Length of stay in days 11 days<br>Mean days wearable was worn (SD) 2.9 (1.4)<br>Discharge status/location<br>Home 24 (62%)<br>Home with community care support 7(18%)	the use of wearables in inpatient settings				[illegible]					While many patients were initially motivated to participate in the study to contribute to research, patients completing the study reported that the wearable enhanced their self-monitoring and increased their physical activity. The wearable provided patients with an opportunity to monitor and learn more about their health information, thus contributing to their self-efficacy. Many believed this experience enriched their experience as they welcomed the novelty and became more curious to track their activity during their hospital stay.Patients understood that the data from the wearable can contribute to a clinician's treatment and management plans and can overall allow clinicians to enhance patient health outcomes. Clinician endorsement of the wearable could be a driving factor to its use in	Device functionality<br>Patients were also concerned about the Fitbit's accuracy, sensors, activity detection, and the enabling of notifications. With regard to accuracy, patients were concerned about the Fitbit's ability to compete with hospital monitors to provide readings with a similar accuracy. Notifications had been disabled on the devices; however, patients often thought they might have been useful for alerts or perhaps activity reminders.	N
Examining individuals' adoption of healthcare wearable devices: An empirical study from privacy calculus perspective	2016	INTERNATIONAL JOURNAL OF MEDICAL INFORMATICS	Li, H and Wu, J and Gao, YW and Shi, Y	10.1016/j.ijmedinf.2015.12.010	This paper explores the predictors of individuals' adoption of healthcare wearable devices.	Quantitative	China	General public	[illegible]<br>Percentage<br>Gender Female 172 51.65%<br>Male 161 48.35%<br>Age 25 or under 116 34.83%<br>26–35 90 27.03%<br>36–45 83 24.92%<br>46 or older 44 13.22%<br>Education Bachelor 154 46.25%<br>Master 145 43.54%<br>Doctoral 34 10.21%<br>Internet experience Less than 1 year 14 4.20%<br>1–2 year 56 16.82%<br>3–5 year 124 37.24%<br>More than 5 year 139 41.74%<br>Health status Healthy 185 55.56%<br>Poor health 102 30.63%<br>Unhealthy 46 13.81%	healthcare wearable devices					[illegible]						N
Utilization of Patient-Generated Data Collected Through Mobile Devices: Insights From a Survey on Attitudes Toward Mobile Self-Monitoring and Self-Management Apps for Depression	2019	JMIR MENTAL HEALTH	Hartmann, R and Sander, C and Lorenz, N and Bottger, D and Hegerl, U	10.2196/11671	The aim of this survey was to provide descriptive data upon usage and anticipated usage of self-monitoring and self-management of depression and preferences of potential users in terms of documented parameters and data-sharing options.	Quantitative	Unspecified	depression patients	Characteristics Statistics<br>Gender, n (%)<br>Female 514 (76.9)<br>Male 154 (23.1)<br>Age (years), mean (SD) 39.31 (12.857)<br>Occupation, n (%)<br>Employed 334 (50.0)<br>Self-employed 26 (3.9)<br>In education: completing education 87 (13.0)<br>Taking care of household 27 (4.0)<br>Retired 88 (13.2)<br>Unemployed 70 (10.5)<br>Other 36 (5.4)<br>Relationship, n (%)<br>Single 262 (39.2)<br>Married 347 (52.0)<br>Other 59 (8.8)	Mobile Self-Monitoring and Self-Management Apps for Depression	Within the affected sample, a proportion of 81 individuals (12.1%, 81/668) reported to have installed and used an MSSD at least once in their life. Moreover, 24 individuals installed an MSSD but did not use it (3.6%, 24/668). The majority of the subsample of affected individuals (78.0%, 521/668) stated to have never used any respective app.						[illegible]		Analysis showed that people want personally relevant information and feedback	but also focus on handling sensitive data	Within the last 3 years, UH was an advisory board member for Lundbeck, Takeda Pharmaceuticals, Servier and Otsuka Pharma. UH was a consultant for Bayer Pharma and a speaker for Bristol-Myers Squibb, Medice Arzneimittel, Novartis, and Roche Pharma. The remaining authors declare that the research was conducted in the absence of any commercial or financial relationships that could be construed as a potential conflict of interest.

Risk assessment on the construction site with the use of wearable technologies	2021	AIN SHAMS ENGINEERING JOURNAL	AL-Sahar, F and Przegalinska, A and Krzeminski, M	10.1016/j.asej.2021.04.006	As wearable technologies become more popular and affordable, they can be applied to monitor the locations of machinery and the staff on the construction site. Based on that risky configurations can be detected, and relevant actions can be undertaken to protect working people from any injuries. Different types of wearable technologies are presented together with the discussion of their applicability in monitoring in the construction sector. Moreover, the survey is done to better recognition of health risks. Based on that the recommendations are formulated for future applications of wearable technologies as an innovative tool of risk management in construction.	Quantitative	United Arab Emirates	people working at counstruction sites	The experts were from several fields of construction projects, Civil engineers', planning Engineers, Architect, and surveyors, etc.	Risk assessment on the construction site with the use of wearable technologies			The first meeting results produce shows that the construction sites have many issues that must take it in safety consideration and find the proper technique to track it.									N
Anxious or empowered? A cross-sectional study exploring how wearable activity trackers make their owners feel	2019	BMC PSYCHOLOGY	Ryan, J and Edney, S and Maher, C	10.1186/s40359-019-0315-y	This study explores wearable users' affective responses to their devices and how these relate to personality traits and individual differences.	Quantitative	Australia	General public	Participants were 200 current users and 37 previous users of a wearable activity tracker (see Table Table1).1). The majority of participants (72%) were female and had completed a university degree (60%). Participants' average age was 33.1 (SD 12.4) and participants checked the data on their device 6.2 (SD 4.6) times on average per day.	wearable activity trackers			Two-way ANOVA results indicated that affect was significantly associated with wearing conditions (previous or current user; during wear and when unable to wear), $F (3, 460) = 182.152$, $p = < 0.001$. There were statistically significant associations between affect scores with current or previous wear status ($p = 0.036$), wearing or unable to wear status ($p = 0.004$) and an interaction between current or previous wear status and wearing or unable to wear status ($p = 0.004$). This interaction effect is illustrated in Fig. 1. Previous and current users' self-reported affect scores followed a similar trajectory, whereby affect was higher (more positive) during wear but declined significantly when not wearing (see Fig. Fig.11).	Results suggest that using a wearable is a positive experience for users with little risk of negative psychological consequences. Whilst experiences of negative affect were uncommon, individuals low in conscientiousness or openness to experience are at greater risk of negative affect and all users may experience negative affect such as anxiety or frustration when prevented from wearing their device. Findings contribute to mounting evidence of wearables' safety and appeal as health behaviour tools and highlight the importance of examining psychological and experiential aspects of activity tracking.								N
A Kinect and Inertial Sensor-Based System for the Self-Assessment of Fall Risk: A Home-Based Study in Older People	2016	HUMAN-COMPUTER INTERACTION	Ejupi, A and Gschwind, YJ and Valenzuela, T and Lord, SR and Delbaere, K	10.1080/07370024.2015.1085309	The aims of this study were to (a) empirically examine the feasibility of the iStoppFalls (Information and communications technology-based System to Predict & Prevent Falls) assessment, a Kinect and inertial sensor-based test for regular and unsupervised fall risk assessments at home, (b) investigate the experience of older adults with this home-based self-assessment, and (c) make recommendations for future assessments.	Mixed	Australia, Germany, and Spain	Older adults	Ten participants from Sydney were invited to participate in the qualitative study. All agreed to be interviewed. The participants were in average 78.1 ± 6.1 years old; seven (70%) were female. Half of the interviewees performed the assessment at least twice or more on their own, and half reported a fall in the past 12 months prior to the study. The computer literacy was high; eight (80%) participants owned a computer and used it in average 3.0 ± 0.8 hours per week.	A Kinect and Inertial Sensor-Based System for the Self-Assessment of Fall Risk			Most of the participants considered the experience with the iStoppFalls assessment as positive.	the majority of participants stated they were interested in that information, especially the participants who had already experienced a fall.	In general, participants reported no issues with the five-time-sit-to-stand test. Only one participant explained that she had knee problems and did not like to perform the test.<br><br>The exergame approach with full-body interaction was considered as very interesting. No participants had a problem in understanding the concept of controlling the avatar. However, sometimes the avatar irritated participants with unusual poses which resulted from problems in detecting the correct movements of participants. the comments can be categorized in usability and hardware issues. One participant who experienced hardware issues emphasized the need for reliable equipment	the iStoppFalls assessment have been observed. First, participants had a general interest in their health and fall risk. Others mentioned that they were interested in their health and fall risk to improve on the deficits. Second, participants wanted to help and please the researchers.<br><br>Normally, fall risk assessments are conducted in a clinical environment supervised by a health professional. The home-based assessments were perceived as more natural, less time consuming, and more convenient.	In general, the participants preferred simple visualizations and written text. Participants discussed the importance of the accuracy and reliability of the information given in the feedback and how the information had an impact on their motivation to be more active.	The participants who did not perform the assessment explained that they were already aware of their risk of falling and therefore careful. Further, they mentioned technical issues, personal reasons (i.e., health problems or home situations), or that they did not know about that functionality of the system as the main reasons that prevented them from performing the assessment.			Safety and Support<br>No adverse events have been reported in this study.	UNDECLARED
Digital Health Intervention for Asthma: Patient-Reported Value and Usability	2018	JMIR MHEALTH AND UHEALTH	Merchant, R and Inamdar, R and Henderson, K and Barrett, M and Su, JG and Riley, J and Van Sickle, D and Stempel, D	10.2196/mhealth.7362	The aim of this study was to evaluate patient satisfaction, perception of usability and value, and desire to continue after 12 months of using a digital health intervention to support asthma management.	Quantitative	USA	asthma patient	Among the participants who completed the survey (N=89), the average and median ages were 42.7 and 45 years, respectively; 85% (76/89) of participants were aged older than 18 years, 15% (13/89) were aged between 12 and 17 years, and 64% (57/89) of participants were female. Participants used a variety of device types to access the digital platform, including smartphones (49%, 44/89; 27%, 24/89 iOS and 23%, 20/89Android) and hub base stations for those without smartphones (51%, 45/89). In addition, 74% (66/89) of participants had private insurance and 26% (23/89) had public insurance. At the start of the study, 30% (27/89) of participants were well controlled, 61% (54/89) were not well controlled, and 9% (8/89) had an unknown control status.	Digital Health Intervention for Asthma			Participants reported their degree of satisfaction with the inhaler sensor device, with 79% (70/89) of respondents reporting "very satisfied," 20% (18/89) reporting "somewhat satisfied," and 1% (1/89) reporting "not at all satisfied" (Figure 2). The final question of the survey asked participants whether they would like to continue using the digital intervention: 72% (64/89) of the participants reported that they were interested in continuing to use the sensor and platform beyond the study. There were no significant differences among those participants who were interested in continuing or those who were not based on a participant's device type, age, gender, insurance type, syncing history, or asthma control at intake (see Multimedia Appendix 5).	There were no significant differences in responses to this question across participants' device type, age, sex, insurance, asthma control at intake, or syncing history (see Multimedia Appendix 2).	A total of 37 participants (40%, 37/89) stated the inhaler device was "easy," including 3 subthemes of "easy to use" (32%, 29/89), "easy to maintain" (6%, 6/89), and "easy to understand" (2%, 2/89). Moreover, 23 participants (26%, 23/89) described the size of the device as "small" and "compact" (Table 3). Of the subthemes relating to the size of the device, 10 participants (11%, 10/89) specified that it did not "obstruct" or "interfere" with their use of their inhaler and "was not bulky." Furthermore, 17 participants (19%, 17/89) described the sensor's convenience, with the 2 subthemes of the device being "portable" and functioning well.		recommendations for sensor improvements. Of the participants who reported specific ideas for improvements (44%, 39/89), the most frequent response was for a longer battery life (20%, 18/89). Participants also reported wanting a smaller size device (13%, 12/89). The remaining participants reported wanting a more secure fit (4%, 4/89) or recommended additional features such as adding a dose counter (6%, 5/89).Some participants reported not learning anything from the reports: 16 participants (18%, 16/89) reported "none," and 3 participants (3%, 3/89) reported not learning anything because they were already well controlled.<br><br>Participants were asked to provide suggestions for improving the reports or platform. A total of 59 participants (66%, 59/89) reported no improvements were needed, and 11 participants (12%, 11/89) responded with "not sure" or "no comment." The remaining responses (21%, 19/89) offered specific suggestions including more educational content on how to improve asthma, information about how to subsidize medication costs, improvements in the app login process, additional customization options for how to receive reports, changing the cadence of the reports, and extending the sensor battery life.			commonly identified triggers, documented across 79% (42/53) of the participants who identified new triggers A second primary theme focused on improved self-awareness. A total of 19 participants (21%, 19/89) specifically reported learning from monitoring the timing, location, and frequency of their medication use. Moreover, 10 participants (11%, 10/89) described learning strategies for managing their asthma such as taking their controller medications and avoiding specific triggers. Furthermore, 6 participants (7%, 6/89) reported that they discovered they were not as in control of their asthma as they had believed or they confirmed that their treatment plan helped them stay controlled. In addition, 5 participants (6%, 5/89) learned that they are accountable for their self-management and have the capacity to control their asthma. Participants could opt to discuss their data with their providers; 46% (41/89) of the participants reported that they had conversations with their providers about their data.		DVS, KH, MB, JR, and DS are Propeller Health employees and receive salary and stock options. RM, RI, and JGS report no conflicts.
A Virtual Reality Lower-Back Pain Rehabilitation Approach: System Design and User Acceptance Analysis	2015	UNIVERSAL ACCESS IN HUMAN-COMPUTER INTERACTION: ACCESS TO LEARNING, HEALTH AND WELL-BEING, UAHCI 2015, PT III	Su, WC and Yeh, SC and Lee, SH and Huang, HC	10.1007/978-3-319-20684-4_37	In this study, a VR-based LBP rehabilitation system utilizing wireless sensor technologies to assist physiotherapists and patients in undertaking three stages of rehabilitation exercises for low back health is proposed. A total of twenty LBP patients have been recruited for this study, and a user acceptance of technology questionnaire is used to investigate the effectiveness and efficiency of the system proposed.	Quantitative	Taiwan	patients with Lower-back pain rehabilitation		A Virtual Reality Lower-Back Pain Rehabilitation Approach			participants and indicates that most participants are satisfied with this VR system. Specifically, the feedback from visual, auditory responses by the system and information on the screen are a useful resource to guide the system users to complete their tasks. Furthermore, patients also feel that they are interested in the design of the avatar and projected information on the screen and this also encourages them to step into a new form of training. To that end, participants have readiness to recommend this system to their friends and relatives when these individuals do have similar rehabilitation requirements.				In the meantime, participants also look forward to the addition of a serious game design to be added and implemented within these rehabilitation exercises.					UNDECLARED
Contactless remote monitoring of sleep: evaluating the feasibility of an under-mattress sensor mat in a real-life deployment	2022	HEALTH SYSTEMS	Sadek, I and Abdulrazak, B	10.1080/20476965.2022.2072777	In this paper, we first discuss commercially-available non-wearable devices that measure sleep, in which we highlight the features associated with each device, including sensor type, interface, outputs, dimensions, power supply, and connectivity. Second, we evaluate the feasibility of a non-wearable device in a free-living environment.	Quantitative	Singapore	Older adults		Contactless remote monitoring of sleep under-mattress sensor mat					In contrast, they were more interested in non-intrusive sensors like the proposed sensor mat. For these reasons, we implemented a non-intrusive system for the sleep monitoring process. Another issue to consider was offline storage; it was important to keep a local copy of the data on the built-in micro-SD card to avoid data loss. For some reason, the Internet could stop working, and sometimes the resident could unplug the router for cleaning or other housework. The 4 GB internal storage could keep the binary files for a few days; hence data could be retrieved again.			They were worried about recharging the device, forgetting to wear the device. This situation may not necessarily hold for all age groups, but it may happen due to ageing or age-related diseases such as dementia. Additionally, residents and caregivers were against the use of microphones and/or cameras.				N
Privacy Attitudes among Early Adopters of Emerging Health Technologies	2016	PLOS ONE	Cheung, C and Bietz, MJ and Patrick, K and Bloss, CS	10.1371/journal.pone.0166389	This study sought to understand privacy attitudes of early adopters of emerging health technologies.	Qualitative	USA	Older adults	Descriptive information of study participants.<br>Participant Characteristics PGP (N = 7) HDE* (N = 11)<br>Age 18–25 0 9.7%<br>26–35 0 26.6%<br>36–45 42.9% (N = 3) 23.0%<br>46–55 0 21.6%<br>56–65 42.9% (N = 3) 13.9%<br>65+ 14.3% (N = 1) 5.2%<br>Gender Male 71.4% (N = 5) 34.5%<br>Female 28.6% (N = 2) 65.2%<br>Race/Ethnicity White/Caucasian 85.7% (N = 6) 79.3%<br>Other 14.3% (N = 1) 20.7%<br>Education Less than a 4-year college degree 14.3% (N = 1) 9.6%<br>4-year college degree 42.9% (N = 3) 29.2%<br>Postgraduate/professional degree 42.9% (N = 3) 61.1%<br>Marital Status Yes 85.7% (N = 6) 65.6%<br>No 14.3% (N = 1) 34.4%<br>Household Income Less than $50,000	genome sequencing and health devices and apps										respondents generally believe the benefits of contributing personal data to science outweigh the privacy risks.	Key findings have been organized into four themes: (1) personal data privacy is important but difficult to achieve; (2) respondents want control over their personal information; (3) discrimination based on personal data is a significant concern	N
Feasibility and Acceptability of a Mobile-Based Emotion Recognition Approach for Bipolar Disorder	2021	INTERNATIONAL JOURNAL OF INTERACTIVE MULTIMEDIA AND ARTIFICIAL INTELLIGENCE	Daus, H and Backenstrass, M	10.9781/ijimai.2021.08.015	We developed an emotion-sensitive system that analyzes the verbal and facial expressions of bipolar patients in regard to their emotional cues. In this article, preliminary findings of a pilot study with five bipolar patients with respect to the acceptability and feasibility of the new approach are presented and discussed.	Mixed	Germany	patient with bipolar disorder	Four participants, according to DSM-V, could be diagnostically categorized into bipolar I disorder, the fifth person into bipolar II disorder. The participants (between 24 and 51 years old) had an average age of 39.40 years (SD = 9.94).	Feasibility and Acceptability of a Mobile-Based Emotion Recognition Approach for Bipolar Disorder			Thus, from the acceptability point of view, the ambulatory long-term assessment of emotional expressions in bipolar disorder should be possible.				The mentioned conditions, like an easier usage experience (e.g., without microphone or holder) or automatic reactions towards the told stories, matched the original concept of the assistance system [34]. In accordance with the process of development, these aspects had not been implemented within the study setting. They should, however, be technically convertible in the future.					N
Beyond novelty effect: a mixed-methods exploration into the motivation for long-term activity tracker use	2019	JAMIA OPEN	Shin, G and Feng, YY and Jarrahi, MH and Gafinowitz, N	10.1093/jamiaopen/ooy048	Activity trackers hold the promise to support people in managing their health through quantified measurements about their daily physical activities. Monitoring personal health with quantified activity tracker-generated data provides patients with an opportunity to self-manage their health. Many have been conducted within short-time frames; makes it difficult to discover the impact of the activity tracker's novelty effect or the reasons for the device's long-term use. This study explores the impact of novelty effect on activity tracker adoption and the motivation for sustained use beyond the	Mixed	USA	General public	Out of 23 participants, 16 (n = 16) were female.	activity tracker			Whereas some participants were naturally more curious about new digital technology and its functionality (technophiles), others considered themselves only interested in the direct utility of digital devices and their relevance to personal lifestyle. The sample also included a few individuals who were indifferent or even skeptical about their Fitbit device, and used it (often for a short period) only because they had received it as a gift.			The qualitative analysis of the interview data identified personal motivation, social motivation, and gaming motivation as 3 primary factors that encouraged participants' long-term device use beyond the novelty period.						N
A compliance real-time monitoring system for the management of the brace usage in adolescent idiopathic scoliosis patients: a pilot study	2021	BMC MUSCULOSKELETAL DISORDERS	Zhu, C and Wu, Q and Xiao, B and Wang, JH and Luo, C and Yu, Q and Liu, LM and Song, YM	10.1186/s12891-021-03976-5	Patient compliance is essential to the effectiveness of brace treatment for adolescent idiopathic scoliosis (AIS) patients. The objective of this study is to describe and evaluate a compliance real-time monitoring system of the brace usage in AIS patients.	Quantitative	China	adolescent idiopathic scoliosis patients	females) were included in the final analysis. The mean patient age was 12.4 ± 1.5 years (range: 10 ~ 15 years). The follow-up period of all patients was 6 months. The mean baseline brace force was 1.23 ± 0.28 N (range: 0.82 ~ 1.95 N). The mean measured force was 0.79 ± 0.29 N (range: 0.25 ~ 1.60 N). The mean quality compliance was 64.8 ± 22.2% (range: 29.2 ~ 127.7%). The prescribed time of all patients was 23 h. The mean measured time was 14.1 ± 2.9 h (range: 6.8 ~ 18.7 h). The mean quantity compliance was 61.3 ± 12.6% (range: 29.8 ~ 81.6%).	A compliance real-time monitoring system for the management of the brace usage in adolescent idiopathic scoliosis patients			In this study, 96.4% (27/28) patients were satisfied with the use of the monitoring system, among whom 21.4% (6/28) are very satisfied and 75.0% (21/28) are somewhat satisfied.									N
Understanding and meeting injection device needs in multiple sclerosis: a survey of patient attitudes and practices	2011	PATIENT PREFERENCE AND ADHERENCE	di Cantogno, EV and Russell, S and Snow, T	10.2147/PPA.S14903	All established disease-modifying drugs for multiple sclerosis require parenteral administration, which can cause difficulties for some patients, sometimes leading to suboptimal adherence. A new electronic autoinjection device has been designed to address these issues. Patients with relapsing multiple sclerosis currently receiving subcutaneous or intramuscular interferon beta-1a, interferon beta-1b, or glatiramer acetate completed an online questionnaire (July 4-25, 2008) that surveyed current injection practices, experiences with current injection methods, and impressions and appeal of the new device.	Quantitative	Canada, France, Germany, Italy, Spain, and the US	Patients with relapsing multiple sclerosis	survey in Canada (n = 35, 8%), France (n = 35, 8%), Spain (n = 45, 11%), Germany (n = 50, 12%), Italy (n = 47, 11%), and the US (n = 210, 50%). Patient demographic and clinical characteristics are presented in Table 2. Respondents had been receiving injected therapy for a mean of 4.2 years. The most common current therapy was subcutaneous IFN beta-1a, which was being received by 189 respondents (45%). A further 85 respondents (20%) were currently receiving treatment with intramuscular IFN beta-1a, 60 (14%) with subcutaneous IFN beta-1b, and 88 (21%) with glatiramer acetate.	injection device needs in multiple sclerosis			Approximately one-third (35%) of respondents described themselves as early adopters of new technologies, while 57% described themselves as cautious adopters, and only 8% as late adopters.Approximately two-thirds (65%) of patients found the new device "very appealing" (score of 8–10), with a higher proportion among current users of subcutaneous IFN beta-1a (73%) and IFN beta-1b (70%) than among users of intramuscular IFN beta-1a (54%) and glatiramer acetate (56%) favoring the new device. Overall, the new device was rated as moderately appealing (score of 3–7) by 30% of patients, and not at all appealing (score of 0–2) by 5%.		Most respondents were very comfortable in general with new technologies. On a scale of 0–10 (0 = not at all comfortable, 10 = very comfortable), the mean score was 8.0 (median 8.0, range 0–10), and 69% of patients scored between 8 and 10; 29% were somewhat comfortable (score of 3–7), and only 2% were uncomfortable (score of 0–2).	When patients were asked to rate their satisfaction with their current injection method on a scale of 0–10 (0 = not at all satisfied, 10 = very satisfied), the mean score was 6.7 (median 7.0, range 0–10). Overall, 40% of respondents were "very satisfied" (score of 8–10) with their current injection method (Table 3), with a lower percentage among users of vials and syringes (23%) than among users of prefilled syringes (39%) or autoinjectors (46%).				Overall, 96% of respondents identified a benefit that would encourage them to ask their nurse or doctor about the new device. The most appealing benefits were the ability to tailor injection settings (such as speed of injection and depth of needle insertion) to individual needs (cited by 39% of respondents), and the dosing log that allows reliable monitoring of regular dosing (cited by 38%, Figure 3).	Overall, 23% of respondents considered that the device had no drawbacks. The most frequently stated drawback was fear that the device might malfunction, which was cited by 37% of respondents.	UNDECLARED
Evaluation of Patient Willingness to Adopt Remote Digital Monitoring for Diabetes Management	2021	JAMA NETWORK OPEN	Oikonomidi, T and Ravaud, P and Cosson, E and Montori, V and Tran, VT	10.1001/jamanetworkopen.2020.33115	The study analyzes why patients adopt remote digital monitoring (RDM) for diabetes.	Quantitative	Unspecified	diabetes patients	Characteristic<br>Participants, No. (%)<br>(N = 1010)a<br>Age, median (IQR), y 51 (37-63)<br>Gender<br>Men 394 (39)<br>Women 572 (57)<br>Prefers to self-describe 44 (4)<br>Country of residence<br>France 360 (36)<br>Canada 212 (21)<br>United States 138 (14)<br>Other 300 (30)	Remote Digital Monitoring for Diabetes Management							participants would adopt RDM only if it was much more effective at reducing hypoglycemic episodes<br>compared with their current monitoring; in 1835 assessments (65%), participants would adopt RDM<br>if was just as or somewhat more effective. The main factors associated with required effectiveness<br>were food monitoring ($\beta = 0.32$; SE, 0.12; $P = .009$), real-time telefeedback by a health care<br>professional ($\beta = 0.49$; SE, 0.15; $P = .001$), and perceived intrusiveness ($\beta = 0.36$; SE, 0.06; $P < .001$).<br>Minimum required effectiveness varied among participants; 34 of 36 RDM scenarios (94%) were<br>simultaneously required to be just as or less effective by at least 25% of participants and much more<br>effective by at least 25% of participants.					Mrs Oikonomidi reported receiving a fellowship from the doctoral network of the École des Hautes Etudes en Santé Publique No other disclosures were reported.
Requirements for Unobtrusive Monitoring to Support Home-Based Dementia Care: Qualitative Study Among Formal and Informal Caregivers	2021	JMIR AGING	Wrede, C and Braakman-Jansen, A and Van Gemert-Pijnen, L	10.2196/26875	The objective of this study was to explore the expected benefits, barriers, needs, and requirements toward unobtrusive in-home monitoring from the perspective of formal and informal caregivers of community-dwelling people with dementia.	Qualitative	Netherlands	patient with dementia and caregivers	Age of caregiver (years), mean (SD) 60.5 (13.1)<br>Age of care recipient (years), mean (SD) 82.6 (5.5)<br>Active as caregiver (years), mean (SD) 10.5 (10.1)<br>Time since diagnosisa (years), mean (SD) 4.6 (3.5)<br>Gender, n (%)<br>Female 16 (84)<br>Male 3 (16)<br><br>Age (years), mean (SD) 39.3 (11.3)<br>Care contact hours per week provided to community-dwelling people with dementia, mean (SD) 20.3 (6.3)<br>Work experience in current home care profession (years), mean (SD) 15.4 (9.1)<br>Gender, n (%)<br>Female 15 (94)<br>Male 1 (6)	Requirements for Unobtrusive Monitoring to Support Home-Based Dementia Care						The top 5 monitoring goals seen as most useful by both groups included fall detection and prevention, and monitoring day and night rhythm, personal hygiene (eg, dressing, grooming, bathing, and toileting), nocturnal restlessness, and eating and drinking behavior. Monitoring goals rated as nonrelevant by both formal and informal caregivers included leaving the house (the action of doing so), social interaction (indoors), and telephone use (frequency of use). In general, both groups showed comparable results, although informal caregivers appeared to be less interested in monitoring eating and drinking behavior, nocturnal restlessness, and walking distance/speed compared with formal caregivers.	Functionality Such a coaching function could aid in tasks that typically require learning and decision making. As described by participants, assistance should focus on maintaining day structure (eg, reminders about certain times for eating, sleeping, or medication intake) or, based on the level of inactivity, providing suggestions for exercising or taking a walk.<br>Furthermore, as formal caregivers are interested in deviations over time, with a special interest in detecting periods deviating from the expected disease progression, the system should enable recognizing patterns and deviations, and provide the possibility to analyze on a weekly or monthly basis, thereby functioning as an analysis tool.<br><br>Moreover, the system should be capable of autonomously detecting emergency situations and sending alarms to the most suitable caregiver(s) in charge, thereby resolving the problem of current (mostly wearable) safety technology that the patient might forget to wear or might be out of reach in emergency situations. To prevent false alarms, the system should open a communication channel to the patient first to check the need to intervene. However, the system should generally be context-aware instead of applying generic threshold values in determining alarming deviations.<br><br>Furthermore, as indicated by participants, the system must offer fine-grained data-sharing options for sharing care-relevant information with			Cross-checking self-care information<br>Better self-care surveillance<br>In-person control visits<br>Extended independent living<br><br>Safety at home<br>Detecting and removing factors that hinder independence<br>Helpful for initiating extra care needed<br>Decision support for transition to residential care<br>Objective communication and substantiation<br><br>Supporting objective communication around patient's situation<br>Substantiating diagnostics and indications<br>Prevention and proactive measures<br><br>Responding more quickly to care needs to prevent health risks<br>Improved insight into inhibiting and activating factors of patient's behavior/ mood<br>Theme brought forward by only informal caregivers<br><br>Emotional reassurance	Information overload<br><br>Risk of feeling monopolized by the system<br>(Un)certainty of whether to respond to monitoring information<br>Risk of disturbing daily work routines<br><br>Privacy concerns<br>Risk for misuse of monitoring data<br>Risk of losing control about data sharing<br>Tradeoff privacy infringement versus extended independence<br>Ethical concerns: dehumanizing care<br><br>Risk of replacing human contact by technology<br>Risk of undermining the formal caregiver's professional view	N
Needs of Lung Cancer Patients Receiving Immunotherapy and Acceptance of Digital and Sensor-Based Scenarios for Monitoring Symptoms at Home-A Qualitative-Explorative Study	2022	INTERNATIONAL JOURNAL OF ENVIRONMENTAL RESEARCH AND PUBLIC HEALTH	von Kutzleben, M and Galuska, JC and Hein, A and Griesinger, F and Ansmann, L	10.3390/ijerph19159265	The development of immunotherapy in the treatment for lung cancer has changed the outlook for both patients and health care practitioners. However, reporting and management of side effects are crucial to ensure effectiveness and safety of treatment. The aim of this study was to learn about the subjective experiences of patients with lung cancer receiving immunotherapy and to explore their potential acceptance of digital and sensor-based systems for monitoring treatment-related symptoms at home.	Qualitative	Germany	Lung Cancer Patients	Participants N = 21<br>Sex (%) 14 male (67%)<br>7 female (33%)<br>Age (in years) Median 65<br>Range 50–81<br>Histological type Adenocarcinoma 16<br>Squamous cell carcinoma 4<br>Small cell carcinoma 1<br>Stage II 1<br>III 3<br>IVA 7<br>IVB 9<br>Stage unclear 1<br>Therapy goal Curative 3<br>Palliative 17<br>Unclear 1<br>Therapy Scheme Mono-IT * 2<br>Mono-IT *, status post RT/CT/RCT 6<br>Combination treatment * (IT + RCT/CT, vaccination if applicable) 4<br>Status post IT + RCT * 1<br>RET-Inhibitor (BLU−667) 1<br>RCT 1<br>Status post RCT, RT and/or surgery, IT-	Digital and Sensor-Based Scenarios for Monitoring Symptoms at Home	Most of the participants were unfamiliar with digital and sensor technology approaches in the health context.		Initially, the interviewees expressed an intuitively negative attitude and discomfort towards the idea of linking one's own body or living environment with technical systems. Ambient systems in particular were viewed with skepticism, due to their potential for ubiquitous observation of the entire household. Potential benefits were not seen at first sight and the increasing mechanization in this area is perceived as "exaggerated" (13mT, line 816). In general, the interviewees believed that they could report symptoms and complaints themselves or considered such technical solutions only useful for people living alone. With a more intensive thematic discussion, this attitude tends to weaken in the course of the interviews.				Next to feedback from the system to the patient on how to react adequately, interviewees suggested to implement an emergency call function and found it essential that the technical monitoring included a solution for direct communication with their health care practitioners. Overall, the expressed needs regarding monitoring systems varied greatly depending on the individual situation and symptoms experienced in the past. Monitoring was perceived as particularly useful for symptoms that had been experienced in the past. Still, most participants felt that benefits of monitoring would not (yet) be great enough at the time of the interview, although stating that this could change in the future if their own health condition worsened. In general, a sensitive detection of an incipient emergency situation was seen as much more useful than an undirected continuous monitoring of the patient at home:	Lack of assumed usefulness was the main argument against it.Another argument against a technical monitoring at home were possible disadvantages of using monitoring technology. Some participants worried about being disturbed by the technical devices in their domestic environment due to sounds, exposure to radiation, and hassle if data had to be provided proactively by the user (e.g., in the case of online reporting of symptoms).		An improved assessment of symptoms and side effects by using a technical monitoring system was perceived as potentially beneficial.<br>Participants expressed different attitudes towards a system that would be able to submit information to their health care practitioners. Some participants found it promising that technical solutions might enable them to receive out-patient cancer treatment while improving quality and safety of the therapy. Yet, others were concerned about their autonomy. A few interviewees assumed that monitoring throughout the night could be of extra benefit, as some symptoms (e.g., nocturnal coughing) might pass unnoticed while asleep. Furthermore, a monitoring system should be able to register changes in context and activity, and effects on measured parameters, as temperature inside their homes or outside during sport activities.<br><br>Another assumed benefit of technical monitoring was support in challenging situations. Related to the difficulties in deciding whether a perceived indisposition might be harmless or serious, participants saw a potential to prevent adverse events or an acute health crisis.	A few patients were concerned that such systems might place the disease even more in the center of their lives, therefore making it difficult putting the disease and related threats aside in phases with little symptoms. Additionally, interviewees expressed the latent fear that their autonomy and self-determination could be violated by digital and sensor-based monitoring systems.<br><br>3.4.3. Sub-Category: Concerns Related to Privacy and Data Protection<br>Issues of data protection and privacy turned out to be relevant and sensitive aspects for potential users of home-based technical monitoring systems. Interviewees expressed various concerns, despite being generally willing to take certain risks if the medical	N

Experiences of Using a Continuous Glucose Monitoring System in Children-A Descriptive Study with Parents in the Republic of Georgia	2021	HEALTHCARE	Kheladze, N and Kristensson, L and Johansson, A and Crang-Svalenius, E and Ivarsson, B	10.3390/healthcare9111556	The purpose of this study was to describe parents' experiences during their daily lives and support needs when a child uses a Flash Glucose Monitoring system (FGM).	Qualitative	Republic of Georgia	diabetes children and parents	Men 3 (15) Boys 9 (45) (median (IQR)) 17 (6–62.25)<br>Women 17 (85) Girls 11 (55) (median-year & month) 1 year 5 month<br>min-max<br>4–96<br>Age (year) Age (year, (n/%)) Libre experience<br>mean ± SD 38 ± 4 0–3 2 (10) (median (IQR))<br>7 (3.25–30)<br>min–max 30–45 4–7 7 (35) min–max 1–72<br>8–12 7 (35)<br>12–16 4 (20)	a Continuous Glucose Monitoring System in Children								Problems/Weakness According to the parents, very few of the children experienced any discomfort using the device. A small number had slight skin irritation, but one specific problem mentioned by many parents was keeping the device in place when doing physically energetic activities, as the protection tape tended to loosen. However, this was not considered a major problem, and overall the device together with the protection tape was considered much more hygienic than frequently taking blood samplings. Otherwise, there was no discomfort described. "My son is an active rugby player and even though we try to attach it with extra measures it often gets removed during exercise or games".<br><br>Showering and bathing were not considering as causing any problems, but when swimming, the device could need extra adhesive material. Some parents had experienced malfunction episodes with the device, but nothing that seriously jeopardized the medical treatment. In these cases, the manufacturer replaced the malfunctioning device free of charge.<br><br>Some of the parents described initial difficulties pairing the FGM device with a mobile phone, but these difficulties were perceived as primary inexperience on the part of the parents.<br><br>Economic Aspects Economic concerns regarding the device were a		"Advances in technology significantly improved everyday life", from two categories; Improvements in quality of life & Elements of challenges,		N
Understanding older adults' attitudes toward mobile and wearable technologies to support health and cognition	2022	FRONTIERS IN PSYCHOLOGY	Fowe, IE and Boot, WR	10.3389/fpsyg.2022.1036092	In an online survey, we assessed community-dwelling older adults' (N = 92) attitudes towards the use of wearable and mobile technologies for (1) predicting cognitive decline, (2) assisting with adherence to healthy activities, and (3) collecting self-report data to understand current and predict future health states.	Quantitative	USA	Older adults	Their average age was 71.23 years (SD = 4.44), and the sample was 59% female. Due to the volunteer nature of the sample and the demographics of the registry, the sample was largely from a high socioeconomic status background. The sample was mostly white and non-Hispanic (92%), most reported an income of $60,000 or more (80%), and most had a bachelor's degree or higher (78%).	mobile and wearable technologies			Participants rated all technology solutions as well above neutral (3) in terms of recommending the use of the technology to the individual described in the scenario. Contrasts found that participants demonstrated a stronger preference to recommend the app to support health (M = 4.17, SD = 0.82) over the smartwatch to predict cognitive decline (M = 3.91, SD = 0.90; p < 0.01, Cohen's d = 0.34; Bonferroni correction).	However, more positive general attitudes toward technology (as measured by the Tech Readiness Index) and higher technology proficiency (as measured by the Mobile Device Proficiency Questionnaire) were associated with more positive technology attitudes toward the technologies to support health and cognition. Also, contrary to expectations, poorer health was associated with less positive attitudes toward the described technologies.Not surprisingly, a strong positive association was observed between participants' interest in the described technologies and their willingness to adopt them. Perceived usefulness also correlated strongly with participants' willingness to adopt. Perceptions of usefulness and interest also correlated with participants' likelihood of recommending the technologies to others. Finally, negative associations (though weaker) were observed between privacy concerns and interest and willingness to adopt, as well as participants' ratings of usefulness of the described technologies and whether participants would recommend these technologies to others.							Privacy concerns<br>Participants generally neither agreed nor disagreed that individuals described should have privacy concerns when using these three pieces of technology (Figure 1). Responses were close to "neutral" for all technologies, though there were significant differences as a function of technology type (F(2,182) = 3.599, p < 0.05; ηp2 = 0.006). This was driven by more privacy concerns for assessing cognition compared (M = 3.02, SD = 1.09) to supporting adherence (M = 2.80, SD = 1.08; p < 0.01, Cohen's d = 0.38; Bonferroni correction).	N
The use of technology in cancer care: applying Foucault's ideas to explore the changing dynamics of power in health care	2009	JOURNAL OF ADVANCED NURSING	Forbat, L and Maguire, R and McCann, L and Illingworth, N and Kearney, N	10.1111/j.1365-2648.2008.04870.x	The use of technology in cancer care: applying Foucault's ideas to explore the changing dynamics of power in health care. This study is a report to identify the utility of a hand-held side-effect monitoring system for people receiving chemotherapy in the home care setting.	Quantitative	UK	people receiving chemotherapy.	Participants who formed the sub-set of 12 people contributing to interviews were: two men, 10 women. Three patients had colorectal cancer, and nine had breast cancer. The age range was 38–66 years, with a mean of 50·58.	The ASyMS© system is a mobile telephone-based remote symptom monitoring system which is used to log and manage the side-effects of chemotherapy in the home care setting			The notion of 'monitoring' in the above account directly invokes ideas of surveillance. However, it is presented not as daunting/unwarranted, but as reassuring.							Interviewees felt that the system opened further lines of communication and allowed for a greater volume of communication. Thus, far from being at the mercy of the medical gaze, patients felt that the symptom monitoring system gave them easier access to cancer specialists. The notion of easy access subverts the typical understanding of medical surveillance, meaning that it is the clinician who is responsive to the patient's reports and needs at any given time. This disturbance of the usual dynamic suggests that the use of technology may be an important tool in challenging traditional hierarchical models of health care which may marginalize patients, thereby contributing to the promotion of a patient-driven model of care.<br>The speaker marked out the need for the system for other patients who might hesitate in contacting the hospital.<br>Experiences of surveillance<br>Interviewees were asked for feedback on how they felt when staff got in touch as a consequence of worrying symptoms that had been reported using the system. Overwhelmingly, patients felt this was extremely useful and not troubling	the patient's agency is reduced and they take on a more passive role. It is possible that such medical surveillance may be internalized by the patient, which in turn may lead to them failing to take a more assertive approach.Another interviewee's ideas suggested that the system, and professionals' responses, sometimes placed the onus on the patient to take the lead	UNDECLARED
Accuracy, User Acceptability, and Safety Evaluation for the FreeStyle Libre Flash Glucose Monitoring System When Used by Pregnant Women with Diabetes	2018	DIABETES TECHNOLOGY & THERAPEUTICS	Scott, EM and Bilous, RW and Kautzky-Willer, A	10.1089/dia.2017.0386	Accuracy of the FreeStyle Libre Flash Glucose Monitoring System has not been evaluated in pregnant women with diabetes. The aim of this study was to determine accuracy (compared to self-monitoring of blood glucose [SMBG]), clinical safety, and acceptability of the FreeStyle Libre System when used at home by this population.	Quantitative	United Kingdom, Austria	Pregnant Women with Diabetes	Characteristic Diabetes type Mean ± SD Median Range N<br>Age (years) Gestational 31.0 ± 4.9 31.0 21–41 39<br>Type 1 28.4 ± 5.2 29.0 19–37 24<br>Type 2 33.4 ± 3.7 33.0 27–39 11<br>Overall 30.5 ± 5.1 31.0 19–41 74<br>Body mass index (kg/m2) Gestational 34.2 ± 6.8 33.2 20.8–48.2 39<br>Type 1 27.8 ± 4.5 27.7 19.5–35.1 24<br>Type 2 35.9 ± 8.4 33.8 23.7–50.4 11<br>Overall 32.4 ± 7.1 31.7 19.5–50.4 74	Flash Glucose Monitoring System					Statements about sensor application (88.9%–95.8%), sensor wear and use (55.4%–100.0%), and comparisons to SMBG (93.7%–100.0%) were rated favorably (strongly agree/painless or agree/almost painless) by most participants (Fig. 2).							All authors' institutions received financial support from Abbott Diabetes Care to conduct the clinical trial. E.M.S. received honoraria/fees for consulting from Abbott Diabetes Care outside of the submitted work. A.K.W. and R.W.B. have no competing interests. The study sponsor (Abbott Diabetes Care) designed the study protocol in collaboration with the principal investigator and provided all study materials. The sponsor was involved in collecting data and reporting results. The sponsor also gave approval to submit for publication. The corresponding author had full access to all the data in the study and, together with all authors, had final responsibility for the decision to submit for publication.
Automated Personalized Feedback for Physical Activity and Dietary Behavior Change With Mobile Phones: A Randomized Controlled Trial on Adults	2015	JMIR MHEALTH AND UHEALTH	Rabbi, M and Pfammatter, A and Zhang, M and Spring, B and Choudhury, T	10.2196/mhealth.4160	MyBehavior, a mobile phone app, was designed to process tracked physical activity and eating behavior data in order to provide personalized, actionable, low-effort suggestions that are contextualized to the user's environment and previous behavior. This study investigated the technical feasibility of implementing an automated feedback system, the impact of the suggestions on user physical activity and eating behavior, and user perceptions of the automatically generated suggestions.	Mixed	USA	General public	students (76%), 4 professionals (24%), 8 females (47%), and 9 males (53%), and all were between the ages of 18 and 49 (mean 28.3, SD 6.96, lower quartile [q25]=22, median [q50]=26.3, upper quartile [q75]=36). All participants reported low-to-moderate levels of physical activity. The majority of participants were experienced mobile phone users—9 participants (53%) had previous experience using a food diary, and 6 participants (35%) had previously kept an exercise log. After the randomization, participants in the groups were similar in terms of level of active lifestyle and experience with using mobile-based self-management tools.	Automated Personalized Feedback for Physical Activity and Dietary Behavior Change With Mobile Phones			In the suggestion-rating survey, the experimental group (mean 3.4, SD 1.2, q25=2.75, q50=3, q75=4), with MyBehavior suggestions, intended to follow personalized suggestions more than the control group (mean 2.5, SD 1.6, q25=1, q50=2, q75=4) intended to follow the generic suggestions. A nonparametric Mann-Whitney U test [32] found this difference to be statistically significant (P<.001, 95% CI 0-1.001, effect size = 0.99).							First, participants in the experimental group described the activity suggestions to be actionable and relevant to their lives. Control group participants appreciated that the generic suggestions reminded them of good habits.	However, they often faced problems incorporating the suggestions into their daily lives. The following quotes were taken from the daily diaries of participants.In qualitative feedback, similar to physical activity suggestions, experimental group users found the suggestions to be more actionable and reported to make more changes compared to control group users who found the suggestions to be hard to work on.	N
The Design and Evaluation of Personalised Ambient Mental Health Monitors	2010	2010 7TH IEEE CONSUMER COMMUNICATIONS AND NETWORKING CONFERENCE-CCNC 2010	Blum, JM and Magill, EH and IEEE		We have developed a sensor network infrastructure for mobile phones and homecare using a rule-oriented programming architecture to monitor the activity signatures of people with Bipolar Disorder (BD). We are evaluating the effectiveness of the technology in an ongoing technical trial with control participants as a precursor to studying the effectiveness of the system for use with people with BD.	Mixed	UK	people with Bipolar Disorder (BD).	high degree of exposure to the trial equipment and technical trial protocols prior to beginning the technical trial. They all lived with at least one other housemate and sought permission of their housemates prior to installing the equipment.	Personalised Ambient Mental Health Monitors	The participants had no or little prior experience of being monitored outside of the PAM project.		For three of the participants, their feelings about the degree of monitoring in society ranged from generally negative, but acknowledging value for medical purposes, to wholy positive (no response was recorded for the fourth participant).		The participant reported positive feelings towards the mobile phone's form factor and screen quality, but he found the mobile phone questionnaire and transfer applications burdensome, and reported a fairly low compliance rate.owever, he reported that even though answering questionnaires was a quick process it was still overly intrusive and his compliance was low. He also reported that he missed many on-screen notifications and preferred audio-based notifications.						However, during his exit interview, the participant expressed concern regarding the installation procedures. He described a sense of loss of control owing to having three people taking part in the installation and a feeling that the installation took too long.	N
Patient Attitudes Toward Mobile Phone-Based Health Monitoring: Questionnaire Study Among Kidney Transplant Recipients	2013	JOURNAL OF MEDICAL INTERNET RESEARCH	McGillicuddy, JW and Weiland, AK and Frenzel, RM and Mueller, M and Brunner-Jackson, BM and Taber, DJ and Baliga, PK and Treiber, FA	10.2196/jmir.2284	To assess the attitudes of renal transplant recipients toward mobile phone based remote monitoring and management of their medical regimen; and to identify demographic or clinical characteristics that impact on this attitude.	Quantitative	USA	kidney transplant recipients	Variable Mean (± SD) or Proportion<br>Age 53.1 ± 13.4 (Median 52)<br>Gender (Male) 65% (64/98)<br>Race (Black) 66% (65/99)<br>Marital status (Married) 64% (63/98)<br>Education level (≤High School) 38% (38/99)<br>Employment (Part or Full Time) 22% (21/98)<br>Annual income (<$30,000) 57% (44/77)<br>Months since kidney transplant 29.2 ± 54.5<br>More than one transplant 12% (12/99)<br>Perceived Stress Scale score 10.9 ± 6.5<br>Morisky Scale score 6.1 ± 1.1	mobile phone-based health monitoring	Only 7% (7/98) of participants had any prior knowledge of mHealth remote monitoring technology before being surveyed (Table 2).		On the matter of health information, 80% (79/99) indicated that they were comfortable with a health care provider monitoring their health information using remote monitoring technology and 76% (75/99) felt confident that their privacy could be adequately protected. Almost all participants (95%, 94/99) felt that it was important to follow their doctor's directions and 87% (86/99) thought that remote monitoring technology would help them effectively communicate with their health care providers about their medical conditions.	blacks had a slightly more positive attitude toward mHealth than whites as gauged by their willingness to use the technology if it came at no cost to them (P=.02). There were no significant differences between blacks and whites in the level of education (P=.62), annual income (P=.16) or employment status (P=.46).<br><br>Characteristics of Respondents with Positive Attitude Toward mHealth<br>Respondents who answered either 'agree' or 'strongly agree' to the query as to whether they would use the mHealth system, as demonstrated, if it were free, were more likely to be employed (P=.04), the recipient of their first transplant (P=.02), already using a medication tray at home (P=.04) and the owner of a working mobile phone (P=.04). These respondents were also more likely to own a smartphone (P=.01) and to have used a mobile phone to text (P=.02), email (P=.01), browse the internet (P=.002) or download an application or ringtone (P=.03). As can be seen in Table 4, they also reported higher levels of perceived stress (P=.01).			When asked about how they would prefer to receive instructions from their health care providers, most respondents preferred that communication be done via phone call, with voicemail being the most common second choice. Text messaging was the third most common choice, with only a small fraction of patients indicating that they were interested in receiving instruction primarily via live video conferencing.			However, most of the participants felt that mobile technology would be helpful in reminding them to follow their doctor's directions (81%, 80/99). The majority also felt that the technology would allow their doctor to make more rapid adjustments to their medication regimen (84%, 83/99). Furthermore, most of the participants, 79% (78/99), indicated that if they were provided the mHealth system at no cost and instructed on its use that they would use it as directed by their health care provider. The addition of free technical support did not significantly increase their willingness to use the devices.		N
Usability testing of an electronic pain monitoring system for palliative cancer patients: A think-aloud study	2019	HEALTH INFORMATICS JOURNAL	Taylor, S and Allsop, MJ and Bennett, MI and Bewick, BM	10.1177/1460458217741754	Pain experienced by advanced cancer patients is often poorly controlled due to inadequate assessment. We aimed to test an electronic pain monitoring system (PainCheck) with advanced cancer patients and health professionals.	Qualitative	UK	palliative cancer patients	Patients<br>Sex<br>Female (n) 5<br>Male (n) 8<br>Age (median, range) 65 (45–87)<br>Diagnosis<br>Prostate (n) 3<br>Breast (n) 2<br>Lymphoma (n) 2<br>Myeloma (n) 2<br>Lung (n) 1<br>Oesophagus 1<br>Oropharyngeal 1<br>Ovarian 1<br>Health professionals<br>Sex<br>Female (n) 14<br>Male (n) 2<br>Age (median, range) 43 (30–56)<br>Number of years' experience in current role (median, range) 8.5 (1–25)<br>Professional group<br>Palliative care doctor (n) 5<br>Clinical nurse specialist (n) 4	an electronic pain monitoring system for palliative cancer patients							Participants made a number of suggestions of ways PainCheck could be improved. These suggestions focused on how PainCheck could be made easier to navigate or how functions could be clarified:To be implemented successfully, patients and health professionals felt a clear process needed to be established so everyone using PainCheck was clear who was responsible for taking action.	A proportion of participants were concerned about problems being missed or thought response speed may be affected so they would prefer to communicate by phone. Patients who would opt for telephone communication at the moment felt their views might change in the future if there was a transition to using electronic reporting methods and the healthcare team encouraged it:		Despite their concerns, patients and health professionals did think there were patients who would enjoy using the system and would benefit from completing the reports. Patients felt PainCheck would help to highlight particular issues they have been experiencing or help them to focus their thinking and put their pain into perspective:		N
Capturing Rest-Activity Profiles in Schizophrenia Using Wearable and Mobile Technologies: Development, Implementation, Feasibility, and Acceptability of a Remote Monitoring Platform	2018	JMIR MHEALTH AND UHEALTH	Meyer, N and Kerz, M and Folarin, A and Joyce, DW and Jackson, R and Karr, C and Dobson, R and MacCabe, J	10.2196/mhealth.8292	This study aimed to describe the development, implementation, feasibility, acceptability, and user experiences of the Sleepsight platform, which harnesses consumer wearable devices and smartphones for the passive and unobtrusive capture of sleep and rest-activity profiles in people with schizophrenia living in their homes.	Quantitative	UK	Schizophrenia patient	Inclusion criteria were ICD-10 criteria for schizophrenia, aged between 18 and 65 years, and capacity to consent to research; the criteria for exclusion were gross cognitive, sensory, or motor impairments that precluded the use of study devices. Overall, 8 participants had negative symptoms subscale scores of at least 20 (moderate intensity or greater on 5 of the 7 items) and 2 greater than 25 (severe intensity or greater on at least 5 items). All were receiving antipsychotic treatment, and 7 of the participants were prescribed clozapine, as indicated in the United Kingdom for psychosis that is resistant to other antipsychotics. A total of 8 participants lived alone and 7 lived with a spouse, children, or wider family. None of the participants were in regular paid employment. All participants owned a mobile device of their own, and just over half of the participants had a touchscreen device.	Wearable and Mobile Technologies			Overall, responses to the end-of-study questionnaire relating to the Sleepsight system and each of its elements were positive (Multimedia Appendix 1).		A total of 14 participants completed the end-of-study interview; the majority found the system to be easy to grasp and use, though 1 participant found it complicated. Technology-Related Factors Half of the users commented that the wearable was at times uncomfortable: the strap would feel tight, which was particularly bothersome in bed. However, most of the users reported initial discomfort, which improved as the study progressed. One participant reported intermittent concern over radiation emerging from the optical heart rate monitor. There were no reports that the sleep metrics were inaccurate; however, 3 participants felt that the Fitbit overestimated step count. A third of participants found value in using the Fitbit for setting and attaining physical activity goals. Charging of the Fitbit or smartphone did not raise any concerns.A sense of being "acknowledged" as a specific consequence of using the symptom diary was reported by 3 individuals. Moreover, 2 mentioned how it encouraged family to be involved and became a focus for education. In addition, 6 participants stated that the symptom diary became repetitive and tedious, particularly when the users felt their symptom burden was low, and that if there were no incentive, they would be unlikely to answer the questionnaire on a daily basis. Two users found some questions difficult to understand. A wish to have greater flexibility in the timing of diary completion was raised by 2 users.		Many felt that measuring sleep patterns would be an important element of self-management and could imagine how psychological or pharmacological interventions delivered in the early stages of relapse could be therapeutic. 4 participants felt that their engagement in the study was influenced by the knowledge that their data were not being analyzed and responded to in real time, and if this were to have been the case, they would have greater levels of engagement.	Illness-Related Factors A quarter of participants expressed concern that adherence would diminish during relapse. First, users' cognitive function and therefore the ability to interact with the technology might decrease as symptoms escalate. Second, users may develop suspicion toward the devices, leading to discontinuation. Generally, users felt that the wearable device would be better tolerated in this scenario than the smartphone diaries.		All participants found the personalized encouragement text messages to be helpful in improving motivation.<br>Two-thirds of participants found that completing the daily symptom diary helped them reflect on mental state and gain greater insight into the links between their symptoms and behaviors, especially sleep.	3 participants reported feeling occasionally suspicious toward the technology and worried that their personal information was at times being monitored. However, this did not lead to discontinuation. Overall, 5 participants commented on how they would have concerns over false alarms	RJ is an employee of AstraZeneca.
A critical occupational perspective on user engagement of older adults in an assisted living facility in technology research over three years	2020	JOURNAL OF OCCUPATIONAL SCIENCE	Holthe, T and Halvorsrud, L and Lund, A	10.1080/14427591.2020.1758200	This study sought to investigate how eight older adults in an assisted living facility in Norway, aged 81-92 years, evaluated user inclusion in a 3-year technology-oriented research project.	Qualitative	Norway	Older adults	adults aged between 81 and 92 years. All were mobile, although half used mobility aids. Participants had resided at the assisted living facility from 6 months to 16 years	assisted living facility in technology						motivation for project engagement with the sub-themes openness to learning new things, preparing for own old-age, participation for others' benefit, and appreciating being treated as a citizen;		difficulty explaining the project to others, treatment as guinea pigs versus co-researchers, and commitment and feeling of responsibility		using technology for hope and goals,	technology as an occasional burden, and failure of technology.	N
Investigating When, Which, and Why Users Stop Using a Digital Health Intervention to Promote an Active Lifestyle: Secondary Analysis With A Focus on Health Action Process Approach-Based Psychological Determinants	2022	JMIR MHEALTH AND UHEALTH	Schroe, H and Crombez, G and De Bourdeaudhuij, I and Van Dyck, D	10.2196/30583	The aim of this study was to examine when, which, and why users stopped using a digital health intervention. In particular, we aimed to investigate whether psychological determinants of behavior change were predictors for attrition.	Quantitative	Belgium	General public	Of these participants, the mean age was 36.7 (SD 16.3) years, 69.1% (327/473) of participants were female, 66.4% (314/473) had a high level of education (college or university degree), 30.2% (143/473) were overweight, 42.3% (200/473) had a partner, and 49.0% (232/473) met the guidelines for either PA or SB.	a Digital Health Intervention to Promote an Active Lifestyle								[illegible] out the questionnaires took a lot of my time" (28/51, 55%), "I don't have time" (26/51, 50%), "The intervention doesn't provide useful content" (21/51, 41%), "I experienced technical problems with the website or app" (20/51, 39%), "I already meet the health guidelines for PA/SB" (16/51, 31%), and "I experienced medical/emotional problems" (8/51, 16%).<br><br>The following reasons were reported not to be important to stop using the intervention: "I am not interested in the topic" (1/51, 2%) and "I don't want to change my behavior" (1/51, 2%).				N

Clinical evaluation of IDAS II, a new electronic device enabling drug adherence monitoring	2007	EUROPEAN JOURNAL OF CLINICAL PHARMACOLOGY	Santschi, V and Wuerzner, G and Schneider, MP and Bugnon, O and Burnier, M	10.1007/s00228-007-0364-7	The goal of this study was to evaluate clinically the acceptability of the IDAS II (Intelligent Drug Administration System), a new electronic device that enables drug adherence monitoring.	Quantitative	Swietherland	hypertensive patient	Sex (men/women)<br>12/13<br>Mean age, years (range)<br>58.0 (35–75)	a new electronic device enabling drug adherence monitoring			The assessment of patient satisfaction revealed that the majority of the patients considered both electronic devices to be a reliable reminder (84% with the MEMS vs. 75% with the IDAS II). Eight patients (33%) did not take any interest in the IDAS II device, and eight (33%) considered it to be useless. Approximately half of the patients expressed a willingness to use both devices in the future.		All patients were able to handle the MEMS device, and [illegible]% of them were able to use the IDAS II device appropriately. More than one third of the patients (9/24; 38%) indicated that pressing down on the tablets to get them out of the special blister cards of the IDAS was difficult; however, replacing the blister card with a new one was easy for nearly all patients (23/24; 96%).<br><br>One patient experienced difficulties in opening the IDAS II, in releasing the latches and in pressing down on the tablets to get them out of the blister card. One patient did not use the IDAS II correctly, and the device did not record the doses.<br><br>No patient expressed problems reading the information displayed on both devices. Most of the patients characterised the display information of the IDAS II (21/24; 88%) and the MEMS device (21/25; 84%) as "easy to understand". The answers differed in terms of the usefulness of the visual and audible reminders. The MEMS LCD display information indicating the number of doses taken during the present day and the number of hours elapsed since the last opening were considered to be useful by 64% (16/25) and 28% (7/25) of the patients, respectively. The IDAS II digital display indicating the time since last dose, the visual blinking reminder and the audible reminder were considered to be useful by 46% (11/24), 38% (9/24) and 42% (10/24) of patients, respectively. Four patients asked the pharmacist to deactivate the IDAS II audible reminder.<br><br>Patients reported two types of technical failures with the IDAS device. First, the IDAS II had a higher incidence of LCD display failures than the MEMS device (42	Patients preferred the blister pack (IDAS II) to the bulk pill-container (MEMS) because of better expected hygiene. More patients preferred the IDAS II as a supporting device over the MEMS.	However, nine patients said that neither of the devices was of any help for the simple reason that they already had a strong daily routine for drug intake (i.e. teeth-brushing, mealtimes, walking the dog and shaving).		In terms of drug intake, 14 patients with the MEMS (56%) and 12 patients (50%) with the IDAS II reported that the devices helped them maintain a more regular drug intake.	Only two patients felt that they were being "observed" too much with both devices.	N
The Views and Needs of People With Parkinson Disease Regarding Wearable Devices for Disease Monitoring: Mixed Methods Exploration	2022	JMIR FORMATIVE RESEARCH	Kenny, L and Moore, K and O' Riordan, C and Fox, S and Barton, J and Tedesco, S and Sica, M and Crowe, C and Alamaki, A and Condell, J and Nordstrom, A and Timmons, S	10.2196/27418	This study aims to understand the views and needs of people with Parkinson disease regarding wearable devices for disease monitoring and management.	Mixed	Ireland	individuals with Parkinson disease	Of the respondents, 56% (18/32) were male and 44% (14/32) were female, ranging in age from 50 to 83 years, with a median age of 68 years.	Wearable Devices for Disease Monitoring						Wearer feedback was the least highly rated category in the survey. Monitoring and feedback of blood pressure was also a desirable feature Participants suggested that data collected could be used to activate a response or intervention.<br>The ability of a wearable device to alert the person to nonmotor symptoms, such as mood, stress, and sleep, was stated as important People were especially interested in being able to know how their data compared with the data of other people with Parkinson disease and the potential solidarity that it could bring through knowing they were not alone in a certain symptom	[illegible] respondents highly rated a small, easy-to-use device that would not interfere with their routine; they responded strongly agree, with 64.4% (116/180) and 67% (60/90) overall score, to the categories for wearability and user interface, respectively (Table 2).<br><br>The wearability and usability of wearable devices for Parkinson disease similarly emerged in the focus groups, and it was felt that wearable device design should consider the user needs to ensure compliance and adoption. Although the size and weight of the device were important, the aesthetics of the device appeared less significant. Participants felt that wearable device design should consider the complexity of Parkinson disease symptoms, especially motor dexterity. Participants also discussed the importance of ease of use, device weight, and a design which reflected the needs of people with Parkinson disease.<br><br>Participants felt that although most participants felt comfortable with technology, some may feel intimidated. Designs should make allowances for a person's possible lack of knowledge or exposure to a specific technology. It was discussed how some would need general guidance to feel more comfortable, which would also enable greater user adoption of the device:<br><br>Another possible barrier was the cost of technology; wearable devices should not be expensive, as this may exclude some people with		Clinical usefulness was the highest rated thematic category in the survey (Table 2). People with Parkinson disease felt strongly that a device should increase clinical accuracy, reduce travel, and integrate as part of their care team. In the focus groups, participants emphasized that technology should objectively monitor their symptoms, and it was deemed beneficial if this information could be relayed to their medical care team: A wearable device that captures Parkinson disease data could provide richer health information to clinicians, which may aid in the improved assessment of Parkinson disease. The capturing of individual health parameters would eliminate the need for diary-based recording of symptoms, which was believed to be problematic at times. Although it was important for health care teams to have access to the data, people also wanted a sense of ownership over their own health care. One focus group included a discussion on whether medical professionals would like technology that could alter the dosage of medicines they prescribe and that this may require a change in attitude from some health professionals		N
Remote Measurement in Rheumatoid Arthritis: Qualitative Analysis of Patient Perspectives	2021	JMIR FORMATIVE RESEARCH	White, KM and Ivan, A and Williams, R and Galloway, JB and Norton, S and Matcham, F	10.2196/22473	Rheumatoid arthritis (RA) is characterized by recurrent fluctuations in symptoms such as joint pain, swelling, and stiffness. Remote measurement technologies (RMTs) offer the opportunity to track symptoms continuously and in real time; therefore, they may provide a more accurate picture of RA disease activity as a complement to prescheduled general practitioner appointments.The objective of this study is to determine the important outcomes for disease management in patients with RA and how these can be best captured via remote measurement.	Qualitative	UK	patients with Rheumatoid Arthritis	23 years to 77 years (mean 55.78, SD 17.54) who had lived with a diagnosis of RA for 1-47 years (mean 20.22, SD 14.33; Table 1). Ethnicities included White (n=6), White and Black African (n=1), Pakistani (n=1), and Caribbean (n=1).	Remote Measurement in Rheumatoid Arthritis			There is a general consensus that the ability to track fluctuations and transmit such data to clinicians would aid in individual symptom management and the effectiveness of clinical care.			Suggestions for visually capturing symptom fluctuations in an app were proposed					FM received honoraria or speaking fees from Pfizer (less than $10,000) for contributing to work unrelated to this piece of research.
Caregivers' Perceived Usefulness of an IoT-Based Smart Bed	2022	DISTRIBUTED, AMBIENT AND PERVASIVE INTERACTIONS, SMART ENVIRONMENTS, ECOSYSTEMS, AND CITIES, PT I	Bacchin, D and Pernice, GFA and Sardena, M and Malvestio, M and Gamberini, L	10.1007/978-3-031-05463-1_18	This study investigates caregivers' perceived usefulness, a key factor of the Technology Acceptance, of an innovative IoT patient monitoring system, called smart-bed, able to provide valuable information about patients without being in contact with them	Qualitative	Italy	Caregivers	In total, the study participants were 29 (F = 19, Mage = 39, SDage = 9).	an IoT-Based Smart Bed			The results showed that in 100% of the focus groups, in all 6, the participants declared that they found the system useful for their work.			During the FGs, the caregivers highlighted the smart-bed system's more important and interesting functions.The first was the creation of alerts, which emerged in 2 FG out of 6, those with hospital nurses. The patient's vital signs control was a high-discussed point. Another theme is the control of risks for the patient.A further parameter desired by users is wet sensing, which emerged in 2 FG out of 6. It was indicated as useful to prevent injuries. Finally, other wishes that emerged individually in only one of the FGs are the integration of the system with the IT medical record.			This result underlined the usefulness of the system during the night shift, but its use was perceived positively also during the day. As was highlighted by the hospital nurses (FG1), the system would work greatly also during the day, especially for the management of hectic or particularly busy working moments where the attention of the operators is less present.<br><br>In 4 of the 6 FG, participants also discussed about how using the data recorded by the smart bed. In summary, some participants expressed their interest in analyzing this data to improve their working environment. Safety was among the most cited aspects (3 FG out of 6) for the patient and the operator's work. The SHO find useful receiving alarms from the system.<br><br>Furthermore, during the focus groups, the participants discussed about what is the working environment in which it is more beneficial to insert the bed system (5 FG out of 6). In the case of nurses in the Hospital of Padua (FG1), belonging to a work environment with frequent use of telemetry, they found only some	The methodology used also revealed some criticalities and limitations of the system.<br><br>First of all, in 4 out of 6 FG participants were worried by the presence of alarms other than hospital ones, a possible source of alarm fatigue.<br><br>There is also a risk, which emerged in 2 FG out of 6, on the acceptance of the system due to the trustworthiness of the data.<br><br>It is also interesting to highlight a problem detected in one of the FGs, in which the discretion of mobile devices for receiving alarms was discussed because it could impact on the professionalism of the operators.<br><br>The need to not overload the already overburdened bureaucratic commitments has also emerged as a possible limitation of the system.<br><br>Another possible obstacle concerns the	UNDECLARED
Wearable Activity Monitoring in Day-to-Day Stroke Care: A Promising Tool but Not Widely Used	2021	SENSORS	Braakhuis, HEM and Bussmann, JBJ and Ribbers, GM and Berger, MAM	10.3390/s21124066	This cross-sectional study explores the use, perspectives, and barriers to wearable activity monitoring in day-to-day stroke care routines amongst physical therapists.	Quantitative	Netherlands	patient with Stroke	Demographic characteristics of respondents.<br><br>Total (n = 103) Users (n = 28) (27%) Non-Users (n = 75) (73%) p-Value<br>Age, mean (SD) 42.2 (12.06) 41.70 (13.24) 45.30 (12.11) 0.212<br>Gender (m/f) 26/76 8/20 18/56 0.420	Wearable Activity Monitoring in Day-to-Day Stroke Care	More than half of the non-users (59%) were familiar with activity monitoring before filling in this questionnaire. Similar percentages of users (54%) and non-users (53%) used a smartphone app or consumer-grade activity tracker for monitoring their own lifestyle and sports activities. Two participants (1.9%) considered themselves as people who were initially reluctant to use new healthcare technology and innovations. Most of the therapists in the total study sample described themselves as a person who had no problem going along with pioneers in healthcare technology and innovation but who did not initiate it themselves (60%). Only one (0.9%) of the therapists described himself as someone who invented and designed new healthcare technology and innovations and		All participants (users and non-users) were asked for their opinion about the usefulness of activity monitoring for stroke patients. Six out of seven suggested purposes were considered useful by more than half of the study sample (Figure 3).One significant difference was found between users and non-users: the users perceived creating awareness as more useful than non-users (p = 0.031). The participants were asked if they could come up with useful purposes other than noted in the question. Sixteen participants (16%) filled in the open-ended question on useful purposes other than mentioned in the question (Figure 3). Providing insight into a patients' demands vs. their capabilities (n = 6) was the most common purpose. Two mentioned heart rate and one mentioned arm/hand use.		Figure 2B shows the activity monitor outcomes of interest during treatment. Most of them were interested in the number of steps. Additional outcomes of interest reported by users were heart rate and demands vs. capability, or in other words, the relation between what a patient did compared to what the patient was capable of.	that they were instructed or motivated by external factors such as other colleagues who were already working with activity monitors or research/projects initiated by their organizations.	The most present barriers reported by the whole sample were lack of skills and knowledge of patients (65%), not being sure what monitor to purchase (54%), finding it too expensive (47%), and taking too much time (27%).		Almost all therapists used the monitor to create awareness for the patient with regard to their physical behavior (96%). Giving feedback about their physical behavior (82%) was also often recognized as a useful activity monitoring purpose. Several positive and enthusiastic thoughts on activity monitoring were provided. Participants report that activity monitoring offers valuable insight into a patients' behavior.	they had doubts about the added value to the standard care relative to the effort. A few stated that applying technology was not always a holy grail and could not define therapy. Multiple participants mentioned that the usefulness was highly dependent on the age and stroke severity of the population.	N
Attitudes of Professional Caregivers and Family Members Regarding the Use of Monitoring Devices to Improve Assessments of Pain and Discomfort During Continuous Sedation Until Death	2020	JOURNAL OF PAIN AND SYMPTOM MANAGEMENT	Six, S and Van Overmeire, R and Bilsen, J and Laureys, S and Poelaert, J and Theuns, P and Deschepper, R	10.1016/j.jpainsymman.2020.02.015	Assessing consciousness and pain during continuous sedation until death (CSD) by behavior-based observational scales alone has recently been put into question. The aim of this study was to find out what influences professional caregivers' and family members' (FMs) attitudes regarding the use of monitors during CSD.	Qualitative	Belgium	Professional Caregivers and Family Members, patients in Continuous Sedation Until Death	Characteristics Physicians Nurses FMs<br>Number of interviewed participants 8 12 15<br>Sex<br>Male 3 4 8<br>Female 5 8 7<br>Age (yrs)<br>Younger than 30 3 3 —<br>30–39 1 1 —<br>40–49 2 3 2<br>Older than 50 2 5 13<br>Setting<br>Locoregional hospital (palliative care unit) 2 5 12<br>Academic hospital 4 4 —<br>Nursing home 2 3 3<br>Total number of interviews 11 14 7<br>FMs = family members.	Monitoring Devices to Improve Assessments of Pain and Discomfort During Continuous Sedation Until Death			Almost all CGs and FMs believed that using monitoring devices to measure consciousness and pain during CSD more objectively is a substantial improvement. Nearly every participant indicated they had some degree of uncertainty about the accuracy of their observational assessment.<br>In general, all interviewed CGs and FMs were open to the idea of using monitoring devices		with a small number of CGs expressing some initial concerns that the sight of a sensor patch on the forehead of the patient could perhaps be unacceptable for some FMs.		Setting<br>Palliative care units and nursing homes typically adhere to the principle of creating a feeling-at-home environment, where medical technology has no place. This tenet however may be overstated and should leave room for noninvasive monitoring technology<br>Emotional valence of using monitors<br>Some CGs find it difficult to accept that monitoring devices in a CSD context are necessary (although behavior-based observation alone has already been shown unreliable). Continuing professional education can address this<br>Acceptability to FMs<br>Although almost all CGs see the added value of monitoring devices, most of them fear that the sight of sensors on the forehead would be unacceptable to FMs. However, contrary to this, no FM reported having any issues with that<br>FMs<br>Uncertainty avoidance<br>FMs wanted to be sure that their relative was comfortable and free of pain and perceived the extra measures by monitors as an added value to achieve this<br>Objective monitoring had a reassuring effect on some FMs, and they reported feeling somewhat better knowing that their relative was being followed so closely for signs of discomfort<br>Doing the best you can	CGs<br>Setting<br>High-tech care settings such as ICUs allow for easy use of monitoring devices<br>Uncertainty avoidance<br>Objective assessments give trust and confidence to CGs<br>Awareness that care can be improved<br>Realizing that unresponsiveness ≠ unconsciousness; observational means of assessment may not always detect suffering in a context of CSD<br>Using state-of-the-art monitoring techniques to provide the currently best possible care. Monitoring can help in guiding titration of medication, reducing the risk of oversedation or undersedation. This could also prove helpful to prevent CG burden	Peace of Mind<br>Both CGs and FMs mentioned that dignity in dying was very important to both of them. Dignity plays a crucial role to be able to avoid or minimize CG burden and/or emotional burden.		N
Analysis of Health Consumers' Behavior Using Self-Tracker for Activity, Sleep, and Diet	2014	TELEMEDICINE AND E-HEALTH	Kim, J	10.1089/tmj.2013.0282	With the ever-increasing availability of health information technology (HIT) enabling health consumers to measure, store, and manage their health data (e.g., self-tracking devices), more people are logging and managing their own health data for the purpose of promoting general well-being. The aim of this study is to verify the HIT acceptance model (HITAM) in the context of the health consumer's attitude, behavioral intention, and behavior of utilizing self-trackers. Furthermore, the study aims to gain better understanding of self-tracking behavior in the context of logging daily activity level,	Quantitative	Korea	General public	The average age of the 52 participants who answered the preselection questionnaire was 24.56 (±4.9) years. Of these individuals, 44 agreed to wear the PMD and logged and tracked activity, sleep, and diet data.	Self-Tracker for Activity, Sleep, and Diet			The model accounts for 66.8% of the variance in "Perceived Usefulness," 83.1% of the variance in "Attitude," and 48.4% of the variance in "Behavioral Intention"		43.9% of the variance in "Perceived Ease of Use,"						N
Personal metrics: Users' experiences and perceptions of self-tracking practices and data	2020	SOCIAL SCIENCE INFORMATION SUR LES SCIENCES SOCIALES	Ajana, B	10.1177/0539018420959522	Overall, this article highlights the benefits and risks of self-tracking practices as experienced and articulated by the participants, while providing a critical reflection on the rise of personal metrics and the culture of measurement and quantification.	Mixed	USA, UK	General public	68.5% of participants identified as female, 30.9% identified as male and 0.8% identified as 'other'. In terms of participants' age, 33.9% fell within the 26–35 age category, 26.7% within 36–45 category, 10.2% within 18–25 category, and 29.5% of participants were over 45.	self-tracking practices and data				Participants' attitudes towards privacy and data protection are seemingly dominated by a lack of concern regarding the use and sharing of self-tracking data with third parties. Some of the overarching sentiments vis-à-vis these issues can be roughly categorized according to feelings of 'trust' towards companies and how they handle data, a sense of 'resignation' in the face of what is perceived as an all-encompassing and ubiquitous data use, feelings of 'self-insignificance' which translates into the belief that one's data is of no value to others, and the familiar expression of 'the innocent have nothing to hide'.					As the findings of the study demonstrate, self-tracking carries many benefits to the user, particularly with regard to increasing motivation and encouraging a healthier and more active lifestyle.	Nevertheless, the data-driven and quantifying aspects of self-tracking can sometimes lead to excessive forms of self-monitoring which can increase the pressure on the user to perform according to certain standards of health, resulting in feelings of inadequacy, at times, and inability to enjoy the exercise activities themselves.	N
Seeking connectivity to everyday health and wellness experiences: Specificities and consequences of connective gaps in self-racking data	2018	DIGITAL HEALTH	Yli-Kauhaluoma, S and Pantzar, M	10.1177/2055207618779714	This study examines situations when self-trackers face difficulties in engaging with, and reflecting on, their data with the aim of identifying the specificities and consequences of such connective gaps in self-tracking contexts.	Qualitative	Finland	General public	The age of the interviewees ranged from 28–57 years (nine male, 18 female).	health and wellness self tracker										[illegible] tracking devices actively search for constant connectivity to their everyday experiences and particularly health and wellness through personal data but often become disappointed. The results suggest that in connective gaps the personal data remains invisible or inaccurate, generating feelings of confusion and doubt in the users of the self-tracking devices. These are alarming symptoms that may lead to indifference when disconnectivity becomes solidified and data ends up becoming dead, providing nothing useful for the users of self-tracking technologies.<br><br>Conclusions<br>High expectations which are put on wearables to advance health and wellness may remain unmaterialised due to connective gaps. This is problematic if individuals are increasingly expected to be active in personal data collection and interpretation regarding	N
The quality of health and wellness self-tracking data: A consumer perspective	2022	JOURNAL OF THE ASSOCIATION FOR INFORMATION SCIENCE AND TECHNOLOGY	Zhang, Y and Trace, CB	10.1002/asi.24591	Information quality (IQ) is key to users' satisfaction with information systems. Understanding what IQ means to users can effectively inform system improvement. We identified eight dimensions that consumers apply to evaluate self-tracking data quality: value-added, accuracy, completeness, accessibility, ease of understanding, trustworthiness, aesthetics, and invasiveness.	Qualitative	USA	General public	Among the 20 participants, most were female, aged from18 to 64, Non-Hispanic White, and had a high level ofeducational attainment (Table2). Their occupationsincluded college student, academic program coordinator,student academic advisor, postdoctoral research fellow,editor, development specialist, investment manager, andexplorer. Most of them used an iPhone and had an activelifestyle	health and wellness self-tracking data				[illegible] apply to evalu-ate self-tracking data quality: value-added, accuracy, completeness, accessibil-ity, ease of understanding, trustworthiness, aesthetics, and invasiveness. Thesedimensions fell into four categories—intrinsic, contextual, representational,and accessibility—suggesting that consumers judge self-tracking data qualitynot only based on the data's inherent quality but also considering tasks athand, the clarity of data representation, and data accessibility. We also foundthat consumers' self-tracking data quality judgments are shaped primarily bytheir goals or motivations, subjective experience with tracked activities, mentalmodels of how systems work, self-tracking tools' reputation, cost, and design,and domain knowledge and intuition, but less by more objective criteria suchas scientific research results, validated devices, or consultation with experts.							UNDECLARED
Exploring Access as a Process of Adaptation in a Self-Monitoring Perinatal eHealth System: Mixed Methods Study From a Sociomaterial Perspective	2023	JMIR FORMATIVE RESEARCH	Auxier, J and Savolainen, KT and Bender, M and Rahmani, AM and Sarhaddi, F and Azimi, I and Axelin, AM	10.2196/44385	We examine the patient engagement process of access occurring during the adaptation of eHealth self-monitoring use from a sociomaterial perspective.	Mixed	Finland	pregnant women	Characteristics High-use group (n=14) Low-use group (n=6)<br>Age (years), mean (SD) 32 (2.42) 29 (3.01)<br>BMI, mean (SD; range) 24.95 (3.49; 17.43-31.64) 26.48 (6.92; 20.96-39.84)<br>Number of children, n (%)<br>1 6 (43) 2 (33)<br>2 3 (21) 1 (17)<br>0 5 (36) 3 (50)<br>Frequency of other mobile app use in daily life, n (%)<br>Daily 8 (57) 3 (50)<br>Weekly 5 (36) 2 (33)<br>Monthly 1 (7) 0<br>Rarely 0 1 (17)	a Self-Monitoring Perinatal eHealth System				When the data recordings were not in agreement with women's own bodily perceptions or knowledge of events, they often referred less to the app for information. Participants described a process of adapting after receiving access to the eHealth self-monitoring system. The process incorporated personal choices to use the system, depending on eHealth literacy, comfort with the technology, and interest in eHealth, as a tool for health promotion. Participants expressed that personal self-awareness, perceptions of trusting their own bodies, and participation in goal setting activities emerged as important elements of using the eHealth system. Women in the high-use group found themselves becoming focused on the practical skills of monitoring and interpreting data. Unlike the low-use group, this active focus and concentration on maintaining good self-monitoring techniques made it difficult for them to use the data initially in relevant ways for their own health promotion. Women in the low-use group were instead concerned about whether they could incorporate this device into their daily lives, for example, remembering where they put the ring	The women who found the use of their mother tongue helpful stated that they could internalize the feedback better and that this added clarity to the messages given by the app. Some women expressed that they struggled to always wear this ring because the size and style appears to be best suited to men or it was too big and sometimes caught on items or their children during handling.				Use Related to Self-Awareness Participants began looking at their daily activity and sleep pattern data using the app. Women refer to bodily self-awareness as critical to adapting to the daily viewing of personal health data. Some women continued to be interested in seeing the daily sleep and activity patterns and told the researcher that they liked becoming aware of their own daily habits. Multiple women in the high-use group stated that they never knew they slept poorly before using the service and that after learning this, they gave themselves permission to rest more. Many women in the high-use group discussed that they had confidence in their own body rhythms and health habits and felt that the App was used for reassurance, that things were as well as they felt them to be. Some participants also noted that this system would be well suited to persons with some health challenges but maybe not needed for someone like them.		N

Patients' perception towards chronic disease self-management and its program: A cross-sectional survey	2023	CHRONIC ILLNESS	Halim, NAA and Sopri, NHA and Wong, YY and Mustafa, QM and Lean, QY	10.1177/17423953231185385	This study aimed to assess the perceptions towards chronic disease self-management and its program among patients with chronic diseases.	Quantitative	Malaysia	Patients with chronic disease	completed the survey (Table 1). The mean age of respondents was 47.7 ± 12.8 years old with more than half (56.7%) of the respondents being female. The majority of them were Malays (58.9%) followed by Chinese (23%), and Indians (16.3%). Over half of the respondents (51.5%) had attended college/university but some (5.2%) with no formal education. A total of 30.4% of them reported to have monthly income of RM 2000 – RM 2999 and 91.1% lived with their family members.	chronic disease self-management and its program			The most preferred method was mobile application (58.5%), followed by hands-on self-management skills training (57.4%) then online learning via website and emails (55.6%) and patient's sharing session (51.9%). While the least preferred method was telephone-based support (38.1%).					Near to half of the participants preferred individual sessions (44.8%). Most of the respondents wanted the session once monthly (37.8%) with up to five sessions (90.4%), with each should last for 1–2 h (82.2%). They preferred doctors and other healthcare providers (48.2%) or community health workers (38.5%) rather than peers (13.3%) as educational providers. They also believed that the government (80.0%) should bear the cost of the program, with only 7.4% willing to self-pay. In the case of self-payment, most were willing to spend less than RM100 (87.0%) for the program.				N
Barriers and Facilitators of Using Sensored Medication Adherence Devices in a Diverse Sample of Patients With Multiple Myeloma: Qualitative Study	2018	JMIR CANCER	Asfaw, AA and Yan, CH and Sweiss, K and Wirth, S and Ramirez, VH and Pate, PR and Sharp, LK	10.2196/cancer.9918	This study aimed to explore patients' (1) perceptions of their health before multiple myeloma (MM) including experiences with chronic medications, (2) perceptions of adherence barriers and facilitators, and (3) attitudes toward using sensored medication devices (SMDs).	Qualitative	USA	Patients With Multiple Myeloma	Sex, n (%)<br>Female 9 (45)<br>Male 11 (55)<br>Age in years<br>Median 59<br>Range 29-71<br>Race/ethnicity, n (%)<br>African American 16 (80)<br>White 3 (15)<br>Hispanic/Latino 1 (5)<br>Marital status, n (%)<br>Married 10 (50)<br>Single 8 (40)<br>Widow 1 (5)<br>Divorced 1 (5)<br>Type of insurance, n (%)<br>Private 7 (35)<br>Medicaid 6 (30)<br>Medicare B 6 (30)<br>Uninsured 1 (5)	Sensored Medication Adherence Devices			Patients initial reactions to being presented with the SMDs were split with half reacting positively.<br><br>Although virtually everyone had a concern or doubt about the SMDs, when asked if they would be interested in trying a bottle in the future, 12 (60%, 12/20) patients expressed a willingness to test one out.		Interestingly, several of the same SMD features were viewed positively by some and negatively by others. For example, participants were informed of the reminder features of SMDs such as text messaging and audio or visual alerts. and a 67-year-old white married male who described himself as being comfortable with technology expressed that the SMDs made "a simple task too complicated." He highlighted that some older people, not himself, would find the SMDs "too high tech." Another pointed out that "many older people don't have their cell phones with them all the time," making the text reminders ineffective. Others focused on the bottle closure, suggesting that the lids would be difficult for people with arthritis to open or that they lids were not childproof. Furthermore, 2 patients were concerned that the cost of medications might go up if patients were asked to use SMDs. Finally, the 68-year-old African American married male stated that he was willing to use an SMD but was very clear that it was not foolproof. He expounded upon how someone could take out medication and never actually ingest it. As a result, he felt the technology was fatally flawed.			In addition, 3 patients were very satisfied with their current approach to medication management and simply did not like the idea that using an SMD required them to change their system. Moreover, 5 patients were not interested in using an SMD because they traveled or were out of the house often. They did not want to carry the bottle with all the pills when they only needed a few doses. They were concerned that if they removed all of the pills that they needed during their travels and left the bottle at home, this would be recorded inaccurately. Although seeing no personal benefit to using an SMD, these patients recognized the potential benefit to individuals who were older, had dementia, lived alone, or were otherwise struggling to remember to take their medications.		The 60-year-old African American female who resided in a shelter and was open about her poor medication adherence felt that the SMDs would help her look forward to taking her medications. She found the technology novel and exciting. Moreover, 3 people mentioned that they liked the fact that they could "see what was going on," referring to the Web-based platform that plotted the day and time that the bottle was open.A total of 2 patients were excited about the reminder alerts and 1 acknowledged that the alerts would be beneficial as she was already using her cell phone for this function; however, 3 (15%, 3/20) patients felt the alerts would be annoying, as expressed in other similar ways that can be summarized as "I don't want that bottle talking to me/beeping at me." Similarly, 4 (20%, 4/20) patients perceived they would benefit by having the provider notified of any missed doses. They liked the idea of having their providers gain access to the SMD data to "help monitor" them.	Several additional concerns were expressed even by those who were enthusiastic about the SMDs. Not surprisingly, privacy issues were identified by several patients. Some felt using the devices would be invasive even if used for a good reason, Furthermore, they felt that it suggested the provider did not trust what the patient told them.	
Explaining engagement in self-monitoring among participants of the DESMOND Self-monitoring Trial: a qualitative interview study	2015	FAMILY PRACTICE	Eborall, HC and Dallosso, HM and McNicol, S and Speight, J and Khunti, K and Davi, MJ and Heller, SR	10.1093/fampra/cmv060	To explore experiences of SMBG and urine monitoring following structured education. We specifically addressed the perceived usefulness of each monitoring method and the associated well-being.	Qualitative	UK	Diabetes patients	Eighteen adults with newly diagnosed type 2 diabetes who were participating in the DESMOND Self-monitoring Trial took part in the interview study. Ten had been allocated to SMBG and eight to urine self-monitoring; seven were women, and the age range was 29–80 years.	DESMOND Self-monitoring Trial					Interviewees reported SMBG as accurate, convenient and useful. Declining use was explained by having established a pattern of managing blood glucose with less frequent monitoring or lack of feedback or encouragement from health care professionals.<br>Many initially positive views of urine monitoring progressively changed due to perceived inaccuracy, leading some to switch to SMBG. Perceiving diabetes as less serious was attributable to lack of symptoms, treatment with diet alone and—in the urine-monitoring group—consistently negative readings. Urine monitoring also provided less visible evidence of diabetes and of the effect of behaviour on glucose.<br>Conclusions.<br>The findings highlight the importance for professionals of considering patients' preferences when using self-monitoring technologies, including how these change over time, when supporting the self-care behaviours of people with type 2 diabetes.							have no conflicts of interest. JS is a member of the Roche Diagnostics Australia (RDA) Accu-Chek Advisory Board. In the past 3 years, her research centre has received consultancy fees from RDA in respect of her attendance at advisory board meetings, as well as from Abbott Diabetes Care, Eli Lilly, Johnson & Johnson, Medtronic, Merck Sharp and Dohme, Novo Nordisk, RDA and Sanofi Diabetes, for speaking engagements and/or unrestricted educational grants. KK has acted as consultant and speaker for AstraZeneca, Boehringer Ingelheim, Janssen, Lilly, MSD, Novartis, Novo Nordisk and Sanofi. He has received grants in support of investigator initiated trials from AstraZeneca, Boehringer Ingelheim, Lilly, Novartis, Novo Nordisk, Roche and Sanofi. MJD has acted as consultant, advisory board member and speaker for Novo Nordisk, Sanofi-Aventis, Lilly, Merck Sharp & Dohme, Boehringer Ingelheim, Janssen and Astra Zeneca and as a speaker for Mitsubishi Tanabe Pharma Corporation. She has received grants in support of investigator and investigator initiated trials from Novo Nordisk, Sanofi-Aventis and Lilly. SRH reports
See I told you I was taking it! - Attitudes of adolescents with asthma towards a device monitoring their inhaler use: Implications for future design	2017	APPLIED ERGONOMICS	Howard, S and Lang, A and Sharples, S and Shaw, D	10.1016/j.apergo.2016.06.018	This paper considers the positive and negative perceptions of this novel device and provides new insight into the attitudes of adolescents towards inhaler monitoring, as well as future directions for design and development of monitoring devices for asthma and other chronic medical conditions.	Mixed	UK	adolescents with asthma	Seven adolescents with asthma took part in the study. Their ages ranged from 11 to 16 years (mean of 13.4). Five of the participants were male and two were female. All seven participants used a seretide MDI and were required to administer two doses twice daily as part of their typical treatment regime.	a device monitoring their inhaler use			As can be seen from Fig. 3, for all seven themes in both adolescent participants and their parents, there were noticeably more positive responses compared to negative. the parents were in greater agreement with statements related to this theme compared to their children.		Tests showed there was no significant difference between adolescents and parents for 'Ease of Use' (p > 0.025). However, there was a tendency for the adolescents to be more critical for this theme, with 3 'strongly disagree' responses and 21 'disagree' responses compared to only 1 'strongly disagree' and 8 'disagree' for the parents (see Fig. 3). This suggests that this may be an important factor to take into consideration for the adolescents.<br><br>Also of note in the data, although not statistically significant, is the larger amount of 'strongly disagree' responses from the adolescents (13) for the theme 'Aesthetics' compared to the parents (2) (see Fig. 3). This again indicates that this may be an important factor to take into consideration when designing devices for this demographic.		Participants provided suggestions and recommendations for how the SmartTrack device could be improved to more effectively meet their needs. Subthemes here were; better reminders, device aesthetics and usability.	there was general concern for the social acceptability of the device in terms of its appearance. Subthemes here were: drawing of attention, unwanted questioning and influencing likelihood of use.		Participants expressed enthusiasm for utilising the SmartTrack device to help them take control of their asthma. Subthemes here were: utilising data for proof of independence, demonstrating responsibility, and reminders to facilitate better adherence.When discussing the SmartTrack's ability to record information on their inhaler use, participants spoke of the effects that sharing this information could have as well as the way they would like to receive feedback on their adherence. Subthemes here were: Awareness of monitoring; communicating and sharing with others; and validity of data on inhaler use.		N
A formative study exploring perceptions of physical activity and physical activity monitoring among children and young people with cystic fibrosis and health care professionals	2018	BMC PEDIATRICS	Shelley, J and Fairclough, SJ and Knowles, ZR and Southern, KW and McCormack, P and Dawson, EA and Graves, LEF and Hanlon, C	10.1186/s12887-018-1301-x	The research aimed to explore patients' perceptions of physical activity (PA) and the acceptability of using PA monitoring devices with children and young people with Cystic Fibrosis (CF).	Mixed	UK	children and young people with cystic fibrosis and health care professionals	P Age Group (Years) Gender Allocated device(s) Compliance (Days worn) Valid days included (≥10 h·day) Mean wear time (h)<br>1 ≤10 Female ActiGraph & Pedometer 6 4 12.5<br>2 >10 Male ActiGraph & Fitbit<br>3 >10 Male ActiGraph & Pedometer<br>4 >10 Male GENEActiv 7 6 16.3<br>5 ≤10 Female ActiGraph & Pedometer<br>6 >10 Male GENEActiv & Smartphone<br>7 >10 Female GENEActiv 6 6 13.3<br>8 ≤10 Male GENEActiv & Fitbit 7 4 23.3<br>9 >10 Female GENEActiv & Fitbit 7 6 14.8	physical activity and physical activity monitoring among children and young people with cystic fibrosis and health care professionals					participants. Participants reported finding the "watch-like" designs easier to wear, with some participants citing issues with the fitting, positioning and comfort of the ActiGraph hip-worn device.Participants trialling the GENEActiv commented on the thickness of the wrist strap, some finding it interfered with activities. The visual appearance of the devices often attracted attention from others. This was not perceived to be an issue by some participants, though for some older participants this attention was unwanted, with one opting to tell inquisitive others that the device was a broken watch.**he LED display of the consumer devices was reported as a desirable feature, with participants enjoying the interactive nature of these devices.**<br><br>**Other participants found the level of information available and interactive dashboard to be overwhelming and complicated, with some opting not to use it at all.**<br><br>**P6 reported that the smartphone based application provided sufficient PA-related information and was simple to use.**<br><br>**The research-based devices do not provide participants with PA-related**						Participants opted to remove the devices during activities due to concerns about damaging the devices and/or causing injury to others.	N
Microdialysis-based 48-hour continuous glucose monitoring with GlucoDay (TM): Clinical performance and patients' acceptance	2006	DIABETES TECHNOLOGY & THERAPEUTICS	Kubiak, T and Worle, B and Kuhr, B and Nied, I and Glasner, G and Hermanns, N and Kulzer, B and Haak, T	10.1089/dia.2006.8.570	This study was designed to assess clinical performance and patients' acceptance of the minimally invasive microdialysis-based continuous glucose monitoring system GlucoDay(TM) (Menarini Diagnostics, Florence, Italy) with a targeted monitoring time of 48 h.	Quantitative	Germany	Diabetes patients		Microdialysis-Based 48-Hour Continuous Glucose Monitoring with GlucoDay			All patients statedthey would wear the device again		Figure 4 shows the results of the scales as-sessing pain during catheter application andexperienced discomfort while wearing the de-vice. Whereas hardly any pain was associatedwith the catheter application, a mean discom-fort of 3.6 on the 5-point scale was observedwhile wearing the device.							UNDECLARED
Knowledge and Practice of Glucose Self-Monitoring Devices among Patients with Diabetes	2023	SUDAN JOURNAL OF MEDICAL SCIENCES	Mohamed, S and Mukhtar, M and Aseed, K and Osman, D and Dawelbait, M and Saeed, A and Ahmed, N and Fadul, N and Idriss, R	10.18502/sjms.v18i2.13598	With blood glucose self-monitoring, patients may assess how well their glucose level is controlled, and change their treatment plan as advised by their physicians.The study explores the challenges for people using self-monitoring devices.	Quantitative	Sudan	Diabetes patients	Moreover, 60% of the patients were males while 40% were females. Most (32.8%)<br>were in their fifth decade; 24.8% in their fourth, 22.4% were >60 years of age, 8.8% were<br>in their third decade, and 11.2% were <30 years old. In addition, 44 (35.2%) patients were<br>unemployed, while the rest were either students (4.8%), employees (17.6%), free workers<br>(33.6%), or retired (8.8%). While 21.6% of patients were illiterate, 28% were at primary<br>school level, 23.2% at secondary school level, and 27.2% at collage level. Majority of<br>patients (71, 56.8%) were diagnosed with diabetes <10 years ago (Table 1).	Glucose<br>Self-Monitoring Devices among Patients with<br>Diabetes	The study revealed that 82 patients (65.6%) had prior basic knowledge about glucose<br>self-monitoring devices, while the rest (34.4%) had none. Sources of information were<br>self-learning (6, 4.8%), physician education (38, 30.4%), pharmacists (7, 5.6%), and<br>relatives (34, 27.2%) (Table 1). Overall, 67 (53.6%) patients used a glucose self-monitoring<br>device before, while 58 (46.4%) had not. Most patients (32) who had used devices before<br>were taught by their physicians, while others were taught by their relatives, neighbors, pharmacists, or had taught themselves through self-learning methods (Table 2).							In total, 37 (29.6%) patients<br>considered that financial reasons constitute a major challenge preventing them from<br>gaining access to a glucose self-monitoring device, while others (19, 15.2%) chose lack<br>of prior knowledge and education, and significant number of patients (52, 41.6%) could<br>not provide a specific challenge				N
Stakeholder Perspectives on In-home Passive Remote Monitoring to Support Aging in Place in the Province of New Brunswick, Canada: Rapid Qualitative Investigation	2022	JMIR AGING	Read, EA and Gagnon, DA and Donelle, L and Ledoux, K and Warner, G and Hiebert, B and Sharma, R	10.2196/31486	This study aimed to explore perceptions of in-home passive remote monitoring technology designed to support aging in place from the perspective of older adults, their family or friend caregivers, social workers, and government decision-makers in the province of NB, Canada.	Qualitative	Canada	Older adults	Of the 11 caregiver participants, 9 (82%) were adult children caring for parents, 1 (9%) was caring for a relative or sibling, and 1 (9%) was caring for both a parent and a sibling. Of the 11 caregivers, 1 (9%) lived with the client, 2 (18%) lived outside of the province, and 8 (73%) lived in close proximity to the client. Approximately 55% (6/11) of caregivers reported their loved ones having a camera in their home. Of the 8 social worker participants, 6 (75%) had received a 1-hour training session from either the passive remote monitoring company or the local contractor hired to service the passive remote monitoring company's clients. None of the participants confirmed that they had participated in the pilot study. In the decision-maker group, of the 7 participants, 3 (43%) were former social workers who had clients using the remote monitoring system, and 2 (29%) were hired by a community organization	In-home Passive Remote Monitoring to Support Aging			Approximately 29% (2/7) of the decision-maker participants in our study were directly involved in bringing the remote monitoring system to the province.<br><br>Although the fit was identified as being very important, social workers were divided on their perceptions of which clients they should recommend passive remote monitoring to. Cognitive status and wandering behaviors were also considered by the social workers when making their recommendations. Some viewed the technology as an excellent service for clients who needed high levels of supervision and felt the technology could delay or avoid residential care. In contrast, some social workers felt that clients with substantial cognitive impairments and a high propensity to wander were too risky for passive remote monitoring as they always needed a person with them or at least the caregiver to continually monitor their electronic devices for any notifications.					Both decision-maker participants indicated that there were few barriers to government adoption. However, they noted a lack of awareness of the service. Barriers to Adopting CareLink Advantage Perceived barriers to the adoption of CareLink Advantage included a lack of awareness and knowledge of the service, preference for traditional or familiar home support services, additional caregiving responsibility required, hesitancy to promote a service offered by a private company, costs, privacy, and language.Decision-makers felt that the biggest barrier to adoption was communication between social workers and caregivers or clients about the service. They identified several structural factors that influenced social workers' knowledge about the passive remote monitoring system and their ability to discuss it with their clients and caregivers. Social workers' gaps in knowledge about available services were thought to be the result of a combination of high turnover and insufficient orientation to the services. For example, one of the decision-makers shared the following:<br><br>In addition, decision-makers felt that because of the social workers' demanding workloads, they had little time to learn more about the passive remote monitoring system or take on the responsibility of teaching or guiding caregivers about the technology:<br><br>There was a sense that social workers, clients, and caregivers were	Social workers played a key role in determining who was a good fit for the service based on their assessment and circumstances and shared information about the system only when they thought it would be appropriate.<br>Family or friend caregivers were also identified by almost all participants as playing a key role in deciding to adopt the remote monitoring system.	However, all 4 groups reported that clients using the passive remote monitoring system were able to stay in their homes longer or completely avoid going to a nursing home<br>Decision-makers highlighted that it supported client choice:<br>Clients also reported increased accessibility to emergency services when required and increased peace of mind and sense of security.<br>Caregivers stated that the passive remote monitoring system allowed them to keep track of evolving care needs such as increased supervision, maintaining client routines, ensuring adequate nutrition, and assisting with adherence, adjustments to treatment, and medication protocols through observation of changes in behaviors such as increased sleep in older adults.<br>Similarly, social workers identified CareLink Advantage as facilitating the tracking of evolving client care needs. They emphasized that although the passive remote monitoring system helped keep clients home longer, at some point, institutional placement was often unavoidable when care needs were beyond what home support services could offer. Interestingly,	Interestingly, clients themselves had few personal privacy concerns about the monitoring, although they acknowledged that caregivers and visitors coming into their homes might feel it was an intrusion.<br><br>On the other hand, family or friend caregivers were concerned about the privacy of the client but felt that this was outweighed by the additional sense of security and honoring their wish to stay in their own home.<br><br>Consistent with the views of the clients in our study, some caregivers reported that clients did not mind the cameras and sensors and did not have objections related to privacy. They also identified the privacy of paid caregivers coming into the home as a concern.<br><br>Social workers' concerns about privacy were centered on the use of technology, which conflicts with provincial privacy legislation. These concerns focused mainly on the video components of the system	N
Feasibility, Acceptability, and Performance of a Continuous Temperature Monitor in Older Adults and Staff in Congregate-Living Facilities	2022	JOURNAL OF THE AMERICAN MEDICAL DIRECTORS ASSOCIATION	Kirkendall, ES and McCraw, J and Ganesh, S and Lang, S and Mariotti, M and Evered, M and Ghoreyshi, A and Williamson, J and Zamora, Z	10.1016/j.jamda.2022.03.004	Residents of congregate-living facilities are susceptible to disability and mortality from infection given the presence of advanced age, multimorbidity, and frailty-as demonstrated in the recent COVID pandemic. This study assessed the feasibility, acceptability, and applicability of a continuous temperature monitoring device in a congregate-living facility with residents of independent living, assisted living, and their care-providing staff. We hypothesized that a wearable device compared with daily manual temperature assessment would be well tolerated and more effective at detecting temperature variances than current standard of care body temperature assessment.	Quantitative	USA	Older adults	Table 1. Characteristics of Study Participants Enrolled<br><br>Characteristics All Participants Staff Older Adult Residents Characteristic Older Adult Residents<br>No. of participants 35 10 25 No. of participants* 17<br>Gender Male: 10 Male: 0 Male: 10 Total medical conditions<br>Female: 25 Female: 10 Female: 25 Mean 8.4<br>Age (mean ± SD) 68.2 ± 22.7 33.9 ± 10.1 81.9 ± 4.9 Min–Max 0–19<br>Race White: 33 White: 8 White: 25 Total medications<br>Asian: 1 Asian: 1 Mean 7<br>Black: 1 Black: 1 Min–Max 2–13<br>Phone Android: 15 Android: 2 Android: 13 Total allergies<br>Operating system iOS: 25 iOS: 8 iOS: 12 Mean 1.9<br>Min–Max 0–12	a Continuous Temperature Monitor in Older Adults and Staff in Congregate-Living Facilities					Five participants (all independent living) discontinued the patch before the 90-day duration was completed, for a total participant completion rate of 85.7% (30 of 35). No staff participants or resident participants were lost to follow-up, and the completion rate for staff was 100%. The rate for residents was 80% (20 of 25). According to our a priori predefined outcome criteria, completion rates for staff were 90% and residents were slightly lower at 80%. Device discontinuation reasons included the following: device lost by participant (1), patch connection/pairing problems (1), discontinuation because of skin irritation (1), unable to synchronize mobile phone after a week (1), and reason for discontinuation unknown (1).							UNDECLARED
It's made me a lot more aware': a new materialist analysis of health self-tracking	2019	MEDIA INTERNATIONAL AUSTRALIA	Lupton, D	10.1177/1329878X19844042	With the advent of apps, other software and wearable devices designed to enable people to easily monitor and measure details about their bodies, much attention has been paid to the phenomenon of health self-tracking. In this article, findings from a study involving interviews with 40 Australian self-trackers are discussed and analysed from a feminist new materialist perspective, focusing on relational dimensions, affective forces and agential capacities.	Qualitative	Australia	General public	To ensure an even gender and age spread, sub-quotas were set of 20 men and 20 women, 20 participants aged 40 or below and 20 aged above 40 (the oldest participant was aged 75). The vast majority of participants who agreed to participate resided in the most populous states of Victoria (45%) and New South Wales (48%), and in the capital cities of those states: 80% of the participants came from either Sydney or Melbourne, with the others hailing from Adelaide, Brisbane or regional cities in New South Wales or Victoria. More than half held a university undergraduate or postgraduate degree (53%).	chronic health condition through self-tracker						The pleasure and satisfaction they derived from these practices were key to continuing them as a part of their everyday routines. These affective forces were typically a part of efforts to 'feel more in control'.<br>In these accounts and many others, the idea of the self-responsible citizen dominated.<br>Personal relationships were also a part of the affective forces driving people's practices, as were their self-identities.				The participants' responses to the open-ended questions gave further insights into their practices and rationales for monitoring their bodies. People often referred to becoming 'more aware' of their bodies and how this awareness helped them manage aspects they found problematic. Apps could help with establishing and maintaining this awareness, but simple habits of taking notice of one's body and attempting to remember details from day-to-day were also commonly employed. People's bodily sensory capacities were augmented and extended by the technological affordances of the apps and other devices they used to become more aware of their bodies.	The burden of self-tracking<br>Self-tracking a chronic health condition or other body metrics as a part of preventive health efforts can be time-consuming and requires continued effort.<br><br>Disappointments and frustrations<br>There are other negative aspects to monitoring one's body and health and illness states. It can sometimes generate information that is confronting or disappointing. If the information generated from self-tracking practice suggests that health conditions are degenerating, weight has been gained, blood glucose levels not well managed and so forth – even when the individual has done the best they can to make the numbers 'good' – the affective charge of these insights can be dispiriting. These participants continue to monitor themselves, however, because they also receive value from their practices.	N
I guess I was surprised by an app telling an adult they had to go to bed before half ten': a phenomenological exploration of behavioural 'nudges'	2022	QUALITATIVE RESEARCH IN SPORT EXERCISE AND HEALTH	Toner, J and Allen-Collinson, J and Jones, L	10.1080/2159676X.2021.1937296	In recent years, the role of self-tracking technologies has been investigated, debated and critiqued within qualitative research circles. The principal means by which self-tracking technologies seek to promote health-related behaviours and behaviour change is through the use of 'nudges'. Despite the increasing prevalence of nudge-style modes of body-mind governance, there remains little in-depth qualitative research on people's embodied responses to this form of behavioural management. The current study sought to address this lacuna by drawing on a form of empirical, sociological phenomenology to investigate the lived experience of being 'nudged' by self-tracking technology.	Qualitative	UK	General public		Fitbit ALPHA, MiFit or Apple Watch Series 3					Participants were well aware they had been 'nudged', but on many occasions they proceeded to resist or ignore nudges deemed 'inappropriate'.<br><br>Achieving targets or goals, or receiving positive reinforcement from the device (e.g., via the receipt of virtual rewards) also seemed to hold little meaning for participants. They reported gaining little satisfaction from this form of 'patronising' reward process, which had little impact upon their physical-activity levels:<br><br>The meaningfulness and relevance of the prompts were therefore highly context-dependent and participants took into account their knowledge of past and future activity levels.						Nudges in the form of visual and/or haptic sensations, for example, seek to bring the body, and various bodily processes, to the foreground of conscious reflection. Unfortunately, nudges may prove disruptive because they are, by their very nature, objectifying in rendering the user's body the intentional object of awareness.	N

Algorithmic Self-Tracking for Health: User Perspectives on Risk Awareness and Coping Strategies	2021	MEDIA AND COMMUNICATION	Festic, N and Latzer, M and Smirnova, S	10.17645/mac.v9i4.4162	This article conceptualizes self-tracking as algorithmic-selection applications and empirically examines users' risk awareness related to self-tracking applications as well as coping strategies as an option to deal with risks.	Quantitative	Switzerland	General public		Self-tracking with wearable devices and mobile applications											Overall, awareness of the surveyed risks was low:<br>About four out of ten (39%) to seven out of ten (69%) self-<br>tracking users were not concerned about the risks asso-<br>ciated with their self-tracking practice. For overuse and<br>lack of transparency, "do not agree at all" was the modal<br>category: About half of the internet users did not agree at<br>all that they use their tracking device too much (48%) and<br>disagreed or fully disagreed that they do not know how<br>their application calculates health results (54%). Loss of<br>control over data and measurement inaccuracy were dif-<br>ferent in that the responses were roughly equally dis-<br>tributed: 27% and 30%, respectively, agreed (4) or fully	N
Employees' Acceptance of the Healthcare Internet of Things: A Source of Innovation in Corporate Human Resource Policies	2019	JOURNAL OF INNOVATION ECONOMICS & MANAGEMENT	Baudier, P and Ammi, C and Lecouteux, A	10.3917/jie.030.0089	The IoT is pervading our daily personal and professional lives. This article attempts to fill a gap in the field of IoT by investigating the use of wearable devices as a source of innovation in corporate HR policies.	Quantitative	France	Employee	natives (respondents born after 1980), and 68% digital immigrants (respondents born before 1980). The gender breakdown of our sample shows a higher proportion of female respondents, with 65% being women, versus 35% men. We therefore assume that the topic of health and wellness is of greater interest for women than for men (Waldron, 1988). We also observed a high level of education among our respondents, with 74% of respondents holding a five-year university degree/bachelor's degree.	Healthcare Internet of Things				Therefore, the fact that the wearable technology is perceived as easy to use and that it is your own decision to use it because you consider it to be interesting, directly impact the PU. The Q2 of Attitude (0.287), BIU (0.531), Motivations (0.458), and PU (0.301), all above 0, confirmed the predictive relevance of our model.								UNDECLARED
Monitoring Parkinson's disease symptoms during daily life: a feasibility study	2019	NPJ PARKINSONS DISEASE	Heijmans, M and Habets, JGV and Herff, C and Aarts, J and Stevens, A and Kuijf, ML and Kubben, PL	10.1038/s41531-019-0093-5	Parkinson's disease symptoms are most often charted using the MDS-UPDRS. Limitations of this approach include the subjective character of the assessments and a discrepant performance in the clinic compared to the home situation. Continuous monitoring using wearable devices is believed to eventually replace this golden standard, but measurements often lack a parallel ground truth or are only tested in lab settings. To overcome these limitations, this study explores the feasibility of a newly developed Parkinson's disease monitoring system, which aims to measure Parkinson's disease symptoms during daily life by combining wearable sensors with an experience sampling method application.	Quantitative	Unspecified	patients with Parkinson's disease		Monitoring Parkinson's disease symptoms during daily life					the wrist (60%) and the chest (80%) sensors were comfortable to wear. However, some participants mentioned that the used accessories remained attached to clothes, or that they caused irritation. Two participants (10%) reported that the wrist sensors impaired their arm movement. According to all participants, the wrist sensors did not impair hand movement nor did the three sensors impair movements in general. None of the participants found the sensors were heavy to wear, had problems with putting the sensors on and off, or had problems with charging the sensors. Almost all participants (95%) wore the sensors in public. Some participants mentioned that they thought it was awkward to get questions about the sensors, and therefore they were glad that it was possible to wear the sensors beneath their clothes. Only 30 and 50% of the participants was willing to wear the wrist and chest sensors on a long-term basis. For example, a score of 1 was sometimes the most positive option and sometimes the most negative option. Three participants (15%) found it unpleasant to carry their phone with them all day, for example because they could not due to work. Further, 17 participants (85%) did not mind that the continuous questionnaires showed up at random moments during the day, which was supported by the average score of only 1.7 on the question 'I found this beep disturbing'. This item was scored on a 7 point Likert scale, where 1 corresponded with 'not disturbing at all' and 7 corresponded with 'very disturbing'. Three participants (15%) thought they missed a lot of questionnaires and ten participants (50%) said they would use the ESM application for a longer period than two consecutive weeks.							N
Older adults' readiness to engage with eHealth patient education and self-care resources: a cross-sectional survey	2018	BMC HEALTH SERVICES RESEARCH	Gordon, NP and Hornbrook, MC	10.1186/s12913-018-2986-0	This study examined access to digital technologies, skills and experience, and preferences for using web-based and other digital technologies to obtain health information and advice among older adults in a large health plan. A primary aim was to assess the extent to which digital divides by race/ethnicity and age group might affect the ability of a large percentage of seniors, and especially those in vulnerable groups, to engage with online health information and advice modalities (eHIA) and mobile health (mHealth) monitoring tools.	Quantitative	USA	Older adults	We conducted the survey with a stratified random sample of 5420 KPNC members aged 65–79 who had a primary language of English in their electronic medical record. The initial sample included 1420 non-Hispanic white (white), 1500 African-American/black (black), 1500 Hispanic/Latino (Latino), 500 Filipino, and 500 Chinese adults. Each racial/ethnic group had approximately equal numbers of men and women in three age strata (65–69, 70–74, and 75–79). The Filipino and Chinese samples were smaller than the others because their data were originally intended to be used for pilot study purposes. Due to requirements of the larger patient portal use study of which this survey was one component, the survey sample was restricted to members who had been continuously enrolled in KPNC for at least 2.5 years at the time of the survey.The sample is predominantly white (79%) and educated beyond high	online health information and advice modalities (eHIA) and mobile health (mHealth) monitoring tools.				Approximately 12% of seniors said they had used a health-related app on a smartphone or tablet computer in the past 12 months, but usage was lower among the older groups.<br><br>One-third of seniors (but 60% of those with a smartphone or tablet) said they had downloaded any type of app from a website at least once, but again, this declined with age, and blacks, Latinos, and Filipinos were less likely than whites to have done so.								N
Unobtrusive in-home monitoring of cognitive and physical health: Reactions and perceptions of older adults	2008	JOURNAL OF APPLIED GERONTOLOGY	Wild, K and Boise, L and Lundell, J and Foucek, A	10.1177/0733464807311435	Although the potential benefits of unobtrusive in-home sensing technologies for maintaining health and independence of older adults have been highlighted in recent research, little is known about their views toward such technology. The aims of this project were to identify monitoring needs and expectations of community- residing elderly and their family members.	Qualitative	USA	Older adults	Participants were selected from a group of subjects being followed in the Community Brain Donor Program (CBDP) of the OHSU Layton Aging & Alzheimer's Disease Center. Those who met the research criteria were sent a letter in the mail, introducing the purpose of the study and requesting that they call the CBDP coordinator if they did not wish to be contacted about the study. Each individual who agreed to participate was asked to bring a spouse, other adult family member, or friend to participate in a concurrent (Family member/Friend) focus group. Twenty-three older adults and 16 family member/friends agreed to participate. The average age of the participants was 80.6 (range 66–91); family member/friends' mean age was 71.5 (range 42–86).	Unobtrusive In-Home Monitoring of Cognitive and Physical Health				There were more positive than negative comments for both groups, with the proportion of positive comments relative to total rated comments tending to be higher for the family member/friend groups (70%) than for the older adult groups (58%).		Maintaining independence in the home An overarching theme in much of the discussion was the desire for older people to remain in their own homes. Of particular interest was monitoring that would identify and respond to immediate needs, such as falls. Call buttons, alarms, and responding to falls were uppermost in the minds of a number of participants.	In analyzing the comments in which specific monitoring desires were mentioned, a few topics emerged consistently. Respondents often mentioned specific medical measures such as blood pressure, heart rate, and blood sugar level in open discussion.<br><br>In general, the non-medical measures were less important to the older adults, whereas those measures were often seen as equally as useful as more medically oriented measures by their family members/friends. For example, walking, outings, and general activity level were seen as very important by the family member/friend groups, and considerably less so by the older adults. This might be interpreted as the former's desire to know if the latter is "doing OK" or "having a normal day" in addition to how well the older adult might be doing medically.	Negative comments tended to revolve around two main concerns. One was whether a "high tech" method was an improvement over the tried and true informal or "low tech" approach, as voiced by this woman: "We have a system in our church we use the buddy system. There are people…it keeps people in touch." These negative comments most frequently related to doubts about the usefulness of a monitoring system. There was substantial skepticism about whether a monitoring system would be worth the effort and expense of setting it up and analyzing the data resulting from such a system, as voiced by two older male participants: "I fail to see how in this whole network of information…you've showed us, are going to accomplish anything," and "…but what is it really going to do? It seems like a big program for a little result."<br><br>A second area of negative comments centered around the difficulty that a number of participants had in identifying with a potential future situation in which they would need to be monitored. Some participants simply could not conceptualize future deterioration in functioning, especially related to cognitive deficits: "I know pretty much what my life is and what my problems are and aren't and I don't need a piece of paper to tell me what they are." One older adult concluded, "I was going to say that at this stage I'm very active and I just don't see any need."		Positive comments were focused on the potential for informing individuals, families, and providers of changes in an elder's condition, objective updates on activities of daily living, and providing efficient emergency assistance.<br><br>Maintaining independence in the home An overarching theme in much of the discussion was the desire for older people to remain in their own homes. Of particular interest was monitoring that would identify and respond to immediate needs, such as falls. Call buttons, alarms, and responding to falls were uppermost in the minds of a number of participants.<br><br>Undetected cognitive decline Many participants discussed the value of monitoring to detect gradual decline over time that might be otherwise difficult to detect. Often, this was mentioned in the context of cognitive decline. The participants were especially attentive to the potential benefits of monitoring for addressing issues of dementia.	Sharing of information In general, both sets of groups agreed that legitimately useful monitoring was quite acceptable. There were no expressed concerns about sharing data from monitoring with one's physician. The general sentiment was that if it was useful for the doctor, then there would be no objection to collecting the information. In offering advice to monitoring technology researchers, one older male participant recommended: "Ask my doctor if he'll use it. If he felt like he needed to monitor me I would be receptive to it."<br>The question of whether information should be shared with family members received a more mixed response. In general there was recognition of how useful monitoring data could be for family members, especially for those living at a distance. Offered one older adult, "I could see where it would be a great comfort to my family… if they wanted to know how I was getting along."<br><br>Tradeoff between privacy versus usefulness	UNDECLARED
Acceptability and Usability of a Wearable Device for Sleep Health Among English- and Spanish-Speaking Patients in a Safety Net Clinic: Qualitative Analysis	2023	JMIR FORMATIVE RESEARCH	Purnell, L and Sierra, M and Lisker, S and Lim, MS and Bailey, E and Sarkar, U and Lyles, CR and Nguyen, KH	10.2196/43067	Sleep disorders are common and disproportionately affect marginalized populations. Technology, such as wearable devices, holds the potential to improve sleep quality and reduce sleep disparities, but most devices have not been designed or tested with racially, ethnically, and socioeconomically diverse patients. Inclusion and engagement of diverse patients throughout digital health development and implementation are critical to achieving health equity.This study aims to evaluate the usability and acceptability of a wearable sleep monitoring device-SomnoRing-and its accompanying mobile app among patients treated in a safety net clinic.	Qualitative	USA	patients from a mid-sized pulmonary and sleep medicine practice	Gender<br><br>Male 5 (24) 6 (27.5)<br><br>Female 4 (19) 5 (24)<br><br>Prefer not to say 0 (0) 1 (4.5)<br>Age (years)<br><br>26-35 5 (24) 5 (24)<br><br>36-45 2 (10) 3 (14)<br><br>46-55 1 (4.5) 4 (19)<br><br>≥56 1 (4.5) 0 (0)<br>Race or ethnicity<br><br>White/not Hispanic 1 (4.5) 8 (38)<br><br>Hispanic 8 (38) 1 (4.5)<br><br>Native American/Pacific Islander/other 0 (0) 3 (14)	a Wearable Device for Sleep Health Among English- and Spanish-Speaking Patients in a Safety Net Clinic	First, most participants already had prior knowledge or ownership of wearables and compared SomnoRing's ease of use to other wearables they had used.				Despite the wearable sleep device's written instructions and app only being available in English, both English- and Spanish-speaking participants were pleased with the wearable sleep device's ease of use, as demonstrated by the mean System Usability Scale score of 88 out of 100 (Table 2), implying that the wearable sleep device's characteristics are compatible with widespread use.<br><br>Overall, participants found the wearable sleep device comfortable to wear and hardly noticeable to the extent that it did not disrupt their sleep.<br><br>In addition to the simplicity of the SomnoRing, participants felt that the wearable sleep device was more accurate in capturing their quality of sleep because it caused minimal interruption to their sleep routine.	"Overall, family and peer influence served as key motivators for participants to seek help with their sleep problems, and more specific to the wearable use. Many participants' motivations to seek help for their sleep problem and, subsequently, to adhere to a wearable like the SomnoRing, were rooted in a desire to improve their sleep problem for their family's benefit.<br>The clinical care team influenced participants' acceptance and intentions to use the technology. "	Participants Still Had an Interest in Understanding the Complex Data and Metrics in the App and Needed More Assistance With Data Interpretation and Related Behavior Changes<br><br>Beyond additional interpretation of data, many felt like the app or SomnoRing should be clearer about specific and actionable behavior changes that might improve sleep quality. As it was currently presented, participants didn't feel inclined to "look into [the reports] that deep to actually try to change anything." Participants also provided constructive feedback about how to further improve the ease of use of the SomnoRing, such as by improving the fit and battery. For the battery, participants shared "the hardest part was probably plugging it in to charge it" because they often found it difficult to remember to do so. One participant reflected on how they went "straight to work, and [they] didn't charge the ring" because of the rush of the day. By forgetting to charge the ring when they came home, they found that "there [wasn't] enough battery in there for the night," which either made them wear the SomnoRing an extra night or delay their sleep to wait until the SomnoRing battery was charged. As for fit, the wearable sleep device was either too snug for some participants as it got "a bit uncomfortable by the end of the night" since "it leaves you with a kind of feeling like when you wear really tight socks." A few others were concerned that the wearable sleep device would not securely stay on their finger.	participants' housing status, access to insurance, and expense of devices were discussed as more direct barriers to using the SomnoRing in everyday life.<br>However, despite encouragement from loved ones, participants commented on costs and insurance status inhibiting their prioritization of sleep care and ability to afford technologies like the SomnoRing.				Somnology provided funding for this study and waived the right to see data as well as the right to analyze and interpret results. MS and KHN received contract funding from Somnology. SL received contract funding from AppliedVR, InquisitHealth, and Somnology. MSL is a part-owner (shareholder) of Somnology and is employed by Redwood Pulmonary Medical Associates. EB is employed by Redwood Pulmonary Medical Associates. US receives contract funding from AppliedVR, InquisitHealth, and Somnology; is supported by a gift from The Doctors Company Foundation; serves as a scientific or expert advisor for nonprofit organizations HealthTech 4 Medicaid and HopeLab (no compensation); is a member of the American Medical Association's Equity and Innovation Advisory Group (for which she receives honoraria); and previously served as a clinical advisor for Omada Health and as an advisory board member for Doximity (for which she received honoraria). CRL receives contract funding from AppliedVR, InquisitHealth, and Somnology.
Patients' Information Needs Related to a Monitoring Implant for Heart Failure: Co-designed Study Based on Affect Stories	2023	JMIR HUMAN FACTORS	Davat, A and Martin-Juchat, F	10.2196/38096	RealWorld4Clinic is a European consortium that is currently developing an implantable monitoring device for acute heart failure prevention.This study aimed to identify the main issues and information needs related to this new cardiac implant from the patients' perspective.	Qualitative	France	patient with heart failure	summarized in Table 1. Of the 26 participants, 16 (62%) were men, and 10 (38%) were women. The average age was 65 (SD 17; range 21-89) years. Most (21/26, 81%) of the patients had already been implanted with a medical device with remote monitoring capabilities, and some (12/18, 67%) were part of a remote follow-up program using connected objects. Most (21/26, 81%) of the patients were living with heart failure, and 12% (3/26) had experienced it before receiving a heart transplant. The (2/26, 8%) exceptions were patients with suspected cardiac disease who had contacted us after receiving the	a Monitoring Implant for Heart Failure				This confirms our first hypothesis: the determining factor in the acceptance or refusal of an implant is the trusting relationship with a health care team. Oudshoorn [25] has even suggested that patients do not really have a choice because these implants are the present standard of care.				To better understand patients' motives for rejecting implants, another theme should be explored: the "foreign body" or cyborg theme.Whether these results are easily transposable to monitoring implants is unclear. Implanting a monitoring implant under the skin is safer than implanting a pacemaker or a defibrillator, the leads for which can be a source of medical complications [33,34]. However, there is greater public awareness about pacemakers and defibrillators than about monitoring devices. Moreover, pacemakers and defibrillators actively contribute to the health of their wearer, whereas a cardiac monitor may seem excessive if it is presented only as a diagnostic tool		When their symptoms increase or if they do not use the devices for a couple of days, they immediately receive a call from a nurse who checks up on their situation daily. These calls are seen as proof of the existence and effectiveness of the monitoring program		N
Qualitative study of user perspectives and experiences of digital inhaler technology	2022	NPJ PRIMARY CARE RESPIRATORY MEDICINE	Adejumo, I and Patel, M and McKeever, TM and Shaw, DE and Bains, M	10.1038/s41533-022-00320-9	Electronic monitoring devices (EMDs) have been trialled in interventions to improve inhaled corticosteroid adherence and clinical outcomes. This study sought to understand the perceptions and experiences of EMD end-users.	Mixed	UK	athma patient	Non-interview participants Interview participants Control participants Intervention participants<br>Number 8 28 13 15<br>Median age (IQR) 50.4 (33.2, 57.0) 46.7 (33.5, 54.2) 41.9 (32.2, 49.5) 50.3 (34.9, 58.6)<br>Female n (%) 5 (63) 19 (68) 8 (62) 11 (73)<br>Caucasian n (%) 6 (75) 24 (86) 10 (77) 14 (93)<br>Percentage adherence, mean (SD)a 65.9 (32.2) 65.5 (32.6) 60.2 (34.4) 70.1 (31.4)<br>Percentage adherence, median (IQR)a 59.3 (49.3, 96.5) 81.0 (36.5, 91.5) 53.4 (36.3, 88.2) 83.4 (43.6, 96.3)	digital inhaler technology			Participants were generally aware that their inhaler use was being monitored and were broadly accepting of this.		Most participants found the devices bulky. This was generally seen as a negative characteristic, associated with decreased ease of use or increased social embarrassment.		Future applications of digital inhaler technology—potential improvements and uses<br>There was consensus that embedding EMD technology within inhalers would be superior to attached devices, although the financial and environmental costs of this were questioned.<br><br>When discussing the contexts for feedback of EMD data in routine care, most participants felt feedback should be coordinated by primary care given pre-existing relationships, access to health records, and ability to provide continuity of care.<br><br>Many participants accepted elements of remote feedback, but few wanted it to replace in-person interactions altogether. Most expected clinician feedback to be situated in the context of their routine asthma reviews.<br><br>Participants primarily anticipated that EMD data would provide their healthcare teams with detailed, accurate and objective information to inform better decision-making in the future. Most expressed a desire for joint responsibility to self-monitor and expected support in interpreting data in a way that would have meaning and utility.<br><br>Several participants felt that platforms integrating inhaler use data with environmental (e.g. pollen counts, pollution), physiological (e.g. heart		Overall, participants were open to having an EMD in the future, largely based on the potential to facilitate better communication with clinical teams, empower self-management, and reduce health system burdens.	Both control and intervention participants noted that the awareness of being monitored increased their awareness of how they used their inhalers, including implications for current asthma status.Access to objective inhaler use data gave a few participants more confidence to request support from their clinical teams.	There were concerns around depersonalisation, highlighting that a fully remote, automated system was unlikely to be acceptable as part of routine care.For a few, GPS data use was "invasive" and "irrelevant", highlighting concerns about privacy and autonomy.<br><br>Autonomy also appeared to be a concern when discussing automated responses to acute deterioration. Several participants communicated that they did not want to lose control of decision-making, even if acutely unwell. This need for control may have been why phone calls and in-app alerts were seen as more acceptable than paramedics responding in person to automated alerts.	I.A declares PhD studentship funding from an investigator-led grant from GlaxoSmithKline paid via institution related to this work. M.P., T.M.M., D.E.S. and M.B. have no conflicts of interest to declare related to the submitted work.
Tolerability of a comprehensive cardiorespiratory monitoring protocol in an epilepsy monitoring unit	2018	EPILEPSY & BEHAVIOR	Gehlbach, BK and Sainju, RK and Tadlock, DK and Dragon, DN and Granner, MA and Richerson, GB	10.1016/j.yebeh.2018.06.006	Recent reports of fatal or near-fatal events in epilepsy monitoring units (EMUs) and an increasing awareness of the effects of seizures on breathing have stimulated interest in cardiorespiratory monitoring for patients undergoing video-electroencephalography (EEG) recording. Patient and provider acceptance of these extra recording devices has not previously been studied and may represent a barrier to widespread adoption. We queried EMU subjects regarding their experiences with a monitoring protocol that included the continuous measurement of oral/nasal airflow, respiratory effort (chest and abdominal respiratory inductance plethysmography), oxygen saturation, and transcutaneous CO2.	Quantitative	USA	EMU patients	Variables Epilepsy (N = 71) Nonepileptic spells (N = 29) p value Adjusted p value<br>Age (year; median, lower, and upper quartiles) 39 (30, 50) 35 (23.5, 48) 0.1 0.8<br>Female sex—no. (%) 44 (62) 18 (62) 0.99 1.0<br>Prior video-EEG study—no. (%) 26 (37) 2 (7) 0.0026 0.02081<br>Prior sleep study—no. (%) 14 (20) 5 (17) 0.77 p = 1.0<br>Number of EMU days (median, lower, and upper quartiles) 4 (3, 6) 3 (2, 3) 0.00026 0.00208<br>Number of respiratory monitoring days in EMU (median, lower, and upper quartiles) 3 (2, 4) 2 (1, 3) 0.019 0.152<br>Seizure/spell captured during video-EEG study—no. (%) 53 (75) 21 (72) 0.818 1.0<br>Obstructive sleep apnea—no. (%) 10 (14) 5 (17) 0.689 1.0	epilepsy monitoring unit			Seventy-three percent of the participants reported being moderately to highly satisfied with the comprehensive monitoring protocol. Univariate analyses showed level of satisfaction to be unrelated to diagnosis of epilepsy, age, gender, duration of video-EEG monitoring, duration of respiratory monitoring, BMI, seizures or spells captured during the study, history of prior video-EEG, and history of prior polysomnography (all with p = NS).	Eighty-two percent of the participants reported moderate to strong agreement that advance knowledge of the monitoring would not have changed their decision to proceed with the video-EEG study, a rate that did not differ between patients with or without epilepsy (84.5% vs 72.4%, p = NS).	The percentage of subjects who reported no or mild discomfort for each component was 73.7% for EEG, 84.8% for ECG, 45.5% for nasal airflow, 71.7% for RIP, and 74.7% for tcCO2 monitoring. These rates were not different (p = NS, Table 2) between patients with and without epilepsy. When compared with EEG electrodes, a significantly smaller proportion of subjects reported no or mild discomfort with airflow monitoring (73.7% vs 45.5%, Bonferroni adjusted p = 0.004) while other components were comparable (p = NS). In univariate analyses, level of discomfort with airflow monitoring was independent of age, epilepsy diagnosis, duration of EMU stay, length of respiratory monitoring, gender, BMI, prior video-EEG, and prior sleep study (all with p value greater than the Bonferroni adjusted significance level of 0.00625). Two variables—history of prior video-EEG and duration of EMU stay—were significantly different between patients with or without epilepsy and were therefore selected for further analysis to determine their effect on patients' comfort or satisfaction with the monitoring protocol. These variables showed no significant associations with any outcome (data not shown).Sixty percent of subjects generally agreed (with variable strength of agreement) that comprehensive monitoring did not interfere with their sleep, and 63% agreed that it did not interfere with daily activity. These rates were not significantly different between subjects with or without epilepsy (data not shown).Subjects occasionally commented on aspects of the protocol that bothered them. Examples included "equipment frequently beeping", "nasal airflow hard to keep in place", "belts needed frequent checks", and "too many wires, often							This study was supported by the following:<br>1)<br>the National Institute of Neurological Disorders and Stroke program grant U01NS090414,<br><br>2)<br>the National Institutes of Health CTSA program grant U54TR001356I,<br><br>3)<br>Citizens United for Research in Epilepsy (CURE), and<br><br>4)<br>the Beth L. Tross Epilepsy Research Fund.<br><br>The SenTec Digital Monitoring System was provided to the investigators by SenTec AG, Therwil, Switzerland. The company was not involved in the design, execution, analysis, or reporting of this study. The authors report no other conflicts of interest.
Sensor acceptance model - Measuring patient acceptance of wearable sensors	2008	METHODS OF INFORMATION IN MEDICINE	Fensli, R and Pedersen, PE and Gundersen, T and Hejlesen, O	10.3414/ME9106	This project focuses on how patients respond to wearable biomedical sensors, since patient acceptance of this type of monitoring technology is essential for enhancing the quality of the data being measured.	Quantitative	Unspecified	patient		wearable sensors			forthe36patientsrangedfrom2.67to10.0,<br>withameanvalueof7.8(SD=1.7).Comparisonofthetwogroups(wirelessandreferencegroup)didnotshowanysignificantdifferenceattheaggregatelevel,however,the<br>subscaleHygienicAspectsshowedasignificanthigherscoreforthewirelesssensor									Ass.Prof.RuneFensliisashareholderinthecompany<br>WirelessPatientRecordingMedicalAS,<br>whodevelopsandproduceswirelessECG-<br>sensors,andisassistingthecompanyinresearchprojects.Otherwisethere<br>arenoconflictsofinterests.

Digital Inequalities in the Use of Self-Tracking Diet and Fitness Apps: Interview Study on the Influence of Social, Economic, and Cultural Factors	2018	JMIR MHEALTH AND UHEALTH	Regnier, F and Chauvel, L	10.2196/mhealth.9189	We aimed to determine under which social, economic, and cultural conditions individuals in France were more likely to be actively invested in the use of self-tracking diet and fitness apps for better health behaviors.	Qualitative	France	General public	The sample included 60 women, which was attributable to the high proportion of women enrolled in Weight Watchers and using MyFitnessPal. The majority of sport app users were men. Interviewees were aged between 23 and 70 years, with a mean age of 43 years. Most of them were employed in intermediate professions, such as technicians or nurses (31/79, 39%), or were clerical workers (21/79, 27%). Interviewees also had diverse social profiles, in terms of occupational status, associated with their respective self-tracking device. Sport app users tended to belong to high-income milieus, users of MyFitnessPal to the intermediate categories, and Weight Watchers members to intermediate- to low-income milieus (Table 2). Among sport app users, we found that 8 participants used watches (Garmin, Suunto), 5 used mobile phone apps (Runkeeper, Runtastic), 2 used Fitbit wristbands, 6 used 2 devices (mostly a	Self-Tracking Diet and Fitness Apps						Quadrants of Figure 1 labeled "Resistant," "For self-improvement," and "For sharing".Interviewees used the apps for a diversity of uses, including measurement, tracking, quantification, and participation in digital communities.		A digital divide was highlighted, comprising a major social gap. Social conditions for appropriation of self-tracking devices included sociodemographic factors, life course stages, and cross-cutting factors of heterogeneity.<br><br>Conclusions<br>Individuals from affluent or intermediate social milieus were most likely to use the apps and to participate in the associated online social networks. These interviewees also demonstrated a preventive approach to a healthy lifestyle. Individuals from lower milieus were more reluctant to use digital devices relating to diet and physical activity or to participate in self-quantification. The results of the study have major implications for public health: the digital self-quantification device is intrinsically less important than the way the individual uses it, in terms of adoption of successful health behaviors.				N
Perspectives on wellness self-monitoring tools for older adults	2013	INTERNATIONAL JOURNAL OF MEDICAL INFORMATICS	Huh, J and Le, T and Reeder, B and Thompson, HJ and Demiris, G	10.1016/j.ijmedinf.2013.08.009	Our purpose was to understand different stakeholder perceptions about the use of self-monitoring tools, specifically in the area of older adults' personal wellness.	Qualitative	USA	older adults and caregiver	Inclusion criteria for older adults were to be a resident of the independent retirement community and be aged 62 years or older.	wellness self-monitoring tools			Older adults generally struggled in making sense of the concept of self-monitoring, but presented insights in various social dynamics around how family members can become stakeholders of older adults' self-monitoring tools. Older adults showed passiveness in controlling their own wellness information but showed favorable response over sharing wellness information with their health care providers, which they saw as a potential way of increased patient-doctor communication. At the same time, sharing wellness information had some dependencies.									N
Antecedents for Older Adults' Intention to Use Smart Health Wearable Devices-Technology Anxiety as a Moderator	2022	BEHAVIORAL SCIENCES	Jeng, MY and Pai, FY and Yeh, TM	10.3390/bs12040114	Taking "technological anxiety" as the moderating variable to develop the questionnaire scale, the quantitative research with structural equation model is applied to discuss the older adults' intention to use smart health wearable devices.	Quantitative	Taiwan	Older adults	The effective questionnaire samples contain responses from 74 males (44.6%) and 92 females (54.4%), where the age group of 60–69 appears the most at 119 (71.6%), followed by 39 people aged 70–79 (23.5%). Eighty people show having the education of university or above (48.2%), followed by 44 with senior high (vocational) school (26.5%). Fifty-seven people are in the service industry (34.3%), followed by 50 people who are retired (30.1%). Seventy people show their monthly disposable income to be 20,000–40,000 NT dollars (42.2%), followed by 44 with 40,001–60,000 NT dollars (26.5%). Up to 102 people live with family members (61.4%), followed by 55 living with a spouse (33.1%). Ninety-nine people have experience in using smart health wearable devices (59.6%). An amount of 123 people show the volunteer willingness to use smart health wearable devices (74.1%).	Smart Health Wearable Devices-Technology			. The research results reveal that users with higher technology readiness, and older adult users with higher technological interactivity,		present a higher perceived ease of use and perceived usefulness.		Technological anxiety would affect users' attitude and further influence the intention to use.					N
Older adults' perspectives of smart home technology: Are we developing the technology that older people want?	2021	INTERNATIONAL JOURNAL OF HUMAN-COMPUTER STUDIES	Ghorayeb, A and Comber, R and Gooberman-Hill, R	10.1016/j.ijhcs.2020.102571	This paper explores older people's views of smart home monitoring technology and compares these between people with direct experience and those without.	Qualitative	UK	Older adults	We recruited a total of thirteen older people over the age of 65 (range 65 – 89 years of age). Five out of the thirteen participants self-identified as male and two identified as belonging to minority ethnic groups. This demographic approximates the population in the city, with 15% minority groups and 54% female in the most recent data (Council, 2020). All participants were living independently in their own homes. At the time of data collection, eight participants were living alone and five lived with partners. Both groups were broadly equivalent for age, gender education and other technology experience. Seven participants held postgraduate degrees, three of whom obtained these degrees when aged over 40 years. Another two participants held a bachelor's degree or equivalent. Four participants had school education. Beyond experience of SPHERE technology, most participants had some experience with smart	smart home technology			Among participants who had already tried the smart home monitoring technology, acceptance increased over time and with use. Non smart home participants focused on the extent to which this technology might increase household's vulnerability and they considered the technology somewhat intrusive and noticeable. It appeared that the more positive views of participants who had direct experience of smart homes related to the degree of trust between them and the researchers who installed and maintained the smart home system. Both groups of participants shared views about the technical feasibility, affordability, impact on relationships, and about the engagement and competencies of those who would view the monitoring data				They suggested that the technology would be more acceptable if it was possible to customize functionality and features.				They expressed fewer concerns than non smart homes participants regarding privacy, trust, usability, and more concerns about utility.	N
Patient Perspectives on the Ambulatory Glucose Profile Report for Type 1 Diabetes Management in Adults: A National Online Survey	2023	CANADIAN JOURNAL OF DIABETES	Mackett, K and Gerstein, H and Santesso, N	10.1016/j.jcjd.2023.01.001	Continuous and flash glucose monitoring devices produce data reports (e.g. ambulatory glucose profile [AGP]) that can be used by people with diabetes and health-care providers (HCPs). Clinical benefits of these reports have been published, but the patient perspective is underreported.We conducted an online survey for adults with type 1 diabetes (T1D) using continuous/flash glucose monitoring to understand their use and attitudes toward the AGP report. Barriers and facilitators related to digital health technology were explored.	Quantitative	Canada	Diabetes patients	A total of 402 respondents consented to participate in the survey; however, 29 did not meet the eligibility criteria and another 82 were excluded, as they did not answer any questions after the eligibility questions or had duplicate IP addresses and time stamps. Thus, 291 eligible respondents completed the survey and were included in the analyses. There were some missing data, as response rates to individual questions ranged from 66% to 94%. Nearly 75% of respondents were female, 52% were of North American ethnic or cultural origin, 63% were <40 years of age, and 65% had been living with T1D for >15 years. Almost 80% of respondents were educated with trade school, college, or university degrees and were employed (Table 1).	Ambulatory Glucose Profile Report for Type 1 Diabetes Management in Adults					Most respondents answered that they understood most/all parts of their AGP report (83%)	Most respondents answered that they understood most/all parts of their AGP report (83%), and close to all (92%) felt motivated about managing their diabetes (Table 2). Greater understanding was associated with feeling motivated to keep up with their diabetes management (OR=2.61; 95% CI, 1.45 to 4.71) and with reporting that their diabetes is well-managed (OR=2.37; 95% CI, 1.35 to 4.14) (Supplementary Table 2). Of the 217 respondents somewhat or very motivated to keep up with their diabetes management, 134 looked at their AGP on their own and with a health-care provider, 36 only looked at their AGP report with a health-care provider, and 28 did not look at their AGP report. Only 51% of the respondents were very/completely satisfied with operating the apps and websites used to generate the AGP report (Table 2). We did not find that older age was associated with less satisfaction; in fact, respondents <30 years of age may have been less likely to be satisfied compared with respondents aged ≥50 (OR=0.54; 95% CI, 0.13 to 2.18) (Supplementary Table 2). However, there were some comments about	Despite the majority of participants indicating that the AGP report is useful, accurate, and worthy of recommendation to their peers, few respondents were completely/very satisfied with the clarity or layout of the report, and less than half thought it was easy to interpret (Table 2). Some constructive feedback included defining the statistical terms within the AGP report and how the scores are calculated.<br><br>Respondents expressed improvement in diabetes management when using the report (Table 2), such as identifying patterns unbeknownst to them and lowering of A1C. Respondents also appreciated the ability to visualize their glucose data, which enabled them to easily pinpoint times of dysglycemia and then set personal goals	Security of personal health information does not appear to be a barrier to the use of this technology, as 60% of respondents were reportedly satisfied (Supplementary Table 1). Less than 30% of respondents were completely/very satisfied with the insurance coverage (Table 2). Multiple respondents expressed in the open-ended comments difficulties and frustration with out-of-pocket expenses due to lack of coverage, and the fear of a worsening condition due to loss of insurance coverage as they age. Many expressed that having coverage for a device would keep people out of the hospital and save unnecessary visits to the emergency room.				N
Where horizontal and vertical surveillances meet: Sense-making of US COVID-19 contact-tracing apps during a health crisis	2022	MOBILE MEDIA & COMMUNICATION	Baik, J and Jang, E	10.1177/20501579221078674	Analyzing user reviews of seven US digital contact-tracing apps for COVID-19, this article unpacks how the new form of surveillance technology is understood and experienced by individuals during a global health crisis.	Qualitative	USA	General public	user reviews	COVID-19 contact-tracing apps				[illegible]<br>users expressed frustration about the ineffectiveness of vertical surveillance through contact-tracing apps due to technical malfunctions. The informativeness and accuracy of contact tracing mattered to users.<br><br>Trust and distrust<br>Another factor that affects individuals' perceptions on vertical surveillance is the level of trust they have in institutional entities.<br><br>As such, users often conceived their participation not as a burden but rather as a matter of course. Users' acceptance and promotion of the surveillance technology was in consideration of a broader community around the app users, such as their beloved ones, neighbors, the vulnerable, and even the human species as a whole.						[illegible]<br>Contact-tracing apps also mediate many-to-many interactions among citizens, helping them cope with a health crisis through collaborating with and concern for community members. As reflected in these reviews, contact-tracing apps were empowering their users by mitigating anxiety, letting them feel safer, and giving a sense of hope that their life will soon be normalized. The users not only perceived contact tracing as a tool to control their body's potential exposures to the virus, but also envisioned it as a facilitator that helps go back to normal. Like other self-tracking technologies, contact-tracing apps appear to foster the restoration of agency and autonomy over individual bodies in this unsettling health crisis (Sharon & Zandbergen, 2017), and the identity of each individual (e.g., health condition, age, occupation) plays a role in their perception of the surveillance technology.	As such, the existence of contact-tracing apps in some states and lack thereof in other states might have been inviting senses of both solidarity and alienation in the US, as those who live in the states with contact-tracing apps encourage fellow residents to adopt them while those who live in the states without any apps cannot reap perceived benefits of the surveillance technology. Certain technical and user experience (UX) limitations also made users concerned about their alienating effects for some community members (e.g., vulnerable populations or the elderly). Users pointed out the lack of awareness and the limited accessibility of the apps.	UNDECLARED
Health technology identities and self. Patients' appropriation of an assistive device for self-management of chronic illness	2020	SOCIOLOGY OF HEALTH & ILLNESS	Wienroth, M and Thomsen, LLH and Hostgaard, AM	10.1111/1467-9566.13079	In recent years, assistive technologies have gained acceptance as tools for supporting chronically ill patients in achieving improvements in physical activity. However, various healthcare and sociological studies show contradicting results regarding the physical and social impact of using such devices. This paper explores real-time user appropriation of an assistive monitoring/tracking device, the pedometer, in a healthcare intervention, with a particular focus on the technology identities users attribute to the pedometer.	Qualitative	Denmark	Patients with chronic illness	Focus-group interviewsNo. of participantsWomen/men1. Patients: before handing out the pedometerGroup 12/2Group 24/0Group 32/0In total8/22. Patients: when handing in the pedometerGroup 12/1Group 23/0Group 31/2In total6/3	Pan assistive device for self-management of chronic illness			The analysis of respondents'accounts shows that monitoring devices become partof users'complex socio-technical ensembles in which the use of the device and itstracking of activity is constantly negotiated through experimentation with type andfrequency of use; interpretation of knowledge and experience gained via thedevice; and negotiation of expectations, wellbeing, and the value of quantifiedknowledge for the management of chronic illness									UNDECLARED
Anticipated barriers and facilitators for implementing smart inhalers in asthma medication adherence management	2023	NPJ PRIMARY CARE RESPIRATORY MEDICINE	van de Hei, SJ and Stoker, N and Flokstra-de Blok, BMJ and Poot, CC and Meijer, E and Postma, MJ and Chavannes, NH and Kocks, JWH and van Boven, JFM	10.1038/s41533-023-00343-w	Smart inhalers are electronic monitoring devices which are promising in increasing medication adherence and maintaining asthma control. A multi-stakeholder capacity and needs assessment is recommended prior to implementation in healthcare systems. This study aimed to explore perceptions of stakeholders and to identify anticipated facilitators and barriers associated with the implementation of smart digital inhalers in the Dutch healthcare system.	Qualitative	Netherlands	athma patient	mean age<br><br>34.7 (13.3) 9F	smart inhalers							Suggestions that were made are sharing information only during consultations by showing the app to the HCP, or to offer the possibility to enable data sharing only when patients experience symptoms (Q24).	User-friendliness<br>All participant groups cited "user-friendliness" as a facilitator for the implementation of smart inhalers (Q4). Components contributing to user-friendliness that were reported included: (i) inhaling should not be more complicated with an attached or integrated smart inhaler (e.g. more inspiratory flow needed, harder to activate the device) compared to a usual inhaler, (ii) push messages should not have a pedantic character and must be limited in frequency, (iii) installing and using the app and portal should be intuitive and should not require much effort, (iv) prescribing smart inhalers should be easy, and (v) data should be collected passively, with as little effort as possible by patients and HCPs (i.e. no technical issues, no manual data uploads). In addition, the possibility to personalise the app was cited as a facilitator by patients, HCPs and developers (e.g. possibility to change timing and frequency of reminders), as it could avoid annoyance and increase alignment with treatment goals (Q5). Developers considered it necessary to involve all stakeholders including the end-user in the development process, to improve the usability (Q6).<br><br>Compatibility<br>A barrier to implementation cited by all participants groups was the lack of compatibility on different levels. First, patients can use more than one inhaler and patients who switch inhalers should still be able to use a smart inhaler. This means that smart inhaler devices need to be		Perceived benefits of smart inhaler use by patients included improved medication adherence and improved inhalation technique.<br>However, patients also indicated that they do not always know whether they are taking the correct dose and whether their inhalation technique is correct, and that sharing this data could help them to discuss this with their HCP and to stay controlled or become better controlled (Q25). HCPs emphasised that data sharing could be used to start a discussion on medication use with a patient.	Theme 5: Data safety and ownership<br>This theme addressed the ethical question who owns the data, as well as data sharing between HCPs and patients, and the privacy and security of smart inhaler data. Knowing who has access to data and the guarantee that data is properly secured was found to be important to patients. Additionally, patients highlighted that they perceive a smart inhaler to be unsafe with regard to data privacy when it is sold online, because there may be commercial interest (Q20). Data sharing between patients and HCPs was considered as a barrier on the one hand and as a facilitator on the other. A reason to not share data with the HCP, is that patients consider it personal information. This was corroborated by HCPs, who mentioned that sharing data is something patients should decide for themselves (Q22). Furthermore, patients described that they doubt the feasibility of processing such a large amount of data by	received consultancy fees, speaking fees and/or research grants from Aardex, AstraZeneca, Chiesi, European Commission COST Action 19132 (ENABLE), GSK, Menarini, Novartis, Pfizer, Teva, Trudell Medical, and Vertex, all unrelated to this study. J.W.H.K. reports grants, personal fees and non-financial support from AstraZeneca, grants, personal fees, and non-financial support from Boehringer Ingelheim, grants and personal fees from Chiesi Pharmaceuticals, grants, personal fees and non-financial support from GSK, non-financial support from Mundi Pharma, grants and personal fees from TEVA, personal fees from MSD, personal fees from COVIS Pharma, grants from Valneva, outside the submitted work; and Janwillem Kocks holds <5% shares of Lothar Medtec GmbH and 72.5% of shares in the General Practitioners Research Institute. B.M.J.F.-d.B. and N.S. were employed by General Practitioners Research Institute (GPRI) at the time of the study. In the past 3 years (2019-2021), GPRI conducted investigator- and sponsor-initiated research funded by non-commercial organisations, academic institutes, and pharmaceutical companies
A qualitative analysis of the effectiveness of telehealthcare devices (i) are they meeting the needs of end-users?	2017	BMC HEALTH SERVICES RESEARCH	Campling, NC and Pitts, DG and Knight, PV and Aspinall, R	10.1186/s12913-017-2408-8	There are many telehealthcare devices currently available ranging from personal alarms, automated pill dispensers and fall detectors through to monitoring devices for blood sugar, blood pressure and heart rate. Many devices remain unused once acquired or shortly after a period of initial use. The study used a qualitative design involving focus groups and interviews. End users' opinions of telehealthcare devices were examined through focus groups along with the views of market experts and key supply chain players through telephone interviews to ascertain their views on the devices.	Qualitative	UK	patient	The group was balanced for gender, with eight participants being male and seven female. Their ages ranged between 65 and 84 years of age, four being between 65 and 69, three between 70 and 74, five between 75 and 79 and three from 80 to 84. In terms of health problems, four stated that they had mobility issues (one used a mobility scooter). One participant was using a pendant alarm (supplied via social services and operated via the local council). The majority (with a sole exception) owned a mobile phone and all were using computers/tablets and email. A number of the group also had access to blood pressure monitors at home, which had been privately purchased. The group in Oxfordshire also saw high attendance with 12 of the 13 participants originally recruited attending. Eleven of the group were female and one male. The age range was wider than with the Bedfordshire group, from 60 to over 85 years of age. Two were aged between 60 and 64, two	personal alarms, automated pill dispensers and fall detectors through to monitoring devices for blood sugar, blood pressure and heart rate.							Aesthetic appearance<br>For some, device aesthetics were clearly important particularly with wearable devices<br>For the experts the key was well-designed technologies, which people wish to use, and prevent stigmatisation.<br><br>Practicality and ease of use<br>All the study participants identified the need for good design of telehealthcare products. Good design elements were viewed as practicality, ease of use, efficiency, confidence inspiring and flexibility to adapt to changeable needs and an illness trajectory.<br>The lack of focus on end-user needs<br>The expert participants in the study identified the essential requirement of focussing on end-user needs.	Another participant spoke of these barriers as "perceptions around people's ability to use technology" (Interviewee 014). This was associated with the need to provide "confidence to patients that the use of these technologies doesn't mean that it's taking away… contact when they need it, and care when they need it from their clinician, or carer, or whoever it might be" (Inteviewee 014).				N
Perceptions of seniors with heart failure regarding autonomous zero-effort monitoring of physiological parameters in the smart-home environment	2017	HEART & LUNG	Grace, SL and Taherzadeh, G and Chang, ISJ and Boger, J and Arcelus, A and Mak, S and Chessex, C and Mihailidis, A	10.1016/j.hrtlng.2017.04.007	To understand the perceptions of seniors with heart failure (HF) regarding smart-home systems to monitor their physiological parameters.	Qualitative	Canada	seniors with heart failure	Characteristic n (%)/mean ± SD<br>Sociodemographic<br>Age 74.96 ± 6.69<br>Sex (% Male) 19 (73.1%)	seniors with heart failure regarding autonomous zero-effort monitoring of physiological parameters in the smart-home environment			Participants voiced strong opinions regarding whether they liked or disliked technology. Four sub-themes arose. First, participants reported difficulty in operating general technology they currently have in their home. The second sub-theme related to feelings of discomfort with, or the design of, technology at home. The third sub-theme related to perceptions regarding health monitoring technology specifically in the home. The final sub-theme related to previous exposure to remote health monitoring.				Theme 5: Preferences for Autonomous Monitoring<br>The final theme encompasses the participants' preferences for autonomous monitoring, in five areas. The first sub-theme was participants' preferred parameters monitored during the trial. These were most often blood pressure and pulse. The second sub-theme was preferred rooms to place the monitoring devices. Most participants found comfort in having the devices placed in their bedroom to be monitored while asleep. The third sub-theme was preferred objects in the home in which to embed monitoring technology.<br>The fourth sub-theme was the noted comfort in having the sensor in the home. The final sub-theme addressed additional parameters which HF patients would like monitored. This sub-theme included an array of responses.	Theme 4: lack of perceived need for continuous health monitoring<br>Some participants expressed strong views that there was no need for continuous health monitoring.		[illegible] monitoring<br>Participants reported six main benefits they could derive from autonomous health monitoring. The first benefit was health status information availability. Participants perceived their health care providers and family members would benefit from being able to access information about their well-being remotely. The second benefit was independence. Participants desired to stay in their own home for as long as possible. The third benefit was re-assurance or security.<br>The fourth perceived benefit was that autonomous monitoring could potentially be life-saving. The fifth benefit was risk mitigation, as one participant stated:The last perceived benefit associated with autonomous monitoring was unobtrusiveness.	Theme 3: perceived disadvantages of autonomous monitoring<br>The participants communicated six disadvantages associated with autonomous monitoring. The first sub-theme was affordability. The second sub-theme was related to data use. Equipment malfunction concerns were the next sub-theme identified. The fourth disadvantage was fear of technology obsolescence. The fifth sub-theme was multi-user concerns. Participants raised questions around concurrent use of the autonomous monitoring devices by different members of the household. The final sub-theme was participants' concern that information about their health status could be troubling or stressful.	N

Adoption of Telehealth: Remote Biometric Monitoring Among Peritoneal Dialysis Patients in the United States	2017	PERITONEAL DIALYSIS INTERNATIONAL	Lew, SQ and Sikka, N and Thompson, C and Cherian, T and Magnus, M	10.3747/pdi.2016.00272	We examined participant uptake and utilization of remote monitoring devices, and the relationship between remote biometric monitoring (RBM) of weight (Wt) and blood pressure (BP) with self-monitoring requirements.	Quantitative	USA	Peritoneal Dialysis Patients	Demographics N %<br>Age (years)<br>18–55 125 46.5<br>>55 144 53.5<br>Age [median (IQR)] 56 (43.6–64.3)<br>Gender (n=267)<br>Male 152 56.5<br>Female 115 42.8<br>Race<br>Black 102 3 7. 9<br>Non-Black 167 62.1<br>Primary insurance<br>Medicare 186 69.1<br>Education (n=186)<br>Less than high school equivalency 15 8.1<br>High school degree or equivalency 37 19.9<br>College or beyond 134 72.0<br>Residential area (n=187)<br>City 129 68.9<br>Suburban 40 21.4<br>Rural 18 9.6<br>Duration on dialysis prior to study entry,	Remote Biometric Monitoring among Peritoneal Dialysis			This study demonstrated RBM was acceptable to PD patients and resulted in improvements in frequency, accuracy, and communication of measurements to medical personnel relative to self-report. We did not identify differential uptake with regard to demographic characteristics, although frequency of use differed modestly by race.					Some limitations to RBM include the cost of equipment and services, additional work for healthcare providers, especially nurses who review daily results and address breached readings, and patient intrusion with frequent calls regarding breached readings.		Remote biometric monitoring allows for increased communication between patient and PD staff to improve patient adherence and perhaps outcomes.		This project was supported by a Center for Medicare and Medicaid Innovation grant #1C1CMS331004. The funding period started July 1, 2012, and ended on June 30, 2015. The contents of this publication are solely the responsibility of the authors and do not necessarily represent the official views of the US Department of Health and Human Services or any of its agencies. The research presented here was conducted by the awardee. These findings may or may not be consistent with or confirmed by the independent evaluation contractor.
Maternal satisfaction and acceptability of foetal electrocardiographic (STAN (R)) monitoring system	2011	ARCHIVES OF GYNECOLOGY AND OBSTETRICS	Parisaei, M and Harrington, KF and Erskine, KJ	10.1007/s00404-009-1268-9	To assess the acceptability of the foetal electrocardiographic (STAN(A (R))) monitoring system by patients at a London Hospital, before its introduction into routine clinical care.	Quantitative	UK	patients in labour	6.3); 40% of the study population were African, 30% white, 10% Asian and the remaining 20% were Caribbean, Black English, Turkish, Chinese, Somalian and mixed white and black. Eighty-three percent (CI 75, 89) of the patients spoke fluent English, and those who did not had an advocate in their language of choice available to them.<br><br>Seventy-five percent of women were nulliparous. Seventy-eight percent of women (CI 70, 85) had been deemed to be low risk at their antenatal booking.	foetal electrocardiographic (STAN®) monitoring system	The women were all asked if they understood how the STAN® system monitored their baby's well being in labour. Communicating technical medical terms to a patient in a language they understand is paramount in offering excellent clinical care. Ninety-five percent (CI 87, 99) of the mothers felt they understood the physiological basis behind STAN®.		Acceptability<br>Women were asked if they thought the STAN® system is an acceptable way of monitoring their baby in labour. After delivering and having been monitored with STAN® 95% (CI 87, 99) of women felt that it was an acceptable way of monitoring their babies in labour.				Recommendations<br>When asked if they would recommend the STAN® system to friends who are going to be mothers, 89% (CI 80, 95) of women would recommend the system to their friends who were pregnant. However, the majority would only do so if they were high risk and needed to be continuously monitored.			Reassurance<br>Women were asked if they felt reassured by having the STAN® system as well as the CTG monitor in labour. Ninety-six percent (CI 89, 99) of the women felt reassured by having a combination of FECG as well as CTG to monitor their baby's well being in labour.		N
Patient experience and healthcare utilization for a COVID-19 telemedicine home monitoring program offered in English and Spanish	2022	PLOS ONE	Vartanian, KB and Holtorf, M and Cox, EJ and Diaz, G and Khurana, H and Schlegel, S and Raganit, C and Ong, B and Czartoski, T	10.1371/journal.pone.0270754	This study examines engagement, experience, and utilization patterns for English and Spanish-speaking patients engaged in the COVID-19 home monitoring program (HMP).	Quantitative	France	HMP participants	the HMP (Table 1). English was spoken by 75.5% of HMP participants and Spanish was spoken by 18.2%. Among the HMP participants, 51.7% identified as female, 45.9% identified as White, 33.7% identified as Hispanic/Latino, 4.9% identified as Black, and 4.7% identified as Asian. A little less than half had their qualifying COVID-19 encounter in the emergency department (47.5%), while 31.8% came from inpatient care, and 17.2% came from outpatient care. Thirty percent had commercial insurance coverage, 27.2% were on Medicare, and 25% were on Medicaid. The most common symptom at presentation was shortness of breath (38.2%) followed by cough (34.5%). About half of the HMP participants	a COVID-19 telemedicine home monitoring program					Patient engagement and satisfaction with HMP<br>Engagement with the interface, defined as the average number of completed responses to the three times daily text prompts, was high (87.2 ± 23.3%) and was similar between Spanish- and English-speaking participants (77.6 ± 22.4 vs. 80.1 ± 20.9%) (Table 2). The vast majority of respondents indicated that devices were easy to use (97.7%, Table 3), and they felt safe recovering at home with the HMP (96.9%). The high percentage reporting ease of use and feeling safe recovering was true for English and Spanish-speakers. Overall, 79.1% of participants indicated 9–10 on the likelihood that they would recommend the HMP and the overall net promoter score was 71.5. Again, this high level of satisfaction was observed among both English and Spanish speakers (NPS = 67.4 and 77.5, respectively).					Care utilization<br>Overall, COVID-19 positive participants in the HMP were more likely to have an outpatient encounter compared to non-participants. For Spanish-speaking COVID-19 positive participants, the likelihood of both primary care and outpatient encounters was higher at 30 days compared to COVID-19 positive Spanish-speaking non-participants. For COVID-19 positive English-speaking participants, the likelihood of outpatient encounters was higher at both 7 days and 30 days, and the likelihood of primary care encounters were higher at 30 days compared to COVID-19 positive English-speaking non-participants (Table 4). COVID-19 positive participants in HMP also were more likely to have an ED visit in the first 7 and 30 days following their COVID-19 diagnoses, and this was also true among COVID-19 positive English and Spanish-speaking participants.		N
On the body of the consumer: performance-seeking with wearables and health and fitness apps	2019	SOCIOLOGY OF HEALTH & ILLNESS	Hardey, M	10.1111/1467-9566.12879	Contributing to critical digital health research and the sociology of health consumption, this study investigates the phenomenon of self-tracking and interpretation of consumer data via wearable technology and mobile fitness software applications (apps).	Qualitative	UK	General public	Those responding within two weeks were recruited, yielding 11 participants aged 19–67 (six women, five men). One was a law student; the others held various professional roles, including a semi-retired consultant. Household income ranged between approximately £15,500 and £80,000, averaging £50,000. Every participant used at least two wearable devices, with one (a personal trainer and occupational therapist) using 11 at any one time. Pseudonyms have been used.	wearables and health and fitness apps						These consumers are driven by a profound motivation to visualise and embody a long-term state of fitness.					Participants were also hyper-aware of advertising and promotional methods used to engage consumers. The findings raise concerns about the validity of personal fitness data, and how its collection promises improved personal health while visually promoting sought-after fit bodies.	N
Wearable Activity Trackers: A Structural Investigation into Acceptance and Goal Achievements of Generation Z	2021	AMERICAN JOURNAL OF HEALTH EDUCATION	Kamble, A and Desai, S and Abhang, N	10.1080/19325037.2021.1955229	The study investigates the acceptance of wearable activity trackers (WAT) by Generation Z users and draws upon achievement goal theory by theorizing how and why consumers use WAT.	Quantitative	India	generation Z	As the study intended to study specifically generation Z, the participants were Indian citizens studying in a higher education institution. Respondents signed a disclosure statement informing the participants of their voluntary participation. Researchers checked the duplication of data and reviewed missing data.<br><br>The percentage was of male respondents was 62.04% belonging to Gen Z. The age group considered for Gen Z was from 18 to 25 years, with 59% of the respondents undertaking an undergraduate/bachelor degree. Considering the research being carried out in early 2020, the youngest WAT user was 18 years old, born in 2002, and the most aged WAT user was born in the year 1995, thus concurring the starting age of Gen Z consumers.Citation10,Citation11 Out of all the respondents, 27.16% were 20 years old, followed by 21.60% of 22	Wearable Activity Trackers				The slope test results indicated that the impact of self-monitoring on intention to use by Gen Z gets stronger as the value of the mastery goal orientation rises.	The SEM results indicated that the structural model had satisfactory levels of fit indicesCitation81(p642) (see Table 2): χ2/df = 2.651, CFI = 0.955, GFI = 0.817, NFI = 0.916, IFI = 0.941, TLI = 0.935, PCFI = 0.832, PGFI = 0.688, PNFI = 0.805, AGFI = 0.931 and RMSEA = 0.064.<br><br>Starting with H1, perceived ease of use to using wearable activity trackers showed a positive relationship with attitude (H1, β = .160, p < .005) and significantly predicted it. For H2, wearable activity trackers' perceived usefulness showed a positive relationship and significantly predicted the attitude toward WAT use (H2, β = .352, p < .005). Furthermore, H3 and H6 were also supported by the results. For H3, the attitude toward WAT showed a significant positive relationship with the intentions to use WAT (H3, β = .227, p < .005). For H6 subjective norm to IU (H6, β = .109, p < .005), thus supporting the hypothesis. Furthermore, H4 was supported with PEOU (H4, β = .208, p < .005), subjective norm to PU was not supported (H5, β = .122, p > .005). Therefore, based on this model, 45% of the variance in attitude toward wearable activity tracker was explained, and 38% of intentions to use WAT variance. (Figure 2 and Table 5)	For hypotheses testing concerning motivational affordances and goal orientation, first, the motivational affordances on the intentions to use WAT were analyzed without the interactions of the performance and mastery goal orientations. All the hypotheses H7a H8a H9a were supported. Self-monitoring borne a positive relationship with intentions to use WAT (H7a, β = .481, p < .005). For H8a and H9a, rewards and social comparison showed a positive significant relationship with intentions to use wearable activity trackers; (H8a, β = .117, p < .005) and (H9a, β = .226, p < .005).<br><br>Next, we focused on the interactions of each motivational affordance with mastery and performance goal orientations in isolation. As interaction effect sizes are usually small, a conservative interpretation is 0.005 ("small"), 0.01 ("medium"), and 0.025 ("large").Citation82 To aid interpretation, we plotted the significant interactions (Figure 3).						N
Assembling the 'Fitbit subject': A Foucauldian-sociomaterialist examination of social class, gender and self-surveillance on Fitbit community message boards	2020	HEALTH	Esmonde, K and Jette, S	10.1177/1363459318800166	Using postings on Fitbit community message boards and the theoretical frames of Michel Foucault and sociomaterialist scholars, the goal of this article is to analyse the experiences of those who choose to self-track using a Fitbit and the constellation of barriers and facilitators (human and non-human) related to social class and gender that enable and constrain one's ability to use a Fitbit as intended.	Qualitative	USA, Canada, UK	General public	community message board titled 'Get Moving', which as of 12 May 2017 had almost 3000 discussion threads on physical activity and lifestyle. While demographic information of the posters on the message boards is difficult to ascertain as the majority of posters did not allude to it in their profile, it is worth noting that fitness tracker users tend to skew female, to be in the high-income bracket, and to be between the ages of 35–54 years (NPD Connected Intelligence, 2015). When users discussed their geographical location, it appears most were based in the United States, Canada or Britain.	Fitbit subject				Ultimately, one's ability to engage in the idealized use of the Fitbit in the minds of its users, or what we term the 'Fitbit subject assemblage', is structured by numerous material and social factors that must be taken into account when examining the mechanics of power in fitness tracking.								N
Community Engagement to Optimize the Use of Web-Based and Wearable Technology in a Cardiovascular Health and Needs Assessment Study: A Mixed Methods Approach	2016	JMIR MHEALTH AND UHEALTH	Yingling, LR and Brooks, AT and Wallen, GR and Peters-Lawrence, M and McClurkin, M and Cooper-McCann, R and Wiley, KL and Mitchell, V and Saygbe, JN and Johnson, TD and Curry, KE and Johnson, AA and Graham, AP and Graham, LA and Powell-Wiley, TM	10.2196/mhealth.4489	The objective of the study was to use qualitative and quantitative methods to evaluate the feasibility and acceptability of physical activity (PA)-monitoring wristbands and Web-based technology by predominantly African American, church-based populations in resource-limited Washington, D. C. neighborhoods.	Mixed	USA	patients with obesity-related cardiovascular disease	Variable<br>Sex, n (%)<br><br>Female 3/8 (37)<br><br>Male 5/8 (62)<br>Age, years<br><br>Mean (SD) 53.3 (12.2)<br><br>Range, years 28-70<br>Race, n (%)<br><br>Black/African American 8/8 (100)<br>Marital status, n (%)<br>Single 1/8 (12)<br>Married 7/8 (87)<br>Education, n (%)<br>Some college 3/8 (37)<br>College degree 2/8 (25)<br>Technical degree 1/8 (12)<br>Graduate/professional degree 2/8 (25)<br>Annual household income US (n=7), n (%)	Wearable Technology in a Cardiovascular Health and Needs Assessment Study					Regarding the feedback theme, multiple participants reported a desire for immediate, more accessible feedback complementary to the colored lights on the wristband. Additionally, participants tended to be disappointed with their personal vigorous activity minutes and requested more information on the types of activity that would register as vigorous.<br><br>Regarding the PA monitor design; participants raised concerns about physical discomfort and the monitor features. Some participants recommended a different clasp to prevent accidental detachment, and two participants experienced skin irritation where the wristband was secured. Several participants experienced difficulty differentiating between the colored lights, and a few participants found the wristband to be bulky in size.<br><br>An overarching theme was the ambiguity of the project goals outlined in the initial training. During training, participants were instructed not to modify their routine PA; however, the preset 30-minute vigorous activity goal of the wristband led some participants to modify their activities in pursuit of "show[ing] green" by reaching 30 minutes of vigorous activity in a single 24-hour period. In relation to the theme of recording PA, those participants who engaged in activities not measured by the wristband (eg, water sports, weight lifting, etc) preferred if the wristband would have captured all PA, regardless of its nature.<br><br>Participants also discussed issues about the centralized hub and its availability for synchronizing. There was one participant that reported that the hub did not work at		To address design concerns of the PA monitor, we ensured proper wristband attachment during training and warned participants of potential skin irritation issues. Participants were advised to wear the wristband loosely. To address potential misunderstandings of the colored-light system, we incorporated a detailed description of the wristband's colored lights during device training and on the "Helpful Hints" sheet, a frequently asked questions sheet created for at-home reference. With ambiguity over project goals as an overall participant concern, we redesigned the written and in-person education component. In training, we explicitly stated project goals verbally and in writing. Additionally, we developed two instructional training videos on device and hub usage that were used during device training and then made publically available after the event for participant reference. We also incorporated more hands-on, in-person training where participants could trial the website, test wristband lights, and upload wristband data.<br><br>Due to participant concerns about the hub, we improved our troubleshooting correspondence. We communicated weekly with the device company and the participants to identify and address hub issues, and we incorporated a report sheet next to the hub for streamlined reporting of issues. To address hub availability issues, we provided participants with a schedule of "hub hours" and the option to synchronize their wristbands at any of the participating churches. As per participant recommendation, we identified a point-person within each					N
Feasibility and Usability Aspects of Continuous Remote Monitoring of Health Status in Palliative Cancer Patients Using Wearables	2020	ONCOLOGY	Pavic, M and Klaas, V and Theile, G and Kraft, J and Troster, G and Guckenberger, M	10.1159/000501433	We aimed to evaluate feasibility as well as patients' acceptance of remote monitoring using wearables in palliative cancer patients.	Mixed	Swtherland	Palliative Cancer Patients	Between February 2017 and February 2018, a total of 68 patients fulfilled all the eligibility criteria. Of these, 31 patients consented to participate and were included. One patient died only a few days after discharge before any usage of wearables, thus no data are available for this patient. Nine of the 30 patients included in the analysis were female and 21 were male. The median age was 64 years (range 39–85). Table 1 provides more details about demographic data on participants and eligible patients not willing to participate. None of the patient characteristics differed significantly between study participants and eligible non-participants. Information on previous experience with electronic devices as well as reasons for rejection of study participation are reported in a not yet published paper, together with the results on the predictive ability of remote monitoring.	Continuous Remote Monitoring of Health Status in Palliative Cancer Patients Using Wearables					In general, 20 patients stated a positive overall rating concerning the study.Only 6 participants specified in any of the weekly interviews that it felt uncomfortable to wear the bracelet while sleeping. Another 3 patients reported that it was uncomfortable to wear the bracelet when the weather was too hot – the elastic belt led to annoying sweating. All these patients participated during the summer, commencing in June 2017. Six patients reported the elastic belt becoming loose within a few weeks. One of the patients then tried to wear it on their leg and rated this as very convenient. Another patient continued to have problems with an uncomfortable elastic belt even after exchanging to another one – he had a slightly larger upper arm and experienced that the band rolled up. Two patients would have favored wearing the bracelet on their wrist. Here are some examples: "The devices are quite eye-catching," "The biggest problem was that the battery of the smartphone switched to energy-saving mode due to fast consumption despite normal usage," and "It is cumbersome to operate even more devices." The last quote was from a patient who had to walk on crutches and reported difficulties in putting the bracelet on and wearing the smartphone on their body. One participant discussed the issue of data security with friends and relatives. Three patients declared spontaneously that they would like to see all their data collected by the wearables. Nine participants in total stated that they had to recharge the battery of the wearables (either smartphone or bracelet or both) very often. Four patients stated that having no display of the battery load of the bracelet was cumbersome and would therefore like to see the percentage of battery charge.Only 2 study participants (6.7%) had problems handling the wearables from the outset of the study. Both participants had no previous experience with smartphones or activity tracking watches. One of the 2 patients who struggled		(prolong battery life of wearables, display of battery life of the bracelet, and 2 patients commented that they would suggest a bracelet for the wrist). Three patients suggested that the data captured during the study should be discussed with the patients				A secondary issue was mainly that the step count or the heart rate were not displayed correctly (according to the judgement of participants), or not at all. Furthermore, the sensor occasionally lost connection to the smartphone and had to be reconnected "manually."	N
A Wireless Patient Monitoring System for Hospitalized Older Adults: Acceptability, Reliability and Accuracy Evaluation	2014	2014 IEEE-EMBS INTERNATIONAL CONFERENCE ON BIOMEDICAL AND HEALTH INFORMATICS (BHI)	Baig, MM and GholamHosseini, H and Connolly, MJ and Kashfi, G and IEEE		This paper aims to address some of the current issues and challenges facing wireless monitoring systems by adopting a robust three-way cross validation data collection approach. Our main focus is to evaluate the developed system in terms of user acceptability, reliability and accuracy.	Quantitative	New Zealand	Older adults	We evaluated the proposed system with 30 hospitalized older adults (17 males) with average age of 82.4 years.	A wireless patient monitoring system for hospitalized older adults					System achieved an overall mean score of seven; however there were some cases where the outcome was six or less. Comfort in using blood glucose monitor was recorded with a mean score of six. This low score of comfort was given by the participants due to the light pain caused by needle applied at the fingertip for sample collection. Ear temperature device was recorded seven because participants had some difficulties in operating the device. Using the sample size of 30, confidence coefficient of 1.96 (95% confidence interval) with 0.5 power, we calculated the mean (average), maximum, minimum, range, standard deviation and margin of error for each category (mobility, usability, comfort and acceptability) and for each device.							UNDECLARED
SWEET-HOME ICT technologies for the assessment of elderly subjects	2013	IRBM	Robert, P and Castelli, E and Chung, PC and Chiroux, T and Crispim, CF and Mallea, P and Bremond, F	10.1016/j.irbm.2013.01.015	Functional assessments are designed to ascertain a person's ability to perform activities of daily living (ADL) and provide valuable diagnostic as well as care-planning information. Currently, the gold standard for the assessment of functional ability involves clinical rating scales. However, scales are often limited in their ability to provide objective and sensitive information. In contrast, information and communication technologies (ICT) may overcome these limitations by capturing more fully the functional, as well as behavioral and cognitive disturbances associated with Alzheimer disease (AD). In this context, the ANR Tec San 2009 SWEET-HOME project aims at building an innovative framework for modeling ADL.This study tests the acceptance of	Quantitative	France and Taiwan	Older adults	Sixteen participants with mild-to-moderate AD and 10 age- and gender-matched Healthy Control (HC) participants from the Nice Memory Center were evaluated clinically, cognitively, and functionally (Using the Instrumental Activity of Daily Living Evaluation [IADL-E]).	SWEET-HOME ICT technologies for the assessment of elderly subjects			All the 64 French participants (22 healthy controls, 30 MCI, 12 AD) who accepted to be tested using the system indicated that the assessment has been perceived as pleasant (83%). The duration and level of difficulty of the assessment has been perceived as adapted respectively by 98% and 96% of the participants. Finally, 67% of the participants were ready to accept such assessment using multiple sensors in a room of their own home but only for a limited time during the day.									P. Robert, F. Bremond declare to be involvedin another European project (FP7)related to the same topic. The other authors declare that they have noconflicts of interest concerning this article.
Young people's uses of wearable healthy lifestyle technologies; surveillance, self-surveillance and resistance	2019	SPORT EDUCATION AND SOCIETY	Goodyear, VA and Kerner, C and Quennerstedt, M	10.1080/13573322.2017.1375907	In this article we focus on young people's uses of a wearable health device - Fitbit - and the associated health app.	Qualitative	UK	Young people	One hundred 13–14 years olds (53 females, 47 males), from five physical education classes in two schools were invited to participate. A minimum age of 13 was selected due to Fitbit's terms and conditions on age of use (Goodyear, Citation2017). The two schools varied in their socio-economic background, that added variability to the data through providing different contexts in which the young people would use and experience the Fitbit. School one was a non-selective private school in the South-East of England. School two was a comprehensive state school based in the North-West of England.	Young people's uses of wearable healthy lifestyle technologies			In turn, the young people resisted the educational value of the Fitbit and demonstrated a sceptical stance toward introducing health devices in school and physical education settings.								step and calorie burning targets set by the Fitbit device encouraged the young people to do more physical activity. Increases in physical activity occurred because of the self-surveillant practices promoted by the Fitbit through; (i) the monitoring and recording of steps and calories burned, and (ii) peer comparison (or monitoring). Surveillance and self-surveillance practices, however, were clearly connected to health equating to fitness and being 'fit' or not being 'fat'. These narrow interpretations of health, equally, underpinned resistance. Daily step and calorie burning targets, (i) did not sustain young people's engagement with the device beyond a few weeks, (ii) promoted negative feelings, and (iii) the device was resisted because it did not record physical activity accurately as part of young people's daily lives.	N

Older adults' experiences of using a wearable activity tracker with health professional feedback over a 12-month randomised controlled trial	2020	DIGITAL HEALTH	Brickwood, KJ and Williams, AD and Watson, G and O'Brien, J	10.1177/205520762 0921678	This study examined older adults' experiences with, and perceptions of, wearable activity trackers combined with health professional feedback after a year's use as part of a randomised controlled trial.	Quantitative	Australia	Older adults	A total of 20 of the 28 participants who completed the activity tracker intervention consented and took part in the focus groups (females n = 12, males n = 8, age 73.6 ± 5.5 years, BMI 31.4 ± 6.3 kg/m2). Participants were completing an average of 7098 ± 3384 steps per day and had a range of diagnosed chronic conditions including cardiovascular disease, diabetes, osteoarthritis and pulmonary conditions. All participants had at least one diagnosed chronic condition, with 65% (13 out of 20) participants having two or more diagnosed chronic conditions. Only one participant reported not using technology. The remaining participants reported using a mix of devices in their day-to-day lives, with mobile phone use more prevalent than smartphone use (50% compared to 30%). For participants who reported receiving support, the majority (55%) relied on friends or family with only one participant	Older adults' experiences of using a wearable activity tracker			Acceptability<br>The high rate of Jawbone use suggests that the Jawbone activity tracker was well-accepted by older adults (Jawbone was worn on an average of 86% (316 out of 365) of possible days). The majority (80%, n = 16) of participants used words such as 'encouraged', 'good' and 'motivating' to describe the Jawbone activity tracker, indicating an overall positive experience for most participants							Four themes were identified in the thematic analysis of the focus groups and included: increased sense of awareness of activity levels is related to motivation; the level of engagement with the activity tracker influences the user experience; the role of feedback from a health professional in providing ongoing support; and the role of habits in supporting long term behaviour change.		N
Adolescents with idiopathic scoliosis and their parents have a positive attitude towards the Thermobrace monitor: results from a survey	2017	SCOLIOSIS AND SPINAL DISORDERS	Donzelli, S and Zaina, F and Martinez, G and Di Felice, F and Negrini, A and Negrini, S	10.1186/s13013-017-0119-x	A temperature monitor is used to objectively measure brace wear time in adolescent idiopathic scoliosis. The reliability of this device have been demonstrated, and some specialists introduced the use of a compliance monitor as a standard of care in everyday clinical practice, as we did since 2010 with the Thermobrace (TB). The attitude towards these objective monitors has never been investigated. The present study aims to investigate the attitude of parents and patients towards the use of temperature sensors for measuring brace wear compliance.	Quantitative	Unspecified	Adolescents with idiopathic scoliosis and their parents	Among 364 invited patients to complete anonymously the questionnaire, 336 adhered by completing it; therefore, globally, the rate of responders was very high (93.2%), and most of them completed all the required sections of the questionnaire. The mean age of the recruited patients was 14.65 (SD 2.36), the mean Cobb angle was 34.18 (SD 13.57), and average brace wear prescription was 21.76 h per day (SD 2.53). We did not ask parents about their age, profession, nor other personal data.	Thermobrace monitor			Globally, the interviewed patients and parents showed a very positive attitude towards the TB monitor; in fact, the mean percentage of parents stating a completely or at least partially positive attitude towards this electronic device was 94.0% while among patients it was 85.6%.							All the answers have been evaluated and classified into three categories: the group of parents who wrote a very detailed description of the TB and its advantages, thus illustrating a very good understanding of the usefulness of the device (8.1%), the group who understood at least partially the usefulness of the TB sensor (88.0%), and those who completely misunderstood the significance and use of the TB sensor (1.5%).<br><br>A second question aimed to deepen the usefulness of the TB sensor; in fact, responders had to decide to whom the TB is most useful; multiple choices were allowed. This question showed a rate of responders of 100%. 35.3% of the responders consider the TB useful for parents, 56.9% consider it useful for patients, and 86.5% state that the TB sensor is useful for physicians. Only 0.6% answered "for none" and 1.2% answered "others." When considering the combined answers, we obtained the following rates: the TB is considered useful for both parents and patients by 22.5% of responders, for physicians and patients by 46.5% of responders, and for physicians and parents by 31.1% of responders. Among those		No competing interests to be declared by the authors, except for SN and AN who have some ISICO stocks.
Understanding persistence in the use of Online Fitness Communities: Comparing novice and experienced users	2016	COMPUTERS IN HUMAN BEHAVIOR	Stragier, J and Vanden Abeele, M and Mechant, P and De Marez, L	10.1016/j.chb.2016.06.013	Mobile and wearable technologies facilitate physiological data collection for health and wellness purposes. Users typically access these data via Online Fitness Community (OFC) platforms (e.g., Fitbit, Strava, RunKeeper). These platforms present users with functionalities centered on self-monitoring, social networking and enjoyment. In order to fully benefit from these functionalities, users need to make a habit out of integrating OFC use into their everyday workout routines. However, research suggests that users often fail to use OFCs over a longer period of time. This study sheds light on the factors that explain persisted OFC use.	Quantitative	Belgium	General public	Approximately 8000 tweets were collected in a one week period using the open-source solution yourTwapperkeeper as a tool for capturing tweets with the hashtag #Strava.	the use of Online Fitness Communities				significant correlation with enjoyment motives. The observation of these correlations led us to add three paths when fitting the model: a direct path from self-regulatory motives and social motives to habitual Strava use and a direct path from social motives to enjoyment motives. Perceived usefulness was also found to positively predict habitual Strava use More particularly, the direct path from enjoyment motives to perceived usefulness was significantly different between the groups, implying that enjoyment motives only impact significantly on perceived usefulness for experienced users. Although the parameter strength of perceived usefulness on habitual Strava use differed substantially between novice and experienced users, this difference did not								UNDECLARED
Smartphone Self-Monitoring to Support Self-Management Among People Living With HIV: Perceived Benefits and Theory of Change From a Mixed-Methods Randomized Pilot Study	2015	JAIDS-JOURNAL OF ACQUIRED IMMUNE DEFICIENCY SYNDROMES	Swendeman, D and Ramanathan, N and Baetscher, L and Medich, M and Scheffler, A and Comulada, WS and Estrin, D	10.1097/QAI.00000 00000000570	PLH (n = 50), primarily African American and Latino, were recruited from 2 AIDS services organizations and randomly assigned to daily smartphone (n = 34) or biweekly Web-survey only (n = 16) self-monitoring for 6 weeks. Smartphone self-monitoring included responding to brief surveys on medication adherence, mental health, substance use, and sexual risk behaviors, and brief text diaries on stressful events. Qualitative analyses examine biweekly open-ended user-experience interviews regarding perceived benefits and barriers of self-monitoring, and to elaborate a theoretical model for potential efficacy of self-monitoring to support self-management for multiple domains.	Qualitative	USA	people living with HIV	Daily Mobile A<br>"Assessment" Daily Mobile B<br>"Behavior<br>Change" Bi-weekly<br>Web Only C Total<br>Genderb<br>Female 1 7.1 4 20.0 1 6.7 6 12.2<br>Male 11 78.6 15 75.0 14 93.3 40 81.6<br>Transgender 2 14.3 1 5.0 0 0.0 3 6.1<br>Race<br>Black 7 50.0 8 40.0 9 56.3 24 48.0<br>Latino 1 7.1 3 15.0 4 25.0 8 16.0<br>Native American 0 0.0 1 5.0 0 0.0 1 2.0<br>White 4 28.6 6 30.0 3 18.8 13 26.0<br>Mixeda 2 14.3 2 10.0 0 0.0 4 8.0<br>Sexual orientationb<br>Bisexual 3 23.1 3 15.0 0 0.0 6 12.2<br>Gay 8 61.5 13 65.0 13 81.3 34 69.4<br>Heterosexual 2 15.4 4 20.0 3 18.8 9 18.4	Smartphone self-monitoring to support self-management among people living with HIV					Self-monitoring functions include reflection for self-awareness, cues to action (reminders), reinforcements from self-tracking, and their potential effects on risk perceptions, motivations, skills, and behavioral activation states. About one-third of participants reported that surveys were too long, frequent, or tedious. Some smartphone group participants suggested that daily self-monitoring was more beneficial than bi-weekly due to frequency and in-the-moment availability.				Participants also reported therapeutic benefits related to self-expression for catharsis, non-judgmental disclosure, and in-the-moment support. About twice as many daily self-monitoring group participants reported increased awareness and behavior change support from self-monitoring compared to bi-weekly web-survey only participants.	Benefits of Daily Smartphone Self-Monitoring Versus Bi-Weekly Web-Surveys<br>Participants' responses also suggested potential for greater benefits from daily smartphone self-monitoring compared to bi-weekly web-surveys alone. In general, participants in Group C (bi-weekly, web-only) reported fewer benefits when queried about useful aspects of completing surveys, and those reports tended to be less elaborate and centered around reflective processes, as shown in responses in Tables 2 and and3 3. In addition, a few participants noted benefits specific to daily mobile self-monitoring related to in-the-moment availability and daily routines,<br><br>"Cell phone is more helpful than the web survey at keeping me on track because it's always there." (Grp B, Age 39, Male, Native American);<br>"The surveys are time sensitive so I can say how I feel in a particular moment of the day." (Grp A, Age 51, Female, Black);<br>"Helps me keep a 'log', like therapy—but can do it every day instead of waiting for a week to see your therapist…Nice to do it throughout the day, multiple	Negative Feedback on Self-Monitoring<br>Participants identified a number of negative qualities of the surveys, such as being repetitive, too long, confusing, and intrusive. About one-third of participants described the surveys as "repetitive," "redundant", "monotonous," or "tedious," including participants in the bi-weekly web-survey only group. About one quarter of participants reported that surveys were too long or too frequent, with all but one participant being from the mobile phone groups. Some participants suggested improvements, for example,<br><br>"I like it, but I wish it was more than the same questions every day" (Grp A, Age 61, Male, Black).<br>About 20 percent of participants across the three study groups reported that the surveys were intrusive or were concerned about privacy, primarily around the sexual behavior surveys and the detailed partner-level reports. In one extreme example, likely in reference	N
How Do You Sleep? The Impact of Sleep Apps on Generation Z's Well-Being	2023	JOURNAL OF INTERACTIVE MARKETING	Attie, E and Meyer-Waarden, L	10.1177/109499682 21142806	Mobile health (mHealth) apps have fundamentally changed the usage of smartphones in people's daily lives. In this context, sleep apps, the most popular mHealth apps, can track and enhance user well-being. Understanding the antecedents of the usage of sleep apps is of timely research interest. This study focuses on whether and how sleep apps influence Generation Z users' well-being (as this generation represents a promising market segment for smart devices).	Quantitative	France	generation Z	A total of 182 responses were valid for statistical analysis (72% women; Mage = 20.4 years, SD = .82).	Sleep Apps on Generation Z's Well-Being				Consumers rate higher levels of well-being and usefulness regarding the sleep app before use than after use. Perceived usefulness can enhance usage and, in turn, well-being.				However, privacy concerns remain the primary reason for consumers' reluctance toward mHealth apps.	Health app managers should understand the importance of the utilitarian benefits of disruptive technologies, which can be enhanced through empowerment (i.e., self-tracking, self-knowledge, and self-management) and well-being benefits.	The proportions of mobile phone groups' (A and B) participants reporting awareness and change benefits		N
Personalization of wellness recommendations using contextual interpretation	2018	EXPERT SYSTEMS WITH APPLICATIONS	Afzal, M and Ali, SI and Ali, R and Hussain, M and Ali, T and Khan, WA and Amin, MB and Kang, BH and Lee, S	10.1016/j.eswa.2017.11.006	A huge array of personalized healthcare and wellness systems are introduced into the portfolio of digital health and quantified-self movement in recent years. These systems share common capabilities including self-tracking/monitoring and self-quantifications, based on the raw sensory data. These capabilities provide solid ground for the users to be more aware of their health; however, such measures are inefficient for changing the unhealthy habits of the users. In order to induce healthy habits in the users, a system must be capable of generating context-aware personalized recommendations. The main obstacle in this regard is the contextual interpretation of recommendations based on user's current context and contextual preferences. To resolve these issues, we propose a methodology of cross-context interpretation of recommendations (CCIR) for personalized health and wellness services.	Quantitative	South Korea, Pakistan, Vietnam, Spain, Myanmar, Ecuador, India, China, Bangladesh, and Uzbekistan.	General public	participants to observe the given context and respond to the recommendations. Among 50 participants, 40 were end users and 10 were domain experts who were having certain level of knowledge of the wellness domain and recommender systems. The portfolio of the end user participants includes 30 males and 10 females in the middle-aged group (25–49 years). These participants belong to 10 different nationalities including South Korea, Pakistan, Vietnam, Spain, Myanmar, Ecuador, India, China, Bangladesh, and Uzbekistan. The distribution of participants based on gender and ethnicity are shown in Table 7.	Mining Minds platform consists of an array of innovative services, tools, and techniques, working collaboratively to investigate human's daily life data, generated from heterogeneous resources, for personalized health and wellness support (Banos et al., 2016). It orchestrates the whole function into a layered approach to provide the personalized digital health and wellbeing services.			From these statistical analysis, we infer that the participants of both the groups are in agreement and have a higher level of central tendency and a lesser level of variability in their responses to the system.Finally, we record the response from both end-users and experts to find out the mutual relationships in their responses. Because of the unequal number of participants in each group, we converted the response values to the percentage response values as shown in Table 10. The average value (%) for the end-users is recorded as 86.688, which is very close to the average value (%) 88.500 of the expert users, means the participants of both the groups have almost similar opinion about the system. Also, a value-by-value comparison of the values in Table 10 is depicted in Fig. 8. It can be seen from the figure that there exists a very close relationship in the response of the participants from both the groups. It leads us to the conclusion that both the groups are agreed in their opinion with respect to the positive response, thus the system is considered to be acceptable.		Both RB and RI have same recommendations for 4 out of 14 aforementioned contexts. Hence, in only 10 out of 14 contexts most participants marked RI's recommendations ``unreasonable''. Varied personal preferences could be one of the reasons since some of these contexts are rather rare e.g. ``outdoors, commuting'', ``gym, sleeping'', and ``yard, sleeping'', etc. This analysis necessitates acquiring of personal preferences of the user							N
Charting Design Preferences on Wellness Wearables	2016	PROCEEDINGS OF THE 7TH AUGMENTED HUMAN INTERNATIONAL CONFERENCE (AUGMENTED HUMAN 2016)	Rantakari, J and Inget, V and Colley, A and Hakkila, J and ACM	10.1145/2875194.28 75231	This paper presents a study on people's preferences with wearable wellness devices. The results are based on an online survey (n=84), where people assessed different features in wearable wellness devices.	Quantitative	Finland	General public	Of these, 54 (64%) were female and 30 (36%) male. The average age of the participants was 29 years, ranging from 20 to 61 years (SD = 9.8), and 90% of them came from Finland.	Wellness Wearables					Battery lifetime was significantly more important for women than men, p = .057 (FU6: The device battery lasts for at least 24 hours). Sharing and competitive features were rated significantly higher by men than women (FS20 p = .0735 and FS21 p = .0138). In particular, for FS21 (FS21:I can easily compare my performance to other people's performances) the difference between genders was significant at the p<0.05 level. In general it may be noted that female participants tended to give higher scores on design requirements related to effortless use of the device (FU6, ID13) and 'self' focused aspects – privacy and comparison to one's own performance (F19, FS22). Male participants paid more attention to technical aspects, such as the configuration of the device, automatic activity recognition and detailed feedback.		24 hours (FU6) and that the device should be comfortable to wear (FF5). The least prioritized features were, the ability to share results with other people (FS20) and the ability to compare your performance with other people's performances (FS21). However, social aspects and sharing came up in comments. Overall, all requirements related to the form factor (5 items) and functionality (2 items) were ranked in the top half, i.e. among the 11 highest prioritized features of the total of 22. Hence, the industrial design aspects in the design requirements for wearable wellness technologies were emphasized. Additionally, in the free text comments, the industrial design and form factor aspects also gained attention. The figure shows that overall concern when regarding the requirements for wearable health devices was given to functionalities and form factor. In the freeform comment section at the end of the survey, sustainability and green values were raised as an additional theme. In addition, some participants commented on the technical aspects and data analyzing capabilities of the devices. Moreover, the viewpoint of multiple device ownership was mentioned: "[The device] should integrate itself into my device ecosystem" (#1,	The main reason for stopping using the devices was that users felt that they did not get relevant feedback from the devices (53%).				UNDECLARED
Designers in White Coats: Deploying Ovum, a Fertility Tracking Device	2020	PROCEEDINGS OF THE 2020 CHI CONFERENCE ON HUMAN FACTORS IN COMPUTING SYSTEMS (CHI'20)	Homewood, S and Boer, L and Vallgarda, A and ACM	10.1145/3313831.33 76528	The research product Ovum was designed to facilitate a DIY, shared, domestic experience, rather than an expert-led, individual, clinical experience of fertility tracking. Ovum uses the method of saliva sampling to determine ovulation. This paper unpacks experience of seven couples trying to conceive who participated in a three-month long project of Ovum.	Qualitative	Sweden	couple who were attempting to conceive/women	All participants were living in Sweden. All were cis-gendered, heterosexual couples, though we did not choose participants based upon these criteria. Ages of participants ranged from 23 to 43	a Fertility Tracking Device				broader reflections on gender roles in fertility tracking; "if I would rewind history I would do it earlier, I would take this conversation about how can we both be a part of this earlier. Like this is our journey together. I just thought that it was my responsibility. But it isn't! To check it out and to have control over it and it's my cycle and I have to know. But I don't have to, we, we can do it together." (P1A).	Initial reactions from participants after receiving the device were that is was "easy" (P1A, P2A, P6A), "clear" (P2A, P6A), and "user friendly" (P4A). These opinions refer both to interactions with the device and with the method of saliva tracking. It quickly became evident to us that we could not easily separate these two aspects of the device. Since saliva tracking is not a well-known or experienced method of fertility tracking, reactions to it were strong and in fact were the major topic of our interviews. Initial perceptions of the device changed over the threemonth study. This was due to the fact that the method of salivatory tracking proved to give inconsistent results for all participants at one time or another.						Ambiguity in the results shown by Ovum were received as signals that it was their own bodies that were producing these erratic results.	UNDECLARED
Refined Analysis of Older eHealth Users From an Agency Perspective: Quantitative Telephone Interview Study	2023	JMIR AGING	Brainin, E and Neter, E	10.2196/40004	This study aimed to obtain a complete and detailed description of eHealth uses among older adults; examine whether demographic characteristics such as age, gender, and education (previously related to eHealth misuse) are still associated with the various eHealth clusters; and determine whether contextual factors such as changes in the health condition of older eHealth users or their loved ones are associated with older adult eHealth use.	Quantitative	Israel, Arab, and Russia	Older adults	The characteristics of the participants are presented in Table 1. The sample comprised 63.1% (279/442) women and 95.2% (421/442) Jewish participants. Participants' age ranged from 50 to 87 years with a mean age of 61.05 (SD 8.23) years. The mean age of the men participants was considerably higher than that of women participants (mean 62.2, SD 8.85 vs mean 60.38, SD 7.77, respectively; F1,439=5.07; P=.03). Approximately half (225/442, 50.9%) of the sample had postsecondary education, and 74.4% (329/442) were married. A total of 38% (168/442) of the participants reported one or more chronic ailments.	eHealth						third step. Offline health care use (β=.09; t395=1.91; P=.06), health information seeking for family and friends (β=.27; t395=5.55; P<.001), frequency of internet use (β=.09; t395=1.94; P=.05), and eHealth literacy (β=.25; t395=5.00; P<.001) were found to be significantly associated with instrumental and administrative information seeking such that the more participants used offline health care services, looked for health information for family and friends, used the internet frequently, and had high eHealth literacy, the more they sought instrumental and administrative information.When controlling for personal attributes, age, gender, and education were no longer predictors of eHealth use, nor was a chronic ailment. Instead, internet experience, frequency of internet use, and perceived eHealth literacy were associated with 3 eHealth clusters. Looking for health information for family and friends predicted all 4 eHealth clusters.Many older adults can overcome structural barriers such as age, gender, and education.						N
Patient perception and use of an insulin injector/glucose monitor combined device	2004	DIABETES EDUCATOR	Gooch, B and Hassman, DR and Liang, J and Smedegaard, JK and Skovlund, S and Berg, B and Lyness, W and Schneider, SH and Bergenstal, R and Bode, BW and Gooch, B and Green, A and Hassman, DR and Hollander, PA and Jovanovic, L and Lerman, S and Normandin, DM and Passaro, M and Rizvi, S and Schneider, S and Schwartz, S and Shelmet, J and Sievers, R and Weinstein, R and Weiss, R and Zervos, M and The	10.1177/014572170 403000223	This clinical trial assessed patient preference, satisfaction, and use of an insulin injector/glucose monitor combination device versus syringes and a separate glucose monitor	Qualitative	Unspecified	Diabetes patients		Glucose Monitor Combined Device					Most of the patients preferred using the combination device to syringes and a separate meter.							UNDECLARED
Clinicians and Older Adults' Perceptions of the Utility of Patient-Generated Health Data in Caring for Older Adults: Exploratory Mixed Methods Study	2021	JMIR AGING	Kim, B and Ghasemi, P and Stolee, P and Lee, J	10.2196/29788	This study aims to explore the similarities and differences in the perceptions of older adults and clinicians with regard to how various types of patient-generated health data (PGHD) can be used to care for older adults.	Mixed	Canada	Older adults	Of the 9 participants, 4 (44%) identified themselves as clinicians, including 1 primary care physician, 2 registered nurses, and 1 registered physiotherapist. The mean age of the clinicians was 38.3 (SD 7) years, and 3 of them were women. The remaining 56% (5/9) of the participants identified themselves as health care users. The mean age of this group was 81.0 (SD 9.1) years, and 4 of the 5 older adults were women (Table 1).	Patient-Generated Health Data in Caring for Older Adults					Not all older adults agreed with the suspected tendency toward selective disclosure of PGHD. Two older adults expressed that they were less likely to share PGHD when they were noncompliant and inclined to share only compliant information. However, one participant was comfortable sharing their PGHD regardless of compliance. Older adults perceived PGHD to be difficult to use as the volume of data would be too large, and it would be time-consuming to gain an understanding of their health and the effectiveness of care. Clinicians expressed the significance of a decision support system to act on the PGHD.			Older adults were reluctant to share their PGHD with their clinicians as they were uncertain of the use of PGHD by their clinicians and the skill levels of their clinicians to use them. A large amount of data was viewed as a major hindrance to the use of PGHD by both older adults and clinicians. Older adults felt overwhelmed when trying to review and understand the data. Older adults felt discouraged from sharing the data as they perceived that reviewing PGHD was a time-consuming task and felt that clinicians would not have enough time. Despite the issues of information overload and lack of time, clinicians saw clinical value in collecting more PGHD.		Older adults felt that monitoring PGHD increased the transparency of their (lack of) engagement in healthy behaviors. Older adults understood that increased transparency encouraged and motivated compliance, although this was not explicitly stated.Older adults and clinicians generally agreed on the benefit that increased transparency arising from PGHD sharing has on the care they provide or receive. Patients also raised the need for additional education, which might improve the understanding of the need for PGHD.Stress level as PGHD was discussed only by older adults, and it was portrayed as having significant importance for overall well-being.	Older adults and clinicians perceived that the lack of clinical knowledge by patients leads to a collection of irrelevant PGHD and decreases the usefulness of the information. In contrast, older adults viewed education on self-management as a key component in understanding the importance of PGHD. Gaming mHealth apps and wearable devices used by patients to collect favorable data were viewed as a threat to the reliability of PGHD.	N
Exploring the Influencing Factors on User Experience in Robot-Assisted Health Monitoring Systems Combining Subjective and Objective Health Data	2023	APPLIED SCIENCES-BASEL	Neef, C and Linden, K and Richert, A	10.3390/app130635 37	Featured Application is health monitoring system that combines commercially available products, such as sensing devices and apps, with a social robot to conduct health assessments and collect subjective and objective health data. In this work, we present an exploratory user experience (UX) and usability study we conducted with older adults as the target group of the system and with younger expert users who tested our system.	Quantitative	Germany	Older adults	participants from the target group, whose ages ranged from approximately 50 to 90 years. Five of the eight participants were between the ages of 60 and 79, two were between 80 and 89, and one participant was aged between 50 and 59. They all lived in the same care facility in Germany and were recruited by facility staff, who were instructed to select participants interested in testing assistive technologies in the context of health monitoring.	Robot-Assisted Health Monitoring Systems Combining Subjective and Objective Health Data					The UX and usability of the individual components of the system were rated highly in questionnaires in all sessions. All participants also said they would use such a system in their everyday lives, demonstrating the potential of these systems for self-managing users' health. Finally, we found factors such as previous experience with social robots and technological expertise to have an influence on the reported UX of the users.							N
Smartphone Self-Monitoring by Young Adolescents and Parents to Assess and Improve Family Functioning: Qualitative Feasibility Study	2020	JMIR FORMATIVE RESEARCH	Swendeman, D and Sumstine, S and Brink, A and Mindry, D and Medich, M and Russell, M	10.2196/15777	This study aimed to examine user experiences of feasibility, acceptability, and reactivity (ie, changes in awareness and behaviors) of using a smartphone app for self-monitoring of family functioning with 36 participants across 15 family dyads and triads of young adolescents aged 10 to 14 years and their parents.	Qualitative	USA	Young Adolescents and Parents	Children were on average aged 12 years (SD 1.44), mothers were 46.25 years (SD 3.81), and fathers were 40.33 years (SD 3.51). Approximately half were white (n=20), one-third were Latino (n=11), 9% (3/35) were Asian, and 3% (1/35) were black. Tables 2 and and3 3 present more demographic results for children and parents, respectively.	Smartphone Self-Monitoring by Young Adolescents and Parents to Assess and Improve Family Functioning			Conclusions<br>The results of this study support the feasibility and acceptability of using smartphone EMA by young adolescents and parents for assessing and self-monitoring family daily routines and interactions.							Over 80% (25/31) of participants reported increased awareness of their families' daily routines and functioning of their families. Most also reported positive behavior changes in the following domains: decision making, parental monitoring, quantity and quality of time together, communication, self-regulation of stress and conflict, discipline, and sleep.		N

Creating connections - the development of a mobile-health monitoring system for heart failure: Qualitative findings from a usability cohort study	2016	DIGITAL HEALTH	Chantler, T and Paton, C and Velardo, C and Triantafyllidis, A and Shah, SA and Stoppani, E and Conrad, N and Fitzpatrick, R and Tarassenko, L and Rahimi, K	10.1177/205520761 6671461	There is significant interest in the role of digital health technology in enabling optimal monitoring of heart failure patients. To harness this potential, it is vital to account for users' capacity and preferences in the development of technological solutions. We adopted an iterative approach focussed on learning from users' interactions with a mobile-health monitoring system.	Qualitative	UK	heart failure patients	[illegible] included 58 people with a median age of 77 years (range 21–94), 21 (36%) were female, three (5%) represented ethnic minorities, 40 (23%) suffered from diabetes and 10 (18%) from Chronic Obstructive Pulmonary Disease (COPD). From a social perspective 12 (21%) participants lived on their own, three (5%) lived in sheltered accommodation, 39 (67%) lived with their partner, and four (7%) resided with other family members. The qualitative research subgroup did not differ considerably from the main sample apart from the fact that it included all participants from ethnic minority backgrounds and less people living on their own (Table 1).	Internet-enabled tablet computers	Self-monitoring was defined as 'keeping account of myself' (P9) by one of these participants, a way of knowing more about one's health and learning more about specific readings such as blood pressure and weight.				Participants described receiving a message from the team as highly motivational since it provided evidence of a connection between themselves and health professionals. They were not just monitoring their symptoms in isolation but there was someone else reviewing their data with them. The contact button in turn served a dual purpose; it helped resolve technical problems, and humanised the system by creating opportunities for connection and conversation.<br><br>The system included a 'how to keep healthy' section where participants could access video clips. Participants expressed varied opinions about these resources, ranging from very positive to ambivalent. A recently diagnosed man found that the patients' stories helped him come to terms with his condition whereas others were not interested in hearing about others peoples' experiences. For some this was because they thought they knew all they wanted to know about their condition, while for others it was because they were more interested in materials tailored to them individually.		Our findings indicate that digital health technologies need to create and extend connections with health professionals, be incorporated into users' daily routines, and be personalised according to users' technological competencies and interest in assuming a proactive or more passive role in monitoring their condition.	As self-monitoring was a novel activity for many participants, they were often unclear about how to evaluate the data they were collecting. Whilst most participants were keen to record their symptoms and measurements and increase their understanding of their condition, they were concerned and hesitant about assuming responsibility for interpretation. Indeed, this perceived responsibility was described as worrying by one couple (P3) because they did not know whether the participant's measurements were within acceptable parameters. Similarly, three other participants (P13, P36, and P38) stated that they preferred to leave the interpretation of their measurements to health professionals. Despite some concerns about interpretation, the practice of self-monitoring was described variously as 'reassuring something that increases your confidence, helps you set and evaluate exercise and weight loss goals, and enables you to gauge your need for medical attention'.				N
Developing mHealth Remote Monitoring Technology for Attention Deficit Hyperactivity Disorder: A Qualitative Study Eliciting User Priorities and Needs	2016	JMIR MHEALTH AND UHEALTH	Simons, L and Valentine, AZ and Falconer, CJ and Groom, M and Daley, D and Craven, MP and Young, Z and Hall, C and Hollis, C	10.2196/mhealth.50 09	To explore patients', parents', and health care professionals' views and attitudes toward using digital technology for remote monitoring during titration for attention deficit hyperactivity disorder (ADHD).	Qualitative	UK	patients with ADHD, parents	Both the patient and parent samples were predominantly white British, and were mainly male, and female, respectively. In all groups, there was a wide spread of ages, apart from YP, who were all aged 12 or 13 years. The HCP sample included 9 medical staff and 15 non-medical clinical staff, of which 11 were prescribers, 5 were in non-clinical health care roles, and 2 were IT managers.	Remote Monitoring Technology for Attention Deficit Hyperactivity Disorder					All participant groups were positive about using RMT in the treatment of ADHD, They identified that RMT had the most potential for use in the ongoing management and support of ADHD, rather than during the distinct titration period.<br><br>Conclusions<br>RMT has the potential to augment support and care for ADHD, but it needs to go beyond the titration period and offer more to patients and families than monitoring through outcome measures. Developing and evaluating an mHealth app that incorporates the key features identified by end users is required.			but they were also aware of barriers to its use, especially around access to technology and integrating RMT into clinical care.		Participants identified features of RMT that could improve the quality of consultations and support greater self-management.		Conflicts of Interest: DD receives education, travel, and research funding from Eli Lilly and Co., Shire Pharmaceuticals, and HP Pharma. CH receives research funding from Shire Pharmaceuticals
Users' psychological perception and perceived readability of wearable devices for elderly people	2016	BEHAVIOUR & INFORMATION TECHNOLOGY	Fang, YM and Chang, CC	10.1080/0144929X. 2015.1114145	In this study, three common wearing positions - wrist, upper arm, and neck - were investigated in terms of psychological perception and perceived readability.	Mixed	Taiwan	older adults	The sample comprised 24 participants aged over 50 years in Miaoli area, Taiwan. 54% of them were older than 65 years of age. 71% of them were female; and 50% of participants had experience of using smartphones. They did not have any mental or physical disabilities.	wearable devices for elderly people			The interview results showed that the wrist device received the highest acceptance for all participants. For the other two positions, the participants claimed that the arm devices were not easy to read. The devices would interfere with putting on or taking off the clothes. For this reason, the arm device was less welcome than wrist devices. The opinions for the device attached to the neck were controversial. Some of them claimed its image was easy to be associated with negative objects such as a dog collar. This device received better acceptance by female participants. By contrast, the other participants stated that they could accept the device attached to the neck because of its sensor functions of detecting comprehensive physiology data. One of the participants preferred the function for brainwave monitoring, because of her previous cerebral disease. Some participants suggested that, because these devices needed to be attached on the body for a long time, the volume of these devices should be reduced, or even be hidden under the clothes. But they were not willing to sacrifice the size of the screen to compromise the convenience of reading.	To summarise the differences in attitude by gender factor (Table 2), both genders had the highest acceptance of the wrist device and the lowest acceptance of the neck device. Female participants had a more positive impression of wearable devices than male participants did.To summarise the effects of participants' experience with using smartphones (Table 3), it can be concluded that smartphone users showed higher acceptance of wrist devices, but less acceptance of neck devices. Inexperienced smartphone users were more positive towards arm devices.To summarise the factor of participants' requirement of recording physiological data regularly (Table 4), participants who needed to record data showed higher acceptance of wrist devices, and less negative feelings towards the neck device. Participants who did not need to record data showed higher acceptance of wrist devices, but relatively low acceptance of the neck device.To summarise the factor of the need to visit the hospital regularly for follow-up health examination (Table 5), the	The data revealed that the participants showed strong willingness to demonstrate the wrist wearing parts of the body. However, the wearing the devices at the necks had an average rating at 2.84. It was the least favourable location for ageing users. Additionally, all the participants in general showed weaker willingness to demonstrate the wearable devices.<br><br>The data also showed that three wearing parts caused less anxiety with the total average rated at 3.60. It can be seen that ageing seniors usually felt uncomfortable when using the wearable devices.<br><br>It revealed that all three different wearing parts of the body provided acceptable viewing angle for users' to check out the displayed information, with the average incidence of wearing on the wrist highly rated at 5.74. It can be concluded that wearing the device on the wrist provided the best viewing angle among different parts of the body.<br><br>It revealed that three wearing parts in general can provide an acceptable convenience for information reading. Among them, the average incidence of wearing the device on the wrist was highly rated at 6.00. It can be seen that the wrist provided the most convenient angle for information reading.<br><br>To conclude the results of four groups of questionnaires, comparing the device attached to the three positions with Likert scale for psychological perception (demonstration willing and anxiety), the wrist device received the highest	Most of the participants in this study did not live with their children, and had some chronic diseases such as hypertension or diabetes. The main reason why they welcomed this device was its function for informing their family about the health condition, easing the family members' concern and worry. With positive attitudes, the participants looked forward to these devices in the market in the near future.						N
Monitoring and Managing Lifestyle Behaviors Using Wearable Activity Trackers: Mixed Methods Study of Views From the Huntington Disease Community	2022	JMIR FORMATIVE RESEARCH	Morgan-Jones, P and Jones, A and Busse, M and Mills, L and Pallmann, P and Drew, C and Arnesen, A and Wood, F and Domino-HD Consortium	10.2196/36870	This study aimed to explore the perceptions, motivators, and potential barriers relating to the adoption of wearable activity trackers by people with Hungtington disease (HD) for monitoring and managing their lifestyle and sleep.	Mixed	UK	people with Huntington Disease	Participants, n (%)<br>Age group (years)<br>≤24 7 (6.7)<br>25-34 18 (17.1)<br>35-44 23 (21.9)<br>45-54 20 (19)<br>≥55 35 (33.3)<br>Not specified 2 (1.9)	Wearable Activity Trackers				The only differences observed were in the behavioral intention questionnaire response score because of age (Table 4), with the oldest age group (≥55 years) having a lower median response score than the two youngest age categories (≤24 years and 25-34 years). However, pairwise comparisons were not statistically different when P values were adjusted for multiple comparisons (adjusted P>.05). Responders who had a genetic diagnosis of HD were found to have a significantly more positive median response for the ease of use, enjoyment, attitude, and behavioral intention sections when compared with those who cared for or were a family member of someone with HD.	The participants were overwhelmingly positive regarding the use of wearable activity trackers (Table 3). All sections of the questionnaire produced a median cohort response >2.5, representing a tendency for positive opinions toward the use of wearable activity trackers (such as devices being advantageous, easy and enjoyable to use, and compatible with one's lifestyle and users being able to understand the information from wearable activity trackers and willing to wear them).	There was a strong desire to receive feedback from a wearable activity tracker to increase personal motivation, allowing the visualization of goals:	All participants agreed that battery life was an important factor when considering if a device was easy to use. It was acknowledged that although charging a device was a necessity, a short battery life was not synonymous with ease of use. Those who already owned a wearable device noted how often and for how long charging was required:<br><br>Concerns were raised as to whether people with HD, particularly as the disease progresses, would be able to remember to remove a device, charge it, and put it back on. This process needed to be simplified and as infrequent as possible. A device that is waterproof and, therefore, reduces the need to take off and put back on again was similarly deemed beneficial. It was also recognized that a simple watch strap would be required so that those with progressive motor symptoms would be able to wear and remove the device. One participant mentioned using different types of straps with their current device, including a magnetic strap that easily clicks together.<br><br>The preferred location for a wearable activity tracker was the wrist; however, interest was also shown in devices that could be worn under clothes so that they could be worn more discreetly. Comfort was believed to be a key factor in device adoption, with participants wanting a device that was as unobtrusive as possible and not bulky or large. However, it was recognized that if there was a need and benefit from using a device, then appearance was not a critical factor in device		The participants also believed that some medical professionals were not very receptive to patients producing their own data. Participants believed that getting clinicians involved in the process of deciding what to measure and how to feed back to health professionals could improve their acceptance of the technology.	The participants suggested that wearable activity trackers opened up the possibility of new ways for clinicians, researchers, and patients to diagnose, monitor, and manage HD. The need to improve how patients with HD interact with their health professionals was noted, along with the advantages of generating longitudinal data to assist in clinical decision-making rather than relying on how a patient presents on a particular day in the clinic:<br><br>Those who had experience of using wearable activity trackers described them as being an integral component of a healthy lifestyle, giving them motivation and encouragement to reach their goals and a way of recording success:	Concerns about data security were also raised. It was felt that the misuse of personal data by third parties could lead to potential discrimination and that people with neuropsychiatric disease features may be obsessed with data security. Dependence was felt to lead to unnecessary stress when trying to meet health-related targets: However, the participants recognized that not hitting short-term targets may lead to disappointment and subsequent demotivation. For someone with HD who is seeing a decline in function and potentially not meeting their activity targets, this decline in function may be psychologically damaging:<br><br>Although many benefits of using wearable activity trackers were noted, there was a strong feeling that these will only be realized if wearable technologies can record measurements with a reasonable level of accuracy (with mention of an 85% accuracy threshold considered as acceptable):	N
Clinically Meaningful Use of Mental Health Apps and its Effects on Depression: Mixed Methods Study	2019	JOURNAL OF MEDICAL INTERNET RESEARCH	Zhang, RW and Nicholas, J and Knapp, AA and Graham, AK and Gray, E and Kwasny, MJ and Reddy, M and Mohr, DC	10.2196/15644	This study aimed to classify user behaviors in a suite of mental health apps and examine how different types of app use are related to depression and anxiety outcomes.	Mixed	USA	depression patients	A total of 301 eligible participants were enrolled in the randomized trial. The majority of participants were female (228/301, 75.7%), and the mean age was 37 (SD 11.84) years, ranging from 18 to 69 years. Most (237/301, 78.7%) of the participants identified themselves as white, 29 (9.6%) as African American, 10 (3.3%) as Asian, and 25 (8.3%) as "other." The mean baseline level of depression (PHQ-9) was 13.21 (SD 4.63), and the mean baseline level of anxiety (GAD-7) was 11.98 (SD 4.02). A total of 10 participants discontinued treatment and were lost to follow-up.	Mental Health Apps										Participants who engaged in self-tracking experienced reduced depression symptoms, and those who engaged with learning and goal setting at a moderate level (ie, not too much or not too little) also had an improvement in depression.		Conflicts of Interest: DCM has an ownership interest in Adaptive Health, Inc, which has a license from Northwestern University for IntelliCare and has accepted honoraria from Apple Inc. AKG and MJK have received consulting fees from Actualize Therapy, LLC. None of the other authors have conflicts to declare.
Patients' Use of Self-Monitored Readings for Managing Everyday Life with COPD: A Qualitative Study	2013	TELEMEDICINE AND E-HEALTH	Huniche, L and Dinesen, B and Nielsen, C and Grann, O and Toft, E	10.1089/tmj.2012.01 35	Effects of self-monitoring depend on how patients engage with readings and how this engagement is used for managing chronic disease. This article reports on a study of how chronic obstructive pulmonary disease (COPD) patients made use of readings during 16 weeks of self-monitoring.	Qualitative	Denmark	people with COPD	TELEREHABILITATION AND INTERVIEW PARTICIPANTS BY GENDER<br>VARIABLE MALE (N=8) FEMALE (N=14)<br>Age (years) (IQR) 69.4 (64, 73.8) 66.4 (44.6, 81.1)<br>Married/cohabiting (n) 3 4<br>Living alone (n) 5 10<br>Employed (n) 0 2<br>Retired (n) 8 12<br>Smoker (n) 2 2<br>Former smoker (n) 6 12<br>Using oxygen at home (n) 0 2<br>FEV1 (L) (IQR) 0.97 (0.11, 1.41) 0.81 (0.36, 1.34)<br>Weight (kg) (IQR) 85.74 (50.8, 108.1) 70.23 (50, 85.9)<br>Body mass index (kg/m2) (IQR) 28.4 (18.6, 35.7) 26.36 (20.2, 34)<br>Oxygen saturation (% on ambient air) 92.5 (90, 95) 93.3 (91, 96)<br>Blood pressure (mm Hg) (IQR) 142/83 (107/80, 180/90) 134/81 (97/52,	self-monitored readings for managing everyday life with COPD						Evaluating State of Health<br>The majority of patients interviewed (17 of 22) stated that they engaged with readings as part of their efforts to manage chronic illness.<br><br>Keeping Track of Health<br>Patients also used readings to keep track of their health. Judging When to Contact Health Professionals<br>Several patients stated that they used readings to help them judge whether and when to contact a health professional. Making and Revising Plans for Activities<br>Patients also used readings to plan activities for the day. For example, a patient commented that a reading of low oxygen levels may prompt a decision to postpone a planned walk into town because of fear of not being up to it.Motivating Exercise<br>Other patients used readings of low oxygen levels for the opposite purpose, namely, to motivate as much physical exercise as they are capable of that day in order to enhance oxygen uptake. Engaging Close Relatives<br>Several patients experienced home monitoring as a way for relatives to become involved in the				On the one hand, numbers are encouraging when they are better than usual or better than expected and reassuring when they stay within the acceptable range over time.	On the other hand, numbers may be experienced as depressing when worse than usual or worse than expected, as worrying when falling below an acceptable range, and as disturbing when fluctuating or not corresponding to patients' own sense of health. Even if being out-of-range is not positive, the worrisome experience of feeling poorly was scaffolded by the explanation provided by readings	N
Personal Health Technologies in Employee Health Promotion: Usage Activity, Usefulness, and Health-Related Outcomes in a 1-Year Randomized Controlled Trial	2013	JMIR MHEALTH AND UHEALTH	Mattila, E and Orsama, AL and Ahtinen, A and Hopsu, L and Leino, T and Korhonen, I	10.2196/mhealth.25 57	This study investigated personal health technologies in supporting employee health promotion targeting multiple behavioral health risks.	Mixed	Finland	Employee	Technology group, n=114<br>Sex, male (%) 33 (28.9%)<br>Age years, mean (SD, min–max) 44.6 (SD 7.1, 30–55)<br>BMI kg/m2, mean (SD, min–max) 27.1 (SD 4.0, 19.6–34.3)<br>Education (% college/university or higher) 67 (58.8%)	Personal Health Technologies				The sustained users were slightly older than the non-sustained users (Table 4).	The weight scales and pedometer were regarded as the best technologies for supporting wellness management in all post-intervention questionnaires, followed by Wellness Diary and heart rate belt (Figure 4).According to the usefulness statements, the scales, pedometer, and Wellness Diary were also most often the highest rated (Multimedia Appendix 1). For most technologies, satisfaction decreased slightly over time.	According to the interviews, the important motivational factors of the technologies were the ability to see one's progress and be reminded to do an activity, such as walking or exercising (Multimedia Appendix 2). The main appeal of the weight scales and pedometer were their simplicity, ease of use, and concreteness.		[illegible] study (Multimedia Appendix 3). In most cases this meant they had either stopped using certain technologies or started using them less often because they no longer needed them or needed them less. The users also changed technologies during the study. In the case of the pedometer, the most common reason reported was that the user had already learned enough. The reasons for the decrease in usage of the Wellness Diary were a change of routines from daily entries to entering several days' data at a time or stopping usage of certain variables due to a perceived lack of need. The interviewees also reported taking breaks, and recommended that the technologies could better support such intermittent usage. A typical time to take a break was summer holidays. Some of the respondents continued to use the technologies after a break but others did not.Typical barriers to using the technologies included problems with the phone or the Portal being down. An unexpected barrier to use reported by some interviewees was a sense of irritation at being pressurized by the technologies to do healthy activities (Multimedia Appendix 2).		[illegible] motivated them to maintain or improve personal wellness. The same was true for 68–83% of respondents about the pedometer and for 43–59% about the Wellness Diary. The benefits of the Wellness Diary were the record it provided of personal progress and development in long-term health data through graphical feedback. The heart rate belt and analysis report were valued for the interesting and all-round feedback. The benefits of the Mobile Coach were seen to be its adaptive exercise programs and coaching. The interviewees also noted the benefits of SelfRelax, which provided relaxation programs for specific situations, such as falling asleep, relaxing after work, or unwinding after a challenging encounter.Apart from the health insights, willingness to change and lower stress, the perceived benefits decreased somewhat from 6 to 12 months.		N
Users' Perceptions of a Digital Stress Self-monitoring Application: Research Insights to Design a Practical Innovation	2022	UNIVERSAL ACCESS IN HUMAN-COMPUTER INTERACTION: NOVEL DESIGN APPROACHES AND TECHNOLOGIES, UAHCI 2022, PT I	Smitt, MS and Montakhabi, M and Morton, J and van Leeuwen, C and Bombeke, K and Jacobs, A	10.1007/978-3-031-05028-2_22	The purpose of this study is to explore the impact of such digital self-monitoring technology for stress.	Qualitative	Belgium	General public	Variable<br>Mean (SD)/N (%)<br>Gender<br>8 females (67%)<br>Age<br>53 years (12)<br>Tech proficiency (1:5)<br>4.29 (0.69)	a Digital Stress Self-monitoring Application					The majority of the participants experienced a discrepancy between their perception of daily experienced stress and the stress predictions coming from the stress algorithm.<br><br>The second group of participants decided to reject the technology as the discrepancy between their own stress experience and the algorithmic findings resulted in a disbelief of distrust in the technology.		Request for Transparency： Regardless of the type of emotional response the participants had to their stress predictions, most of them expressed a curiosity to understand how stress can be determined. Quality Assessment of Algorithm.<br>Participants who expressed a discrepancy between the self perceived stress and the algorithm outcome, wondered about the quality of the stress algorithm.			The use of self-monitoring for stress made several participants reflect on their behaviour, emotions and cognition. A In this study we found that participants expressed more 1) self-awareness, such as "I have been listening more to my body" (P04), 2) better recognition of behavioural patterns when stressed, such as "I became more aware of the hours in the day and what I did at that time because of the study." (P04), referring to the smartphone questions about their stress experience, and 3) a better sense of time and activities due to the reflective questions. It made participants reflect on their day and on their emotions more than they would do without the use of the wearable, together with the triggered smartphone questions	lthough several participants expressed doubts about the accuracy of the algorithmic predictions, partaking in the study did have advantages that were independent from the evaluation of the technology.	UNDECLARED
Attitudes of Heart Failure Patients and Health care Providers towards Mobile Phone-Based Remote Monitoring	2010	JOURNAL OF MEDICAL INTERNET RESEARCH	Seto, E and Leonard, KJ and Masino, C and Cafazzo, JA and Barnsley, J and Ross, HJ	10.2196/jmir.1627	The objective of our study was to assess the attitudes of heart failure patients and their health care providers from a heart function clinic in a large urban teaching hospital toward the use of mobile phone-based remote monitoring.	Mixed	Canada	Heart Failure Patients	Variable Response, N=94<br>Mean age, years (SD) 54.6 (13.4)<br>Gender Male 74 (79%)<br>Female 20 (21%)<br>Ethnicity Caucasian 71 (76%)<br>African Canadian 7 (8%)<br>Southeast Asian 4 (4%)<br>Chinese 4 (4%)<br>Other 7 (8%)<br>Marital status Married 62 (67 %)<br>Never married 17 (18%)<br>Divorced 10 (11%)<br>Widowed 4 (4%)<br>Highest education achieved Less than high school 7 (8%)<br>High school 25 (27%)<br>Trade or technical training 16 (17%)<br>College/university undergraduate 37 (40 %)<br>Postgraduate 8 (9%)<br>Income < $15,000 20 (21%)<br>$15,000 - $29,999 17 (18%)<br>$30,000 - $49,999 17 (18%)<br>$50,000 - $74,999 14 (15%)	Mobile Phone-Based Remote Monitoring			The patients indicated that they thought it was important to monitor their weight and blood pressure. They were slightly more comfortable using a mobile phone than a computer (P < .001, 2-tailed paired Student t test, t 89 = 4.13), but rated the comfort level high for both. Most patients could easily access a computer. Patients rated their confidence in looking up health information on a mobile phone and computer equally high. Patients indicated moderately high confidence that their privacy would be secure if their health information was accessible by a computer.		All patients who owned home blood pressure monitors and weight scales thought they were easy to use. In addition, some patients had access to a computer and many already owned a mobile phone. Several of the patients who were not accustomed to the technology stated that they would be able to receive help from family members (eg, their spouses and children).<br>Both the interviewed patients and clinicians thought that older and less technologically savvy patients could have trouble operating the mobile phone. In particular, they thought that the small buttons and font on the mobile phone could cause difficulty to some patients. However, none of the interviewed patients thought that they themselves would have significant problems using the equipment.	Interviewed patients stated that they would be willing to try using the proposed remote monitoring system under the following conditions and caveats:<br>First, the monitoring system should be an adjunct to their relationship with their clinician at the heart function clinic. It should not be a replacement.<br><br>Second, patients would adhere to taking daily measurements long-term if they perceived clear tangible benefits from using it. The patients also stated that they would monitor their weight, blood pressure, and other factors more closely if their heart condition ever worsened.	Third, the system should be as easy to use as possible. They also requested appropriate training and a way to get technical support if they needed it.	Fourth, some patients questioned the necessity of monitoring their blood pressure daily. Some patients did not believe that they needed to take their blood pressure daily because their blood pressure in the past had been stable.<br>System not suitable for all patients Patients with poor vision could have trouble reading the mobile phone screen, and patients with inadequate manual dexterity could have problems entering information on the mobile phone keypad. However, none of the interviewed patients thought they themselves would have these problems. Patients also had concerns of getting used to the technology, but they thought they would be able to learn to use it with technical support and training. Some patients stated that their family members could help them use the technology.<br>Inappropriate automated instructions The system might instruct them to go to the emergency department (ED) unnecessarily, which would contribute to the backlog in the ED. They were also concerned about the anxiety that unnecessarily urgent alert messages could cause.		Most patients perceived that monitoring their weight and blood pressure was important to help manage their heart failure condition.	Security/ privacy In general, patients did not have major security concerns about using the monitoring system as long as reasonable measures were taken to protect the confidentiality of their information.	UNDECLARED
Co-design and prototype development of the 'Ayzot App': A mobile phone based remote monitoring system for palliative care	2023	PALLIATIVE MEDICINE	Carey, N and Abathun, E and Maguire, R and Wodaje, Y and Royce, C and Ayers, N	10.1177/026921632 31162408	To co-design, develop and evaluate a mobile phone based remote monitoring system for use by palliative care patients in Ethiopia	Qualitative	Ethiopia	palliative care patients, family carers	Participants were aged 30–50 years. Patients had a diagnosis of cancer or HIV/AIDS, and healthcare workers 3–15 years palliative care experience.	A mobile phone based remote monitoring system for palliative care			accept		Comfortable for patients and carers to use; simple and easy		User interface design<br>Attractive graphics, use more pictures and less text; would be good to able edit diary notes; can photos be of Ethiopians rather than people with same colour skin<br>Font size – can this be changed/edited so can be changed by user. Some minor language issues that need to be addressed<br>Future consideration Content:<br>Provide information on constipation, side-effects, and drug interactions from medicines, exercise, more spiritual advice, cultural medicine<br>Having a care-giver version<br>Add in more signposting to help patient/carer use the App, navigating back and forwards around different sections, frequency of use<br>More pictures and less text			Lot of benefits – in terms of improving knowledge, provides readily accessible information that supports decision making<br>Good information on spiritual care, wound care, medicines and other symptoms such as pain, nausea<br>Advantages Very helpful – provides information that I did not realise would be helpful to know<br>It contains everything I need to know in a friendly way<br>Good information, makes patients feel knowledgeable,<br>community now asking me for information<br>Have learnt a lot and been able to share that knowledge with my family and community<br>Content is helpful and does work in terms of symptom control<br>Addresses palliative care holistically<br>Helpful self-assessment and diary to monitor symptoms<br>Increases confidence for family members<br>During Covid-19 has been very helpful due to restricted visits<br>Helpful if not able to access hospital or healthcare professionals, for those with smart phones in the city		N
A mixed methods exploratory study of women's relationships with and uses of fertility tracking apps	2018	DIGITAL HEALTH	Gambier-Ross, K and McLernon, DJ and Morgan, HM	10.1177/205520761 8785077	Digital self-tracking is rising, including tracking of menstrual cycles by women using fertility tracking apps (FTAs). However, little is known about users' experiences of FTAs and their relationships with them. The aim of this study was to explore women's uses of and relationships with FTAs.	Mixed	UK	women	We discarded one response so all sample demographics should be out of 240. We have doubled checked and they are all calculated out of 240. The mean age of the respondents was 29 years (range 18–61). The majority of respondents were educated to undergraduate level (n = 111, 46%) and were white (n = 220, 92%). Most of the respondents were from the UK (n = 195, 81%) although the survey received a global response.	fertility tracking apps			Of the 89 respondents who reported using FTAs, 98% find the apps useful.		Tracking helps to be prepared/plan around period Inaccurate (usually reported in women with irregular periods)<br>Informative Upsetting and cause anxiety (usually in women who are having fertility difficulties)<br>Tracking of other function e.g. sleep, mood and see how it correlates to cycle<br>Reminders are annoying<br>Notifications and reminders<br>To maintain a history of cycles<br>Easy to store, access, share data<br>Predicts fertile days, can plan or avoid sex<br>Community provides reassurance	[illegible] use of FTAs were:<br>To observe cycle (n = 63, 72%);<br>To conceive (n = 30, 34%);<br>To inform fertility treatment (n = 10, 12%);<br>As contraception (n = 3, 4%).<br>A total of 11% of respondents reported 'other' reason for use. Some examples of these included: to be prepared for menstruation; to track hormonal acne and mood swings; monitoring successful conception; and trying to see patterns to link to other symptoms. These reasons were not recoded to fit with our options given that participants chose to select other and provide additional text.<br><br>The majority of women reported tracking their cycles so they could share their data with a healthcare professional or to have as a precautionary measure	Recommended changes to FTAs coded into 12 main themes.<br><br>1 Eliminate ads<br>2 Add more mood choices<br>3 Make it easier to log data<br>4 More information (links to current research, relevant forums, fertility information, contraception information, general women's health information)<br>5 Improved accuracy<br>6 Features for someone going through fertility treatment<br>7 Add reminders<br>8 Add note-taking feature<br>9 More specialised recommendations<br>10 Link to other health apps<br>11 Manually adjust data<br>12 Change design Design (content and delivery)	Responses to Q18 'If you do not use FTAs, please explain why' coded into nine themes.<br><br>1 Never knew about them<br>2 On contraception or not sexually active – do not see the need<br>3 Haven't found one to use that I like yet<br>4 Menopausal or sterilised<br>5 Do not trust them<br>6 Use a diary/regular calendar<br>7 Apps are not user friendly/do not like them<br>8 Terrible with technology/do not have phone storage space<br>9 Too stressful/too much pressure (for women trying to get pregnant)	Social recommendations Two women reported that their mother had recommended that they start tracking but not specifically via an app. Two others said that the app Clue was recommended by a friend. One woman came across an app recommended on an online forum and another woman saw Natural Cycles advertised on social media. The remaining women discovered the apps on their own after a recommendation from their doctor to start tracking their cycles. This is evidence of the fact that discussing periods is becoming less of a taboo and women are sharing their tracking practices in their social circles both in person and online.	Education Many women found the apps educational and admitted that this was the first time they have truly understood their own bodies. The use of the apps is allowing women to better understand each stage of their cycle and to find correlations between their cycle and symptoms.	Scepticism The two women trying to conceive expressed the most scepticism about the accuracy and reliability of the apps. A number of respondents reported not sharing their data with anyone (43%, n = 38). Other responses were: share with partner (39%, n = 35); share with family and friends (10%, n = 9); share with healthcare professional (18%, n = 16); share with fertility specialist (10%, n = 9); and share with online community (2%, n = 2).	N

Smart Home Sensing and Monitoring in Households With Dementia: User-Centered Design Approach	2021	JMIR AGING	Tiersen, F and Batey, P and Harrison, MJC and Naar, L and Serban, AI and Daniels, SJC and Calvo, RA	10.2196/27047	This study investigates the functional, psychosocial, and environmental needs of people living with dementia, their caregivers, clinicians, and health and social care service providers toward the design and implementation of smart home systems.	Qualitative	UK	people with Dementia		Smart Home Sensing and Monitoring in Households With Dementia							[illegible]	[illegible]	[illegible]			
Empirical Study Based on the Perceptions of Patients and Relatives about the Acceptance of Wearable Devices to Improve Their Health and Prevent Possible Diseases	2019	MOBILE INFORMATION SYSTEMS	Guillen-Gamez, FD and Mayorga-Fernandez, MJ	10.1155/2019/4731048	The purpose of this study is to analyse the perceptions of the subjects involved in the research regarding the acceptance of wearable devices, taking into account variables, such as age and gender.	Quantitative	Spain	Patients and Relatives	The sample consisted of a total of 606 patients and relatives belonging to a public health centre in the community of Madrid (Spain). The predominant gender of the participants in the study was female (60.2%) compared to 39.8% male. Regarding age, the sample obtained an average of 32 years, with a standard deviation of 10.55. In addition, Table 1 shows the number of devices per gender according to the part of the body where they prefer to wear the wearable device. It is observed that both sexes prefer on the wrist, followed by hanging on clothes.	the Perceptions of Patients and Relatives about the Acceptance of Wearable Devices to Improve Their Health and Prevent Possible Diseases			[illegible]	[illegible]	[illegible]	[illegible]					[illegible]	s.
Attitudes and preferences toward monitoring symptoms, distress, and quality of life in glioma patients and their informal caregivers	2016	SUPPORTIVE CARE IN CANCER	Boele, FW and van Uden-Kraan, CF and Hilverda, K and Reijneveld, JC and Cleijne, W and Klein, M and Verdonck-de Leeuw, IM	10.1007/s00520-016-3112-7	Glioma patients and their informal caregivers face many challenges in living with the disease and its disease specific consequences. To better meet their needs, a system to monitor symptoms, distress, and quality of life could prove useful. We explored glioma patients' and caregivers' attitudes and preferences toward monitoring in general and specifically toward paper-and-pencil and computerized (eFlealth) options.	Qualitative	Netherlands	glioma patients and their informal caregivers	https://www.ncbi.nlm.nih.gov/pmc/articles/PMC4877415/figure/Fig1/	monitoring symptoms, distress, and quality of life in glioma patients and their informal caregivers			Both patients and caregivers thought receiving feedback on the results of monitoring was essential.							[illegible]		s.
Stakeholder垍 experiences of living and caring in technology-rich supported living environments for tenants living with dementia	2023	BMC Geriatrics	Daly-Lynn, J. and Ryan, A. and McCormack, B. and Martin, S.	10.1186/s12877-023-03751-2	The present study examines experiences of people who have dementia and live in technology enriched supported care models. Additionally, it explores caregiver attitudes towards technology use with the housing scheme.	Qualitative	Ireland	people with dementia and caregivers	Participants were not asked specific demographic information in line with recommendations set out for inclusive engagement with people living with dementia in qualitative research [51, 52]. Two interviews were excluded after it became clear the individuals were not living in an environment with technology to support their care. Informal caregivers completed N = 20 surveys and formal caregivers completed N = 31.	technology-rich supported living environments for tenants living with dementia					[illegible]						[illegible]	s.
Clinical utility of a mHealth assisted intervention for activity modulation in chronic pain: The pilot implementation of pain ROADMAP	2023	European Journal of Pain (United Kingdom)	Andrews, N.E. and Ireland, D. and Deen, M. and Varnfield, M.	10.1002/ejp.2104	Being able to successfully self-regulate one's activity levels is a key adaptation strategy for many people with chronic pain. This study aimed to explore the clinical utility of a mobile health platform (Pain ROADMAP) for assisting with the delivery of a tailored activity modulation intervention for people with persistent pain.	Quantitative	Australia	people with chronic pain	The average age for the entire cohort was 46.9 years and slightly more males participated.	mHealth assisted intervention for activity modulation in chronic pain			Overall, participants reported low levels of feeling self-conscious while wearing the Actigraph activity monitor and minimal skin irritation. Generally, participants indicated that they remembered to put the activity monitor on and did not avoid wearing the monitor. A few participants commented that the activity monitor was bulky, uncomfortable to wear at night or interfered with sleep. App notifications to remind people to wear the activity monitor and the ability to wear the monitor on other parts of the body was recommended as way to improve the monitoring.<br><br>Participants largely reported that they found time to enter data into the app, carried the mobile phone with them and remembered to enter their data. A few participants reported that the notification tone was annoying, and that the app notification can be distracting/interfere with driving. Participants made a number of suggestions on how the app could be improved. The majority of these suggestions were minor modification such as customisable notification tones and the ability to edit data once it has been entered. One participant suggested real-time feedback would be helpful, and another recommended an easier to understand user guide.<br><br>All participants specified that the graphs and feedback provided by the clinician was valuable. Eight participants indicated that the feedback was extremely valuable (i.e. rating 6/6) and six participants reported the feedback very valuable (i.e. rating 5/6). In addition, all participant revealed that they would recommend the monitoring to others and that the specific									s.
Real-World User and Clinician Perspective and Experience with MiniMed?780G Advanced Hybrid Closed Loop System	2023	Diabetes Therapy	Kesavadev, J. and Basanth, A. and Krishnan, G. and Shankar, A. and Sanal, G. and Jothydev, S.	10.1007/s13300-023-01427-z	We assessed the performance of the advanced hybrid closed loop (AHCL) system in real-world settings for individuals with type 1 diabetes (T1DM) as well as user and clinician perspectives and satisfaction.	Quantitative	India	adults with T1DM/parents of children and adolescents with T1DM	The users from our center (n = 25; T1DM; 17 female; age 13.8 ± 7.49 years; A1C 6.54 ± 0.45%; duration of diabetes 6 ± 6.78 years; 6 insulin pump naive) were very satisfied with the 780G AHCL system.	MiniMed™ 780G Advanced Hybrid Closed Loop System			The users from our center (n = 25; T1DM; 17 female; age 13.8 ± 7.49 years; A1C 6.54 ± 0.45%; duration of diabetes 6 ± 6.78 years; 6 insulin pump naive) were very satisfied with the 780G AHCL system.		[illegible]					[illegible]		
Use of Cellular-Enabled Remote Patient Monitoring Device for Hypertension Management in Pregnant Women: A Feasibility Study	2023	Maternal and Child Health Journal	D. Jones, R. and Allison, M.K. and Moody, H. and Peng, C. and Eswaran, H.	10.1007/s10995-023-03628-1	This study aimed to measure the feasibility of and patient satisfaction with using an integrated model of cellular-enabled remote patient monitoring devices for blood pressure supported by a 24/7 nurse call center.	Mixed	Unspecified	Hypertension Pregnant Women	11 participants are reported in Table 1. Most participants were white (72.7%), non-Hispanic (100%), married (63.6%), had attended at least some college or technical school (63.6%), and reported an annual household income between $25,000 – $75,000 (63.6%). One out of four (27%) participants reported that this was their first pregnancy. Most participants had one or more children (81.8%). The majority of participants had previously delivered pre-term (62.50%) and reported this to be their first pregnancy with BP issues or preeclampsia (87.50%).	Cellular-Enabled Remote Patient Monitoring Device for Hypertension Management in Pregnant Women			The average score was 75.91 (SD = 23.70), which suggests high participant satisfaction with the device.							[illegible]	[illegible]	
Stepping Beyond Efficacy: Understanding the User Experience of Wearables for Children with Idiopathic Toe Walking in the Natural Setting	2023	Human Factors in Healthcare	Boyd, L. and Soangra, R. and Mukhar, L. and Kling, R. and Grant Beuttler, M.	10.1016/j.hfh.2023.100044	Idiopathic Toe Walking is an atypical gait pattern that results in limited mobility, pain, and higher risk of falling. Current therapeutic interventions lack the ability to be implemented outside the clinic or lab. Beyond this, it is unclear if gait analysis and real-time feedback is technologically feasible to attain within a user's natural environment. In our research we aimed to understand the child and parent participants' experience of an innovative wearable system.	Qualitative	USA	Children with Idiopathic Toe	Eight surveys were from parents of children 7-13 years old and six from children ages 8-13 years at the time of survey collection.	Understanding the User Experience of Wearables for Children with Idiopathic Toe Walking in the Natural Setting			The survey results indicated that the Smart Stepper system was identified as a preferred method of intervention because it was easier to implement than other alternatives. Multiple parents commented on the benefits of the shoe insert compared to casting or orthoses with one parent stating, "Shoe inserts are better as it has built in vibration that reminds my son not to toe walk." [illegible]		[illegible]	[illegible]	[illegible]			[illegible]		[illegible]
Flash glucose monitoring in young people with type 1 diabetes - A qualitative study of young people, parents and health professionals: ? € It makes life much easier'	2023	BMJ Open	Beasant, L. and Cullen, F. and Thomas, E. and Kandiyali, R. and Shield, J.P.H. and McGregor, D. and West, N. and Ingram, J.	10.1136/bmjopen-2022-070477	Flash glucose monitoring for patients with T1 diabetes avoids frequent painful finger-prick testing, thus potentially improving frequency of glucose self-monitoring. Our study aimed to explore experiences of young people using Freestyle Libre sensors and their parents, and to identify benefits and challenges to National Health Service (NHS) staff of its adoption in their care provision.	Qualitative	UK	diabetes young people and parents	Children and young people interviewed n=10 Parents interviewed n=14 from 12 families<br>Age range 8–17 (average 14 years)<br>Child's age range 6–17 (average 12 years)<br>Gender 5 Female, 4 males, 1 prefer not to say Parent gender 11 Mothers/female carers, 3 fathers<br>Ethnic group 9 White, 1 white/black Caribbean Ethnic group 14 White	Flash glucose monitoring in young people with type 1 diabetes			Overall young people wanted to keep using flash monitoring sensors and parents felt positive about the future for their child because technology (including CGM and insulin pumps) was always evolving and getting better (quote 2.4.3).				[illegible]	[illegible]		[illegible]		
Exploring Views and Expectations of Clinicians, Nurses, and Kidney Transplant Recipients Regarding Protocol Development of a Study to Investigate Feasibility of the Implantable Doppler Probe in Kidney Transplantation: Patient-Public Involvement Consultations	2023	Experimental and Clinical Transplantation	Malik, M.S. and Abideen, Z.-U. and Akoh, J.A. and Houlberg, K.	10.6002/ect.2023.0025	The shortage of donor organs is the most limiting factor in kidney transplant practice today. New monitoring technologies are being investigated to reduce graft loss due to vascular complications. We assessed the feasibility of a novel blood flow monitoring device, the implantable Doppler probe, in kidney transplant surgery.	Qualitative	Unspecified	Kidney Transplant Recipients	4 kidney transplant patients.	a novel blood flow monitoring device, the implantable Doppler probe,<br>in kidney transplant surgery.			The first subtheme was positive perceptions of the monitoring device, which included categories such as benefits to the NHS, health care professionals, and kidney transplant patients. The participants felt that the use of the device would have a positive effect on the NHS Trust. The participants felt that the device may have the potential to improve patient and graft outcome, by the early identification of vascular complications. It also decreased the surgery-related anxiety of patients.				[illegible]	[illegible]				s.

Assessing the feasibility of a web-based outcome measurement system in child and adolescent mental health services ?myHealthE a randomised controlled feasibility pilot study	2023	Child and Adolescent Mental Health	Morris, A.C. and Ibrahim, Z. and Heslin, M. and Moghraby, O.S. and Stringaris, A. and Grant, I.M. and Zalewski, L. and Pritchard, M. and Stewart, R. and Hotopf, M. and Pickles, A. and Dobson, R.J.B. and Simonoff, E. and Downs, J.	10.1111/camh.12571	Interest in internet-based patient reported outcome measure (PROM) collection is increasing. The NHS myHealthE (MHE) web-based monitoring system was developed to address the limitations of paper-based PROM completion. MHE provides a simple and secure way for families accessing Child and Adolescent Mental Health Services to report clinical information and track their child's progress. This study aimed to assess whether MHE improves the completion of the Strengths and Difficulties Questionnaire (SDQ) compared with paper collection. Secondary objectives were to explore caregiver satisfaction and application acceptability.	Quantitative	UK	caregiver	Male 74 (75.5) 74 (75.5)<br>Mean age at trial start (SD) 14.3 (2.7) 14.3 (2.8)<br>Ethnicity, n (%)<br>White 39 (39.8) 46 (46.9)<br>Black 35 (35.7) 23 (23.5)<br>Asian 2 (2.0) 1 (1.0)<br>Mixed 13 (13.3) 15 (15.3)<br>Other or not stated 9 (9.2) 13 (13.3)<br>Level of deprivation, n (%)<br>1st (least deprived) 22 (23.2) 25 (25.8)<br>2nd 21 (22.1) 27 (27.8)<br>3rd 24 (25.2) 24 (24.7)<br>4th (most deprived) 28 (29.5) 21 (21.7)<br>Co-morbid diagnosis, n (%)<br>ADHD 48 (49.0) 39 (39.8)<br>LD 14 (14.3) 11 (11.2)<br>Emotional disorder 14 (14.3) 17 (17.4)<br>Mean CGAS score (SD) 53.1 (10.7) 54.6 (8.8)<br>Mean days of active care (SD) 592.1 (196.4) 563.0 (210.4)<br>Mean attended F2F events (SD) 6.0	a web-based outcome measurement system in child and adolescent mental health services			A total of eight SUS questionnaires and usability interviews were completed. The mean SUS score for users of the website was 78/100 indicating that the application was 'acceptable' to users.				Practicalities of safe electronic data collection which is useful, easy and quick; simplicity of use depends on existing computer skills, needs ask their child for help; self management-visualization feedback improved symptom knowledge; want information to be reviewed by clinicians and symptom deterioriation to be prioritised; platform engagement issues-not suitable at discharge or following negative treatment experiences; want support service information specific to their community to be included; want accessible versions of MHE developed						The authors give thanks to the families and Kaleidoscope staff who participated in this trial and the MHE digital development team – Digital Marmalade (see https://www.digitalmarmalade.co.uk/) – with particular thanks to Andy McEniry and Jeremy Jones. J.D conceived the trial aims, supervised data analysis and writing. A.C.M led on data analysis and manuscript writing. M.P. assisted with study design and data acquisition. All authors reviewed and provided critical revisions to the manuscript and approved the final version of the manuscript. The remaining authors declare that they have no competing or potential conflicts of interest.
User perspectives on long-term remote active electronic self-monitoring of mood symptoms in bipolar spectrum disorders	2023	Journal of Affective Disorders	Gordon-Smith, K. and Saunders, K.E.A. and Morton, T. and Savage, J. and South, M. and Geddes, J. and Craddock, N. and Jones, I. and Jones, L.	10.1016/j.jad.2022.12.090	User feedback is crucial in the development of electronic self-monitoring tools for bipolar spectrum disorders (BSD). Previous studies have examined user experiences in small samples self-monitoring over relatively short time periods. We aimed to explore the experiences of a large sample of individuals with BSD engaged in long-term remote active electronic self-monitoring.	Quantitative	UK	patient with bipolar disorder	complete the survey, 362 (62.7 %) responded. The sample of 362 individuals is described in Table 1. 67.4 % were female (in all cases sex at birth matched gender identity) with a median age of 54 years. 62.1 % had completed higher education. The majority had a DSM-IV diagnosis of bipolar I disorder (56.1 %) or bipolar II disorder (39.0 %), with the remaining minority having schizoaffective disorder bipolar type (1.4 %) or bipolar disorder not otherwise specified (3.6 %). Median age of onset of BSD was 19 years and the median lifetime number of episodes of depression and (hypo)mania was 9 and 6 respectively. 63.8 % had at least one past psychiatric admission and 50.8 % had a lifetime history of suicide attempt. The median length of time individuals had been using True Colours was 28 months (range 1–36 months). 44.5 % had chosen to add additional personalised questions	long-term remote active electronic self-monitoring of mood symptoms in bipolar spectrum disorders			88.4 % of survey respondents (320/362) reported that they found using True Colours extremely or moderately helpful (40.6 % and 47.8 % respectively) (Fig. 1A). 87.8 % (318/362) reported that they had looked at their True Colours mood graphs, and of these 86.4 % (275/318) reported finding looking at their graphs extremely or moderately helpful (44.3 % and 42.1 % respectively) (Fig. 1B).			One third had shared their graphs, with 89.9 % finding it helpful to share with HCPs and 78.7 % helpful to share with family/friends.				The most commonly reported perceived benefits included having a visual record of mood changes, patterns and/or triggers (50.6 %) and helping to identify early warning signs (13.0 %). 12.6 % commented that completing their True Colours questionnaires each week encouraged them to reflect on their mood on a regular basis which they found helpful. Some individuals also made additional generic broad positive comments about True Colours for example how they felt it would be helpful for others to use also (7.5 %) and 6.7 % specifically commented that a key motivation for using True Colours was helping with research. Less commonly reported perceived benefits included True Colours helping individuals recognise aspects of their mood symptoms they were previously less aware of (5.4 %), being reassuring by showing improvement in mood (4.2 %), confirming individual's own subjective impression of their mood (3.3 %) and individuals finding it reassuring to receive the True Colours emails every week (1.7 %).	The most commonly reported perceived limitation of True Colours was the questions not being finely-tuned or comprehensive enough (30.1 %). Individuals also reported a limitation as being that their True Colours graphs did not reveal anything new (13.0 %), in a number of cases this was because individuals reported their mood had been stable over the duration of time they had been using True Colours. 8.4 % reported experiencing technical/usage problems and 4.6 % mentioned the presentation of the True Colours graphs was not immediately clear and/or that they had experienced difficulties understanding the graphs. A very small proportion of individuals reported that completing weekly mood ratings reminded them of being unwell (1.7 %) and 1.3 % reported that they would not complete True Colours when they were unwell.	N
Barriers to and Facilitators of Using Remote Measurement Technology in the Long-Term Monitoring of Individuals With ADHD: Interview Study	2023	JMIR Formative Research	Denyer, H. and Deng, Q. and Adanijo, A. and Asherson, P. and Bilbow, A. and Folarin, A. and Groom, M.J. and Hollis, C. and Wykes, T. and Dobson, R.J.B. and Kuntsi, J. and Simblett, S.	10.2196/44126	Remote measurement technology (RMT) has the potential to address current research and clinical challenges of attention-deficit/hyperactivity disorder (ADHD) symptoms and its co-occurring mental health problems. We aimed to evaluate the barriers to and facilitators of using RMT in individuals with ADHD compared with a group of people who did not have a diagnosis of ADHD. We also aimed to explore participants' views on using RMT for 1 or 2 years in future studies.	Qualitative	UK	Individuals With ADHD	ADHDa Remote Technology pilot study Debrief interviews<br><br>ADHD group (n=20) Comparison group (n=20) ADHD group (n=10) Comparison group (n=12)<br>Gender (female), n (%) 15 (75) 15 (75) 8 (80) 11 (91)<br>Age (years), mean (SD) 27.49 (6.04) 27.79 (6.17) 28.03 (6.36) 27.75 (6.74)<br>Ethnicity, n (%)<br><br>Asian 1 (5) 3 (15) 0 (0) 3 (25)<br><br>Black 1 (5) 0 (0) 1 (10) 0 (0)<br><br>White 16 (80) 17 (85) 9 (90) 9 (75)<br><br>Mixed or multiple ethnic groups 2 (10) 0 (0) 0 (0) 0 (0)	Long-Term Monitoring of Individuals With ADHD								Barriers to and facilitators of using RMT were categorized as health-related, user-related, and technology-related factors across both participant groups. When comparing themes that emerged across the participant groups, both individuals with and without ADHD experienced similar barriers and facilitators in using RMT. The participants agreed that RMT can provide useful objective data. However, slight differences between the participant groups were identified as barriers to RMT across all major themes. Individuals with ADHD described the impact that their ADHD symptoms had on participating (health-related theme), commented on the perceived cost of completing the cognitive tasks (user-related theme), and described more technical challenges (technology-related theme) than individuals without ADHD. Hypothetical views on future studies using RMT in individuals with ADHD for 1 or 2 years were positive.				JK has given talks at educational events sponsored by Medice; all funds are received by King's College London and used for studies of attention-deficit/hyperactivity disorder (ADHD). PA has received funding for research by Vifor Pharma, has given sponsored talks, and been an advisor for Shire, Janssen-Cilag, Eli-Lilly, Flynn Pharma, and Pfizer regarding the diagnosis and treatment of ADHD; all funds are received by King's College London and used for studies of ADHD. The other authors report no conflicts of interest.
Passive collection of geolocation data by older people with cognitive impairment: Feasibility and user experiences for use in research	2023	Gerontechnology	Liddle, J. and Ireland, D. and Krysinska, K. and Lamont, R. and Kang, K. and Reppermund, S. and Sachdev, P.S. and Gustafsson, L. and Brauer, S. and Pachana, N.A. and Brodaty, H.	10.4017/GT.2023.22.1.811.05	To explore the feasibility, user experiences, and processes needed to support the smartphone-based collection of older people with cognitive impairment.	Mixed	Australia	Older people with cognitive impairment	Eighteen participants with mild cognitive impairment (n=15) or probable dementia (n=3) (10 male, age M=82.86 years, SD = 3.74) collected geolocation data. The level and type of recorded cognitive impairment at the closest data collection to their participation in the substudy are as follows (probable dementia 3 (16.7%); Nonamnestic single domain MCI 6 (33.3%); Amnestic multiple domain MCI 4 (22.2%); Nonamnestic multiple domain MCI 1 (5.6%) Nonamnestic MCI without subjective complaints 1 (5.6%))<br>Participants collected between 105 and 240 hours of geolocation data (M = 161.5 hours, SD = 31.7).	Passive collection of geolocation data by older people with cognitive impairment			All participants were able to participate in choosing locations and a case during the setup, expressing preferences and discussing how the data collection would fit in with their daily life. Some participants had limited prior technology experience but had volunteered for a study using technology. Some participants indicated that they found the heatmaps generated from their data and being involved in the research to be interesting.		noted that just carrying the phone was preferable to be asked to write down their travel patterns or remember them in an interview. although one participant found the charging connecter (micro-USB) difficult to orientate and plug in and two worried about the potential damage to the phone by taking it out of the house to community locations (like the pub) and required reassurance and confirmation of how to use the phone.			"stuffed up" the phone. …Found the phone appeared to have simply been turned off. Apparently, the screen had gone to sleep not long after I had left, and she thought it meant the phone wasn't working" [Field notes – Participant E] This was generally resolved with a repeat explanation or troubleshooting. During the data collection, two participants expressed concerns in relation to messages received on the phone (operating system update requests). One accidentally turned off the phone Figure 2. Example Heatmaps of lifespace data (the location of data collected has been shifted to preserve anonymity of participants) One had concerns about the cost of charging the phone for the week of usage and another asked to repeat data collection once they became used to charging and carrying the phone. One participant reported family members had chastised her for participating as she "is no good at technology" (Participant D). Only one participant had incomplete data due to opting out. She turned off and packed up the beacons due to (unrelated) alarms from other devices in her house after a power outage				PS is an Advisory committee member for Biogen Australia. HB is an advisory board member or consultant for Biogen, Nutricia, Roche and Skin2Neuron. The other authors declare they have no conflicts of interests
What Makes Consumers Adopt a Wearable Fitness Device? The Roles of Cognitive, Affective, and Motivational Factors	2023	International Journal of e-Business Research	Zhang, J. and Mao, E.	10.4018/IJEBR.323204	Wearable fitness devices are equipped with internet connectivity and capable of tracking, storing, and transmitting health data. An online survey was conducted among 297 non-wearable-users in the U.S. to collect data. Structural equation modeling was used to test the intention model.	Quantitative	USA	General public	range from 19 to 71. The average age of our sample is 30. The gender was relatively evenly distributed (female: 154; 51.85%). EthnicityAsian / Pacific Islander 123 41.41%White or Caucasian 108 36.36%Hispanic or Latino 35 11.78%Black or African American 15 5.05%Multicultural 12 4.04%Other 4 1.35% Personal Income (before taxes)Less than $10,000 104 35.02%$10,000 - $19,999 56 18.86%$20,000 - $29,999 45 15.15%$30,000 - $39,999 34 11.45%$40,000 - $49,999 15 5.05%$50,000 - $74,999 23 7.74%$75,000 - $99,999 10 3.37%$100,000 or more 10 3.37	Wearable Fitness Device				The results showed that three factors—cognitive, affective, and motivational—emerged as key determinants of consumers' intention to adopt wearables, with affective factors showing the most explanatory power. The role of the price factor was also revealed									UNDECLARED
Personal Health Data Tracking by Blind and Low-Vision People: Survey Study	2023	Journal of Medical Internet Research	Lee, J.G.W. and Lee, K. and Lee, B. and Choi, S. and Seo, J. and Choe, E.K.	10.2196/43917	Personal health technologies, including wearable tracking devices and mobile apps, have great potential to equip the general population with the ability to monitor and manage their health. However, being designed for sighted people, much of their functionality is largely inaccessible to the blind and low-vision (BLV) population, threatening the equitable access to personal health data (PHD) and health care services. This study aims to understand why and how BLV people collect and use their PHD and the obstacles they face in doing so.	Mixed	UK	Blind and Low-Vision People	who self-identified as totally blind (TB; 79/156, 50.6%) or LV (77/156, 49.4%) participated in our survey.<br>Approximately two-thirds of them were CT (102/156, 65.4%), 19.2% (30/156) were PT, and 15.4% (24/156) had NE tracking PHD. More than half of the respondents were visually impaired since birth (83/156, 53.2%), and approximately one-third became visually impaired after birth and had been visually impaired for at least 10 years (51/156, 32.7%). Most respondents (151/156, 96.8%) rated their health condition as fair or better.<br>Respondents varied in age, with 22.4% (35/156) aged 35 to 44 years and 21.2% (33/156) aged 55 to 64 years; 68.6% (107/156) were female, 29.5% (46/156) were male, and 1.9% (3/156) were nonbinary. More than two-thirds of the respondents were White (107/156, 68.6%), 10.9% (17/156) were Asian, and 5.8% (9/156) were Black or African	Personal Health Data Tracking by Blind and Low-Vision People						To sustain healthy status<br>To prevent health deterioration<br>To be aware of family- or aging-related issues<br>A means to improve oneself<br>Enabling general health improvement, especially weight loss<br>Activity as a step toward external goals, for example, losing weight so that one can try new activities<br>Learning from system-provided health coaching<br>Being inspired by encouragement given by system<br>Way to learn about themselves<br>Diagnostic tool for health, for example, blood sugar monitoring<br>Allowing a comparison of PHD with system's global averages<br>Tracking personal health activity progress<br>Mere curiosity<br>Outcome analysis<br>Inspecting correlations of actions toward health<br>Measuring the effectiveness of medications and treatments<br>Using systems as a historical record keeper<br>With and for others	Exercise (115/132, 87.1%) and weight (101/132, 76.5%) were the 2 most popular items the CT and PT respondents reported tracking (Figure 1). Other highly ranked items were sleep (63/132, 47.7%), heart rate (61/132, 46.2%), food (60/132, 45.5%), water (58/132, 43.9%), and blood pressure (55/132, 41.7%).	Blocked from initiating tracking<br>No accessible options for tracking certain activities<br>Visual roadblocks prevent users from initiating tracking, for example, inaccessible device pairing codes<br>Lacking information on accessibility of available apps and devices<br>Accessible equipment can be unaffordable for the population<br>Adaptation tools not supported by systems<br>Incompatibility with tools, for example, screen reader and braille output<br>Lack of visibility options, for example, magnification or contrast<br>Misapplied aid, for example, screen reader compatible but giving incorrect label for button<br>Missing out on features because of disability<br>Limited from features on devices because of lack of accessibility, for example, screens on wearables<br>Inaccessibility when graphs are used to portray data in apps<br>Specific app features not enabled with accessibility<br>Information delay<br>Waiting for data to be transferred to accessible app<br>Waiting for aid of others in reading data<br>Negative awareness of sighted users' experiences<br>Measuring time expenditure against sighted users' efforts<br>Jealousy in how others benefit from features inaccessible to them<br>Comparison of prior experience to current use with declined vision				N
Health-Seeking Behavior and Its Associated Technology Use: Interview Study among Community-Dwelling Older Adults	2023	JMIR Aging	Zhang, Y. and Lee, E.W.J. and Teo, W.-P.	10.2196/43709	Understanding older people's health-seeking behavior (HSB) is crucial for uncovering their health needs and priorities and developing appropriate policies to address them and avert their disease progression. This study aimed to investigate HSB and the associated technology use among the older population, ultimately proposing implications for practice to address their unmet health needs.	Qualitative	Singapore	Older Adults	The 15 participants were aged between 55 and 73 (mean 65.8, SD 6) years, among whom 9 (60%) were women, 13 (87%) were Chinese, 6 (40%) were still working, and 8 (53%) had a personal monthly income of <SG $1000 (<US $752). All participants (15/15, 100%) owned a personal smartphone, but 13% (2/15) of them did not have Wi-Fi access at home.	digital technologies			most widely used digital technologies are the mobile health apps and wearable devices with the associated wellness programs launched by the government and local companie<br><br>Conclusions<br>Unlike the commonly held belief that older adults resist technologies and lack technological proficiency, our findings showed that technologies could play a promising role in facilitating older adults' health seeking. Our findings have implications for the design and implementation of health services and policies.							, and they have the potential to enhance health communication, promote health maintenance, and increase access to health services; information communication technologies and social media, though not primarily designed for health purposes, play a substantial role in easing the process of seeking health information and managing symptoms.		N
Integration of the Vision of People With Diabetes Into the Development Process to Improve Self-management via Diabetes Apps: Qualitative Interview Study	2023	JMIR Diabetes	Klemme, I. and Wrona, K.J. and De Jong, I.M. and Dockweiler, C. and Aschentrup, L. and Albrecht, J.	10.2196/38474	This study aims to contribute to the technology development process of diabetes apps by exploring individual perspectives on desired features and content of diabetes apps among people with diabetes.	Qualitative	Germany	Diabetes adults	age: 25-78 6M 10F	Diabetes Apps							to improve their QoL and allow them to live as comfortably as possible, such as informative predictions through artificial intelligence, improvements in signal loss and value delay through smartwatches, improved communication and information-sharing capabilities, reliable information sources, and user-friendly and discreet messaging options through smartwatches. In addition, according to people with diabetes, future apps should show improved sensors and app connectivity to avoid incorrect values being displayed. They also wish for an explicit indication that displayed values are delayed. In addition, personalized information was found to be lacking in apps.					N
Toward Diabetes Device Development That Is Mindful to the Needs of Young People Living With Type 1 Diabetes: A Data- and Theory-Driven Qualitative Study	2023	JMIR Diabetes	Brew-Sam, N. and Parkinson, A. and Chhabra, M. and Henschke, A. and Brown, E. and Pedley, L. and Pedley, E. and Hannan, K. and Brown, K. and Wright, K. and Phillips, C. and Tricoli, A. and Nolan, C.J. and Suominen, H. and Desborough, J.	10.2196/43377	This study aimed to examine young people's and their caregivers' experiences with diabetes technologies in an exploratory way and relate the findings to the existing technology acceptance and technology design theories.	Qualitative	Australia	Diabetes young people	with T1DM (female: n=7, 44%; male: n=9, 56%) aged between 12 and 17 years and accompanied by a parent (mother: n=11, 69%; father: n=4, 25%; both: n=1, 6%). We focused on this age group because of 3 reasons. First, government subsidies are limited to those aged <21 years [34]. Second, younger individuals have been reported to be "more exposed to new technologies and easier to absorb the new technological advancements with minimum effort" [35]. Third, adolescence—with a transition from childhood to adulthood—is accompanied by general life challenges affecting diabetes management [36,37].	Diabetes Device											Privacy concerns or related aspects were not reported throughout the interviews, and none of the participants made any mention of data privacy. reality deviated from these expectations, with inaccuracy problems reported in continuous glucose monitoring devices and technical failures occurring in both continuous glucose monitoring devices and insulin pumps.	N
Feasibility and Acceptability of an Internet of Things[illegible]nabled Sedentary Behavior Intervention: Mixed Methods Study	2023	Journal of Medical Internet Research	Huang, Y. and Benford, S. and Li, B. and Price, D. and Blake, H.	10.2196/43502	Encouraging office workers to break up prolonged sedentary behavior (SB) at work with regular microbreaks can be beneficial yet challenging. We previously developed an IoT-enabled SB intervention, called WorkMyWay, by applying a combination of theory-informed and human-centered design approaches. This study aims to evaluate the feasibility and acceptability of the WorkMyWay intervention and its technological delivery system.	Mixed	UK	Employee	Characteristic Value<br>Age (years), mean (SD; range) 40.5 (11.0; 25-63)<br>Gender, n (%)<br>Male 3 (20)<br>Female 12 (80)<br>Highest education level completed, n (%)<br>University preparatory degree 2 (13)<br>Undergraduate degree 6 (40)<br>Postgraduate degree 7 (47)Self-reported occupational time spent (hours), mean (SD; range)<br>Sitting 6.2 (1.5; 2.4-8.2)<br>Standing 0.9 (1.3; 0-4.8)<br>Walking 0.8 (0.6; 0.145-2)<br>HEavy labor 0.1 (0.5; 0-1.9)<br>Total 8.0 (0.9; 7.25-10)<br>Height (cm), mean (SD; range) 169.3 (7.5; 155-180)<br>Weight (kg), mean (SD; range) 72.0 (13.6; 49-90)<br>BMI (kg/m2), mean (SD; range) 25.0 (4.1; 18.4-33.0)<br>Underweight (≤18.5), n (%) 1 (7)	an Internet of Things–Enabled Sedentary Behavior Intervention					The qualitative analysis identified 6 themes, which lent support to the high acceptability of WorkMyWay, though delivery was compromised by issues concerning Bluetooth connectivity and factors related to user behaviors.<br><br>Conclusions<br>It is acceptable and feasible to deliver an SB intervention with an IoT system that involves a wearable activity tracking device, an app, and a digitally augmented everyday object (eg, cup). More industrial design and technological development work on WorkMyWay is warranted to improve delivery.		Fixing technical issues, tailoring to individual differences, soliciting organizational supports, and harnessing interpersonal influences could facilitate delivery and enhance acceptance.			Although no significant change was observed in either objective or self-report OSPA, postintervention improvements were significant in the automaticity of regular break behaviors (t14=2.606; P=.02), retrospective memory of breaks (t14=7.926; P<.001), and prospective memory of breaks (t14=–2.661; P=.02).		N
Using a Proximity-Detection Technology to Nudge for Physical Distancing in a Swedish Workplace during the COVID-19 Pandemic: Retrospective Case Study	2022	JMIR Formative Research	Zetterholm, M.V. and Nilsson, L. and Jokela, P.	10.2196/39570	The recent COVID-19 pandemic has contributed to the emergence of several technologies for infectious disease management. Although much focus has been placed on contact-tracing apps, another promising new tactic is proximity tracing, which focuses on health-related behavior and can be used for primary prevention. We aimed to evaluate the feasibility of using a wearable device to nudge for distance and to gather important insights about how functionality and interaction are experienced by users.	Mixed	Sweden	Employee	The age distribution of the participants was between 28 and 68 (mean 46.2, SD 1.96) years.	Using a Proximity-Detection Technology to Nudge for Physical Distancing in a Swedish Workplace During the COVID-19 Pandemic			Some participants were curious about the technology, while others were skeptical. :<br><br>People were a bit unprepared, but acceptance grew over time.	The descriptive statistics show that after the production project, there were no negative responses, the number of neutral responses decreased, and both skeptical and positive responses increased. The median value before and after distribution was quite positive (rating 4). The difference between attitudes before and after the intervention was not statistically significant (W=108; P=.80), so the null hypothesis cannot be rejected.	preventive strategy. The connection between the distance tags and the preventive context was a key issue for their feasibility. This holistic approach involved a combination of measures. For instance, face masks were used in situations when distance could not be maintained. In addition to face masks, other preventive measures included handwashing stations, fever scanning at the entrance, and the formation of social bubbles. The latter meant that people working closely together at the event also shared hygiene facilities. The interviewees further described that the combination of distance tags, personal protective equipment, handwashing stations, effective communication, and people overseeing the adherence to these measures was necessary for a successful preventive strategy:<br>Of course, there are occasions when it won't work, in this kind of production. In these cases, we have other measures.<br><br>The interviewees emphasized the importance of the event's venue as being sufficiently spacious for individuals to maintain physical distance. One of the key aspects for the successful use of distance tags is that the tags fit in the physical environment. They are particularly useful in places and situations where people meet and tend to stand close to each other or where people's paths cross, for example, by coffee machines. Manual adjustments regarding proximity detection to fit the conditions of the event could be made, but there were situations in which the distance tags were not deemed useful. Some survey respondents commented that the tags were impractical or of no use during work tasks that required close interaction:	Several survey respondents noted that their participation was motivated by a desire to prevent infection in themselves and others	Several interviewees described that the device had a high-pitched noise, which caused irritation and frustration, especially in situations in which individuals were prevented from maintaining distance or in situations where the noise caused disruption The interviewees and survey respondents agreed that the key function is the immediate warning provided by the distance tag. Some were interested in additional functions, such as data on the number of close interactions, or the use of these devices for contact tracing. Others wanted a battery charge indicator related to the previously mentioned complaints regarding the short battery time. Less than half of the survey respondents were interested in additional functions or in using an app connected to the device (12/30, 40% of respondents). However, attitudes differed and most clearly rejected those ideas (14/30, 46% of respondents) or were hesitant to add more features (3/30, 10% of respondents):			There were also indications that this technology has the potential to facilitate habitual changes. Several interviewees stated that they are better or much better at maintaining their distance now, even when they were not wearing their devices:When the distance tags were first implemented, managerial staff had varying expectations. Some were skeptical and others were not sure what to expect or how the system would work. Some participants also expressed interest and curiosity. After a few weeks of using the distance tags, the general perception of the interviewees and most survey respondents was that the tags had been successful and fulfilled their purpose. The others were not initially assured that this type of device was needed. Several interviewees said that not everyone considered keeping distance to be a problem; that is, they thought that they were already good at maintaining sufficient distance from others. However, using proximity technology to continuously monitor behavior made it clear that this was not the case. Several interviewees described that the device helped them be attentive to distance and taught them how much distance they needed to maintain:		N

Pregnancy outcomes in infertility patients diagnosed with sleep-disordered breathing with wireless wearable sensors	2022	Sleep Medicine	Walter, J.R. and Lee, J.Y. and Snell, B. and Park, J.B. and Kim, D.H. and Xu, S. and Benthan, K.	10.1016/j.sleep.2022.09.026	To study the feasibility of home-based assessment of sleep disordered breathing (SDB) on early pregnancy success after in vitro fertilization with novel wearable sensors.	Quantitative	USA	infertility patients	[illegible]	wireless wearable sensors			The sensor system scored favorably among users with the average SUS score reported as 75.4 (SD 14). A score over 68 is considered above average.								[illegible]
A qualitative study of the acceptability of remote electronic bednet use monitoring in Uganda	2022	BMC Public Health	Alexander, S.M. and Agaba, A. and Campbell, J.I. and Nambogo, N. and Conklin, C.S. and [illegible]	10.1186/s12889-022-13393-5	Distribution of long-lasting insecticide treated nets (LLINs) is the most widely used intervention for the prevention of malaria [illegible] SmartNet in Ugandan households.	Qualitative	Uganda	General public	[illegible]	remote electronic bednet use monitoring					[illegible]	[illegible]	[illegible]	[illegible]	[illegible]	[illegible]	[illegible]
Wearable device use and technology preferences in cancer survivors with or at risk for atrial fibrillation	2022	Cardiovascular Digital Health Journal	Faro, J.M. and Yue, K.-L. and Singh, A. and Soni, A. and Ding, E.Y. and Shi, Q. and McManus, D.D.	10.1016/j.cvdhj.2022.09.002	Cancer survivors face increased risk of heart disease, including atrial fibrillation (AF). [illegible]	Quantitative	USA	cancer survivors with or at risk for atrial fibrillation	The study cohort of 424 cancer survivors [illegible]	Wearable devices in cancer survivors with or at risk for atrial fibrillation				[illegible]		[illegible]			[illegible]		[illegible]
The Socioeconomic Indicators Linked to Parent Health-Related Technology Use: Cross-sectional Survey	2022	Journal of Medical Internet Research	McCall, M.P. and [illegible] and Highlander, A. and Jones, D.J.	10.2196/37455	This study aimed to explore parental health-related technology use (HTU) [illegible]	Quantitative	USA	children and parent	[illegible]	a range of hardware devices [illegible]				[illegible]							
Risks of Mobile Ambient Sensors and User Awareness, Concerns, and Preferences	2022	ACM International Conference Proceeding Series	Mehrnezhad, M. and Makarounas, C. and Gray, D.	10.1145/3549015.3554171	We run an online user study (N=197) and evaluate user awareness, concerns, and preferences for mobile ambient sensors when accessed via apps and websites.	Mixed	UK	General public	197 participants completed our questionnaire. [illegible]	Mobile Ambient Sensors	[illegible]					[illegible]				[illegible]	UNDECLARED
A head-to-head comparison between Guardian Connect and FreeStyle Libre systems and an evaluation of user acceptability of sensors in patients with type 1 diabetes	2022	Diabetes/Metabolism Research and Reviews	Yeoh, E. and Png, D. and Khoo, J. and Chen, Y.Y. and Sharda, P. and Low, S. and Lim, S.C. and Subramaniam, T.	10.1002/dmrr.3560	A user-calibrated real-time continuous glucose monitoring (rt-CGM) system is compared to a factory-calibrated flash glucose monitoring (FGM) system and assessed in terms of accuracy and acceptability in patients with type 1 diabetes (T1D).	Quantitative	Singapore	Diabetes patients	[illegible]	sensors in patients with type 1 diabetes					[illegible]		[illegible]		[illegible]		[illegible]
Monitoring of rheumatoid arthritis: a patient survey on disease insight and possible added value of an innovative inflammation monitoring device	2022	Rheumatology International	[illegible]	10.1007/s00296-021-05026-9	To enable patients with rheumatoid arthritis (RA) [illegible]	Quantitative	Netherlands	rheumatoid arthritis patient	[illegible]	Monitoring of rheumatoid arthritis by an innovative inflammation monitoring device.						[illegible]	[illegible]		[illegible]	[illegible]	[illegible]
Analyzing technology acceptance and perception of privacy in ambient assisted living for using sensor-based technologies	2022	PLoS ONE	Wilkowska, W. and Offermann, J. and Spinsante, S. and Poli, A. and Ziefle, M.	10.1371/journal.pone.0269642	This paper empirically investigates users' technology acceptance and privacy perceptions related to sensor-based applications implemented in private environments [illegible]	Quantitative	Germany	General public	[illegible]	ambient assisted living for using sensor-based technologies			[illegible]	[illegible]	[illegible]		[illegible]		[illegible]	[illegible]	[illegible]
Real-World User Experiences with a Digital Pill System to Measure PrEP Adherence: Perspectives from MSM with Substance Use	2022	AIDS and Behavior	Chai, P.R. and Goodman, G.R. and Bronzi, O. and Gonzales, G. and Baez, A. and Bustamante, M.J. and Najarro, J. and Mohamed, Y. and Sullivan, M.C. and Mayer, K.H. and Boyer, E.W. and O'Cleirigh, C. and Rosen, R.K.	10.1007/s10461-022-03594-9	Once daily oral pre-exposure prophylaxis (PrEP) is highly effective for preventing HIV transmission [illegible]	Qualitative	USA	men who have sex with men (MSM) who use substances.	[illegible]	Digital Pill System to Measure PrEP Adherence			[illegible]		[illegible]	[illegible]	[illegible]				[illegible]
Improving the user experience of televisits and telemonitoring for heart failure patients in less than 6 months: a methodological approach	2022	International Journal of Medical Informatics	Savoldelli, A. and Vitali, A. and Remuzzi, A. and Giudici, V.	10.1016/j.ijmedinf.2022.104717	The research work presents a method to improve the user experience of a telemedicine service based on the combination of televisits and telemonitoring with wearable sensors for heart failure patients.	Quantitative	Italy	heart failure patients	Gender Female 2 Male 13 Age [years old] 63.4 ± 6.7	telemonitoring for heart failure patients			Satisfaction questionnaires of patients and clinicians reached respectively 65.18% and 63.83%, while PSSUQ scores were respectively 91.73% and 81.70%.		Both groups of end users confirmed a good level of usability and their satisfaction about the ease of use and the perceived quality of the instrumentation. Moreover, 73% of patients did not require help from caregivers to use the kit of sensors.						UNDECLARED
Experiences of current vital signs monitoring practices and views of wearable monitoring: A qualitative study in patients [illegible]		Journal of Advanced [illegible]	Areia, C. and King, E. and Ede, J. and Young, L. and Tarassenko, L. and Watkinson, P. and Vollam, [illegible]		To understand current experiences of vital signs monitoring of patients and clinical staff on a surgical ward, and views on the introduction of wearable ambulatory monitoring into the general ward environment.				We interviewed six female and nine male patients, age ranged from 30 to 91 years old (median 68), also reflective of the [illegible]										[illegible]	[illegible]	

What Factors Predict the Adoption of Type 2 Diabetes Patients to Wearable Activity Trackers拼pplication of Diffusion of Innovation Theory	2022	Frontiers in Public Health	Chen, P. and Shen, Y. and Li, Z. and Sun, X. and Feng, X.L. and Fisher, E.B.	10.3389/fpubh.2021.773293	This study aims to explore the predicting factors of Chinese elderly type 2 diabetic patients' adoption intention to wearable activity trackers and their actual wearing behavior, using diffusion of innovation theory as the theoretical framework.	Quantitative	China	Diabetes Patients	this study, 342 male patients and 383 female patients. The majority of patients were the elderly, with a mean age of 60.3 ± 7.6 years old. The educational level of recruited patients was generally lower, with only 17.9% of patients receiving a college education and 25.6% receiving a high school education. The vast majority of participants (94%) were married and 40.4% of patients had an average monthly family income of 4,001 RMB and above. The mean duration of having type 2 diabetes in this study was 5.8 ± 3.5 years and the majority of patients (66.9%) had no diabetes-related complications.	Wearable Activity Trackers				The results indicated that observability was the primary influencing factor of patients' adoption intention (β = 0.775, P < 0.001). Relative advantage (β = 0.182, P = 0.014) and perceived social image (β = 0.080, P = 0.039) also had a positive influence while perceived risk (β = −0.148, P < 0.001) exerted a negative influence. In addition, results showed that the more intention led to the better actual wearing behavior (β = 0.127, P = 0.003). Observability (β = 0.103, P = 0.005), perceived ease (β = 0.085, P = 0.004), and relative advantage (β = 0.041, P = 0.009) also indirectly influenced the wearing behavior.								N
Smart Wristwatch and Apps for Healthy People with Congenital Diseases and a Healthy Lifestyle	2022	IEEE International Conference on Industrial Engineering and Engineering Management	Gozali, L. and Rafael, G. and De Candra, C. and Masrom, M. and Gunadi, A. and Zagloel, T.Y.M.	10.1109/IEEM55944.2022.9989656	An electronic device designed for use by an individual to actively or passively collect data relevant to that individual's health condition and behaviour. This research aims to develop a smart and healthy watch product design that can answer today's human health needs.	Quantitative	Indonesia	Healthy People with Congenital Diseases	GenderThe first variable that was asked in the questionnaire was gender. Of the 180 respondents reached in this questionnaire, there were 93 men and 87 women.	Smart Wristwatch and Apps							What kind of material do you want for a smart health watch?The results of questionnaire shows that stainless steel 77 people, copper 7 people, plastic 65 people, rubber 27 people, and 4 people chose other materials.<br><br>What features are needed on a Smart Health Watch? In the variable features needed on a Smart Health Watch, respondents chose 130 people for the Calorie requirement counter, 107 people for the sports program, 99 people for the daily water consumption counter, 89 people for hologram reminder schedule, 118 people for food calorie meter, heart rate gauge as many as 137 people, social media as many as 57 people, and other features as many as 16 people in table 1.	For replacing smartwatches, including the reason for internal damage as many as 39 people, do not understand the product as many as 7 people, less comfortable to wear there are 18 people, the battery is easy to run out there are 17 people, and other reasons there are 99 people.				UNDECLARED
How does passive remote monitoring technology affect perceived outcomes for older adults, their family and friend caregivers, and the healthcare system?	2022	Gerontechnology	Weeks, L.E. and Warner, G. and Nesto, S. and Rushton, H. and Ledoux, K. and Hiebert, B. and Donelle, L.	10.4017/GT.2022.21.1.504.10	The utilization of technology such as help from remote monitoring technologies (RMT) that send notifications from sensors (e.g., motion sensors, cameras, medication administration monitoring) to a recipient (e.g., family or friend caregiver, healthcare professional) can help to support care provision. Our objective is to report qualitative findings from the perspective of family and friend caregivers to examine: How does providing passive RMT to older home care recipients affect perceived outcomes for recipients, their family and friend caregivers, and the healthcare system?	Qualitative	Canada	older adults, their family and friend caregivers	interviewed ranged in age from 47-81 (mean=63.4, SD=10.4) and included 11 females (n=7 daughters, 3 wives, 1 sister) and 4 males (n=1 husband, 1 son, 1 brother-in-law, 1 friend). A total of 9 lived with the home care client and 8 were employed full or part-time. Seven had completed college or beyond and 6 reported some difficulty having enough financial resources to meet their household's needs. most are married, most earn more than 20000 a year, most thought their income is adequate or have difficulty	passive remote monitoring technology			Supporting aging in place emerged as an overarching theme as participants clearly discussed how passive RMT supports frail older adults to remain living in their homes longer or potentially eliminate the need for higher levels of care.					For example, while monitors outside the home were available, the people involved in these interviews only utilized monitors inside the client's home. One participant felt that monitors outside the home would be useful. Two participants identified that the system does not relay notifications when there are power outages. One participant whose mother had physical declines due to dementia during the study found the passive RMT more beneficial when her mother was more mobile. While the notifications could be tailored to the preferences of the caregivers, some may not have made full use of this tailoring process. Some caregivers found that receiving the notifications caused them some level of annoyance, stress, or anxiety, such as getting notifications at times they did not want to receive them, such as at nighttime or while at work.		Participants identified many ways that passive RMT supports the health and well-being of home care clients. A key theme focused on the perceived positive impact of the passive RMT on the overall well-being of family and friend caregivers.		UNDECLARED
Patient Acceptability of a Novel Technological Solution (Ambient Intelligent Geriatric Management System) to Prevent Falls in Geriatric and General Medicine Wards: A Mixed-Methods Study	2022	Gerontology	Dollard, J. and Hill, K.D. and Wilson, A. and Ranasinghe, D.C. and Lange, K. and Jones, K. and Boyle, E.M. and Zhou, M. and Ng, N. and Visvanathan, R.	10.1159/000522657	The Ambient Intelligent Geriatric Management (AmbIGeM) system used wearable sensors that detected and alerted staff of patient movements requiring supervision. The AmbIGeM clinical trial was conducted in two geriatric evaluation and management units and a general medical ward, in two Australian hospitals, and a subset of participants were recruited.	Mixed	Australia	patients	participants were female (cf., 55% for the trial) and the mean age was 83 years (i.e., similar to the trial) (Table (Table2).2). Only 8% had a diagnosis of dementia (cf. 17% in the trial). More than half of those interviewed, and one-third of those surveyed, had been admitted with a fall in the last 7 days (cf., 29% in the trial). On average, participants recruited for the post-survey and interview had been on the ward for 7.9 (SD = 4.5) days and 7.0 (SD = 3.6) days, respectively. No participants had fallen in hospital prior to completing the post-survey. Two participants had fallen in hospital prior to being interviewed.	Ambient Intelligent Geriatric Management System to Prevent Falls in Geriatric and General Medicine Wards			Participants found the AmbIGeM system and its purpose to be beneficial, appropriate, and valuable and indicated that they would value implementation in health care.		Participants considered the system as usable, being in the main comfortable to wear, with little personal impact, privacy concerns, or disruption to the ward experience or routine.Minor concerns were expressed about the singlet, but the sensor was acceptable and suggestions were made to expand capabilities of the sensor.					Some participant's perceived sense of safety was enhanced by the system monitoring participant movement and location, as participants believed nurses would be able to quickly attend to them if needed and prevent falls. Participants and their families thought that the system was potentially a useful backup system when participants were unable or forgot to press the call bell.benefits of technology for other people at risk of falls	Theme 4: Insufficient Participant Information and Understanding Not all participants thought that the patient information provided to participants in the clinical trial was adequate. Further, some participants' understanding of the purpose and mechanism of the system was incorrect.	A patent titled "System, method, software application and data signal for determining movement" was filed bythe Associate Professor Ranasinghe and Professor Visvanathan (lapsed 2015). Professor Visvanathan is the Director of Aged & Extended Care Services at The Queen Elizabeth Hospital in South Australia within which SA GEMU sits. The remaining authors have no conflicts of interest to declare.
Smartphone Ownership and Interest in Mobile Health Technologies for Self-care Among Patients With Chronic Heart Failure: Cross-sectional Survey Study	2022	JMIR Cardio	Leigh, J.W. and Gerber, B.S. and Gans, C.P. and Kansal, M.M. and Kitsiou, S.	10.2196/31982	Heart failure (HF) is a highly prevalent chronic condition that places a substantial burden on patients, families, and health care systems worldwide. The purpose of this study is to assess smartphone ownership and patient attitudes toward using mHealth technologies for HF self-care in a predominantly minority population in an urban clinical setting.	Quantitative	USA	Patients With Chronic Heart Failure	were approached and asked to participate in the survey. A total of 100 eligible patients (mean age 61.3, SD 12.3; range 29-93 years) agreed to participate, yielding a response rate of 69.4% (100/144). As shown in Table 1, most patients (63/100, 63%) were women, self-identified as Black or African American (61/100, 61%), were aged 50-64 years (49/100, 49%), had high school education or lower (65/100, 65%), and reported being retired or on disability (55/100, 55%). Of the 100 patients, only 49 (49%) reported their annual income. Of those who did report their annual household income, 53% (26/49) made <US $25,000 per year.	Mobile Health Technologies for Self-care Among Patients With Chronic Heart Failure			.Of the 68 patients who owned a smartphone, 15 (22%) reported using ≥1 mHealth app for self-monitoring of HF and other health-related data, 36 (53%) said that they did not use mHealth apps but would be interested in doing so in the near future, and 17 (25%) said that they were not interested at all in using mHealth apps. The interest in using wearables was high among patients who did not own an activity tracker (61/85, 72%). Also, the majority of study participants (76/100, 76%) reported willingness to transmit monitored data to their physician using mHealth devices.	lder adults (aged ≥65 years) were less likely to use these features than younger and middle-aged adults There was a significant relationship between education and current use of mHealth apps (ΦC=0.374; χ23=9.5; P=.02).	Most participants who owned tablets (n=38) reported being either comfortable or very comfortable (30/38, 79%) using a tablet. Of the 58 patients who owned a computer or laptop, 78% (45/58) reported being comfortable or very comfortable, and 22% (13/58) reported being somewhat or not comfortable using a computer.Among those who reported using mHealth apps for HF self-care (15/68, 22%), frequently used apps included Fitbit, Apple Health, and Samsung Health for physical activity tracking (14/15, 93%); MyFitnessPal, Lose it!, and Eat24 for tracking of diet and low-sodium foods (11/15, 73%); and Apple Health or Samsung Health for monitoring of blood pressure (13/15, 87%) and glucose levels (6/15, 40%). Overall, as shown in Table 3, the most popular health parameters that patients with smartphones would like to self-monitor using mHealth apps were physical activity (46/68, 68%), blood pressure (44/68, 65%), medication intake (40/68, 59%), weight (38/68, 56%), and diet (36/68, 53%).							N
Usability, Acceptability, and Satisfaction of a Wearable Activity Tracker in Older Adults: Observational Study in a Real-Life Context in Northern Portugal	2022	Journal of Medical Internet Research	Domingos, C. and Costa, P. and Santos, N.C. and P阮o, J.M.	10.2196/26652	This study aimed to (1) assess acceptability, usability, and user satisfaction with the Xiaomi Mi Band 2 in Portuguese community-dwelling older adults in a real-world context; (2) explore the mediating effect of the usability on the relationship between user characteristics and satisfaction; and (3) examine the moderating effect of user characteristics on the relationship between usability and user satisfaction.	Quantitative	Portugal	Older Adults	Participants had a mean age of 68.41 (SD 3.11) years, and 45.5% (50/110) were identified as males. The mean years of formal education were 7.95 (SD 5.38).	Wearable Activity Tracker in Older Adults			Overall, the average ratings for user experience with the Xiaomi Mi Band 2 were 6.45 (SD 0.78) for PEOU, 6.74 (SD 0.55) for PEC, 6.85 (SD 0.47) for CANX, and 6.60 (SD 0.97) for BI.	and depression) collectively explained 16.8% of usability variance. Furthermore, individual characteristics and usability collectively explained 39.1% of the variance in satisfaction. Specifically, depression negatively impacted usability and satisfaction, with a significant effect on usability (β=−.369; P<.01); while, regarding education, a positive, but not significant, usability and satisfaction effect was observed (education > satisfaction: β=−.121; P<.23; education > usability: β=−.130; P<.25). Despite confirming the theoretical model, most research hypotheses were not statistically proven with adequate goodness of fit. Nonetheless, usability seems to be a strong predictor of user satisfaction.	Regarding usability, the overall SUS score ranged from 55 to 100 (mean [SD] 92.70 [10.73]), with 96% (106/110) of the participants reporting a score above the acceptability baseline of the SUS. Moreover, 45.5% (50/110) of the participants classified the activity tracker achieved as best imaginable (Table 6). Thus, these results suggest that the Xiaomi Mi Band 2 is a usable wearable activity tracker among older adults.These results indicate that participants found that the Xiaomi Mi Band 2 is an easy-to-use and easy-to-control device, potentially perceiving its usefulness regarding health benefits and having the intention to use it in the future.Finally, all participants reported a user satisfaction experience above the USEQ baseline value defined as a good experience, with a mean USEQ score of 23.30 (SD 2.40; Table 4). Moreover, 85.5% (94/110) of the participants rated the satisfaction with the Xiaomi Mi Band 2 as excellent (Table 6). Still, despite older adults reporting good satisfaction with the device, concerns were noted regarding the clarity of the technology's information.							N
User experiences and perceptions of health wearables: an exploratory study in Cambodia	2021	Global Health Research and Policy	Liverani, M. and Ir, P. and Wiseman, V. and Perel, P.	10.1186/s41256-021-00221-3	Considering this potential, the purpose of this study was to explore user experiences and perceptions of a health wearable in Cambodia.	Quantitative	Cambodia	general	A total of 156 adult participants completed the study. These included 60 hypertensive participants (30 in Phnom Penh and 30 in Kampot), 60 non-hypertensive adults (30 in Phnom Penh and 30 in Kampot), 18 civil servants and 18 postgraduate students in Phnom Penh. Only one participant at baseline (a civil servant in Phnom Penh) did not complete the follow-up survey due to watch malfunction.<br><br>Table 1 shows the demographic and socio-economic characteristics of the participants. On average, participants were aged 53.2 years (SD: 14.5; range: 22–79). In Phnom Penh, 59.8% of participants had some secondary or higher education and 52.6% were in the highest socio-economic status group. By contrast, most participants in Kampot had attended only primary school (48.3%) and were in the lowest socioeconomic group (71.7%).	health wearables			Although health wearables were new to most participants, the technology acceptance was positive overall. 89.1% said they would continue using the watch and 76.9% would recommend it to either friends or relatives, .		Despite this positive attitude towards the device, only 34% of participants used the watch all the time during the study period, as instructed. When asked to provide further explanation, the most frequently mentioned barrier to utilisation was low battery life (n = 75/186), which lasted 3.9 days on average (SD = 2.9). Other reasons that were frequently given to explain sporadic use included watch malfunction (n = 28/186), the inconvenience of wearing the watch while taking a bath or shower (n = 36/186) and farming (n = 19/186). A minority of participants (n = 3) also complained they were "annoyed" by the watch, while others said the display was too small. Of note, two participants reported "pain in the arm" and a "sense of tingling in the chest" after wearing the watch.<br><br>Specific features of the wearable device such as the measurement of heart rate, steps, and blood pressure were used by 89.1, 85,3 and 80.1% of participants respectively. Only 32,1% used the calories tracker (Fig. 1). In keeping with this pattern, when asked to name the most useful functions, the majority of participants (62.8%) named blood pressure measurement as the first choice, followed by heart rate monitor as the second choice (48.1%) and step counter as the third choice (19.9%). As expected, being hypertensive or older than 45 years was significantly associated with a preference for blood pressure measurement. Only a minority of participants (25.6%) used the linked smartphone application, largely students (84.2%) and civil servants (72.2%)		.Lastly, when asked if they had any suggestions for improvement, most participant mentioned that the watch should be more resistant to water or scratches (n = 56/123) and its battery life (n = 49/123) could be improved.	Considering the potential marketing of a similar product as part of a public health programme, we were interested in exploring how much people would be willing to pay for it. In both study locations, respondents were willing to purchase the device for an average price of 46,774 Cambodian riels (US$ 11.4 Range: US$ 2.4–48.7). However, only 60% of participants were willing to buy the product. Socioeconomic status had a statistically significant effect on mean scores of WTP (Table 4).		while 94% said the device stimulated them to think more frequently about their health (Table 3)		N
Patient and clinician experiences and opinions of the use of a novel home use medical device in the treatment of peripheral vascular disease - a qualitative study	2021	Journal of Foot and Ankle Research	Sedgwick, C.E. and Growcott, C. and Akhtar, S. and Parker, D. and Pettersen, E.M. and Hashmi, F. and Williams, A.E.	10.1186/s13047-021-00496-2	As part of the development process, this study aimed to explore patient and clinician opinions of the boot.	Qualitative	Norway	Patient with peripheral vascular disease	3F, 4M;	a novel home use medical device in the treatment of peripheral vascular disease			Positive experiences from device use emerged throughout most interviews:		Most found the device comfortable and easy to use:<br>All were able to use the device independently:<br>All described accommodating the device in their homes and around routines. One patient described using the device for longer than the prescribed daily 2 hours:	Experiences of using FlowOx™ revealed recurrent dialogue from patients that focused on symptomatic changes to lower limb health. Personal accounts highlighted significant functional daily living changes from diagnosis and through disease progression. Pain levels dictated physical activity limits:<br><br>The risk of limb loss for those with peripheral arterial disease ultimately led to experiencing feelings of fear and worry:	Some practical usage issues were revealed with the seal component. Pulling on the seal proved difficult for some. It was postulated that the elderly may have difficulties with the design.:<br><br>Differences in the seal material lead to an increase in size, corrosion and problems achieving maximum pressure:	Views on how much they were willing to pay along with marketing suggestions for the device were mixed:<br><br>"So monetary wise it's certainly is worth a lot in terms of improving people's functionality in their everyday life … it is a foot saver, you could say." [p5]<br><br>"I call it the wonder machine."[p4]<br><br>"I can't set a price on it because it saved my life. It gave me my life back, in a way, so for me it's … priceless … one suck and you're hooked." [p1]		Outcomes included improved healing, circulation, and pain reduction.Two patients gave lengthy detail to the changes to their lower limb health during device use and that had resulted in a complete symptomatic reversal, varying degrees of wound healing and complete wound resolution:<br><br>Some described fond feelings for the device and relief in avoiding amputation. Continued use was described as necessary to prevent symptom deterioration:<br><br>These positive health improvements were described as having a significant impact on the ability to carry out daily tasks:		N
Willingness to Share Data from Wearable Health and Activity Trackers: Analysis of the 2019 Health Information National Trends Survey Data	2021	JMIR mHealth and uHealth	Rising, C.J. and Gaysynsky, A. and Blake, K.D. and Jensen, R.E. and Oh, A.	10.2196/29190	This study aims to identify sociodemographic, health, and digital health behavior correlates of US adults' willingness to share wearable data with health care providers and family or friends.	Quantitative	USA	general	The analytic sample comprised 1300 wearable users. Analysis of weighted data showed that women (55.03%) and men (44.97%) each constituted approximately half of the sample. The majority were under 50 years of age (64.1%), urban residents (88.07%), non-Hispanic White (64.69%), and reported having an education beyond high school (85%). Individuals with annual household income under US $75,000 comprised 44.02% of the sample, with the remaining 55.98% having an annual household income of US $75,000 or more (Table 1).	Wearable Health and Activity Trackers			A majority of individuals reported that they would be willing to share wearable data with health care providers (81.86%) and with family or friends (69.1%).	Regression analysis showed that individuals who reported higher (vs lower) health self-efficacy (odds ratio [OR] 1.97, 95% CI 1.11-3.51), higher (vs lower) trust in health information from a physician (OR 1.98, 95% CI 1.12-3.49), and higher (vs lower) levels of physical activity (OR 2.00, 95% CI 1.21-3.31) had significantly greater odds of reported willingness to share wearable data with providers. Among the digital health behaviors, higher (vs lower) frequency of wearable use (OR 2.15, 95% CI 1.35-3.43) and use of mHealth technologies to help communicate with providers (OR 1.99, 95% CI 1.09-3.63) were significantly associated with willingness to share wearable data with providers (Table 3). only individuals who reported higher (vs lower) levels of physical activity had higher odds of reported willingness to share wearable data with family or friends (OR 1.70, 95% CI 1.02-2.84; P=.04). In the bivariate analyses, willingness to share wearable data with providers was significantly associated with trust in health information from a physician and level of physical activity. Willingness to share wearable data with family or								N
Willingness to use a wearable device capable of detecting and reversing overdose among people who use opioids in Philadelphia	2021	Harm Reduction Journal	Kanter, K. and Gallagher, R. and Eweje, F. and Lee, A. and Gordon, D. and Landy, S. and Gasior, J. and Soto-Calderon, H. and Cronholm, P.F. and Cocchiaro, B. and Weimer, J. and Roth, A. and Lankenau, S. and Brenner, J.	10.1186/s12954-021-00522-3	We sought to understand the preferences and minimum necessary conditions for wearing a device capable of sensing and reversing opioid overdose among people who regularly use opioids.	Mixed	USA	people who use opioids	The study sample was mostly white (47%) and majority male (59%). Participants reported a median duration of opioid use of 12 years (IQR: 8–20 years), with 71% of participants reporting one or more lifetime overdoses.	a wearable device capable of detecting and reversing overdose among people who use opioids in Philadelphia					Reliance on bystander interventions was the least preferred of the device methods, with 54% responding they would likely wear a device that straps naloxone to their body for a bystander to administer, and 63% responding they would be likely to wear a device that senses opioid overdose and alerts a bystander. Solutions with purely monitoring functionality were the most preferred, with 77% responding likely to wear a device that monitors vital signs and 73% responding that they were likely to wear a device that senses opioid overdose. Reliance on trained medical intervention received intermediate responses, with 71% responding that they were likely to use a device that alerts medical first responders of an opioid overdose and 69% responding that they were likely to wear a device that indicates the wearer is at risk of overdose (like a medical ID). Finally, 67% of respondents indicated it is likely they would wear a device that senses an overdose and administers naloxone if needed. This likelihood remained consistent regardless of individual-level factors assessed based on Chi-squared analysis summarized in Additional file 2: Table 1.<br><br>Responses varied significantly between questions asking preferred location to wear a sensor. A watch-appearing bracelet (77%) and a wrist-like bracelet (73%) had the highest proportion likely to wear. A necklace (51%) and a skin patch on the upper arm (55%) were the only other locations that more than 50% of respondents indicated they were likely to wear an overdose-sensing device. Data regarding preferences for shoulder or thigh location for a device to automatically sense and reverse opioid overdose are separately presented in Additional file 3: Table 2.	Everyone More than any specific subpopulation, participants expressed a need for a device to automatically sense and reverse opioid overdose for anyone with an ongoing opioid addiction. One participant said such devices should be "hand[ed] out like Narcan." Another participant cited the danger of overdosing on a drug supply of unknown potency as a reason why the device would be widely useful: "You can overdose from 10 bags [of heroin] or 1 bag, you never know."<br><br>People who are homeless A number of participants highlighted people who are experiencing homelessness and use opioids as particularly vulnerable to overdose.<br><br>People with low tolerance for opioids Participants identified a number of populations at increased risk of overdose due to diminished tolerance and thus in greater need for the proposed device. These included prison, people who had recently been released from jail or prison, people who had recently returned to use, and people coming to the Kensington-area from the surrounding tri-state area in order to use drugs, thus	This is related to two factors: (1) size of the device, as 89% of participants stated a preference for the smallest of the non-functional prototypes, and (2) concealment, with many participants describing blending with clothing and skin tone and having the appearance of a consumer "smart" device as preferred features.<br><br>Comfort Participants expressed the importance of the device being comfortable. One participant relayed the desire for a "soft, bendable" contact surface that would not cause discomfort during sleep if worn overnight. Others were discomforted by the texture of the box component and the straps of the non-functional prototypes.<br><br>Indifference A number of participants reported that if the device had the potential to reverse an otherwise fatal overdose, there is no aspect of the device's appearance that would dissuade them from wearing it, citing the life-saving functionality of the proposed device.			In open-ended conversations regarding the device concept, both overall positive and negative sentiments were present. Numerous participants were excited by the life-saving potential of the device and were confident such technology would keep them safer.	Negative sentiments expressed by participants included aversion toward the possibility of "losing a high" after a false positive leads to reversal agent injection when overdose has not occurred, and inability to distinguish opioid overdose from non-opioid overdose leading to an ineffective reversal attempt. Some participants were also concerned of law enforcement misinterpretation of the device as a stash of drugs underneath clothing or a GPS monitoring device. Finally, some participants worried that a configuration of the device that alerted bystanders to an overdose would leave the person overdosing vulnerable to physical harm or robbery at the hands of ill-intentioned bystanders. A minority of participants believed that the device would be misplaced or stolen.	N
Utility and usability of an automated COVID-19 symptom monitoring system (CoSMoS) in primary care during COVID-19 pandemic: A qualitative feasibility study	2021	International Journal of Medical Informatics	Lim, H.M. and Abdullah, A. and Ng, C.J. and Teo, C.H. and Valliyappan, I.G. and Abdul Hadi, H. and Ng, W.L. and Noor Azhar, A.M. and Chiew, T.K. and Liew, C.S. and Chan, C.S.	10.1016/j.ijmedinf.2021.104567	This study aimed to evaluate the feasibility of a COVID-19 symptom monitoring system (CoSMoS) by exploring its utility and usability with end-users.	Qualitative	Malaysia	patients diagnosed with suspected COVID-19 infection or close contact with a COVID-19 patient	AGE:29-60; 8F, 7M	an automated COVID-19 symptom monitoring system (CoSMoS) in primary care during COVID-19 pandemic					Theme 1: Familiarity with existing apps smoothen implementation Overall, patients preferred to use their existing social media app for COVID-19 symptoms monitoring. Two subthemes emerged regarding the installation of Telegram and CoSMoS.<br>3.3.1.2. Subtheme 1b: Using different approaches to guide patients to install the app Although patients faced some difficulties installing Telegram, they could complete the Telegram installation with guidance, such as a setup guide (visual guide) and step-by-step guidance via phone calls.<br>3.3.2. Theme 2: User-centered and evidence-based content of home monitoring app Four subthemes emerged related to the content of CoSMoS.<br>3.3.2.1. Sub-theme 2a: Use of simple questions with a user-friendly interface Patients commented that the questions asked in the CoSMoS bot are simple and easy to answer. Patients just clicked on the option which suits them the best. The clickable buttons improved CoSMoS's usability greatly without having to type their symptoms.<br>3.3.2.4. Sub-theme 2d: Redundant content in the Telegram bot Asking about cyanosis "Is your face or lips turning blue?" was one of the questions recommended by the World Health Organization (WHO) and local guidelines. However, patients had difficulty understanding the question asking about cyanosis. Doctors felt that the question asking about cyanosis was redundant.		3.3.2.2. Sub-theme 2b: Updating the content according to the latest evidence Patients and doctors suggested adding more questions on COVID-19 symptoms, especially anosmia when more COVID-19 cases reported presenting with anosmia.	3.3.1.1. Subtheme 1a: Unfamiliarity with app poses challenges in installation Current Telegram users had no problem installing CoSMoS. For new Telegram users, some struggled to install and navigate the Telegram. Patients preferred to use the existing app on their phones, such as WhatsApp, rather than installing a new app.		3.2.1. Theme 1: Providing close monitoring for patients who were quarantined at home Patients found that CoSMoS was useful in providing remote monitoring without having to visit a doctor. Patients felt better when doctors monitored them closely through the CoSMoS system. CoSMoS reassured patients by providing close monitoring. It reduced their uncertainty and emotional stress during the COVID-19 pandemic.<br>3.2.2. Theme 2: Providing continuity of care to patients CoSMoS provided continuity of care to patients after being discharged from the primary care clinic. Doctors found that CoSMoS enabled them to build a good rapport with patients and developed a trustable patient-doctor relationship.<br>3.2.3. Theme 3: Providing timely decision support for patients and doctors Patients commented that CoSMoS helped them decide whether to go to the hospital or stay at home when their symptoms changed. Patients found that the automated feedback given to them after entering their symptoms was useful in their decision-making. They felt that the advice given were appropriate to their condition and easy to understand.		N

Developing a sensor-based mobile application for in-home frailty assessment: a qualitative study	2021	BMC Geriatrics	Blinka, M.D. and Buta, B. and Bader, K.D. and Hanley, C. and Schoenborn, N.L. and McNabney, M. and Xue, Q.-L.	10.1186/s12877-021-02041-z	We conducted focus groups discussions to solicit input from older adult care recipients (non-frail, pre-frail, and frail), their informal caregivers, and medical providers about their preferences to tailor a mobile app to measure frailty in the home using sensor based technologies.	Qualitative	USA	patient for in-home frailty and caregiver	Ninety potential participants were contacted for the study and 15 care-recipients and their caregivers agreed to participate. Due to health and transportation problems, one care-recipient and three caregivers were unable to participate. The mean age for care recipients was 76 (standard deviation (sd) = 7). Most were Caucasian (71%), female (64%), and reported being in good or better health (43%). Similarly, caregivers were mostly Caucasian (75%) and female (67%), with a mean age of 68 (sd = 8). Assistance provided by caregivers included cooking, shopping, physical support (e.g. walking), household chores and driving to doctors' visits. Medical providers were mostly female, nurse practitioners, registered nurses, or licensed practical nurses (n = 6), followed by physical and occupational therapists and support staff (n = 5), and geriatricians (n = 3). All were involved with the care of older adults.	a sensor-based mobile application for in-home frailty assessment								Subtheme 2: concerns about home-based sensors Providers identified concerns about affordability and access for some of their patients. As one provider explained, "If it involves an Internet connection, a number of our participants do not have Internet and can't afford it. "A main concern for care recipients was care quality rather than access		Subtheme 1: benefits of home-based sensors Providers, care-recipients and caregivers felt that sensors would be useful to provide objective data and would be helpful and cost-effective way to track changes associated with frailty and declining health. They also anticipated that sensors could reduce health-care costs, which would be good for the patient, caregiver and provider, and would enhance the independence of older adults.	[illegible] Care recipients worried that the use of sensors would tend to replace or reduce in-person visits.<br>Care-recipients, caregivers, and providers shared concerns about the large volume of data generated and whether the data could be interpreted and reported in a timely and useful manner. Additionally, providers had unique concerns regarding responsibility for data administration and reporting (including to whom the data would be reported), and how such data could be useful in preparing overall care plans for older adults.<br><br>Subtheme 1: concerns about data interpretation, reporting and responsibility Care-recipients and caregivers had common concerns about the large volume of data generated, and whether that amount of data could be interpreted in a timely and useful manner.<br>Subtheme 2: data security Care-recipients, caregivers, and providers had concerns regarding data security, and how the data	N
Real-World Patient Experience of Long-Term Hybrid Closed-Loop Insulin Pump Use	2021	Canadian Journal of Diabetes	Wang, L.R. and Malcolm, J. and Arnaout, A. and Humphrey-Murto, S. and LaDonna, K.A.	10.1016/j.jcjd.2021.02.006	Understanding of patient experiences and adaptations to hybrid closed-loop (HCL) pumps beyond the confines of short-term clinical trials is needed to inform best practices surrounding this emerging technology.	Qualitative	Canada	Diabetes patients	Demographic characteristics of study participants, collected via questionnaire and verified with health records where possible<br><br>Characteristic Value<br>Gender, female, n (%) 12 (57%)<br>Age, years<br><30 3<br>30–39 0<br>40–49 6<br>50–59 9<br>60–69 2<br>70+ 1<br>Education, n (%)<br>Graduate or professional degree 9 (43%)<br>Bachelor/associate degree 5 (24%)<br>Some college 3 (14%)<br>High school or GED 1 (5%)<br>Not disclosed 3 (14%)<br>Diabetes duration, years, mean ± SD 27.5±13.9<br>Duration of pump use, years, mean ± SD 13.9±7.6	Long-Term Hybrid Closed-Loop Insulin Pump Use			Participants described having high hopes for auto-mode, initially anticipating that "it would always keep you 100% in range" (patient [P] 4) or "it was just set it and everything was going to be wonderful" (P11). Several participants described eagerly anticipating its arrival in Canada; one participant "had been watching literally for years" for news regarding the pump (P2).		Many participants felt that auto-mode did not significantly reduce the work of managing their diabetes, describing it as "annoying" (P1, P2, P8–13, P17, P19, P20) or "tedious" (P12). Participants pointed to the unexpected work of attending to frequent alarms and calibrations, troubleshooting sensor problems and checking the pump often to monitor its activity:	Participants appeared to cluster into 2 groups, emphasizing different motivations for trying auto-mode, including 1) those who felt significant struggle to achieve glycemic targets (A1C 8% to 10%, or severe lows), and whose main goal was to improve their A1C or hypoglycemia; and 2) those with controlled diabetes (A1C 6.5% to 7.3%), who emphasized reducing the burden of management, or wanting the latest technology.<br><br>Participants with uncontrolled diabetes described "battling" with blood sugars (P7, P14), having "labile" (P16) or "brittle" (P10) diabetes, "not (being) the best patient" (P19) or having suboptimal results despite best efforts:<br><br>In addition, some patients described traumatic experiences with severe hypoglycemia or hospitalization:<br><br>These patients almost unanimously cited the desire for more stable blood sugars as the main reason for switching to auto-mode.		Part of their disappointment was associated with the perception that users had to adapt their lifestyle to achieve their desired glycemic control).<br><br>For some, the glycemic benefits did not justify the demands of the system, leading to discontinuation:		Most participants praised the ability of auto-mode to reduce hypoglycemia, improve A1C and improve stability overnight. The main complaints were regarding frequency of alarms and user input, sensor problems and inadequate responsiveness to hyperglycemia.	Frustration with hyperglycemia and perceived lack of effective means to correct it were accompanied by the urge to intervene with workarounds. Nearly all participants with A1C <7.5%, and a few with higher A1Cs, mentioned having tried "lying to the pump" (P20) to provide extra insulin. Participants familiar with "cheating" often acknowledged its potential for negative consequences, as it prevents the pump from learning from its shortfalls, thus "digging the hole deeper" (P18) in terms of inadequate insulin delivery.<br><br>Nevertheless, some participants felt that phantom bolusing was justified or necessary in some circumstances, citing inadequate "learning" by the pump (P11), rationalizing "fake carbs" as "catching up" for underestimated carbs (P2, P18), or to address predictable glucose spikes triggered by intense exercise or circadian rhythms (P1, P3, P6, P11), which the pump was unable to control. They reflected on the trade-offs	N
The understanding, acceptability, and relevance of personalised multidimensional physical activity feedback among urban adults: evidence from a qualitative feasibility study in Sri Lanka	2021	BMC Public Health	Arambepola, C. and Perera, M. and Gillison, F. and Peacock, O. and Thompson, D.	10.1186/s12889-021-10774-0	This study assessed the understanding, acceptability, and relevance of personalised multidimensional physical activity feedback in urban dwellers in Colombo, Sri Lanka.	Qualitative	Sri Lanka	General public	Characteristics<br>Adults at risk of NCD (n = 11)<br>Apparently healthy adults (n = 18)<br>Healthcare providers (n = 6)<br>Sex<br>Male 4 (36.4%) 11 (61.1%) 2 (33.3%)<br>Female 7 (63.6%) 7 (38.9%) 4 (66.7%)<br>Current marital status Single 2 (18.2%) 4 (22.2%) 0 (0%)<br>Married 9 (81.8%) 14 (77.8%) 6 (100%)<br>Highest level of education Grade 1–10 3 (27.3%) 3 (16.7%) 0 (0%)<br>Passed G.C.E. (O/Level)a 0 (0%) 7 (38.9%) 0 (0%)<br>Passed G.C.E. (A/Level)a 3 (27.3%) 3 (16.7%) 0 (0%)<br>Vocational training 0 (0%) 1 (5.6%) 5 (83.3%)<br>University education 5 (45.5%) 4 (22.2%) 1 (16.7%)<br>Monthly family income (in rupees)<br>> 60,000 7 (63.6% 5 (27.8%) 6 (100%)<br>41,000 to 60,000 0 (0%) 4 (22.2%) 0 (0%)	personalised multidimensional physical activity feedback among urban adults						Theme 3 – motivation to change behaviour (Table 4)<br>Theme 4 – consideration of the multidimensional nature of physical activity (Table 5)<br>Around half of the participants appeared to re-evaluate their own current PA levels; and suggest more suitable or tailored options to change behaviour that would fit with the practical issues, physical abilities, and health conditions that they faced. ergy expenditure.	Theme 1 – understanding the feedback received on PA profile (Table 2) For instance, the colours used in traffic light system, health wheel and spikes to represent different intensities in the activity graphs, as well as the concepts on non-sedentary time, moderate & vigorous bouts, and calorie burn were confusing to some.	In contrast, only one adult at risk of NCDs and two apparently healthy adults showed apathy towards the feedback in the day by day activity section, partly because, although such information was novel, it did not provide a straightforward message on the prediction of their disease risk related to PA. This was also coupled to the fact that activity graphs in the day-by-day activity section were more difficult to understand.		The feedback showed some potential to counteract common social myths about PA, such as whether age is a barrier to PA, that activities of daily living can contribute to fitness and calorie burn, and that even short bouts of exercise can be important. Also, the feedback was novel as it provided new knowledge about PA guidelines as well as awareness on their own behaviour.		N
搭 question of trust?and 揅 leap of faith?study participants' perspectives on consent, privacy, and trust in smart home research: Qualitative study	2021	JMIR mHealth and uHealth	Kennedy, M.-R. and Huxtable, R. and Birchley, G. and Ives, J. and Craddock, I.	10.2196/25227	This study aims to explore the ethical considerations around consent, privacy, anonymization, and data sharing with participants involved in SPHERE (Sensor Platform for Healthcare in a Residential Environment), a project for developing smart technology for monitoring health behaviors at home.	Qualitative	UK	General public	[illegible] occupants, n (%) Association with university Attendance of >1 pre-event<br>H01 Family 4 (25) Yes Yes<br>H02 Family 4 (25) Yes Yes<br>H03 Family 2 (13) Yes No<br>H04 Couple 2 (13) Yes Yes<br>H05 Individual 1 (6) No Yes<br>H06 Individual 1 (6) No Yes<br>H07 Shared residence 2 (13) Yes No Household ID and participant ID Gender Age (years), range<br>H01<br>P01 Male 51-60<br>P02 Female 51-60<br>P03 Female 11-16<br>P04 Female 11-16<br>H02<br>P05 Male 31-40<br>P06 Female 31-40<br>P07 Male 0-5<br>P08 Male 0-5<br>H03<br>P09 Female 41-50	SPHERE (Sensor Platform for Healthcare in a Residential Environment)			Whatever their specific motivations for taking part, all households expressed satisfaction with the consent process, including the information received and the opportunity to have their questions answered.	Trust in Research<br>Participants exhibited a great deal of trust in the project and the team, which underpinned their perceptions of the adequacy of the arrangements around informed consent, privacy, and data use. Four factors appeared relevant to developing and maintaining their trust: being a trustworthy project and team, transparently providing participants with necessary information, allowing participants control over their participation, and the participants having prior positive research participation experiences.		In terms of causal factors, households primarily became involved in SPHERE after attending public engagement events or because of existing links to the university. Approximately 71% (5/7) of the households had at least one member who had attended an engagement event. These events were described as informative and "interesting" (P14 from H06). Some participants also noted how attendance had enabled them to contribute to the development of the project, for example, following a "lively debate" about the "level of intrusion we might be prepared to accept" (P01 from H01). Participation in such events was not mentioned by the other 29% (2/7) of households, although those households did refer to an existing affiliation with the university. This was also mentioned by 60% (3/5) of those who attended engagement events.<br><br>Participants had a range of motivations for becoming involved; however, all had in common an altruistic desire to benefit others, which found expression in participation as they expected SPHERE to generate future health-related benefits. Although the participants were aware that the research would not	However, equipped with their knowledge of what participation had involved, some offered suggestions about disclosure to future participants to make consent more informed and practical. Many expressed an interest in receiving feedback on the data generated from their household, as well as about how the researchers were using the data collected. However, the latter was more for interest—"There's no reason for me to know it anyway" (P13 from H05)—and some participants appreciated that the research team would not be able to predict all future uses of the data, as "[i]t's kind of exploratory research in that way" (P05 from H02). However, many households did indicate that they would have liked more explanation of the practical ramifications of and daily burdens associated with participation rather than those items typically included in information leaflets:	However, 5 areas of difficulty and tension also emerged, concerning recall of information, understanding of that information, information overload, timing of information provision, and the possibility that consent might be more theoretical than actual.			Participants confirmed that privacy and confidentiality were important to them; however, they expressed few concerns about these, provided that 4 conditions were satisfied: the data collected (and shared) were anonymous, the data were nonsensitive, data collection was unobtrusive; and, consistent with their altruistic motivation, the research (including any follow-on studies) was directed toward the public good.	N
Demonstrating the feasibility of digital health to support pediatric patients in South Africa	2021	Epilepsia Open	Davies, E.H. and Fieggen, K. and Wilmshurst, J. and Anyanwu, O. and Burman, R.J. and Komarzynski, S.	10.1002/epi4.12527	Resources for management of epilepsy in Africa are extremely limited reinforcing the need to develop innovative strategies for optimizing care. Studies have shown that the prevalence of epilepsy in low- and middle-income countries is substantially greater than in more resourced countries. The objective of this report was to demonstrate that mobile Health (mHealth) technologies have the potential to improve the management of epilepsy in Africa.	Quantitative	South Africa	pediatric patients	[illegible] engagement consisted of 19 males (48.7%) and 20 females (51.3%) with a median age of ten years at the time of inclusion, ranging from 4-16 years. Most of the patients were black African (69.2%). Although more than half were living in formal housing, 69.2% of participants share their bed. Although the majority of caregiver participants (82.1%) were the mother, a number of grandmothers were the primary caregiver (12.8%).	wearable device			Median engagement with the wearable device and monthly mPROs was 30.8% and 57.1%, respectively.					However, most participants (87%) had to be given smartphones for them to have Bluetooth capabilities and access to the study app. Tolerability to the device was impacted by the difficult living circumstances of caregivers that induced fear of loss or theft.				OA and SK are employees at Aparito. EHD is employee at Aparito and holds shares. RJB, KF, and JW have no competing interests.
Multiparameter continuous physiological monitoring technologies in neonates among health care providers and caregivers at a private tertiary hospital in Nairobi, Kenya: Feasibility, usability, and acceptability study	2021	Journal of Medical Internet Research	Ginsburg, A.S. and Kinshella, M.-L.W. and Naanyu, V. and Rigg, J. and Chomba, D. and Coleman, J. and Hwang, B. and Ochieng, R. and Ansermino, J.M. and MacHaria, W.M.	10.2196/29755	This study aims to assess the feasibility, usability, and acceptability of 2 noninvasive, multiparameter, continuous physiological monitoring technologies for use in neonates in an African health care setting.	Qualitative	Kenya	Neonates, Caregivers	Overall, the use of the relevant technologies was observed with 12 neonates, and observations took between 2 and 10 minutes per technology. In addition, 27 interviews were conducted, including 10 caregivers (9 mothers, 1 father), 2 HCP-D (study nurses), 10 HCP-I (4 medical doctors, 6 nurses), and 5 health care administrators (nurse managers, program administrators, and hospital unit supervisors). One HCP-I and no caregiver declined to participate. All health care administrators and HCPs had a postsecondary education. There were 2 HCPs with diplomas in nursing (1 HCP-D and 1 HCP-I), and all other health professionals had bachelor's or master's degrees. Health professionals had a median of 9 (<1-29) years of work experience in the medical field. In addition, all but 1 caregiver had a postsecondary education. The median age of caregivers was 33 (range 28-38) years, and they had a median of 2 (range	Multiparameter Continuous Physiological Monitoring Technologies in Neonates	Half of the caregivers interviewed also mentioned that the investigational technologies were simpler, less intimidating, and more acceptable than conventional monitoring technologies.				Ease of use and efficiency of the technologies for neonatal care were major usability facilitators reported by most HCPs (9/12, 75%), health care administrators (4/5, 80%), and caregivers (7/10, 70%).The potential for wireless features to improve work efficiency was another major usability factor reported by the participants.<br><br>Fewer attachments have also been reported to increase efficiency in care by reducing the need to disconnect and reconnect the neonate during HCP examinations and caregiving needs, such as breastfeeding. Speaking of the EarlySense technology, a health care administrator said:<br><br>However, with the EarlySense technology specifically, there were concerns from HCP and health care administrators (5/17, 29%) of monitoring during disruptions when the neonate was off the mattress during breastfeeding or otherwise being carried by caregivers.<br><br>Small size and portability were reported by some HCPs and health care administrators (8/17, 47%) as potential facilitators and challenges to usability. Although portability and convenience are linked, there was also concern about misplacement and theft because of their small size and portability. For example, a health care administrator said that it would be critical for the Sibel technology:<br><br>Other usability challenges included concerns about infection control, shared by 41% (7/17) of the HCPs and health care administrators. In addition, 26% (7/27) of overall participants expressed concerns that equipment may be too large for preterm and					Acceptability Factors for the Investigational Technologies<br>Perceptions of neonate comfort were a major acceptability facilitator reported by many of the HCPs (6/ 12, 50%), health care administrators (4/5, 80%), and caregivers (610, 60%).	Concern about potential harm around electrical fields and wireless connectivity was a major acceptability challenge mentioned by caregivers (8/10, 80%) and HCPs (7/12, 58%).	N
What matters the adherence with BP 24-hr self-monitoring wearable device among hypertensive patients? A population-based survey	2021	Translational Behavioral Medicine	Zhang, Y. and Yang, N. and Si, G. and Zhang, Y. and Dong, Z. and Huang, Y. and Tan, X.	10.1093/tbm/ibz069	This study was designed to evaluate user characteristics of blood pressure (BP) wearable technology among a community-based population in a rural area of China.	Quantitative	China	hypertensive patients	[illegible] all survey and measurements at baseline. Of the 317 subjects, 171 (53.94%) were male, with mean age of 64.57 years (SD: 11.68, range: 23–98). Across the 30-day study period, 52.68% of the sample uploaded BP wristband data at least once daily. The 52.68% with uploaded data were defined as users (mean age: 63.95 years, SD: 12.06, 56.89% male), whereas the remaining 47.32% were nonusers (mean age: 65.25 years, SD: 11.23, 50.67% male). Baseline characteristics for the users and nonusers are summarized in Table 1. A significant difference between users and nonusers was observed for concomitant disease(s) (p = .011,).	BP 24-hr self-monitoring wearable device among hypertensive patients		As shown in Table 3, both the user and nonuser groups reported some level of technology fluency other than "Not at all" on the CEW Fluency Scale for the 17 investigated skills.		Individuals reporting a higher level of compliance in hypertension management are more likely to interact with a wearable BP-monitoring device than persons with lower levels of compliance.									N
Prospective Intention-Based Lifestyle Contracts: mHealth Technology and Responsibility in Healthcare	2021	Health Care Analysis	Feng-Gu, E. and Everett, J. and Brown, R.C.H. and Maslen, H. and Oakley, J. and Savulescu, J.	10.1007/s10728-020-00424-8	This paper has two components. Firstly, it details empirical findings from a survey of 81 members of the United Kingdom general public on public attitudes towards individual responsibility and rationing healthcare, prospective and retrospective responsibility, and the acceptability of lifestyle contracts in the context of mobile health technology. Secondly, we draw on the empirical findings and propose a model of prospective intention-based lifestyle contracts, which is both more aligned with public intuitions and less ethically objectionable than more traditional, retrospective models of responsibility in healthcare.	Quantitative	UK	patients	Demographic information Category % of total participants<br>Gender Male 30.9<br>Female 69.1<br>Non-binary 0<br>Prefer not to say 0<br>Age 18–24 23.5<br>25–34 32.1<br>35–44 21.0<br>45–54 8.6<br>55–64 9.9<br>65+ 4.9<br>Prefer not to say 0<br>Highest education level Primary school education 0<br>Secondary school education 27.2<br>Undergraduate tertiary education 48.1<br>Postgraduate tertiary education 24.7<br>Prefer not to say 0<br>Political orientation Very left-wing/liberal 11.1<br>Moderately left-wing/liberal 19.8<br>Somewhat left-wing/liberal 23.5<br>Neither left-wing/liberal nor right-	mhealth			Participants held mixed opinions overall regarding the use of digital trackers instead of a discussion with a physician to assess lifestyle, with 44.5% agreeing and 40.8% disagreeing with such a practice. Due to the comparative strength of objections, however, the mean score (M = 2.90) for the Likert-type item indicated overall disagreement.								Predictably, participants had concerns about using mHealth technology to hold people accountable for their behaviours. We found that 59.3% of participants agreed either partly or completely that doing so would damage the doctor–patient relationship, while 65.4% agreed either partly or completely that people would problematically change their behaviours if they were being monitored. Interpretations of what constitutes problematic changes in behaviour might differ between participants, but might include making behavioural changes for undesirable reasons or taking advantage of flaws in the monitoring devices to misrepresent health behaviours.<br><br>While the difference did not reach standard statistical significance on this small sample [t(80) = −1.96, p = 0.054], participants appeared slightly more likely to approve of using mHealth technology to monitor adherence to a lifestyle contract (M = 3.26)	N
Wearable Devices Are Well Accepted by Patients in the Study and Management of Inflammatory Bowel Disease: A Survey Study	2021	Digestive Diseases and Sciences	Hirten, R.P. and Stanley, S. and Danieletto, M. and Borman, Z. and Grinspan, A. and Rao, P. and Sauk, J. and Chang, L. and Arnrich, B. and B?ttinger, E. and Keefer, L. and Sands, B.E.	10.1007/s10620-020-06493-y	The aim of this survey study is to understand patient preferences and interest in wearable technology	Quantitative	USA	people with Inflammatory Bowel Disease	Four hundred and seventy-two patients were approached to participate in this study, with 400 subjects completing the survey, representing an 84.7% response rate (Table 1). The median age of subjects was 34 years (range of 18–80 years), and 51.5% of the cohort were women. Two hundred and eighty-one subjects (71.1%) obtained a bachelor's degree or higher. One hundred and sixty-six subjects (42%) had an annual household income of $100,000 or greater.<br><br>Two hundred and thirty subjects (57.6%) classified their IBD as Crohn's disease, 127 subjects (31.8%) classified their disease as ulcerative colitis, while 23 subjects (5.8%) classified their disease as indeterminate colitis. One hundred and thirty-two subjects (33.7%) classified their disease activity as being in remission, while 136 (34.7%), 85 (21.7%), and 39 (9.9%) classified their	Wearable Devices Management of Inflammatory Bowel Disease			Current/prior device users were more likely to choose the smart watch for research (p < 0.001) and routine management (p < 0.001), while those with disease in remission were more likely to pick jewelry for research (p = 0.004) and routine IBD management (p = 0.002). Smart eyewear was the least popular, chosen by only 5.9% and 4.6% of subjects for research and routine management, respectively.	Three hundred and sixty-six subjects (93.8%) reported that they would use a wearable device if it could provide important information about their health and help their doctor manage their IBD, with those with an income ≥ $100,000 compared to those with an income < $100,000 (p = 0.009), and those who are current/prior device users compared to those who have never used a wearable device (p = 0.002), being more likely to feel this way.		Both disease activity stratified based on remission versus active disease (p = 0.04) and current/prior device use (p = 0.04) were associated with a willingness to use smart watches at an increased frequency for research, while current/prior device use (p = 0.005) was associated with a willingness to use them at an increased frequency for routine disease monitoring.Subjects identified the three types of information they would be most interested in having their wearable device provide. These were stress monitoring (72%), information regarding exercise (63.3%), and information about sleep (58.2%) (Fig. 4). Those with active disease compared to those in remission were significantly more likely to choose stress monitoring as being important (p = 0.03).	The top three device-related factors that are most important when using wearable technology (Fig. 5) for research and routine IBD management were comfort (83.5%, 84.8%), ease of use (57.1%, 56.5%), and efficacy (32.8%, 34.2%), respectively. Current/prior device users were more likely to value comfort compared to those who never used wearable devices (p = 0.001). Subjects agreed/strongly agreed that updates about the information collected from the devices (85.6%) and feedback from the physician on the information collected (89.5%) would make them want to continue in a research study (Fig. 6). Overall, for all analyses, education level was not significantly associated with subject preference.			Three hundred and forty subjects (89.0%) believed that wearable devices can provide important information about their health that could help their doctors manage their disease.		HealthMode, Inc., and received research support from a Crohn's and Colitis Foundation Career Development Award (grant number 607934). BES discloses consulting fees from 4D Pharma, Abbvie, Allergan, Amgen, Arena Pharmaceuticals, AstraZeneca, Boehringer-Ingelheim, Boston Pharmaceuticals, Capella Biosciences, Celgene, Celltrion Healthcare, EnGene, Ferring, Genentech, Gilead, Hoffmann-La Roche, Immunic, Ironwood Pharmaceuticals, Janssen, Lilly, Lyndra, MedImmune, Morphic Therapeutic, Oppilan Pharma, OSE Immunotherapeutics, Otsuka, Palatin Technologies, Pfizer, Progenity, Prometheus Laboratories, Redhill Biopharma, Rheos Medicines, Seres Therapeutics, Shire, Synergy Pharmaceuticals, Takeda, Target PharmaSolutions, Theravance Biopharma R&D, TiGenix, Vivelix Pharmaceuticals; received honoraria for speaking in CME programs from Takeda, Janssen, Lilly, Gilead, Pfizer, Genetech; and received research funding from Celgene, Pfizer, Takeda, Theravance Biopharma R&D, Janssen. LK received research funds from Abbvie; is a consultant to Pfizer
Patient and Provider Perspectives on Remote Monitoring of Pacemakers and Implantable Cardioverter-Defibrillators	2021	American Journal of Cardiology	Fraiche, A.M. and Matlock, D.D. and Gabriel, W. and Rapley, F.-A. and Kramer, D.B.	10.1016/j.amjcard.2021.03.023	The use of remote monitoring technology for cardiovascular electronic implantable devices has grown significantly in recent decades, yet several key questions remain about its integration into clinical care. We performed semi-structured interviews of patients, clinicians, and device clinic technicians involved in clinical remote monitoring of cardiovascular implantable devices at our institution.	Qualitative	USA	cardio patient	Of the participants, 32% were female, 82% identified as white/Caucasian, 10% Asian, 4% Black, and 4% Hispanic. The average age of the interviewees was 61 (range 27–84 years). The majority of participants had college (46%) or some degree of post-graduate (43%) education training.	Remote Monitoring of Pacemakers and Implantable Cardioverter-Defibrillators	Most patient and physician interviewees articulated some lack of knowledge surrounding how remote monitoring functions. These knowledge gaps were highlighted in patients when participants were asked to compare remote monitoring technology to inpatient telemetry.						integrated into remote monitoring alerts. However, opinions how the alerts should be tailored to preferences were more variable. Some interviews felt that patients should be notified for all alerts, but it was the viewpoint of others that that only life-threatening alerts should be communicated:A struggle emerged between patients and clinicians surrounding expectations about remote monitoring as a 24-hour safety mechanism. Several patients described remote monitoring as akin to an alarm system whereas device clinic providers explicitly stated they purposefully attempt to educate patients to the contrary in order to empower them not to rely on remote monitoring.<br><br>Finally, cost transparency prior to device implantation was important to both patients and clinicians. Many interviewees supported formalized education in remote monitoring.	Patients also articulated limited memory of formal education regarding the device and how it functions.			Many participants, particularly patients, admitted a lack of understanding about how remote monitoring works, yet also felt that this was not concerning as long as the device did what it was designed to do.By contrast, several providers and a few patients did express concerns about knowledge deficiencies regarding remote monitoring.	UNDECLARED

European portuguese version of the user satisfaction evaluation questionnaire (useq): Transcultural adaptation and validation study	2021	JMIR mHealth and uHealth	Domingos, C. and Costa, P.S. and Santos, N.C. and P败o, J.M.	10.2196/19245	The purpose of this study was to conduct a transcultural adaptation of the User Satisfaction Evaluation Questionnaire (USEQ) into European Portuguese and validate the adapted questionnaire. Additionally, we aimed to provide information about older adults' satisfaction regarding the use of an activity tracker (Xiaomi Mi Band 2).	Quantitative	Portugal	older adults	Characteristics of the study participants (N=110).<br><br>Characteristics Values<br>Age, years, mean (SD) 68.41 (3.11)<br>Gender, male, n (%) 50 (45.5)<br>Education, years of formal schooling, mean (SD) 7.95 (5.38)<br>Mini-Mental State Examination, total score, mean (SD) 26.95 (2.00)<br>Geriatric Depression Scale, total score, mean (SD) 6.05 (4.58)	Wearable activity trackers			All participants reported user satisfaction experiences above "good," with 90% of all participants reporting excellent satisfaction with the device.	Furthermore, a significant correlation (r=−0.319, P=.001) between depressive mood and user satisfaction was noted; a higher score on the GDS (ie, higher depressive mood) was negatively associated with satisfaction with the device								N
Evaluation of smartwatch usage based on the perspective of expectation confirmation model (ecm), healthology, hedonic, and habits factors	2021	ICIC Express Letters	Adiyatma, W. and Oktavia, T. and Adhikara, C.T. and Gaol, F.L. and Hosoda, T.	10.24507/icicel.15.07.693	Smartwatch is a development in the field of wearable technology. Several studies have specifically discussed the characteristics that can increase continuance intention in using a smartwatch. The purpose of this study is to analyze the use of smartwatch hardware that is integrated with supporting applications based on the perspective of the Expectation Confirmation Model (ECM), healthology, hedonic, and habits factors to explain the intentions of the continuance of the smartwatch.	Quantitative	Indonesia	General public		smartwatch				this study found that the perceived usefulness of using a smartwatch will increase user satisfaction, so this can represent user expectations and it can increase the intentions of the continuance of the smartwatch. Additional analysis revealed that healthology and habits an important role in the intentions of the continuance of the smartwatch. The results of this study state that hedonic factors do not have a significant effect on continuance intention								N
Wrist-worn alcohol biosensors: Applications and usability in behavioral research	2021	Alcohol	Wang, Y. and Fridberg, D.J. and Shortell, D.D. and Leeman, R.F. and Barnett, N.P. and Cook, R.L. and Porges, E.C.	10.1016/j.alcohol.2021.01.007	In this paper, we describe three pilot projects examining the associations between reported drinking behavior and transdermal alcohol concentration (TAC) derived from a new, wrist-worn alcohol biosensor (BACtrack Skyn) in diverse participant groups and settings.	Mixed	Three unpaid healthy volunteers (researchers on our research team at the University of Florida	General public	young, healthy, male/female	Wrist-worn alcohol biosensors					The design of the Skyn was described positively by eight out of 12 participants with statements that it was lightweight, comparable to a watch or bracelet, and comfortable to wear. However, three participants commented on difficulty pairing the Skyn device with the app in order to upload data. One participant stated, "it was hard to upload data [and] pairing did not work well". Another experienced an error while uploading that resulted in a loss of data. Considering the app is still in active development, future software updates may fix some of these concerns.		The main recommendations for device improvement were to make the device waterproof (five out of 12 participants), to lengthen the battery life of the device and include a notification of battery level (four out of 12 participants), and to include notifications about the progress of data uploads and app pairing (four out of 12 participants).				[illegible] significant differences in ratings for the EMA and biosensor procedures on 12 of the 14 questions (see Table 2). The EMA procedure was rated significantly higher than the biosensor for agreement on one item ("Most people would learn it quickly"), although participants agreed with the statement for both devices, on average. Participants also significantly disagreed more with one item ("I felt self-conscious while using") in the case of EMA compared to the Skyn but, on average, participants disagreed with the statement for both devices. On average, participants also disagreed that either EMA or the Skyn influenced their behavior while drinking or made them drink less, suggesting that the devices can capture real-world drinking with minimal influence on behavior (i.e., reactivity) when participants are blinded to the TAC results.	UNDECLARED
Patient and family perceptions of telehealth as part of the cystic fibrosis care model during COVID-19	2021	Journal of Cystic Fibrosis	Jaclyn, D. and Andrew, N. and Ryan, P. and Julianna, B. and Christopher, S. and Nauman, C. and Powers, M. and Gregory S, S. and George M, S.	10.1016/j.jcf.2021.03.009	The purpose of this study was to understand how people with CF (PwCF) and families of PwCF experienced the shift to telehealthcare delivery.	Quantitative	USA	cystic fibrosis patient		monitoring at home	Most patients surveyed (72% adult, 77% pediatric) had no prior experience with telehealth and despite this, most (87% of all surveyed) denied any difficulty accessing their telehealth visit (Fig. 2).		Despite some concern with lack of in-person assessments, there was striking interest in using telehealth as part of future care. Most patients (53% adult, 69% pediatric) expressed an interest in having "some" visits be conducted virtually, with an additional smaller group (34% adult, 21% pediatric) preferring for "most" future visits to take place virtually (Fig. 5). Both groups of respondents (94% adult, 97% pediatric) were interested in technology that would allow for at-home assessment of pulmonary function (Fig. 5) to allay concerns discussed above.								[illegible] regarding lack of throat/sputum cultures in both the adult population (54% expressing at least moderate concern) and pediatric population (64% expressing at least moderate concern). Fifty-two percent of adult respondents and respondents on behalf of the pediatric population expressed concern regarding lack of pulmonary function testing. A majority of surveyed patients and parents/guardians reported low concern regarding the lack of other measures: lack of weight and lack of vital signs with physical exam (Fig. 4).	UNDECLARED
Assessing the Feasibility of a Novel mHealth App in Hematopoietic Stem Cell Transplant Patients	2021	Transplantation and Cellular Therapy	[illegible] T. and Ramalingam, S. and Romero, K. and Ren, Y. and Bohannon, L. and Arellano, C. and Jonassaint, J. and Miller, H. and Barak, I. and Fish, L.J. and Choi, T. and Gasparetto, C. and Long, G.D. and Lopez, R.D. and Rizzieri, D.A. and Sarantopoulos, S. and Horwitz, M.E. and Chao, N.J. and Shah, N.R. and Sung, A.D.	10.1016/j.jtct.2020.10.017	To first evaluate patient interest in mHealth apps, we designed and administered an interest survey to patients at the 2017 BMT-InfoNet reunion.	Quantitative	USA	Hematopoietic Stem Cell Transplant Patients	A total of 32 surveys were collected at the Bone Marrow Transplant InfoNet Symposium from HCT survivors and caregivers. The median age was 62 (IQR 26-76) and 55% of participants were male. Of those surveyed, 73 percent had graduated from college or received some postgraduate education, and 27% had a high school education or some college coursework in addition to a high school diploma. Smartphone usage was prominent among this group, with 79% of participants reporting that they used their smartphone multiple times per day.	mHealth App in Hematopoietic Stem Cell Transplant Patients			Patients rated satisfaction/acceptability at an average of 2.2 (out of 4).		Mean technical feasibility, was rated at 3.0 (out of 4). Patients rated ease of use at an average of 3.1 (out of 4).							Conflicts of Interest: Jude Jonassaint is an officer of Sicklesoft.
The use of continuous foetal monitoring technologies that enable mobility in labour for women with complex pregnancies: A survey of Australian and New Zealand hospitals	2021	Midwifery	Fox, D. and Maude, R. and Coddington, R. and Woodworth, R. and Scarf, V. and Watson, K. and Foureur, M.	10.1016/j.midw.2020.102887	The aim of this survey was to determine how often, and for whom, wireless and beltless technologies are being used in maternity settings across Australia and New Zealand and to identify any barriers to their uptake.	Mixed	Australia and New Zealand	women with complex pregnancies	[illegible] hospitals and 19% private/other hospitals, a close representation of the initial sample, which was made up of 78% public hospitals and 22% private/other hospitals. Of 187 Australian facilities contacted, 133 responded (71%). Similarly, 16 out of the 21 (71%) New Zealand facilities responded to the survey. A total of 74% of responding hospitals identified as obstetric led facilities (n=109), 14% identified as midwifery led facilities (n=20) and 13% identified as 'other' (n=19). Of the 19 facilities that identified as 'other', 79% stated that they had both obstetric and midwifery led models of care in their facility (n=15) and 21% indicated that their facility offered shared care between midwives and either a General Practitioner (GP) or Obstetrician (n=4).	continuous foetal monitoring technologies that enable mobility in labour for women with complex pregnancies			Several midwife respondents indicated that they felt wireless CTG monitoring was beneficial for women and expressed a desire for the wireless technology to be more readily available. Midwife respondents who had used both wired and wireless CTG wanted to see an increase in the availability and use of wireless monitoring:		Loss of contact<br>The second theme that emerged from the free text responses was 'Loss of contact'. Whilst midwife respondents liked the fact that wireless CTG technology provides freedom of movement for women, they reported frequent problems with maintaining contact with the foetal heart rate and uterine activity signals, particularly when women were mobilising in labour:<br><br>When loss of contact persisted, midwives reported that women were often asked to move into a position that would allow for clearer auscultation of the foetal heart rate, thus disturbing her labour:<br><br>Although supportive of women's needs to mobilise in labour, midwives were frustrated by the need to frequently readjust the CTG when loss of contact occurred.<br><br>In addition, frustrations around misplacement of transducers when they were accidentally discarded with bed linen and the high costs associated with replacing them were evident. Other issues that were identified included problems relating to battery life and recharging, damage to transducers and difficulty when monitoring women with increased body mass index (BMI).		Anticipating new technology<br>There was a strong sense that an improved product was required, in order to meet the needs of childbearing women in labour. It was anticipated that a new beltless product would relieve women of the discomfort they experienced when wearing elastic belts to hold the CTG in place:<br><br>It was also anticipated that new beltless foetal monitoring technology would reduce the need for midwives to reposition the transducers:<br><br>Midwife respondents were hopeful that new foetal monitoring technology would limit disruption to the labouring woman by reducing the need to reposition the monitoring device:			Most identified freedom of movement (n=124, 99%) and women's sense of choice and control (n= 108, 86%) to be positively impacted by wireless/beltless monitoring. Other common responses included normal vaginal birth rate (n=47, 38%), reduction in epidurals (n=38, 30%) and reduced length of labour (n=29, 23%).In addition to benefits for women, it was recognised that wireless CTG had benefits for midwives. For example, it was seen to reduce potential workplace hazards:		The first author, Dr Deborah Fox, has received honoraria for presenting workshops to clinical midwives on freedom of movement and positioning in labour and birth, from Philips Australia and New Zealand.
Satisfied or Frustrated? A Qualitative Analysis of Need Satisfying and Need Frustrating Experiences of Engaging With Digital Health Technology in Chronic Care	2021	Frontiers in Public Health	Wannheden, C. and Stenfors, T. and Stenling, A. and von Thiele Schwarz, U.	10.3389/fpubh.2020.623773	To explore how the use of a digital tool for self-monitoring and communication with healthcare satisfies or frustrates basic psychological needs across four spheres of user experience: interface, task, behavior, and life.	Qualitative	Sweden	people with chronic care	Demographic details of respondents (N = 122).<br><br>Variable Frequency Percent<br>Gender<br>Male 46 38%<br>Female 64 52%<br>Missinga 12 10%<br>Age in years<br>20–29 3 2%<br>30–39 6 5%<br>40–49 8 7%<br>50–59 31 25%<br>60–69 44 36%<br>70 or older 18 15%<br>Missinga 12 10%<br>DHT used forb<br>Hypertension 83 68%<br>Heart failure 10 8%<br>Mental illness 24 20%<br>Don't know/Other 13 11%<br>Missingc 4 3%<br>Use of functionalities<br>Activity tracker bracelet 105 86%	Digital Health Technology in Chronic Care										Autonomy satisfaction was identified in respondent descriptions of the ability to take more responsibility in monitoring their own health parameters (task), controlling their self-care (behavior), and taking themselves more seriously (life). Relatedness satisfaction was identified in respondent descriptions of improvements in getting and keeping contact with healthcare staff (tasks) and responsiveness of healthcare staff on patients' reported health observations (behavior).	Autonomy frustration concerned experiences of inflexibility in the technology (interface), feeling pressured to perform self-monitoring (task), feeling guilty if not engaging in health-promoting activities (behavior), and identification with one's diagnosis (life). Relatedness frustration concerned challenges in the communication and collaboration with healthcare staff induced by the digital health technology (task). Some described that they did not get answers to the questions they had posed in the chat. Others described challenges in knowing what types of questions they could pose and daring to write about their needs and experiences. Some also described the lack of personal contact by communicating digitally and the desire to meet healthcare staff face-to-face. Frustration was also expressed in relation to the experience of insufficient support and feedback from healthcare staff in one's self-care (behavior).	N
Perceptions and expectations of adults with type 1 diabetes for the use of artificial pancreas systems with and without glucagon addition: Results of an online survey	2021	Nutrition, Metabolism and Cardiovascular Diseases	Taleb, N. and Quintal, A. and Rakheja, R. and Messier, V. and Legault, L. and Racine, E. and Rabasa-Lhoret, R.	10.1016/j.numecd.2020.10.006	The first hybrid artificial pancreas (AP) systems with insulin only (mono-hormonal) have recently reached the market while next generations systems are under development including those with glucagon addition (bi-hormonal). Understanding the expectations and impressions of future potential users about AP systems is important for optimal use of this clinically effective emerging technology.	Quantitative	Canada	diabetes people	The 123 participants (54% women) had a mean (SD) age of 40.2 (14.4) years old, have had their diabetes for 23.7 (14.1) years and 81% of them had at least a collegial level of education (58% at university level, 23% collegial level, 17% high school level, 3% primary or other). The last glycated haemoglobin (HbA1c) was reported to be ≤ 7% (53 mmol/mol) by 37% of the participants, 7–8% (53–64 mmol/mol) by 34%, above 8% (64 mmol/mol) by 19% and was unknown by 10%. CSII and MDI were used by 58% and 42% of the participants, respectively. CGM was utilized permanently or occasionally by 43% of the participants and 37% were concomitant users of CSII and CGM (Fig. 1). The most challenging situations for glucose control are presented in Fig. 2 and anxiety levels about glucose control in Table 1.	adults with type 1 diabetes for the use of artificial pancreas systems with and without glucagon addition	Of the total respondents, 91% understood how AP systems work, 79% trusted AP systems with correct insulin dosing, 73% were willing to replace their current treatment with an AP system, 68% declared that physical activity would be facilitated by AP systems and 43% anticipated modifications in the way they plan their meals during AP system use.	After accounting for age, sex, education level, CSII use, CGM use and diabetes duration, the following associations were significant. 1) Better odds for understanding AP functioning were found for CSII users (OR (95% CI); 12.2 (1.2–119.9); p = 0.03). 2) Better odds for trusting the algorithm with correct insulin dosing were found in males (3.4 (1.1–10.1; p = 0.03). 3) Regarding willingness to replace current treatment modality with an AP system, additional variables were accounted for, notably AP function understanding, AP trusting with insulin dosing, hypoglycemia anxiety, hyperglycemia anxiety, the overall satisfaction with glucose control and the perceived effect on quality of life.	When asked about what the preferred type of treatment if all options were made available in the market while disregarding the issue of higher costs with the addition of glucagon, 71% chose bi-hormonal AP systems, 20% chose mono-hormonal AP systems, 5% chose treatment with insulin pumps, and 4% chose multiple daily insulin injections.	Of these factors, willingness to shift to an AP system was positively associated with expecting improvement in quality of life (10.9 (3.0–39.7); p < 0.001), with trusting AP systems with insulin dosing (3.6 (1.0–12.6); p = 0.047), and was negatively associated with the overall satisfaction over their current glucose control (0.2 (0.07–0.9); p = 0.04).				Participants expected the following negative aspects linked to bi-hormonal PA use, in order of importance: plausibly higher costs (58% of participants), the addition of a medication (17%), the complexity of the system with the need of two injection sites (24%), and body image (5%). Overall, 28% of participants did not expect negative issues arising from bi-hormonal AP systems.		Of the total participants, 80% would expect an improvement in their quality of life with an AP system. A reduction in overall anxiety related to diabetes management was expected by 63% of the respondents and in the need for snacks by 41%. Only 6% of participants did not think it would be useful. Participants thought that bi-hormonal AP systems would be most useful in these situations, in order of importance: physical activity (39% of participants), meals (35%), when not at home (33%), during the night (32%), and during the day (17%). More specifically, in terms of physical activity-related hypoglycemia, 89% thought bi-hormonal AP systems would be very useful or useful. Better glycemic control and quality of life with bi-hormonal AP systems in comparison to mono-hormonal AP systems were expected by 78% and 67% of participants, respectively (Table 2).	Anxiety about letting an intelligent algorithm control their glucose levels was expressed by 18% of participants while 88% affirmed that it would be important for them to be able to ignore or modify AP system recommendations when they want to. Only 4% felt anxious about letting AP systems collect their personal and glycemic data (Table 2).	[illegible] from the Canadian Diabetes Association, Astra-Zeneca, Eli Lilly, Cystic Fibrosis Canada, Merck, Novo-Nordisk, and Sanofi-Aventis. He has been a consultant or member of advisory panels of Abbott, Amgen, Astra-Zeneca, Boehringer, Carlina Technology, Eli Lilly, Janssen, Medtronic, Merck, Neomed, Novo-Nordisk, Roche, Sanofi-Aventis, and Takeda. He has received honoraria for conferences by Abbott, Astra-Zeneca, Eli Lilly, Janssen, Medtronic, Merck, Novo-Nordisk, and Sanofi-Aventis. He has received in kind contributions related to closed-loop technology from Animas, Medtronic, and Roche. He also benefits from unrestricted grants for clinical and educational activities from Eli Lilly, Lifescan, Medtronic, Merck, Novo Nordisk, and Sanofi. He holds intellectual property in the field of type 2 diabetes risk biomarkers, catheter life and the closed-loop system. RRL, LL and VM received purchase fees from Eli Lilly in relation with closed-loop technology. LL received consulting fees from Lilly and Dexcom, and received (to institution) research funding from Merck, Astra-zeneca and Sanofi. NT, AQ and ER do not have any competing interests
SQoF-WEAR Project. The Use of Wearable Devices to Identify the Impact of Stress on Workers?Quality of Life ?2021	Engineering Proceedings	7	https://www.scopus.com/inward/record.uri?eid=2-s2.0-85145202017&doi=10.3390%2fengproc2021007025&partnerID=40&md5=3793085958311df4cd907402a1a2e32b	Quality of Life	There is interest in the use of wearable devices to monitor and control stressors and their influence on the condition of workers. This study aims to identify the level of job stress and its influence on the quality of life of workers	Quantitative	Portugal	worker	36 workers participated in the study. A total of 58.3% belonged to the University of A Coruña, were women (53.8%), and were under 30 years of age (30.8%). Most of the participants (61.9%) had moderate work-related stress and considered that their stress level had increased somewhat to quite a lot due to the COVID-19 situation.	The Use of Wearable Devices to Identify the Impact of Stress on Workers' Quality of Life											questionnaires show that participants had slight difficulties falling asleep and low sleep quality (39.9%). By contrast, wearable reported that participants attained optimal sleep habits. Data from wearable Xiaomi Mi Band 5 show that participants walked on average 5780 steps. Regarding sleep, participants slept 60 min of deep sleep and 233 min light sleep.	N
Determinants of wearable healthcare technology usage in Vietnam	2021	CEUR Workshop Proceedings	Truong, V.-A.T. and Truong-Dinh, B.Q.		This study considers the antecedents of the actual use behavior of wearables users by inheriting the earlier models (i.e., TAPB, TPB) to determine which and how internal and external factors affect consumer's adoption of wearable devices in healthcare.	Quantitative	Vietnam	General public	61.5% female. The respondents in a group from 18 to 24 years old occupied 69.2% of the sample, while the group of 25 to 49 years old and the group over 50 accounted for 27.3% and 3.5% of the sample. 55.9% of the participants in the sample were students, while employees and self-employed accounted for 30.8% and 6.3%. Another occupation was 7% of the sample. The sample showed the majority of respondents currently use a smartwatch, accounting for 67.3%. Health tracking devices, heart rate monitor chest straps represent 16.4% and 13.3%, respectively. Other types of devices related to mask, sensors, etc., define 3% of the sample.	Wearable Healthcare Technology Usage				Except for knowledge reflected via information search, all remaining dimensions positively affect wearables adoption in healthcare. It means that the knowledge of health-related wearables is not needed to be the prerequisite of smart devices usage. This type of knowledge concerning with information search about healthcare wearables to manage user's health.								UNDECLARED
Use of in-home activity monitoring technologies in older adult veterans with mild cognitive impairment: The impact of attitudes andcognition	2021	Gerontechnology	Leese, M.I. and Dorociak, K.E. and Noland, M. and Gaugler, J.E. and Mattek, N. and Hughes, A.	10.4017/GT.2021.20.2.10.06	This exploratory pilot study incorporated both quantitative and qualitative approaches to examine mild cognitive impairment (MCI) and cognitively intact older adults?attitudes (i.e., usability, acceptability, digital readiness, barriers) and adherence to in-home technologies after undergoing 7 months of in-home activity monitoring.	Quantitative	USA	older adult veterans with mild cognitive impairment	The sample consisted of white (N = 30, 100%), primarily male (n = 28, 93.3%) military veterans (enlisted n = 23, 76.67%; officer n = 7, 23.33%) with an average age of 73.46 years (SD = 5.16) and 14.93 years of education (SD = 2.03). Average individual annual earnings fell in the range of $25,000–$34,999, with a normal distribution of income scores.	in-home activity monitoring technologies					[illegible] (e.g., "I don't like the watch because it's hard to see the hands on the clock and there are no numbers"). A second set of participants (n = 5) reported difficulties adjusting to the technology and incorporating it into their daily routine (e.g., "I had not worn a watch for the past few years so it has been difficult getting used to wearing one again"). An additional theme (n = 4) raised concerns about the potential impact of cognition on engagement with the technologies (e.g., "I must admit that the color tests on the SMART can be a bit confusing, even for someone who is intelligent and alert"). Finally, 2 participants reflected a limitation of the current study design being that researchers did not give participants' reports on their progress throughout the study (e.g., "I would be interested in my readings of movement over the course of the study").			Technology barriers were correlated with memory (r = .43, p = .018) and global cognition (r = .36, p =.049), such that those with better cognitive performance endorsed more technology barriers.				N
Attitudes towards a sensor-feedback technology in gait rehabilitation of patients after stroke	2021	Disability and Rehabilitation: Assistive Technology	Nieboer, M. and Jie, L.-J. and Willemse, L. and Peek, S. and Braun, S. and Wouters, E.	10.1080/17483107.2021.1936664	The main aim of this study was to assess attitudes towards a sensor-feedback technology in gait rehabilitation of patients after stroke.	Qualitative	Netherlands	people after stroke	[illegible] (Table 1), of whom 13 were male. Age ranged from 38 − 87 years and the time since stroke varied from 17 to 172 months. Cognitive capacity, measured using the Montreal Cognitive Assessment (MoCA) ranged from 15 to 27 points (max score is 30 points [Citation27]). Six participants lived alone and ten participants used a walking aid, being a cane (n = 6), a cane and/or walker (n = 3) or an ankle foot orthosis (n = 1). Two participants had no experience with a mobile phone before using the "Stappy" system. Based on the log data received after the interviews, the frequency of the use of "Stappy", varied. There were six participants who either did not use the "Stappy" system (n = 2) or used it only once (n = 4). Seven participants used "Stappy" 2–7 days (moderate users) and three participants used "Stappy" more than seven days during the two-week period (frequent	a sensor-feedback technology in gait rehabilitation of patients after stroke			The environment's response to the use of "Stappy" was predominantly positive<br><br>A number of participants also stated that they needed help from family. Participants who experienced impairments after stroke, needed help to attach and remove the sensors after training; "I have to wait for someone to remove the 'things' from my shoes (p15)". Some participants informed their own therapist about the support during training; "I have talked about this with my physiotherapist (p10)". Some suggested that they would have liked to use "Stappy" in an earlier stage of the rehabilitation; "It's a shame it wasn't available at the same time as the physiotherapy training (p11)".		Ease of use<br>Participants had a variety of experiences regarding the ease of use, ranging from getting acquainted with the system to experiencing difficulties using "Stappy".<br><br>Putting the sensors on shoes after a stroke was problematic for a number of participants;<br><br>Although most participants were used to having their own mobile device (mobile phone or smartphone), participants did not mind using an extra smartphone to receive feedback about their gait. In general, they appreciated the use of the interface of the app. As a result of the user-friendly interface, people did not experience any problems; "The app was easy to work with, it works intuitively (p5)".<br><br>Charging was generally without problems In general, the quality of the feedback was also positively appreciated. The audio voice that provided the feedback was experienced as pleasant, although some participants indicated that they would prefer to be able to choose a voice.	Participants were instructed to use "Stappy" for gait training purposes but there was a number of participants who were looking for ways to integrate training in their daily life walking activities. Participants stated that they would like to use "Stappy" more frequently as an assistant in daily life activities.		Pertaining to the participants' attitudes towards the technology, there were participants who indicated in general that they had "little interest in technology (d6)" or felt that they were "no longer fast enough" or "too lazy (p1)" to learn how to use "Stappy" during gait training.Participants also mentioned external factors that influenced the non-use of "Stappy" on a regular basis. Apart from "Stappy", participants indicated that the weather conditions negatively affected the occurrence of outdoor gait training "I am fearful to walk outside when it is dark (p1)" or "I will not leave the house when the temperature outside is too hot (p2)". Another participant indicated "It is slippery when it rains and then I don't dare (p15)".Other factors were purely personal and were mainly related to the participants' attitude towards going out for a gait training session. Not everyone experienced sufficient motivation to keep training on a regular basis after the rehabilitation period whether or not impeded by physical or mental limitations		First, participants stated that "Stappy" helped them to be consciously aware of the importance of being physically active with the goal of improving their mobility; Participants also mentioned the importance of repeating what they had learned in their physiotherapy sessions. A number of participants indicated that during the training period makes them prone to falling back into their old suboptimal walking pattern.		N

Wearables for residents of nursing homes with dementia and challenging behaviour: Values, attitudes, and needs	2021	Gerontechnology	Peeters, M.W.H. and Schouten, G. and Wouters, E.J.	10.4017/GT.2021.20.2.7.06	Challenging Behaviour (CB) occurs in up to 80% of nursing home residents with dementia. To identify the values, needs, and attitudes of multiple stakeholders regarding wearables in the care of people with dementia and CB.	Qualitative	Netherlands	people with dementia	consisted of community-dwelling people with dementia (n=7), their informal caregivers (n=5) and 2 case managers.	Wearables for residents of nursing homes with dementia and challenging behaviour			Generally, informal caregivers and nurses accepted the use of the wearable as a supportive technology in the care of people with CB and dementia.		Sensor design Stakeholder thought the size of the Empatica E4 was too big, worrying them that it might draw too much attention (and accompanied resistance). The design of the wearable was indicated as being not appropriate for people with dementia. The wristband is worn like a watch, but not recognizable as a 'classic watch'. It has to be tightened around the wrist, which is uncomfortable for people with vulnerable skin. Finally, the power button is on the outside of the watch, within reach of the person wearing it.		[illegible] indicated they want to receive written information about the technology and are willing to follow a workshop. User comfort Additionally, in order not to spend more time than necessary, they also indicated that they want the technology to be reliable (i.e., no malfunction) and easy to use. User comfort Participants empathized that a wearable sensor should fit comfortably and should not be experienced as an additional stimulus. Ideally, a sensor should be placed out of sight or, when this is not possible, have another function such as a classic watch. Furthermore, water resistance and small sizes where indicated as valuable properties. It was suggested that a dummy-wearable could be worn by the person with dementia, so the person was used to the wearable during the actual measurements.Customizability No participant could think of a design of a wearable sensor that would be suitable for all people with dementia. Small and flat wearable sensors in clothing were mentioned as a good option for people who do not take of their clothing during the day. As a general design, a patch (like a band aid, see Figure 2B) was estimated as most promising. In their opinion, however, a wearable should be modifiable for a person (like jewellery), or a person should be able to choose between different wearables.			measurements of a wearable sensor were considered to be useful for (in)formal caregivers, but not so much for people with dementia themselves.	One participant was concerned regarding possible false positives. Participants were worried about stigmatization. Both informal caregivers and the nursing staff were not concerned about privacy issues regarding the wearable, as they consider biomedical variables as de-individualized values.	N
Technostress from persuasion: Wearable users' stressors, strains, and coping	2021	International Conference on Information Systems, ICIS 2020 - Making Digital Inclusive: Blending the Local and the Global	Rieder, A. and Schache, K. and Vuckic, S. and Jung, R.		We conduct an exploratory study of wearable technologies' adverse effects on users based on twenty-five narrative interviews.	Qualitative	Switzerland	General public	Full sample # (%) Devices used1 # (%) N 25 (100) Fitbit 12 (48) Gender # (%) Apple 10 (40) Women 13 (52) Garmin 10 (40) Men 12 (48) Other2 4 (16) Occupation # (%) Use duration # (%) Students 9 (36) 6-12 months 4 (16) Professionals 16 (64) 13-24 months 8 (32) Age >24 months 13 (52) Mean Median Min Max 35.6 33 19 54	Wearable Users' Stressors						• Control: Controlling performance and/or health outcomes • Validation: Seeking approval for behaviors	Device-handling • Automated data management and evaluation • Goal management and rewards • Self-tracking • Social comparison features • Activity reminder	Tolerance • Unwillingness to adjust • Distancing • Rationalizing • Compensation • Gaming the system • Alternative validation			Related to technologi-cal properties: • Inaccuracy • Unreliability • Complexity • Inflexibility Related to transparen-cy and evaluation: • Over-transparency • Over-dependence • Discrepancy between feeling and data Emotional and cognitive strains: • Feeling restricted in usage • Feeling rejected • Demotivation • Lack of recognition • Guilt • Evaluation issues Behavioral strains: • Obsessive behavior	UNDECLARED
Satisfaction and acceptability of telemonitored home-based exercise in patients with intermittent claudication: Pragmatic observational pilot study	2021	JMIR Rehabilitation and Assistive Technologies	Cornelis, N. and Buys, R. and Dewit, T. and Benoit, D. and Claes, J. and Fourneau, I. and Cornelissen, V.	10.2196/18739	Current guidelines recommend supervised exercise training (SET) as a first-line treatment in patients with intermittent claudication (IC). SET has been shown to be more effective than home-based exercise therapy (HBET). The aim of this study is to assess patient satisfaction and acceptability of a structured HBET program using wearable technology and elastic band resistance exercises.	Quantitative	Unspecified	Patients With Intermittent Claudication	Out of 41 eligible patients, 21 (50% recruitment success) volunteered to participate (15 men and 6 women).	Telemonitored Home-Based Exercise in Patients With Intermittent Claudication			[illegible] range 4-5) with the HBET program. These results were reflected in high adherence to the prescribed walking sessions (GPS and logbook combined=mean 89%, SD 25; GPS only=mean 86%, SD 28), with 75% (15/20) of the patients completing all prescribed walking sessions. In contrast, patients were less compliant with resistance training (mean 85%, SD 22; 56% (9/16) completed all prescribed sessions and 20% (4/20) of patients did not return their logbook) and did not prefer this exercise alternative over conventional walking therapy (mean 2.65, SD 0.8; median 3, range: 1-5). Furthermore, it is important to note that participants perceived the home-based program as safe (mean 4.4, SD 0.6; median 4, range 3-5). Most participants also stated that they would re-enroll in the exercise program (mean 4.4, SD 0.5; median 4, range 4-5) and would recommend it to their peers (mean 4.5, SD 0.5; median 4.5, range 4-5). Qualitative reporting revealed that participants were positive about the option to visualize progression using the recorded training logs (n=2) or trigger to improve (n=2), personal guidance (n=2), and flexibility (n=2).		However, resistance training (n=7) and pain during sessions (n=2) were perceived as less enjoyable.							N
HRV and Stress: A Mixed-Methods Approach for Comparison of Wearable Heart Rate Sensors for Biofeedback	2021	IEEE Access	Umair, M. and Chalabianloo, N. and Sas, C. and Ersoy, C.	10.1109/ACCESS.2021.3052131	This paper introduces a mixed-methods approach to compare the data quality and user acceptance of the six most common wearable heart rate monitoring biosensors.	Mixed	Turkey	General public	In total, we recruited 32 healthy participants (22 Males and 10 Females, age =28.4±5.98 years, BMI =25.61±6.49).	Wearable Heart Rate Sensors for Biofeedback					Participants described wrist-worn devices such as Galaxy Gear 2, Empatica E4, and also the Polar OH1 armband as more wearable compared to other sensors. They described wrist-worn devices as comfortable and lightweight. All participants found these electrodes to be uncomfortable while taking off the skin<br><br>In terms of design and aesthetics, participants preferred the Samsung Gear S2 smartwatch over Empatica E4. They mentioned that besides offering sensing capabilities, smartwatches also provide other functionalities for everyday use. They described it as "appealing", "everyday objects", and "stylish". Participants also mentioned that smartwatches are more socially acceptable than other sensing devices, and they are more likely to wear them over the long term. Furthermore, participants regarded First beat bodyguard 2 and BITalino (r)evolution as medical devices. They mentioned that they would not like to wear it in public places and over longer times as they are obtrusive and would attract attention and lead other people to believe that the wearer has a medical condition.<br><br>Results mirror the qualitative analysis highlighting that participants' preference of wrist and arm-worn devices in terms of aesthetics, wearability and comfort, long-term use, and social acceptance, followed by Polar H10 and First beat bodyguard 2 and BITalino (r)evolution.							UNDECLARED
Perspectives and recommendations of individuals with tetraplegia regarding wearable cameras for monitoring hand function at home: Insights from a community-based study	2021	Journal of Spinal Cord Medicine	Bandini, A. and Kalsi-Ryan, S. and Craven, B.C. and Zariffa, J. and Hitzig, S.L.	10.1080/10790268.2021.1920787	Wearable cameras have great potential for producing novel outcome measures of upper limb (UL) function and guiding care in individuals with cervical spinal cord injury (cSCI) living in the community. To analyze feedback from individuals with cSCI regarding the use of wearable cameras to record daily activities at home, in order to define guidelines for improving the design of this technology and fostering its implementation to optimize UL rehabilitation.	Mixed	Canada	individuals with tetraplegia	Mean age ± SD 56.2 ± 7.4 1F 12M	wearable cameras for monitoring hand function at home			Most of the participants (61.5–69.3%) expressed little concern about having data of their daily life used by clinicians and researchers for monitoring hand function, regardless of the type of information being accessed (i.e. raw videos or summary measures of hand function, Fig. 1). Moreover, participants would be more comfortable wearing a first-person camera at home than in public. All participants agreed that it would be useful to let researchers and clinicians access information regarding hand use at home. However, participants reported lower agreement when asked if they would find it useful to access this information (Fig. 3).		Theme 1 – comfort and technical aspects<br>Several participants found the camera uncomfortable for periods longer than 30 min, mostly because of its weight and overheating on the forehead. Other negative aspects were the limited battery life (approximately 1.5–2 h) and the awareness that sound was also being recorded (Table 3, quotes 2–4).<br>Regarding the importance and difficulties of collecting and reviewing videos, 69.3% of participants considered this technology easy-to-use (statement 10, Fig. 4). The percentage of disagreement and neutral responses was higher when participants were asked if it was easy or important to review the videos before sharing them with researchers (statements 11 and 13, Fig. 4). Instead, all participants agreed that it was important to start and stop the recordings at any time (statement 12, Fig. 4).<br><br>Conflicting responses were obtained when exploring the interference of technology with routine (statements 14, Fig. 5). In fact, 53.8% of participants felt that collecting videos interfered with their routine, whereas the rest of them either disagreed or gave neutral response. Most of participants (61.6%) expressed concern for the presence of household members in the video (statements 15, Fig. 5).<br><br>Regarding the potential use of the technology if prescribed by a doctor or therapist, there was an overall agreement by the participants that they would use this technology if it were prescribed for recording specific activities at home. Conversely, participants showed less willingness to use it at home all the time for a few weeks as part of the rehabilitation process (Fig. 6).		Suggestions for improvement focused on potential strategies to alleviate some of the above issues, such as having lighter and less intrusive cameras that would be easier to don and doff, and that could conduct longer recordings to capture more natural behaviors. Extending battery life and avoiding the recording of sounds were also noted (Table 3, quotes 5–7).			There was wide consensus among participants on the fact that recording videos while conducting activities at home was the benefit of this technology. Participants believed that hand use information captured within a natural context would allow clinicians and therapists to improve their understanding of hand function in real world conditions, conduct better assessments, and optimize interventions (Table 6, quotes 1–3). Moreover, the possibility to quantify hand use over time was seen by some participants as another benefit for individuals with cSCI, who could use this technology as an incentive for pursuing hand rehabilitation, by checking their improvements over time (Table 6, quote 4).	Theme 2 – privacy<br>Participants identified several positive aspects that helped prevent privacy concerns and alleviated the worries of collecting video during daily routines at home, for example: the egocentric point of view of the camera, which did not show the user's face; the possibility to start and stop the recording at any time; and detailed orientation and instructions from the research team (e.g. avoiding or stop recording during certain activities or in public places) (Table 4, quotes 1–2).<br><br>Despite these aspects, major privacy concerns emerged from the interviews. The possibility to record other people's faces, especially if they did not consent to be part of the study, was concerning. Moreover, the possibility to capture objects that carry any sort of personal information (identity documents, phone numbers, etc.), and recording the audio were flagged as worrisome (Table 4, quotes 2–5).	SKR is the CEO of Neural Outcomes Consulting Inc. which is the manufacturer of GRASSP products. The other authors report no conflicts of interest.
Implantation of an innovative intracardiac microcomputer system for web-based real-time monitoring of heart failure: Usability and patients?attitudes	2021	JMIR Cardio	D'Ancona, G. and Murero, M. and Feickert, S. and Kaplan, H. and 鍋er, A. and Ortak, J. and Ince, H.	10.2196/21055	Heart failure (HF) management guided by the measurement of intracardiac and pulmonary pressure values obtained through innovative permanent intracardiac microsensors has been recently proposed as a valid strategy to individualize treatment and anticipate hemodynamic destabilization. The aim of this study was to evaluate the usability and patients?attitudes toward a new permanent intracardiac device implanted to remotely monitor left intra-atrial pressures (V-LAP, Vectorious Medical Technologies, Tel Aviv, Israel) in patients with chronic HF.	Quantitative	Worldwide	People with Heart Failure	Attribute Value<br>Age (years), mean (SD), range 67.0 (10.32), 49-86<br>Male, n (%) 17 (81)<br>Female, n (%) 4 (19)	Web-Based Real-Time Monitoring of Heart Failure					Patients seemed to be comfortable in starting the measurements, during the measurements, and understanding when the measurement was completed and that the belt had to be unlocked before reconnecting to the charger (Table 2 and Figure 5).There was a general trend for higher scores of usability during follow-up, with an increase in the score for overall comfort with using the technology and specifically with wearing and adjusting the measuring thoracic belt.							Vectorious Medical Technologies has supported the V-LAP trial and provided a grant to cover expenses related to the publication of this manuscript. GD has received consultancy fees from Vectorious Medical Technologies. The other authors have no conflicts of interest to declare.
A mobile application to monitor mode, content and duration of health navigation services for people living with HIV in Guatemala	2021	AIDS Care - Psychological and Socio-Medical Aspects of AIDS/HIV	Alvis-Estrada, J.P. and Davis, D.A. and Guevara, K.G. and Santa Luce, R.L. and Loya-Montiel, M.I. and Northbrook, S. and Barrington, C.	10.1080/09540121.2020.1734173	We designed a mobile application (app) for navigators to report their interactions with men who have sex with men living with HIV (n = 374), including: (1) mode of support; (2) content; and (3) duration. We assessed sociodemographic and behavioral characteristics of the study sample and calculated monitoring system indicators. We also conducted qualitative interviews with navigators (n = 7) and used thematic analysis to assess app acceptability and usability.	Quantitative	Guatemala	people with HIV	[illegible] 97.3% (n = 364) accepted participation in navigation and of those, 95.3% (n = 347) had at least one interaction with their navigator during the 12-month intervention period. The median age of navigated participants was 30 years (range: 18–63). Approximately three-quarters (74.9%) self-identified as gay. The most widely reported ethnic group was ladino or mestizo (89.2%). Most resided in the department (state) of Guatemala, almost half (45.8%) had at least some or completed university education, and the median monthly income was Q2936 or USD$381 (Table	A mobile application to monitor mode, content and duration of health navigation services for people living with HIV			useful		They generally found the monitoring system easy-to-use and a benefit to them and the project. However, navigators varied in terms of how and when they entered data into the system. Roughly half reported entering data on a daily basis. Others reported entering data on a weekly basis by reviewing call and message logs on their phones as well as their written notes. Another important finding was that navigators indicated they did not document all interactions in the app. For example, general remote greetings and check-ins were sometimes not documented as an interaction.<br><br>Navigators suggested the app could be improved by incorporating a drop-down list of participant ID's to avoid having to manually enter IDs, which was difficult for some and led to manual cleanup for the study team. They also requested that a copy of the reports they entered in the app be automatically emailed to them for their personal records, as they were unable to review what they had reported after submission.							N
Fertility and digital technology: narratives of using smartphone app 慛atural Cycles?while trying to conceive	2021	Sociology of Health and Illness	Grenfell, P. and Tilouche, N. and Shawe, J. and French, R.S.	10.1111/1467-9566.13199	We draw on in-depth, qualitative interviews, adopting a critical digital health studies lens (a sub-field of science and technology studies), to explore the experiences of cisgender women and partners with one such app, Natural Cycles, in the context of their daily lives.	Qualitative	UK	women	[illegible] 'users', all cisgender women, aged 24–43 (17 aged 30+, 1 aged 40+); three of whom described their ethnicity as African, Black, Caribbean or Black British; three as Asian or Asian British; two as mixed or multiple ethnic groups; and 18 as white (16 as white British). All women had a current partner; one of these partners was female and the remaining were male. Most (n = 17) were employed full-time and 'comfortable' or 'really comfortable' (n = 15) on their current income (a minority were part-time, self-employed, studying or retired; nine 'neither struggling nor comfortable' or 'struggling' on income; none 'really struggling').	smartphone app 'Natural Cycles'					We found that many women valued the technology as a 'natural', inobtrusive alternative to biomedical intervention, and a means of controlling and knowing their bodies, amid a dearth of fertility-related education and care. Yet this technology also intervened materially and affectively into the spaces of their lives and relationships and privileged disembodied metrics (temperature) over embodied knowledge. Meanwhile, app language, advertising and cost have contributed to characterising 'typical' users as white, heterosexual, affluent, cisgender women without disabilities. In the context of neoliberal shifts towards bodily self-tracking, technologies appealing as novel, liberating and 'natural' to individuals who can access them may nevertheless reproduce highly gendered reproductive responsibilities, anxieties and broader health and social inequalities.							N
Adoption of telemedicine applications among Saudi citizens during COVID-19 pandemic: An alternative health delivery system	2020	Journal of Infection and Public Health	Yamin, M.A.Y. and Alyoubi, B.A.	10.1016/j.jiph.2020.10.017	This study aims to investigate individual behavior towards adoption of telemedicine application based on wireless sensor network applications (WSN) during COVID-19 pandemic.	Quantitative	Saudi Arabia	General public	A research survey was conducted towards Saudi citizens in voluntary setting.	telemedicine applications			79.5% in individual behavior to adopt wireless sensor network application.	Findings of the structural model indicate that wireless sensor network application was jointly predicted by performance expectancy, social influence, effort expectancy, facilitating condition, task technology fit, awareness and self-efficacy and explained substantial variance								N
Parkinson抯 disease: current assessment methods and wearable devices for evaluation of movement disorder motor symptoms - a patient and healthcare professional perspective	2020	BMC Neurology	AlMahadin, G. and Lotfi, A. and Zysk, E. and Siena, F.L. and Carthy, M.M. and Breedon, P.	10.1186/s12883-020-01996-7	There has been a rising interest in wearable devices for evaluation of movement disorder diseases such as Parkinson disease due to the limitations in current clinic assessment methods such as Unified Parkinson Disease Rating Scale (UPDRS) and the Hoehn and Yahr (HY) scale. This study aims to identify the perspectives of healthcare professionals and patients linked to current assessment methods and to identify preferences, and requirements of wearable devices.	Qualitative	UK	people with Parkinson's disease	Data source<br>Gender Male : Female<br>Number of participants<br>Age (year) Mean ± SD (range)<br>PD Duration (year) Mean ± SD (range)<br>Focus Group 1<br>2 : 1<br>3<br>Focus Group 2<br>3 : 3<br>6<br>73.83 ±10.69 (56 - 88)<br>8.5 ±7.29 (2 - 24)<br>Focus Group 3<br>2 : 1<br>3<br>Interviews<br>1 : 3<br>4<br>57.75 ±6.29 (52-65)<br>NA	wearable devices for evaluation of movement disorder motor symptoms	When asked about wearable technology, most patients have not heard about or used wearable technology.		Following an explanation of the purposes of this technology as a part of this study, the majority of patients taking part in this study stated that they would be willing to use wearable technology and to be monitored 24/7		The role of aesthetics and design for acceptance and adoption<br><br>Following an explanation of the purposes of this technology as a part of this study, the majority of patients taking part in this study stated that they would be willing to use wearable technology and to be monitored 24/7, as long as the device is not invasive or on an undesired part of body (e.g., neck or ankle). Patients stated the preferred part of the body was the wrist, as one would wear a watch; this was echoed by every participant in this study.<br><br>None of the patients had any concerns about device visibility. Perhaps somewhat remarkably, they wanted the device to identify them as people affected by PD, so it might indicate to the community they may need help.		Patients and healthcare professionals want wearable technology that eases and refines treatments<br>There were some suggestions to other functionalities to the device, such as fall detection and medication reminders, as this information could help healthcare professionals and patients alike, as forgetting medications and falls are common in PD patients/older age generally [54, 75].<br>add a medication reminder option to the wearable devices and the applications as patients report facing issues with remembering taking the right medication on time. Additionally, patients also suggested a help call button would be useful on the device to summon an emergency contact when needed. Regarding wearable design aspects, there are a number of similarities between interviews and focus group discussions in terms of what patients would want, including for the device to be comfortable, non-invasive, waterproof, durable, small, and easy to fasten.				Issues related to technology such as violation of privacy, difficulty in learning how to use technology, fear and discomfort of using technology and lack of human interaction were not particularly prominent in the discussion. Except for one patient who thought that the fear or dislike of modern technologies (Techno-phobia) could be a barrier for many elderly people, which echoes healthcare professionals' perspectives.	N
User-centred assistive technology assessment of a portable open-area body weight support system for in-home use	2021	Disability and Rehabilitation: Assistive Technology	Kokkoni, E. and Galloway, J.C.	10.1080/17483107.2019.1683236	The purpose of this study was to determine the level of feasibility of an in-home application of a novel portable body weight support system (PBWSS), designed for community use.	Quantitative	Switzerland	Parents and their infants	Twenty parents and sixteen infants participated in the study. Both parents of four infants volunteered to participate in the sessions, and both responses were considered. The group consisted of 11 (six male) typically developing (TD) infants between the age of 8.2 and 11.8 months (M = 9.9 ± 1.2 months) and five (two male) infants with DS between the age of 12.4 and 19.7 months (M = 17 ± 3 months). The average height and weight of all infants was 74 cm (SD = 5.1) and 9.3 kg (SD = 1.6), respectively. All infants were able to independently maintain their head in midline while placed at different postural positions (prone and sitting), and crawl or scoot for a distance at least twice their body length. Exclusion criteria were a history of seizure disorder, non-correctable vision problems and/or other severe impairments or conditions that would make their participation difficult. For the DS group, a physician clearance was	a portable open-area body weight support system for in-home use			Parents' overall satisfaction of the device from both groups was combined as visual inspection of separate data did not show clear differences. First, most parents perceived the device to be safe with 19 out of 20 (95%) reporting the top two most positive items on the 5-point Likert scale ("Not at all" - "A great deal") (Figure 4(A)).		Second, 79% of parents scored the three most positive items on the 10-point Likert scale ("Strongly Disagree" – "Strongly Agree") for the ease of device use and 67% for the ease of device setup (Figure 4(B)). Lastly, 62% of parents scored the three most positive items on the 10-point Likert scale ("Strongly Disagree" – "Strongly Agree") indicating their infant was comfortable while using the device, and 73% of parents scored the same items indicating their infant's use of the device was convenient for them (Figure 4(B)).	Parents' responses to the need for and hypothetical use of the device were again considered separately for those of TD infants and those with DS. No parent of TD infants reported a strong need for having the device at home as none scored the top two most positive items on the 5-point Likert scale ("Not at all" - "Extremely") (Figure 5(A)). Five out of the 14 parents (36%) reported the mid-item ("Moderately"), and nine (64.3%) reported the last two items (including the last item-no need at all). In contrast, the majority of parents of the DS group (60%) reported the second most positive item (Figure 5(A)). The rest of the parents (40%) reported "Moderately" and "Slightly". No parent of the DS group reported the last item – no need for the device. Lastly, on the hypothetical question of how much they would use the device if they had it at home, the majority of parents in both groups (57% of parents of TD infants and 50% of parents of the DS group) reported 1–2 hours per day (Figure 5(B)).	Although, in general, the parents were satisfied with the device, a common suggestion was the need for modifications of the wearable harness. The fact that the harness is one size is a potential limitation to consider, especially in any follow-up long-term study, where body dimensions continuously change.			The majority of parents noted positive changes on their infant's behaviour. The responses from the two groups of parents (TD and DS) were considered separately (Figure 3(A)). Eight of the 14 parents (57%) of TD infants reported the top two most positive items on the 5-point Likert scale ("None" - "A great deal"). Five of the six parents (83%) of infants with DS reported the top two most positive items on the 5-point Likert scale ("None" - "A great deal"). No parents reported the last item that reflects a no change on the infant's behaviour ("None").<br><br>Figure 3. Parents' responses on infant's behavioural change. A. Parents' observations of the general behavioural change. B. Parents' responses on parental and infant enjoyment.The majority of parents observed and reported that their infant was happy while using the device. Parents' responses on their own and their infant's enjoyment are reported altogether (Figure 3(B)). With respect to infant enjoyment, 79% of parents scored the three most positive items on the 10-point Likert scale ("Strongly Disagree" – "Strongly Agree"), with 47% of parents reporting the highest level ("Strongly Agree").		N
Mobile health app with social media to support self-management for patients with chronic kidney disease: Prospective randomized controlled study	2020	Journal of Medical Internet Research	Li, W.-Y. and Chiu, F.-C. and Zeng, J.-K. and Li, Y.-W. and Huang, S.-H. and Yeh, H.-C. and Cheng, B.-W. and Yang, F.-J.	10.2196/19452	Chronic kidney disease (CKD) is a global health burden. Self-management plays a key role in improving modifiable risk factors. The aim of this study was to evaluate the effectiveness of wearable devices, a health management platform, and social media at improving the self-management of CKD, with the goal of establishing a new self-management intervention model.	Quantitative	Taiwan	Patients With Chronic Kidney Disease	The mean age of all participants was 51.22 years (SD 10.98) and 73.5% were men. The baseline characteristics of these participants are presented in Table 1. The patients with stage-2 CKD outnumbered those with stages 3 and 4. There were no differences in comorbidities between groups. The patients in the intervention and control groups had similar levels of serum creatinine. Most of the participants had a bachelor's degree, although more participants in the intervention group had a university degree compared with the control group.	Mobile Health App to Support Self-Management for Patients With Chronic Kidney Disease			The investigators provided individualized dietary suggestions online for intervention group participants. The most frequent suggestions included increasing the amount and types of vegetables and fruits, reducing protein intake, and adjusting calories. Most of the participants in the intervention group (19/25, 76%) gave positive feedback about the intervention. Six of the 25 (24%) participants in the intervention group felt that wearing the smart wristband was inconvenient							88% (22/25) reported better changes in dietary and exercise habits		N

Comparison of mobile health technology use for self-tracking between older adults and the general adult population in canada: Cross-sectional survey	2020	JMIR mHealth and uHealth	Jaana, M. and Par? G.	10.2196/24718	This study presents contributions to an underresearched area on older adults and mHealth technology use. The aim of this study was to compare older adults' use of mHealth technologies to that of the general adult population in Canada and to investigate the factors that affect their use.	Quantitative	Canada	Older Adults and the General Adult Population	Characteristics Older adult population, n=682, n (%) General population, n=3427, n (%) Total, N=4109, n (%) P value<br>Gender <.001<br>Male 400 (58.6) 1718 (50.1) 2118 (51.5)<br>Female 282 (41.3) 1709 (49.9) 1991 (48.5)<br>Regiona <.001<br>Atlantic provinces 56 (8.2) 237 (6.9) 293 (7.1)<br>Quebec 153 (22.4) 833 (24.3) 986 (24.0)<br>Ontario 265 (38.9) 1310 (38.2) 1575 (38.3)<br>Prairies 37 (5.4) 229 (6.7) 266 (6.5)<br>Alberta 50 (7.3) 387 (11.3) 437 (10.6)<br>British Columbia and territories 121 (17.7) 431 (12.6) 552 (13.4)<br>Highest education levelb,c .09<br>Primary and secondary school 181 (26.7) 758 (22.5) 939 (23.2)<br>College/CEGEP 177 (26.1) 972 (28.8) 1149 (28.4)	Mobile Health Technology Use for Self-Tracking Between Older Adults and the General Adult Population	When asked about their familiarity with smart devices/wearables for health, 82.7% of the older respondents (383/463) indicated having heard of these technologies, but only 32.1% of the older adults (123/383) were somewhat familiar or very familiar with them.		The overall satisfaction and intention to continue using mobile apps were favorable in both groups.	significant differences were observed in terms of region (P=.005 and P=.03 for traditional trackers and digital trackers, respectively, in Alberta) and chronic conditions (P<.001 and P=.003 for traditional trackers and digital trackers, respectively). Older adults living in the province of Alberta were 4.9 times more likely to be in the digital self-tracking group than the older adults living in other Canadian regions. Compared with older adults living with no chronic condition, older adults with chronic conditions were 0.4 times less likely to be digital self-trackers.	The overall satisfaction with and the intention to continue using these smart devices/wearables were high. The older adults who used wearables and smart devices reported being very satisfied (mean 4.1 on the 5-point Likert scale), perceived their devices to be user-friendly (mean 4.2), and had a firm intention to continue using them in the future (mean 4.2). Importantly, respondents perceived these devices as relatively useful.					About 6 out of 10 users said that they maintained or improved their health status by using digital self-tracking devices. Approximately 66% (31/47) of the older adult users of smart devices/wearables reported they were more informed or more knowledgeable about their health condition due to the use of these devices. For their part, 53% (25/47) of the older adult users said they felt more confident taking care of their health or more autonomous in the management of their condition. Interestingly, feeling less anxious about one's own health and having more informed discussions with a doctor were not perceived as major benefits among the older adult group.		N
A Method to Evaluate and Improve the Usability of a Robotic Hand Orthosis from the Caregiver Perspective	2020	Proceedings of the IEEE RAS and EMBS International Conference on Biomedical Robotics and Biomechatronics	Meyer, J.T. and Dittli, J. and Stutz, A. and Lambercy, O. and Gassert, R.	10.1109/BioRob49111.2020.9224376	In this study, the perspectives and performances of 15 participants (3 clinical, 12 non-clinical) placed in a caregiver role to prepare and don a wearable robotic hand orthosis on a wheelchairbound mock end-user were investigated.	Mixed	Swtherland	caregiver	yrs, 12 non-clinical and 3 clinical background; 9 female) were recruited and placed in a caregiver role. Participants with non-clinical background, herein referred as "non-clinical caregivers", were randomly assigned to one of two test groups A and B, whereas those with clinical background ("clinical caregivers") were manually allocated to the same groups. Group A (n = 10) consisted of 8 non-clinical and 2 clinical caregivers, group B (n = 5) of 4 and 1, respectively.	The RELab tenoexo is a fully portable, robotic hand orthosis for people suffering from hand sensorimotor deficits					The following anecdotes (translated from German) aim to highlight the value of RTA and to support the findings of the other results:<br>General impression: "I was stressed out seeing all the individual parts since I have no technical background."<br>Cables and plugs (tasks 4 & 5): "The plug is similar to a USB, but not exactly – this confused me."<br>Glove fitting (task 6): "It was quite hard to put on the glove.", "The glove did not seem to fit perfectly."<br>Receiving units (task 8): "I was not sure if the receiving unit was placed correctly."							N
Signal quality and patient experience with wearable devices for epilepsy management	2020	Epilepsia	and Glasstetter, M. and B鶴tcher, S. and Gregg, N.M. and Laks Nandakumar, A. and Joseph, B. and Pal Attia, T. and Viana, P.F. and Bruno, E. and Biondi, A. and Cook, M. and Worrell, G.A. and Schulze-Bonhage, A. and D顆pelmann, M. and Freestone, D.R. and Richardson, M.P. and	10.1111/epi.16527	Noninvasive wearable devices have great potential to aid the management of epilepsy, but these devices must have robust signal quality, and patients must be willing to wear them for long periods of time. Patients were asked to rate the usability and comfort of each device in several categories.	Quantitative	Australia	patient with epilepsy	The majority of respondents identified as fe-male, and the most represented age cohorts are 20-29 and 30-39 years of ag	wearable devices for epilepsy management				Being in the 60- to 69-year-old age group was significantly correlated with more negative usability responses (P < .05). Being in the 30- to 39-year-old age group was significantly correlated with more positive long-term comfort responses (P < .01).	The Empatica E4 was the preferred device more often than other devices. The Biovotion device had the most positive results for both ease of manipulation and usability, followed by the two wrist-worn devices, the Empatica E4 and GENEActiv. GENEActiv had the most positive results for both long-term comfort and comfort during sleep. The Byteflies sensor dots were most preferred for potential sei-zure prediction. However, all devices generally had positive responses.							E.N. and D.F. are employees of Seer Medical. M.C. and D.F. are shareholders in Seer Medical. No other authors have conflicts to declare. We confirm that we have read the Journal's position on issues involved in ethical publication and affirm that this report is consistent with those guidelines.
Implementing mobile health聆nabled integrated care for complex chronic patients: Patients and professionals?acceptability study	2020	JMIR mHealth and uHealth	de Batlle, J. and Massip, M. and Vargiu, E. and Nadal, N. and Fuentes, A. and Bravo, M.O. and Colomina, J. and Drudis, R. and Torra, M. and Pallis? F. and Miralles, F. and Barb? F. and Torres, G. and Balastegui, M.A. and Loxano, S.A. and Al-Jouja Llorente, L. and Sancho, T.A. and Ba驴res, E.A. and Vila, A.A. and Baron Burriel, J.M. and Claret, R.B. and Sague, A.B. and Ponce, E.B. and Franque, M.B. and Roig, ?B. and Santiago, C.B. and Sarramona, A.C. and Corretge, A.C. and Morales, J.C. and Gass? M.C. and Ortiz, F.C. and Del Pozo Garcia, D. and Morell, R.D. and Vilamaj?	10.2196/22136	Integrated care (IC) can promote health and social care efficiency through prioritization of preventive patient-centered models and defragmentation of care and collaboration across health tiers, and mobile health (mHealth) can be the cornerstone allowing for the adoption of IC. This study aims to assess the acceptability, usability, and satisfaction of an mHealth-enabled IC model for complex chronic patients in both patients and health professionals.	Quantitative	Spain.	complex chronic patient	The mean age was 78 (SD 9) years in both study arms, with patients in UC1 being 10 years older than patients in UC2 on average. Patients in UC1 had a higher burden of comorbidities than patients in UC2 (Charlson Comorbidity Index in UC1: mean 6.9, SD 2.1; UC2: mean 4.2, SD 1.6; P<.001). No statistically significant differences were found between patients in the usual care arm and patients in the IC arm, regardless of UC. Regarding the analyses of health care professionals, UC1 included 1 hospital case manager, 3 hospital physicians, 3 primary care case managers, 9 primary care physicians, and 6 primary care nurses, and UC2 included 1 hospital case manager, 1 hospital physician, 1 hospital surgeon, 1 hospital anesthesiologist, 2 primary care case managers, 9 primary care physicians, and 6 primary care nurses.	mobile health聆nabled integrated care for complex chronic patient			Patients and professionals' satisfaction with the deployed technology for IC is presented in Tables 2 and and3.3. Patients in UC1 reported an overall NPS score of +67% and patients in UC2 of +45%, whereas professionals involved in UC1 scored −25% and in UC2 −35%. Similarly, although the mean score for SUS was 75 (SD 19), the mean score for professionals was 61 (SD 20).		Table 4 reports on the actual use of technology by the patients experiencing IC. The actual use of the physical activity tracker was outstanding, with up to 77% (58/75) of patients having reported measures over 60 days out of 90. The ratio of times reported over times prescribed for the rest of sensors that could be proposed to patients ranged from 37% for oxygen saturation to 67% for weight. Finally, the use of the messaging function allowing patients to ask or answer requirements for or from the professional care team was high, with a median (P25-P75) of 19 (10.5-41) in UC1 and 10 (5-22) in UC2 over the 90-day intervention.							N
Immersive Virtual Reality and Gamification Evaluation on Treadmill Exercise by Using Electrophysiological Monitoring Device	2020	ACM International Conference Proceeding Series	Libriandy, E. and Arlini Puspasari, M.	10.1145/3429551.3429553	This research focused on evaluating the application of virtual reality technology based on gamification principle by electrophysiological monitoring device.	Quantitative	Indonesia	General public	students (N = 24; 11 women) with age ranged from 19 to 23 years old (M = 20.33, SD = 0.99). They all have normal category of Body Mass Index (BMI), normal or corrected-to-normal vision and absence of motor impairments. Participants were blind to the hypothesis of the experiment. The experiment was conducted at Ergonomic Centre Laboratory in the University of Indonesia. Figure 2 shows the participant wearing VR during treadmil exercise experiment.	Electrophysiological Monitoring Device			The subscales are competence, perceived choice, pressure tension and interest enjoyment that are below significant level of 0.05									UNDECLARED
Engagement, acceptability, usability, and preliminary efficacy of a self-monitoring mobile health intervention to reduce sedentary behavior in Belgian older adults: Mixed methods study	2020	JMIR mHealth and uHealth	Compernolle, S. and Cardon, G. and van der Ploeg, H.P. and van Nassau, F. and de Bourdeaudhuij, I. and Jelsma, J.J. and Brondeel, R. and van Dyck, D.	10.2196/18653	The aim of this study is to evaluate engagement, acceptability, usability, and preliminary efficacy of a self-monitoring-based mHealth intervention developed to reduce older adults' sedentary behavior.	Mixed	Belgium	Older Adults	A total of 36 older adults expressed interest in participation. Out of these 36 participants, 2 (6%) of them could not be reached to make an appointment for the first home visit and 4 (11%) decided to withdraw from the study after receiving detailed study information. Reasons for withdrawal were health problems (2/36, 6%), lack of time (1/36, 3%), and death of a spouse (1/36, 3%). As such, 30 older adults completed the baseline measurements. Out of these 30 participants, 2 (7%) of them were excluded, as baseline data showed that they did not fulfill the inclusion criteria (ie, they were not able to walk 100 meters without severe difficulties). In addition, 2 (7%) participants dropped out during the intervention period due to health problems (1/30, 3%) and lack of motivation (1/30, 3%). Consequently, posttest data were collected from 26 out of 28 participants (93% retention).	a Self-Monitoring Mobile Health Intervention			The participants mainly reported positive feelings, such as being motivated, surprised, and interested. Only a minority (3/28, 11%) indicated that they thought the intervention was not interesting and not helpful. There were mixed opinions on the preferred kind of feedback (ie, tactile vs visual). Some thought the vibrations were more useful, whereas others favored the visual information on the app.		The main themes that were identified were the design and the ease of use, wearing preferences, problems and solutions, the focus, and the perceived relevance. The intervention was considered easy to use, and most participants described the design as clear. The only remark on the design were the colors of the behaviors. Participants frequently cited that it would be more logical if sedentary behavior (ie, the behavior that should be limited) were displayed in red and the number of steps (ie, the behavior that should increase) in green. Participants expressed mixed preferences regarding the way to wear the device. Some participants (11/28, 39%) preferred to use the elastic band, whereas others (13/28, 46%) preferred to wear it in their pockets. Frequent problems that older adults, especially women, experienced included small or loose pockets, loss of the device, and the imprint of the elastic band on their clothes after wearing it. Out of the 28 older adults, 5 (18%) indicated that they used a handkerchief to ensure that the device was fixed in their pocket and could not flip over. Despite the fact that the aim of the intervention was to reduce sedentary behavior, only 2 participants (7%) mainly focused on the sedentary behavior information. Although the majority of older adults rated the intervention as highly relevant, some older adults were not convinced about the relevance. The most important reasons for limited perceived relevance were (1) the fact that they do not spend a lot of time sitting, or at least think they are not sitting a lot, and thus have no need to reduce their sedentary time and (2) the fact that they often see no other option but sitting to perform certain tasks.	They indicated that the main reasons to access the app were out of curiosity, to go through their day, and to see the impact of certain physical activities. Participants also emphasized that they consulted the app more frequently in the beginning of the intervention period, compared to the end of the intervention period.				Participants commonly reported that the intervention changed their thinking (ie, they became more aware of their sedentary behavior) but not their actual sedentary behavior. The latter result was supported by quantitative data derived from the activity monitor (see Table 2). Sitting and standing time were very similar at pre- and postmeasurements. There was a small improvement in steps of around 400 per day. This improvement was not significant, probably because of the small sample size.		N
Fluspot: Seasonal flu tracking app exploiting wearable IoT device for symptoms monitoring	2020	- 5th South-East Europe Design Automation, Computer Engineering, Computer Networks and Social Media Conference	Chloros, D. and Ringas, D.	10.1109/SEEDA-CECNSM49515.2020.9221843	The purpose of this paper is to explore the challenges in the development as well as the possibilities opened by applications that track the transmission of diseases and how IoT systems can be used to record symptoms.	Quantitative	Greece	General public		Seasonal flu tracking app exploiting wearable IoT device for symptoms monitoring			The vast majority of responders 93.5% reported that they consider the ability to monitor a disease spread via our system as useful or very useful. Encouraging results were received on our question on whether they trust such a system; more than two out of three, 69.3%, responded positively, albeit having some reservations.									UNDECLARED
How people with parkinson's disease and health care professionals wish to partner in care using ehealth: Co-design study	2020	Journal of Medical Internet Research	Wannheden, C. and Reven錺, ?	10.2196/19195	Worldwide, the number of people with Parkinson disease (PD) is predicted to double between the years 2005 and 2030. This study aimed to explore how co-care could be operationalized in PD care, supported by eHealth.	Qualitative	Sweden	People with Parkinson's Disease	The collaborative work was performed in a series of 4 half-day co-design workshops during May and June of 2016 [26,27]. Participants in the co-design workshops included 7 PwP (4 women) and 9 HCPs (4 women). Among the HCPs, 3 were registered nurses, 4 were neurologists, and 2 were registered physiotherapists.	eHealth			core eHealth functionalities and their expected values, describes 6 desired eHealth functionalities for supporting PD co-care between PwP and HCPs: (1) self-tracking, (2) previsit forms, (3) graphical visualization, (4) clinical decision support, (5) self-care recommendations, and (6) asynchronous communication. The second theme, individual and organizational constraints, describes constraints that need to be addressed to succeed with an eHealth service for co-care. Individual constraints include eHealth literacy and acceptance; organizational constraints include teamwork and administrative workload. The majority of the questionnaire respondents (31/37, 84%) perceived that they would benefit from an eHealth service similar to the demonstrated prototype. All prototype functionalities were rated as very important or important by the majority of respondents (ranging from 86% to 97% per functionality).									N
揑t's All Sort of Cool and Interesting呑ut What Do I Do With It??A Qualitative Study of Stroke Survivors' Perceptions of Surface Electromyography	2020	Frontiers in Neurology	Feldner, H.A. and Papazian, C. and Peters, K. and Steele, K.M.	10.3389/fneur.2020.01037	Stroke is one of the most common neurologic injuries worldwide. Over decades, evidence-based neurorehabilitation research and advancements in wireless, wearable sensor design have supported the deployment of technologies to facilitate recovery after stroke. Surface electromyography (sEMG) is one such technology, however, clinical application remains limited. The purpose of this study was to foreground the perspectives of stroke survivors to gain a better understanding of their experiences in neurorehabilitation, the technologies they have used during their recovery, and their opinions of lab-designed and commercially-available sEMG systems.	Qualitative	USA	people after stroke	To be included in the study, participants must have been over the age of 18 years, have the cognitive capability to consent for themselves, have had any type of stroke in the past or be the spouse or caregiver of the stroke survivor, and demonstrate proficiency communicating in English. Potential participants were excluded from the study if they were under the age of 18 years, not able to cognitively consent for themselves, or communicate proficiently in English. Stroke survivors with aphasia or limited communication capabilities were included in the study along with their spouses/caregivers. Ten participants across the Seattle metropolitan region completed the study, including eight stroke survivors and two spouses/caregivers. Of the stroke survivors, four were male and four were female, ranging in age from 49 to 78 years old (mean = 65 years), and ranging from 6 months to 12 years post-stroke	Surface Electromyography			Three major themes emerged from the data: (1) "Surface EMG has potential.but…"; (2) "Tracking incremental progress over days or years is important"; and (3) "Neurorehabilitation technology is cumbersome". These themes inform an overarching construct that sEMG could be valuable for stroke survivors, but the process and products must be simple and meaningful to their recovery in order to achieve greater uptake in both clinical and community settings during rehabilitation. Within these themes, the participants identified several key features of existing sEMG systems that were appealing, as well as features that presented barriers to use. Participants also offered innovative ideas and solutions for future iterations of sEMG technology and key insights for improved technology translation in healthcare.		Three themes emerged from the data: (1) "Surface EMG has potential….but…" highlights the recognition of sEMG as a valuable tool but reveals a lack of understanding and need for clear meaning from the data; (2) "Tracking incremental progress over days or years is important" highlights the persistence of hope and potential benefit of sEMG in detecting small changes that may inform neurorehabilitation practice and policy; and (3) "Neurorehabilitation technology is cumbersome" highlights the tension between optimizing therapy time and trying new technologies, managing cost, logistics and set-up, and desired technology features.							N
Raising the Responsible Child: CollaborativeWork in the Use of Activity Trackers for Children	2020	Proceedings of the ACM on Human-Computer Interaction	Oyg鵲, I. and Epstein, D.A. and Chen, Y.	10.1145/3415228	To provide an overall view of how families naturally use activity trackers towards collaborative management of family health, we systematically identified 9 trackers designed for children available on 4 consumer electronics retailers.	Qualitative	USA	children and parent	In total, we coded 2,628 user reviews, 1,789 of which were from consumer retailer websites and839 were from mobile application retailers	activity tracker			Our findings indicate children's and parents' collaborative use of these technologiesbeyond health and wellness. Parents state that their children enjoy practicing independence and rewards whilecontributing to family health management and daily life requirements.							teach their children to become more responsible for their health, daily tasks, and schedule.However, the current designs give limited agency on child's side and require parents' active participation forwearable-app coordination. For these reasons, they do not fully address parents' expectations in decreasingtheir workload. On the other hand, they have the potential to facilitate family interaction with challengesstructured around the data reported through trackers		UNDECLARED
Evaluation of 1-Year in-Home Monitoring Technology by Home-Dwelling Older Adults, Family Caregivers, and Nurses	2020	Frontiers in Public Health	Pais, B. and Buluschek, P. and DuPasquier, G. and Nef, T. and Sch髊z, N. and Saner, H. and Gatica-Perez, D. and Santschi, V.	10.3389/fpubh.2020.518957	This 12-month observational study aimed to evaluate a new in-home monitoring system among home-dwelling older adults (OA), their family caregivers (FC), and nurses for the support of home care.	Quantitative	Swtherland	in-Home Monitoring Technology by Home-Dwelling Older Adults, Family Caregivers, and Nurses	The baseline characteristics of the 21 included patients are shown in Table 1. The mean age was 85 years, half were men, and half received home care for more than 1 year. Patients took on average 9 drugs daily, 90% of patients were treated with 5 drugs or more per day, and 81% reported using a weekly pillbox to facilitate their drug intake. During the follow-up, 43% of patients were hospitalized at least once. Most patients were diagnosed with comorbidities, such as cardiovascular diseases, hypertension, dyslipidaemia, and diabetes.	in-Home Monitoring Technology			(DomoCare®) in helping staying at home (OA: 100%, FC: 85%), improving home care (OA: 100%; FC: 92%), preventing domestic accidents (OA: 85%; FC: 85%), and reducing family stress. The FC (69%) rated higher in-home sensors in improving quality of life compared to OA (54%) and nurses. More than half of OA (69%), FC (69%), and nurses (70%) considered ambient sensors can help reducing family stress. Overall, the majority of OA were satisfied with DomoCare®. Nurses tended to be less enthusiastic than OA, particularly regarding the technical (e.g., dimension, solidity) and practical settings of ambient sensors (e.g., ease of installation and use; Supplementary Figure 1).Regarding the wearable sensors (Figures 3, ,4),4), most of OA and FC rated higher activity tracker in helping staying at home (OA: 85%; FC: 77%) and improving home care (OA: 69%; FC: 77%) compared to ECG. Most nurses rated higher activity tracker in improving quality of life (60%) and reducing family stress (85%). Regarding the ECG, the majority of the OA (92%), FC (77%), and nurses (75%) underlined that ECG can help reduce family stress. More than half of OA, FC, and nurses were skeptical regarding the prevention of domestic accidents for the wearable sensors [activity tracker (OA: 54%; FC: 69%; nurses: 60%); ECG (OA: 62%; FC: 54%; nurses: 40%)]. Overall, OA and nurses were satisfied with the Activity tracker and ECG. However, nurses tended to be less enthusiastic than OA, particularly with ECG, reporting that wearable sensors should be made smaller, lighter, and more	The opinion tended to be more frequently favorable toward ambient sensors (76%; 95% CI: 61–87%) than toward Activity tracker (65%; 95% CI: 50–79%) and ECG (60%; 95% CI: 45–75%). On average, OA (74%; 95% CI: 46–95%) and FC (70%; 95% CI: 39–91%) tended to be more enthusiastic than nurses (60%; 95% CI: 36–81%).					Finally, the majority of OA, FC, and nurses reported that they would like to continue using in-home sensors in case of insurance reimbursement.	The majority of OA, FC, and nurses considered that in-home sensors (ambient and wearable) can help staying at home, improving home care and quality of life, preventing domestic accidents, and reducing family stress (Table 2).		PB and GD are employed by DomoSafety S.A., which is the manufacturer of the displayed ambient sensor system (DomoCare®). The remaining authors declare that the research was conducted in the absence of any commercial or financial relationships that could be construed as a potential conflict of interest.
Using telemedicine and wearable technology to establish a virtual clinic for people with Parkinson's disease	2020	BMJ open quality	Evans, L. and Mohamed, B. and Thomas, E.C.	10.1136/bmjoq-2020-001000	The Parkinson's Kinetigraph (PKG) is a wrist-worn device providing objective motor assessment, generating a report used by clinicians to optimise medication regimens. A pilot study of VC was designed using quality improvement methodology.	Quantitative	UK	people with Parkinson's disease	n 61<br>Mean age (years) 70.1<br>Age range (years) 50–86<br>Male/female (n) 44/17<br>Mean BKS 33.7<br>Mean levodopa equivalent dose (mg) 463.98	Using telemedicine and wearable technology to establish a virtual clinic for people with Parkinson's disease			Of questionnaire respondents, 89% were satisfied with VC (n=41). At VC, the clinician was able to make a treatment decision comparable to a face-to-face clinic in 79% of cases (n=48).		Reasons appointments were deemed unsuccessful included issues with the PKG, speech or hearing problems and complex phase of disease. VC appointments, including administration time, last on average 22 min. This compares to 20 min face-to-face appointments but these do not include administration time.							N
Usability Testing of a Sensor-Controlled Digital Game to Engage Older Adults with Heart Failure in Physical Activity and Weight Monitoring	2020	Applied Clinical Informatics	Radhakrishnan, K. and Julien, C. and O'Hair, M. and Baranowski, T. and Lee, G. and Allen, C. and Sagna, A. and Thomaz, E. and Kim, M.	10.1055/s-0040-1721399	Poor self-management of heart failure (HF) has contributed to poor health outcomes. Sensor-controlled digital games (SCDGs) integrates data from behavior-tracking sensors to trigger progress, rewards, content, and positive feedback in a digital game to motivate real-time behaviors. To assess the usability of an SCDG prototype over a week of game-playing among 10 older adults with HF in their homes.	Mixed	USA	Older Adults with Heart Failure	approached for the study, 18 (45%) consented to participate of which 8 dropped out of the study before enrollment. Reasons for declined participation included being not interested, moving to a different location, caregiver responsibilities, or lack of time. Usability of the SCDG was tested with 10 participants (50% females and 50% White; Table 2). Eight used iPhones, and two used Android phones. Six and five participants reported baseline exercise and weight-monitoring, respectively, according to below recommended guidelines which consists of exercising 4 to 6 times in a week and weighing 7 days in a week.	Sensor-Controlled Digital Game to Engage Older Adults with Heart Failure in Physical Activity and Weight Monitoring			Overall, 9 of the 10 participants reported that they would recommend the game to others with HF. According to patient 1 (P1), "it was very positive. It took me out of my element. I'm not used to playing games, so I found that comical and enjoyable." According to P3, "I thought the game was silly in that it was really too basic. But, I got into it and got motivated to walk."		enjoyable to play, but 1 participant (P10) who had not played digital games before reported that she did not understand the game.<br>The most common usability issues identified out of a total of 52 issues during the in-home testing with the first five participants were related to the user interface (UI; n = 15) and functionality (n = 9; Table 3). Issues included inability to locate the back button to return back to the main game from screens of other game features (n = 4) or missing healthy recipes and other incentives in the SCDG shop (n = 5). The font size of the word game was too small for the participants to maneuver the letters easily (n = 2). Some participants were unable to install the SCDG application as their phones were not updated with the latest software updates (n = 2).In response, we redesigned the back button to be more prominent at all game screens. Healthy recipes and local restaurant suggestions were featured more prominently in the SCDG's Shop. The mini games were adapted to be more age friendly by increasing the font size of the heart health relevant word game. Before beginning application installs, phones were checked for latest software updates. The improved version of the SCDG did not reveal any more errors during in-home testing with the remaining five participants.		Connecting game features with reality influenced participants' engagement with the SCDG. Nine participants liked the sound of birds in the background which reminded them of time they spent by a lake or in a forest.<br>Participants also suggested awarding points instead of deducting points for selecting healthy recipes in the shop.<br>Only three participants expressed satisfaction with the healthy recipes given as incentives in the game's shop or the game's local restaurant suggestions; a majority of the participants simply missed these game features.<br>Eight participants experienced issues with syncing weight with the game. Similarly five participants reported issues with consistently syncing the tracker with the game, and this adversely impacted their engagement.	Barriers to engage in behaviors included pain, onset of illness (three participants), and the short duration of 1 week for the usability assessment. This participant was among the participants who reported the lack of an instruction sheet as a barrier to engage with the SCDG.		Some participants found that the SCDG revealed their behavior patterns. For example, for P2, "the greatest takeaway was that I don't do enough exercise. I don't walk enough."		M.O. is the owner of Good Life Games, Inc. company which develops health games for hire provided the prototype game images for this study. The other authors do not report any conflict of interest in conducting this study.

Use of tablets and smartphones to support medical decision making in US adults: Cross-sectional study	2020	JMIR mHealth and uHealth	Langford, A. and Orellana, K. and Kalinowski, J. and Aird, C. and Buderer, N.	10.2196/19531	Tablet and smartphone ownership have increased among US adults over the past decade. However, the degree to which people use mobile devices to help them make medical decisions remains unclear. The objective of this study is to explore factors associated with self-reported use of tablets or smartphones to support medical decision making in a nationally representative sample of US adults.	Quantiative	USA	General public	Characteristics Use tablet/smartphone to help with medical decisions (n=944), weighted percentage (standard error) Do not use tablet/smartphone (n=1377), weighted percentage (standard error) Odds ratio (CI) P value Demographics Age (years) Overall .003 18-34 28.4 (3.4) 26.8 (2.1) Reference 35-49 31.2 (2.8) 30.1 (1.8) 0.98 (0.61-1.57) .93 50-64 31.5 (2.3) 28.8 (1.6) 1.03 (0.67-1.60) .89 65-74 7.0 (0.7) 9.4 (0.7) 0.70 (0.44-1.11) .13 75+ 2.0 (0.3) 4.8 (0.5) 0.38 (0.22-0.67) .001 Median age (years) 45.1 (0.96) 46.2 (0.70) 0.99 (0.99-1.00) .27 Gender Female 58.6 (2.4) 44.2 (1.8) Reference Male 41.4 (2.4) 55.8 (1.8) 0.56 (0.41-0.76) <.001 Race ethnicity Overall .16	Tablets and Smartphones to Support Medical Decision Making in US Adults				[illegible]								N
Using sensor-based technology for safety and independence ?the experiences of people with dementia and their families	2020	Scandinavian Journal of Caring Sciences	Malmgren F[illegible]ge, A. and Carlsson, G. and Chiatti, C. and Lethin, C.	10.1111/scs.12766	This study aimed to explore experiences, needs and benefits with using sensor-based technology for safety and independence in the homes of people with dementia and their family members.	Qualitative	Sweden	people with dementia and their families	Person with dementia (n = 9)Age in years, (mean, SD)81.6 (64–92)Female gender, n (%)5 (55)Cognitive function, (MMSE), 0–30 (mean, SD)19 (14–24)Deterioration (GDS),1–74 (3-5)Dementia diagnosis n (%)Alzheimer disease7 (78)Vascular dementia1 (11)Unknown1 (11)Informal caregivers (n = 21)Age in years, (mean, SD)59 (29–79)Female gender, n (%)12 (52)Cohabiting with the person with dementia7 (32)Relation to the person with dementiaHusband/wife6 (28)Adult child, son/daughter16 (72)Friend/Other0 (0	sensor-based technology for safety and independence - the experiences of people with dementia and their families	The majority of persons with dementia were notaware of the installed technology. However, regardingthe technology that they were aware of, they expressedeither that they did not understand it or that it made lifeeasier, for both themselves and their family members		[illegible]		[illegible]		[illegible]	[illegible]	[illegible]	[illegible]	[illegible]	N
Wearable devices and measurement data: An empirical study on ehealth and data sharing	2020	Proceedings - IEEE Symposium on Computer-Based Medical Systems	Alaqra, A.S. and Kane, B.	10.1109/CBMS49503.2020.00090	The development of medical applications and services is growing but is hampered by security and privacy concerns and a lack of trust by users. This interview study with 29 users of wearable devices reports perspectives on privacy concerns towards sharing of measured data in general, and measured stress data in the workplace.	Qualitative	Sweden and Ireland.	General public	In total, 29 interviews were carried out between Ireland (10) and Sweden (12). Seven (7) interviewees prefer not to acknowledge their country of residence (where the interview was conducted). The Age range of the interviewees is between 21–60. 30% of interviewees are in the Age Group 50–60, and 27% are in Age Group 31–40. 50% of those interviewed are under 40. We achieve an almost equal gender balance among our interviewees, with 16 (53%) Males and 12 (40%) Females. One interviewee prefers 'not to say' their gender. Of the interviewees, 19 (63%) describe themselves as having a technical background and 12 (40%) as athletes. 7 (24%) describe themselves as both athletic and as having a technical background.	Wearable devices and measurement data: An empirical study on ehealth and data sharing			[illegible]			[illegible]			[illegible]		[illegible]	UNDECLARED
Developing a mobile app for monitoring medical record changes using blockchain: Development and usability study	2020	Journal of Medical Internet Research	Sung, M. and Park, S. and Jung, S. and Lee, E. and Lee, J. and Park, Y.R.	10.2196/19657	Although we are living in an era of transparency, medical documents are often still difficult to access. Blockchain technology allows records to be both immutable and transparent. Using blockchain technology, the aim of this study was to develop a medical document monitoring system that informs patients of changes to their medical documents. We then examined whether patients can effectively verify the monitoring of their primary care clinical medical records in a system based on blockchain technology.	Quantitative	Korea	patient	Characteristic Hospital A (n=40) Hospital B (n=30) Total (N=70) P value Sex, n (%) .29 Male 9 (23) 3 (10) 12 (17) Female 31 (77) 27 (90) 58 (83) Respondent, n (%) .47 Guardian 19 (47) 18 (60) 37 (53) Patient 21 (53) 12 (40) 33 (47) Age groups (years), n (%) .40 19-20 0 (0) 1 (3) 1 (1) 20-29 5 (12) 1 (3) 6 (9) 30-39 22 (55) 17 (57) 39 (56) 40-49 12 (30) 10 (33) 22 (31) 50-59 1 (3) 0 (0) 1 (1) >60 0 (0) 1 (3) 1 (1) Occupation, n (%) .24 Information technology 1 (3) 0 (0) 1 (1) Office job 5 (12) 5 (17) 10 (14) Management 2 (5) 0 (0) 2 (3) Professional 4 (10) 3 (10) 7 (10)	a mobile app for monitoring medical record	The majority of participants stated that they were aware of bitcoin, whereas less than half were aware of blockchain. Similarly, respondents were more familiar with bitcoin than with blockchain. However, respondents had less trust in bitcoin than in blockchain. In terms of the recognition of medical record changes, participants tended to not allow changes in their medical records. Subsequently, participants stated a need for a medical record monitoring system. There was only one participant who reported having experienced medical document changes in the first questionnaire. The medical records were changed to correct the wrong information entered previously. In terms of digital literacy, most of the questions were answered as "good" digital literacy (Table 2).		The mean SUS score indicated "satisfactory" usability.										EL is the employer of Medibloc, Inc, and SJ is an employee of Medibloc, Inc. JL provided informatics consultation services to the Yonsei University School of Medicine and MediBloc Inc since 2018.
Continuous monitoring of vital signs in the general ward using wearable devices: Randomized controlled trial	2020	Journal of Medical Internet Research	Weenk, M. and Bredie, S.J. and Koeneman, M. and Hesselink, G. and Van Goor, H. and Van De Belt, T.H.	10.2196/15471	This study aimed to identify positive and negative effects as well as barriers and facilitators for the use of two wearable devices: ViSi Mobile (VM) and HealthPatch (HP).	Qualitative	Netherlands	patients	Gender, n (%) Male 18 (60) 22 (73) 20 (67) Female 12 (40) 8 (27) 10 (33) Age (years), median (range) 63 (26-76) 56 (27-88) 62 (34-77) Measurement period (days) of participation in the study, median (range) 3 (1-4) 3 (1-5)	Continuous Monitoring of Vital Signs in the General Ward Using Wearable Devices					[illegible]	[illegible]		[illegible]		[illegible]	[illegible]	N
Experiences with wearable activity data during self-care by chronic heart patients: Qualitative study	2020	Journal of Medical Internet Research	Andersen, T.O. and Langstrup, H. and Lomborg, S.	10.2196/15873	This study aimed to contribute to understanding how patients with a chronic disease experience activity data from consumer self-tracking devices related to self-care and their chronic illness.	Qualitative	Denmark	Chronic Heart Patients	Of the 27 patients, 25 patients were male (93%), and 2 patients were female (7%); the average age was 57 years. The sample largely reflected the demographic profile of ICD patients in Denmark in 2017, when 18% of patients were female and the majority of procedures were carried out on patients aged 55-74 years [42].	Wearable Activity Data During Self-Care by Chronic Heart Patients						[illegible]						N
Perceived acceptability of wearable devices for the treatment of mental health problems	2020	Journal of Clinical Psychology	Hunkin, H. and King, D.L. and Zajac, I.T.	10.1002/jclp.22934	This study examined the potential acceptability of wearable devices (e.g., smart headbands, wristbands, and watches) aimed at treating mental health disorders, relative to conventional approaches.	Quantitative	Australia	mentalhealth help-seekers	Age44.63 ± 15.2518–34144 (33.7)35–54150 (35.1)55+133 (31.1)Gender male184 (43.1)EducationDiploma or below177 (41.5)Bachelor degree178 (41.7)Postgraduate degree72 (16.9)Relationship statusSingle/divorced/separated156 (36.5)Married/committed relationship271 (63.5)Household income<$35,000112 (26.2)$35,000–$65,00092 (21.5)$65,000–$105,000103 (24.1)>$105,000120 (28.1)Socioeconomic disadvantage1019.11 ± 56.40Rural/remote124 (29.0) A similar proportion of males and females over abroad range of ages (18–78) responded although those over the age of 65 were less represented. Participants werepredominantly either professionals (37.9%) or not formally employed (e.g., students, retired, or unpaid	wearable devices for the treatment of mental health problems	Around two-fifths (40.7%) of respondents initiallyreported awareness of wearable devices for mental health. However, participants later reported relatively lowknowledge of the nature of these devices in the context of a description of them during the actual survey. Whilemore than a fifth of respondents indicated that they presently used other types of wearable devices, such as fitnesswearables, only eight respondents reported that they currently used a wearable device for mental health or well-being.		Few respondents indicated [illegible] whereas around two-fifths [illegible] in using such a device	[illegible]								UNDECLARED
Physical Activity Surveillance Using Wearable Activity Monitors: Are US Adults Willing to Share Their Data?	2020	American Journal of Health Promotion	Hyde, E.T. and Omura, J.D. and Fulton, J.E. and Weldy, A. and Carlson, S.A.	10.1177/0890117119900587	Wearable activity monitors (wearables) have generated interest for national physical activity (PA) surveillance; however, concerns exist related to estimates obtained from current users willing to share data. We examined how limiting data to current users who are willing to share data associated with PA estimates in a nationwide sample.	Quantitative	USA	General public		Wearable Activity Monitors			from 40.1% (95% CI: 33.[illegible] CI: 69.8-81.8) for their he[illegible] users, 81.2% (95% CI: 75[illegible] with at least 1 of the peop[illegible] multiple people or organi[illegible] was 62.2% (95% CI: 58.9[illegible] 80.7) among current wear[illegible] current users willing to sh[illegible] (Figure 2). When examini[illegible] or organizations with who[illegible] prevalence of being active [illegible] 75.2% for their health-car[illegible] 80.1% for their health ins[illegible] group (data not shown).										N
Patients?measurement priorities for remote measurement technologies to aid chronic health conditions: Qualitative analysis	2020	JMIR mHealth and uHealth	Simblett, S. and Matcham, F. and Curtis, H. and Greer, B. and Polhemus, A. and Nov[illegible], J. and Ferrao, J. and Gamble, P. and Hotopf, M. and Narayan, V. and Wykes, T.	10.2196/15086	Remote measurement technology (RMT), including the use of mobile phone apps and wearable devices, may provide the opportunity for real-world assessment and intervention that will streamline clinical input for years to come. The aim of this study was to conduct a consultation exercise on the clinical end point or outcome measurement priorities for RMT studies, drawing on the experiences of people with chronic health conditions.	Qualitative	UK	people with chronic health conditions—MDD, MS, or epilepsy	[illegible] 28 to 65 years of age, took part in three focus groups; 67% (16/24) of participants were women. Of the 24 participants, 16 (67%) returned for a further member-checking session to verify the findings. This meant that six focus groups were run in total. There was a similar distribution of men and women across the focus groups. However, participants with a history of MDD were, on average, slightly older. The time spent living with the chronic health condition varied; the people with MS had, on average, been living with their condition for the shortest amount of time.	Remote Measurement Technologies to Aid Chronic Health Conditions						[illegible]						N
Using geocaching to promote active aging: Qualitative study	2020	Journal of Medical Internet Research	Fornasini, S. and Dianti, M. and Bacchiega, A. and Forti, S. and Conforti, D.	10.2196/15339	This study aimed to investigate the effectiveness of activities combining geocaching and self-tracking technologies to promote active aging, evaluate the usability of technologies, and explore the ways in which technologies have been integrated in the organizational context under examination to determine the user experience of participants.	Mixed	Italy	older adults	Demographics Number of participants Age (years) 60-70 6 71-80 7 Above 80 1 Gender Male 1 Female 13 Level of education Primary school N/Aa Secondary school 3 Tertiary school 7 Degree 4	Using Geocaching to Promote Active Aging						[illegible]						N

Comparing the usability and acceptability of wearable sensors among older Irish adults in a real-world context: Observational study	2020	JMIR mHealth and uHealth	Keogh, A. and Dorn, J.F. and Walsh, L. and Calvo, F. and Caulfield, B.	10.2196/15704	This study aimed to compare multiple wearable sensors in a real-world context to establish their usability within an older adult (>50 years) population.	Mixed	Ireland	Older adults	Gender (n)<br>Male 5<br>Female 3<br>Age (years), mean (range) 62 (53-72)<br>Level of education (n)<br>Third level 3<br>Secondary level 4<br>Primary level 1<br>Employment status (n)<br>Retired 4<br>Employed 4<br>Experience with wearable devices (n)<br>Yes (current or past) 6<br>No 2	Wearable Sensors	Six participants reported feeling comfortable or very comfortable using technology. Three were wearing an activity tracker, while the remaining three had worn them in the past. The final two participants rated their technology comfort levels as medium, with no previous experiences of using wearable devices.		The median score for all devices on the acceptability questionnaire was 3.5 (0.5; 3.2-3.6).		The median score for all devices on the SUS was 57.5 (IQR 12.5; min-max=47.5-63.8) out of a possible score of 100. None of the tested devices were deemed to be good by participants, as all seven achieved scores of less than 68 (30). Actibelt achieved the highest median result of 63.8 (IQR 12.5; min-max=47.5-67.5), while Hexoskin achieved the lowest median result of 47.5; min-max=37.5- 57.5 (Table 3). The results for all of the devices fall between the 10th and the 30th percentile, meaning that all were considered below average. Comfort of Devices Participants believed that wrist-worn sensors were the most versatile and easy to use, and therefore, the most suitable for long-term use. Ease of Use of Devices The devices that required little to no interaction from participants were considered the easiest to use (ie, Actibelt, Actiwatch, and Actigraph GT9X Link). The devices most difficult to use were also those that were the least favored. Specifically, Hexoskin was considered as an excessive burden on participants, as it required users to moisten the chest sensors within the vest frequently (every 15-20 min) to capture the heart and breathing rate data accurately.	[illegible] (1.0; 3.6-5.2) on the 7-point Likert scale. No device achieved very high results (Table 3). Hexoskin was the only device to score below the midpoint of Likert scale (3.6; 3.4-4.5), suggesting that participants would not be autonomously motivated to wear this device.Likelihood of Wearing a Device During a Trial Participants agreed that the purpose of use is a key enabler for long-term compliance. These views were particularly noted by those not currently wearing an activity-tracking device. Although these participants explained that they did not personally feel the need to track their own activities, they suggested that they would not object to wearing a device for longer periods (ie, 8-12 weeks). For most devices, participants reported that they would only wear them only if it was necessary, suggesting that their use of these devices would be born out of compliance rather than a specific, intrinsically motivated intention				Perceived Usefulness of Devices For the majority of participants, the best devices were those they felt they received the most feedback from (ie, Wavelet, Biovotion). Nonetheless, participants were able to understand how these devices may still be valuable to others, including clinicians and researchers, and thus, were prepared to wear these devices in the name of science:		JD, FC, and LW work for Novartis.
A Qualitative Examination of Stay Quit Coach, A Mobile Application for Veteran Smokers with Posttraumatic Stress Disorder	2020	Nicotine and Tobacco Research	Herbst, E. and Mccaslin, S.E. and Hassanbeigi Daryani, S. and Laird, K.T. and Hopkins, L.B. and Pennington, D. and Kuhn, E.	10.1093/ntr/ntz037	Smoking is a lethal public health problem that is common in US military veterans, particularly those with posttraumatic stress disorder (PTSD). Mobile applications (apps) to promote smoking cessation are a scalable and low-cost approach that may facilitate treatment engagement. This qualitative study examined the acceptability, user experience, and perceptions of a smoking cessation app, Stay Quit Coach (SQC), when incorporated into evidence-based smoking cessation treatment. US military veterans with PTSD who smoked at least five cigarettes per day for 15 of the past 30 days and stated an interested in cessation were eligible to participate.	Qualitative	USA	Veteran Smokers With Posttraumatic Stress Disorder	Of the 93 veterans screened for the study, 39 were eligible. Eleven (28.2%) of those eligible declined due to either geographic distance or time commitment of office visits; seven (17.9%) did not respond to outreach; one (2.6%) was relocating; and 20 (51.3%) enrolled in the study. Participants ranged in age from 24 to 68 years (M = 40.18, SD = 15.97). One participant (5.9%) reported Hispanic ethnicity. Three (17.6%) identified as Asian, five (29.4%) as black/African American, one (5.9%) as Pacific Islander/Native Hawaiian, five (29.4%) as white, and two (11.8%) as more than one race. Additional demographic and baseline characteristics of the enrolled sample are shown in Table 1. All participants chose to use their own mobile devices. Nine (45.0%) used an iPhone device and 11 (55.0%) used an Android device. Seventeen (85.0%) of the 20 participants completed all study visits and completed the qualitative	A Mobile Application for Veteran Smokers With Posttraumatic Stress Disorder					[illegible] moderately high comfort level with mobile technology. Younger participants were significantly more likely to view mobile technology as compatible with their beliefs and preferences (r = −.52, p = .033). Age was not significantly correlated with ratings of relative advantage (r = −.32, p = .219), complexity (r = −.33, p = .193), or future use intentions (r = −.20, p = .442).<br>Positive Features of the App<br>The features of the app described as most helpful were the psychoeducational information on PTSD and smoking (58.8%, n = 10 endorsed as helpful) and the money-saved calculator (52.9%, n = 9 endorsed as helpful). Both the information within the app as well as the money-saved calculator were perceived as sources of reinforcement, motivation, and distraction for users, which they reported were helpful techniques to overcome cravings and urges to smoke. The calculator was described by some as a helpful and motivating way to visualize their progress.<br><br>As for the educational information within the app, participants were not only able to learn more about the actual effects of smoking, but many even used it to distract themselves from an urge by reading about all the negative aspects of smoking. Nearly one-third (29.4%, n = 5) of participants also noted that the reminders of their personal reasons to quit, self-scheduled motivational messages delivered as push notifications (29.4%, n = 5), and tools for coping with stress and negative emotions (23.5%, n = 4; eg, developing coping plans for specific triggers, the controlled breathing exercise) as helpful.	The most frequently described reason for using the app was to reinforce their smoking cessation efforts (52.9% of participants). Several participants specified that the motivational text messages and push notifications as well as the money-saved calculator (money saved after quitting) were helpful sources of reinforcement and motivation.<br><br>Nearly half (41.2%, n = 7) of participants expressed using the app to address tobacco cravings and urges, and 29.4% (n = 5) described using the app as a means of distracting themselves when triggered. Participants used the app to distract themselves and cope with urges and cravings to smoke by using the breathing tool, looking through the information provided, or giving them something to do with their hands.	Suggestions for Improving the App<br>The most frequent recommended changes pertained to improving features that increase the level of engagement and interactivity with the user. Six participants (35.3%) suggested that the app be modified to include the ability to create a unique profile. Suggestions for the profile consisted of being able to access personal motivators and triggers that had been added by the user, being able to share progress with others through texts or social media if desired, and more opportunities for social interaction and social support within the app. Suggestions for increasing user control included the increased ability to customize frequency and timing of motivational reminders, as well as an option to personalize features on the home page.<br><br>Other participant suggestions included app enhancement by incorporating content such as news updates or current events news feed, ability to store data within "the cloud," incorporating games or other features for distraction, and simplifying the language. Increased ability to track ones use of coping tools and cigarette use, as well as other tobacco products (eg, chewing tobacco and electronic cigarettes [e-cigarettes]), to monitor and detect patterns in craving onset, were also noted as potential improvements by several participants. Some also suggested that graphs be generated to visually represent the user's progress in decreasing cigarette consumption.	The most commonly cited barriers to using the app were issues with using the money-saved calculator while reducing cigarette use (eg, unable to use this feature unless one has already quit smoking; 35.3%, n = 6) and the timing of reminders and notifications (eg, inability to adequately customize timing of the notifications; 29.4%, n = 5). A smaller number (23.5%, n = 4) believed that they lacked the technological skills necessary for comfortably using the app; these participants expressed they would have benefitted from additional guidance or in-app instructions on how to use the different app features.<br>concern of privacy			Two participants (11.8%) also noted that the app did not allow for enough privacy and suggested that the app logo depicting a "stop smoking" icon and/or name be revised to be more discreet when displayed on someone's phone screen.	N
Early detection of mild cognitive impairment with in-home sensors to monitor behavior patterns in community-dwelling senior citizens in Singapore: Cross-sectional feasibility study	2020	Journal of Medical Internet Research	Rawtaer, I. and Mahendran, R. and Kua, E.H. and Tan, H.P. and Tan, H.X. and Lee, T.-S. and Ng, T.P.	10.2196/16854	Dementia is a global epidemic and incurs substantial burden on the affected families and the health care system. A window of opportunity for intervention is the predementia stage known as mild cognitive impairment (MCI). Individuals often present to services late in the course of their disease and more needs to be done for early detection; sensor technology is a potential method for detection. The aim of this cross-sectional study was to establish the feasibility and acceptability of utilizing sensors in the homes of senior citizens to detect changes in behaviors unobtrusively.	Quantitative	Singapore	older adults	[illegible] (n=21) Mild cognitive impairment (n=28)<br>Age (years), mean (SD) 73.0 (5.3) 75.1 (6.3)<br>Gender, n (%)<br>Male 7 (33) 9 (32)<br>Female 14 (67) 19 (68)<br>Years of education, mean (SD) 7.0 (4.0) 4.5 (3.9)<br>Employment (currently employed part time), n (%) 8 (38) 2 (7)<br>Housing type—Housing Development Board 1-2 room flat, n (%) 14 (67) 20 (71)<br>Marital status, n (%)<br>Never married 8 (38) 10 (36)<br>Separated/divorced 7 (33) 9 (32)	In-Home Sensors to Monitor Behavior Patterns in Community-Dwelling Senior Citizens in Singapore			A total of 83% (41/49) of the participants gave positive feedback at the study conclusion.		Some negative feedback included needing to charge the wearable, worrying about electricity costs, and finding the water usage sensor inconvenient. (2) Not used to the blinking light. (3) Found trouble in wearing the sensor watch. (4) Feel uncomfortable with the gadget near bathroom."					In-home sensor is very helpful for senior citizens living alone. In case anything happens to me, I know someone will help me, feel very secure and safe.Many participants found it reassuring that the system was able to detect deviations in their daily activity patterns and liked it that someone was aware if they declined physically or psychologically.		N
Perspectives on the prospective development of stroke-specific lower extremity wearable monitoring technology: A qualitative focus group study with physical therapists and individuals with stroke	2020	Journal of NeuroEngineering and Rehabilitation	Louie, D.R. and Bird, M.-L. and Menon, C. and Eng, J.J.	10.1186/s12984-020-00666-6	The objective of this study was to identify important considerations in the development of stroke-specific lower extremity wearable monitoring technology for rehabilitation, from the perspective of physical therapists and individuals with stroke.	Qualitative	Canada	stoke patients	Focus group 5: Individuals with stroke (n=3)<br>Sex, male/female<br>2/1<br>Age, median (range), years<br>55 (47–62)	stroke-specific lower extremity wearable monitoring technology			Despite these barriers, the overall sentiment across the focus groups was one of cautious acceptance		In addition to the specific measurements that therapists desired, the analysis revealed several key design features relating to wearability, usability, and functioning that participants considered necessary for stroke-specific wearable monitoring technology. Therapists and individuals with stroke alike agreed that the prospective device should be small, inconspicuous, and lightweight. It should be easily applied and operated with the use of one hand, and as one of the individuals with stroke [illegible]	Participants with stroke emphasized specific muscle activation and timing of activation as their primary measures of interest above and beyond step counts and movement speed. Participants in each focus group listed many considerations and features that could be incorporated into a prospective wearable monitoring device. A multitude of desired measurements was suggested, from joint range of motion and muscle activation, to temporospatial features of gait and symmetry of weightbearing. Physical therapists envisioned an ideal stroke-specific wearable monitor that could do anything from providing a kinematic [illegible]		[illegible] design flaws with current and prospective technology that limit their relevance or utility in therapy.A common concern identified by participants with stroke and physical therapists was the potential cost of future wearable monitoring technology, as well as who should pay for the device. The conversation around cost was intertwined with the discussion around how the prospective technology would be used. If used purely as an in-clinic assessment device, physical therapists would purchase the technology depending on its price.Physical therapists also alluded to administrative and infrastructural reasons that limit adoption of current and new technology into stroke rehabilitation. One such administrative barrier is infection control policy, captured in the following exchange between two hospital physical therapists:Other infrastructural practice barriers that influence physical therapists' decision to utilize technology include limited resources, including both [illegible]			Concerns over accuracy and calibration were also raised, that device reliability or an involved calibration process could deter therapists. Pain, discomfort, and skin sensitivity were also brought up by therapists, as well as participants with stroke, as obvious reasons to avoid using wearable technology.An additional administrative concern is data safety, especially around devices with Internet connectivity. One of their colleagues (P6) expressed a similar concern about developing a reliance on technology if over-utilized	N
Facilitators and barriers to a contingency management alcohol intervention involving a transdermal alcohol sensor	2020	Heliyon	Villalba, K. and Cook, C. and D閔ieux, J.G. and Ibanez, G.E. and Oghogho, E. and Neira, C. and Cook, R.L.	10.1016/j.heliyon.2020.e03612	In preparation for a large, contingency management study for HIV-positive and HIV-negative persons with heavy drinking, the aims for the study were to (1) explore barriers and facilitators to participating in a contingency management intervention using the SCRAM ankle monitor as the potential alcohol measure for the intervention; (2) explore levels of appropriate compensation for using the SCRAM and for study assessments as part of a contingency management intervention study; and (3) attitudes and beliefs on lifestyle changes as a consequence of wearing the SCRAM among HIV-positive and HIV-negative heavy drinkers in Florida.	Qualitative	USA	drinking people	Location(n = 37)<br>Miami, FL 18participants<br>Gainesville, FL 19participants<br>Characteristics<br>Sex, No (%)<br>Men 18 (49%)<br>Women 19 (51%)<br>Race, No (%)<br>African American 16 (43%)<br>White 18 (49%)<br>Multiracial 3 (8%)<br>Hispanic, No (%)<br>Yes 11 (30%)<br>HIV status (%)<br>HIV-positive 15 (40%)<br>HIV-negative 3 (8%)<br>Unknown 19 (52%)	a transdermal alcohol sensor			Attitudes towards wearing the SCRAM sensor around family, friends, or coworkers were mixed, with some participants expressing feelings of embarrassment and fear, especially those who had problems with the justice system.			Participants who were contemplating changing their alcohol drinking behavior were more inclined to wear the SCRAM sensor and participate in the contingency intervention.					Several participants indicated more significant concerns with police encounters if wearing the SCRAM sensor; they were afraid of police harassment or misconduct.	N
Patient Engagement in Medical Device Design: Refining the Essential Attributes of a Wearable, Pre-Void, Ultrasound Alarm for Nocturnal Enuresis	2020	Pharmaceutical Medicine	Caswell, N. and Kuru, K. and Ansell, D. and Jones, M.J. and Watkinson, B.J. and Leather, P. and Lancaster, A. and Sugden, P. and Briggs, E. and Davies, C. and Oh, C. and Bennett, K. and DeGoede, C.	10.1007/s40290-019-00324-w	To date, no pre-void wearable alarm exists to treat nocturnal enuresis (NE) night-time bedwetting, and children with NE and their families are disappointed in relation to the post-void moisture alarms and medicine currently available. Development of a safe, comfortable and non-invasive wearable pre-void alarm and associated technology, using advanced mechatronics, is underway (the MyPAD device).The aim of this study was to help us understand the families' experience of the condition of enuresis, and to provide opinion relating to existing NE alarms, designed to detect moisture, and most importantly, the initial design of the MyPAD wearable technology.	Qualitative	UK	children with NE and their parent(s)	Child<br>Age<br>Gender<br>Parent in attendance<br>1<br>11<br>F<br>Mum 1<br>2<br>7<br>F<br>Mum 2 and Dad 2<br>3<br>8<br>F<br>Mum 3<br>4 (not present)<br>11<br>F<br>Mum 4	a Wearable, Pre-Void, Ultrasound Alarm for Nocturnal Enuresis					The first thing to note is [illegible] they had used previously [illegible] 'air raid siren' and parent [illegible] 'like being in a war zone' [illegible] were hoping to respond [illegible] the families gave a sense [illegible] life. The families wanted [illegible] the parents; weighing up [illegible]<br><br>The fact that some children [illegible] and Child 3.<br><br>Further sleep concerns [illegible] particular concern was [illegible] sounding if the bladder [illegible]<br><br>Although this is a sleep [illegible] appropriate for the family [illegible] urinary tract disease (LUTD) [illegible] postponement, dysfunctional [illegible] suggests, using MyPAD [illegible] may cause unnecessary [illegible]<br><br>Sleep-related concerns [illegible]		Another key theme was a sense of children wanting to 'fit in' [illegible]			However, it was offset throughout by a strong sense of perseverance, resilience and of parents spreading encouragement to others.	Here when the experience is described as 'soul-destroying' one can get a sense of the exhausting challenge of getting up throughout the night to change the bed. This highlights the emotional effect and stress associated for the parent and child. In the last sentence the parent uses 'all' and 'every' which again gives a sense of feeling overwhelmed by the routine of changing clothes and changing sheets.<br><br>For some parents, the pre-void system also addressed another source of frustration by "catching it before it happens" as they felt that a moisture alarm was not addressing the problem.<br><br>Although the parents in this sample were particularly knowledgeable about NE and different treatments, there still appeared to be a lack of information provided about how a post-void alarm should work, i.e., it should awaken the child as they begin to void, and the child should awaken and finish voiding in	For each Author, Noreen Caswell, Kaya Kuru, Darren Ansell, Christian DeGoede, Martin Jones, Benjamin Jon Watkinson, Peter Leather, Andrew Lancaster, Paula Sugden, Eleanor Briggs, Carl Davies, Chooi Oh, and Kina Bennett, no conflict of interest was declared.
Mobile Health Technology for Pediatric Symptom Monitoring: A Feasibility Study	2020	Nursing Research	Vaughn, J. and Gollarahalli, S. and Shaw, R.J. and Docherty, S. and Yang, Q. and Malhotra, C. and Summers-Goeckerman, E. and Shah, N.	10.1097/NNR.0000000000000403	Pediatric blood and marrow transplant (PBMT) patients experience significant symptom distress. This pilot study seeks to (a) explore the feasibility of integrating mHealth technologies to monitor symptom data for PBMT patients and (b) evaluate the study design, measures, and procedures.	Mixed	USA	children	Children (N=10) were between 10–17 years of age (Mean = 14.4 years) and 60% female. 5 black, 4 white, 1 hispanic.	Mobile Health Technology for Pediatric Symptom Monitoring			Of 12 children and their parents approached, 10 enrolled in the study (enrollment rate 83%). Seven (70%) remained in the study for at least 40 days. Two children withdrew from the study within 48 hours and a third withdrew on day 10 (attrition rate 30%). One child withdrew on day 40, the other six stayed in the study from 76–118 days. Acceptability was also evaluated using the structured interview data with 6 children who remained in the study for more than 40 days. All 6 who were interviewed responded (n = 6) that they were satisfied using the mobile devices.		Children (n = 6) reported [illegible] encountered no technical [illegible] days reported that it was [illegible] average, children wore [illegible] recorded data in the app [illegible] indicator of app usability [illegible] shaded regions represent [illegible]			Children (n = 6) reported missing data from the wearable was due to [illegible] the device), feeling too [illegible] prevented wear. For the app, [illegible] to forgetfulness, pain, [illegible]				N
Using Self-Study and Peer-to-Peer Support to Change 揝ick?Care to 揌ealth?Care: The Patient Perspective	2020	Frontiers in Digital Health	Nebeker, C. and Weisberg, B. and Hekler, E. and Kurisu, M.	10.3389/fdgth.2020.00002	Personal health data access creates opportunities to study health indicators 24/7 and in real time. This is especially useful for patients with hard-to-diagnose or treat diseases, which led to a self-formed patient group called Project Apollo. This study understands the experience and expectations of patients affiliated with Project Apollo.	Qualitative	USA	patients	A total of nine (N = 9) individuals participated in an interview, with six responding to a follow-up survey. Participants were all adults over 18 years old and consisted of six females and three males. The estimated age range was 25–75 years of age with all reporting having complete college with the majority having a graduate degree. Participants included eight (N = 8) patients and one (N = 1) clinician associated with Project Apollo.	wearable sensors, affordable lab screenings, genetic and genomic sequencing, and real-time health tracking apps.						In fact, our brief survey revealed that nearly all participants [illegible]	The motivation to revolutionize the healthcare system [illegible]		What has led patients to Project Apollo is their shared frustration with the healthcare system. They receive community support that, along with advances in technology and access to health and research experts, fosters their motivation to study their health conditions through observational self-tracking and N-of-1 studies. A common theme of community support is in empowering their decisions to go forward with self-studies to supplement their healthcare decision-making. In addition to the peer-to-peer community support, they expressed the need for access to health experts and researchers in the process of their self-study research. Ultimately, their shared hope is that through the self-study research combined with advances in technologies, they will facilitate the transformation of the broken sickcare system to a patient-centered, precision health ecosystem.			N
Smart glasses in health care: A patient trust perspective	2020	Proceedings of the Annual Hawaii International Conference on System Sciences	Klinker, K. and Wiesche, M.		Using an online survey with 437 respondents, we find that it is important to properly explain to patients why smart glasses are being used and to proactively address data privacy concerns.	Mixed	Germany	General public	This left us with a total of 437 valid responses to our survey (manipulation: 222 (= 50.8%), control 215 (49.2%)). 69 participants (15.8%) were male and 368 (84.2%) were female. The average age was M = 45.12 years (SD = 13.93). Overall 387 (88.6%) stated that they have received surgery in the past. The manipulation group (n = 222) was comprised of 42 males (18.9%) and 180 females (81.1%). The average age in the manipulation group was M = 45.2 years (SD = 13.87). The control group (n = 215) consisted of 27 males (12.6%) and 188 females (87.4%). The average age was M = 45.0 years (SD = 14.01).	Smart Glasses				Within the manipulation group we checked whether age and gender had an effect on any of the measured constructs. Table 3 lists all constructs that significantly correlated with age in our manipulation group. Age was significantly negatively correlated with the disposition to trust humans as well as the benevolence, integrity and overall trust in the caregiver. All constructs of the manipulation group that significantly differed between male and female participants are listed in Table 4. Overall, females reported higher risk perceptions and privacy concerns, while perceiving smart glasses to be less useful and being less willing to opt-in to medical procedures involving smart glasses than men.						The positive comments on the smart glasses focused on the reduction of errors, higher productivity, and suggestions for other use cases in health care. There were also positive comments which mentioned that smart glasses might be able to prevent others from seeing confidential information.	Some survey participants found that caregivers wearing smart glasses look inhuman. Especially because it is difficult to see the caregiver's eyes. Some comments suggest that survey participants would find it difficult to build a trusting relationship with a person wearing smart glasses. Patients voiced concerns about the misuse of their medical data. They would not like their medical insurance to have access to data that was recorded during their hospital stay.	UNDECLARED
Feasibility and acceptability of wearable sleep electroencephalogram device use in adolescents: Observational study	2020	JMIR mHealth and uHealth	Lunsford-Avery, J.R. and Keller, C. and Kollins, S.H. and Krystal, A.D. and Jackson, L. and Engelhard, M.M.	10.2196/20590	Wearable sleep electroencephalogram (EEG) devices may represent user-friendly methods for assessing sleep among adolescents, but no studies to date have examined the feasibility and acceptability of sleep EEG wearables in this age group. The goal of the research was to investigate the feasibility and acceptability of sleep EEG wearable devices among adolescents aged 11 to 17 years.	Quantitative	USA	Adolescents	A total of 123 adolescents and their guardians expressed interest in study participation and completed an eligibility prescreening via telephone. Of these individuals, 3 were excluded due to use of sleep medications and 13 declined to participate in the study following the phone screen. A total of 107 participants completed the initial in-person visit. One individual attended the in-person visit but declined participation following the consent process. Two additional individuals completed the study in full but are not included in this analysis due to difficulty retrieving the devices attributable to circumstances surrounding the novel coronavirus (ie, North Carolina COVID-19 shelter-in-place order), leaving a final sample of 104.	Wearable Sleep Electroencephalogram Device Use in Adolescents			Adolescents were most likely to rate themselves as satisfied with participation in the study based on their experience with the sleep EEG (53/103, 51.5%) and actigraphy (71/103, 68.9%) devices, followed by ambivalence (neither satisfied nor dissatisfied; 39/103, 37.9%, for the sleep EEG; 31/103, 30.1%, for the actigraph). Very few adolescents rated themselves as dissatisfied with their participation in the study based on experience with the sleep EEG (11/103, 10.7%) or actigraph (1/103, 1.0%; Figure 5)<br><br>The majority of adolescents would recommend the study to a friend based on their experience using the sleep EEG (77/98, 79%) and actigraphy (88/98, 90%; Figure 6).	.Interestingly, there was a statistically significant association between satisfaction and device comfort while sleeping (χ24,n=103=17.21, P=.002); the small number of adolescents who rated themselves as dissatisfied with the study based on sleep EEG use were also more likely to have experienced significant (moderate to severe) device-related discomfort (number observed = 9, number expected = 3.74). Similarly, there was a statistically significant association between satisfaction and significant (moderate to severe) device-related sleep disturbances (χ 24,n=103=21.28, P=.002), such that adolescents who rated themselves as dissatisfied with sleep EEG use were more likely to have experienced significant device-related sleep disturbances (number observed = 9, number expected = 2.14).	Comfort The majority of [illegible] uncomfortable range wh[illegible] (95/103) and sleeping [illegible] Figure 2). When asked [illegible] adolescents most frequently [illegible] caught/tangled or becom[illegible] during sleep) or the adh[illegible] was itchy). A handful of [illegible] changing sleep position [illegible] that they would mess up [illegible] to discomfort while using [illegible] issues related to the light [illegible] sleep, and the wristband [illegible] bulky). For both devices [illegible] minimal or mild.Wearab[illegible] related interference with [illegible] typical of their normal s[illegible] that the sleep EEG devi[illegible] 39.8%). Similarly, the m[illegible] wristworn actigraph as [illegible] (31/103, 30.1%). The m[illegible] to EEG were difficulties [illegible] (23/104), followed by p[illegible] of the reported disturba[illegible]							Authors do not have any conflicts of interest to report in regard to the devices used in this study. ADK has received research funding from Janssen Pharmaceuticals, Axsome Pharmaceutics, Reveal Biosensors, The Ray and Dagmar Dolby Family Fund, and the National Institutes of Health. He has received consulting fees from Adare, Axsome Therapeutics, Big Data, Eisai, Evecxia, Ferring Pharmaceuticals, Galderma, Harmony Biosciences, Janssen Pharmaceuticals, Jazz Pharmaceuticals, Millennium Pharmaceuticals, Merck, Neurocrine Biosciences, Pernix, Otsuka Pharmaceuticals, Sage, and Takeda. Authors JRLA, CEK, SHK, LJ, and MME do not have any conflicts of interest to report.
Feasibility of using mobile technology to improve physical activity among people living with diabetes in Asia	2020	Asian Pacific Island Nursing Journal	Lukkahatai, N. and Soivong, P. and Li, D. and Jaiman, P. and Thamkaew, S. and Chaiwong, D. and Hiranlalit, N. and Inouye, J.	10.31372/20200504.1110	This study aims to examine the feasibility and the impact of the immediate performance feedback feature of the wearable device on physical activity (PA).	Quantitative	Thailand.	Diabetes patients	participants diagnosed with T2DM, ages 39–70 years old (mean ± SD = 56.5 ± 7.2) were included in this analysis. The majority were female (n = 86, 69%) and married (n = 103, 82%). Approximately 90% had primary school education. More than 68% were overweight and obese (BMI < 22.9). Their fasting blood glucose (FBS) ranged from 70 to 329 (mean ± SD = 143.2 ± 45.3). The hemoglobin A1C ranged from 5.3% to 11.5% with a mean of 8.3% (SD = 1.8) (	Mobile Technology to Improve Physical Activity Among People Living with Diabetes in Asia			Acceptability and Satisfaction. The majority of participants in both groups reported that they liked using the device (79% of the AW group and 71% of the UW group). More than 55% of participants in the AW group liked the device because of the ability to see the step count; the majority of the UW group (37%) did not answer this question. Nearly 70% of participants in the aware group felt that this device helped increase their exercise; 82% of participants in the unaware group did not think that the device increased their exercise (Table 2).		No adverse events (discomfort, allergic reactions, or device malfunctions) were reported for both groups.							N

Wearable activity-tracking device feature preference amongst south african generation y students	2020	International Journal of eBusiness and eGovernment Studies	Muller, C.	10.34111/ijebeg.202012101	As such, this study explored South African Generation Y students' feature preferences on wearable activity-tracking devices in order to assist device manufacturers and marketing practitioners in developing and marketing devices that will appeal to this large segment.	Quantitative	USA	Generation Y students	Of the 600 questionnaire distributed, 543 were returned. Of the returned questionnaires, 480 fell within the defined target population. As such, this study had an 80 percent actual response rate. Table 1 indicates that there were slightly more females (59.4%) than males (40.2%) and that the majority of the sample fell within the Black/African (89%) ethnicity group. Smaller representations came from other ethnic groups, those are 3.3 percent Coloured individuals, of which 2.1 percent were either Indian or Asian individuals, and 5.2 percent of the sample fell within the White ethnic group. The sample represented all of the age groups, 7 where the majority of the sample participants were aged between 18 and 21 years. Of the nine South African provinces, only one province (Northern Cape) had no representatives, with the highest representation being from the Gauteng province. There were slightly	WEARABLE ACTIVITY-TRACKING DEVICE	Therefore, it is evident that the awareness and adoption of wearable activity-tracking devices is still in the early phase amongst members of the South African Generation Y cohort.		However, despite this low ownership of activity-tracking devices, it is reassuring that 76.5 percent of the sample participants had shown an interest in tracking their daily activity.			it can be deduced that they are more likely to use more advanced technology, such as wearable activity trackers, to track their preferred metrics in future.	It is evident from Table 3 that a wearable activity-tracking device's function for measuring heart rate and blood pressure was the main priority according to the majority, 64.8 percent, of the sample participants. Tracking steps and distance travelled (46.7%), calculating daily calories burnt (43.1%), tracking sleep patterns (38.5%), GPS tracking (38.5%), and being waterproof or water resistant (33.5%) all received favour by more than 30 percent of sample participants. Wearable activity-tracking devices' ability to measure perspiration (13.3%), alerting the user of prolonged inactivity (7.5%), as well as being customisable by means of interchangeable bands (3.3%) were the three least favoured features among the sample participants. This indicates to device manufacturers where the focus should be regarding device functionality. In order to make this data more useful to the industry, which could lead to a more practical implementation for device manufacturers to develop specific devices, retailers, e-commerce sites, and their relevant marketing departments to advertise and promote the preferred features, this study focused on the five most important features of a wearable activity-tracking devices based on the sample participants' responses. The results derived from Table 3 indicate that the top five preferred features amongst the sample participants include measuring heart rate and blood pressure (311 responses), followed by tracking steps and distance travelled (224 responses), calculating calories burnt (207 responses), and tracking sleep patterns and GSP tracking which received the same amount of					UNDECLARED
Feasibility of a mobile health tool for mothers to identify neonatal illness in rural Uganda: Acceptability study	2020	JMIR mHealth and uHealth	Matin, S.B. and Wallingford, A. and Xu, S. and Ng, N. and Ho, A. and Vanosdoll, M. and Waiswa, P. and Labrique, A.B. and Acharya, S.	10.2196/16426	The aim of this study was to determine if mothers in rural Uganda were willing and able to use the NeMo system during the first week of their infant's life. We also assessed mothers' responses to the device's recommendation to seek care.	Quantitative	Uganda	mothers, neonates	Characteristic Values<br>Age (years), mean (SD) 27.2 (6.84)<br>Distance from hospital (km), mean (range) 7.78 (0.40-24)<br>Parity, mean (range) 3.5 (1-7)<br>First time mothers, n (%) 5 (25)<br>Own a phone, n (%) 11 (55)<br>Have ever used a smartphone, n (%) 8 (40)<br>Able to charge a phone, n (%) 18 (90)<br>Attended ANCa, n (%) 19 (95)<br>ANC sessions attended, mean (range) 4 (0-5)<br>Knew danger sign, n (%)<br>Failure to breastfeed 8 (40)<br>Convulsions 5 (25)<br>Breathing difficulty 2 (10)<br>Lethargy 2 (10)	a Mobile Health Tool for Mothers to Identify Neonatal Illness in Rural Uganda			Willingness to Use<br>Using a composite scoring system (strongly agree=5 and strongly disagree=1), the statement "I have used the device regularly" had a composite score of 4.72 (median 5, IQR 1). The statement "I would trust this device to assess the health of my baby" had a composite score of 4.5 (median 4.5, IQR 1). All 18 mothers who completed the study answered that they would like to use the device again if they had another baby, and 94% (17/18) of mothers said that they would recommend using the device to other women in their village. During the exit survey, 5 mothers stated that they thought the device should be used more than three times a day and suggested instead that recommended daily usage be increased to four or five times.		Ease of Use<br>All mothers agree or strongly agree that the device was easy to use, giving a composite score of 4.89 (median 5, IQR 0). All 18 mothers answered that they had become more comfortable using the device over the course of the study.<br><br>Device Maintenance<br>Out of the 20 enrolled mothers, 11 (55%) were able to charge phones at home, whereas the rest needed to charge at a neighbor's house or nearby charging station up to 1.5 km from their home. During recruitment, 10% (2/20) of the mothers answered that they would have major difficulties (ie, have to travel a long distance or pay) and were, thus, provided with power banks to facilitate charging. Out of the 18 participants who completed the study, 11 (61%) charged the phone at least once during the study period. There were no adverse system events (including device breakage or loss of either phone or device) during the study despite 55% (10/18) of participants expressing concerns about losing or breaking the device. All devices remained intact and functional by day 6 when the devices were collected.					Perception of Usefulness<br>All mothers agree or strongly agree that the device helped them know when to seek care for their babies, resulting in a composite score of 4.66 (median 5, IQR 0). The most common reasons given for agreeing with this statement were that the device can assess breathing rate, which the mother cannot do on her own (4 mothers), and the device displays the danger signs (3 mothers). In total, 89% (16/18) of the participants who completed the exit survey said that other mothers would find the device useful.	Common themes include concerns about the electrical safety of the device, potential stress to the baby caused by the band, and unwillingness to unwrap the baby to put the device on when the weather is cold. All of the reported concerns or questions were answered by the team when they were raised.	N
Patient and carer survey of remote vital sign telemonitoring for self-management of long-term conditions	2019	BMJ Health and Care Informatics	Walkden, J.-A. and McCullagh, P.J. and Kernohan, W.G.	10.1136/bmjhci-2019-100079	We set out to verify stakeholders' perspectives of remote vital sign telemonitoring.	Mixed	USA	Patient and carer	83.5% of whom used the regionally commissioned TF3 service. There was an approximate gender balance of patients (49 males and 39 females, 9 did not respond). The mean age of patients was 68.8 years (69.5 years for TF3 and 64 years for U-Tell);	remote vital sign telemonitoring for self-management of long-term conditions			Analysis showed that respondents were supportive of the technology with 90.7% of patients agreeing or strongly agreeing with the statement	These views were independent of the technology used and not associated with the patient's long-term conditions, gender or age. There were no reported adverse incidents.						: the remote monitoring system assisted me in managing my health on a day-to-day basis. The patients liked the technology largely because it provided empowerment and control for self-management and allowed them to continue with their lives without major disruption.		N
Patients' experience of wearing multimodal sensor devices intended to detect epileptic seizures: A qualitative analysis	2020	Epilepsy and Behavior	Simblett, S.K. and Biondi, A. and Bruno, E. and Ballard, D. and Stoneman, A. and Lees, S. and Richardson, M.P. and Wykes, T.	10.1016/j.yebeh.2019.106717	This study assessed the first-hand experiences of people with epilepsy using wearable devices, continuously over a period of time. The aim was to understand how acceptable and easy they were to use, and whether it is reasonable to expect that people will use them.	Qualitative	UK	Patients with epilepsy	Demographic & clinical Empty Cell<br>Gender Female: N (%) 7 (33.3)<br>Age (years): mean ± SD 40.4 ± 13.3<br>Epilepsy Duration (years): mean ± SD 21.8 ± 13.5<br>Self-reported seizure frequency (seizure/month): mean ± SD 22.3 ± 38.0<br>Number of AEDs: mean ± SD 2.7 ± 1.1<br>Period of data collection prior to interview (days) mean ± SD 3.09 ± 1.59<br>Participants reporting previous experience with a wearable device N (%) 7 (33.3)<br>Duration of prior experience with wearable device (months): mean ± SD 7.3 (4.5)<br>Educational level completed: N (%)<br>University graduate<br>A level/ B-tech/ Apprenticeship (high school exams taken age 18)<br>GCSE (high school exams taken age 16)<br>Did not achieve GCSE<br>6 (28.5)<br>9 (42.8)	multimodal sensor devices intended to detect epileptic seizures			Twenty-one people with epilepsy wore one, or more, wearable devices for an average of 112.81 (SD = 71.83) hours.	Willingness to use a smartphone app to complete questionnaires depended on the frequency, number of questions, and support.	Participants found the devices convenient, and had no problem wearing them in hospital or sharing the data collected from them with the researchers and medical professionals. However, the presence of wires, bulky size, discomfort, and need for support, moderated experience. Participants' thoughts about wearing them in everyday life were strongly influenced by how visible and perceived accuracy.							N
Patients?views of wearable devices and AI in healthcare: findings from the ComPaRe e-cohort	2019	npj Digital Medicine	Tran, V.-T. and Riveros, C. and Ravaud, P.	10.1038/s41746-019-0132-y	Wearable biometric monitoring devices (BMDs) and artificial intelligence (AI) enable the remote measurement and analysis of patient data in real time. Our objective was to describe patients' perceptions of the use of BMDs and AI in healthcare.	Quantitative	France	Patients	conditions [861 (73%) female] participated in the study between May and June 2018 (participation rate: 48%) (Table 1). The mean age was 49.7 years (SD = 14.5). Patients' conditions included diabetes (n = 121), asthma (n = 77), rheumatologic conditions (n = 367), neurological disorders (n = 234) and cancer (n = 107). A total of 649 (54%) participants had multimorbidity (mean number of conditions 2.5 [SD = 2.4]). In total, 590 (50%) participants reported using e-health or m-health tools for health [smartphone apps for 246 (21%), wellness wearable devices for 61 (5%), medically prescribed wearable device (e.g., continuous glucose monitoring tool) for 50 (4%), and health internet	wearable devices and AI in healthcare			Approximately 20% of patients with chronic conditions were opposed to the use of BMDs and AI-based tools in their care in all presented situations. This proportion ranged from 17% for the use of patients' skin photographs and AI to screen for skin cancer to 21% for the use of a smart shirt and AI to guide physical therapy. Accordingly, about 80% of participants were ready for the use of technology in their care. However, only a fraction of these patients were ready for the use of AI without human control (from 10% for the use of patients' skin photographs and AI to screen for skin cancer to 36% for the use of AI chatbots to assist in determining how urgent their problems were).	We found no clear association between patients' demographic or clinical characteristics and their readiness to use BMDs and AI-based tools in their care (Supplementary Table 4).						we found that 47% of participants considered BMDs and AI as a great opportunity (rating > 7/10) (IQR of opportunity ratings: [5-9]) (Supplementary Table 1). With the open-ended questions, patients identified 47 potential benefits of the use of technology in healthcare. They believed that it could improve their follow-up and the reactivity of care (55%), reduce their burden of treatment (23%) or facilitate physicians' work (eg, by automating repetitive tasks) (21%) (Fig. 1, Table 2, Supplementary Table 2).	BMDs and AI as a great danger (rating >7/10) (IQR of danger ratings: [2–6]). With the open-ended questions, patients identified 31 potential risks for the use of technology in healthcare. They feared that it could inadequately replace human intelligence in care (28%), represent serious risks for hacking (13%), or lead to misuse of private patient data by caregivers, insurance companies, etc. (14%) (Fig. 1, Table 3, Supplementary Table 3). Overall, 20% participants considered that the potential benefits of technology greatly outweighed its potential dangers (opportunity > 7/danger < 3), whereas only 3% felt that negative aspects outweighed potential benefits (opportunity < 3/danger > 7) (Supplementary Table 1).	N
Determinants of users?intention to use smartwatch	2019	Journal of Theoretical and Applied Information Technology	Baba, N.M. and Baharudin, A.S. and Alomari, A.S.		Thus, this study aims to investigate the level of Malaysian individuals' intention to use a smartwatch and to identify and assess their impacts.	Quantitative	Malaysia	General public	respondents were less than 30 years old, thus this shows that the majority of the respondents are in their young age. The gender analysis revealed that 70% of the respondents were female while the remaining were male. Also, for the ethnicity, more than half of the populace are Malay, followed by Chinese with 23% while only 44 respondents are Indians with 7.9% representation. This finding revealed almost the true representative of the ethnicity of the Malaysian citizens.	SMARTWATCH			This results implies that students in Malaysia perceived smartwatch as a task-oriented smart device used for utilitarian usefulness that they can use to increase work productivity and quickly accomplish certain tasks.	Based on the results, the studied verified that perceived usefulness has positive impact on users' intention to use smartwatch. This implies that higher PU will increase users' intention to use smartwatch. Also, PEOU showed a positive impact on intention to use smartwatch and this implies PEOU is an essential factor of users' intention to use smartwatch.				Results from the unverified relationship of the study showed that cost, privacy, and health risk did not negatively affect users intention to use smartwatch. This implies that higher cost would not hinder users' intention to use smartwatch and that potential users considered the cost of adopting the smartwatch devices as negligible when they compare the anticipated benefits arising from using the devices. Furthermore, result for the privacy entails that the increase of privacy of smartwatch devices will not lead to decrease in the intention to use smartwatch. Equally, the health risk findings implies that users' perceived health risk perception would not deter their intention to use smartwatch devices.				UNDECLARED
A 12-week pilot study of acceptance of a computer-based chronic disease self-monitoring system among patients with type 2 diabetes mellitus and/or hypertension	2019	Health Informatics Journal	Yan, M. and Or, C.	10.1177/1460458217724580	This study tested a structural model examining the effects of perceived usefulness, perceived ease of use, attitude, subjective norm, perceived behavioral control, health consciousness, and application-specific self-efficacy on the acceptance (i.e. behavioral intention and actual usage) of a computer-based chronic disease self-monitoring system among patients with type 2 diabetes mellitus and/or hypertension.	Quantitative	Hong Kong, China	diabetes patients	Characteristics<br>Age, mean ± SD 69.9 ± 9.7 years<br>Gender, n (%)<br>Male 46 (38.7)<br>Female 73 (61.3)<br>Living status, n (%)<br>Live alone 36 (30.3)<br>Live with family 83 (69.7)<br>Type of disease, n (%)<br>Type 2 diabetes mellitus 1 (0.8)<br>Hypertension 74 (62.2)<br>Comorbid type 2 diabetes mellitus and hypertension 44 (37.0)<br>Duration of disease since diagnosis, mean ± SD<br>Type 2 diabetes mellitus 10.0 ± 7.2 years<br>Hypertension 9.5 ± 7.8 years<br>Education, n (%)<br>Primary school or lower 69 (58.0)<br>Secondary school 39 (32.8)<br>College or higher 11 (9.2)<br>Computer experience, n (%)<br>Absence 72 (60.5)	a computer-based chronic disease self-monitoring system among patients with type 2 diabetes mellitus and/or hypertension					Patients' average ratings for application-specific self-efficacy, attitude, health consciousness, perceived behavioral control, perceived ease of use, perceived usefulness, subjective norm, and behavioral intention ranged from 5.38 to 5.69; the lower bounds of the ratings were above five at the 95 percent confidence level. Except for perceived ease of use, the absolute values of skewness and kurtosis were less than 1, indicating normal distributions of the data.							N
"you have to know why you're doing this": A mixed methods study of the benefits and burdens of self-tracking in Parkinson's disease	2019	BMC Medical Informatics and Decision Making	Riggare, S. and Scott Duncan, T. and Hvitfeldt, H. and H縊glund, M.	10.1186/s12911-019-0896-7	This study explores opinions and experiences of people with Parkinson's disease (PwP) in Sweden of using self-tracking.	Mixed	Sweden	people with Parkinson's disease	Age<br>26–35 yrs<br>36–45 yrs<br>46–55 yrs<br>56–65 yrs<br>66–75 yrs<br>76–85 yrs<br>> 86 yrs<br>Women<br>1<br>6<br>14<br>28<br>30<br>9<br>Men<br>11<br>23<br>46<br>11<br>1	self-tracking in Parkinson's disease	51% said that they have made observations through their tracking that has enabled them to understand their PD better and 67%, said that they have made changes to their PD management as a result of tracking, see Fig. 7. For PwP under 65, this rate was significantly higher (69 and 73% respectively) compared to those older than 65 (39 and 61% respectively) ($\chi2(2)$ =15.841, p = .0004; $\chi2(2)$ =5.998, p = .05), see Fig. 4. One in two, 53%, were frustrated with how difficult it is to track and 36% think it is difficult to know how to make use of the results of tracking			Mode of tracking seems to be unrelated to gender, age, and education level. When it comes to time since diagnosis however, PwP diagnosed more than five years ago were significantly more likely to track using pen and paper (66%) than those diagnosed five years ago or less (41%) ($\chi2(1)$ =10.533, p = .001), see Table 3.The most common aspects to track in the survey were medication intake times (67%), medication types (62%), and physical activity/exercise (61%),		All respondents in the interviews talked about reasons for self-tracking. Several of them mentioned that they have a mind-set for self-tracking and therefore may be more interested than PwP in general. They also demonstrated an awareness of the highly individual presentation of PD, that the illness will be very different for different individuals and that each person needs to understand their own symptoms and how to manage them. Some respondents also described self-tracking as a means to achieve increased awareness of their illness and its progression.Similarly, in the survey (see Fig. 3), the most common response to why people self-track, was that they expect it to enable them to understand their PD better (74%).PwP younger than 65 are significantly more likely to state that self-tracking enables them to understand their PD better (87%) than PwP older than 65 (63%) ($\chi2(2)$ =13.215, p = .001), see Fig. 4.	Our interview respondents described knowing what and how to track as important. The primary parameter to track among the participants was medication intakes, with the purpose to optimise timings. Other parameters important to measure were stress, diet and sleep. Different influencers were also acknowledged as a challenge in the interpretation of the collected data, and several participants described how important it was to understand these complexities in order to benefit from tracking.<br><br>The respondent with the most experience of tracking also expressed more abstract reasoning around how to capture the right measure when tracking, and what effects the choice of measurement can have. The same respondent also reflected on the challenges of defining more subjective measures to find a measure that is both efficient and reliable. What does it mean to me to "feel well"?	In Fig. 3 is also presented that about one in two of our survey respondents (53%) state that they self-track because they enjoy it. However, they also think it is challenging to track; 58% find it difficult to know what to track and 61% find it difficult to know how to track. No more than 22% say that they try to track everything and 18% say they try to track all the time. In contrast, 61% say they track sometimes and that they track specific aspects of their PD. PwP who have been diagnosed for more than five years are significantly more likely to try to track all the time (21%) than those who were diagnosed less than five years ago (13%) ($\chi2(2)$ =6.676, p = .04), see Fig. 5. In total 36% of PwP share their experiences of tracking with others, see Fig. 3. Those who have been diagnosed for more than five years are significantly more likely to share with others (46%) compared to those diagnosed less than five years ago (23%) ($\chi2(2)$ =10.084, p = .006), see Fig. 5.		The interview respondents also mentioned self-tracking as a tool to find and understand correlations between health status or symptoms and medication intakes. It is a way to recall how they were doing over time, for example to remember how symptoms fluctuate. Both of these reasons were chosen by 73% of the respondents of the survey, see Fig. 3.<br><br>Some of the interview respondents talked about how they take an active approach in the management of their PD. Self-tracking was described as a way to stay in control, and to take personal responsibility for one's health using an active approach.<br>Several of the interview respondents mentioned that they had to learn a lot themselves to develop the understanding for the causalities and inferences necessary to really benefit from tracking.<br><br>Some of our interview respondents used tracking results as a trigger to contact healthcare to handle worsening symptoms.	Potential risks related to tracking are the fixation of tracking and not to let it take over your life. Most frequently mentioned by our interview respondents was the risk of PwP getting fixated or obsessed with tracking.<br><br>On the issue of data privacy, our interview respondents in general expressed a trust in the healthcare system and did not see any major risks when it comes to sharing their data, neither with healthcare nor with other patients or caregivers. Sharing was considered positive as long as it was on their terms and with appropriate security measures in place.	N
Pregnancy monitoring mobile application user experience assessment	2019	2019 International Conference on Information and Communications Technology, ICOIACT 2019	Wicahyono, G. and Setyanto, A. and Raharjo, S. and Munandar, A.	10.1109/ICOIACT46704.2019.8938446	This research, therefore, proposes a mobile application to automatically record pregnant women mobility and the facility of image processing to read the weight of the scale. This paper aims to discuss the user experience in the implementation of the mobile smart birth monitoring application.	Quantitative	Indonesia	pregnant women		Pregnancy Monitoring Mobile Application					the application. The second lowest is the efficiency at 1.100, the score is just slightly above the lowest border of above average. Therefore, efficiency is also need attention to be improved. More detail result is presented in table 2.<br><br>With regards to the novelty score, there are 4 item represents novelty which are number 3 (creative – dull), number 10 (inventive - conventional), number 15 (usual – leading edge) and 26 (conservative – innovative). Among four items, question about usual leading edge recorded score 0, which is bad. Considering that the user has been intensively use this application for three months, this score is far for satisfying. Let consider figure 3 and 4, the user interface is consider as usual design, therefore a massive change in the design needs to be performed.<br><br>Efficiency come second least score and just in the border between below average and above average category. There are four items on this dimension which are number 9, 20, 22 and 23. Among four question 23, (cluttered – organize). The score of this question at 0.7, therefore an improvement of tidy design need to be done. User also still feel that the application is slow according to question number 9. Generally speaking the user experience shows many improvement needed for the application success in the future.							UNDECLARED
Breast cancer survivors?perspectives on a home-based physical activity intervention utilizing wearable technology	2019	Supportive Care in Cancer	Kokts-Porietis, R.L. and Stone, C.R. and Friedenreich, C.M. and Froese, A. and McDonough, M. and McNeil, J.	10.1007/s00520-018-4581-7	To gain breast cancer survivors' perspectives on participation in a home-based physical activity intervention and the factors that contributed to their acceptance and adherence to physical activity.	Qualitative	Canada	Breast cancer survivors	Age (years); mean ± SD<br>58 ± 7<br>58 ± 10<br>58 ± 9 Marital status<br>Married or common law; n (%)<br>4 (66.7)<br>17 (77.3)<br>35 (77.8)<br>Other; n (%)<br>2 (33.3)<br>5 (22.7)<br>10 (22.2)<br><br>Education<br>≤High school; n (%)<br>1 (16.7)<br>5 (22.7)<br>8 (17.8)<br>≥Post-secondary school; n (%)<br>5 (83.3)<br>17 (77.3)<br>37 (82.2)<br>Ethnicity<br>Caucasian; n (%)	Breast cancer survivors' perspectives on a home-based physical activity intervention utilizing wearable technology								Feeling busy was the most impactful barrier followed by lack of motivation and weather (Fig. 1). Lack of interest, lack of knowledge, and having no one to exercise with were consistently reported as having "no impact" for all time points (data not shown).		Wearable technology was identified as a tool to create self-awareness and reinforce physical activity. The activity tracker provided information not otherwise accessible to participants.	Wearable technology was also perceived as a source of judgment and failure by participants. These sentiments often occurred when the device did not accurately reflect its user's physical activity<br><br>Some participants found that wearable technology created a "distorted view" of how active they were,	N
Wearable technology to support early child language experiences: What's important to parents and clinicians?	2019	Studies in Health Technology and Informatics	Choo, D. and Dettman, S. and Dowell, R. and Cowan, R.	10.3233/SHTI190772	The present study explored salient requirements of a wearable language tracking device from the users' perspectives.	Quantitative	Australia	mothers of children with significant hearing loss who were enrolled in early intervention programs and four mothers of children with normal hearing	Of the 23 participants, two mothers (22.2%) were between 25 and 34 years of age and seven mothers (77.8%) were aged between 35 to 44 years. There were five mothers of children with significant hearing loss who were enrolled in early intervention programs and four mothers of children with normal hearing. The majority (n=8) resided in the state of Victoria except one mother who lived in New South Wales	Wearable Technology to Support Early Child Language Experiences					participants (78.3%) assigning it a rating of 10 (Mean=9.6; Median=10; IQR=0). A high proportion of participants rated reliability (69.6%) and the ability for the device to provide clear and useful results (69.6%) as important features. There were 9 of 16 items on the questionnaire which were assigned the highest importance value of 10 by at least half (50%) to 78.3% of the participants in the sample. The value placed on these 9-items indicated that it was substantially important for a body worn device to be: comfortable; compact (light and small); reliable; easy to attach to the body/clothing and to operate (and maintain); and to: have clear and readable instructions; have motivating features for users; and not affect normal daily behavior; and to provide clear and useful results.							N

Experiences of adults with type 1 diabetes using glucose sensor-based mobile technology for glycemic variability: Qualitative study	2019	JMIR Diabetes	Ritholz, M.D. and Henn, O. and Castillo, A.A. and Wolpert, H. and Edwards, S. and Fisher, L. and Toschi, E.	10.2196/14032	Adults with type 1 diabetes (PWDs) face challenging self-management regimens including monitoring their glucose values multiple times a day to assist with achieving glycemic targets and reduce the risk of long-term diabetes complications. The aim of this qualitative study was to explore PWDs' experiences using Sugar Sleuth, a glucose sensor-based mobile app and Web-based reporting system, integrated with the FreeStyle Libre glucose monitor that provides feedback about glycemic variability.	Qualitative	USA	Diabetes patients	Demographic characteristics Semistructured interviewed (n=10) Not interviewed (n=20) P value<br>Gender, n (%)<br>.32<br>Male 6 (60) 12 (60)<br>Female 4 (40) 8 (40)<br>Age (years), mean (SD) 52 (15) 56 (14)<br>.53<br>Ethnicity, n (%)<br>.02<br>Non-Hispanic 9 (90) 16 (80a)<br>Hispanic 1 (10) 1 (5a)	Glucose Sensor-Based Mobile Technology						Approaches to Glycemic Data: Overview Versus Narrow View<br>A total of 4 participants described utilizing an overview approach to their glycemic data, which included actively reviewing graphs and app information to better understand cause and effect regarding their glycemia and to plan for future self-care:<br>On the contrary, 6 participants described having a narrow view or focusing only on the immediate data and were reluctant to use the app or Web-based information to review data. However, they were still engaged with the app, that is, the app still prompted them to enter in the suspected causes of glucose excursions within hours of their occurrence. This narrow view suggests that the immediate information they received was sufficient, and they did not want or need to focus on past information and generally wanted to move on:		Interestingly, this second participant was the only Hispanic person in the study. He voiced concern that it might be too difficult for PWDs from a lower socioeconomic or a different cultural background to adopt this technology because they do not have access to the same information or resources as those from the majority culture. He pointedly illustrated his concern when reporting how much it bothered him that he could not understand some of the choices offered by the app:		[illegible]<br>Participants described Sugar Sleuth as providing an empowering educational tool that informed their lifestyle choices and promoted their engagement in diabetes self-care tasks. Sugar Sleuth also fostered preemptive self-care actions as well as improved discussions with their clinicians. Furthermore, participants reported that Sugar Sleuth provided constant and immediate feedback that increased their understanding of the factors contributing to their glycemic excursions (variability) and how and when to address these excursions:<br>Source of Psychosocial Support<br>Participants described Sugar Sleuth as offering relief from worry and as helping them cope more effectively with T1D. They perceived the constant information that Sugar Sleuth provided as making for less glycemic uncertainty; they and others worried less about whether or not they were hypo- or hyperglycemic. These feelings contributed to participants feeling more positive about their	Only 2 participants reported negative perceptions about their use of the Sugar Sleuth system. They perceived the app as too demanding of information or as not attuned to the socioeconomic backgrounds of diverse populations. For example, one participant stated that the app's demand for information did not always fit with where he was in his glycemia. He noted how difficult it was to answer questions about the causes of his hypoglycemia while he was in the midst of a hypoglycemia episode:	MDR, ET, and HW work at the Joslin Diabetes Center. The Joslin Diabetes Center has received research funding from Abbott Diabetes Care. LF has received compensation as a consultant from Abbott Diabetes Care.
Why do older adults use wearable devices: A case study adopting the senior technology acceptance model (STAM)	2019	PICMET 2019 - Portland International Conference on Management of Engineering and Technology: Technology Management in the World of Intelligent Systems, Proceedings	Yu-Huei, C. and Ja-Shen, C. and Ming-Chao, W.	10.23919/PICMET.2019.8893767	This study applied the Senior Technology Acceptance Model (STAM) and investigated the factors influencing the acceptance of new technology by focusing on smart watch and bracelet adoption among the elderly.	Qualitative	Taiwan	Older Adults	The older generation consisted of 11 males and 4 females aged 50-79 years. The younger generation consisted of 5 males and 10 females aged 21-56 years. In general, the average age difference between the older and younger generation was about 27 years old in average.	Older Adults Use Wearable Devices	Both the older and younger generations have experience with at least one technology product (e.g., mobile phone). There is only one older interviewee who purchased the wearable device by himself, and 14 people were gifted them from family members or peers. However, 8 of the younger participants bought the devices on their own, and 6 obtained them from family members and peers, while one participant gain it from lucky draw.		were willing to do so.Regarding the usefulness of the wearable devices, 13 older adults and 14 younger participants responded positively while 2 and 1 responded negatively, respectively.		According to Table 3, the main functions that were used by the old and young generations were healthcare and exercise measurements. Thus, differences between other items were not significant among the two generations.On the perception of learning, 10 out of 15 in the older group felt that the wearable devices were easy to learn, while 2 complained that they were difficult, and 3 were neutral..<br>For the younger generation, 12 people thought they were easy to use, while 1 found them difficult to use, and 2 were neutral.<br>From the older generation 2 people thought it was too complicated to use. 3 people commented that it was easy to forget how to use after learning.<br>For the Young Generation, 1 person thought it was easy to forget after learning. 1 person commented it was complicated. 1 person noted that the text of the manual was too small to read.<br>• Perceived ease of use<br>For the older generation, 11 out of the 15 agreed that the wearable device was easy to use. 2 claimed that the device was neither easy nor difficult to use, and 2 participants said that it was difficult to use. Of the younger generation, 12 out of the 15 felt that it was easy to use, 2 felt that the device was neither easy nor difficult to use, and 1 stated that it was difficult to use.<br>In addition, the negative factors were that it was uncomfortable and lead to itching		However, both generations accepted the products in principle but were unsatisfied by their performance and hoped they could be improved. In general, they were satisfied with the healthcare, sleep monitoring, and exercise recording aspects and unsatisfied with the battery life, strip quality, incorrect measurements and because it was too complicated.<br>Also, the product design was not fancy enough, the screen should be larger, and it should auto adjust to changes in brightness. Finally, the voice control functions should be improved.	• Information source<br>This study found that three of the eight did not use the products because they had never heard of them yet were willing to do so. Thus, regarding the elderly consumer and technology acceptance, Gilly and Zeithaml [6] claimed that the manufacturer should contact older people instead of depending on word-of-mouth.<br>There were many reasons given for not using wearable devices, including not feeling it was necessary, being too complicated to operate, not having social influence and not getting the related information.<br>In addition, due to aging, older people are less curious, and they are often not aware of new technology products. However, most of them are willing to consider adopting wearable devices because of their health concerns.<br>• Culture issue<br>Concerning the cultural influence, two interviewees mentioned that if their family members or colleagues used the technology products and said that it was really important and helpful, then they would consider using the products.<br>• Learning issue<br>Learning abilities decline with older age, therefore, this study highlights	The results of this study showed that most of the older generation received information regarding their wearable devices from family members and peers (11 people out of 15 in our interview group), while only one saw it from a website, and the other 3 learned information from advertisements and magazines.<br>For the younger generation, 5 out of 15 learned about them from their family members and peers, 8 via the internet, and 2 from advertisements and magazines. Therefore, the older generation obtained technology information more often from family members and peers, whereas the younger generation were more active in searching for information online or via advertisements and magazines.			UNDECLARED
Self-management maintenance inhalation therapy with eHealth (SELFIE): Observational study on the use of an electronic monitoring device in respiratory patient care and research	2019	Journal of Medical Internet Research	Kuipers, E. and Poot, C.C. and Wensing, M. and Chavannes, N.H. and De Smet, P.A.G.M. and Teichert, M.	10.2196/13551	Electronic inhalation monitoring devices (EIMDs) are available to remind patients with respiratory diseases to take their medication and register inhalations for feedback to patients and health care providers as well as for data collection in research settings. This study aimed to assess the validity as well as the patient-reported usability and acceptability of an EIMD.	Quantiative	Netherlands	Respiratory Patient	A total of 32 patients were included by 18 students (Table 1). In 3 pharmacies, no patients eligible and willing to participate were available. The mean age of the included patients was 48.1 years (range, 20-69 years), and 17 patients were female (53.1%). Medication adherence in the previous year calculated from dispensing data was 81.3%. Most patients used their medication twice a day (n=26). Three patients (9%) used an additional diary to note their daily inhalations. Twenty-five patients (78%) completed the questionnaires and provided individual comments on the EIMD (	n Electronic Monitoring Device in Respiratory Patient Care and Research			In the acceptability questionnaire, patients rated the EIMD generally as useful (76% neutral or agree) and 84% rated the EIMD program as not time-consuming (Table 2). With regard to recommending the EIMD to other patients, the majority rated this item as neutral (n=12, 48%). In addition, 80% of the patients indicated that they were not willing (n=15) or uncertain ("maybe," n=5) about continuing the program themselves, and 70% (n=14) of these patients reported that they had only participated in this study because they wanted to help the students fulfil their assignment and facilitate the research. The five patients who were positive about continuation reported that they participated mainly to gain personal insight into their inhalation patterns. The patients generally felt positive about the pharmacists' role in counselling.<br>Some of the previously mentioned technical issues reported by some patients resulted in a level of frustration or confusion because of missed or unnecessary reminders due to missing data, which impacted their acceptance of the app (Textbox 1). The reminders and motivational messages were appreciated by 9 and 4 patients, respectively.		The majority of patients indicated that the app was easy to use and not unnecessarily complex; the mean SUS score was 68.9 (SD 11.34; range, 52.5-90).		Several patients suggested more individualization of the settings in the app, such as inclusion of a personal choice to share data with a specific health care provider and management of their dose regimen settings.					N
Perception of older adults toward smartwatch technology for assessing pain and related patient-reported outcomes: pilot study	2019	JMIR mHealth and uHealth	Manini, T.M. and Mendoza, T. and Battula, M. and Davoudi, A. and Kheirkhahan, M. and Young, M.E. and Weber, E. and Fillingim, R.B. and Rashidi, P.	10.2196/10044	Chronic pain, including arthritis, affects about 100 million adults in the United States. Smartwatches can be used as digital ecological momentary assessment (EMA) tools for real-time collection of pain scores.The aim of this study was to explore the attitudes and perceptions of older adults towards design and technological aspects of a smartwatch framework for measuring patient report outcomes (PRO) as an EMA tool.	Mixed	USA	Older Adults	Characteristics Total Female Male P value<br>Participants, n (%) 19 14 (74) 5 (26) .01<br>Age (years), mean (SD) 72.7 (6.1) 72.0 (6.7) 75.5 (5.8) .22<br>Access to Wi-Fi, n (%) 17 (89) —a — —<br>Own a smartphone, n (%) 14 (74) — — —<br>Own a smartwatch, n (%) 1 (5) 1 (7) 0 (0) 1	Older Adults Toward Smartwatch Technology for Assessing Pain and Related Patient-Reported Outcomes			The overall attitude of the participants toward the smartwatch technology was positive. They showed interest in the direct phone-call capability, availability of extra apps such as the weather apps and sensors for tracking health and wellness such as accelerometer and heart rate sensor. Nearly three-quarters of participants showed willingness to participate in a one-year study to wear the watch daily.		Concerns were raised regarding usability, including accessibility (larger icons), notification customization, and intuitive interface design (unambiguous icons and assessment scales).	Participants expressed interest in using smartwatch technology for PRO assessment and the availability of methods for sharing data with health care providers.						N
Complementing a clinical trial with human-computer interaction: Patients?user experience with telehealth	2019	JMIR Human Factors	Jalil, S. and Myers, T. and Atkinson, I. and Soden, M.	10.2196/humanfactors.9481	We investigate how the user experience during a clinical trial can be researched and what a clinical trial can learn from human-computer interaction research.	Qualitative	Australia	Diabetes patients	Participation in the CUE was voluntary. A total of 12 patients initially agreed to participate. However, three of them opted out of the CUE study because they were not available during the designated time frame. Nine patients participated in the CUE study. Five of them were considered part of the aging population with an age of at least 64 years, and four participants were within the age range of 50 to 63 years (Table 3). Participants were given pseudonyms that were incredibly different from the participants' original names. In addition to the nine participants, five family members occasionally provided feedback. Of these five, only two family members permitted us to use their quotes.	This research investigates the UX of patients with type 2 diabetes mellitus (T2D) with a telehealth in-home monitoring device to manage T2D from home.					Lack of Wireless Capability The device only functioned with wired internet that had to be connected through a cable through the telephone port in a patient's house. Heidi, Serena, and Uma mentioned that having wires was a problem of the device:<br>Lack of Visual Data In the current system, each time the patients conducted a test, they were presented with instant data on their blood pressure and blood glucose levels. However, when the patients conducted the next scheduled test, they could not see the previous data.<br>Lack of Medication NThis meant that the database did not contain a full list of all possible diabetes medications that the patients in this clinical trial were using.<br>Mismatch With Life Due to Immobility of the Device The device currently works only with internet cables. All the patients stated that a mobile unit would have been much more suitable than the current device.<br>Glucometer Discomfort and Pain When a patient uses a glucometer, a small drop of blood is obtained by pricking the skin with a lancet. The drop of blood is placed on a disposable test strip that the meter reads and uses to calculate the blood glucose level. Slight discomfort is experienced when the lancet pricks the skin of the finger.<br>Patients had frustrations using the device due to slow responses and sometimes during unresponsive states. Even after participating in the clinical trial for more than 3 months, the patients often had problems with the device.Difficulty in Measurement	Motivation Two participants, Vince and Heidi, mentioned that using the device motivated them to manage their diabetes:<br>Build a Habit Pete lives alone, and he stated that he had developed a habit from using the device for 6 months in the clinical trial.<br>Awareness Enrollment in the clinical trial had made Serena aware of her well-being. The device would make her do things regularly.				Feel Safe Daily monitoring provided safety and comfort to the patients.<br>Reduced Doctor Visits Patients stated that they had fewer visits to the doctor during the time enrolled in the clinical trial. They indicated that they did not have to see a doctor every 3 months, which is the traditional treatment. Instead, they spoke with the nurse every 2 weeks, which decreased the doctor visits unless there was something urgent.		N
Real world usage characteristics of a novel mobile health self-monitoring device: Results from the Scanadu Consumer Health Outcomes (SCOUT) study	2019	PLoS ONE	Waalen, J. and Peters, M. and Ranamukhaarachchi, D. and Li, J. and Ebner, G. and Senkowsky, J. and Topol, E.J. and Steinhubl, S.R.	10.1371/journal.pone.0215468	We sought to identify factors associated with consistent long-term use of the Scanadu Scout multi-parameter vital sign monitor among individuals who invested in the device through a crowd-funding campaign.	Quantitative	USA	General public	Of the 4525 individuals invited to participate, 4000 subjects signed the informed consent and 3872 completed the baseline survey (Fig 3). Participants' mean (standard deviation) age was 47 (13) years; 84% were men, most were married or had a domestic partner (66%) and had a bachelor's or graduate degree (79%). Overall, 45% reported having at least one current health problem (Table 2). The cohort reported high levels of medical device use, with more than 75% of participants using a device (which included scales, thermometers and home blood pressure monitors) at least once a week. Use of "smart wellness" technologies was also prevalent, with more than 50% reporting consistent use of an activity tracker and 43% using mobile health apps (Table 2). The majority agreed that mobile devices were consistent with their approach to health (87%) and could help them stay healthier (70%) (S1 Table). Compared with men,	a novel mobile health self-monitoring device			Follow-up surveys answered by a subset of participants showed that at 12 months the majority of participants reported satisfaction with device use, with approximately 80% reporting the device was easy to use and 87% stating they would be willing to participate in a study testing the device again (Table 4). Overall, 28% of participants reported making changes to their diet and 34% made changes to their exercise routine. Despite continuous recommendations to the contrary, 26% of users reported making medication or supplement changes based on device results, 60% of whom reported consulting a doctor or other health care provider about the change (Table 4). However, there were no adverse events reported by any of the users in association with the medication changes. At 12 months, consistent long-term users of the device were statistically significantly more likely than the rest of the cohort to report sharing results from the device with their doctors or other healthcare provider (59% vs. 29%), that the device was easy to use (94% vs. 80%), that they were able to complete the scan to obtain measurements, that they would recommend the device to family and friends and that they would be likely to participate in a study of the device again (Table 4). When included in a logistic regression model controlling for the statistically significant baseline characteristics, ease of use and sharing results with healthcare providers remained independent predictors of consistent long-term use (S3 Table).	Comparing baseline characteristics of the consistent long-term users of the device—participants who were in the trial for at least 26 weeks with recordings during 80% of those weeks and averaging 3 or more uses per week, n = 93 (2.7% of the study population)—with the rest of the participants, consistent device users were statistically significantly older (mean age 56 years vs. 47 years) and more likely to live in a household without children (74% vs. 64%), use other medical devices at least once daily (45% vs. 26%), and report at least one current medical problem (54% vs. 45%) (Table 3). There were no differences in measures of health-related quality of life. In a multivariable logistic regression model of the association with frequent device uses which included all 4 variables, current medical problem was no longer statistically significant (S3 Table).	Participants who responded to all three of the follow-up surveys (n = 706) showed a marked change in the perception of the amount of time required to use the device and the distraction it caused, with approximately 60% reporting the device "interrupted my daily activities" and "required too much of my time" at 3 months decreasing to less than 20% at 12 and 18 months (S5 Table).					Percent reporting diet and exercise changes increased significantly between 3 months and 12 months, plateauing at 30% and 36% at 18 months respectively.		UNDECLARED
Acceptability and feasibility of implementing accelorometry-based activity monitors and a linked web portal in an exercise referral scheme: feasibility randomized controlled trial	2019	Journal of Medical Internet Research	Hawkins, J. and Charles, J.M. and Edwards, M. and Hallingberg, B. and Mcconnon, L. and Edwards, R.T. and Jago, R. and Kelson, M. and Morgan, K. and Murphy, S. and Oliver, E.J. and Simpson, S.A. and Moore, G.	10.2196/12374	Exercise referral schemes (ERSs) are recommended for patients with health conditions or risk factors. To determine the feasibility and acceptability of implementing an activity-monitoring device within the Welsh National ERS to inform a decision on whether and how to proceed to an effectiveness trial.	Quantitative	UK	General public	16.3) years and mostly female (101/156, 64.7%) and white (Table 2). There was substantial socioeconomic bias in uptake, with more than half of recruited participants residing in the least deprived quintile of Wales.Income, nb (%)<br>Less than £5000/year 4 (5) 4 (7) 8 (5.5)<br>£5000-£9999 7 (8) 11 (18) 18 (12.4)<br>£10,000-£15,499 22 (27) 15 (24) 37 (25.5)<br>£15,500-£20,999 18 (22) 10 (16) 28 (19.3)<br>£21,000-£30,999 12 (15) 10 (16) 22 (15.2)	Activity Monitors			At T1, 93% (79/85) of participants reported understanding the use of a control group, whereas 84% (72/86) either agreed or strongly agreed that it was acceptable to only give the MWK to half of the participants and 96% (82/86) either agreed or strongly agreed that it was acceptable that the MWK was given to half of the participants at random. Despite high acceptability of randomization in quantitative data, the staff reported that some clients were disappointed by control group allocation. Although the use of other devices was similar across arms, interview data from intervention participants suggested that some might have bought a different activity monitor if they had been allocated to the control group.		Use of both intervention components was reported by approximately half of the participants, though in both cases this diminished over time. At T2, 57% of intervention participants (31/54) reported using the MWK at some point during the study. However, only 8% (4/49) had used it in the past month. Just under half reported using the MWC at some point during the study, with only 6% (3/47) having used the MWC within the past month. See Multimedia Appendix 2 for a summary of mean scores for acceptability and usage questions. Patient interviews suggested that some engaged with the device initially but stopped owing to a combination of device malfunctions and the novelty factor wearing off:	A small majority (26/46, 57%) reported that the device met their expectations in terms of motivating them to be physically active; qualitative data suggest that the reasons it did not meet expectations were linked to the issues reported above:	The proportion of patients reporting that they would use either device in future if they could was 37% (17/46) for the MWK and only 15% (7/46) for the MWC. In the qualitative interviews, participants suggested that they would be more likely to use the intervention in the future if it was easier to understand, technical issues were addressed, and it had better wearability:	Other factors influencing engagement included lack of access to a computer and/or internet, poor IT literacy, and technical issues with charging and syncing the device, sometimes highlighting reliance on a relative or the instructor to support continued use				N
Smart technology for healthcare: Exploring the antecedents of adoption intention of healthcare wearable technology	2019	Health Psychology Research	Chau, K.Y. and Lam, M.H.S. and Cheung, M.L. and Tso, E.K.H. and Flint, S.W. and Broom, D.R. and Tse, G. and Lee, K.Y.	10.4081/hpr.2019.8099	We used self-administrated online survey to explore adoption intentions of healthcare wearable devices in 171 adults residing in Hong Kong.	Quantitative	Hong Kong, China	General public	The survey was promoted using a convenience sampling approach by e-mail to contacts and promoted on social media in Hong Kong by the research team. Data collection took place from October 1st 2018, to January 5th 2019) a total of 14 weeks. To ensure the validity of our study, we designed several screening questions to assess the accuracy of respondent's knowledge of healthcare wearable technology. The screening questions include: "Have you ever bought a healthcare wearable technology product?", "Have you ever used wearable technology product?" and "Have you ever read product reviews about healthcare wearable technology products on website/ social media platforms/ blogs / forums?". Respondents without experience of healthcare wearable technology were excluded from the current study. After exclusion, a total of 310 consumers were invited to participate in the survey. In total, 237	healthcare wearable technology			As presented in Figure 2, the results support three of the five hypotheses, whereas the remaining two hypotheses are marginally accepted. Regarding the antecedents of perceived usefulness, the impact of perceived irreplaceability on perceived usefulness was the strongest (β=.437, p<.001), followed by perceived convenience (β=.205, p<.05), supporting H2 and H3. Additionally, the impact of perceived credibility on perceived usefulness is strong and marginally significant (β=.227, p<.10). Thus, H4 was marginally accepted. Regarding the antecedents of adoption intention, the impact of perceived usefulness on adoption intention was strong and significant (β =.728, p<.001), supporting H1. Finally, the impact of health belief on adoption intention was marginally significant (β=.126, p<.10). Therefore, H5 was marginally supported.		Furthermore, the results also presented the indirect effects of the exogenous variables, including perceived convenience, perceived credibility and perceived irreplaceability on adoption. In particular, the indirect effects of perceived irreplaceability (β=.318, p<.001) and perceived convenience (β=.149, p<.05) on adoption intention were strong and significant, whilst the indirect effects of perceived credibility (β=.165, p<.10) on adoption intention was marginally significant.							UNDECLARED
A look at the security and privacy of Fitbit as a health activity tracker	2019	ACMSE 2019 - Proceedings of the 2019 ACM Southeast Conference	Orlosky, J. and Ezenwoye, O. and Yates, H. and Besenyi, G.	10.1145/3299815.3314468	In this paper, we present the results of a study with 24 participants who used and evaluated a small form factor personal health device, the Fitbit Blaze.	Quantitative	USA	General public	Ourexperimentwaswithin-subjects,including24participants(13male) with anaverageageof25.73(stdev3.61).Participantswerescreenedto ensurelowcardiovascularrisk,abodymassindex(BMI)under29, andtheabilitytocompletephysicalactivityonatreadmill.Asaway topreventexposureoftheparticipantstoadditionalsecurityrisks, experimentersguidedparticipantsthroughthesetupofadummy	Fitbit											Primaryresultsofthesubjectiveratingswerethat securityconcernsoutweighedprivacyconcerns andthatparticipantsweremore willingtousethetrackerforresearchthanforpersonaluse.<br>Relevantcommentsfromthefocusgroupsindicatedthatparticipantswereuncomfortablewith Fitbit'sapproachofusingsocialnetworkprofilestoidentifyfriends,wereunsurehowmuchoftheir datawouldbesharedwith3rdpartyservices,and wereespecially concernedaboutlocationdatabreachesandmisuse.Participants indicatedtheywouldbecomfortablesharingFitbitdatawithmedicalandresearchentities,butcommercialapplicationofFitbitdata suchasforinsurance,advertising,orworkplacewellnessmetwith resistanceforfearofabuse.	UNDECLARED
Information needs for designing a home monitoring system for children with medical complexity	2019	International Journal of Medical Informatics	Nkoy, F.L. and Hofmann, M.G. and Stone, B.L. and Poll, J. and Clark, L. and Fassl, B.A. and Murphy, N.A.	10.1016/j.ijmedinf.2018.11.011	Background Children with medical complexity (CMC) are a growing population of medically fragile children with unique healthcare needs, who have recurrent emergency department (ED) and hospital admissions due to frequent acute escalations of their chronic conditions. Objective To: 1) assess information needs for operationalizing CMC home monitoring, and 2) determine technology design functionalities needed for building a mHealth application for CMC.	Quantitative	USA	children with medical complexity and caregiver	Thirteen primary caregivers of CMC participated in 3 focus group sessions, including 4 in the first, 5 in the second and 4 during the last session. Participants were mostly female (2 males), Caucasian (1 Asian), and married (1 single). Caregivers included one mother of two children, a mother/father dyad, a grandmother, a father, and 9 mothers of 14 children; five of the children were adopted. Overall, the 13 CMC of these participants were between ages 18 months and 19 years (mean = 9 years), most of them had cerebral palsy due to prematurity, anoxic encephalopathy, congenital infections or structural anomalies; one had a genetic syndrome; all had functional limitations in mobility, self-care, and communication. The medical complexity of this sample was high, including having private duty nursing (n = 7), gastrostomy tubes (n = 13), tracheostomies (n = 5), and seizures (n = 9). Of these 13 CMC, there	a home monitoring system for children with medical complexity			Based on their responses regarding perceptions of common symptoms preceding ED/hospital admissions in their CMC, parents agreed with our proposition to track (through the CMC home monitoring system) key measures (e.g., oxygen saturation, temperature, heart rate, respiratory rate, pain, feeding, mental status, seizures, and overall caregiver worry about the child's health) in order to monitor and identify early changes in symptoms that may signal deteriorations.				Parents universally stated that home monitoring with a tracking tool for their children would be valuable if it were also to provide access to a HCP for quick evaluation and management of their child's symptoms, and thereby keep the child at home whenever possible.<br>Overall, participants offered practical suggestions for enhancing usability of a new home monitoring system for CMC, and identified 5 key functionalities: 1) symptom tracking (for the child only not parents) of commonly reported drivers of ED/hospital admissions, recording baseline, fluctuations, and especially during early periods of symptom escalations, rather than daily; 2) user friendly, with attractive features to facilitate data entry, such as the use of voice, radio buttons, and drop down menus; 3) a free-text field for caregivers for providing reasons for child's worsening symptoms with a summary of interventions attempted at home; 4) ability to provide direct access (integration) to a HCP via text and/or email messaging, and to allow real-time sharing of child data with the HCP to facilitate care; and 5) ability to upload and post a photo or video and allow visual recall of the child by his/her HCP as well as the child's current appearance to facilitate safe care.					N

A clinic-based tablet application to support safer conception among HIV serodiscordant couples in Kenya: feasibility and acceptability study	2019	mHealth	Velloza, J. and Ngure, K. and Kiptinness, C. and Quame-Amagl, J. and Thuo, N. and Dew, K. and Kimani, M. and Gakuo, S. and Unger, J.A. and Kolko, B. and Baeten, J.M. and Celum, C. and Mugo, N. and Heffron, R.	10.21037/mhealth.2019.01.04	The objective of this analysis is to examine the feasibility and usability of short message service (SMS) messages and a mobile application to support safer conception for HIV serodiscordant couples.	Mixed	Kenya	HIV serodiscordant couples	We enrolled 74 HIV serodiscordant couples, including 34 (45.9%) couples with an HIV-infected female partner. The median age of the female partners at enrollment was 30 years [interquartile range (IQR), 27–35 years; Table 1). All HIV-uninfected participants accepted PrEP at enrollment and all participants living with HIV were on ART by their 1-month follow-up visit. A total of 143 (96.6%) participants had at least one follow-up visit, and 47 pregnancies and 0 HIV infections were diagnosed during follow-up.	A clinic-based tablet application to support safer conception among HIV serodiscordant couples in Kenya			Qualitative data revealed that the SMS messages and SCIP-App were feasible and acceptable for fertility tracking and were recommended for broader use within their community. Four main themes emerged as described below		Ease of responding to SMS and incorporating SMS into daily life<br>Participants overwhelmingly found the messages easy to respond to because they were asking about "how I do the tests".<br><br>The basal body temperature question was the most difficult to respond to because it required participants to type in multiple digits and include a decimal place.<br><br>They appreciated being able to choose their preferred language ("I chose Kikuyu so that I can understand it better", HIV-infected female, age 37). Participants did report technical issues receiving SMS messages if "Safaricom is having problems" or they were "in rural areas where there are problems with electricity". Messages could be reportedly delayed anywhere between a few hours and three days, but "when there are delays someone is informed". Participants appreciated communication with the study staff about Safaricom service outages.				Women reported that the SMS messages "reminded me to do the [ovulation] tests" and served as a useful record of their fertility indicators over time, which they appreciated if they did not use paper records or if they forgot to update their paper records<br><br>In addition, the daily messages made participants feel like "there are people who are concerned about me" and increased their satisfaction with clinic services and engagement in the safer conception intervention<br><br>SCIP-App was also useful for retrospectively tracking participants' fertility indicators, PrEP use, and viral load, predicting upcoming peak fertility days, and improving their understanding of their fertility during monthly clinic visits.Other participants enjoyed looking at the application with the counselor every month as a way to "increase my knowledge" about fertility indicators and share their data back with them . Men found SCIP-App to be helpful for "understanding a woman's fertility" and they reported that the mHealth tools "educated" them and helped them to communicate with their partner about peak fertility timing and safer conception strategies.	Concerns around confidentiality and disclosure of SMS messages<br>A few participants reported concerns about answering the messages in front of other people because they did not want questions about sexual behavior to be seen. However, these concerns were relatively minimal and were not specifically related to HIV status disclosure. Concerns around confidentiality also arose when participants visited the clinic and saw their information on the tablet application, although this appeared to be more of an issue among older participants and those who did not have smart phones:<br><br>One participant described an incident where her brother saw the messages on her phone and then she had to tell him about the study, but this was the only instance of unintended disclosure reported. Several participants said they deleted the SMS messages from their phones or requested to receive the messages in the evening when they were likely to be	N
The feasibility of a using a smart button mobile health system to self-track medication adherence and deliver tailored short message service text message feedback	2019	JMIR Formative Research	Ellis, R.J.B. and Hill, J.H. and Kerley, K.D. and Sinha, A. and Ganci, A. and Russell, C.L.	10.2196/13558	The study aimed to evaluate the feasibility and acceptability of using an mHealth system for medication adherence tracking and intervention delivery.	Quantitative	Indiana	patients with CKD	A total of 5 patients with CKD, stages 1-4, were enrolled in the study, with the majority being men (60%), white (80%), and Hispanic/Latino (40%) of middle age (52.6 years, SD 22.49; range 20-70).	a Smart Button Mobile Health System to Self-Track Medication Adherence and Deliver Tailored Short Message Service Text Message Feedback					A total of 2 participants reported technology problems during the study. Troubleshooting included ensuring the phone app was open and Bluetooth was turned on, as well as confirmation that the smart button was pressed when taking pills. Although each participant gave confirmation, messages were still not received by these 2 participants. A total of 1 participant texted the RA study phone to troubleshoot, as SMS text messages were not received, but the phone number the participant texted from did not match the phone number provided by the participant at study enrollment. The participant validated that only 1 mobile number was used for both voice and SMS text messages. This problem remained unresolved.						N
Development of an ambulatory biofeedback app to enhance emotional awareness in patients with borderline personality disorder: Multicycle usability testing study	2019	JMIR mHealth and uHealth	Derks, Y.P.M.J. and Klaassen, R. and Westerhof, G.J. and Bohlmeijer, E.T. and Noordzij, M.L.	10.2196/13479	The aim of the study was to develop an ambulatory biofeedback app for mental health care that helps with learning to better recognize changes in personal emotional arousal and increases emotional awareness.	Mixed	Netherlands	Patients with Borderline Personality Disorder		Ambulatory Biofeedback App to Enhance Emotional Awareness in Patients with Borderline Personality Disorder			The patients rated the system after the second iteration of the first cycle. The overall rating was good (average score of 78.8). Of 4 participants, 3 rated the app as good to excellent (85 to 97.5), whereas one rated it as poor (42.5) [38]. The latter participant experienced trouble in working with the mobile phone and smartwatch in general and indicated that her age could play a role. She would therefore have liked to see the user interface simplified and more foolproof. She also would have liked to have an easy to understand user manual to come with the app.<br><br>Technology Overall, patients were positive about the ease of use of the app on the smartwatch and mobile phone. They also liked the design and indicated they had no problems with having to wear a smartwatch—although not all of them were used to wearing a watch. Moreover, 3 patients found it cumbersome that the app was on a mobile phone of the project, so they had to carry it besides their own device. Given the test phase, all understood why the app was not installed on their own devices.		The reliability of the app was perceived as average to slightly unreliable.	All patients experienced the app as discreet and inconspicuous. All would have liked more data visualization. They stressed it was important that the data visualization should remain neutral for reasons of discreetness while still being intuitively understandable. Regarding the Watch face GUI, users found it important that they could choose from several designs.<br><br>Patients stressed that the app should be compatible with their therapies. This need was already addressed, as physiological arousal was represented in a two times five stage process that is compatible with the current user setting (DBT and systems training for emotional predictability and problem solving [3,42]) as well as cognitive behavior therapy (CBT). Patients indicated that other ways of breaking down in levels would still be welcome for the use of the app in other forms of therapy. One of the patients believed that the levels of the physiological scale were representing the subjective emotional states that were used in the main model on emotions in therapy.<br><br>The timing and frequency of feedback given by the app (via the vibration motor in the smartwatch) yielded mixed reactions. One patient said the notifications matched the perceived moments of arousal well. For 2 patients, the frequency of notifications was acceptable. One patient received a bit too many notifications, especially since they were accompanied by a vibration of the smartwatch that was noticed by other				Regarding privacy, all patients answered that future sharing of the information generated by the app with their therapists would not be an issue for them.	N
Validation and acceptability of a cuffless wrist-worn wearable blood pressure monitoring device among users and health care professionals: Mixed methods study	2019	JMIR mHealth and uHealth	Islam, S.M.S. and Cartledge, S. and Karmakar, C. and Rawstorn, J.C. and Fraser, S.F. and Chow, C. and Maddison, R.	10.2196/14706	Blood pressure (BP) is an important modifiable cardiovascular risk factor, yet its long-term monitoring remains problematic.This study aimed to validate a wrist-worn cuffless wearable BP device and assess its acceptability among users and health care professionals.	Mixed	Australia	hypertensive patients	Male, n (%) 10 (50)<br>Age (years)<br><br>Mean (SD) 20.3 (5.4)<br>Range 37.2-18.5	a Cuffless Wrist-Worn Wearable Blood Pressure Monitoring Device Among Users					Although the home BP monitoring device was simple to use and did not interrupt daily activities, participants identified that obtaining BP measurements was reliant on them taking action and initiating a measurement. Most participants preferred the unobtrusive design and automated measurements of the wearable BP device.	Although additional wearable device parameters such as heart rate and sleep quality were not formally considered in this study, participants liked being able to view these data on the device display and/or in the smartphone app.	Barriers to using the wearable device included difficulty in obtaining a good fit for people with small wrists, the need for regular charging, and a motion-activated light that woke some participants during sleep.			Although both users and health care professionals identified many advantages of the wearable BP device compared with other devices, accuracy was a consistent theme identified by both participant groups. Terms such as validity, consistency, and reliability came up frequently in both groups, demonstrating the strong presence of this theme.<br><br>Most health care professionals described using and encouraging home BP monitoring for their patients. They acknowledged using patient-reported BP data when available, but they reported concerns regarding its reliability. When asked about the use of a wearable BP device by patients, all health care professionals expressed interest in this method as long as accuracy could be demonstrated. Health care professionals foresee that the benefits of a wearable device could include convenience (small size and regular automated measurements) and improved adherence to monitoring.	N
Using wearable physiological monitors with suicidal adolescent inpatients: Feasibility and acceptability study	2019	JMIR mHealth and uHealth	Kleiman, E. and Millner, A.J. and Joyce, V.W. and Nash, C.C. and Buonopane, R.J. and Nock, M.K.	10.2196/13725	This study aimed to evaluate the feasibility and acceptability of using a wearable behavioral and physiological monitor, the Empatica E4, to continuously assess a group of suicidal adolescent inpatients.	Mixed	USA	suicidal adolescent inpatients	Participants' age ranged from 12.5 to 18.6 years (mean 16.3, SD 1.6), 78% (39/50) of the sample was female and 92% (46/50) of the sample was white (4%, 2/50 was Asian and the remainder indicated that they identified with another race). Participants together provided 487 total days of data (mean 9.74 days per participant, SD 13.81 days, range 1-76 days). The average length of stay in the hospital (ie, day of intake to day of discharge) was 10.7 days (SD 13.86 days, range 1-77 days). There were no demographic differences by age or sex (there were too few cases to examine racial differences) on any key study variables including hours wearing the monitor per day (age: r=0.05, P=.77 sex: t11.12 (two-tailed)=0.13, P=.90), number of button presses (age: r=0.10, P=.58; sex: t23.94 (two-tailed)=1.26, P=.22), and dropout status (age: t5.81 (two-tailed)=0.09, P=.93; sex: χ21=0.8, P=.38).	Using wearable physiological monitors with suicidal adolescent inpatients					On average, across nearly all items assessing comfort, participants tended to rate their discomfort wearing the monitor below 5 out of 10 (with 10 meaning more discomfort). As can be seen in the middle columns of Table 1, there was a significant negative correlation between hours worn and ratings of how uncomfortable the monitor was, such that the more participants rated the monitor as uncomfortable, the fewer hours they wore it. No other correlations were significant after correcting for multiple comparisons. As can be seen in the rightmost columns of Table 1, after correcting for multiple comparisons, there were only 2 significant findings: (1) those who dropped out were significantly more likely to rate the device as uncomfortable and (2) were more likely to note that they could feel the device.				Positive/neutral statements about device<br><br>Felt OK when wearing monitor "No different than normal." 0.47 5.62 35 (7)<br><br>Could tolerate negatives "It isn't very comfortable but I managed." 0.58 6.84 5 (11)<br><br>Something positive about monitor's looks "It looked cool." 0.76 9.05 5 (11)	Complaints about monitor<br><br>Discomfort/uncomfortable in general "It felt extremely uncomfortable." 0.68 8.08 22 (47)<br><br>The monitor was too big/clunky "It was bulky and inconvenient." 0.9 10.74 17 (36)<br><br>It should have a clock "I would probably make it have a clock." 0.92 10.89 11 (23)<br><br>The material was uncomfortable "I'd make the wristband out of a thinner material." 0.41 4.83 7 (15)<br><br>Discomfort sleeping/at night "Was uncomfortable during sleep." 0.92 10.93 5 (11)<br><br>EDAb sensors were uncomfortable "Circular sensors are too big and rub too much." 1 11.87 3 (6)<br><br>The monitor does not look good "It could be	N
Factors Affecting Caregivers?Acceptance of the Use of Wearable Devices by Patients With Dementia: An Extension of the Unified Theory of Acceptance and Use of Technology Model	2019	American Journal of Alzheimer's Disease and other Dementias	Dai, B. and Larnyo, E. and Tetteh, E.A. and Aboagye, A.K. and Musah, A.-A.I.	10.1177/1533317519883493	This study aims to examine the factors affecting caregivers?acceptance of the use of wearable devices by patients with dementia by extending the Unified Theory of Acceptance and Use of Technology (UTAUT) model with 2 additional constructs: resistance to change (RC) and technology anxiety (TA).	Quantitative	sub-Saharan Africa	Patients With Dementia	Table 2. Age and Years of Experience Distribution.<br>N = 350 Min Max Mean Standard Deviation<br>Age 20 70 32.88 12.21<br>Years of experience handling people with dementia 2 30 4.72 2.31 N = 350<br>Description Frequency Percentage<br>Gender Male 137 39.10<br>Female 213 60.90<br>Marital status Single 217 62.00<br>Married 114 32.60<br>Divorced 9 2.6<br>Legally separated 9 2.6<br>Widowed 1 0.30<br>Educational level BSc 146 41.70<br>Diploma 188 53.70<br>Certificate 13 3.70<br>No formal education 3 0.90	Wearable Devices by Patients With Dementia				The results of the extended model show that social influence, effort expectancy, facilitating conditions, and behavioral intention (BI) all had P < .05, thus were statistically significant in explaining the perceived BI and actual use of health-care wearable devices among patients with dementia as reported by caregivers. However, P value for RC and TA was greater than .05, hence were insignificant.							N
The Effect of Security, Privacy, Familiarity, and Trust on Users' Attitudes Toward the Use of the IoT-Based Healthcare: The Mediation Role of Risk Perception	2019	IEEE Access	Alraja, M.N. and Farooque, M.M.J. and Khashab, B.	10.1109/ACCESS.2019.2904006	This paper explores users' attitudes towards using IoT technologies to receive healthcare services.	Quantitative	Oman	General public	The population of the research included all residents of Oman, whether as citizens or expatriates, aged between 18 years and 60 years. The population of Oman was stated in NCSI in 2018 to be 4,654,722. Of this number, 55.9 per cent were Omani while the other 44.1 per cent were expatriates [69]. However, this study targeted only owners of smart phones which had an available Internet connection.	the Use of the IoT-Based Healthcare				and familiarity affected trust in the IoT. Furthermore, these levels of trust in the IoT were found to affect both users' perceptions of risk in, and their attitude towards, using the IoT. The users' risk perception partially mediated the relations between users' trust and their attitude regarding use of the IoT. The framework was supported and interpreted by 40 per cent of the variance in the attitude towards using the IoT in healthcare, while the mediator showed 47 per cent of the variance in the attitude towards using the IoT in healthcare.							UNDECLARED
What Do Users like About Smart Bottle? Insights for Designers	2019	Lecture Notes in Computer Science (including subseries Lecture Notes in Artificial Intelligence and Lecture Notes in Bioinformatics)	Poh, Z. and Chong, C.Y. and Teh, P.-L. and Joseph, S. and Huey, S.L.W. and Ramakrishnan, N. and Parthiban, R.	10.1007/978-3-030-22580-3 24	The study aims to explore the young bottles users' needs and requirements of smart bottle through focus groups discussions as well as to propose a system design of smart bottle that integrates with the fluid intake monitoring system, medical adherence system and storage compartment.	Qualitative	Australia	General public	Gender<br>Male 9 64.29%<br>Female 5 35.71%<br>Age group 18–20 6 42.86%<br>21–30 7 50.00%<br>31–40 1 7.14%<br>Education<br>High school 9 64.29%<br>Bachelor degree/professional qualification 1 7.14%<br>Master/PhD 4 28.57%	Smart Bottle			Additionally, participants discussed about their current usage of the water bottle and their medication or supplement intake. All the participants (100%) carried their water bottle around with them wherever they went so that they could have water by their side all the time. This is particularly common for people living in tropical climate such as Malaysia and Singapore.		During the focus groups discussions, a prototype of water bottle, connected to a mobile application was shown to the participants (Fig. 1). Majority of the participants (92.86%) found the app to be user friendly and simple, but several (7.14%) participants thought that the app had too many pages. Participants also provided feedback to improve the app. Fifty-seven percent of the participants thought that the app should have more colors to look more attractive. Fourteen percent of the participants suggested the font to be larger, while 28.57% thought that there should be more options in the settings such as body measurements or lifestyle.Participants also suggested several new ideas to improve the reminder feature. While many participants (78.57%) did not have additional comment on the improvement on reminder part, 7.14% had suggested to have a reminder on refilling water, another 7.14% proposed to connect the reminder through a wearable device (Fitbit), while the remaining 7.14% recommended to have an interactive screen or Artificial Intelligent such as Siri on the water bottle for communication. In regard to having medicine reminders, 71.43% of the participants viewed that it is impractical to be integrated with a water bottle. This was mainly because they were young adults and did not have the need to take medicine. However, 14.29% of the participants recommended the medicine reminder to be held on the app, 7.14% suggested on the water bottle and 7.14% suggested on both app and water bottle.	All 14 participants expressed their views on the design of water bottle. In terms of bottle size, most of the participants (64.29%) used bottles that can hold at least one liter of water, compared with 1.5 L and less than 1 L. Eighty-six percent of the participants suggested tall slim bottle shape and the remaining 14% of the participants recommended flat surfaced bottles.<br><br>There was a preference for non-slip grips, as the weight of the water bottle when filled, will not cause it to fall. Forty-three percent of the participants suggested curved design grip, 35.71% participants proposed rubber grip and 21.43% recommended bottle handle. For the material of the water bottle, there was a preference for plastic bottles as it is more durable and impact resistant compared to aluminum bottles. Ninety-three percent of the participants prefer a plastic bottle to other materials such as aluminum or metal. Durability of the bottle was one of the features that most of the participants valued. They preferred bottles that are sturdy and those that do not break, dent or are fragile. All the 14 participants would opt for a sturdy water bottle.<br><br>For cap designs, there was a preference for screw caps as it was more secure and leak proof. Ninety-three percent of the participants prefer screw caps, while none of the participants like flip top, and 7.14% likes a straw water bottle. Participants used different water bottle brands, namely Bros and Tupperware are relatively popular. Thirty-six percent					UNDECLARED
More than just friends: In-home use and design recommendations for sensing socially assistive robots (SARs) by older adults with depression	2019	Paladyn	Randall, N. and Bennett, C.C. and [illegible]banovic, S. and Nagata, S. and Eldridge, L. and Collins, S. and Piatt, J.A.	10.1515/pjbr-2019-0020	As healthcare turns its focus to preventative community-based interventions, there is increasing interest in using in-home technology to support this goal. This study evaluates the design and use of socially assistive robots (SARs) and sensors as in-home therapeutic support for older adults with depression.	Qualitative	USA	Older Adults with Depression	Participant recruitment took place in a midwestern city in collaboration with a local outpatient mental health clinic. The recruitment criteria for older adults specified participants should be over 55, live independently in their own homes, have an active depressive disorder diagnosis, and present with a co-occurring physical illness. We recruited participants who were over 55, instead of 65, as people with depression show cognitive signs of aging earlier than those without depression [47]. Those who had also been diagnosed with psychosis (e.g., schizophrenia) or dementia were not eligible for the study. In total, 10 older adults were recruited. They ranged from 56 to 67 years old, with an average age of 62.8 years. While recruitment of both male and female participants was attempted, no males volunteered for this study; therefore, all participants were female. This might be due to the larger overall incidence of	In-Home Use and Design Recommendations for Sensing Socially Assistive Robots (SARs) by Older Adults with Depression			Workshops showed older adults and clinicians saw several potential uses for robots and sensors to support in-home depression care. Long-term in-home use of the robot allowed researchers and participants to situate desired robot features in specific practices and experiences of daily life, and some user requests for functionality changed due to extended use.	Sensor data showed that participants' attitudes toward and intention to use the robot were strongly correlated with particular Circadian patterns (afternoon and evening) of robot use. Sensor data also showed that those without pets interacted with Paro significantly more than those with pets, and survey data showed they had more positive attitudes toward the SAR. Companionship, while a desired capability, emerged as insufficient to engage many older adults in long-term use of SARs in their home.							UNDECLARED
Older women's perceptions of a programmable video monitoring system at home: A pilot study	2018	Gerontechnology	Lapierre, N. and Meunier, J. and Arnaud, A.S. and Filiatrault, J. and Paquin, M.-H. and Duclos, C. and Dumoulin, C. and Rousseau, J.	10.4017/gt.2018.17.4.006.00	To monitor night walks and the person-environment interaction, a programmable video monitoring system (VS) was implemented at home. This study explores users' perceptions of this VS.	Qualitative	Canada	Older women	Participants were recruited in Montreal, between June 2015 and July 2016, according to the following selection criteria: (1) ≥ 65 years old; (2) ≥ one fall per year; (3) Woke up at night to go to the toilet; (4) Lived alone; (5) Had a standard ceiling height (≈243 cm); (6) Were either continent (no urine leakage during the past year) or incontinent (score ≥ 6 on the short form of the International Consultation on Incontinence Questionnaire and at least three urine leakages per week) (Avery et al., 2004). Women with diagnosed psychiatric disorders or using walking aids were excluded.	Older women's perceptions of a programmable video monitoring system at home	Most participants understood the recording based on movement detection in a chosen time slot. They all had a favourable opinion of this functioning		Before the experiment, all participants appreciated the look and small size of the VS. Participants mentioned that recording in a time slot preserved their privacy. One participant said: "It's good that it stops at some point. I'm not sure I would have participated otherwise; I would have been very hesitant" (F175). At the end of the experiment, participants still had a positive opinion of the system's operation. Recording in one time slot reassured them. One said: "I didn't have to worry about the cameras. In my mind, I trusted them not to work [during the day]" (F001). The recording on movement detection was a positive element; one participant said: "It [recording on movement detection] was good; I felt less spied on" (F054). Furthermore, the LED flashing when recording reassured participants that the system was operating properly. Participants appreciated customising the cameras' location; one said: "It's not intrusive at all. I could open cupboards easily. I didn't run into it. In the bedroom, it was in the corner" (F041). Regarding the customised time slot, one participant explained: "What had reassured me was that we had set hours [time slot]. Of course I wouldn't have undressed during the day; that's why I chose midnight to 5:00, because I was in bed at those times. (...) it doesn't affect my privacy." (F001) Conversely, one participant mentioned that the customisation of the VS was not important to her because she was out during the day. Instead, she appreciated the customisation of the implementation procedure and explained: "I chose the days and scheduling of the calls; you adapted to my schedule. (...) I didn't have to worry about wanting to leave and she [the first author] didn't call. (…) I didn't have any trouble with that." (F148)		On day 0, participants had positive opinions regarding the VS implementation. They mentioned feeling comfortable and safe:. However, one participant qualified this opinion saying: "I… feel comfortable, but I will keep an eye open, I will pay attention to what is happening" (F148). At the end of the experiment (day 8), participants reported a positive experience. Despite a rather positive opinion before the implementation, one participant found it difficult to accept the camera in her bedroom. However, two other participants' perceptions became more positive.	Video images were stored in each camera's SD card; participants preferred this rather than storage in the Cloud. One participant had difficulties understanding how the Cloud works and preferred storage in SD cards because it appeared easier. Another considered both storage types risky				Overall, before the experiment, participants reported no apprehension about being videotaped.However, some raised concerns such as dropping the camera or being filmed in the shower. (F041) Participants could see examples of the recorded images on day 2 and day 5 and noticed that images were already blurred because of the infrared vision; it reassured them that their privacy was respected. Influence on behaviour Some participants changed their behaviour during the experiment. One participant said that she did not shower in her bathroom for fear of being filmed while naked or damaging the VS by splashing it (see other examples in Table 2). However, two other participants said they had not changed their habits during the experiment.	N

Critical factors for adoption of wearable technology for the elderly: Case study of Thailand	2018	PICMET 2018 - Portland International Conference on Management of Engineering and Technology: Managing Technological Entrepreneurship: The Engine for Economic Growth, Proceedings	Srizongkhram, S. and Shirahada, K. and Chiadamrong, N.	10.23919/PICMET.2018.8481990	The research aims to determine the critical factors that affect the expectation of the customers, including the elderly and caregiver, to adopt wearable device technology.	Quantitative	Thailand	the elderly and caregiver.	The characteristics of the data are shown in Table III. 71 % of the respondents are female. The percentage of the customer groups are 33%, 17%, and 50% which correspond to the elderly, the formal caregiver, and the informal caregiver respectively. The number of respondents based on education background are as follows 1) 13% of Junior and High school. 2) 49% of Bachelor's degree. 3) 36% of Master's degree. 4) 2% of Doctor's degree. Before conducting the next step of the experiment, the result shows that the grouping of the questions is needed to be changed. Table IV. shows the new question grouping. The factor highlighted in blue are removed due to question repetition. The hypothesis model in Fig. 4 is deemed inappropriate. A new hypothesis model was tabulated (Fig. 5).	Adoption of Wearable Technology for the Elderly				Specifically, the Family Value is considered as an insignificant factor for technology readiness. In addition, high modification Indices between other factors are shown. The relation of Family Value to other factors were further studied. The state of art shows that the family is important in making a decision related to health for the elderly [17]. Moreover, the family members' perspective is concerned with the improvement of the elderly's quality of life [15]. The informal caregiver, to a certain extent, makes a decision about new technology for their elderly [19]. As a result, the family value is considered to be a supporting factor for interest and passion, the value of money, and health value factor.				Technology readiness, and behavior that related to character of wearable as shown in Fig. 5. Some items were removed from the model as they yielded low factor loading or deemed redundant to other factor. The structural equation model had a reasonable fit to the data: CMIN/DF= 2.164, GFI= 0.692, RMSEA= 0.091, and CFI0.829.From the results of H1a (β=0.063, p<0.001) and H1b (β=0.091, p<0.05), we speculated that Interest and Passion would positively affect the positive and negative side of technology readiness. The indicates that both sides are significant.<br><br>The results of H2a (β=0.102, p<0.001) and H2b (β=0.146, p<0.05) show that Value of Money would positively affect the positive and negative side of technology readiness, which indicates that both sides are significant.<br><br>H3a (β=0.111, p<0.001) and H3b (β=0.150 n.s.) yield the results which signify that Health Value would positively affect the positive side of technology readiness and negatively affect the negative side of technology readiness. It shows that Health Value is the significant predictor of the positive side of technology readiness but insignificant to the negative side.<br><br>The value of H4a (β=0.114, p<0.001), H4b (β=0.093, p<0.001) and H4c (β=0.111, p<0.001) infer that Family Value would positively affect				UNDECLARED
Exploring user needs for a mobile behavioral-sensing technology for depression management: Qualitative study	2018	Journal of Medical Internet Research	Meng, J. and Ali Hussain, S. and Mohr, D.C. and Czerwinski, M. and Zhang, M.	10.2196/10139	The main objectives of this study are to (1) understand the usefulness of a mobile sensing platform (ie, iSee) in improving counseling services and assisting students?self-management of their depression conditions, and (2) explore what types of behavioral targets (ie, meaningful information extracted from raw sensor data) and feedback to deliver from both clinician and students' perspectives.	Qualitative	USA	people with depression	For the 9 clinicians (aged 31-55 years; mean 42 years, SD 5.83; 8 female), 3 were clinical psychologists, 3 were clinical counselors, 2 were educational psychologists, and 1 was a clinical social worker. For the 12 student participants (aged 19-22 years; mean 21, SD 1.22; 7 female), 5 were mildly depressed with PHQ-9 (PHQ-9 is a diagnostic assessment of major depression disorder) scores ranging from 9 to 14, and 7 were moderately depressed with PHQ-9 scores ranging from 15-19. Out of the 12 student participants, 10 were undergoing treatment (ie, antidepressants) with the student health center and all of them were receiving psychological counseling at the MSU counseling center or counseling service outside the MSU campus.	a Mobile Behavioral-Sensing Technology for Depression Management								Usefulness: Support Continued Self-tracking Nine out of the 12 student participants interviewed mentioned that they had used some kind of mobile tracking apps but unfortunately, it was difficult to continue the self-tracking practice. The discontinuance of self-tracking is mainly due to the amount of effort and time spent on inputting one's data.Barriers From a Student Perspective Three out of the 12 student participants expressed their concerns about not being able to check on their behavioral targets on a regular basis. Student 7 stated:		Need for Data on Integrated Behavioral Targets Seven out of the 12 student participants pointed out that they found data that could explain the correlations between various behavioral targets and their depression would be helpful to increase self-awareness.	[illegible] Feedback Four student participants tried to avoid seeing negative feedback because of the increased feeling of helplessness.<br><br>In contrast, 6 student participants expressed their love and need for positive supportive messages.<br><br>During the interview, we prompted students with questions about privacy as a potential barrier of using iSee. Nine student participants did not feel privacy was an issue as long as their data were "anonymous." Eleven students were satisfied with the privacy issue when the researcher explained that iSee would deidentify personal data before transmitting to the cloud where the data would be stored. One student would like an option to choose what data to share:	N
Development of a Rehabilitation Monitoring System on Web-Based Application for Patients with Knee Pain	2018	iEECON 2018 - 6th International Electrical Engineering Congress	Tipprom, A. and Soontornpipit, P. and Sillabutra, J. and Satitvipawee, P. and Viwatwongkasem, C.	10.1109/IEECON.2018.8712291	This research aimed to design and develop the system that can provide two-way communication for rehabilitation monitoring between physical therapists and patients with knee pain.	Qualitative	Thailand	Patients with Knee Pain		Development of a Rehabilitation Monitoring System on Web-Based Application for Patients with Knee Pain			Both physical therapists and patients had positive attitudes toward the rehabilitation monitoring system.		However, some physical therapists and patients found that it was inconvenient using web application on mobile phone.		They suggested developing the system in native mobile applications such as iOS and Android.			[illegible] communicate with each other easily. Physical therapists mentioned that this system was useful for reminding them about prescribed exercise that they assigned to each patient. Patients reported that this system help them to recall exercise instructions and to follow the exercise protocol better. Moreover, all patients were happy with the monitoring function as they can recheck their recovery progress. Both physical therapists and patients agreed that this system did not add more tasks or having complicated functions because they often used web application and internet.		UNDECLARED
Opinions on the use of technology to improve tablet taking in >65-year-old patients on cardiovascular medications	2018	Journal of International Medical Research	Holender, A. and Sutton, S. and De Simoni, A.	10.1177/0300060518770578	This study was performed to evaluate the perceptions of the use of technology to improve cardiovascular medicine taking among patients aged >65 years.	Qualitative	UK	older patients on cardiovascular medications	8F, 4M; age: 65-76	Opinions on the use of technology to improve tablet taking in >65-year-old patients on cardiovascular medications	Participants in the first focus group were accustomed to receiving phone reminders about healthcare appointments, had an understanding of mobile technology, and welcomed the idea of interventions through text messaging or phone alerts/reminders.Participants in the second focus group were not familiar with smartphones, but all used wristwatches and preferred interventions using this technology, such as smartwatches.	Participants in both focus groups felt that those least familiar with technology would be the older, more vulnerable part of the population. Interestingly, they were also felt to be those who in theory would benefit most from the technology, thus negating its benefit.Nevertheless, the participants recognised that over time this will become less of a problem as the population as a whole becomes more familiar with technology.						Presence of technology in everyday life Individuals' beliefs regarding necessity determine whether they feel technology is necessary for improving their medication taking.Importance attributed to taking medication Most participants recognised that considering medication taking to be important would strengthen their beliefs regarding the necessity of the use of technology.The participants also acknowledged that some people intentionally fail to take their tablets because of motivation issues. The participants felt that technology is unlikely to help improve tablet taking in these circumstances and that these patients will likely not consider technology for their medication taking.Participants in the first focus group discussed the issue of elderly people with significant physical and cognitive impairments who no longer wish to live and believe that medications are no longer needed. Although in theory they may be helped with tablet taking by technology, they might not wish to engage with it. In these cases, technology cannot be forced upon them.		Technology as memory aid One of the major perceived benefits of using technology to improve tablet taking was its use as a memory aid. Participants recognised that forgetting to take medications was a significant problem that would only increase with age.<br><br>Technology can improve monitoring by care team The storage of patient data through technology offers healthcare teams and carers the opportunity of being able to access patients' records of medication taking and therefore better monitor the patients' medicine taking.<br><br>Technology and the use of resources Participants showed awareness of the scarcity of resources in healthcare, both in terms of the economic costs of technology itself and its implementation, and the time that healthcare professionals spent monitoring collected data. Elderly people constitute a small proportion of smartphone owners, although they represent the population who would benefit most from this technology in terms of help with tablet taking. Making it accessible to them was felt to	Concerns<br>Technology and patient–professional communication A concern voiced by participants was that distance monitoring of tablet taking through technology can reduce the frequency of healthcare professionals' visits to patients or that their interactions may become somehow impersonal. Patients could be at risk of receiving less face-to-face care, in turn making the elderly more vulnerable.<br><br>Technology and patients' accountability to the care team and to self Participants felt that if they knew they were being monitored, they would be more accountable for their tablet taking and that this would encourage them to take their medication. Furthermore, greater involvement of the care team or their family support network would contribute to holding them accountable.<br><br>At the same time, however, the participants acknowledged the risk that the technology would delegate the responsibility of tablet	N
Wearable technology in epilepsy: The views of patients, caregivers, and healthcare professionals	2018	Epilepsy and Behavior	Bruno, E. and Simblett, S. and Lang, A. and Biondi, A. and Odoi, C. and Schulze-Bonhage, A. and Wykes, T. and Richardson, M.P.	10.1016/j.yebeh.2018.05.044	We were interested in their current use of digital technology as well as their willingness to use wearables to monitor seizures. We also explored the role of factors influencing engagement with technology, including demographic and clinical characteristics, data confidentiality, need for technical support, and concerns about strain or increased workload.	Quantitative	UK	patients with epilepsy, caregivers	haracteristics PWE<br>(N, %) Caregivers<br>(N, %) Healthcare professionals<br>(N, %)<br>Number 52 13 22<br>Age group<br>Under 20 2 (3.8) 0 (0.0) 0 (0.0)<br>20–29 9 (17.3) 2 (15.3) 2 (9.1)<br>30–39 25 (48.1) 4 (30.8) 10 (45.4)<br>40–49 5 (9.6) 4 (30.8) 4 (18.2)<br>50–59 11 (21.2) 3 (23.1) 5 (22.7)<br>60–69 0 (0.0) 0 (0.0) 1 (4.6)<br>Gender<br>Female 43 (82.7) 12 (92.3) 14 (63.6)<br>Employment<br>Full-time 20 (38.5) 6 (46.1) 21 (95.5)<br>Part-time 10 (19.2) 4 (30.8) 0 (0.0)<br>Unemployed 20 (38.5) 3 (23.1) 1 (4.5)<br>Retired 2 (3.8) 0 (0.0) 0 (0.0)	Wearable technology in epilepsy: The views of patients, caregivers, and healthcare professionals			The majority of PWE (80.0%) were willing to use a wearable device for seizure tracking. When asked what type of device would be acceptable for seizure recording/detection in the medium term (weeks) and long term (years), the majority (> 69.0%) in each group indicated either a smartphone or a smartwatch (Fig. 4). A plain wristband (with no watch face), a ring-styled wearable, and a sensor under the mattress were additional options for PWE and HP. Headbands and small EEG electrodes were less favored:	For PWE, age, gender, epilepsy duration, and seizure type and frequency did not influence the willingness to use any of the device options provided (p > 0.05).			3.6. Designing acceptable technology<br>3.6.1. Measurement parameters<br>When provided with a list of parameters they would consider more useful to help track seizures in the long term (Fig. 5), all groups selected body movements, HR, breathing rate, and sleep quality. The majority of PWE favored multiple parameters if measured simultaneously through the same device:<br><br>Less preferable options were location monitors such as global positioning system (GPS), how many times the smartphone is used, and voice quality. Caregivers appeared to be less in favor of mood, concentration, attention, and memory while HP were less interested in monitoring skin temperature and environmental factors than PWE. Further desirable features for PWE were stress levels, medication taken, fluid intake, alcohol drinking, and oxygen saturation while HP suggested mental state, emotion, stress, blood sugar, and observations made by family and friends.<br><br>One of the participants in the PWE group expressed the need to extend seizure detection to different seizure types:<br><br>3.6.2. Data accessibility and use<br>Having the information constantly sent to a computer was preferred by PWE (55.8%) and CG (53.8%) rather than the option of having the	3.5.1.1. Correlations between confidentiality and the willingness to use wearable devices for seizure tracking<br>For both PWE and CG, age (p = 0.3), gender (p = 0.3), and current use of digital technology (p = 0.4) did not impact on concerns about data confidentiality. Moreover, for PWE, concerns about confidentiality did not influence their willingness to use wearable devices (p = 0.7).<br><br>3.5.2.1. Correlations between need of technical support and the willingness to use wearable devices for seizure tracking<br>For both PWE and CG, age (p = 0.1), gender (p = 0.2), and current use of digital technology (p = 0.1) did not influence the need for technical support. Of note, PWE currently using a wearable device reported less need for support although this trend was not statistically significant (p = 0.09). The need for technical support did not influence PWE's willingness to use wearable devices (p = 0.7).<br>When specifically asked about the use of wearable devices, thirty (57.7%) PWE did not own any wearable. The reasons were mainly uncertainty about their usefulness (21.1%), device cost (9.6%), availability (7.7%), appearance (3.8%), and burden/comfort (1.9%). Nevertheless, 19/30 (63.3%) were interested in wearables and had been thinking about purchasing one. Of the 22 (42.3%) PWE who already owned one, 20 (38.5%) were using it regularly. These wearables were mainly used for exercise tracking and general health monitoring, but there was a range of other uses (see Fig. 2). Among the device features		When asked to rank the greatest benefit of having seizures recorded, PWE prioritized:<br>having the information for their own knowledge (59.6%)<br>being able to share the information with their doctors (48.1%)<br>having a device that alerts relatives when you have a seizure (36.5%)<br>Less preferable options were the following: having a device that can alert medical services (26.9%), having the information available to discuss with your employer (19.2%), and having the information available to discuss with welfare benefits services (15.4%):	3.5. Concerns about using digital technology and wearable devices<br>3.5.1. Confidentiality<br>When asked about information and data confidentiality, 31 PWE (59.6%) reported concerns about the information not being confidential with nearly half stating specific concerns about privacy, a third for the lack of control over the information, and just one-quarter because of potential negative effects on their work or their ability to drive. Only four CG (30.8%) reported concerns about the information not being confidential, three about the lack of control over the information, and one about potential negative effect on their work.<br><br>3.5.2. Need for technical support<br>Only a small proportion of PWE (28.8%) considered technical support unnecessary. People with epilepsy (48.0%) and CG (53.8%) preferred technical support to be provided in a written document or video tutorial while HP favored person-to-person	N
Young people's perspectives on and experiences of health-related social media, apps, and wearable health devices	2018	Social Sciences	Goodyear, V.A. and Armour, K.M.	10.3390/socsci7080137	This paper synthesizes findings from three separate projects that investigated over 1600 young people's (age 13-19) perspectives on and experiences of health-related social media, apps, and wearable health devices.	Quantitative	UK	Young people	Midlands and the South East and North West of England. Of the 12 schools, 3 were private, 4 were state schools, and 5 were academies, of which 2 were faith schools. The schools were located in diverse socio-economic contexts and included students from a range of ethnic backgrounds.	health-related social media, apps, and wearable health devices										Many young people experience a range of positive benefits for their physical activity, diet/nutritional, and body image related behaviors.	Yet there are a number of risks, and young people report on the power of digital health technologies to shape, influence, and change their health-related behaviors.	N
Diffusion of the digital health self-tracking movement in Canada: Results of a national survey	2018	Journal of Medical Internet Research	Pare, G. and Leaver, C. and Bourget, C.	10.2196/jmir.9388	The aim of this study was to investigate the diffusion of the digital self-tracking movement in Canada. It provides a comprehensive, yet detailed account of this phenomenon. It examines the profile of digital self-trackers, traditional self-trackers, and nontrackers, further investigating the primary motivations for self-tracking and reasons for nontracking; barriers to adoption of connected care technologies; users' appreciation of their self-tracking devices, including what they perceive to be the main benefits; factors that influence people's intention to continue using connected care technologies in the future; and the reasons for usage discontinuance.	Quantitative	Canada	General public	The sample was composed of 2118 men, representing 51.55%. In terms of age, 27.84% of all respondents (1144/4109) were millennials (18-34 years), whereas 35.17% (1445/4109) consisted of baby boomers (55+ years). As expected, the majority of respondents were from the two largest Canadian provinces, namely, Ontario (1575/4109, 38.33%) and Quebec (986/4109, 24.00%). About 1 out of 5 respondents had a gross family annual income of less than $40,000 CAD, whereas 35.58% (1462/4109) had annual family incomes superior to $80,000 CAD. Our survey participants were more educated than the Canadian population according to data from the 2016 national census. Almost half of the respondents had a university degree compared with 28.70% for the whole population, 6 out of 10 respondents were workers (2386/4109), less than 4% (3.68%, 151/4109) were students, and slightly over 23% (22.8%, 937/4109)	Digital Health Self-Tracking Movement in Canada	At the time of our survey, 74% of respondents (or 86% of those with a mobile phone or tablet) had already heard of consumer health wearables and smart medical devices. However, the level of familiarity with these remains relatively low, as only 16% of respondents who had already heard of such devices also reported being "very or extremely" familiar with them.	We found that the level of familiarity with these tools is negatively correlated with age (r=-.21; P<.001) and positively correlated with family income (r=.14; P<.001). Indeed, millennials (χ24=50.0; P<.001) and people with annual family income over $80,000 CAD (χ24=28.2; P=.03) were more likely to be familiar with digital self-tracking devices than the other groups.	Overall, respondents perceive these devices as highly useful. About 7 out of 10 users (398/580) As shown in Figure 1, our model suggests that an individual's intention to continue using health wearables and smart devices is mainly influenced by his or her level of satisfaction. In turn, user satisfaction is influenced by the extent to which initial expectations toward these devices are confirmed, as well as by two factors from the Technology Acceptance Model (TAM) proposed by Davis [19], namely, ease of use and perceived usefulness.	The second group, traditional self-trackers represents 25.58% (1051/1669) of our sample and 38.64% of all self-trackers (1051/2720). Unlike digital self-trackers, this group comprises slightly more women than men. The majority of traditional trackers are aged 55 years and older, retired, with a gross family income substantially less than digital trackers. Importantly, individuals in this group are more likely to be living with one or several chronic diseases than digital self-trackers (47.07% vs 28.36%, respectively).<br><br>The third group, nontrackers, represents 33.80% of our sample (1389/4109). Like traditional trackers, this group comprises slightly less men than women. However, nontrackers are found in all age groups. They are less educated and have lower gross family income on average than the other two groups. One in four nontrackers reported having one or several chronic conditions. The most common reasons given by these respondents for not tracking any aspect of their health were as follows: "the information provided by my physician is sufficient" (335/1389, 24.12%), "no	easy to use	The use of digital self-tracking devices and mobile apps is mainly the result of motivations tied to sustaining individual well-being rather than monitoring or mediating medical problems or illnesses. More precisely, 57.94% of our respondents (967/1669) said they use connected care technologies mainly to know more about their condition and monitor changes in parameters that they consider important for their health. Another common motivation was associated with the day-to-day encouragement that digital health self-tracking technologies provide as people strive to meet their personal goals (883/1669, 52.91%). Importantly, 42.06% (702/1669) said they use digital self-tracking tools to monitor their progress in fitness or athletic training. For their part, motivations such as "follow the treatment plan prescribed by my physician" (447/1669, 26.78%), "improve communication with my physician" (400/1669, 23.97%), and "reduce the number of medical visits" (381/1669, 22.83%) were not the primary drivers of use. Quite conversely, traditional self-trackers tend to monitor specific clinical parameters related to chronic conditions such as weight, heart rate, glucose level, and medication		We observed a slightly reduced observation of this phenomenon, with 25.54% of owners (259/1014) who had stopped using their connected devices at the time of the survey. When asked "Why did you stop using your device(s)?" a majority of respondents (111/259, 42.9%) said they had "lost interest after a while." Other reasons included malfunctioning of the device (51/259, 19.7%), doubts about the reliability of the data (39/259; 15.1%), and "the device was acquired more out of curiosity" (38/259, 14.7%). Most interestingly, we found that while abandoning use of these devices was not associated with gender, age, region, education, or main occupation, it was more prevalent among those who perceive there current health status as "poor or fair" compared with those who self-report their health status as good or excellent (χ24=6.6; P=.048).Our results indicate that 46.93% of this segment (955/2035) did not see the interest in owning such tools. Other obstacles to greater diffusion of digital self-tracking devices were related to cost (836/2035, 41.08%), lack of knowledge about the value or benefits associated with the use of these devices (368/2035, 18.08%), and doubts about the reliability of data (341/2035, 16.76%). As many nonowners have limited knowledge of the value proposition for such devices, it is not surprising to observe that intentions to buy and adopt one in the near future were relatively low. Indeed, slightly less than 15% of nonowners (14.99%, 305/2035) reported that they intend to acquire a health wearable or smart medical device in the next 12 months.		Overall, respondents perceive these devices as highly useful. About 7 out of 10 users (398/580) said that they have maintained or improved their health status by using digital self-tracking connected devices. Importantly, a majority of users report they are more informed or more knowledgeable about their health condition. Close to 6 out of 10 users (435/580) said they feel more confident taking care of their health or more autonomous in the management of their condition. On the other hand, feeling less anxious about one's own health and having more informed discussions with a doctor were not perceived as major benefits digital self-trackers in our study.	This study indicates that there are relatively few people who regularly share the data captured with their digital self-tracking devices. In fact, only 34.87% of users (582/1669) reported that they share their personal health data. When they do so, it is primarily with family members (352/582, 60.5%), friends (294/582, 50.5%), and to a much lesser extent, an HCP such as a family doctor (195/582, 33.5%) or a pharmacist (50/582, 8.6%).	N
Adolescent and caregivers?experiences of electronic adherence assessment in paediatric problematic severe asthma	2018	Journal of Child Health Care	Stewart, A.C. and Gannon, K.N. and Beresford, F. and Fleming, L.	10.1177/1367493517753082	This study explored the experiences of adolescents and their caregivers regarding adherence to inhaled corticosteroids which are assessed through an electronic monitoring device (EMD).	Qualitative	UK	Adolescent in paediatric problematic severe asthma and caregivers	Eleven eligible adolescents and their caregivers attended the clinic during the recruitment period, of whom eight consented to participate. Five adolescents were female and three were male, ranging in age from 11 to 15 (mean = 12.86, SD = 1.57). Caregivers were female and mothers of the participating child. The participants were 75% White British and 25% Asian British.	electronic adherence assessment in paediatric problematic severe asthma										For several of the participants, the EMD was seen as an extension of this preventative treatment, viewed as something that had been introduced to help the adolescents 'get better', reduce the need for hospitalization or having to use their reliever inhalers.	In some cases, this raised issues of mistrust and fear in the healthcare relationship, promoting a sense of surveillance of young people and caregiver.<br>Similar to their caregivers, the monitoring process also contributed to a sense of mistrust in the relationship between the adolescent and healthcare professional, with some of the adolescents predicting that the information recorded could land them in trouble, with limited opportunities to explain their side of the story.	N
Informing evaluation of a smartphone application for people with acquired brain injury: a stakeholder engagement study	2018	BMC Medical Informatics and Decision Making	Kettlewell, J. and Phillips, J. and Radford, K. and Dasnair, R.	10.1186/s12911-018-0611-0	The aim of this paper is to describe the process of exploring the barriers and enablers for the uptake and use of Brain in Hand in clinical practice, identify potential adaptations of the app for use with people with acquired brain injury (ABI), and determine whether the behaviour change wheel can be used as a model for engagement.	Quantitative	UK	ABI survivors and carers	Total = 50 Age Gender<br>18–30 31–50 51+ Male Female<br>ABI survivors (n = 20) n 3 9 8 15 5<br>% total 6% 18% 16% 30% 10%<br>Carers (n = 5) n 1 0 4 2 3<br>% total 2% 0% 8% 4% 6%<br>Professionals (n = 25) n 2 12 11 4 21<br>% total 4% 24% 22% 8% 42%<br>Overall n 6 21 23 21 29<br>% total 12% 42% 46% 42% 58%	a smartphone application for people with acquired brain injury:					Capability<br>Participants felt they would be confident using the app if they were existing technology users (22/24, 95%), but only a quarter of people felt it was completely appropriate for the ABI population (6/25, 24%).	Most people said they would be motivated if they could see a use for BiH (39/41, 95%); and after physically seeing the app, 65% (17/26) of people said they wanted to use it (Table (Table3).3).<br>ABI survivors felt that using a mobile phone app as an assistive aid was viewed as 'normal' behaviour, i.e., reduced stigma, and would be a motivator for use (Table (Table2).2).	One interesting suggestion was to use the traffic light system to monitor positive behaviours, such as a feeling of wellbeing, rather than focusing on negative behaviours. All stakeholders identified the potential for personalisation to individual needs, one of the difficulties with existing technologies, as an important enabler to uptake. All stakeholders identified the need for voice recognition and the ability to verbally add things to the diary, along with having visual entries (e.g. adding pictures, diagrams and videos to diary entries, such as therapy exercises, making dinner, etc.). Professionals and carers suggested having an app specifically for mentor use, to monitor user activity without needing computer access. Professionals voiced concern over the use of the red button, the potential for it to be overused and who would be responsible for responding to it.	This finding is reflected by comments from professionals who felt that users needed an awareness of their problems and an ability to use strategies for the app to be effective (Table 2). Most stakeholders identified clear barriers during group discussions: visual and physical impairments, lack of technical skill, poor literacy skills, and cognitive problems faced by ABI survivors. Professionals identified potential problems associated with the use of BiH, such as difficulty switching between screens.The main barriers identified were the lack of interest in technology, not seeing a use for it and a lack of existing evidence of its use in ABI. ABI survivors were concerned about their privacy and a feeling of being monitored, and this was expressed as a potential barrier to uptake. Stakeholders felt that setting up the app may be time consuming and excessive reminders may be annoying.<br>Stakeholders highlighted the inconvenience of having to access a computer to edit the diary and functions of the app, which could be a potential barrier to uptake.<br><br>The groups identified a potential barrier to uptake being competition from apps that have similar functions, but are free or have a one-off cost. Technological problems were identified as major barriers, specifically by individuals who were worried about loss of phones, the battery running down, compatibility with other systems and loss of data following phone updates. There was a concern about use in the work place, as for many people it is not appropriate to use a phone whilst		The questionnaire identified that most people (39/41, 95%) felt BiH would be useful for memory problems, to help with activities of daily living (35/41, 85%) and to support independence (33/41, 80%), such as taking responsibility of appointment planning.		N
Patient attitudes towards remote continuous vital signs monitoring on general surgery wards: An interview study	2018	International Journal of Medical Informatics	Downey, C.L. and Brown, J.M. and Jayne, D.G. and Randell, R.	10.1016/j.ijmedinf.2018.03.014	This study aims to discover what patients think of monitoring in hospital, with a particular emphasis on intermittent early warning scores versus remote continuous monitoring, in order to inform future implementations of continuous monitoring technology.	Qualitative	UK	Patient	Six patients were male; 6 patients were female. Their ages ranged from 42 to 83 years. The number of days spent in hospital at the time of interview ranged from 5 to 27 days, and the number of days spent wearing the patch at the time of interview was between 1 and 15 days.	remote continuous vital signs monitoring on general surgery wards			Overall, patients reported positive experiences of vital signs observation rounds. Patients were keen to emphasise their appreciation of face-to-face nursing contact, and their concerns that remote monitoring might replace this.		Eight of the 12 patients mentioned their irritation at being woken up for observation rounds.<br><br>An important issue for patients was that of comfort. Most patients (10/12) found the patch so comfortable that they forgot they were wearing it.<br><br>Whilst they had no complaints about comfort, two patients expressed concerns about the practicalities of wearing the patch.					Many patients expressed that they saw remote monitoring having the most value for nursing staff. Patients were aware of how busy the nurses were and so could appreciate the benefit of the patch in terms of freeing up nurses' time.	A number of patients expressed reservations about the reliability of the technology. One patient expressed concerns about data security. Others were more worried about system failure.However, the most common reason for mistrusting the technology was the lack of feedback, especially if no notifications were sent by the device.	N
The burden of a remote trial in a nursing home setting: Qualitative study	2018	Journal of Medical Internet Research	Donnelly, S. and Reginatto, B. and Kearns, O. and Mc Carthy, M. and Byrom, B. and Muehlhausen, W. and Caulfield, B.	10.2196/jmir.9638	The aim of this study was to explore the burden associated with a remote trial in a nursing home setting on both staff and residents.	Qualitative	Ireland	older adults and nursing home staff	Characteristics of residents.<br><br>Characteristic Value<br>Age (years), mean (SD) 80 (10)<br>Gender, n (%)<br><br>Male 4 (36)<br><br>Female 7 (64)<br>Mobility<br><br>Had a fall in past 6 months, n (%) 3 (27)<br><br>Uses mobility aid, n (%) 4 (36)<br><br>Timed up and go (seconds at baseline), mean (SD) 28 (17)<br>MMSEa score, n (%)<br><br>30-25 (normal) 1 (9)<br><br>24-21 (mild or early) 4 (36)<br><br>20-10 (moderate) 2 (18)	mobile and wearable technologies											Among staff, we found that the following eight characteristics of burden emerged: (1) comprehension, (2) time, (3) communication, (4) emotional load, (5) cognitive load, (6) research engagement, (7) logistical burden, and (8) product accountability. The watch emerged as a consistent source of burden throughout the trial for HCAs who were responsible for charging it. Similarly, the Samsung mobile phone (hereafter referred to as the "phone") was a source of burden for SAs responsible for syncing it with the watch to support data transfer. Among residents, we found only four sources of burden: (1) comprehension, (2) adherence, (3) emotional load, and (4) personal space.	N
The role of individual differences on perceptions of wearable fitness device trust, [illegible]	[illegible]		Rupp, M.A. and Michaelis, [illegible]	[illegible]	We examined the impact of several user (i.e. personality, age, computer self-efficacy, physical activity level) and device characteristics (trust, usability, and motivational affordances) on the behavioral intentions to use a wearable fitness device.				One hundred and three participants, 48 males and 55 females, ranging in age from 18 to 83 years of age were recruited for this study (M = 36.51 years, SD = 23.40 years; Table 1). Participants were recruited from both the participant [illegible]					We found computer self-efficacy, physical activity level, as well as personality traits indirectly increased the desire to use a fitness device and influenced the saliency of perceived motivational affordances. Additionally, trust, usability, and perceived motivational affordances [illegible]								

Mobile multimedia health applications and their potential impact on the human population	2018	2018 IEEE Long Island Systems, Applications and Technology Conference, LISAT 2018	Ansari, A. and Gerais, E. and Taylor, G. and Tyrpien, B. and Elleithy, A.	10.1109/LISAT.2018.8378018	In this paper we have conducted a survey that included 10 questions and involved 91 survey participants where 85.4% own a smartphone. More than half, 56.7%, of the survey participants responded that they have previously used or are using a health application and 57.8% own a wearable device that possesses health related functionality. The survey revealed uncertainties concerning health related applications and wearable technology revealed.	Mixed	USA	General public		Mobile multimedia health applications	A nother possibility could be that too many of the people who took the survey are not properly or sufficiently informed concerning the potential and almost innumerable benefits that health applications can provide them.		The statistics that have been gathered indicate that while there would be benefits to this aspect of our proposal, there are still a considerable amount of people who still would not feel comfortable using health applications with these changes alone.								[illegible] who took the survey expressed that they were not aware of how to detect if an application was legitimate or not. While this still represents a large portion of the population who struggle with this issue, 73.6% of our participants indicate that they would be inclined and willing to use health applications more should this information become available in a more user-friendly and intuitive manner. In other words, for those who had issues with determining legitimate applications from fraudulent ones, a majority reported that they would be more likely to use health applications if this information could be found or was provided more easily. Additionally, three out of five participants who do not currently trust health applications agreed that they would if such changes were	UNDECLARED
User-based evaluation of applicability and usability of a wearable accelerometer device for detecting bilateral tonic束lonic seizures: A field study	2018	Epilepsia	Meritam, P. and Ryvlin, P. and Beniczky, S.	10.1111/epi.14051	Clinical validation studies of seizure detection devices conducted in epilepsy monitoring units (EMUs) can be biased by the artificial environment. We report a field (phase 4) study of a wearable accelerometer device (Epi-Care) that has previously been validated in EMUs for detecting bilateral tonic clonic seizures (BTCS).	Quantitative	New Zealand	patients with bilateral tonic-clonic seizures	[illegible] completed thestudy was 27 years (range=7-72 years); 39 were malesand 32 were females. 46% (n=33) of the patients wereliving at home, 44% (n=31) in an institution, and 10%(n=7) partly in both places. The questionnaire wasanswered by personnel in residential institutions in 45% ofcases (n=32), by a family member in 31%, and by thepatients themselves in 17%. In 7%, it was a combination ofpatient, personnel, and family member (n=3) or personneland family member (n=2). This reflects the profile of thepatients using the device: patients with severe epilepsy andBTCS	a wearable accelerometer device for detecting bilateral tonic-clonic seizures			Regarding satisfaction with using the system: 18% (n=13)of the responders strongly agreed, 32% (n=13) agreed,and 21% (n=15) predominantly agreed (Likert scale med-ian=5.5).		[illegible] (6-7)It is simple to use this device.7 (6-7)The device can effectively detect BTCS.6 (5-7)The rate of false alarms is acceptable.6 (4-7)The use of the device has resulted infewer injuries to the patient.4 (1-6)The device gives clear alarm signals.7 (6-7)The alarm time points from the deviceare clearly readable.7 (6-7)The device has an influence on howmany seizures are logged into theseizure diary.5 (1-6)The device gives error messages whentechnical problems occur.7 (6-7)The sensor (wristwatch) is comfortableto wear.5 (4-6)The connection between sensor anddisplay is functioning well.6 (5-7)There have not been any side effectsrelated to using the system.The reasons given for being dissatisfied were:high FAR, irritation or discomfort from wearing the wrist-watch, unstable connection between the sensor and the con-trol unit, low effectiveness for detecting seizures, shortbattery time, and the device not being water-resistant.In 18 of the 45 patients (40%) who previously had hadseizure-related injuries and who experienced seizures dur-ing the period of the study, the patients and the caregiversagreed that the use of the device had resulted in fewer inju-ries to the patient In 48 patients, BTCS occurred while the device was inuse.							N
Acceptability of mHealth augmentation of Collaborative Care: A mixed methods pilot study	2018	General Hospital Psychiatry	Bauer, A.M. and Iles-Shih, M. and Ghomi, R.H. and Rue, T. and Grover, T. and Kincler, N. and Miller, M. and Katon, W.J.	10.1016/j.genhosppsych.2017.11.010	To assess the feasibility and acceptability of a mobile health platform supporting Collaborative Care.	Mixed	USA	patient	Among the final sample of 17 participants, most were female (n = 10; 59%), white (n = 16; 94%), and had received Collaborative Care services for > 180 days (n = 11; 65%; see Table 2).	a smartphone app to transmit PHQ-9 and GAD-7 scores and sensor data to a dashboard	[illegible] value the mobile platform offered to her and the patients she cared for. In contrast, patients expressed varying levels of understanding of the app's purpose. They had a general understanding that the app collected self-report and passive data and that this information was shared with the clinician. However, most patients could not accurately recall specific information about the type of passive data collected. One patient noted that it was unclear whether the app was intended to help people in crisis. Limited patient understanding did not translate into shorter duration of app use		Perceptions of the impact of the app varied. The majority of participants reported overall satisfaction with the app (n = 10/13; 77%) and thought the app was useful (n = 11/16; 69%). Nearly half of participants reported feeling more connected to their doctor (n = 6/13; 46%) or more confident in managing their mental health (n = 6/13; 46%). Only a few participants endorsed overtly negative views of the app, such as feeling embarrassed (n = 2/16; 13%), but many (n = 9/16; 56%) were neutral on whether the app kept their information private.		All participants who responded reported that the app was easy to use and the amount of time was reasonable (Table 3).	The care manager's role in reinforcing app use Patients' increased awareness of their mental health would not have been sufficient to sustain use for some patients in the absence of care manager involvement (Table 4). Many patients felt that the care manager's response to the data they submitted enhanced their care, although a few noted that the data was not well-integrated into their care. The care manager echoed patients' statements that the app enhanced the care she provides. Patients speculated about how the app may detract from care, although notably nobody indicated that these concerns were realized.	Patients wanted to customize the app to meet their individual needs, for example, by adjusting the timing of prompts, the types of symptoms they were reporting on, or the frequency or content of health tips. Some participants appreciated the badges and reinforcements they received when they completed their check-in surveys, whereas others felt patronized by the motivational language, again suggesting a need to tailor the language to individuals' tastes. Participants desired a more individualized app experience that included directly visualizing their own data, having tailored interventions based on their current states, i.e., just-in-time adaptive interventions, or annotating standardized scores with personal diary-style notes.			Benefits of daily monitoring Despite variable understanding of the app, patients believe that using the app to monitor symptoms caused them to become more mindful of their symptoms (Table 4). This increased awareness of mood allowed them to be more proactive in their coping, although this awareness was received by some with ambivalence. The care manager also felt that monitoring mood more frequently than once or twice a month was beneficial. The platform's alerts, generated mainly in response to daily surveys, were viewed favorably by the care manager in comparison to the existing registry system that identifies people who have not improved after 10 weeks of treatment.	Privacy, confidentiality, and security Patients felt the data they submitted was not entirely secure (Table 4). However, they did not believe that the information reported in the app was too personal and therefore the potential for a data breach was not a major concern. Patients were comfortable sharing information about their mental health symptoms through the app, and the care manager's access to their information was frequently cited as promoting this comfort. Some patients, however, wished to have a better understanding about who else had access to their health information, as well as the ability to control such access. Potential burdens included time and the cognitive demands of filtering patient-reported data to identify the information she needed.	Ms. Kincler is employed by Ginger.io. Ms. Miller was employed by the University of Washington Neighborhood Clinics at the time the study was conducted. None of the other authors have any conflicts of interest to disclose.
Wearables in epilepsy and Parkinson's disease桝 focus group study	2018	Acta Neurologica Scandinavica	Ozanne, A. and Johansson, D. and H[illegible]lgren Graneheim, U. and Malmgren, K. and Bergquist, F. and Alt Murphy, M.	10.1111/ane.12798	The aim of this study was to explore perceptions regarding the use of wearable technology in disease monitoring and management as reported by individuals with epilepsy and Parkinson's disease as well as health professionals working with these patient groups.	Qualitative	Sweden	patients with epilepsy and Parkinson's disease	PwEn=10PwPDn=15HP- En=7HP-PDn=8Number of focus groups (n), with men/with women2/12/111Participant gender (n), men/women6/49/61/60/8Age (median, range)36, 22- 5666, 48- 8044, 35- 5647.5, 26- 64Age at diagnosis (median)16.5, 2- 3860, 34-76NANAIndividuals with experience of wearing sensors (n), yes1915Clinical professionNANAPhysician22Nurse21Physiotherapist02Occupational therapist12Psychologist10Biomedical scientist01Enrolled nurse10PwE, persons with epilepsy; PwPD, persons with Parkinson's disease; HP- E, health professionals working with epilepsy; HP- PD, health professionals working with Parkinson's disease.TABLE 1 Demographics and other characteristics of the participants	Wearables in epilepsy and Parkinson's disease						PwPD also thought that motivation for using wearables would in-crease with more severe disability. Men wondered how the integrity will be ensured at night time and whether the data will be sent to a central where they could be observed wherever they are. They saw similarities to an electronic ankle bracelet and were worried about people's reactions.	3.2.3 | Worrying about inconclusive recordingThe participants were concerned about recordings with insufficient and invalid information. They were concerned about whether the se-lected placement on the body was adequate for purpose and whether enough variables were measured. HP feared that disagreement be-tween sensor data and patients' own perceptions could lead to disap-pointment and mistrust.PwE and PwPD thought that recordings should include measures such as blood pressure, breathing patterns, temperature, and both arm and leg movements. They trusted themselves more than they would trust the recordings. They wondered if wearables will affect pacemak-ers, cause headache, generate radiation, or if mobile phones or other electromagnetic waves will affect the recordings.PwE worried that non- convulsive seizures with impaired con-sciousness would not be registered. It was considered difficult to de-termine whether movements and high pulse were caused by a seizure or by training activity. They also worried that extra hospital visits would be required for adjustment of the sensors. HP- E thought that it will be difficult to separate different seizure types from each other and from voluntary movements and that many seizures may not be registered at all.HP- PD thought that patients' own experiences are more important than the recordings. They thought that patients will be more active as they know they will be monitored.3.3 | Facilitators of usability3.3.1 | Design that simplifiesThe participants thought that attractive design (eg, sports top) would simplify use. It was			The participants thought monitoring may facilitate diagnostics and treatment, be cost- effective, and decrease the number of hospital visits. The patients considered that benefits gained from registration outweigh the possible inconvenience.PwE and HP-E perceived potential benefits of long- term moni-toring since that may provide information on type and frequency of seizures. HP- E thought convulsive seizures may be separated from other types of seizures (eg, non- epileptic seizures). Wearables may provide information about treatment efficacy. PwE thought that a combined use of registration and personal narratives would increase awareness of seizures. Both patients and HP-E thought that a wear-able that could warn about upcoming or ongoing seizures would be of great value.PwPD and HP- PD perceived that monitoring may provide a bet-ter and more truthful overall picture about symptom fluctuations and functioning. Wearables might provide more objective and correct in-formation about movement patterns throughout day and night com-pared to patients' narratives. HP- PD described that information from wearables might be useful when patients find it	Perceiving unclear informationThe participants thought that unclear information might generate unnecessary questions, uncertainty, and paranoia. PwE and PwPD thought that the sensors should be provided by HP; they did not trust private commercial companies.To avoid unclear information, the HP suggested that patients need to be well informed and understand what kind of information is gath-ered, to how long time and how the system works. Patients need to have realistic expectations and feel that recording is worthwhile.HP- E thought that the purpose needs to be connected to treat-ment needs (eg, recording of convulsive seizures may be more rel-evant in order to save lives). The recording should be initiated by a relevant health professional depending on the purpose. HP- PD wor-ried about possible situations where the patients could regulate their own medication based on the results from commercially available wearables.3.2.2 | Fearing lack of integrityThe	Dr. Bergquist reports personal fees, non- financial support, and other from Global Kinetics Inc., Australia. Dr. Ozanne, MD Johansson, Prof. Graneheim, Prof. Malmgren, and Dr. Alt Murphy report no conflict of interests with regard to the present stud
Activity monitors as support for older persons?physical activity in daily life: Qualitative study of the users?experiences	2018	JMIR mHealth and uHealth	Ehn, M. and Eriksson, L.C. and [illegible]erberg, N. and Johansson, A.-C.	10.2196/mhealth.8345	The aim of this study was to investigate how seniors experience using activity monitors (AMs) as support for PA in daily life. The addressed research questions are as follows: (1) What are the overall experiences of senior persons, of different age and balance function, in using wearable AMs in daily life?; (2) Which aspects did the users perceive relevant to make the measurements as meaningful and useful in the long-term perspective?; and (3) What needs and requirements did the users perceive as more relevant for the activity monitors to be useful in a long-term perspective?.	Qualitative	Sweden	Older adults	Participant characteristic Total, n (%)<br>Age (years)<br>75-80 3<br>80-85 3<br>85-90 2<br>Gender<br>Female 6<br>Male 2	Activity Monitors as Support for Older Persons' Physical Activity in Daily Life					To be useful, the devices must be easy to handle. In this study, the users perceived wearables as easy to handle, whereas tablets were perceived difficult to maneuver. Users reported in the diaries that the devices had been functional 78% (58/74) of the total test days.					[illegible] their awareness of own PA had increased. However, the AMs' impact on the users' motivation for PA and activity behavior varied between participants. The diaries showed that self-estimated physical effort varied between participants and varied for each individual over time. Additionally, participants reported different types of accomplished activities; talking walks was most frequently reported. To be meaningful, measurements need to provide the user with a reliable receipt of whether his or her current activity behavior is sufficient for reaching an activity goal. Moreover, praise when reaching a goal was described as motivating feedback.		N
Stroke Patients' Acceptance of a Smart Garment for Supporting Upper Extremity Rehabilitation	2018	IEEE Journal of Translational Engineering in Health and Medicine	Wang, Q. and Timmermans, A. and Chen, W. and Jia, J. and Ding, L. and Xiong, L. and Rong, J. and Markopoulos, P.	10.1109/JTEHM.2018.2853549	The objective is to evaluate to which extent that Zishi a garment equipped with sensors that can support posture monitoring can be used in upper extremity rehabilitation training of stroke patients.	Quantitative	China	stoke patients	17 subjects after stroke were recruited from 3 rehabilitation centers: Huashan Hospital, the Tianshan Hospital, and First Rehabilitation Hospital in Shanghai (China). Their mean age was 55 years (equation M2). However, patients from the First Rehabilitation Hospital were only available to participate in the experiment between their regular training sessions, therefore their questionnaires are only finished in part. The number of involved subjects for each questionnaire is listed in Table 1 together with the results of group Median and one-sample Wilcoxon signed rank test.	a Smart Garment for Supporting Upper Extremity Rehabilitation			However, subjects reported a high 'self-efficacy' score (MDN = 5.5, IQR = 1.5) and an acceptable score for 'attitude towards technology' (MDN = 4.8, IQR = 1.1).		Seven subjects completed the CSUQ questionnaire (results see Fig.9). The system was considered of good usability as the system overall satisfaction score was rated positively (MDN = 5.11, IQR = 0.95). The information on the screen was also perceived to be really easy to understand as subjects rated it very high for the questions addressing the information presented (MDN = 5.83, IQR = 1.67). System usefulness and interface design was rated above the sufficiency threshold (4 over 7).The 'perceived competence' subscale obtained a high score (MDN = 5.6, IQR = 1), which suggests that patients considered themselves competent to use the system.	All 17 subjects filled in the IMI questionnaire. The results (see Fig.7) on the 'interest/enjoyment' subscale indicates that the subjects were positively (MDN = 5.14, IQR = 1.64) motivated to use the system during rehabilitation exercises. The subscale 'value' is scored moderate (MDN = 4.67, IQR = 1.29) suggest that patients thought training with Zishi was moderately important. Subscale 'effort/important' obtained a good score indicates they were willing to put effort. The subscale 'pressure' is scored low and indicates that patients were executing the tasks without pressure.						UNDECLARED
Continuous temperature-monitoring socks for home use in patients with diabetes: Observational study	2018	Journal of Medical Internet Research	Reyzelman, A.M. and Koelewyn, K. and Murphy, M. and Shen, X. and Yu, E. and Pillai, R. and Fu, J. and Scholten, H.J. and Ma, R.	10.2196/12460	A pilot study was conducted to assess the accuracy of sensors used in daily wear socks, obtain user feedback on how comfortable sensor-embedded socks were for home use, and examine whether observed temperatures correlated with clinical observations.	Quantitative	USA	Patients With Diabetes	https://www.ncbi.nlm.nih.gov/pmc/articles/PMC6315272/table/table2/	Continuous Temperature-Monitoring Socks for Home Use in Patients With Diabetes			In these patients' experience while wearing the socks at home, they found the socks to be safe, comfortable, useful, and well-designed.		The mobile app was found to be useful and easy to use. The mean and the median responses to key statements on the mobile app are shown in Table 3. Users found it easy to navigate the mobile app and they found the information provided to be informative about their feet.							RM, HJS, XS, EY, JF, and MM are employees and shareholders of Siren. RP is a consultant to Siren. AMR is an advisor to Siren and shareholder of Siren. The study was sponsored by Siren.
Self-tracking for fertility care: Collaborative support for a highly-personalized problem	2017	Proceedings of the ACM on Human-Computer Interaction	Figueiredo, M.C. and Caldeira, C. and Reynolds, T.L. and Victory, S. and Zheng, K. and Chen, Y.	10.1145/3134671	Infertility is a global health concern that affects countless couples trying to conceive a child. Effective fertility treatment requires continuous monitoring of a wide range of health indicators through self-tracking. The objective was to understand the process in which women engage in tracking relevant information, and the challenges they face.	Qualitative	USA	women	two researchers independently coded the 100 most recent posts and their 377 replies using an open coding technique to identify the main topics present in the data	fertility self-tracking.						methods and we found that many women adopt it without medical supervision for several reasons, including (a) to understand their cycles, (b) to see how their bodies respond to treatments, (c) to communicate with providers more effectively, and (d) to try a more affordable option before resorting to more complex treatments. The following excerpts exemplify some of these reasons.						UNDECLARED
Views of individuals with spinal cord injury on the use of wearable cameras to monitor upper limb function in the home and community	2017	Journal of Spinal Cord Medicine	Likitlersuang, J. and Sumitro, E.R. and Theventhiran, P. and Kalsi-Ryan, S. and Zariffa, J.	10.1080/10790268.2017.1349856	Hand function impairment after cervical spinal cord injury (SCI) can significantly reduce independence. The objective of this study was to understand the views of individuals with SCI on the use of wearable cameras to track neurorehabilitation progress and outcomes in the community.	Quantitative	Canada	individuals with spinal cord injury	13M, 2F, age 34-63	Views of individuals with spinal cord injury on the use of wearable cameras to monitor upper limb function in the home and community			Participants expressed an interest in wearable technology (4.27 ± 1.10)		Participants expressed an interest in wearable technology (4.27 ± 1.10), but found the system only moderately comfortable (3.27 ± 1.44) and were not unanimous in their desire to use such technology (2.87 ± 1.36). everal participants expressed dissatisfaction with the comfort of the current setup (n= 5).		A participant also suggested that the camera be incorporated into glasses or wardrobe to improve the usability and quality of the output (n= 1).	Another individual expressed concern about using the system in public, and drew a contrast in this respect with other wearable technology such as the Fitbit. Participants explained that while they would not necessarily wear the system over a long period of time, they would be willing to use it periodically or over a fixed time period for a few hours to get feedback (n= 2). Similarly, in "other comments," participants also express concerns that wearing a camera felt "weird" even when not recording, coupled with the observation that monitoring for an hour or two at a time would be acceptable (n= 1).		They felt that the information would be most useful to researchers (4.73 ± 0.59), and highly relevant to clinicians (4.47 ± 0.83), though somewhat less to family members (3.73 ± 1.16). The participants also felt that getting access to this information for their own use would be beneficial (4.33 ± 0.90). In this category of questions, some participants' open-ended comments indicated that they saw value in technology that could help them track their own improvements (n= 3), or get feedback from clinicians or researchers (n= 2).	Participants expressed relatively low levels of concern about egocentric video being stored and used by clinicians (mean ± standard deviation of 1.93 ± 1.28), or by researchers (2.00 ± 1.31). Nonetheless, the participants expressed moderate concerns about being asked to wear a camera in their daily life (2.53 ± 1.51). In their open-ended comments, participants raised some concerns about recordings of bystanders (n= 3), washroom and personal care activities (n= 2), or the workplace and computer screens (n= 1). Conversely, participants also expressed trust that the information would be used appropriately (n= 1), saw benefits for research and teaching (n= 1), and stated that they would be willing to use the technology if it could help others with SCI (n= 1). One participant expressed some doubt about the value to clinicians because they felt that doctors' observations were generally different from their own perceived experience. Others thought that the information would be primarily useful to	UNDECLARED
Opportunities and challenges for self-monitoring technologies for healthy aging: An in-situ study	2017	Gerontechnology	Randriambelonoro, M. and Chen, Y. and Yuruten, O. and Pu, P.	10.4017/gt.2017.16.3.006.00	We present the findings of an experiment with 20 older adults using pervasive sensors for 6 weeks at their homes. In particular, we present their attitudes for physical activities and self-monitoring technologies before and after the actual usage. We then discuss the opportunities and challenges for designing self-monitoring technologies for healthy aging.	Qualitative	Switzerland	older adult	The participants include 13 females and 7 males; among them 3 couples took part in the study together. Their ages range between 65 and 89, with a mean age of 77. 6 participants live alone and the rest live with their spouses. Only a few of them still have occasional social activities. 8 of them have serious health conditions limiting them in doing physical activities. All of them have a TV and watch it or use it as background noise on purpose. Most of them use landline phones for communication. 8 of them have smartphones, and among them, four use social media applications (WhatsApp) motivated by their grandchildren; one of them uses a pedometer application introduced by her daughter and 1 uses a photo editing app. 9 participants have tablets and use them mainly for watching news, searching on internet and listening to music. 2 of them had experience using pedometers before the study but they stopped using it after a	self-monitoring technologies for healthy aging			Overall, 15 out of 18 participants expressed willingness to continue using the device, while 3 out of 18 participants did not plan to continue using it.			Participants were motivated to continue using the device because it was engaging, fun and allowed them to set personal goal and track their improvement. However, some of our participants (n=4) were eager to try new tools and devices.		However, some participants did not want to use the devices anymore because of perceived inutility, unwanted behavior related to the tools, and fear of the equipment negatively impacting their health. Another participant (P2) also returned the watch but kept the scale. She didn't like the fact that she wanted to look at the watch all the time to see how much she has done. One participant (P9) dropped the study at the end of the setup phase. Due to a health problem that occurred during the experience, she preferred to stop using the devices, fearing that there would be a link to that.The main barriers of being physically active were the absence of motivation, the incapacity due to health condition and the lack of perceived usefulness of physical exercise. Furthermore, although half of the participants were not technology oriented, personal interest and enthusiasm driven by family towards technology were observed.				UNDECLARED
Tune your sun right: Persuasive app towards healthy & safe sun exposure	2017	ACM International Conference Proceeding Series	Hussain, M.S. and Nicholson, B.D.-R. and Freyne, J.	10.1145/3152771.3156137	We use a commercial wearable UV/light sensor, visualizations, notification messages and educational content to inform a persuasive mobile application (app) to encourage selfmanagement of sun exposure. We report on a preliminary study with three participants investigating usability, user experience and perceived value of the technology solution.	Mixed	Australia	General public	[illegible] aged between 19-22 years (one male, two female) – one full-time office worker, and two university students. Participants used the Android-based app (on personal device) with a Microsoft Band 2 (supplied by investigators) for three days as part of their usual daily life. Following this, data was collected through face-to-face interviews (15 to 30 minutes), and participants completed a 10-item System Usability Scale (SUS) questionnaire [4]. Interviews consisted a total of eight questions that aimed to capture; personal experience using the app with the wearable device, perceived value of its features in terms of raising awareness and encouraging lifestyle change, and the overall perceived value of the technology	persuasive app towards healthy & safe sun exposure			Participants thought the app and its concept was interesting and could be very beneficial for people, particularly Australians.		The SUS scores were high among all three participants: 80.0, 77.5, and 87.5respectively. This indicates satisfaction and ease of use Short PapersOzCHI 2017, Nov 28 - Dec 1, Brisbane, Australia Human - Nature363across all three participants in regards to the app's user interface. The scores are relatively high in terms of SUS, with the average across 500 studies being a score of 68[25] Visualizations were found to be simple, accurate and useful; the dashboard/daily view was particularly useful. The simplicity of the visualization and the short summaries was found to be sufficiently informative while not over loading the user. Due to the short duration of the study, the historical view of UVI exposure was not found to be as useful, but one participant reported seeing meaning information.<br>Participants found the UVI notification (e.g. High) to be most useful.		Similarly, a participant suggested the app should contain information about required daily needs, and tracking of progress towards/beyond those needs.<br><br>Participants reported that they felt the notifications and messages they received were personalized, and informed on their behavior and the environment. Suggestions were made in terms of content and timeliness of delivery. The information about how long they had spent out in the sun was meaningful, for example one participant reported that this type of information could encourage change.			Participants found that the information presented by the app was useful in understanding their time out in the sun, which is something they were unable to track before.		UNDECLARED
Tracking down a solution: exploring the acceptability and value of wearable GPS devices for older persons, individuals with a disability and their support persons	2017	Disability and Rehabilitation: Assistive Technology	Williamson, B. and Aplin, T. and de Jonge, D. and Goyne, M.	10.1080/17483107.2016.1272140	To explore the acceptability and value of three wearable GPS devices for older persons and individuals with a disability and safety concerns when accessing the community.	Quantitative	Australia	older persons, individuals with a disability and their support persons	patient: 2M, 4F age 20-92; carer: 3F 3M, age: 47-68	exploring the acceptability and value of wearable GPS devices for older persons, individuals with a disability and their support persons			On average, participants identified that they were neutral about purchasing the pendant and not likely to purchase the other two devices.		The pendant was reported as being easy to use, easy to wear, helpful in knowing someone was there to contact and that the GPS location was always available. Similarly, the mini GPS phone was reported as being easy to use and that the size and weight were appropriate. The common theme identified for the watch was that participants did not find this device helpful.Following all device trials, the support persons and wearers were most satisfied with the pendant and least satisfied with the watch, with 75% of participants identifying that they would use the pendant in the future and would recommend it to others. A large proportion of participants said that they would not use the mini GPS phone (65%) or the watch in the future (80%). Whilst participants indicated that they would recommend the mini GPS phone (65%), they reported they would not recommend the watch (80%).The pendant was identified as the most comfortable device by 80% of participants. The majority (66%) of participants scored the mini GPS phone as "neutral" and 60% of participants found the watch to be "uncomfortable". Both support persons and wearers indicated no concerns with the appearance of the pendant and the mini GPS phone; however, some participants indicated that the appearance of the watch was too "childish" and "bulky" for an adult to wear. The majority of support persons (86%) associated positive descriptions (e.g., trendy, stylish) with the pendant compared with the watch (67%) and the mini GPS phone (60%). Similarly, wearers associated positive descriptions with the pendant (80%) and the mini GPS phone (75%). In contrast, a large proportion of wearers (67%) associated negative descriptors (e.g., clinical, old) with the watch. Participants indicated that they only received positive comments from others about the devices.		Support persons identified the most important feature of wearable GPS devices as being "easy to use", with all support persons giving this feature a rating of "important" or "very important". In contrast, wearers identified "two-way communication" as most important, with all wearers rating this feature as very important. Wearers also identified "easy to use", "falls detection" and "accuracy of location" to be highly important features. Support persons and wearers both indicated the least important features as being lockable, speed detection and geofencing.			In the initial questionnaire, the most valuable aspect of a wearable GPS device identified by support persons was "I would like him/her to be able to contact me or another person when he/she needs help". Wearers identified the most valuable aspect to be "I want my Key Support/Contact to know where I am located". In addition to this, both participant groups agreed that the second most valuable aspect of using a wearable GPS device was for the wearer to feel safe when out and about in the community.	Ethical considerations<br>Overall, support persons and wearers indicated limited concern regarding their privacy when trialling the devices.	UNDECLARED

Patient Satisfaction with Virtual Obstetric Care	2017	Maternal and Child Health Journal	Pflugeisen, B.M. and Mou, J.	10.1007/s10995-017-2284-1	The purpose of this study was to compare the satisfaction of obstetric patients who received one-third of their antenatal visits in videoconference (Virtual-care? compared to those who received 124 face-to-face visits in-clinic with their physician/midwife (traditional-care?.	Quantitative	USA	patient	In early March, 2016, patient satisfaction surveys were mailed to 430 Virtual-care and 860 Traditional-care patients. These patients were 69% Caucasian and 32% had subsidized insurance. Surveys were determined to be undeliverable to 7.6% Traditional-care (N = 65) and 12.1% Virtual-care (N = 52) patients; denominators were adjusted on the assumption that a total of 378 Virtual-care and 795 Traditional-care patients received the survey. A total of 96 (12.1%) Traditional-care and 75 (19.8%) Virtual-care patients responded, yielding a significantly higher response rate among Virtual-care patients (estimated difference = 7.7%; 95% CI 3.2, 12.4%; p < .001). Virtual-care and Traditional-care respondent backgrounds were comparable, including employment status during pregnancy (p = .09), partner status during pregnancy (p = .16), self-reported Caucasian race (p = .57), home < 5 miles from prenatal care	Virtual Obstetric Care				The requirement that Virtual-care patients connect via videoconference and use a handheld Doppler and blood pressure cuff did not significantly impact overall satisfaction of the Virtual-care respondents. However, questions in this domain scored considerably lower for Virtual-care patients than other domains. While ease of using the blood pressure monitor and Doppler were only slightly lower than the majority of questionnaire item averages, scores for ease of connecting for the visits and quality of connection during the visits were markedly lower.								UNDECLARED
Telemetric intra-cranial pressure monitoring: clinical and financial considerations	2017	British Journal of Neurosurgery	Barber, J.M. and Pringle, C.J. and Raffalli-Ebezant, H. and Pathmanaban, O. and Ramirez, R. and Kamaly-Asl, I.D.	10.1080/02688697.2016.1229752	We have evaluated a Telemetric ICP monitoring system that has been proven, by several other groups worldwide, to permit rapid, repeated and prolonged ICP measurement, in multiple environments.	Mixed	UK	patients with complex neurosurgical conditions.	Four patients aged between 4 and 16 have been implantedto-date, with the telemetric devicesin situbetween 460 and 632days	Telemetric intra-cranial pressure monitoring			Subjective feedback from both the patients (where pos-sible) and their families was overwhelmingly positive		partly due to (a) the system's ease of use, (b) its abil-ity to reduce the number of admissions/tests required and (c) the facility for rapid measurement of ICPthat permitted on-the-spot reassurance of concerns.					The combination of both this andthe ability to markedly improve patient experience, along with generating significant cost-savings, lead theauthors to suggest that the implantation of this system should be strongly considered in selectedindividuals.		N
A case study on older adults' long-term use of an activity tracker	2017	Gerontechnology	Schlomann, A.	10.4017/gt.2017.16.2.007.00	This paper aims at understanding older adults' long-term use of an activity tracker, at identifying reasons for continued or abandoned use and at evaluating possible effects of activity tracking on the level of physical activity.	Qualitative	USA	older adults	Participants' demographic information.<br><br>Characteristic Nonusers (n=17) Short-term users (n=9) Former users (n=11) Long-term users (n=11) All users (N=48)<br>Average age (years), mean (SD) 72.9 (7.5) 72.2 (9.9) 68.9 (2.5) 68.0 (3.1) 70.8 (6.7)<br>Age, range 66-94 66-94 67-73 65-73 65-94<br>Race or ethnicity, n (%)<br><br>White 15 (88) 7 (82) 11 (100) 9 (82) 42 (88)<br><br>African American 0 (0) 2 (18) 0 (0) 2 (18) 4 (8)<br><br>Hispanic 1 (6) 0 (0) 0 (0) 0 (0) 1 (2)<br><br>Asian 1 (6) 0 (0) 0 (0) 0 (0) 1 (2)<br>Gender, n (%)<br><br>Male 7 (41) 2 (18) 2 (18) 2 (18) 13 (27)	The Use of Wearable Activity Trackers Among Older Adults	Many participants occasionally referred to themselves as being laggards and luddites, that is, not being technologically savvy. Short-term users described themselves as having health issues and being active. Long-term users also mentioned having health issues but were more likely to describe themselves as being technologically savvy and early technology adopters.					Short-term and former users were also motivated by the presence of others<br>The presence of others was the top motivator for long-term users.<br>Long-term users, compared with other participants, perceived external factors, such as change of weather, as opportunities.	Some nonusers suggested they would be interested in increasing awareness of their daily activity as it could encourage them to move more.<br><br>Ideal Tracker: Prettier, Bigger, and More Comfortable<br>In their descriptions of an ideal tracker, nonusers expressed great interest in a possibility to monitor diverse activities, such as biking, water aerobics, golf, and others. Nonuser participants also suggested that an ideal activity tracker should count calories burned, monitor heart rate, look good, and be user friendly.<br><br>After trying activity trackers, nonusers found 4 features and functions to be useful: it could be used as a watch to show date and time, it had a comfortable band, it tracked sleep, and it was waterproof. Interestingly, the step count feature was not a favorite feature among the nonusers as most perceived step quantification to be inaccurate.<br><br>After the activity tracker trial, nonusers' preferences for an ideal activity tracker notably changed. Although they continued to suggest that activity trackers should be aesthetically appealing and not ugly, they focused on activity tracker band comfort, which, according to many participants,<br><br>Another characteristic of an ideal tracker that was mentioned by	After the activity tracker trial was over, nonusers continued to ask questions about how the device worked. Their skepticism toward and disappointment with the technology increased because they found that activity trackers had given inaccurate information. It also had limitations in terms of features offered. Participants compared actual step recordings with their perceived activity and found obvious inconsistencies:<br>Other reasons for nonadoption included high price, physical limitations that older adults face as well as inactive lifestyle, little interest and curiosity in trying activity trackers, band discomfort, and technology use difficulties. Technology-related barriers were discussed more extensively after the nonusers tried activity trackers, especially with regard to mobile phone ownership and synchronizing activity trackers with it.<br>Short-term and former users added that defects in activity trackers could or did make them stop using the activity tracker. One short-term user briefly discussed the issue of keeping personal data private when information from the activity tracker is synchronized with a mobile phone or computer and shared on social networks.<br>A few former users also indicated that having a short battery life and losing activity trackers were the reasons why they stopped using the device.		Several nonuser participants named long-term benefits, such as staying away from medications, feeling better, losing weight, and improving physical indicators of health (heart rate and blood pressure), as motivations that could drive tracker use.Long-term users were more likely than participants in other focus groups to see long-term benefits of regular physical activity and, consequently, activity tracker use, such as physical and emotional health (eg, stress reducer), reduced pain, independence and mobility, and long life:	With the lack of knowledge came skepticism as nonusers questioned the ways in which activity trackers worked. Inaccuracy in counting steps raised most of the concerns.	UNDECLARED
Tablet-based well-being check for the elderly: Development and evaluation of usability and acceptability	2017	JMIR Human Factors	Ray, P. and Li, J. and Ariani, A. and Kapadia, V.	10.2196/humanfactors.7240	The aim of this study was to iteratively design, implement, and assess the usability, user friendliness, and acceptability of a tablet-based system to check the well-being of the elderly.	quali	Australia and India	older adults	Demography Number of participants (N=7)<br>Age (in years)<br><br>60-70<br>71-80<br>Above 80 4<br>2<br>1<br>Gender<br><br>Male<br>Female 1<br>6<br>Level of education<br><br>Primary school<br>Secondary school<br>Tertiary school 1<br>3<br>3	Tablet-Based Well-Being Check for the Elderly			Attitude All participants had a positive attitude toward the system and they would like to continue using this system in the future.		Ease of Learning All participants used this system regularly, except one, because of her sickness. They were able to independently operate basic functions like pressing green or red buttons on the screen. However, they were struggling to send messages or to play Web-based games.<br>The researchers also noticed that most elderly people preferred face-to-face learning environment to the user manual. This might be caused by their low technology skills and unfamiliarity with built-in help functions.<br><br>Language barriers had a profound impact on the elderly's learning process. For those with English as a second language, their children attended all training sessions. Participants were more comfortable with training given in their mother tongue.<br><br>Ease of Use In this trial, 5 participants used an iPad and the other 2 used an Android tablet. The latter faced more technical difficulties and were less happy with the size of the screen in comparison with the former. The Android tablet had a shorter battery life and a more sensitive touch screen, which caused more incidents of websites closing accidentally. In addition, because of their unfamiliarity with touch screen devices, the elderly were anxious in using all features of the system at the beginning of the trial.<br>All participants found it difficult to open the prototype page when they accidently closed the Web browser. Even after training, they were not able to open their account page on the screen by themselves. Each requested easier ways to navigate through their account. A shortcut could be created to the home screen to avoid multiple steps or a pop-up alert like "Are you sure you want to close this program—Yes or No"		Design of Interface Some elders might have forgotten to press "I am OK" due to age-related memory loss and dementia. It would be useful to have another option to automatically generate an "OK" message when the user touches any part of the tablet screen. This feature would enable the service provider to continuously monitor the elderly's current condition.<br>Another comment was that pop-up reminders for reminding were too small. One recommendation was to provide a sound alert feature that would notify the elderly when a new message was received or when the medication needed to be taken.<br><br>A few who received many photos from their family had difficulties in identifying new photos from old ones. Difficulties were always found in searching for particular photos since the system was set to automatically scroll down the photos. Some recommendations included the use of timestamp when displaying frames, the Trash Can feature for deleting old photos or multiple duplicate photos, and a scroll feature that enables the user to manually scroll through the photos. A few participants questioned the usability of the system in the monitoring of well-being. They were looking for a system with an emergency alert feature. For instance, for fall incidents, immediate medical intervention might be required. It is important to mention that this well-being check system has been designed to support the elderly's independent living and not for an emergency alert.			Perception of Usefulness Participants agreed that their social connections with their family were significantly improved by using this system. It was also revealed that the ongoing involvement and motivation of the family positively affected their willingness to use such a system.<br>In addition to personalized communication, many participants found pop-up messages for medication reminders useful . One service provider suggested its integration with medication management systems so that care workers would not always be required for the administration of medication to the elderly.<br><br>Overall, digital photo frames have a greater chance of being accepted by the elderly living in residential settings [55]. The digital photo frame is generally easier to obtain and this technology merged with a familiar object, only software modifications are needed when upgrading the system and the process is much simpler when compared with other technologies that required hardware modifications [55].		N
Usability, acceptability, and adherence to an electronic self-monitoring system in patients with major depression discharged from inpatient wards	2017	Journal of Medical Internet Research	Lauritsen, L. and Andersen, L. and Olsson, E. and S冀dergaard, S.R. and N鉅regaard, L.B. and L鴇entoft, P.K. and Svendsen, S.D. and Fr鏑j鍒, E. and Jensen, H.M. and Hageman, I. and Kessing, L.V. and Martiny, K.	10.2196/jmir.6673	We aim to test the usability, acceptability, adherence, and clinical outcome of a newly developed computer-based electronic self-assessment system (the Daybuilder system) in patients suffering from depression, in the period from discharge until commencing outpatient treatment in the Intensive Outpatient Unit for Affective Disorders.	Quantiative	Denmark	depression patients	Mean age was 35.9 years (SD 10.8). Patients had 2.5 previous episodes of major depression (SD 5.7) and a mean duration of current depression of 10.2 months (SD 11.7). Most patients were on sick leave. Only a few patients had attempted suicide in the current episode before admission. Patients were treated with 1.9 drugs (SD 0.9; range 0-4): antidepressants included Selective Serotonin Reuptake Inhibitors (n=12), Serotonin-Norepinephrine Reuptake Inhibitors (n=15), Noradrenergic and Specific Serotonergic Antidepressants (n=11), mianserin (n=11), Tricyclic Antidepressants (n=15), isocarboxazid (n=1), and agomelatine (n=1); antipsychotics included quetiapine (n=8) and olanzapine (n=2); mood stabilizers included lithium (n=5) and lamotrigine (n=4); benzodiazepines (n=3); hypnotics (n=3); and melatonin (n=2).	Self-Monitoring System in Patients With Major Depression Discharged From Inpatient Wards			In general, patients found that the Daybuilder system lived up to their expectations; however, when evaluated at endpoint, patients found that they had registered less data than they had anticipated at baseline (Table 2). Fifty-nine percent of patients (20/34) believed that the system could detect a relapse, and 50% of patients (17/34) believed that the system could influence the course of their illness.		At endpoint, only 50% of patients (17/34) felt that the system had covered their needs for self-monitoring. A total of 33 patients filled in the SUS scale, with a mean value of 86.2 (SD 9.7; range 65-100). Patients not receiving ECT (n=27) had an SUS score of 86.9 (8.9; range 65-100) and patients receiving ECT (n=6) had a score of 82.9 (13.0; range 65-97.5; P=.40).<br><br>The importance of the weekly phone calls was rated on a scale from 0 to 10 (10=highest importance): 58% of patients (19/33) rated in the interval 8-10, 33% (11/33) in the interval 5-7, and only 9% (3/33) in the interval 0-4. The frequency of phone calls was deemed appropriate by 35% of the patients (12/34). Twenty-six percent of patients (9/34) would have liked more frequent phone calls or a combination of consultation and telephone contact, 12% (4/34) suggested being contacted when the Daybuilder registrations showed signs of deterioration or if data entry was missing, 9% (3/34) felt a need for a more flexible and individualized design that would depend on their mental state, and 18% (6/34) did not answer this question.<br><br>Only 9% of patients (3/34) were worried about technical problems and 79% of patients (27/34) found that the system lived up to their expectations. Thirty-two percent of patients (11/34) felt that their self-assessment of mood in the Daybuilder system had influenced their mood, but only one patient felt that it had a negative effect. Adherence with data entry into the Daybuilder application for the 34 completers was high: mood on 93.8% of the days (872/930), sleep on 89.8% of the days (835/930), activity on 85.6% of the days (796/930), and medication on 88.0% of		Other semiqualitative questions at baseline showed that patients expected the Daybuilder system to enable visualization of their condition, give support and structure, enable positive expectations, or give a hope of recovery. Several additional self-monitoring items were suggested, such as social activity, appetite, meals, anxiety, cognitive function, medication, and side effects.					Lasse Benn Nørregaard and Philip Kaare Løventoft are directors of Daybuilder Solutions, were involved in the development of the study version of the Daybuilder system that was used, and have assisted with technical support during the study. Lasse Benn Nørregaard and Philip Løventoft have not been involved in any clinical interaction or assessment of participants in the study. Lars Vedel Kessing was a consultant for Lundbeck and Astra Zeneca within the last three years. All other authors report no conflicts of interest.
Implementation of a home monitoring system for heart failure patients: A feasibility study	2017	JMIR Research Protocols	Kohn, M.S. and Haggard, J. and Kreindler, J. and Birkeland, K. and Kedan, I. and Zimmer, R. and Khandwalla, R.	10.2196/resprot.5744	The Cedars-Sinai Cardiology Center provides a high-touch, intense management program for patients with congestive heart failure (CHF). The objective of our study was to determine whether patients already actively engaged in a high-touch intensive management program for CHF would take on the additional burden of a complex home monitoring effort.	Quantitative	USA	Heart Failure Patients		a Home Monitoring System for Heart Failure Patients			All patients either agreed or strongly agreed that they felt better connected to their care teams and that taking the measurements was worthwhile.							It was found that 8 of the 11 patients (73%) felt better about their health as a result of the program, whereas another 8 of 11 (73%) believed that the care team now had a better picture of their health.		All Sentrian authors are employees, and stockholders, in Sentrian. All Cedars-Sinai authors are employees of Cedars-Sinai Medical Center.
Expanding stroke telerehabilitation services to rural veterans: a qualitative study on patient experiences using the robotic stroke therapy delivery and monitoring system program	2017	Disability and Rehabilitation: Assistive Technology	Cherry, C.O. and Chumbler, N.R. and Richards, K. and Huff, A. and Wu, D. and Tilghman, L.M. and Butler, A.	10.3109/17483107.2015.1061613	The present study reports on a robotic stroke therapy delivery and monitoring system intervention. The aims of this pilot implementation project were to determine participants' general impressions about the benefits and barriers of using robotic therapy devices for in-home rehabilitation.	Qualitative	USA	rural veterans	10M,49-88,Marital status M M M M S S M D S M<br>Employment R, D R, D D R, D D D D D D R<br>Education Level 11 11 12 12 12 16 14 12 12 9<br>Living situation Lives with wife, child, grand-child Lives with wife, child, grand-child Lives with wife Lives with wife Lives with other adult Lives alone Lives with wife Lives with mother and son Lives with other adult Lives with wife	Expanding stroke telerehabilitation services to rural veterans								Barriers to use<br>Although feedback on the devices was overwhelmingly positive there were some barriers to use and suggestions to improvement that were identified during interviews. These included: size and placement of the device, technical difficulties, and wearing and adjusting the device.<br><br>Size and placement<br>One of the complaints about the devices was their size and weight, and the resulting difficulty of moving them around the home as a result.<br><br>Wearing and adjusting the device<br>The most consistent barrier to using the devices reported by participants was difficulty putting the device on and adjusting it by themselves.<br><br>Technical difficulties<br>One technical complaint centered on the computer or software becoming unresponsive or acting erratically.		The following overarching themes were identified as positive benefits of using the devices: increased mobility, a sense of control over therapy and scheduling, an outlet for physical and mental tension and anxiety and increased independence.		N
Analysis of user acceptance of the electronic malaria monitoring system based on technology acceptance model	2017	Journal of Telecommunication, Electronic and Computer Engineering	Lestantri, I.D. and Kardiana, A. and Budi, I. and Indra, D.		Malaria Monitoring System Electronic (e-Mamosys) is an application system developed in an effort to monitor patients with malaria. The application was tested to investigate the effects of beliefs, attitudes towards the acceptance of users.	Quantitative	Indonesia	General public		Electronic Malaria Monitoring System				personalization influential variables have positiverelationshipto both the dependent variable (perceived usefulness and perceived ease of use). In contrast to the variable of computer self-efficacy and trust, theyhave negative effects on both the dependent variables. Judging from the positive effect of the variable personalization, user's information needsif themalaria monitoring system can provide information in accordance with the wishes and needs of the user.At this time,the variable self-efficacy and trust have no effect, hence there are future needs to restore the system from the convenience factor and scalability.The number of respondents should be increased in terms of area, age, education to expand the testing area so that research results can be generalized. Further, other variables should be added to look at other factors thatmayinfluenceuser's acceptance of the application								UNDECLARED
Toward patient-centered telerehabilitation design: Understanding chronic pain patients' preferences for web-based exercise telerehabilitation using a discrete choice experiment	2017	Journal of Medical Internet Research	Cranen, K. and Groothuis-Oudshoorn, C.G.M. and Vollenbroek-Hutten, M.M.R. and IJzerman, M.J.	10.2196/jmir.5951	To identify which telerehabilitation treatment options patients with chronic pain are most likely to accept as alternatives to conventional rehabilitation and assess which treatment attributes are most important to them.	Mixed	Netherlands	Chronic Pain Patients	The majority of the research sample (mean age 43.8 years, SD 14.8) was female (66 out of 104) and had completed a middle-high education (51 out of 104 participants). The majority of the respondents were unemployed (69 out of 104 participants) at the time and had Internet access (97 out of 104 participants). Patients' mean visual analogue scale (VAS) pain score was 6.3 and pain complaints varied in the lower back, hip, knee, joint, and neck areas and lasted longer than 6 months (Table 2).	Chronic Pain Patients' Preferences for Web-Based Exercise Telerehabilitation			[illegible] their consultation via video communication; however, having all consultations with video camera was the least preferred option. Furthermore, patients favored the use of feedback and monitoring technology while exercising and preferred to exercise at a gym location. In addition, they preferred physician contact every session and flexible exercise sessions and favored the highest discount on their health care premium. Conversely, respondents preferred not to undergo treatment that involved video consulting and minimized physician contact, exercising individually in the home environment without feedback and monitoring technology at fixed time frames. The attribute levels are generally well ordered, except for the attribute "consulting frequency." Less frequent supervision (once per 4 exercise sessions) is preferred over more frequent supervision (once per 3 exercise sessions).									N
Informing the design of "lifestyle monitoring" technology for the detection of health deterioration in long-term conditions: A qualitative study of people living with heart failure	2017	Journal of Medical Internet Research	Hargreaves, S. and Hawley, M.S. and Haywood, A. and Enderby, P.M.	10.2196/jmir.6931	The objective of this paper was to report the findings of a study aimed at identifying changes in activity indicative of important changes in health in people with long-term conditions, particularly changes indicative of exacerbation, by exploring the relationship between home activities and health among people with heart failure (HF). We aimed to add to the knowledge base informing the development of home monitoring technologies designed to detect health deterioration in order to facilitate early intervention and avoid hospital admissions.	Qualitative	UK	patients in Long-Term Conditions	[illegible] the interview participants with HF. Participants ranged in age, from younger participants in their 50s to older participants in their 80s, with the majority in their 70s. In addition, many participants suffered from a range of co-morbidities from mild conditions (such as mild arthritis) to potentially life-threatening ones (eg, cancer). The sample included participants who had been diagnosed with HF recently (within the last three years), and those who had been living with HF for longer. Despite efforts to recruit a gender-balanced sample, the sample was predominantly	Lifestyle Monitoring Technology for the Detection of Health Deterioration in Long-Term Conditions					The interviews illustrate that in real life, the relationship between activity and health is not clear cut, thus LM systems would need to take account of this complexity and individual circumstances.		The interviews also highlighted the importance of signs of ill-health relating to appearance, which would require new sensors to be developed. An important sign was swelling. More subtle signs noted by the partners of participants with HF were changes in facial appearance including expression, appearance of eyes, blue lips, and alteration in mood and demeanor.			During an exacerbation of symptoms, participants with HF, for example, described both changes to existing activities (eg, reduced speed in walking and climbing the stairs, staying at home more, and being more sedentary) and undertaking new activities in response to distressing symptoms (eg, sitting up at night to aid breathing, perhaps watching television).		N
Health information privacy of activity trackers	2017	European Conference on Information Warfare and Security, ECCWS	Lehto, M. and Lehto, M.		This paper discusses a study regarding the user perception on the privacy and sensitivity of health information collected with wearable devices. The paper also explores the user perception on health information sensitivity in general, and their willingness to share such information to other parties.	Quantitative	Nigeria	General public	Of the 270 participants, 77.8% (n=211) were male, and the rest (22.2%, n=59) were female. The majority of the participants were between the age of 25 and 34 years (163 participants), the second-largest group was 35-44 years old (39 participants), 33 participants were between the age of 18 and 24 years, 21 participants were between the age of 45 and 54 years, and 14 participants were 55 years old or older.	Health information privacy of activity trackers				Granular Privacy Control<br>Hypothesis 1 states that increased control results in a higher intention to share. Findings supported this result. The path coefficient for granular privacy control was positive and significant (β GPC=.36, P<.001), indicating that granular privacy control yields a higher willingness to share personal fitness-related information with health care providers.<br><br>Perceived Risk<br>Hypothesis 2 proposes a positive relationship between negative perceived risk and the intention to share fitness information. The results provided evidence to support this hypothesis (βPR=−.17, P=.027), which confirms the impact of the possible risk that involves sharing information via systems and with others.<br><br>Perceived Benefits<br>Hypothesis 3 argues that perceived benefits will yield a higher intention to share fitness information with health care providers. The estimate for this relationship was positive and								UNDECLARED
Patient-centered design criteria for wearable seizure detection devices	2016	Epilepsy and Behavior	Patel, A.D. and Moss, R. and Rust, S.W. and Patterson, J. and Strouse, R. and Gedela, S. and Haines, J. and Lin, S.M.	10.1016/j.yebeh.2016.09.012	A survey instrument utilizing a population of patients and caregivers was created to obtain information on the design criteria most desired for patients with epilepsy in regard to wearable devices.	Quantitative	USA	patients with epilepsy		Patient-centered design criteria for wearable seizure detection devices			Respondents thought that sensors for muscle signal (61.4%) and heart rate (58.0%) would be most helpful followed by the O2 sensor (41.4%). There was more interest in these three sensor types than for an accelerometer (25.5%). There was very little interest in a microphone (8.9%), galvanic skin response sensor (8.0%), or a barometer (4.9%).		Based on a rating scale of 1–5 with 5 being the most important, respondents felt that "detecting all seizures" (4.73) is the most important device feature followed by "text/email alerts" (4.53), "comfort" (4.46), and "battery life" (4.43) as an equally important group of features. Respondents felt that "not knowing device is for seizures" (2.60) and "multiple uses" (2.57) were equally the least important device features.			Average ratings differed significantly across age groups for the following features: button, multiuse, not knowing device is for seizures, alarm, style, and text ability. The p-values were all < 0.002. Eighty-two point five percent of respondents [95% confidence interval: 80.0%, 84.7%] were willing to pay more than $100 for a wearable seizure detection device, and 42.8% of respondents [95% confidence interval: 39.8%, 45.9%] were willing to pay more than $200.				UNDECLARED

Impact of self-funding on patient experience of oral anticoagulation self-monitoring: A qualitative study	2016	BMJ Open	Tompson, A. and Heneghan, C. and Sutton, S. and Fitzmaurice, D. and Ward, A.	10.1136/bmjopen-2016-013123	To explore the impact self-funding has on patient experience of oral anticoagulation therapy selfmonitoring.	Qualitative	UK	OAT patient	Thesamplehadamedian ageof54years(range32–87years)andcomprisedof10 men(38.5%).Themajorityhadeitheraprofessional (11,42.3%)oruniversityqualification (9,34.6%)and tendedtoliveinlessdeprivedneighbourhoods(median IndexofMultipleDeprivationScore2010of 10.4).12 Mostinterviewees(11,42.3%)requiredanticoagulation duetoprevioustrombosis;eight(30.8%)dueto havingamechanicalheartvalve;four(15.4%)because ofatrialfibrillationandthree(11.5%)hadantiphospholipidsyndrome.	oralanticoagulation self-monitoring								Interviewees viewed purchasing the monitoring device as along term commitment balancing the limitations of clinic based monitoring against the cost.				N
Patient-Centered Home Care Using Digital Medicine and Telemetric Data for Hypertension: Feasibility and Acceptability of Objective Ambulatory Assessment	2016	Journal of Clinical Hypertension	DiCarlo, L.A. and Weinstein, R.L. and Morimoto, C.B. and Savage, G.M. and Moon, G.L. and Au-Yeung, K. and Kim, Y.A.	10.1111/jch.12787	This study utilized digital medicine (DM) prototypes and telemetric data acquisition to directly confirm medication use and to assess habits of daily living in a hypertensive population.	Quantitative	Canada	patients with Hypertension	Patients, No. 37 Men, No. (%) 23 (62) Race (Caucasian/black/Asian), % 77/5/18 Education (high school/college/graduate school/other), % 23/54/21/2 Height (cm)/weight (kg), ±standard deviation 169±11/90±22	Patient-Centered Home Care Using Digital Medicine and Telemetric Data for Hypertension			Ninety percent of patients did not mind swallowing the DM capsule, and 75% had a positive overall experience using the system. When asked more specifically, the majority of patients did not mind wearing the adhesive sensor for even longer periods, and expressed willingness to use such a product if it helped their physicians to take better care of them.		No serious adverse events, unanticipated adverse events, or study procedure–related adverse events occurred during the study. Mild and transient WS-related skin irritation occurred in 14 patients (40%), and there were no adverse events related to the IS.	When asked more specifically, the majority of patients did not mind wearing the adhesive sensor for even longer periods, and expressed willingness to use such a product if it helped their physicians to take better care of them.						UNDECLARED
Device development guided by user satisfaction survey on auricular vagus nerve stimulation	2016	Current Directions in Biomedical Engineering	Kampusch, S. and Kaniusas, E. and Th[illegible]k, F. and Felten, D. and Hofmann, I. and Sz[illegible]es, J.C.	10.1515/cdbme-2016-0131	In this paper, we present a survey on satisfaction with and demands for a wearable medical device for percutaneous auricular vagus nerve stimulation (pVNS).	Quantitative	Vienna	patients with percutaneous auricular vagus nerve stimulation (pVNS)	From 40 questionnaires for patients 36 (90%) were completed and fulfilled the inclusion criterion. In addition, five questionnaires were completed by operators aged 32.25 ± 12.9 years (50% female) having experience with pVNS therapy of at least 6 months. Responding patients were aged 57.2 ± 17.4 years (25% female) and suffered from chronic lower extremity ulcers (41.7%), chronic pain (33.3%), neurological disorders (25%), acute pain (19.4%), peripheral arterial occlusive disease (8.3%) and other diseases (8.3%) with a history of the disease of 6.1 ± 6.4 years (the total range from 2 weeks to 28 years). Treatment durations of included patients range from 1 week up to 10 years. 48.6% of patients tried other therapies before pVNS treatment with a satisfaction rate of only 22.4%.	a wearable medical device for percutaneous auricular vagus nerve stimulation (pVNS).			Considering overall treatment satisfaction, 82.3% of patients are (very) satisfied, 17.6% sign for neutral and nobody is very dissatisfied or not satisfied. Patients report an improvement in life quality due to pVNS therapy of 1.7 ± 2.8 on a 10 point interval scale from very bad to very good.		Although satisfaction is high, 41.2% of all patients report unwanted side effects (at least once during treatment). This mainly accounts for skin irritation at neck (60%) and ear (26.7%), pain (26.7%), inflammation (20%), bleeding at ear (13.3%), and slight dizziness (13.3%).One fundamental shortcoming of the current device was identified to be the missing robustness for water, since all patients and operators claim the need for the device to be watertight, and thus enable, e.g. showering.Open questions for potential improvements identified the following main shortcomings: limited battery life time, lacking resistance to water, missing flexible design with soft cables, and missing autonomy of the patient and operator (e.g. stimulation amplitude). On the other hand, patients and operators are attracted by the minimal-invasive nature of pVNS, as there are no pharmaceutical components involved and thus there are no pharmaceutical side effects		Patients and operators declared no clear preference concerning the mounting regions of the device. 80.5% of patients want the device fixed with an adhesive at a position near the ear (66.6%), in contrast to hanging it over the ear or the neck. Patients would also accept a placement on the chest, back or the upper arm. There is no clear preference of patients and operators concerning the shape of the device.<br><br>Concerning the user interface, several possibilities of device operation were offered to patients and operators. 81.1% and 100% of all patients and operators, respectively, want to adjust the stimulation amplitude in accordance with the momentary perception. 84.4% of patients and 60% of operators want the stimulation amplitude to be adjustable by both patient and operator. There is no preference for any asked means of interaction with the device (e.g. by buttons). 40.5% and 72.2% of all patients want to disable or take off the device, respectively, in specific situations like showering or when they need to be concentrated, like during car driving.<br><br>An important boundary condition for an effective therapy is the required time interval between two pVNS applications, which depends strongly on the particular disease of the patient and is limited by battery lifetime of the device. On average, patients suggest a time interval between two consecutive treatments of 9.4 days with a range from 3 (wound treatment) up to 21 (chronic pain treatment) days. The device			Concerning activities of daily living (Figure 2), the device application dominantly influences personal hygiene and sleeping, where 31% and 21.4% of the patients report (very) negative influence, respectively.		S. Kampusch, E. Kaniusas and J.C. Szeles are shareholders of SzeleSTIM GmbH.
Truck drivers' perceptions on wearable devices and health promotion: A qualitative study	2016	BMC Public Health	Greenfield, R. and Busink, E. and Wong, C.P. and Riboli-Sasco, E. and Greenfield, G. and Majeed, A. and Car, J. and Wark, P.A.	10.1186/s12889-016-3323-3	We aimed to explore professional truck drivers' views on health promotion delivered via mobile health technologies such as wearable devices.	Qualitative	UK	Truck drivers	The drivers were 42.2 ± 7.8 years old on average, and on average had been in the profession for 16.1 years. All of them were male, and most of them drove a HGV at long distance. Most of them perceived their health as "good" and their lifestyle as "healthy"	Truck drivers' perceptions on wearable devices and health promotion	About a third of the drivers mentioned that they already used mobile health technology devices (mostly apps on their smartphones), but most of them had low or no experience with wearable health technology devices, and were unaware of what kind of wearable health devices are available and which ones are most suitable for their needs.					We identified two sources of motivation for truck drivers for using wearable devices. The first one was positive motivation based on the potential of wearable devices to help prevent undesired health conditions.<br><br>Other drivers wanted to make use such monitoring devices because they were fearful of developing health conditions. They felt that changes or deterioration of their health conditions could be detected by such devices.	When asked what they would expect from such a device, the drivers highlighted the need to have simple, straightforward functions, which should be customized according to their lifestyle:			Several drivers were not sure whether having a health monitoring device at work would be merely of economic interest to the organisation, or whether having health monitoring wearable devices would create a mutual benefit for both the employers and the drivers. Several participants referred to the company's health promotion programme that was launched after the recent fatal events, as a positive change aiming to reduce their health risks and improve wellbeing.<br><br>Possibly due to their work conditions whom they described as 'lonely', the drivers felt reassured to have a device that will be constantly with them and will monitor their health, either as a preventive measure, or out of fear of developing diseases.<br><br>Another aspect of being continuously monitored is their personal data including health conditions being available to their employer. They perceived mHealth monitoring as a way to improve the drivers' work conditions, as the management will have a better view on drivers' work performance	However, other drivers were suspicious about the intentions behind the organisation's efforts (i.e., whether they authentically care for the drivers or motivated by sheer economic interests). They thought that the organisation's investment in the truck drivers' health was also related to the occurrence of several cardiovascular related deaths occurred. Therefore the drivers wondered if the company just took a pragmatic approach to sustain its workforce:<br><br>Since sharing drivers' data with employers may provide indicators on their employees' health, drivers' work performance can also come under scrutiny. Hence, the data can provide evidence on performance, which may or may not protect drivers from being judged unfairly<br><br>The reassurance and benefits of being monitored come at the expense of data privacy and concerns about who will have access to the data collected from the wearable	In 2014, we were collaborating with Philips Research, Telecom Italia (Joint Open Lab, Pisa) and Scuola Superiore Sant'Anna to co-design a health promotional service for professional drivers. This activity was funded by the European Institute of Innovation and Technology (EIT) Digital, under the Health and Wellbeing Action line 'Personal health and wellbeing self-management services (PHASER)' Activity (ID 14036). The current study was part of this EIT Digital activity that aimed to improve health and wellbeing of professional drivers, and had also a partially commercial nature. After exploring general perceptions on wearable devices and health promotional services that we described in this manuscript, we asked drivers about their perception of the proposed design of the specific product (which we do not present in this manuscript). This was declared in the information sheets, in the informed consent sheet and during the focus groups. We continue collaborating with industrial partners and EIT Digital to improve health and wellbeing of truck drivers.
Stakeholder attitudes toward and values embedded in a sensor-enhanced personal emergency response system	2016	Interacting with Computers	Dahl, Y. and Farshchian, B. and Vilarinho, T. and Helbostad, J.L. and Nawaz, A. and Nyg[illegible]d, A.J. and Wik, P.B.	10.1093/iwc/iwv036	This paper provides an empirical understanding of concerns that the application of a sensorenhanced medical alert system, or personal emergency response (PER) system, raises from the perspective of care receivers (users) and care providers.	Qualitative	Norway	care home residents	The user group consisted of 6 male and 14 female care receivers aged 28–103 years (mean | $\hbox {age} = 82.15$ |) living in residential care homes at the two care facilities.	Stakeholder Attitudes Toward and Values Embedded in a Sensor-Enhanced Personal Emergency Response System					Form factor and ergonomics: from the users' perspective, concerns related to form factor and ergonomic aspects of alert devices were: dynamic wearability, aesthetics and inclination of some users to wear the devices openly, and possibilities for self-managing of devices.					The care providers generally sympathized with the users' concerns. The care providers, however, highlight the perceived benefit of being able to visually identify, in an unobtrusive manner, that care receivers carried their alert devices with them.	intelligible system feedback when alert notifications were manually or automatically triggered appeared as a central issue among the users. The occurrence of false-positive fall detections by the sensor belt, and reluctance toward burdening care providers with non-critical issues led some users to argue for having the opportunity to prevent the fall detection belt from sending an undesired alarm notification.	N
Older people's perspectives regarding the use of sensor monitoring in their home	2016	Gerontologist	Pol, M. and Van Nes, F. and Van Hartingsveldt, M. and Buurman, B. and De Rooij, S. and Kr[illegible]e, B.	10.1093/geront/gnu104	The purpose of this qualitative study was to determine the perspectives of older people regarding the use of sensor monitoring in their daily lives.	Qualitative	Netherlands	Older People	The age of the participants ranged from 68 to 94 years, and the participants were all living alone in the community or a senior residence.	Older People's Perspectives Regarding the Use of Sensor Monitoring in Their Home			The majority of the participants seemed to easily accept the sensors as natural in their daily life.	acceptance of the sensor monitoring system at home.<br><br>The social environment's reaction to the sensor monitoring seemed to support the quick acceptance of a sensor system in the home. Most of the participants reported that their visitors or family did not notice the sensors unless the	An added benefit of sensor monitoring in the home is the user-friendliness of the technique. Most of the participants explained that they did not have any technical knowledge and expressed their relief that the sensors did not require any action of them.However, at times, one of the participants was reminded of the sensors in her home. She indicated that the sensor base unit reminded her of having sensors in the home because of a burning LED-light on this unit. This made her feeling uncomfortable. However, when the sensor base unit was placed out of her sight, this problem was solved.	However, other participants were curious about their sensor data and considered this information as helpful to them.		Several reasons were given for this lack of desire. Some participants stated that they did not have sufficient technological knowledge to understand the sensor data from the computer.Other participants stated that they did not want not see their sensor data because they did not want to think about their health problems.		Sensor monitoring contributes to the sense of safety as a premise for living independently alone at home as outlined above. These participants experienced the sensor system as a readily applicable system, giving them the confidence of accessible help.	The participants did not experience the use of sensor monitoring as an invasion of their privacy. On the other hand this system contributes for them to a certain dependency, which can be considered as an unattended side effect of sensor monitoring.	UNDECLARED
Community-Based ECG Monitoring System for Patients with Cardiovascular Diseases	2016	Journal of Medical Systems	Lin, B.-S. and Wong, A.M. and Tseng, K.C.	10.1007/s10916-016-0442-4	This study aims to develop a community-based electrocardiogram (ECG) monitoring system for cardiac outpatients to wirelessly detect heart rate, provide personalized healthcare, and enhance interactive social contact because of the prevalence of deaths from cardiovascular disease and the growing problem of aging in the world.	Quantitative	Taiwan	Patients with Cardiovascular Diseases	The study subjects are 125 older people (66 males and 59 females) in Chang Gung Health and Culture Village. Every subject must use the proposed system first and then complete the given questionnaire.	Community-Based ECG Monitoring System for Patients with Cardiovascular Diseases			Performance expectation on user intention to use the community-based ECG monitoring system is positive and significant.	intention to use the community-based ECG monitoring system. That is, users with user intention to use the system do not have a significant relationship of user endeavour. The anxiety of using the computer may be a reason for the elderly, even if they have no problem to operate the system. It implies that the endeavour expectation of the elderly exists some degree of uncertainty.<br><br>Social influence on user intention to use the community-based ECG monitoring system is positive and significant. It shows that behaviour intention of the elderly is influenced by opinions of other participates around and implies that the proposed system can be suggested to the elderly to improve their healthcare quality.		It implies that the designed system is helpful to the elderly, and they expect to use the proposed system to monitor their health status and give them some suggestions or feedback.			Facilitating condition on user intention to use the community-based ECG monitoring system is positive and significant. It means that the more technology assistance that the proposed system offers, the higher intensity for the elderly to use the system.			N
The influence of culture on older adults?adoption of smart home monitoring	2016	Gerontechnology	Fritz, R.L. and Corbett, C.L. and Vandermause, R. and Cook, D.	10.4017/GT.2016.14.3.010.00	This interdisciplinary study explored the influence of culture on older adults?adoption of smart home monitoring.	Qualitative	UK	older adults	Age: 65-90; caucasian, asian, immigration, retired/not retired	older adults' adoption of smart home monitoring											openness to smart home interventions, including monitoring. Openness depended on (i) the level and specificity of need and whether the smart home would meet that need, (ii) perceived loss of privacy compensated by a feeling of safety and a receipt of health-assistance, (iii) functionality, and (iv) cost.	UNDECLARED
The role of self-control in self-tracking	2016	2016 International Conference on Information Systems, ICIS 2016	Baumgart, R. and Wiewiorra, L.		Self-tracking is defined as using technology to monitor one's own behaviour e.g. sleeping habits or steps. Using established measurements from psychology we investigate how different levels of self-control influence the tracking behaviour of consumers and their expenditures for self-tracking software and hardware. Furthermore, we analyse what motivations to start self-tracking drive different self-tracking activities.	Quantitative	Germany	General public	In the final sample, 54% of the respondents were female and the youngest (oldest) participant was 18 (48) years old while the average age was 26.24 (median=26, SD=5.51).	consumers tracking physical parameters				the odds of consumers tracking physical parameters and spending more for self-tracking software and hardware. Furthermore, higher impulsivity has a negative effect on the likelihood of tracking medical parameters. Overall expenditures depend on the usage frequency of self-tracking tools. We conclude that users with low self-control value self-tracking to a lower degree because they are confronted with negative self-tracking results and emotions.		Tracking behaviour in general is driven by curiosity.						N
Quantify-ME: Consumer acceptance of wearable self-tracking devices	2016	24th European Conference on Information Systems, ECIS 2016	Pfeiffer, J. and Von Entress-F[illegible]steneck, M. and Urbach, N. and Buchwald, A.		In our research project, we aim at answering the question of what drives the usage intention of wearable self-tracking technology. Therefore, based on established technology acceptance theories, we deductively develop an acceptance model for wearable self-tracking technologies which sheds light on the pre-adoption criteria of such devices.	Quantitative	Germany	General public	The average participant is 26.4 years old and earned a university degree. 60% of the respondents are male.	Wearable SelfTracking Devices				usefulness, perceived ease of use, perceived enjoyment, social influence, trust and personal innovativeness to be significant determinants of the behavioral intention to use a wearable self-tracking device. In contrast, in the female group, we find support for the determinants perceived enjoyment, social influence, perceived aesthetics, personal innovativeness, perceived support of health and perceived support of fitness.. For participants younger than 25, we find support for perceived usefulness, trust, personal innovativeness and perceived support of well-being to be significant determinants of the behavioral intention to use a wearable self-tracking device, while for participants older than 25 the determinants perceived usefulness, perceived enjoyment, social influence, perceived aesthetics and personal innovativeness become								N
Mobile and wearable technology needs for aging in place: Perspectives from older adults and their caregivers and providers	2016	Studies in Health Technology and Informatics	Wang, J. and Carroll, D. and Peck, M. and Myneni, S. and Gong, Y.	10.3233/978-1-61499-658-3-486	There is an increasing number of wearable trackers and mobile devices in the burgeoning world of digital health, the purpose of the study is to explore the role of these mobile and wearable tools among older adults aging in place.	Qualitative	USA	Older Adults, Caregivers and Providers	Demographic Characteristics of Older Adult Sample Characteristics Assisted Living (n=17) Independent Living (n=12) Gender Female Race White Black Age (in Years: Mean) Had A Chronic Illness 12 (71%) 14 (82%) 2 (12%) 90 14 (82.4%) 9 (75%) 12 (100%) 0 86 8 (66.7%)	Mobile and Wearable Technology Needs for Aging in Place: Perspectives from Older Adults and Their Caregivers and Providers			All reported that they would be interested in giving residents who use wearable devices feedback during a face-to-face visit with the individual, and that they believed such devices would help them to individualize care to the older adults living in the facility. Similarly, five of the residents said that they would be interested in having a device that monitors their health for them. Overall, most of the residents (24 of 29) reported a preference that someone else would manage their health for them – someone else being either their health care provider (13 of 29) or a family member (3 of 29).				They reported that they would be interested in seeing data on the seniors' breathing, fall risk, walking distance, sleep quality, heart rate, blood oxygen level, body posture, blood pressure, weight, mood, fatigue, blood glucose, steps, hours of sleep, active minutes, calories burned, and location. Some would like for seniors to track this data for self-care, but reported that they would not be interested in reviewing such data. When asked how they would like to access seniors' data, all responded that electronic health records or downloadable files from the device would be a preferred method. Six of the total 29 individuals reported that they would be interested in tracking health information such as steps, distance, calories, hours of sleep, falls, active minutes, sleep quality, heart rate, body posture, blood glucose, and blood pressure.	Five reported that they believe the major obstacle in accessing and reusing information from wearable devices would be legal and accuracy-related.Twenty-two reported not being interested in learning how to use or wear a personal health device after being told that these are small devices to be worn on the body and that are used to track various types of health information. For those who are interested in tracking health information (6 out 29), the most common concern was the price which was reported by 4 of the 6 residents	Five said they were introduced to these devices through family or friends, and four said they were introduced to the technology through work.	All reported that they would be interested in giving residents who use wearable devices feedback during a face-to-face visit with the individual, and that they believed such devices would help them to individualize care to the older adults living in the facility. Three providers strongly agreed that these devices would help older adults age in place for a longer period of time. Four providers strongly agreed that the devices would help them identify precipitating factors that would prevent adverse events such as heart attacks, falls, etc. One person thought it might be burdensome to have to learn how to use it and another person was concerned about the technology getting wet and then not working. This same resident also reported that he/she would be interested in sharing health data collected from the wearable device with other people he knows only from the internet. The other 5 residents who reported being open to wearable devices said that they would not be interested in sharing such data.		UNDECLARED
Activity tracker and elderly: Usability and motivation of mobile healthcare in the context of elderly people	2015	Proceedings - 15th IEEE International Conference on Computer and Information Technology, CIT 2015, 14th IEEE International Conference on Ubiquitous Computing and Communications, IUCC 2015, 13th IEEE International Conference on Dependable, Autonomic and Secure Computing, DASC 2015 and 13th IEEE International Conference on Pervasive Intelligence and Computing, PICom 2015	Rasche, P. and Wille, M. and Theis, S. and Sch[illegible]er, K. and Schlick, C.M. and Mertens, A.	10.1109/CIT/IUCC/DASC/PICOM.2015.211	Wearable devices like activity tracker, measuring motion and steps, enable users to monitor their behaviour and might support a healthier lifestyle. These wearables might also motivate elderly to be active and live healthy. Unfortunately they are mostly designed for younger users and it is unclear if they are usable by older adults In order to answer this research question a four week lasting empirical study was conducted regarding individual motivation and activity trackers' usability with 15 participants older than 60 years.	Mixed	Germany	Older adults	15 participants (8 female, 7 male) having no experience with activity tracker took part in this study (see Table II). The average age was 68 years (SD = 5.29). All participants were retired in average since 12.33 years (SD = 12.64) and use a smartphone in their daily life. 11 out of 15 participants described themselves as sporty. Each participant performed in average 5.2 h (SD = 2.957) sports per week. Sport was for none of the participants a medical requirement. All were interested in measuring their daily activity (measured by a seven-likert-scale (1 = I totally agree; 7 = I totally disagree) the average answer was 1.71 (SD = 1.139). Furthermore, all participants were interested in using an activity tracker.	Activity Tracker and Elderly			and they were willing to apply this kind of product to their daily life.		Based on the interviews participants felt comfortable using an activity tracker	14 out of 15 participants reported in their final meeting an intention to use an activity tracker as therapy support due to motivational aspects (6 out of 15 participants) as well as an objective control (10 out of 15 participants).	The semi structures interviews revealed that older users prefer to use the activity tracker instead of a wristwatch. Therefore the time should be displayed continuously on the display of the activity tracker if no other data is accessed by the user. Participants also reported that it would be useful if the product is waterproof, therefore it could be worn in the shower, during swimming or aqua-fitness. The activity tracker just had one button to interact with. Participants reported this interaction design to be difficult and annoying. They had problems feeling the button under the silicon wristband. The navigation of the activity tracker display works by pushing the button. For example is it necessary to press twice to get the actual time, which was reported to be annoying. Further participants reported that the provided tracker could only be worn on the left hand. If it was worn on the right hand the button was pushed by the participants' cloths as well as the data was displayed upside down. The evaluation of the user behaviour in case of charging the activity tracker show that with increasing time participants were more and more confident in the battery life and thereby decreased the number of charging cycles as well as charged the tracker later and thereby with a lower battery status. The usage of the manual differs between the first two weeks and the second two weeks significantly (t = 3.595; df= 14; p =. 003). Participants used the manual in the first two weeks (up to three times within these two weeks) more often than in the second two weeks (just three participants used it at all). Main reasons for consultation were interest in the different functions of the activity					UNDECLARED
Impact of wearable technology on psychosocial factors of osteoarthritis management: A qualitative study	2016	BMJ Open	Belsi, A. and Papi, E. and McGregor, A.H.	10.1136/bmjopen-2015-010064	To identify the impact the use of wearable technology could have in patients with osteoarthritis in terms of communication with healthcare providers and patients' empowerment to manage their condition.	Qualitative	UK	people with osteoarthritis	21patientswithkneeosteoarthritis fromtheLondonarea (agerange45–65years) participatedinatotaloffourfocusgroups. Recruitmentcontinueduntildatasaturation.	wearable technology on psychosocial factors of osteoarthritis management			Patients'responsessuggestedapositive attitudeontheimpactwearabletechnologycouldhave onthemanagementofosteoarthritis.								thattheuseofwearabledeviceswouldbenefitpatients intermsoffeelingincontroloftheircondition, providingthemwithawarenessoftheirprogress, empoweringintermsofself-managementand improvingcommunicationwiththeirclinician. Conclusions:Thispapersuggestspositivepatient perspectivesontheperceivedbenefitswearable technologycouldhaveonthemanagementof osteoarthritis.		N

A user-centred home monitoring and self-management system for patients with heart failure: A multicentre cohort study	2015	European Heart Journal - Quality of Care and Clinical Outcomes	Rahimi, K. and Velardo, C. and Triantafyllidis, A. and Conrad, N. and Shah, S.A. and Chantler, T. and Mohseni, H. and Stoppani, E. and Moore, F. and Paton, C. and Emdin, C.A. and Ernst, J. and Tarassenko, L. and Rahimi, K. and Velardo, C. and Shah, S.A. and Moore, F. and Tarassenko, L. and Cleland, J. and Emptage, F. and Farmer, A. and Fitzpatrick, R. and Hobbs, R. and MacMahon, S. and Perkins, A. and Altmann, P. and Chandrasekaran, B. and Ernst, J. and Foley, P. and Hersch, F. and Salimi-Khorshidi, G. and Noble, J. and Woodward, M.	10.1093/ehjqcco/qcv013	We aimed to develop and evaluate a user-centred and adaptive system for health monitoring and self-management support in patients with heart failure.	Quantitative	UK	patients with heart failure	Baseline clinical and socio-demographic characteristics<br><br>Age in years, median (IQR) 77 (65–83)<br>Women, n (%) 18 (35)<br>Diabetes mellitus, n (%) 21 (40)<br>Chronic obstructive lung disease, n (%) 10 (19)<br>Moderate or severe valvular disease, n (%) 24 (46)<br>Left ventricular ejection fraction, median % (IQR) 34 (28–50)	A user-centred home monitoring and self-management system for patients with heart failure			All participants indicated that they would recommend the system to others, should it become more widely available. At the final visit, when the home monitoring system was to be retrieved, 20 participants (38%) asked whether they could keep it for self-management support after study completion.		83% of participants reported that they were using the system fully independently and the remainder required some assistance from their caregivers. 93% of participants felt that the system was easy or very easy to use.					Despite the fact that no actual medical intervention was offered in this study, 78% of participants agreed or strongly agreed to the statement that 'using the system helps me manage my health better'.		N
Personal experiences of electronic measurement of medication adherence in elderly stroke survivors	2015	Drugs and Therapy Perspectives	Chambers, J.A. and O[illegible]arroll, R.E. and Dennis, M. and Sudlow, C. and Johnston, M.	10.1007/s40267-015-0200-6	The aim of this study was to examine the practicality and accuracy of using an electronic monitoring device as a means of measuring medication adherence in elderly stroke survivors, with emphasis on patients' experiences.	Quantitative	UK	elderly stroke survivors	Fifty-eight people used the MEMS pill bottle (n = 29 in each treatment arm), and were analyzed as allocated [9]. All were white British, 64 % were male, and the mean age was 69.2 ± 10.7 years (range 51–85). Patients were of higher socioeconomic status than the average for the region (i.e. 51.7 % were from the least deprived quintile of Scottish Index of Multiple Deprivation vs. 44.1 % for Edinburgh).	electronic measurement of medication adherence in elderly stroke survivors					[illegible] researchers. Patients reported finding it large, cumbersome, top heavy and easy to knock over (this did not result in any electronic failures) .<br><br>Some patients had difficulties opening or closing the bottle, including the spring-loaded cap flying out of their hands. Pharmacy advice made it a requirement to leave pills in blister strips, as removing some medication could result in deterioration as a result of air contact. However, the blister strips were frequently too large for the pill bottle (despite using the largest available bottle), meaning they had to be cut into smaller strips.<br><br>Most patients were on multiple medications and this also posed problems for some, as it meant one of their medications was being treated differently from the others<br><br>Patients frequently expressed interest in the workings of the MEMS cap (e.g. "So the wee spring, when you take the lid off, the spring expands or something" [male aged 79 years, control group]), and quite a few commented on the potential flaws in the system as an accurate record of pill-taking<br><br>However, despite the concerns and difficulties mentioned above, only 4 % of patients did not agree that the MEMS pill bottle was easy or acceptable to use (Fig. 2), and the vast majority agreed or strongly agreed with both statements					Although it was not intended that the MEMS pill bottle would increase adherence, 31 patients (55 %) agreed that they had found the MEMS pill bottle helpful in remembering to take their tablets, whilst only 16 (29 %) disagreed that it was helpful (Fig. 2). Some patients from both groups remarked that knowing it was being measured made them more conscientious in taking their medication .Some patients reporting feeling guilty if they had missed a tablet, as the research fellow would know this from the MEMS recordings However, others expressly said that knowing it was being recorded had not made any difference to them		N
"You get reminded you're a sick person": Personal data tracking and patients with multiple chronic conditions	2015	Journal of Medical Internet Research	Ancker, J.S. and Witteman, H.O. and Hafeez, B. and Provencher, T. and Van De Graaf, M. and Wei, E.	10.2196/jmir.4209	Our aim was to explore the perspectives of individuals with multiple chronic conditions (MCC) using a semistructured interview study. Our research questions were (1) How do individuals with MCC track their own health and medical data? and (2) How do patients and providers perceive and use patient-tracked data?	Qualitative	USA	Patients With Multiple Chronic Conditions	Half of patients were men and half were women; a third (n=7) were black. Ages ranged from 37-89 (mean 64.1; median 66). About two-thirds (n=15) were not currently married. Just over a third (n=8) used English as a second language. One third (n=7) were covered by Medicare (US public insurance for those over age 65); one third (n=7) by Medicaid (US public insurance for those with low income); and the remainder (n=8) by commercial insurance.	Personal Data Tracking and Patients With Multiple Chronic Conditions						patients track for different purposes, ranging from sense-making to self-management to reporting to the doctor,					Four major themes arose from the interviews: (1) tracking this data feels like work for many patients, (2) personal medical data for individuals with chronic conditions are not simply objective facts, but instead provoke strong positive and negative emotions, value judgments, and diverse interpretations	UNDECLARED
Real-time continuous glucose monitoring in type 1 diabetes: A qualitative framework analysis of patient narratives	2015	Diabetes Care	Pickup, J.C. and Holloway, M.F. and Samsi, K.	10.2337/dc14-1855	This study analyzed narratives about experiences of real-time continuous glucose monitoring (CGM) in people with type 1 diabetes.	Qualitative	UK	diabetes patients	There were equal numbers of adults (≥ 18 years of age) with type 1 diabetes (n = 50) and caregivers of children with type 1 diabetes (n = 50), with a similar sex distribution. CGM duration did not differ between children and adults (median [range]: 1.71 [0.1–7.4] vs. 2.10 [0.1–7.5] years, respectively). Most participants used CGM in conjunction with insulin pump therapy (87%), and 71% used the sensor for 75–100% days/month. Two-thirds received partial or full NHS funding for CGM; the remainder were self-funding.	Real-time continuous glucose monitoring in type 1 diabetes			Most participants and caregivers understood and were realistic about the limitations of the system and thought that the positives outweighed the negatives. Typically, most thought that, despite the hard work involved, the "benefits were more than the sum of the parts":There were numerous comments from participants that they "loved" their CGM, that it was "wonderful" or "fabulous," "invaluable," and was "the best thing that happened to me." Many noted the effect on lifestyle—thinking that it was "life changing," or had given them or their child their "life back." One person said that it was "like her guardian angel" and that it "should be available to all who want it":					Accuracy and Reliability.<br>A minority thought CGM was generally accurate. Most remarked that reliability was inconsistent, with inaccuracy at times, incorrect alarms, and sensor failure occurring before the expected lifetime of 6–7 days (86% of those mentioning this issue, 32% of all participants). Several people also reported the need for appropriate calibration (when the blood glucose is not changing rapidly). Many acknowledged the time lag between blood glucose and sensor changes as one reason for apparent inaccuracies:<br><br>Several who wrote about CGM inaccuracy and unreliability, nevertheless, enjoyed a positive experience with improving control.<br><br>Alarms and Alerts.<br>There were several comments on the advantages of predictive alarms for hypoglycemia, but many would like the alarms to be louder, at least at night (8% of all respondents and 28% of alarm issues). There were also many negative comments about alarms being annoying, the intrusion of alarms, disturbed sleep, and a life "living by alarms" (15% of all respondents and 52% of alarm issues):<br><br>Other Technical Issues.<br>Some participants found insertion of the sensor uncomfortable, painful, or "fiddly" and reported itchiness or sensitivity to sensor mount		Reduction in stress and anxiety for both patients and caregivers was common, with many reporting a feeling of reassurance and security and being "more normal." "Peace of mind" was mentioned frequently. CGM users also reported more confidence in managing their diabetes, more freedom and independence in their lives, and improved mood, energy, and general quality of life:<br><br>All respondents who mentioned diabetes control (37% of participants) noted that CGM helped them achieve better control than with SMBG, with lower HbA1c and/or blood glucose levels. Some also noted reduced blood glucose variability:<br><br>Participants (22%) also mentioned the value of detecting or predicting hypoglycemia using CGM and a reduction in frequency and/or severity of hypoglycemic events:<br><br>There were, however, mixed reports from parents about the use of CGM at school. Most reported it could help at school, giving children more self-confidence and independence, and helping with	On the other hand, some respondents noted the stress of always being aware of diabetes results and sometimes the perceived failures to control diabetes. Some felt that CGM puts a child under more pressure and causes the child to feel different from other children. Several participants mentioned the risk of becoming obsessed with CGM data:<br><br>Some participants mentioned that alarms could disturb sleep (10% of those mentioning sleep) and that sleep could produce technical problems, notably, occasional sensor cutout or false-low values if lying on the sensor in bed.	J.C.P. has received speaker and/or consultancy fees from Medtronic, Roche, CeQur, Cellnovo, Eli Lilly, and Novo Nordisk. M.F.H. is chief adviser at INPUT Patient Advocacy, a charity supporting patients' access to diabetes technology (www.inputdiabetes.org.uk), and has received consulting fees from Dexcom and Cellnovo. No other potential conflicts of interest relevant to this article were reported.
Supporting patients to self-monitor their oral anticoagulation therapy: Recommendations based on a qualitative study of patients' experiences	2015	British Journal of General Practice	Tompson, A. and Heneghan, C. and Fitzmaurice, D. and Sutton, S. and Harrison, S. and Ward, A.	10.3399/bjgp15X685645	Clinical trials suggest that oral anticoagulation therapy (OAT) self-monitoring is safe and effective, however little is known about the patient experience of this process. To collect in-depth information about patients' experiences of OAT self-monitoring outside of clinical trial conditions and to produce a set of recommendations on how best to support such patients.	Qualitative	UK	CASM patients	[illegible] agreed to be interviewed (older and male participants were less likely to agree) Table 1 summarises their characteristics as collected at the cohort study baseline. Interviewees tended to live in less-deprived neighbourhoods (median Index of Multiple Deprivation Score 2010 of 10.4) and 73% had a professional qualification and/or a degree, generally reflecting the CASM cohort as a whole. Seven interviews were conducted over the telephone due to the interviewees' distal location from the Oxford-based research team.	patients to self-monitor their oral anticoagulation therapy			Following initial problems using the monitoring device, interviewees described a mostly positive experience.						Desire to self-manage was variable, especially when dosing advice systems worked promptly and reliably. Interviewees overcame patchy healthcare system knowledge and support of self-monitoring by educating themselves. Family and friends provided support with learning to use the monitor and managing OAT dosage adjustments.			N
Quantitative home-based assessment of Parkinson's symptoms: The SENSE-PARK feasibility and usability study	2015	BMC Neurology	C. and Santos, A.T. and Domingos, J. and Abreu, D. and Lobo, R. and Gon[illegible]lves, N. and Barra, M. and Larsen, F. and Fagerbakke, ? and Akeren, I. and Wangen, H. and Serrano, J.A. and Weber, P. and Thoms, A. and Meckler, S. and Sollinger, S. and van Uem, J. and Hobert, M.A. and Maier, K.S. and Matthew, H. and Isaacs, T. and Duffen, J. and Graessner, H. and Maetzler, W.	10.1186/s12883-015-0343-z	The feasibility and usability of an objective, continuous and relatively unobtrusive system (SENSE-PARK System), which consists of wearable sensors (three worn during the day and one worn at night), a smartphone-based App, a balance board and computer software, was tested 24/7 over 12 weeks in a study including 22 PD patients.	Quantitative	Unspecified	patients with Parkinson's symptoms	Nr or participants (male)<br>5 (3)<br>5 (3)<br>3 (0)<br>3 (2)<br>3 (3)<br>3 (3)<br><br>Age (mean ± sd)<br>60.2 (9.8)<br>60.6 (10.5)<br>59.3 (3.7)<br>53 (9.9)<br>66 (2.7)<br>60 (6.2)	home-based assessment of Parkinson's symptoms: The SENSE-PARK			All participants showed willingness to continue in the study from visit 1 to visit 8, and all of them completed the study.		Analysis of the PSSUQ scale by country showed that study participants from Norway had a slightly different profile than those from the other countries, scoring less (i.e. better) with regard to system use in items such as ability to efficiently complete tasks, comfort with using the system, and ease of learning.There were four adverse events registered, three of them being classified as definitely related to the study and one being possibly related to the study							Joaquim J. Ferreira has held consultancy functions with GlaxoSmithKline, Novartis, TEVA, Lundbeck, Solvay, Abbott, BIAL, Merck-Serono, Merz, Ipsen; has received grants from GlaxoSmithKline, Grunenthal, Fundação MSD (Portugal) and Teva; has been a member of the European Huntington Disease Network and has been employed by Centro Hospitalar Lisboa Norte, Faculdade de Medicina de Lisboa. Helen Matthews has been employed byThe Cure Parkinson's Trust. Walter Maetzler has received speaker honoraria from UCB, GlaxoSmithKline and Rölke Pharma. None of the other authors have any competing interests to report.
Patients' behavioral intentions toward using WSN based smart home healthcare systems: An empirical investigation	2015	Proceedings of the Annual Hawaii International Conference on System Sciences	Alaiad, A. and Zhou, L.	10.1109/HICSS.2015.104	Advances in Wireless Sensor Network (WSN) have opened up new opportunities for healthcare systems. WSN based smart home healthcare systems (WSN-SHHS) represent innovations in the area of sensor technology that have promised to improve healthcare quality and to stem rising healthcare costs by facilitating effective patient-medical professional collaboration and smart information sharing, and increasing patients' health observability and remote monitoring. We contextualized UTAUT to the domain of WSN-SHHS.	Quantitative	USA	General public	males accounted for a little more than half of the participants (55.4%), almost 80 percent of the participants belonged to the 18–33 years old age group, about two-fifth of the participant's annual income is less than $30000, and one-fifth of them had graduate degree and one-fifth had some college but no degree. The statistics also shows that all the participants used the Internet and the computer several times a day, more than half of the participants (68.7%) had knowledge about home healthcare, and the majority of them had knowledge about the sensor technology (71%). The diversity of the participant background is beneficial for the purpose of this study.	WSN Based Smart Home Healthcare Systems				The empirical results confirm that performance expectancy, social influence, life quality expectancy, and cost expectancy have direct effects and effort expectancy has indirect effects on patients' behavioral intention to use WSN-SHHS.								UNDECLARED
An ambient assisted living approach in designing domiciliary services combined with innovative technologies for patients with alzheimer's disease: A case study	2015	American Journal of Alzheimer's Disease and other Dementias	Cavallo, F. and Aquilano, M. and Arvati, M.	10.1177/1533317514539724	Alzheimers disease (AD) is one of the most disabling diseases to affect large numbers of elderly people worldwide. In this context, the aim of this work is to demonstrate the technical effectiveness and acceptability of an innovative domiciliary smart sensor system for providing domiciliary assistance to patients with AD which has been developed with an Ambient Assisted Living (AAL) approach.	Mixed	Unspecified	Patients with Alzheimer's disease and caregiver	phases: first, ASPeF sociomedical operators selected those patients who were in the first and second clinical stage of AD, lived alone or with a caregiver, were partially autonomous, and needed support in some of the activities of daily life (ADLs). In this phase, 80 patients were identified, providing a Mini-Mental State Examination test. Then, these patients and their relatives were asked to participate actively in the experimentation and 14 of them accepted (10 women [age 84 ± 5.31], 4 men (age 83.5 ± 5.8) of which 5 were living alone, 3 were living with spouse, and 6 were living with care worker.	An ambient assisted living approach in designing domiciliary services combined with innovative technologies for patients with Alzheimer's disease			During the experimentation, the acceptability, utility, usability, and efficacy of this system were evaluated as quite positive.		During the experimentation, the acceptability, utility, usability, and efficacy of this system were evaluated as quite positive.							N
Examining older adults?perceptions of usability and acceptability of remote monitoring systems to manage chronic heart failure	2015	Gerontology and Geriatric Medicine	Evangelista, L.S. and Moser, D.K. and Lee, J.-A. and Moore, A.A. and Ghasemzadeh, H. and Sarrafzadeh, M. and Mangione, C.M.	10.1177/2333721415618050	This study was conducted to evaluate the feasibility, usability, and acceptability of using remote monitoring systems (RMS) in monitoring health status (e.g., vital signs, symptom distress) in older adults (=55) with chronic heart failure (HF).	Mixed	USA	Older Adults with Chronic Heart Failure	[illegible] 73.1 ± 9.2 years and mean ejection fraction of 27.3 ± 6.7 participated in the study. The average time since HF diagnosis was 5.2 ± 2.1 years. A majority of participants were female (52.4%) and married (57.1%). Ethnic/racial breakdown for the sample was 19% Hispanic, 52% Caucasian, and 29% African American. A little less than a third of the participants (29%) had a high school education or less, and 42.9% of the participants reported an annual income below US$15,000. The New York Heart Association Class for patients was Class II (66.7%) and Class III (33.3%). Thirty-three percent (n = 7) reported not having a smartphone or computer at home and no previous	Older Adults' Perceptions of Usability and Acceptability of Remote Monitoring Systems to Manage Chronic Heart Failure			overall usability and acceptability of the RMS were 4.08 ± 0.63 and 4.10 ± 0.56, respectively (range 1-5).		overall usability and acceptability of the RMS were 4.08 ± 0.63 and 4.10 ± 0.56, respectively (range 1-5). Seventy-three percent reported having no difficulty or little difficulty, and 90% were moderately and extremely satisfied with using the RMS; 95% felt the self-monitoring required "very little" or "no" amount of their time (Table 3).	A majority of the patients (90%) were strongly interested in using RMS in the future; however, 71% expressed the desire to have a bigger monitor to see their health data and complete the surveys and 86% reported wanting to receive their health data in written format versus visual display on the RMS. Three of the older participants indicated that their family members helped them with daily transmission because they could not see the visual display or did not want to learn how to use the RMS; they suggested integrating an audio reporting of their health data. More than half (57%) felt that daily symptom reporting was too intensive and was the least liked aspect of the RMS experience; 9.5% did not understand the need for daily transmission of health data and associated alerts and reminders. One quarter (19%) reported the desire to have someone from the research team help set up their RMS device at home.				All participants felt the feedback they received from the health care provider related to their health data was very helpful toward assisting them with a better understanding of their heart condition and treatment plan. They appreciated having the time to discuss their options with a person who was knowledgeable and open to helping them with self-care management.		N
Health information tailoring and data privacy in a smart watch as a preventive health tool: Qualitative study of users?perceptions and attitudes	2015	Lecture Notes in Computer Science (including subseries Lecture Notes in Artificial Intelligence and Lecture Notes in Bioinformatics)	Yoon, H.S. and Shin, D.-H. and Kim, H.	10.1007/978-3-319-21006-3_51	Wearable technologies, especially smart watches are recently becoming popular and their health-related functions are well recognized. They can be effective to deliver health information to users, because they are able to track their activities. Whereas there is a privacy concern that users' personal health data could be misused according to be monitored their physical conditions via the smart watch. In this light of importance, this qualitative study explores the perceptions of smart watches as preventive health tools with 2 subjects: information tailoring and data privacy.	Mixed	Korea	General public	age: 21-41; 6M, 6F	Smart Watch as a Preventive Health Tool							information tailoring. So we reclassified users with their self-reported preference of using the information tailoring service as three types: personalization, customization and both.			In sum, most participants mentioned that they have a positive view of using smart watches as preventive health tools. Most participants (10/12) reported the potential value of the smart watches as for managing physical conditions daily. All of them mentioned usefulness and convenience of these health related functions to check their activity and conditions.	However, most participants (11/12) also expressed their worrying about health related functions. They raise various questions for inaccuracy, untrustworthiness and possibility of generalization. Power users reported various level of their concern for data privacy, but non-power users expressed rather deep concern and strong interest about their health data privacy.<br><br>Half of power users (3/6) mentioned that concern for health data privacy is an unnecessary anxiety. They focused on positive health related functions and chances to use physical data personally.<br><br>But to the contrary, all of non-power users (4/12) said that they have strong concern for exposing their personal information including health data. The results are totally opposite effects compared to our literature review which founded non-power users have lower protection of online privacy than power users [27]. The 4 participants	UNDECLARED
A knee monitoring device and the preferences of patients living with osteoarthritis: A qualitative study	2015	BMJ Open	Papi, E. and Belsi, A. and McGregor, A.H.	10.1136/bmjopen-2015-007980	To identify perspective of patients with osteoarthritis, in particular design requirements and mode of use, of wearable technology to support the rehabilitation pathway.	Qualitative	UK	patientslivingwith osteoarthritis		Akneemonitoringdeviceandthe preferencesofpatientslivingwith osteoarthritis	Onlyafewpatients(9/21)wereawareo fwhatwearable technologywasandcouldprovidevali dexamplesofsuch systems.		wearabletechnologywereappearanceandcomfort duringuse.Patientsweresupportiveoftheuseof wearabletechnologiesduringrehabilitationandcould recognisetheirbenefitsasmonitorsfortheirprogress, incentivestoadheretoexercise,andtoolsformore informedinteractionwithclinicians.									N
TELEMOLD project: Oximetry			Faria, I. and Gaspar, C. and Zamith, M. and Matias, I.		Standard assessment of long-term oxygen therapy (LTOT) prescription involves hospital-based clinical tests. The authors describe the results of a study aiming to evaluate the clinical relevance of a home telemonitoring system in LTOT optimization.				Table 1. Clinical and Epidemiological Characteristics of Patients (n=35)<br><br>CHARACTERISTIC VALUE<br>Gender<br>Male 28 (80)<br>Female 7 (20)<br>Average age (years) 64.7±10.8<br>Race<br>White 34 (97.1)<br>Black 1 (2.9)<br>School attending (years)a 7.0±4.1<br>Diagnosis<br>COPD 22 (62.9)<br>Interstitial lung disease 6 (17.1)<br>Asthma 3 (8.6)<br>Tuberculosis sequelae 3 (8.6)<br>Chest wall disease 1 (2.9)<br>Tobacco usage<br>Ex-smokers 20 (57.1)<br>Smokers 7 (20)													

Electronic bracelet and vision-enabled waist-belt for mobility of visually impaired people	2014	Assistive Technology	Bhatlawande, S. and Sunkari, A. and Mahadevappa, M. and Mukhopadhyay, J. and Biswas, M. and Das, D. and Gupta, S.	10.1080/10400435.2014.915896	A wearable assistive system is proposed to improve mobility of visually impaired people (subjects). This system was subjected to clinical evaluations with 15 totally blind subjects.	Quantitative	India	visually impaired people	All totally blind participants and primary caretakers gave their written informed consent. The age of the subjects ranged from 20 to 55 years, with a mean of 38.46 years. Three subjects were congenitally blind, and seven subjects were having acquired blindness with a history of more than 15 years (late blind subjects). Five had lost their sight approximately one year before the time of this study and had no residual vision at all. Causes of blindness were diverse: Congenital blindness, glaucoma, optic atrophy, corneal ulcer, optic nerve damage, and macular degeneration. All blind subjects had good skills on usage of white cane.	Electronic bracelet and vision-enabled waist-belt for mobility of visually impaired people			Among the participated subjects, 93.33% (60%) expressed satisfaction with information content of the EBVEWB system (combined aid).		appreciated comfort of the system. Most of the subjects appreciated use of the EBVEWB system for perception of the surrounding environment with fewer efforts as compared to a white cane alone. They were satisfied with proactive alerts and reaction time to avoid obstacles. These subjects were comfortable with the EBVEWB system. They were able to attend external acoustic signals while using this complementary aid. All 15 participating subjects expressed satisfaction with aesthetics of the EBVEWB system. The average SCU scores computed for white cane (20.93) and the combined aid (26.20) were significantly different. All subjects were satisfied with the usage training imparted to them on the EBVEWB system. Majority of subjects showed interest to continue the use of proposed aid for their mobility activities. These subjects acknowledged significant reduction in arching of the cane due to complementary use of the EBVEWB system. The EBVEWB system exploits advantages of both sonar and vision sensors. Considering functions and cost of other mobility systems such as sonic pathfinder ($1,695), Mowat sensor ($775), Miniguide ($330), LaserCane ($3,000), and so forth, the proposed EBVEWB system could be a good option as a mobility aid. The manufacturing cost of the EBVEWB system approximately is $300 per unit.							UNDECLARED
Older adults' perceptions of a neckwear health technology	2014	Proceedings of the Human Factors and Ergonomics Society	Gonzalez, E.T. and Jones, A.M. and Harley, L.R. and Burnham, D. and Choi, Y.M. and Fain, W.B. and Ghovanloo, M.	10.1177/1541931214581380	This study investigated older adults' perceptions of prototypes for a Wireless Event detection and Adherence Monitoring System (WEAMS).	Mixed	USA	older adults	7.13; 9 female, 3 male) were recruited from the Georgia Tech HomeLabparticipant database. HomeLab participantsover the age of 65 taking at least two medications were eligible for this study. Participants were compensated $60 for their participation in the 2-hour in-home research study	Older Adults' Perceptions of a Neckwear Health Technology			All participants stated that they currently use some type of wearablesystem. Nineused watches, 2 used hearing aids, and 2 used Continuous Positive Airway Pressure (CPAP)machines; 1 each used an emergency alert, pedometer, or Transcutaneous Electrical Nerve Stimulation(TENS)unit.	Moreover, this study found perceived need to be an important factor inolder adults' acceptance of the WEAMS.	Rankings for each consumer preference were as follows: functionality (median=7), reliability (6.5), usability (6), comfort (4.5), appearance (4), durability (4), portability (4) and cost (2). There wasa significant difference among consumer preferences (X2(7)=20.639, p=.004). Post-hoc analysis showed no significant differences when using the Bonferonni correction(α =.05/28=.0018). However, there are two noteworthy relationships (p<.01): functionality vs. portability (Z=-2.917, p=.004) and functionality vs. durability (Z=-2.828, p=.005)		Participants preferred prototypes with discrete design features and that were easy to put on and take off.Open-ended participant comments revealed a strong preference for the system to blend in with the user's attire and not draw unwanted attention.	Fourparticipants reported previously using a pedometer but discontinued use because it "stopped working" or they "couldn't figure out how to use it				UNDECLARED
Heart failure patients' perceptions and use of technology to manage disease symptoms	2014	Telemedicine and e-Health	Hall, A.K. and Dodd, V. and Harris, A. and McArthur, K. and Dacso, C. and Colton, L.M.	10.1089/tmj.2013.0146	This study explores patients' perceptions and current use of technology for managing heart failure symptoms (MHFS).	Qualitative	USA	Heart Failure Patients	Table 1.<br>Baseline Characteristics of Participants<br><br>VARIABLE FREQUENCY (%) MEAN SD<br>Age (years) 64.43 10.28<br>Gender<br>Male 10 (66)<br>Female 5 (33)<br>Race<br>White 9 (60)<br>Black 4 (26.7)<br>Hispanic 2 (13.3)<br>Education<br>≤High school 3 (20)<br>GED 1 (6.7)<br>High school 2 (13.3)<br>Some college 5 (33.3)<br>Associate's degree 1 (6.7)<br>Bachelor's degree 2 (13.3)<br>Graduate degree 1 (6.7)<br>Chronic diseases<br>Hypertension 11 (73.3)<br>Diabetes 3 (20)	Heart Failure Patients' Perceptions and Use of Technology to Manage Disease Symptoms	However, mean self-care scores (mean, 29.21; SD, 9.62) confirmed participants' basic understanding of proper MHFS	Participants were asked to define telemonitoring, telehealth, and telemedicine. Most participants defined "telemonitoring" but lacked knowledge of telehealth and telemedicine. Responses varied from no understanding to the following:<br><br>It means that you can submit things through the phone, submit data through the phone.<br><br>Telemonitoring means that, you know, you wear something, there's a device and somehow you connect it and communicate with somebody else.	All participants responded positively toward the general concept of using technology to aid in improving or managing their health in general and for MHFS. :<br>Health applications (apps) were positively perceived but not often downloaded or used.					Barriers to technology use included privacy concerns, mistrust of online information, low computer-use self-efficacy, low e-health literacy, and the associated financial costs.Participants were aware of the importance of daily weighing but offered reasons for not weighing such as forgetfulness, travel, broken or lack of a scale at home, time, not wanting to know their weight daily, or depending on other indicators such as edema for MHFS.		Also present were technology-related themes such as increased access to healthcare services that would facilitate more communication with doctors and sharing of real-time health information, which would provide early indicators for worsening conditions, convenience, increased knowledge, and decreased healthcare costs	Although some participants tempered this positivity with a discussion of being overwhelmed by technology and concerned about keeping up with technological advancements, others liked the ability to obtain objective data	C.D. discloses a financial interest in Blue Box Health, Inc. A.K.H., V.D., A.H., K.M., and L.M.C. declare no competing financial interests exist.
Feasibility of interactive technology for symptom monitoring in patients with fibromyalgia	2014	Pain Management Nursing	Vanderboom, C.E. and Vincent, A. and Luedtke, C.A. and Rhudy, L.M. and Bowles, K.H.	10.1016/j.pmn.2012.12.001	The purpose of this study was to determine the feasibility of technology-enhanced monitoring that engages fibromyalgia (FM) patients using a mobile device.	Quantitative	UK	patients with fibromyalgia	Table 1. Participant Characteristics (N = 20)<br><br>Empty Cell N %<br>Gender<br>Female 20 100<br>Male 0 0<br>Ethnicity<br>White (not Hispanic) 20 100<br>Other 0 0<br>Marital Status<br>Single 0 0<br>Married 11 55<br>Separated 1 5<br>Divorced 7 35<br>Widowed 1 5<br>Education<br>Some High School 1 5<br>Some College 5 25<br>Associate Degree 3 15<br>Bachelor Degree 8 40<br>Post-graduate Education 3 15	interactive technology for symptom monitoring in patients with fibromyalgia					Participants used the monitoring device an average of 5.2 days out of the 7-day study period. The average pain and fatigue levels were reported at approximately midrange, 4.6 and 5.2, respectively, out of a possible score of 10. Activity levels were quite low at 3.0 of a possible 10. Overall, most participants (80%) found the monitoring device easy to use. Participants reported that learning to operate the monitoring device was easy and that it was easy to interact with (75%). Fewer found the monitoring device easy to configure (70%).Device usability fell into two subcategories: appropriateness of data categories in the app and burden of using the device. Learning to use the device was not described as particularly problematic, but having a reference guide to refer to after the training was stated as a needed addition to the intervention.<br><br>One particular problem participants struggled with was how to make changes when the data categories or choices did not reflect what they wanted to report. In particular, adjectives and locations for pain description were limited<br><br>Participants engaged in discussion about the overall burden of using the device for symptom management. The need to develop a routine around inputting data into the device emerged as a key concept. Determining when and how to incorporate the device into daily routine in addition to stopping to think about the type and severity of symptoms experienced were both important considerations during this discussion.	Participants identified several aspects related to using the monitoring device for self-management, including (1) tracking progress over time, (2) balancing attention to symptoms, and (3) nurses reinforcing self-management. The most prevalent response was the benefit of being able to track their symptoms and progress over time.<br>In addition to monitoring their own status, participants unanimously indicated that the nurse feedback served a valuable role in reminding them to use strategies they had learned previously.	Some participants indicated a preference to use a computer or web-based application on a desktop computer over the mobile device, in part because they were already using the computer for work and other activities on a daily basis, thus it was already part of the daily workflow and so would be viewed as less obtrusive. Managing the small keyboard of the mobile device was undesirable for all but one participant.While the ability to receive support from the nurses was overall a positive thing, a need for balance between tracking progress and placing undue attention on symptoms was identified. Participants expressed dissatisfaction with the scheduled three-times-per-day check-in, in part because they viewed this as directing focus to their symptoms.			More than half indicated that using the monitoring device gave them greater control over their FM, and 65% felt it helped them to manage their condition more quickly. Sixty percent of participants indicated that using the monitoring device supported critical aspects of managing their FM. All participants felt that use of the monitoring device made it easier to inform the care team about their condition.	Technical Challenges<br>Despite participants' interest and training in using the monitoring device, several patients experienced technical challenges using the device, and a few had difficulties with the pain-monitoring app. The most common technical difficulty was in connecting with a wireless network. In two cases, the study coordinator made a home visit to assist participants making the wireless connection. For some participants, the pain monitoring application was not always specific enough to address their complaints, especially in terms of the location and type of pain. Participants would like more options for describing their pain. Patients were instructed to include this information in the comments section of the pain report as needed.	UNDECLARED
Remote health monitoring system in a rural population: Challenges and opportunities	2014	IECBES 2014, Conference Proceedings - 2014 IEEE Conference on Biomedical Engineering and Sciences: "Miri, Where Engineering in Medicine and Biology and Humanity Meet"	Minoi, J.-L. and Yeo, A.W.	10.1109/IECBES.2014.7047641	This paper discusses remote health monitoring as a potential application field in telecentres at rural areas. We present the challenges faced and opportunities with emphasis on patients with hypertension using a remote health monitoring system. We will also discuss the potential of deploying mHealth applications as a value added to the telecentres.	Mixed	Malaysia	rural residents	Eleven volunteered to participate and their ages ranging from 40 to 70 year old. The mean age is 60 year old. 7F, 4M	Remote health monitoring system in a rural population					During the participatory study, the EMH website was improved to suit the needs of the doctors, in terms of the browser compatibility, system operability and its functionality.<br><br>Based on the results of the Dimensions of Usability, deploying a remote health monitoring system for rural health care is indeed a plausible concept and is well accepted by all the stakeholders, especially from the medical doctors and the patients, however there are still limitations to the commercialised remote health monitoring system. There are seven components in the Dimension of Usability guidelines: System operability, satisfaction, easy to learn, error-tolerant, engaging, efficiency and effectiveness.					Opportunities<br>The feasibility and the acceptance of the remote BPhealth monitoring system intervention were aimed to promote healthy living and preventative care in a rural community. The success of the participatory study has a positive impact to the primary health care services at the telecentre and the community. It is believed that increased BP monitoring and management will lead to better patient health along with the reduction of complications and associated risk. Such results would hold promises for reducing costs in long term and better patient adherence to treatment. Home or telecentre-based BP measurement is an added value to health care for providing health services and clinically relevant information to the community and therefore can be a more consistent source of information to help manage hypertensive patients.<br><br>A good chance of success with a reliable remote health monitoring system would require a formation of country's policy of tele-medicine under the Federal Ministry of Health as well as a continuous designing, monitoring and evaluation at every stage of	Challenges of Remote Health Monitoring for Rural Health<br>In this sub-section, we will present the issues and challenges we faced while implementing the EHM system at a telecentre. Prior to the participatory study, ethical approval and remote health operator protocols in case of emergencies were set up. This process may take up to 3 to 6 months, depending on the availability of the ethical committee. Sometime during the pilot study, the average time taken from the start to the end of the BP reading for each patient increased to about 10 minutes, without any consultation time taken into account. This is due to the over low bandwidth connectivity at the rural telecentre. Furthermore, the existing EMH system only works on 3G and not on WIFI wireless technology. The broadband 3G connectivity at the telecentre has not been very good. The long waiting time for the patient's record to be downloaded and/or uploaded is the root predicament which causes frustration among users while using	UNDECLARED
Identifying early dehydration risk with home-based sensors during radiation treatment: A feasibility study on patients with head and neck cancer	2013	Journal of the National Cancer Institute - Monographs	Peterson, S.K. and Shinn, E.H. and Basen-engquist, K. and Demark-wahnefried, W. and Prokhorov, A.V. and Baru, C. and Krueger, I.H. and Farcas, E. and Rios, P. and Garden, A.S. and Beadle, B.M. and Lin, K. and Yan, Y. and Martch, S.L. and Patrick, K.	10.1093/jncimonographs/lgt016	CYCORE (CYberinfrastructure for COmparative effectiveness REsearch) is a software-based prototype for a user-friendly cyberinfrastructure supporting the comprehensive collection and analyses of data from multiple domains using a suite of home-based and mobile sensors. This study evaluated the feasibility of using CYCORE to address early at-home identification of dehydration risk in head and neck cancer patients undergoing radiation therapy.	Quantitative	USA	patients with head and neck cancer	Of these, 85 were eligible and were invited to participate; 50 consented (59% participation) and completed training. For nonparticipants who consented to provide their demographic and clinical data (n = 17), we observed differences compared with participants in terms of age, sex, disease site, and previous treatment, with nonparticipants more likely to be women and older (Table 1). Forty-eight (96%) patients completed the study. There were no differences between those who did and those who did not complete the study in terms of demographic or clinical variables. Of the 341 study questionnaires administered to patients, 332 (97%) were completed.	Identifying early dehydration risk with home-based sensors during radiation treatment: a feasibility study on patients with head and neck cancer			The usefulness of providing data to their clinicians during active treatment and the importance of being able to see the data themselves were rated very highly.		Responses indicated high levels of perceived importance of and satisfaction with using the sensors during RT, and high levels of ease of use and self-efficacy.					frequent objective data to health-care providers during active treatment (eg, "I think it is good for [the doctors] to get the feedback instantly, and not rely on a patient to recall exactly what's going on"); perceived usefulness of having access to one's own data (eg, "I learn a lot from the devices, [like] watching your weight or if you're having any problems"); and, perceived value of being in closer partnership with their clinicians for monitoring their care (eg, [I liked] "the convenience of knowing you have [the devices] at home, and that someone is following the data, so if the reading had not been good, I'd be contacted"). Responses also indicated that having access to these data also motivated greater vigilance toward maintaining optimal hydration and nutrition. Responses indicated that the devices were easy to use and did not present an unsustainable		UNDECLARED
Designing an intelligent health monitoring system and exploring user acceptance for the elderly	2013	Journal of Medical Systems	Tseng, K.C. and Hsu, C.-L. and Chuang, Y.-H.	10.1007/s10916-013-9967-y	This research considered the characteristics and the needs of the elderly and developed an intelligent health monitoring system for the elderly with low information literacy living in the nursing home.	Mixed	Taiwan	the elderly	there are 13 males and 19 females. Generally, sample size must be at least 30 for avoiding bias.	an intelligent health monitoring system and exploring user acceptance for the elderly			Performance expectation on user intention to use the platform is positive and significant. It implies that our designed healthcare system is helpful to the elderly, and they expect to use our system to monitor their health status and give them some suggestions or feedback.	Endeavor expectation affects user intention to use the platform. It implies that without additional endeavor or with easy learning urges the elderly toward positive intention to use our healthcare system. We considered the characteristics of the elderly in designing and developing our system and then designed a user-friendly system interface to urge the elderly to adopt our system. One main attractive property of our system is attractive and user-friendly user interface. It implies such a property can urge the elderly toward positive intention to use our healthcare system.<br><br>(iii)<br>The positive and significant impact of social influence on user intention to use the platform. That means that behavior intention of the elderly is influenced by opinions of other the elderly around. It implies that our system can be promoted and installed into other healthcare organizations if it is suitable to improve healthcare quality of the elderly.<br><br>(iv)								UNDECLARED
Quantifying the quantified self: A study on the motivation of patients to track their own health	2013	International Conference on Information Systems (ICIS 2013): Reshaping Society Through Information Systems Design	Gimpe, H. and Ni遲n, M. and G鍵litz, R.A.		We conducted an exploratory survey with 150 self-trackers and developed a Five-Factor-Framework of Self-Tracking Motivations.	Mixed	Germany	patients	The characteristics of our sample are as follows (n=150): Age ranges from 14 to 76 years with mean 34 and median 30 years. 71% of respondents are between 20 and 40 years old. 58% are male, 37% female (5% did not disclose their gender); 41% are employed, 33% students, 17% self-employed, 9% other; 53% are from Europe, 39% from North America, 8% other.	PATIENTS TO TRACK THEIR OWN HEALTH				Correlation of motivational factors with either common personality traits or demographic characteristics is generally low and ranges from -.244 to .209		the first place, 56% agreed that "they just thought they should" start self-tracking. Only 28% "have heard about QS before", 7% are influenced by "friends that started doing so as well" and 8% have been asked by their physician to start tracking some of their vital parameters or symptoms.Factor 1: Self-entertainment – motivated due to the "pleasure-bringing" aspects of self-tracking. Factor 2: Self-association – motivated by the prospect of community citizenship and selfindividualizing aspects within a community. Factor 3: Self-design – motivated by the possibilities of self-optimization . Factor 4: Self-discipline – motivated due to the self-gratification possibilities of self-tracking. Factor 5: Self-healing – motivated by the self-healing possibilities of self-						UNDECLARED
Willingness of older adults to share data and privacy concerns after exposure to unobtrusive home monitoring	2013	Gerontechnology	Boise, L. and Wild, K. and Mattek, N. and Ruhl, M. and Dodge, H.H. and Kaye, J.	10.4017/gt.2013.11.3.001.00	Older adult participants in the Intelligent Systems for Assessment of Aging Changes study (ISAAC) carried out by the Oregon Center for Aging and Technology (ORCATECH) were surveyed regarding their attitudes about unobtrusive home monitoring and computer use at baseline and after one year (n=119)	Quantitative	USA	older adults	Cognitively Intact MCI p-value<br>N 92 27<br>Mean ± SD Mean ± SD<br>Demographics<br>Age (yrs) 83 ± 5 84 ± 4 NS<br>Gender (% Women) 78% 78% NS<br>Education (yrs) 16 ± 3 15 ± 3 NS<br>Ethnicity (% Non-White) 10% 22% NS<br>Self-reported Daily Computer Use 67% 59% NS<br>Self-reported Daily Internet Use 32% 20% NS	older adults unobtrusive in-home monitoring			With respect to attitudes towards monitoring, a strong majority endorsed the concept of being monitored (Q1) and most participants reported that they did not mind being monitored if the data is useful for their doctor (Q2). However, cognitively intact volunteers were largely non-receptive to being videotaped at baseline (20% would agree to be videotaped around the house, Q4) and the percentage agreement dropped to 7% at year 1 (p=0.04). A significantly higher proportion of survey respondents with MCI reported that they did not mind being videotaped at baseline (45%, p=0.02). This percentage declined at Year 1 to 30%, but the change was non-significant.								With respect to the sharing of data, only a small proportion of respondents in both cognitive status groups reported that they did not care who has access to information about them from activity or computer monitoring (Q3). Nearly all participants in both groups reported willingness to have activity monitoring shared with family members and doctors (Q7-10). There were no significant changes in responses to these questions between baseline and year 1 among either group.<br><br>Despite the general receptiveness to being monitored, a number of participants expressed concerns about privacy and the security risks of monitoring/computer use. At baseline, about two-thirds of cognitively intact respondents reported being concerned that their information could be exploited, i.e., that it could be "given to people that don't have a right to it" (Q13) or that it could be "given to people who would use it to harm you" (Q14). The proportion of cognitively	UNDECLARED
The reliability and patient acceptability of the smarttrack device: A new electronic monitor and reminder device for metered dose inhalers	2012	Journal of Asthma	Foster, J.M. and Smith, L. and Usherwood, T. and Sawyer, S.M. and Rand, C.S. and Reddel, H.K.	10.3109/02770903.2012.684253	The SmartTrack (ST) is a new adherence monitoring device for pressurized metered-dose inhalers (pMDI), with remote upload and ringtone reminder capabilities. Our aim was to assess its reliability and patient acceptability.	Quantitative	Australia	athma patient	Age 44.6 (26–72)<br>Female gender (%) 63<br>Highest education level secondary school (%) 38	SmartTrack device: a new electronic monitor and reminder device for metered dose inhalers			Comments on patient acceptability were made by 7/8 patients (Table 3).		All patients reported that it was easy to use the ST to take their medication (mean score 5.6 (range 1–14); 1 = extremely easy, 100 = extremely difficult).							Dr. Foster has received a research grant from GlaxoSmithKline and AstraZeneca and lecture fees from GlaxoSmithKline, Pharmaceutical Society of Australia and AstraZeneca. Dr. Smith has received a research grant from Novo Nordisk and lecture fees from Pharmaceutical Society of Australia. Prof. Rand has participated in advisory boards for TEVA and received consultancy fees for TEVA and Merck. A/Prof. Reddel has participated in advisory boards for AstraZeneca and Novartis, received consultancy fees from GlaxoSmithKline, lecture fees from AstraZeneca, Getz Pharma and MSD, research grants from AstraZeneca and is participating in a data monitoring committee for AstraZeneca, GlaxoSmithKline, Merck, and Novartis. Prof. Sawyer has participated in an advisory board for Astra Zeneca. Prof. Usherwood has no potential conflicts of interest with any companies/organizations whose products or services may be discussed in this article.
The accu-chek mobile blood glucose monitoring system used under controlled conditions meets ISO 15197 standards in the hands of diabetes patients	2012	Scandinavian Journal of Clinical and Laboratory Investigation	and Jonsson, M. and S鎏es, I. and Johansson, C.B. and Delezuch, W. and Hagve, M. and Hardang, I.M.M. and Isaksson, H.S. and Ivarsson, A. and Lehto, L. and Keikkala, E. and Mattsson, N. and Ranta, J.K. and Stavelin, A. and Sudmann, A.A. and Varsi, K.	10.3109/00365513.2012.677544	The aim of this study was to evaluate the analytical quality and the ease of use of the Accu-Chek Mobile, a new glucose monitoring system designed for capillary blood testing by diabetic patients.	Quantitative	Norway	DIABETES PATIENTS	Gender Male 52<br>Female 36	The Accu-Chek Mobile blood glucose monitoring system			Finally, of the 73 who read the user guide, 66 patients (90%) were satisfied.		Ease of use of the test method<br>In the questionnaire, the patients gave a mean score of 4.8 out of 6 for the overall use of the meter, and even higher grades for certain crucial aspects of the operation such as applying blood to the test spot and reading the result on the display. None of the mean scores for the details of the operation were below 4.8. While eight patients reported that the tip cover was difficult to open, 77 patients (82%) did not experience any technical problems.							N

Gerontechnology acceptance by older Hong Kong people	2012	2012 Proceedings of the 29th International Symposium of Automation and Robotics in Construction, ISARC 2012	Chen, K. and Chan, A.H.S. and Chan, S.C.	10.4017/gt.2012.11.02.524.00	The purpose of this study was to investigate the gerontechnology acceptance by the older population in Hong Kong. Acceptance was examined in terms of attitude and usage behaviour.	Quantitative	Hong Kong, China	older adults	The participants were 1012 Hong Kong Chinese adults (256 males and 756 females) aged 55 years or older who were recruited from 17 local (Hong Kong) elderly services centres. This study considered people aged over 55 as elderly because the normal retirement age is 55 for civil servants (specifically, general disciplined services officers) in Hong Kong as well as for female civil servants in Mainland China. The largest groups in each demographic profile category represented in the sample were those aged 75–84 (41.7%), living with household members (72.7%), with primary education (35.5%), married (51.4%), retired (85.4%) and of middle economic status (81.1%). The full characteristics of the sample are shown in Table 1. The results of Kruskal–Wallis test showed that there were significant differences between age groups with respect to education levels ($\chi2$ = 264.875, df = 3, p < 0.000), marital	electronic or digital products or services that can increase independent living and social participation of older persons in relatively good health, comfort and safety.			Older adults in Hong Kong were found to use a variety of types of gerontechnology but with emphasis on basic health and home technologies.	Demographic variables The controlled demographic variables of STAM were age, gender, economic status and education level. Age was found to be negatively associated with PEOU, PU, AT and usage behaviour. However, the age effects on PU and AT were not significant when TAM-related constructs such as anxiety and facilitating conditions were included. This suggests that age directly influences the use of gerontechnology and PEOU, but its effects on PU and AT may be mediated by other factors. Ageing reduced the use of technological products, which implies that current products are not successfully inclusive. It was found that males were more likely to use gerontechnology than females, however, there were no gender differences with regard to attitudes and beliefs concerning technology. Not surprisingly, older people with higher levels of economic status and education were more likely to use gerontechnology. TAM-related constructs there was no support for the hypotheses that								UNDECLARED
Consumers' perspectives of wireless cardiac monitoring: Results of a small New Zealand telehealth project	2011	Health Care and Informatics Review Online	Honey, M.L.L. and Smith, C. and Parsons, J. and Parsons, M. and Burgess, L.		This qualitative study with seven participants aimed to understand the consumer's perspectives of using wireless cardiac monitoring by looking at the human factors involved.	Mixed	New Zealand	patients with a known history of cardiac conditions	The participants comprised two women and five men aged from 42 to 82, all with a known history of cardiac conditions. Figure 1 shows a breakdown of their past cardiac history, with several participants having co-morbidities which are why the percentages add up to more than 100. Core categories were identified from the data and these are described next using illustrative quotes from participants.	Consumers' perspectives of wireless cardiac monitoring					Skin Care Skin care, specifically for those with a hairy chest was an issue. A participant reported that the sensor would not stick to his hairy chest. He felt that he would have to maintain a shaved patch of skin on his chest. No participants reported experiencing any skin irritation or discomfort from wearing the sensor during the project. 6.4. Wireless Monitoring All participants found the wireless cardiac monitoring did not restrict their movement or them completing the assigned activities. In addition, because there were no wires, participants reported that the sensor was no problem to wear at all and they felt that it was easily concealable underneath their clothing. The sensor transmits a signal to the receiver on a mobile telephone. The placement of the mobile on the body was discussed by participants as the phone needed to be in close proximity to the sensor to receive the signal. Some participants held the phone in their hand, which was noted as not being practical in everyday living situations. Either a breast pocket or an inconspicuous holder that could be placed comfortably on the body were solutions suggested. Participants thought the amount of training required to use the equipment confidently would be significant. The equipment included the sensor being attached to the chest and the mobile readied.					Participants considered that they would feel safer being monitored. Some comments focused on the benefits of being continuously monitored. One participant remarked that "the advantages are you are monitored 24/7 by your GP". Another commented on the advantage should anything be wrong with their heart, of knowing "your ECG reading goes direct to the GP if unusual circumstances occur". Participants were also positive about the potential time savings they could make from being wirelessly monitored with comments including "it saves a lot of trouble", and "saves going in to see the doctor. One suggested that this saving in not having to go to an appointment would "ease the anxiety around going to the doctors". Participants felt that the use of teleheath would not change their relationship with their healthcare provider, other than to become more connected to them, which they discussed as a positive thing. It was considered positive, as long as sufficient training was provided to users: "If trained it would be great to use". Participants also said that given the correct training so they interpret the ECG, being able to see the recording would have enabled them to be less anxious about their health and may support them to	However, some participants felt that being able to see their cardiac rhythm on the screen could increase their anxiety and self-diagnosis	UNDECLARED
Medical technology in smart homes: Exploring the user's perspective on privacy, intimacy and trust	2011	Proceedings - International Computer Software and Applications Conference	Ziefle, M. and R[illegible]ker, C. and Holzinger, A.	10.1109/COMPSACW.2011.75	Using the questionnaire method, a total of 165 users (17-95 years) were examined regarding privacy, intimacy and trust issues for medical technology in homes.	Quantitative	Germany	elderly or disabled people	The data of N = 165 participants, 78 males and 87 females, aged between 17 and 95 years (46% female) were analyzed in this study (M = 47.9; SD = 18.7). In order to examine age effects, the sample was split into four age groups: the first group is aged between 17 and 30 years and consists of 35 persons (M = 23.3; SD = 3.7; 43% female), the second age group (N = 38) consists of males (53%) and females (47 %) of the age between 31 and 49 years (M = 41.6; SD = 7), and the third age group contains 46 respondents aged between 50 and 65 years (M = 57, SD = 4.3) with a proportion of 63% females and 27% males. Finally, the oldest age group (age group 4) includes 46 persons aged between 66 and 95 years of age (M = 72.5; SD = 7.4; 54% females). The participants were reached on different ways. Most of the younger participants were university students	video-based monitoring systems for long-term care of elderly or disabled people in smart home environments.			Being asked whether participants would be generally willing to let medical personnel monitor their home, the whole sample answered with, on average, "probably not" (M = 1.4; SD = 0.9). No gender differences were found. Both, males and females, showed the same reluctance, when answering, "probably not" (females: M = 1.5; males: M = 1.4). When looking at age, the youngest group (17–30 years) was most reluctant compared to all other age groups, though the difference was not statistically significant (Fig. 1)The last question within this section that had to be answered was whether participants would allow an intrusion within their private sphere in order to be medically supported. Overall, participants reacted quite indifferently, ranging between "probably not" and "probably" (M = 2.5; SD = 0.95). It is an interesting finding that age and gender did not show linear trends in this question (marginal significant interaction, F (3,136) = 2.2; p < 0.1). As shown in Figure 3, women would accept an intrusion within their private sphere more willingly with increasing age in contrast to men, which are more reluctant with the older they were.	The assessment was identical for both genders (M = 2.98), but was significantly modulated by age (F (3,408) = 2.4; p =. 055) (Fig. 2).							Importance of Privacy Apparently, data protection is not only an explicit requirement, but also seems to be a strong universal claim (M = 3.9 out of 4 points max., SD = 0.5). Regarding the handling of sensitive data, participants strongly agreed that medical data should only be transferred anonymously to physicians or pharmacists (M = 3.4, SD = 0.9), revealing no age and gender differences. all participants, independently of age and gender, attached great importance to password protection (M = 3.4; SD = 0.9). C. Feelings of Trust the extent of fear is quite high in all three cases. Interestingly, the fear that data could be altered or deleted as a result of system malfunctions, like sensor errors or computer viruses is most pronounced, followed by the fear of data delivery without consent and the fear of illegal access. Discretion and Avoidance of Stigmatization	UNDECLARED
Bridging two translation gaps: A new informatics research agenda for telemonitoring of chronic disease	2011	International Journal of Medical Informatics	Hardisty, A.R. and Peirce, S.C. and Preece, A. and Bolton, C.E. and Conley, E.C. and Gray, W.A. and Rana, O.F. and Yousef, Z. and Elwyn, G.	10.1016/j.ijmedinf.2011.07.002	To propose a research agenda that addresses technological and other knowledge gaps in developing telemonitoring solutions for patients with chronic diseases, with particular focus on detecting deterioration early enough to intervene effectively.	Mixed	UK	patients with the exemplar conditions and carers.	We conducted focus groups of patients with the exemplar conditions and their carers.	telemonitoring of chronic disease						There was a consensus that the main driver for telemonitoring should be clinical need.	Sensors for physiological parameters in use today are relatively cumbersome, but an emerging generation of sensors will be insensible and invisible, requiring very low power or being self-powering, and capable of collecting mobile data rather than at a fixed location. Informants suggested better indicators to improve early detection but noted that these may vary in usefulness between patients. They stressed the need for individual baselines and for using trends and multiple signals. Clinical informants valued qualitative parameters, such as changes in activity levels or appearance as sensitive indicators of deterioration. Most current telemonitoring systems use simple thresholds as the basis for triggering an alert (e.g., by a nurse inspecting the data) and there is little automated analysis of data. Some informants expressed the view that medico-legal issues were a significant concern: who takes responsibility (e.g., patient, carer, case manager, community nurse, GP, consultant or a commercial provider) for the accuracy of the detection system and for taking the correct clinical response is a key question. Informants suggested the development of protocols with hierarchies of responses and responders related to the severity of the alert.	They expressed distrust of technology and lack of computer literacy as reasons for not using telemonitoring.		awareness of the early warning signs of deterioration (prodromes).	the problem was not the identification of exacerbations but the lack of adequate responses by healthcare providers.	UNDECLARED
User experiences of mobile wellness applications in health promotion: User study of wellness diary, mobile coach and SeltRelax	2009	2009 3rd International Conference on Pervasive Computing Technologies for Healthcare - Pervasive Health 2009, PCTHealth 2009	Ahtinen, A. and Mattila, E. and V[illegible]en, A. and Hynninen, L. and Salminen, J. and Koskinen, E. and Laine, K.	10.4108/ICST.PERVASIVEHEALTH2009.6007	In this paper, we present the user experience results of three mobile wellness applications: the Wellness Diary, Mobile Coach and SeltRelax. We studied their usefulness, perceived usability, usage habits and motivational factors gathered during the first phase of the trial. The data were collected with user experience questionnaires and interviews, as well as actual usage logs from mobile applications.	Mixed	Finland	General public	The participants of our study consisted of 119 (35 males and 84 females) persons, the so called technology group ofthe Nuadu trial. The participants were employed by the city of Espoo, in Southern Finland. The average age of the participants was 45 years (range: 31-56 years). Out of the participants, 8 had vocational education or had been trained at work, 66 had vocational or polytechnic education and 45 had a Masters level or higher university degree.	The technologies consist of different Web, mobile and wearable solutions. In this paper, we present the user experience results of three mobile wellness applications: the Wellness Diary, Mobile Coach and SeltRelax	The participants had some experience in the use ofwellness technologies prior to the trial. About one third of the respondents had a heart rate monitor (HRM) available prior to the study, but only 3% ofthem used it actively. A step counter was a slightly more familiar device than the HRM for the participants. Forty-one percent of them had a step counter available prior to the study, and 11% were using it actively.				Ease-ol-Use According to the interview responses generally concerning all three applications, the opinion was that their usage was quite easy to learn (17/20). Moreover, the interviewees mentioned some difficulties in the use ofMC: "It was difficult to assess own level of progress... The communication between me and MC didn't work somehow. I didn't' learn to set daily programs either. " (female, 47) Many participants stated that having the application along all the time on phone was a benefit, i.e, it was simple to make the entries: "WD is handy as it is in a mobile phone. I just mark daily exercises, steps and weight because it is always with me." (female, 51) They gave critique towards the font size, which was considered too small, and the size of the buttons ofthe mobile phone: MC was perceived as too complicated to use by several participants. Regarding SR, some participants commented that it did not feel appropriate because it was considered monotonous or Artificial	According to 9/20 interviewees, an important motivational factor in WD was its ability to provide information on the long-term progress of different aspects of wellness, e.g. steps and weight. According to the post intervention questionnaire (Fig. 5), MC had a motivational role in wellness for a great amount of participants. According to the interview results, the participants perceived MC as a personal trainer. It allowed them to see what exercises they should do next. The interview data reveals that some participants used SR for quick relaxation in a successful way. A few participants described how they switched SR on sometimes when going to bed, and that it helped in falling asleep.		The main reason for declines in the usage of wellness applications was being in a hurry. When the participants felt very busy, they did not give attention to the usage of the applications. Another situation when the applications were not usually used was during holidays. Some participants also commented that they had enough technical gadgets in their lives even without wellness applications, and expressed their tiredness in using them The mobile device as a platform caused usability-related problems to the target user group.Some participants also said that they easily forgot to use SR, and in the appropriate situations, e.g. when sitting on the bus, the outside noise made the relaxation inconvenient.		Out of the participants who named weight control as their main goal, 78% stated that WD included appropriate functions for them. Sixty-five percent ofthe participants who had named exercise and fitness as their main target stated that WD included appropriate functions for them. Seventy-one percent ofthe participants who had named stress as their primary target stated that WD included appropriate functions for them. Out of those who named sleeping as their main target, 70% said that WD included appropriate functions. Out of those who set exercise activity and fitness level as their primary target 43% stated that MC included appropriate functions for them. Out of the respondents, who named stress control as their		UNDECLARED
Elderly persons' perception and acceptance of using wireless sensor networks to assist healthcare	2009	International Journal of Medical Informatics	Steele, R. and Lo, A. and Secombe, C. and Wong, Y.K.	10.1016/j.ijmedinf.2009.08.001	This is an exploratory study carrying out qualitative research into the perceptions, attitudes and concerns of elderly persons towards wireless sensor network (WSN) technologies in terms of their application to healthcare. This work aims to provide guidance on the dimensions and items that may be included in the development of a more in-depth questionnaire to further validate the importance of the identified factors as well as the relationships between them. This study aims to contribute to opening up a communication channel between users and researchers, informing the research community in relation to applications and functionalities that users deem as either desirable, inadequate or in need of further development.	Qualitative	Australia	older adults		Elderly persons' perception and acceptance of using wireless sensor networks to assist healthcare			Some of our exploratory findings in this study indicate for example that independence is highly valued by elderly people and hence any system or technology that can prolong that independence tends to be highly regarded, that privacy of WSN health data might not be as important as typically considered, and there are also indications that cost may be the most prominent determinant influencing an elderly person's acceptance of WSNs.	External factors Responses from the participants indicate that user acceptance of WSNs can be affected by external factors including, but not limited to, types of supports available, the type of care that the particular elderly individual is receiving, the elderly person's current housing situation as well as who is willing to subsidize the WSN system. Most participants pointed out that the availability and quality of user support is crucial in determining whether users can interact successfully with the technology. Our findings also indicated that elderly individuals with good social bonds are less likely to think that they have a need for WSNs. It has been pointed out that a WSN system, while it has the potential to extend an elderly person's time at home, may only be useful before they are needed to enter into a nursing facility.	Our findings show that the majority of the participants have had at least one negative experience with technology, and several of them cited previous incidents when they or their friends have unsuccessfully interacted with technology.		Others have also stated their desire to have a simple to understand system 4.1.4.2. Willingness to be trained Many have stated the desire for a demonstration or training to be given so that they would "understand how to use the thing [WSN system]." Others also commented on the fact that a manual should be provided so that they can refer back to it when they cannot remember what was taught during the training. It was also pointed out that the manual should be free of jargon and easy to understand. 4.1.5.1.1. Wearable sensors Most participants initially responded that they do not mind the concept of wearing sensors on their body; however several questioned how they can still be monitored when they are not wearing any clothes or accessories. Participants generally perceive wearable sensors to have similar capabilities with current panic alarm systems suggesting that panic alarms can be better accepted if they are made smaller or less obtrusive. There were also suggestions that wearable sensors should be discreet and disguised into a "watch or a ring" as most elderly persons do not want to be seen wearing a "health monitoring device."	that cost may be the most prominent determinant influencing an elderly person's acceptance of WSNs.			Several participants have stated that they are apprehensive towards technology and demonstrated clear concern about the possibility of having to interact with a WSN system. The majority of the participants emphasized on the fact that computer systems are hard to understand due to their old age. Many have even stated that they are "terrified of a computer." Technology has no brains. And that's the scary thing about it. Because you have to be so precise using it and it can't make up… if you make the wrong choice, the wrong button…A majority of the participants showed no concern about having their health information transmitted wirelessly. One participant even stated, "My Life is an open book" and do not perceive any concerns regarding the confidentiality of his health information as long as a camera is not used to monitor him. Participants place their preference on ensuring that help is readily available over privacy or the confidentiality of their health information.	N
Assessment of sensorized garments as a flexible support to self-administered post-stroke physical rehabilitation	2009	European Journal of Physical and Rehabilitation Medicine	Giorgino, T. and Tormene, P. and Maggioni, G. and Capozzi, D. and Quaglini, S. and Pistarini, C.		The aim of this study was to provide a description of a newly-developed remote rehabilitation system that can be employed both at home and in the hospital, supporting motor rehabilitation for post-stroke patients with upperlimb impairment.	Mixed	Italy	stoke patients	Eligible patients were: 1) hemiplegic patients in stable clinical condition, i.e. post-stroke patients not affected by any metabolic, respiratory or cardiac insufficiency that could not be controlled by adequate therapy; 2) patients with mild motor and/or cognitive impairment (this criterion is essential if we want a patient to use the system alone, or with limited assistance from his/her caregiver). The severity of motor impairment was evaluated by the physician. The estimation of the cognitive impairment was done on the base of the Mini Mental State Examination (MMSE) index,18assuming the conventional cut-off value of 23, above which the impairment is considered sufficiently mild with respect to the possible use of the system. The physician identified the candidate patients and communicated the enrolment to the physiotherapy/speech therapy responsible. Patients and their	sensorized garments as a flexible support to self-administered post-stroke physical rehabilitation			The majority of the patients were satisfied with the system, considered it useful, and would use it at home.									UNDECLARED
Elderly comfort and compliance to modern telemedicine system at home	2008	Proceedings of the 2nd International Conference on Pervasive Computing Technologies for Healthcare 2008, PervasiveHealth	Jasemian, Y.	10.1109/PCTHEALTH.2008.4571027	The aims of the present paper are to investigate, verify and evaluate the elderly heart patients' (EHP) compliance, trust and comfort in relation to a real-time wireless telemedicine system at home.	Quantitative	Denmark	Elderly	Twenty four non risky elderly heart patients, aged (60±5) years (12 males and 12 females), were included.	Elderly comfort and compliance to modern telemedicine system at home	More than 60 % of the elderly are used to employ a mobile phone in daily life and 50 % have a reasonable understanding of the system application.		Only 12 % of the elderly patients do not trust the present wireless remote monitoring system at all, whereas 72 % trust the system.		In average, 80 % of the elderly scored the user-friendly and usability of the system as good. The majority (92 %) could easily manage employing the system. Only 16 % of the elderly sought help from the healthcare personnel in relation to employment of the patient-unit, and few (4 % - 8%) had problem with changing or re-charging batteries for the mobile phone and ECG device as well. 76 % of the elderly (n = 24) scored the reliability of the system as "reasonable", 20 % as "only now and then", and only 4 % scored the reliability as "excellent". The results are summarised in table 1.80 % believe that their comfort is satisfied.						The majority of the elderly believed that their expectation to privacy was in 84 % fulfilled. And only 4% believed that their expectation to privacy was only now and then fulfilled.	UNDECLARED
Patients' perceptions of electronic monitoring devices affect medication adherence in hypertensive African Americans	2008	Annals of Pharmacotherapy	Schoenthaler, A. and Ogedegbe, G.	10.1345/aph.1K640	Electronic monitoring devices (EMDs) are regarded as the gold standard for assessing medication adherence in clinical research. To assess patients' perceptions of EMDs, their acceptance of EMDs, and the relationship of these perceptions to medication adherence in African Americans with hypertension who are followed in community-based practices.	Quantitative	USA	Patients with hypertension	able1.PatientCharacteristics"Characteristicn(%)Age, y (mean ± SO)53.44±11.73Female113 (82)Marital statussingle61 (44)married26 (19)separated41 (30)widower10 (7)Educationelementaryschool30(22)high school/GEO65 (47)some college43 (31)Employmentstatusunemployed108 (78)Type of insuranceMedicare19 (14)Medicaid101 (73)Annual income,$$20,000113 (82)>20,00025 (18)Comorbidconditions026 (19)1-247 (34);i365 (47)SBP, mm Hg (mean ± SO)144.1 ± 19.2OBP, mm Hg (mean ± SO)86.6 ± 11.4	Patients' perceptions of electronic monitoring devices affect medication adherence in hypertensive African Americans					a majority also agreeing thatthey liked the MEMS and used it every day.Alternatively,approximatelyone-third of patients preferred using a pill-boxand one-quarter stated that they did not like travelingwith the MEMS. A minority of patients reported havingProblemsusing the MEMS, such as difficulty opening thetop, or that they found itstressful.,those who reported thatthe MEMS was too bulky and inconvenient were less ad-herent than were those who added no additional com-ment							UNDECLARED
Senior residents' perceived need of and preferences for "smart home" sensor technologies	2008	International Journal of Technology Assessment in Health Care	Demiris, G. and Hensel, B.K. and Skubic, M. and Rantz, M.	10.1017/S0266462307080154	The goal of meeting the desire of older adults to remain independent in their home setting while controlling healthcare costs has led to the conceptualization of "smart homes."	Qualitative	USA	Older adults	A total of fourteen older adults over the age of 65 participated in three focus group sessions (four in the first, three in the second, and seven in the third session). Each session lasted approximately 1 hour (the average length was 64 minutes). Five participants were male and nine were female. All participants were older than 65 years of age.	Senior residents' perceived need of and preferences for "smart home" sensor technologies bed sensor			The bed sensor was perceived overall as useful. The sensor mat was also perceived as useful by most participants. The motion sensor was perceived as an application that could provide ease of mind. Participants focused primarily on the usefulness of such a device in detecting intruders and thus providing additional security, more so than on its usefulness in monitoring their activity levels. The gait monitor was perceived overall as very useful, as most participants expressed concerns about falling and being helpless or not detected in a timely manner. Finally, the video sensor was seen as beneficial in detecting health emergencies, particularly falls, but raised greater privacy concerns than the other technologies. Ten participants stated that they would not want to have a video sensor installed in their residence.			Their comments emphasized the technologies' function of emergency detection rather than their function of proactive moni-toring	For some participants, the size of the device played a role. One participant made a comment about the motion sensor: "I like this one the best; it's so small, really not intrusive . . . ." Other participants determined the usefulness of the devices based on previous personal adverse health events. One participant stated, "If you had told me 2 months ago [about these technologies] I'd say who needs it, but after what I have been through, I see the benefits." This participant had experienced a fall in the apartment and had felt helpless until someone discovered the event. Participants also addressed the issue of integration of the technology in the residence and its degree of visibility to visitors. One participant stated, "If you can hide them and you don't really see them but you know that they are there, that would be my preference."			Finally, the video sensor was seen as beneficial in detecting health emergencies, particularly falls,	other technologies. Ten participants stated that they would not want to have a video sensor installed in their residence.Some participants addressed the issue of stigmatization resulting from installing the technology. Two participants expressed concerns about the accuracy of the devices. One of them specifically addressed the issue of false alarms that could prove burdensome to both residents and facility staff members. Other participants, however, expressed the need to balance safety with privacy and that they would be willing to allow others to monitor their activity levels if that provided an added layer of safety and enhanced their well-being.	UNDECLARED
The role of continuous glucose monitoring in clinical decision-[illegible]	[illegible]	Australian and New Zealand Journal of Obstetrics and [illegible]	Mclachlan, K. and Jenkins, [illegible]	10.1111/j.1479-[illegible]	The continuous glucose monitoring system (CGMS) is a novel tool to assess 24-h glucose fluctuations. To assess the usefulness of CGMS in pregnant women with diabetes for medical decision-making and to evaluate patient tolerability and perception of usefulness.	[illegible]	[illegible]	[illegible]	Sixty-eightstudies were completed in 55 women. Four studies were ofpoor quality, with < 75% of the 72-h trace complete; two ofthese were deemed not of sufficient quality to assess, theother two were adequate. The mean trace duration was 97%,with a median 100% complete. The mean absolute differencebetween the subjects' finger-prick glucose levels and theCGMS [illegible]	continuous glucose monitoring in clinical decision-making in diabetes [illegible]			The CGMS was generally morehelpful in subjects with type 1 diabetes, where 16 of 18(89%) studies yielded additional information, compared to 20 of 36 (56%) in GDM and eight of 14 (57%) type 2diabetes subject studies.The subject feedback regarding the CGMS study wasgenerally positive, with 37 of 48 (77%) respondents feelingthat [illegible]		Forty-four of 48 subjects (92%) reported that the CGMSdevice was either 'very easy to use' or 'easy to use' (asopposed to 'a little difficult' and 'very difficult'). The level ofinconvenience was rated as 'minimal' or 'minor' by 39 of [illegible]					Forty-three of 48 subjects (90%)reported that their understanding of how they could controlblood glucose was either 'clearly better' or 'better' after usingthe CGMS (as opposed to 'the [illegible]		

Does fetal heart rate count? Developing a low cost, alternative powered Doppler fetal heart monitor for use in low resource high mortality settings	2006	IET Seminar Digest	Bezuidenhout, H. and Woods, D. and Wyatt, J. and Lawn, J.	10.1049/ic.2006.0673	To develop a Doppler fetal heart monitor which is accurate and reliable but has alternative options for power supply (wind-up, electric, solar), is robust and is affordable for use in the settings where most women and children die. To compare the reliability of the prototype with a cardiotocograph, assess if the prototype is appropriate for use in low resource settings and if it is acceptable to health workers and patients.	Quantitative	South Africa	fetus and mothers		powered doppler fetal heart monitor for use in low resource high mortality settings			A survey of 97 mothers performed at another labour ward in a secondary hospital showed that 72 mothers (74%) preferred the prototype Doppler fetal heart rate monitor to a cardiotocograph or fetoscope to monitor their baby. All the staff at the clinics found the prototype accurate and useful in the assessment of their patients and appropriate for use in their setting. The prototype was also acceptable to the patients.									UNDECLARED
Using wireless sensor networks for aged care: The patient's perspective	2006	2006 Pervasive Health Conference and Workshops, PervasiveHealth	Steele, R. and Secombe, C. and Brookes, W.	10.1109/PCTHEALTH.2006.361661	This paper presents the findings of a qualitative study on the perceptions and thoughts of elderly people on the use of current sensor network technology for assisted aged care.	Qualitative	Australia	elderly people		wireless sensor network			Overall, the elderly perceive sensor networks as a workable solution to the current health care situation.				[illegible] ambient or wearable monitoring. Their main rationale for this decision is that they will not have to actively remember it, it has the potential to continue working outdoors, and other people will not know that they are wearing it.<br>The elderly expressed a strong desire for some level of control over the system. The level of control may be limited, but they wanted to be able to feel some control over it, or to at least to have the illusion of control.<br>In order to obtain the small level of control that the elderly desire, they must be able to interact with the sensor system in some fashion. This interaction method must be simple, such as the use of a single button or switch.<br>The elderly believe that this technology should be for emergencies only. They strongly reject any attempt to incorporate social aspects into the technology. The participants believed that the system should have one function and one function only - to call for help in the event of an emergency.<br>The elderly strongly rejected the use of any camera, video or still, in	The elderly perceive cost to be the most significant factor in their acceptance and potential take-up of the technology. This was the most central and prominent issue to them, and they expressed a strong desire for the cost of the system to be offset by either family or government subsidisation.			The elderly did not perceive privacy to be a significant concern. They believed their medical data to contain nothing of value to any malicious third party.	UNDECLARED
Exploring the benefits of using motes to monitor health: An acceptance survey	2006	Proceedings of the International Conference on Networking, International Conference on Systems and International Conference on Mobile Communications and Learning Technologies,ICN/ICONS/MCL'06	Lubrin, E. and Lawrence, E. and Zmijewska, A. and Felix Navarro, K. and Culjak, G.	10.1109/ICNICONSMCL.2006.94	This paper reports on the findings of a second anonymous web survey of over 100 participants from Australia, Europe and North America, aimed at investigating the possible acceptance of Motes as a reliable and efficient health monitoring tool.	Mixed	Europe, Australia, Canada and the United States.	General public		Using Motes to Monitor Health	Seventy five people who completed the second survey had not heard of Motes before, twenty five had heard about them whilst three had completed the first survey so did not have to fill it in all again. The vast majority of respondents had not heard of Motes before undertaking this survey.	Of the people who had heard about motes before, twenty (20) were male and five (5) were female. In this group eight (8) classified themselves as students, ten (10) as engineering professionals, five (5) as academics and one (1) as medical. In the cohort that had not heard about Motes before thirty four (34) were male and thirty eight (38) female. This is in marked contrast with our preliminary survey when females were so significantly under-represented that we were not able to draw statistical conclusions for them. The breakdown of professional status for this group was as follows: twenty six (26) students, nine (9) engineers, twenty six (26) academics and eleven (11) medical. For the three people completing the survey for a second time, two (2) were males and one (1) was a female - the female identified herself as an engineer, the males selected engineering and academic for their professional identification.					One major issue identified from the survey was the fact that many people needed more information. one respondent advised us to have a timer or better numbering system to allow people o know how much time was needed to complete the survey. We had advised 5–10 minutes as a suitable time.<br>One participant mentioned that motes 'have a long way to go to be a practical solution in the healthcare environment'. This means that, at the moment, these sensors are not entirely suitable for monitoring an individual's vital signs. However, it is our contention that the main advantage of Motes is the fact that commodity based hardware and open source software such as our MRTG will make the development of healthcare monitoring a lot cheaper in the future.<br>Another survey participant stated that using motes for healthcare 'is an interesting and unexplored issue' but also warned us that we might be putting the cart before the horse. He suggested undertaking some pilot studies using motes in a healthcare environment and publishing these results before running an acceptance survey.<br>The management of large numbers of motes also present challenges such as maintenance, changing of batteries and dealing securely and efficiently with the high load of data being beamed from the motes to the data capture device.				Another participant advised us to mention privacy concerns as this person felt that such issues would influence the decision to use motes in a healthcare environment.	UNDECLARED
To track or not to track: User reactions to concepts in longitudinal health monitoring	2006	Journal of Medical Internet Research	Beaudin, J.S. and Intille, S.S. and Morris, M.E.	10.2196/jmir.8.4.e29	The aim of the study was to elicit specific feedback from health professionals and laypeople about how they might use longitudinal health monitoring data for proactive health and well-being.	Qualitative	USA	General public	Included were 15 women and 6 men, ages 40 to 66 years (mean = 51; SD = 9). Although our target demographic was people in middle life, we additionally interviewed five individuals over age 70 for comparison. Three participants were close affiliates of one of the authors. Seven participants were from the local area of Cambridge/Boston, MA. The remaining participants were interviewed over the phone and represented 10 states.	longitudinal health monitoring						While sorting, participants sometimes expressed personal reasons for selecting a construct.<br>The second sorting exercise, the "investigation sort," offered participants an opportunity to confirm or modify their tracking selections according to a more concrete tracking investigation. Physical activity and stress were the most frequently selected investigations.	While more traditional clinical metrics (blood pressure, heart rate, blood sugar, hormone levels) are represented, there are also many constructs related to the quality of activity (multitasking, variation from routine), mental states (short-term memory, ability to concentrate, laughing), and behaviors (snacking, idle time, bed time).	I wouldn't want to track (a variable or in general) because tracking would…<br>Not apply to me: (eg, smoking, alcohol drinking, pets) (n = 26)<br>Not provide new information: (ie, "I already know this") (n = 22)<br>Not provide valuable information<br>Provide too much information (information overload): Too focused on minute details, missing the big picture (n = 8)		Tracking may be useful for…<br>Supporting behavior change:<br>Making patterns more evident<br>Monitoring health<br>Challenging or validating beliefs<br>Providing a record of events<br>Providing entertainment/Supporting social interaction:	general) because tracking would…<br>Not apply to me: (eg, smoking, alcohol drinking, pets) (n = 26)<br>Not provide new information: (ie, "I already know this") (n = 22)<br>Not provide valuable information<br>Provide too much information (information overload): Too focused on minute details, missing the big picture (n = 8)<br>Threaten self-image:<br>Not provide actionable information<br>Lead to social conflict<br>Promote obsessive or unhealthy reactions<br>Force too much structure: Approaching life too analytically (n = 6); negative feelings about technology aspect (n = 3); reducing things of personal importance to a number (n = 1)<br>Not be suitable for particular activity or behavior	N
Patient experience with, and use of, an electronic monitoring system to assess vaccination responses	2006	Health Expectations	Olmsted, S.S. and Grabenstein, J.D. and Jain, A.K. and Lurie, N.	10.1111/j.1369-7625.2006.00378.x	To evaluate the user experience and acceptability of an electronic patient monitoring system.	Quantitative	USA	Military and civilian personnel	Age (years)<br><26 11 (3)26–35 56 (16) 36–45 184 (51) >45 108 (30)<br>Female 41 (11)Male 318 (89)<br>White 287 (81)Black 45 (13)Other 21 (6)<br>Enlisted 68 (20)Officer 259 (74)Civilian 21 (6)<br>HS or Associates degree 50 (14)Bachelors degree 72 (20)Masters degree or higher 236 (66)	an electronic monitoring system to assess vaccination responses			Most of the vaccinees (144/172; 84%) reported being comfortable with the idea of having a physician track their reaction to the vaccine using their electronic reports, and 67% (116/172) reported being comfortable having their take determined using the electronic reports. However, only 51% (87/172) reported that they were comfortable eliminating the actual take check follow-up visit with their health-care provider if their electronic reports indicated they had a positive vaccine reaction.	older respondents, civilians and whites (compared with other races) were more likely than others to use the electronic monitoring system at least once; however, none of these demographic characteristics achieved statistical significance (Table 3).model indicates that older vaccinees were more likely than younger (P < 0.01), and whites were more likely than African Americans (P < 0.03), to use the system frequently (Table 3).	The electronic system was well received by the vaccinees. Survey responders reported that the system was fast and easy to use (Table 2). 34% (58/171) of respondents who used the system reported using the telephone to make at least one report and 84% (147/175) reported using the Internet at least once. Both the Internet and telephone methods of making reports were reported as being easy. No users reported that the system took too long to make reports. Users also reported that the picture on the web or pocket card closely matched the appearance of their vaccine site (143/169; 85%) and that they were confident that what they reported matched their true reaction to the vaccine (147/171; 86%).			The mean number of reports made by the 822 vaccinees over the 28-day period was 7.4 (±6.6). On the survey, vaccinees reported a variety of reasons for not making daily reports: they forgot (52/137; 38%), they did not have access to a computer or phone (23/137; 17%), they did not have the phone number or web site with them (22/137; 16%), they did not have any abnormal symptoms to report (18/137; 13%) or their symptoms did not change from their previous report (14/137; 10%). Daily reminders (40/124; 32%) and being able to enter 'no changes since the last report' (35/124; 28%) were suggested as ways to increase reporting frequency.				UNDECLARED
Social Reflections on Fitness Tracking Data: A Study with Families in Low-SES Neighborhoods - Proceedings of the 2019 CHI Conference on Human Factors in Computing Systems	2019		Saksono, Herman and Castaneda-Sceppa, Carmen and Hoffman, Jessica and Seif El-Nasr, Magy and Morris, Vivien and Parker, Andrea G.	10.1145/3290605.3300543	In this 2-month qualitative study, we investigated physical activity (PA) tracking practices amongst 14 families living in low-income neighborhoods, where obesity is prevalent. We characterize how social discussions of PA data rarely extended beyond the early stages of experiential learning, thus limiting the utility of PA trackers.	Qualitative	USA	Families in Low-SES Neighborhoods where obesity is prevalent.	children from 14 families. Caregivers' median age was 36 years (IQR=16.5), 10 were female. Child participants' median age was 7 years (IQR=3), 13 were female. Fourteen caregivers were African-American and three were Hispanic. 13 caregivers were single. The median family size was 3.5 with a median 2 children. The median household income was US $21,979 per year or less. Most families qualified for the state's need-based health insurance program (n=12). Most caregivers' highest educational level was some college or vocational training (n=7). Participants lived in low-income neighborhoods (n=9) or government-	Fitness Tracking Data				We characterize how social discussions of PA data rarely extended beyond the early stages of experiential learning, thus limiting the utility of PA trackers. Caregivers and children rarely analyzed their experiences to derive insights about the meaning of their PA data for their wellbeing. Those who engaged in these higher-order learning processes were often influenced by parenting beliefs shaped by personal health experiences.								UNDECLARED
Patient-Oriented Support Roles of Self-Management Systems - Proceedings of the 30th European Conference on Cognitive Ergonomics	2012		Laverman, Martin and van der Boog, Paul and Jansen, Yvonne and Schonk, Bertie J. H. M. and Alpay, Laurence and Neerincx, Mark A.	10.1145/2448136.2448167	A focus group was conducted with eight patients with a chronic kidney disease. The role of self-management systems in their daily care was discussed, guided by a scenario that was based on a framework covering functionalities in such systems from a medical point of view.	Qualitative	Netherlands	patient	physician from the Bronovo Hospital, The Hague. Inclusion criteria were: a chronic kidney disease (CKD) (stage 1-3), diagnosed less than two years ago, experience with self-management and internet, and an ability to express themselves well in Dutch. Eight patients with an average age of 52 (range 31-67) participated in the focus group interview. The interview was led by two of the authors (ML and YJ). First, experiences with and opinionsabout self-management and support through Information and Communication Technology (ICT) systems were discussed. After a short break, the scenario was presented to anddiscussed with the participants.	These systems can range in functionalities from registering and sharing self-measurements with physicians to more complex systems that educate patients, enable online communication with professional caregivers and fellow patients, support goal-setting, monitoring and evaluating patients' health status).			It was also pointed out that the manual should be free of jargon and easy to understand.		However, our participants emphasized that it is not always easy to keep up with this. "This shows that it isnot always practical to use these systems on a daily basis.	The participants especially stressed the importance of self-measurements and feedback from professionals. Participants also emphasized that feedback can be very motivating for them and that self-measuring in itself can provide feedbackPatients that need to change their life style to manage their chronic condition, have to incorporate new routines in their daily life. Next to monitoring health status and receiving feedback, searching for information and interacting with fellow patients is an important part of self-management from a medical perspective.		I would like to get information, but I want to know ifand how much it helps, that could make it tangible and motivating.				UNDECLARED
Designing a Wellness Self-Management Tool for Older Adults: Results from a Field Trial of YourWellness - Proceedings of the 8th International Conference on Pervasive Computing Technologies for Healthcare	2014		Doyle, Julie and Walsh, Lorcan and Sassu, Antonella and McDonagh, Teresa	10.4108/icst.pervasivehealth.2014.254950	This paper reports findings from a 5-month home deployment of YourWellness -- an application that supports older adults in self-reporting on their wellbeing and provides feedback to promote positive wellbeing management.	Quantitative	Ireland	older adults	P ID G Age Own iPad SUS Int / Enj Perc Comp Pres / Tens Val / Use 59 M 67 N 90 4.7 6.8 1 6.25 60 F 69 N 75 5 6 1 6.75 64 M 77 Y 60 5.2 5.4 1.4 5 65 M 75 Y 90 6 7 3.4 7 66 M 68 N 84 4.8 7 1 6.5 67 F 65 Y 93 7 7 1 7 69 M 73 Y 93 5.5 6.4 2.6 7 Avg 70.6 83.6 5.5 6.5 1.6 6.5	a wellness self-management tool for older adults					The average score across all participants is 83.6, with a range from 60 to 93 indicating that participants found the application usable. Only two participants (59 and 65) understood the concept of the traffic light metaphor – i.e. the use of green to indicate positive wellbeing, orange to indicate medium etc. All participants understood the graph and the advice messages and found this information very useful. Perceived Competence Perceived competence within the IMI relates to an individual's perception of how capable they are at performing a certain task. In our study, this refers to the older adult's perception of how well they can use the YourWellness application. The mean perceived competence was 6.5 out of 7 (standard deviation 0.6). 5.3.3Pressure/Tension The pressure/tension subscale assesses the level of tension a person feels while performing a certain task and is a negative predictor of intrinsic motivation. The mean score for pressure/tension was 1.6 (standard deviation 0.97), where a score of 1 is a positive score, indicating that users felt quite relaxed while using YourWellness.	Enjoyment Interest and enjoyment are the most integral factors of intrinsic motivation. The mean interest and enjoyment score was 5.5 (standard deviation 0.8), where 7 is the highest possible score.<br>5.3.4Value/Usefulness A person's perception of the value or usefulness of using an application such as YourWellness can act as a significant motivator for them to not only initially adopt it, but also to continue using it. The mean value/usefulness score was 6.5 out of 7 (standard deviation 0.7), indicating that our participants would find it useful to continue using YourWellness in the future.	With regard to negative aspects, most responses related to the repetitiveness of questions (5), the inclusion of parameters of wellbeing that the individual wasn't necessarily interested in (3), and the lack of facility to 'go back' and change your answer when answering questions (2)					UNDECLARED
MoodWings: A Wearable Biofeedback Device for Real-Time Stress Intervention - Proceedings of the 6th International Conference on PErvasive Technologies Related to Assistive Environments	2013		MacLean, Diana and Roseway, Asta and Czerwinski, Mary	10.1145/2504335.2504406	We present MoodWings: a wearable butterfly that mirrors a user's real-time stress state through actuated wing motion. We designed MoodWings to function both as an early-stress-warning system as well as a physical interface through which users could manipulate their affective state. Accordingly, we hypothesized that MoodWings would help users both calm down and perform better during stressful tasks. We tested our hypotheses on a common stressful task: driving.	Mixed	USA	driver	To test these hypotheses, we designed a within-subjects study with n=11 (3 female, 8 male) participants.	a wearable biofeedback device for real-time stress intervention			Despite MoodWings having the opposite-from-expected effect, users received the idea of an actuated butterfly very positively.		Figure 5 illustrates the responses on a 7-point Likert scale (1=strongly disagree, 7=strongly agree), which were strongly polarized (mean=3.5) about how comfortable they would be.					Most users agreed (mean=4.7 on a 7-point Likert scale, with 1=strongly disagree and 7=strongly agree) that they would find an external manifestation of their real-time stress state useful.		UNDECLARED
Patient Acceptance of Blood Pressure Self-Measurement Equipment Using Context-Aware Patient Guidance - Proceedings of the 12th EAI International Conference on Pervasive Computing Technologies for Healthcare	2018		Lindahl, Camilla and Wagner, Stefan and Uldbjerg, Niels and Schl\"{u}tter, Jacob M. and Bertelsen, Olav W. and S and ager, Puk	10.1145/3240925.3240963	The aim of this study was to evaluate patient acceptance levels when performing self-measurements using a context-aware patient guidance system as part of a blood pressure self-measurement (BPSM) station.	Quantitative	Denmark	pregnant women	ParameterValue:Mean,[95%confidenceinterval]Age (years)29.9,[28.9 –30.8]Gestational age (weeks)12+4,[12+3–12+5]Systolic BP, mm Hg109,[107.5 –110.9]Diastolic BP, mm Hg72.3,[70.6 –73.9]Pulse, beats per minute 79.1,[77.0 –81.3]Mean Arterial Pressure84.6,[83.0 –86.1]	lood Pressure Self-Measurement Equipment			The questionnaire showed that 95% felt 'very comfortable' with self-measurement, 81% strongly agreed to this, whilst 14% partly agreed. Seventeen percent felt 'uncomfortable' having to self-measure, but only 7% expressed strongly agreeing to this. Ninety-three percent disagreed that they felt unnecessarily monitored by the BP equipment, 87% strongly disagreeing to this. Eight percent would have preferred that BP measurements were performed by healthcarepersonne		Ninety-two of the 100 participants experienced self-measurement as being reliable.							UNDECLARED
"But, I Don't Take Steps": Examining the Inaccessibility of Fitness Trackers for Wheelchair Athletes - Proceedings of the 17th International ACM SIGACCESS Conference on Computers & Accessibility	2015		Carrington, Patrick and Chang, Kevin and Mentis, Helena and Hurst, Amy	10.1145/2700648.2809845	We interviewed five wheelchair athletes and three physical and occupational therapists to explore fitness activities, experience with wearable technology, and potential uses for wearable fitness devices.	Qualitative	USA	Wheelchair Athletes	P#Age/SexTime using WheelchairSportsTime in Primary SportP140/F6 yearsHC2+ yearsP2*28/M24+ yearsR<1 yearP357/M37 yearsB, R35 yearsP424/M24 yearsB, T9 yearsP520/M5 yearsHC3 years	Examining the Inaccessibility of Fitness Trackers for Wheelchair Athletes	We asked participants whether they had previously owned or used a wearable fitness device. None of the wheelchair users we interviewed had experience using wearable devices.					Four of our wheelchair users were interested in tracking their personal physical activity. P2P3 was not interested in tracking his own physical activity or using a wearable device for himself. However, he did mention potential benefits for some rugby players.	1)Aesthetics and Device Visibility (V) – The physical appearance of the device and its' visual appeal. This also includes aspects that make the device visible or recognizable from afar. 2)Sensor Hardware (S) – The sensors used in wearable devices to capture information about the user. Combinations of these sensors can be used to produce more detailed information about the user. 3)Analysis of Sensor Data (A) – The method in which raw data is converted into useful information. This refers to how sensor data is actually processed as well as how users think data is processed. 4)Fit and Ergonomics (E) – The intended location and fit on the body for the wearable device. Some systems are more flexible than others with their location. Others are restricted to a certain location either by the form factor or the sensor. 5)Presentation and Feedback (F) – The way in which sensor data is presented and how the user receives and utilizes feedback from the system regarding their activities. Some people prefer devices that look more high-tech, while others are more fashion oriented. Also the overall visibility, whether you can see the device at all, is an important feature. Our participants were generally indifferent regarding specific visual features for wearable fitness devices. Three of the four participants described a preference for devices that were low-profile or inconspicuous. Our participants did not desire a particular aesthetic, however there were some general considerations that were very important. The primary consideration is that the device should	The reasons that were given as to why they had not used one were prohibitive cost, uncertainty of capabilities, misperception of capabilities, and general non-interest.				UNDECLARED
SugarMate: Non-Intrusive Blood Glucose Monitoring with Smartphones	2017	Proc. ACM Interact. Mob. Wearable Ubiquitous Technol.	Gu, Weixi and Zhou, Yuxun and Zhou, Zimu and Liu, Xi and Zou, Han and Zhang, Pei and Spanos, Costas J. and Zhang, Lin	10.1145/3130919	We evaluate SugarMate, a first smartphone-based blood glucose inference system as a temporary alternative to continuous blood glucose monitors (CGM) when they are uncomfortable or inconvenient to wear.	Mixed	China	DIABETES PATIENTS		SugarMate: Non-intrusive Blood Glucose Monitoring with Smartphones					Overall, 78%, 18% and 4% of theparticipants rate SugarMate asConvenient,NormalandInconvenient, respectively..94% of the participants report SugarMate asInstructiveand 6% reportNormal. No participant rates SugarMateasNon-instructive. From the feedbacks, it seems that most participants feel that continuous wearing of CGM devices is moreuncomfortable. Even if periodic re-calibration is required, many diabetic users think it is acceptable. The manualrecording of food intake is again the major concern, especially among non-diabetic participants. We will optimizethe food recording function in our future work		users. Forinstance, speech input instead of a scrolling menu might be a more welcome input mechanism. Intelligent searchhints based on personal input history using recommendation algorithms [15] may help to improve the userexperience when recording food intake, which is highly diversified and challenging to record automatically. In summary, users expect more functionalities of SugarMate such as recommending intensity and time of exercisesand recommending food plans. In this regard, the personalized blood glucose dynamics records collected bySugarMate can be sent to experts to recommend personalized precautionary measures					UNDECLARED
Making Sense of Sleep Sensors: How Sleep Sensing Technologies Support and Undermine Sleep Health - Proceedings of the 2017 CHI Conference on Human Factors in Computing Systems	2017		Ravich and ran, Ruth and Sien, Sang-Wha and Patel, Shwetak N. and Kientz, Julie A. and Pina, Laura R.	10.1145/3025453.3025557	To understand people's perspectives on sleep sensing devices and their potential for promoting sleep health, we surveyed 87 and interviewed 12 people who currently use or have previously used sleep sensors, interviewed 5 sleep medical experts, and conducted an in-depth qualitative analysis of 6986 reviews of the most popular commercial sleep sensing technologies.	Mixed	USA	General public	For smartphone apps, we analyzed reviews from the 4 highest-rated apps from the iTunes Store and the 5 highest-rated apps from Google Play. We selectedreviews in decreasing order ofword count(e.g., longest reviews first),stoppingonce we felt we reacheddatasaturation. For iTunes reviews, we reached data saturationat 280wordcount, analyzing475reviews out of a total of 2000possible reviews. For Google Play reviews,we reached data saturation at 500 word count, analyzing 377 out of a total of14581 possible reviews. Combining both sources, we analyzed 852 app reviews Survey Demographics (87 people)GenderWomen (50), Men (37) Agemin 18, max 73, mean 33.6, median 31 18–23 (12), 24–29 (27), 30–39 (28), ≥40 (22)Currently tracking?tracking (60), discontinued (29)Tracker typesmartphone app (3), fitness tracker (56), dedicated device	commercial sleep sensing technologies										Our findings show that sleep sensors increase awareness inprioritizingsleep and help usersaddress modifiable behaviors and their sleep hygiene. On the other hand, current feedback focuses on sleep metricspeople do not have control to directly change (e.g., time in sleep stages) and this distractsusers from focusing on aspects they have control over that improve sleep health.		UNDECLARED

The MONARCA Self-Assessment System: A Persuasive Personal Monitoring System for Bipolar Patients - Proceedings of the 2nd ACM SIGHIT International Health Informatics Symposium	2012		Bardram, Jakob E. and Frost, Mads and Sz\'{a}nt\'{o}, K\'{a}roly and Marcu, Gabriela	10.1145/2110363.2110370	This paper describes the MONARCA system; a persuasive personal monitoring system for bipolar patients based on an Android mobile phone. The paper describes the user-centered design process behind the system, the user experience, and the technical implementation.	Mixed	Unspecified	bipolar patients		The MONARCA self-assessment system: a persuasive personal monitoring system for bipolar patients					However, all the participants reported that it was much easier touse the phone-based self-assessment approach rather than using theold paper-based ones. The participants found that entering self-assessment data wasquick and smooth. They also found that the system is monitoring auseful set of data, and providing them with the option to enter per-sonal warning signs gives them leeway to adjust for personal needs.However, in an early version of the system, the patients could onlyenter data for the current day. This was seen as very problematicbecause despite the alarm, you could often forget or be unable tofill in the self-assesment.								UNDECLARED
Enabling User-Centered Privacy Controls for Mobile Applications: COVID-19 Perspective	2021	ACM Trans. Internet Technol.	Sharma, Tanusree and Dyer, Hunter A. and Bashir, Masooda.	10.1145/3434777	Several types of COVID-19 apps have enabled healthcare professionals and governments to communicate with the public regarding the pandemic spread, coronavirus awareness, and self-quarantine measures. We conducted an online survey of a midwestern region in the United State to assess people's attitudes toward such apps and to examine their privacy and security concerns and preferences.	Quantitative	USA	General public	participants. The partici-pants included 49.9.% female, 49.2% male, 0.3% nonbinary, 0.2% preferred to self-describe, and 0.3%preferred not to answer. Our sample participant's age ranged from 18 to 90 years old: 18–25 yearsold (16%), 26–35 years (18.72%), 36–45 years old (18.14%), 46–55 years old (16.8%), and so on. Theage groups are quite distributed and reported in Table7in the Appendix. The ethnic backgroundfor our participants were White/European-American (68.3%) followed by Black/African-American(13.3%), Latino/Latina (6.7%), Asian (4.7%), biracial (4.8%), Pacific Islander/Native Hawaiian (0.5%),Native American/Alaskan Native (0.5%), self-described (0.7%), and participants who preferred notto answer (0.7%)	everal types of COVID-19 apps have enabled healthcareprofessionals and governments to communicate with the public regarding the pandemic spread, coronavirusawareness, and self-quarantine measures											Survey resultsfrom 1,550 participants indicate that privacy/security protections and trust play a vital role in people's adop-tion of such apps. Furthermore, results reflect users' preferences wanting to have control over their personalinformation and transparency on how their data is handled. In addition, personal data protection prioritiesselected by the participants were surprising and yet revealing of the disconnect between technologists andusers.	UNDECLARED
Sociocultural Dimensions of Tracking Health and Taking Care	2020	Proc. ACM Hum.-Comput. Interact.	Bhat, Karthik S. and Kumar, Neha	10.1145/3415200	To dig deeper into these negative spaces that may go untracked, uncover potential sources of important health information, and more completely understand current tracking practices, we embarked on an interview study with patients with cardiac diseases in Bangalore, India.	Qualitative	India	patients with cardiac diseases	The patients' ages varied from 20 to 74 years, with an average age of around 50. The patientswere mostly male (12) despite concerted efforts to maintain gender balance. We conducted patientinterviews in their homes (13), workplace (1), and a doctor's clinic (4) (in the absence of thedoctor). In these interviews, we asked participants questions about their diagnosis, interactionswith their doctor, at-home care practices, as well as technology usage for assisting in cardiac healthmanagement.	Sociocultural Dimensions of Tracking Health and Taking Care	Our participants did not identify as digital natives, andusing technology for tracking health did not always come naturally to them.		Tracking was also occasionally viewed as potentiallyunnecessary, depending on specific healthneeds. For example, a doctor participant provided some insight on why tracking could be avoidable:"No I don't feel like [using technology for personal health tracking]. Like, if any risk factoris there, then we might try [using it]. But if no risk factor is there...I get my routineblood sugar test, kidney function tests done...once a year. I don't believe in unnecessaryevaluation and investigation"(D7)Even when participants did use health tracking technologies, this use did not always alignwith their health management needs. Thus, instead of engaging in cardiac health monitoring,participants would more generically track meals or weight-loss. Other participants who usedtracking technologies expressed difficulty in appropriating them for monitoring their cardiachealth. Explaining how the ability to monitor heart rate, without the ability to customize its use,exacerbated P16's heart rate fluctuation, P16's husband said,"[If] it goes above 100, again she getsworried and heart rate goes to 120-130, and I ask her why she's worried...She [has] palpitations...I'mgoing to change the settings of that [so that she can't check heart rate very often]."There are thus several reasons—we found—that may impact the adoption of health trackingtechnologies and consequently impact the generation of health data. Individuals might be morecomfortable with non-digital modes of tracking, see no reason for tracking in periods of goodhealth, rely on non-digital medical tests to monitor wellness, engage in tracking not relevant fortheir health condition, or simply find it stressful.	Gender Dynamics.We encountered the gendered nature of health management several timesin our research. Not only did gender appear to influence who suffered from heart disease, it shapeddoctors' interpretations of their patients' experiences, and treatments that could take place, as wellas conversations that could (or could not) occur.One female participant (P5) introduced us to a commonly held bias regarding heart disease,remarking that her doctor had informed her at the time of diagnosis that"heart disease is predomi-nantly for men."This belief, if widely held, would impact patients and caregivers by preventingwomen from getting themselves regularly checked, for instance.Doctors also appeared to believe that symptoms of cardiac disease were processed differentlyacross genders. One doctor participant (D8) noted,"I think [women] are typically more tolerant towhat they undergo. I don't think they voice their concerns as much..."Another doctor (D7) expressedthat several people came into the hospital each day with purportedly cardiac symptoms, but only5% of those were women. This was incommensurate to		Surveillance Care.We observed a converse effect of reverse parenting among some ofour participants, where spouses and adult children of participants used their self-tracking data toconfirm adherence to healthy lifestyles and care requirements.						UNDECLARED
Personalised Recommendations in Mental Health Apps: The Impact of Autonomy and Data Sharing - Proceedings of the 2021 CHI Conference on Human Factors in Computing Systems	2021		Pieritz, Svenja and Khwaja, Mohammed and Faisal, A. Aldo and Matic, Aleks and ar	10.1145/3411764.3445523	In a randomised placebo study with a two-way factorial design, we analysed the difference between an autonomous user experience as opposed to personalised guidance, with respect to both users'preference and their actual usage of a mental well-being app. Furthermore, we explored users' preference in sharing their data for receiving personalised recommendations, by juxtaposing questionnaires and mobile sensor data.	Mixed	Europe	General public	ographicParticularCompleteQGDGQADACSizeofPopulation2184552404041GenderFemaleMale1131052421252721191723261 5Age15-19#6-112220-24263385725-29236347330-34263946435-39257734440-44316953845-49174632250-54297954455-591142-3260+2453745EducationSecondarySchool69141913149Bachelor'sDegree921722171620Master'sDegree3095466Ph.D.orhigher8122-3TradeSchool1514442Prefernottosay43---1	MentalHealthApps							Thisshowsthat,irrespectiveofreceivingaguidedorautonomousexpe-rience,alluserspreferredtohaveanappthatsuggestsinterventionsforthemins teadofselectingactivitiessolelyontheirown Inaddition,wealsoexploredthedatacategoriesthattheusersinthedataselectionclusterweremostwillingtoprovide.Figure4showstheproportionofuserst hatprovidedaparticulardatamodal-ity.Theerrorbarsinthefgurerepresentthestandarddeviationoftheproportion sobtainedindividuallyfromDGandDA.Theuserswereleastwillingtoprovide1.callhistory(25.0%),2.bluetoothandwifdata(26.1%)and3.noiseintheenvironmentsampledfromthemicrophone(34.0%).Asexpected,theseareddatamodalitiesthathavethelargestprivacyandsecurityconcernsacrossbothusersandtechnologists[13,37,56].Additionally,thedatamodalitiesthatusersaremostwillingtoprovideare1.batterylevel(72.8%),2.numberofstepswalked (71.7%)and3.timespentondiferentapplications(68.5%				Thisindicatesthatallusers—irrespectiveofthewayofdatasharing—preferredtocompletethepersonalityquestionnaireoverprovidingtheirsmartphonedata.llusersbelievedthatcompletingapersonal-ityquestionnairehadlessprivacyrisks(Mdn=4, SD=2)comparedtoprovidingsensingdatafromtheirsmartphones(Mdn=5,SD=3).	UNDECLARED
Evaluating Health Information Apps on Real Life Smart Phone Use - Proceedings of the 2nd International Conference on Future Networks and Distributed Systems	2018		Shaheen, Reda and Kanwal, Safina and Iftikhar, Rimsha and shahid, Muhammad Rehman	10.1145/3231053.3231083	As the population is increasing day by day and the people are more interesting to know about their health information especially the elderly people, so they interact with the online technologies. Now it is important to design the Interface in such a way that the people do not feel frustrated by using them moreover the interface should be user-friendly and the people will not require any training in using these online technologies to view their health information. In this study, the role of emotional valence and arousal, their satisfaction level, the applications usability and what is the reason the different age group people prefer a specific interface format over others will be identified.	Quantitative	Pakistan	General public	Arousal of the people in using the different interface formats of in this research 200 volunteers among which 100 elderly and 100 young adults were taken and SUS, QUIS and SAM questionnaires were conducted among the	Health Information Apps			Elderly people showed their interest in using the Numeric apps rather than Animated and Diagrammatic because these apps increase their satisfaction level and are easier to understand for them. The interest of both the elderly and youth on Diagrammatic apps was normal as these apps have different images which created confusions regarding precautionary measures about their health.									UNDECLARED
Technology and Aging: Users' Preferences in Wearable Sensor Networks - Proceedings of the 4th EAI International Conference on Smart Objects and Technologies for Social Good	2018		Kolasinska, Agnieszka and Quadrio, Giacomo and Gaggi, Ombretta and Palazzi, Claudio E.	10.1145/3284869.3284884	In this paper, we study acceptability and usability of a system for silent monitoring with sensors hidden in wearable objects, which record data and send it to a server for further elaboration. The server collects and stores information, and gives an alert in case of danger. The goal of such a system should be to improve the safety and independence of end users with a technology as little intrusive and visible as possible.	Quantitative	Italy	older adults	Elderly Relatives Medical StaffParticipants4451 51Males25%29,4%31,4%Females75%70,6%68,6%Age range60 to 9724 to 6726 to 64AVG age764440SD age8,536,6911,73 he first section of the survey was dedicated to the personal dataof the respondents. We collected the information about the ageand gender of all the participants (Table 1). The elderly peopleand relatives were also asked about their level of education. Themajority of older adults (71%) obtained a primary education. Amongthe relatives two most frequent answers were consecutively a highschool (31%) and a secondary school (29%) diploma. The surveyaddressed to the health professionals included a question abouttheir specialization. As reported in Figure 1, nurses made up 43%of the respondents from the medical staff group. Other professionsare distributed as follows: 27% of doctors, 16% of	Wearable Sensor Networks	An overall familiarity with technology is lower among the elderly(with 52% of the group claiming to have only a little knowledge ofthis area), in respect to the health professionals (55% evaluate theirknowledge as good or very good) and relatives (31% claim to haveeither an average or very good knowledge of the field)		The majority of the relatives consider WSNsas highly useful both for their loved ones requiring assistance (73%)and for themselves as caregivers (67%, see Figure 4b				Mostof the older adults state their willingness to decide what kind ofdata they share (91%) and when (93%). 82% of the medical staff and69% of the relatives agree that the elderly should be in control ofwhat information to share.In oder to make sure that our target group would not experienceany kind of discomfort using the proposed technology, we asked therespondents to what extent they would feel monitored. As reportedin Figure 6, 68% of the participants answered either 'not at all' or 'alittle bit' to this question (57% and 11% respectively), which showsthat an actual level of discomfort experienced by the elderly is evenlower than estimated by the relatives or medical staff			An additional part of the survey addressed to the medical staffasked them what kind of patients would benefit the most from aWSN and what possible applications it may have. The most com-monly appearing answers for the first question were: older adults(especially if living alone and being relatively independent), demen-tia patients with different types of neurodegenerative diseases (withmost common reference to Alzheimer's disease), patients sufferingfrom chronic diseases, such as diabetes or cardiovascular diseases,psychiatric patients and individuals with physical disability. Amongthe most frequently mentioned applications of the system, therewere: locating the position of patients affected by the wanderingbehavior, monitoring vital signs and falls prevention		UNDECLARED
A User-Centred Approach to Inform the Design of a Mobile Application for STI Diagnosis and Management - Proceedings of the 27th International BCS Human Computer Interaction Conference	2013		Gkatzidou, Voula and Hone, Kate and Gibbs, Jo and Sutcliffe, Lorna and Sadiq, Syed Tariq and Sonnenberg, Pam and Estcourt, Claudia		This paper describes a patient-centred approach to the design of a mobile application to support self-testing for sexually transmitted infections, providing easy and immediate access to diagnosis, treatment via electronic prescription, and partner notification.	Qualitative	UK	STI patient	The inclusion criteria for the study were fairly relaxed to explore a broad and diverse set of preferences, attitudes and perspectives. For this reason screening criteria for the focus groups were only age (16 to 24 years old) and mobile phone ownership. Participants (n=49) were smartphone users and were recruited from a Higher Education Institution (49%) in London and a Further Education College in an economically disadvantaged area in the North East of England with high prevalence of Chlamydia (51%). Median age of participants was 19 years and mosparticipants were female (53%); participants were	a mobile application for STI diagnosis and management							Personalisation most participants felt it would be inconvenient to have to access a computer or log on to a website, and wanted all or most of their interaction to take place directly on the smart phone 4.5 Context of use Participants were asked what the context of use of the mobile app would be and the majority of them agreed that they would access the system on their mobile (rather than on a desk top). They also highlighted that they estimated that their interaction with the system would be in short chunks of time. When asked whether they would download and install the app or access it over the Internet, participants' replies were varied. The majority of them highlighted a preference for an app that would require download and installation (native app), as opposed to an app that they would access through their web browser (web app). Nevertheless, the participants also highlighted a one-off context of use, with deleting the app after they have got their results and treatment				4.1 Legitimacy and Trust Legitimacy was a concern to the majority of the participants who saw the design and the content of the application as cues to determine the legitimacy of the service. During focus group discussions, participants have expressed their overall satisfaction with respect to the content being 'clear' with a 'serious tone', which established the application as a professional and credible medical resource. Participants unanimously agreed that the inclusion of a NHS endorsement or affiliations with Sexual Health Centres will engender further trust inhe service and will establish the online medical consultation as 'credible'. Similar to findings from	UNDECLARED
Patients, Pacemakers, and Implantable Defibrillators: Human Values and Security for Wireless Implantable Medical Devices - Proceedings of the SIGCHI Conference on Human Factors in Computing Systems	2010		Denning, Tamara and Borning, Alan and Friedman, Batya and Gill, Brian T. and Kohno, Tadayoshi and Maisel, William H.	10.1145/1753326.1753462	Implantable medical devices (IMDs) improve patients' quality of life and help sustain their lives. In this study, we explore patient views and values regarding their devices to inform the design of computer security for wireless IMDs.	Qualitative	Israel	cardio patient	The patients' average age was67.9(median= 69, range= 41–80). Our participant population was, on average, ontheir second implanted cardiac device (median= 1, range=1–3+) and had lived with a device for7.8years (median= 6, range =0–18)	a watch, a pedometer, and a heart-rate monitor			An Argument for Multiple Systems: User SatisfactionThe Least Disliked: Proximity BootstrappingIn the absence of a consensus on a liked system, we usethe technique of VSD dams and flows as the inspiration toisolate multiple systems that would achieve more complete"coverage" for the participants. We focused first on "dams,"since it is particularly important at a minimum that everypatient have a choice of a system that they do not activelydislike.As shown in Table 1, the proximity bootstrapmockup is not the most liked of the systems, but the factthat no participants disliked the mockup makes it a goodcandidate for part of a security solution for implantablecardiac devices. This security solution, however, is notoptimal: only27%(3 out of 11) of the participants likedit and would choose to use it.The Most Liked: Emergency and Warning WristbandOnce we had a system that none of the participants in oursample disliked, we shifted our attention to "flows," seekingto select additional system mockups to create a portfolio ofsecurity systems that give a higher percentage of participantsan option that they would like or choose to use. From inspec-tion of the data (see Table 1), the most natural choice appearsto be the emergency and warning wristband mockup, sinceit has high percentages in both categories. Another possiblecandidate might be the criticality-aware IMD. However, ifwe choose two mockups as system options, we should avoidmockups which tend to be liked, disliked, and/or selected bythe same participants, since choosing both systems wouldbe redundant.One way to measure this is to considerthe correlation between the like/dislike scores for pairs	the criticality-aware IMD are positively correlated with theproximity bootstrap (using Kendalls tau-b, a non-parametriccorrelation coefficient,τ= 0.510), so this mockup wouldnot be a useful system to add to the solution portfolio. On theother hand, the like/dislike ratings for the proximity boostrapand the emergency and warning wristband are essentiallyuncorrelated (τ=−0.131). Thus, these two mockups—the proximity bootstrap and the emergency and warningwristband—are a reasonable choice to put together. Betweenthe two of them, 7 out of our 11 participants have at least onechoice of a system that they liked, 6 of the 11 participantshave a system that they said that they would select, all 11can have at least one system that they do not dislike, and8 of the 11 have a choice of two systems that they do notdislike.Satisfying the Stragglers: UV-Visible TattooDespite its poor overall ratings (see Table 1), if we were toadd a third system to the solution portfolio, the best choicewould be the UV-visible tattoo. The criticality-aware IMDhas slightly better ratings overall, but its ratings are highlycorrelated with the proximity bootstrap. On						Mockup System Properties: Pros and ConsAlthough the population size was too small to generatedefinitive statistical analyses, the interview responses doprovide qualitative insight into the perspectives and concernsof patients with implanted cardiac devices. Below is a listof (non-mutually-exclusive) properties—both desirable andundesirable—that surfaced during discussions with partici-pants about the system designs.Security.Participants expressed liking systems becauseof their perceived security benefits and disliking systemsbecause of their perceived security flaws. Moreover, some-times the same system received both positive and negativefeedback on its security properties. As an example, differentparticipants complemented and critiqued the medical alertbracelet system based on its security properties.Someparticipants appreciated that access to their IMD would beprotected by a password, while others objected to the factthat their passwords would be carried around with them andmight be acquired by others.Safety.Participants were very interested in how the mockupsystems affected their safety in emergency situations. Oneof the better-	attoo. Additionally, some participants were concerned thatthe criticality-aware IMD did not have a manual override.Privacy.Some mockup systems were called out as hav-ing negative effects on privacy. In particular, participantsworried that wearing something or having some other visualindicator would force them to broadcast their medical con-dition to others. In the words of one patient:Subject E:I don't like the idea of wearing the wristband...Ialready have a defibrillator. Why do I have to wear some-thing on my hand...to show that I have-, that I have adefibrillator, that there's something wrong with me. No.Aesthetics.Participants disliked some system mockupsbecause they found them to be unaesthetic. One participantcommented that the tattoo was visually too "busy." Therewere also frequent comments about the appearances of thewristbands. The wristbands are meant to be worn at alltimes; some participants saw their unattractiveness as amajor	UNDECLARED
Examining Self-Tracking by People with Migraine: Goals, Needs, and Opportunities in a Chronic Health Condition - Proceedings of the 2018 Designing Interactive Systems Conference	2018		Schroeder, Jessica and Chung, Chia-Fang and Epstein, Daniel A. and Karkar, Ravi and Parsons, Adele and Murinova, Natalia and Fogarty, James and Munson, Sean A.	10.1145/3196709.3196738	Self-tracked health data can help people and their health providers understand and manage chronic conditions. This paper examines personal informatics practices and challenges in migraine, a condition characterized by unpredictable, intermittent, and poorly-understood symptoms.	Mixed	USA	People with Migraine	Survey Participants (279 People)Gender222 female, 51 male, 2 FtM transgender,1 genderqueer, 1 genderqueer man, 2 no answerAge18–96, x̄= 33.8 (7 no answer)Years withMigraine1–65, x̄= 17Apps Tried(106 people)Migraine Buddy (71); 40 apps with <10 people each(14 could not remember)Apps Used(56 People)Migraine Buddy (30); 23 apps with <10 people eachOtherResourcesTried(196 People)paper journals (83), online articles about migraine(70), online forums such as reddit (44), customspreadsheets (30), calendars (24), books aboutmigraine (14); 17 methods with <10 people eachMigraine Interview Participants (13 People)Gender11 female, 1 male, 1 genderqueer manAge20–61, x̄= 32.15Years withMigraine1–30, x̄= 15.8Apps Tried(8 people)Migraine Buddy (5), Migraine Coach (2), Fitbit (1),Clue (1),	Self-Tracking by People with Migraine			Many found technology useful for migraine-related tracking.		hose who had used apps often felt their app was easy to use(53/106) and helped them identify trends and triggers (42/106).However, many found the tools they used failed to fullysupport their goals.	Informed by and building upon prior research onself-trackinggoals (e.g., [12, 26, 42, 46, 46, 61]), we find four primary goalspeople bring to migraine-related self-tracking: 1) answeringquestions about migraines, 2) predicting and preventingmigraines, 3) monitoring and managing migraines over time,and 4) enabling motivation and social recognition.	72 of 106 survey participants who had used apps and 11 of 13interview participants expressed a desire for apps to bettersupport customization (I1,I2,I3,I4,I5,I6,I7,I9,I10,I11,I12).A second key dimension iswhena person tracks, specificallywhether they prefer trackingduringoraftermigraine symptoms.Of 13 interview participants, 7 reported tracking either duringor after a migraine (I1,I2,I4,I6,I7,I9,I19), 4 reported onlytracking after a migraine (I3,I8,I11,I13), and 2 reportedonly tracking during a migraine (I5,I12). Some people cannottrack during a migraine: 36 survey participants mentionedhaving trouble using apps because interacting with a screenduring a migraine was painful.					UNDECLARED
The 'Self' as Barrier for Self-Management Technologies in Healthcare? - Proceedings of the 12th EAI International Conference on Pervasive Computing Technologies for Healthcare	2018		V andenberghe, Bert and Vanhoof, Jasper and Voorend, Roos and Geerts, David and Dobbels, Fabienne	10.1145/3240925.3240974	We designed and evaluated the PICASSO-Tx system: a technology solution to support transplant patients and their self-management of diet, physical activity and medication adherences.	Qualitative	Belgium	transplant patients	19 patients (11 males, 8 females; aged 21–69 with an average age of 52). The patients in this study were recruited by the academic centerfor nursing. We included five patients of the heart, kidney, and lung transplant departments, and four patients of the liver transplant department. We balanced patients who claimed they do or do not self-manage one or multiple of our target behaviors (diet, physical activity, and medication adherence). Next to that, we aimed to balance time since transplantation, sex, age, marital status, education, and employment. We used several inclusion criteria: patients needed to be fit enough to participate, live in or close to Leuven (Belgium), and be able to understand and sign the informed consent forms.	We designed and evaluated the PICASSO-Tx system: a technology solution to support transplant patients and their self-management of diet, physical activity and medication adherences.			department. We balanced patients who claimed they do or do not self-manage one or multiple of our target behaviors (diet, physical activity, and medication adherence).									UNDECLARED
Disability, Age, and Informational Privacy Attitudes in Quality of Life Technology [illegible]	[illegible]	ACM Trans. A[illegible]	Beach, Scott and Schulz, Richard and Downs, Julie and Matthews, Judith and [illegible]	10.1145/[illegible]	Using data from a national Web survey (n=1,518), we focus on perceived acceptability of sharing information about toileting, taking medications, moving about the home, cognitive ability, driving behavior, and vital signs with five targets: family, healthcare providers, insurance companies, researchers, and government.				For this survey, we targeted both disabled andnondisabled adults from the following groups (with the number of respondentsin parentheses): physical disability (348), rheumatoid arthritis (103), stroke(101), emphysema (74), multiple sclerosis (71), baby boomers (age 45–64) notin any of the aforementioned disability/disease groups (303), and older adults(age 65 and over) not in any of the disability/disease groups(610). Members ofthe disability/disease groups could be of any age. Table I. Sample Characteristics and Descriptive Statistics for Entire Sample and by Broad Age CategoryTotal Sample45 - 6465 and older(n = 1,518)(n = 762)(n = 756)n (%)n (%)n (%)GenderMale499 (32.9)198 (26.0)301 (39.8)Female1019 (67.1)564 (74.0)455 (60.2)Age45- 64762 (50.2)762 (100)NA65 - 74591 (38.9)NA591 [illegible]	Quality of Life Technology [illegible]			Re-sults show that sharing or recording information about toileting behavior; sharing informationwith the government and insurance companies; and recordingthe information using video wereleast acceptable. Respondents who reported current disability were significantly more [illegible]									

Shaping the Design of Smartphone-Based Interventions for Self-Harm - Proceedings of the 2020 CHI Conference on Human Factors in Computing Systems	2020		Honary, Mahsa and Bell, Beth and Clinch, Sarah and Vega, Julio and Kroll, Leo and Sefi, Aaron and McNaney, Roisin	10.1145/3313831.3376370	In a move towards enhancing the accessibility of personalized interventions for self-harm, we undertook a three-stage study. We first conducted interviews with 4 counsellors in self-harm to understand how they clinically respond to self-harm triggers. We then ran a survey with 37 young people, to explore perceptions of mobile sensing, and current and future uses for smartphone-based interventions. Finally, we ran a workshop with 11 young people to further explore how a context-aware self-management application might be used to support them.	Mixed	UK	young people self harm	participants from only the LGBTQ+ community, all participants (except 1) knew each-other, mostly through the LGBTQ+ group. However we did not ask participants to disclose or discuss their sexuality (as this was not the focus of our research). Each participant disclosed their gender identity at the beginning of the workshop by discussing their preferred pronouns (male n=6, with only n=2 cisgender males) A total of 37 people responded to our online survey (mean age 16.8 years, sd. 2.1). The sample was predominantly female (21 female, 6 male, and two people identified as gender-fluid)	Smartphone-Based Interventions for Self-Harm			The majority of respondents said that they would be interested in an app to support self-harm (n=31) and were happy to share their phone usage time (n=24), location (n=22), and what health websites or apps they use (n=22).			Over half of participants (n=22) reported that they use their phones to help them when they were feeling distressed. We used an open question to further probe how apps were being currently used, identifying three major coping strategies: consuming audio-visual media (n=8), e.g., "Use to look at things that I love or even just to watch funny videos when I am in a bad way" (P9); seeking peer-support (n=8), e.g., "I try and text my friends to try and distract myself" (P11); and using meditation or mental-health apps (n=11).	Participants discussed opportunities for the phone to initiate distraction techniques (n=9) and providing CBT techniques to help the individual think clearly and rationally about their thoughts and emotions (n=5). Thirdly, they favoured the app to provide instructions about the activity, for example instructing the user to draw a specific image.	For one participant, the phone itself acted as a barrier, protecting them from engagement with other people at times of distress: "It's a social crutch and helps me to cope in a panic attack. If I'm on my phone, people tend not to bother me" (P35).		The first was around helping the user regain a sense of control by calming themselves down during distress (n=10); a desire for human connection and communication was also discussed (n=5). Gaining advice through an application was also seen to have value (n=5 Furthering this, the participants found compliments for pos-itive actions e.g., exercise or completing a task "reward-ing" and considered it to improve mood in other contexts	tracking the negative was perceived to give rise to negative thoughts.	UNDECLARED
Me, My Health, and My Watch: How Children with ADHD Understand Smartwatch Health Data	2022	ACM Trans. Comput.-Hum. Interact.	Ankrah, Elizabeth A. and Cibrian, Franceli L. and Silva, Lucas M. and Tavakoulnia, Arya and Beltran, Jesus A. and Schuck, Sabrina E.B. and Lakes, Kimberley D. and Hayes, Gillian R.	10.1145/3577008	Children with ADHD can experience a wide variety of challenges related to self-regulation, which can lead to poor educational, health, and wellness outcomes. We conducted a deployment study with 10 children, aged 10 to 15, for six weeks, during which they used a smartwatch in their homes.	Qualitative	Unspecified	Children with ADHD	All the child participants were boys between the ages of 10 and 15. ADHD diagnosis is more diagnosed in boys [75, 88, 109]; thus, the recruitment of a greater number of boys was expected.	Children with ADHD Understand Smartwatch Health Data	6 with experience		Having never had a smartwatch, many of the children were excited to receive the new technology.		4.2.1 Tactile Sensitivity. Children with disabilities may experience physical discomfort when wearing a device developed for adults without tactile sensitivity, such as the Apple Watch. 4.2.2 Inattention and Working Memory. Children with ADHD experience difficulty with sustaining attention and attending to details in some contexts 4.2.3 Challenges to Engaging in Data Collection. One child expressed different comfort levels with an automated tracking process depending on the device. 4.3.1 Sleep Data. Many of the children were excited about the prospect of access to their sleep data, but they soon realized that the information given to them by the sleep app was not as they expected. Some children could not understand the data presented to them by the sleep app. 4.3.2 Activity and Fitness Data. This may demonstrate that children are not familiar with the unit of measurement known as calories leading them to compare it to a more familiar unit of measure. The children experienced similar challenges in conceptualizing heart rate, another numerically represented dataset.	4.1.2 Toward Greater Autonomy. When asked about their first impression of the watch, children demonstrated enthusiasm to use it and to track information to support their interests. Additionally, the children in the study demonstrated full awareness of their ADHD symptoms and triggers and described seeing the smartwatch tracking capabilities as a tool to help them identify and manage their triggers.						UNDECLARED
Making Sense of Complex Running Metrics Using a Modified Running Shoe - Proceedings of the 2021 CHI Conference on Human Factors in Computing Systems	2021		Wo\'{z}niak, Pawe\l{} W. and Zbytniewska, Monika and Kiss, Francisco and Niess, Jasmin	10.1145/3411764.3445506	In this paper, we investigate how data from shoe-worn sensors can be visualised to empower runners to improve their technique.	Mixed	Germany, Netherlands, Swtherland	student	female, 26 male, aged 18–32, M = 26.00, SD = 2.73) through our university mailing list and by distributing posters around the campus. Participants attended the study individually and did not communicate with other participants. We required that users declare they were able to run for 20 minutes and arrive in their own running equipment. All participants declared to be advanced amateur runners, training more than once a week and committed to a running regime (i.e. they all belonged to the user group defned by Knaving et al. [24]). There were no participants with professional running experience. Each participant was remunerated with EUR 10. Additionally, isotonic drink and water were available throughout the study	We designed GraFeet—an augmented running shoe that visualises kinesiological data about the runner's feet and gait.			Furthermore, participants commented on how GraFeet used a shoe to represent gait data. They found it useful to relate the position of the visualisation to the location on their feet.		The grand mean of the usability score was M = 71.53 (SD = 12.64). GraFeet was perceived as more usable with M = 76.39 and dashboard was rated on average M = 66.67. A Mann-Whitney U test revealed a signifcant diference (U = 94, p < 0.05)Many participants were confused by the abundance of data ofered in the dashboard. They not only required reference metrics, but also wondered about the meaningfulness of the metrics. One participant requested embedding knowledge in the interface					Form Factor. Some of the users commented specifcally on the idea of presenting the data on an object rather than a traditional screen. They found that it empowered them to have an overview of the situation at a given time point during the run and made the data more relevant: Some of the users reported an increased sense of understanding of their body after using GraFeet.Finally, the majority of GraFeet users expressed a wish to use it longer and anticipated that interfacing with foot sensor data could lead to a better bodily awareness and improved running technique		UNDECLARED
Quantitative and Qualitative Exploration of Electronic Device Position on User Experience of Smart Clothes - Proceedings of the 5th International Conference on Computer Science and Software Engineering	2022		Wang, Ying and Wang, Zexi and Guo, Shihui and Lin, Juncong	10.1145/3569966.3570053	In this paper, we explore gaps in the user experience of using circuit boards in motion-monitored smart clothing. We focus on the effects of board location on the user motion while wearing the smart garment by analyzing a motion capture dataset to explore the least constrained joint points in motion, as well as the user's subjective perception.	Mixed	China	General public	Twenty users were selected for the experiment, including 10males and 10 females distributed between the ages of 22-26. Theduration of the experiment was approximately one hour for eachuser. Each user was interviewed and questionnaires were completedto capture their subjective thoughts as fully as possible	Smart Clothes					board is so present after wearing it that it getsin the way of the movement being performed. Nearly half of theresults were 1 or 2, and the combination of the type of movementat the time and the location of the circuit board suggests that theboard is best placed in a non-primary part of the movement.Comfort level.Comfort refers to whether the user will feelcomfortable and appropriate after wearing smart clothing for exer-cise. The result is that more than 85% of the scores are below 5 or279 equal to 5, which indicates that in most cases, wearing in the rightposition for sports is more comfortable for the user.Firmness.Questions 3 to 6 all reflect the solidity of the simulatedcircuit boards in this experiment from different aspects. The resultsshow that more than half of the experience scores are between 1-2,and more than 87% of users gave scores less than or equal to 5. Itshows that the circuit boards selected in our experiments feel solidwhen users wear them for sports, regardless of their position.Aesthetic level.The aesthetics of the circuit board dependson the answer to question 7 in our objective evaluation section.More than 75% of the users gave a score of less than 5, indicatingthat the location of the circuit board in this paper's experiment isappropriate and aesthetically pleasing to the use							UNDECLARED
Examining Menstrual Tracking to Inform the Design of Personal Informatics Tools - Proceedings of the 2017 CHI Conference on Human Factors in Computing Systems	2017		Epstein, Daniel A. and Lee, Nicole B. and Kang, Jennifer H. and Agapie, Elena and Schroeder, Jessica and Pina, Laura R. and Fogarty, James and Kientz, Julie A. and Munson, Sean	10.1145/3025453.3025635	To understand menstrual cycle tracking practices, we collected and analyzed data from three sources: 2,000 reviews of popular menstrual tracking apps, a survey of 687 people, and follow-up interviews with 12 survey respondents.	Mixed	USA	women	To understand menstrual cycle tracking practices, we collected and analyzed data from three sources: 2,000 reviews of popular menstrual tracking apps, a survey of 687 people, and follow-up interviews with 12 survey respondents.	Menstrual Tracking			Some people appreciate tracking these factors. Women who are trying to conceive often find phone apps useful [28]. Alternatively, 19 women in our study reported using a phone app to avoid becoming pregnant.			We surfaced five reasons women track their menstrual cycles. Women track to: (1) be aware of how their body is doing, (2) understand their body's reactions to different phases of their cycle, (3) be prepared, (4) become pregnant, and (5) inform conversations with healthcare providers. Participants were typically motivated by multiple factors.	Given how crucial prediction accuracy is to menstrual cycle tracking, designers should evaluate additional techniques for modeling and communicating predictions about a woman's cycle. Reasons for tracking likely correspond to different prediction needs. A woman tracking to avoid becoming pregnant would probably prefer her app overestimate her ovulation window. A woman trying to become pregnant may instead prefer a conservative estimate of her ovulation. For both ovulation and period arrival, designers should consider and evaluate interfaces that present probabilities as an alternative to unreliable binary predictions.More people can benefit from tools with gender-neutral themes that do not assume gender in labels, iconography, and functionality. Similarly, when logging sex, apps should be careful not to assume the sexual orientation or gender of either the person logging or their partner(s). Logging sex and reporting on fertility can be desirable for some women and uncomfortable for others. Designs should consider including features to support tracking sex and fertility, but should also support disabling those features.Apps for menstrual cycle tracking should design for customization. Designs should either be discreet by default or provide a neutral non-obvious interface option. Such discreetness is similar to Consolvo et al.'s design choice in UbiFit Garden, abstractly encoding physical activity data as a garden [9]. Many women desire app color schemes, icons, names, and notifications that do not draw attention from others who casually glance at their phone. Designs should support disabling and customizing notifications. Any					UNDECLARED
僩 Don抳 Need a Goal? Attitudes and Practices in Fitness Tracking beyond WEIRD User Groups - Proceedings of the 23rd International Conference on Mobile Human-Computer Interaction	2021		Niess, Jasmin and Wo\'{z}niak, Pawe\l{} W. and Abdelrahman, Yomna and ElAgroudy, Passant and Abdrabou, Yasmeen and Eckerth, Caroline and Diefenbach, Sarah and Knaving, Kristina	10.1145/3447526.3472062	While past work mainly focused on WEIRD (Western, Educated, Industrialised, Rich, Democratic) fitness trackers usersin North America and Western Europe, this paper investigates another perspective on fitness tracking.	Qualitative	USA, Europe and Egypt	General public	Overall,werecruitedN=37participantsagedfrom18–62 (M=30.44,SD=9.71),with24beingmaleand13female. 3.2.1Non-ArabWesternUsers.Forthenon-ArabWesternsample, n=18participantswererecruitedthroughsnowballsampling startingwithsocialmediaposts.Theparticipantswereaged18–41 (M=26.67,SD=5.42);ninemaleandninefemale.Allparticipants werenativesoftheircountryofresidence(withtheexceptionof W8—aUK-basedIrishman).Whiletheonlyinclusioncriterionfor thestudywasusingafitnesstrackerforthreemonthsormore,we foundthattheparticipantsinthenon-ArabWesterngrouplargely reflectedthestereotypeusersasdiscussedby Spieletal.[61], namelywhite,healthyandalreadyfit.Theseaspectswereassessed	FitnessTracking beyondWEIRDUserGroups			Thismaysuggestthat ArabEgyptianusersmaybe morereceptivetotechnologiesthatsupportreflectionratherthan providingprescriptivemeasures	Ouranalysissuggeststhatsomeofthevariabilityinmotivations canbeexplainedbyculturalfactors.			wesuggestthatfuture trackersshouldofferagreaterdegreeofflexibilitytooffera more inclusiveexperience.					UNDECLARED
Wearable Lifestyle Tracking Devices: Are They Useful for Teenagers? - Adjunct Proceedings of the 2015 ACM International Joint Conference on Pervasive and Ubiquitous Computing and Proceedings of the 2015 ACM International Symposium on Wearable Computers	2015		Carrion, Carme and Caon, Maurizio and Carrino, Stefano and Moliner, Liliana Arroyo and Lang, Alex and ra and Atkinson, Sarah and Mazzola, Marco and Perego, Paolo and St and oli, Carlo Emilio and Castell, Conxa and Espallargues, Mireia	10.1145/2800835.2809442	The objective of this study is to investigate what teens think of current wearable devices for fitness tracking, using the results to provide preliminary suggestions for the design of wearable technology aimed at adolescents.	Qualitative	Spain	teenager	Three groups of ten 14-16 years old teens from different schools, chosen by convenience, have participated: a) Boys (middle-class) from an urban partially state-funded school in Barcelona (Figure 1), b) mixed group (lower-middle class) from a partially state-funded school located a mid-city in Barcelona suburbs and c) girls (lower-class) form a public-funded school 80km South of Barcelona	Wearable lifestyle tracking devices	All teens are considered digital natives and they are familiar with Smartphones and use apps. Most of them have their own Smartphone (80%). Generally speaking, students are unfamiliar with the concept of "personal monitoring". When asked to provide examples of activities they monitor in their daily life, they tend to suggest examples of time control and quantifiable aspects (Table 1). In terms of needs by gender, boys are more concerned with their fitness while girls understand healthy and good-looking bodies as a result of being fit and eating well and adequate intake. Knowledge and familiarity with life trackers appeared to be limited; consequently, few opinions were given about what the gadgets may be useful for or how teens can benefit from using them. ("What I				In general terms, they have qualified the life tracker as something useful in abstract terms; their speech reveals that the key to consider the need of a gadget like this is to have a serious goal and strong purposes to achieve it. Thus, they feel that life trackers are not targeting adolescents but other sectors such as frequent sportsmen and sportswomen, older people or patients with heart disease or related health conditions. In general positive aspects are more than negative issues.		The most salient aspects that would drive their choice about the potential use of wearables life trackers are a combination of appearance and utility listed below: xAppealing xGood design and good looking (regarding color and shape) xSmall size xAdequate shape to wear it comfortably xTactile screen xCustomizable gadget xEntertaining xInformativ	The major failure was produced at the very beginning; around half of participants were unable to either download the app or synchronize it with their Smartphones. As a result, the gadget was turned into a fashion complement or, in the best cases, used as a watch. Despite the technical problems, they feel that life trackers are useful (particularly boys). The main uses have been counting calories burned, steps made, and sleeping time. These three functions were of most interesting because they were related to sports and activity contexts which were familiar to the participants and highly associated to goal settings and individual achievement. However, other fitness indicators that may be important and closely related to health status such as blood oxygen seem meaningless to them. The lack of context or references for interpreting the data gathered was mentioned several times. None of the teens mention the differences in accuracy, despite some of them explain cases of wrong monitorisation results, for instance in calories and distance.		Having a "coach" or a set of personalized tips is something they rated as highly positive. As most of them have their own Smartphone, it is given the impression that there is some overlap with the life tracker, or even is simpler and easier than the mobile phone. Life trackers can be seen as a potential substitute for the Smartphones on occasions when doing physical activity because a Smartphone is less "wearable".	Beyond the technical issues the main negative comment is related to control and privacy. They dislike being under control by a machine.	UNDECLARED
nescapable?Cycle of Pain: Supporting Wheelchair Users?Upper Extremity Health Awareness and Management with Tracking Technologies - Proceedings of the 2023 CHI Conference on Human Factors in Computing Systems	2023		Li, Yunzhi and Li, Franklin Mingzhe and Carrington, Patrick	10.1145/3544548.3580660	In this work, we explore how health tracking technologies could support wheelchair users?UE health self-care.	Qualitative	USA	Wheelchair Users	The wheelchairuserswererecruitedhadanaverageageof43.8(SD=12.48)andanaverage20.8yearsofusingwheelchairs(SD=13.62).	SupportingWheelchair Users'UpperExtremityHealthAwarenessandManagementwith TrackingTechnologies	Theparticipantsalsoreportedlimited experiencewithfitness trackingdevicesorapplications.						5.2.1Provideemotionawareness. 5.2.2Supportcapacityunderstanding. 5.2.3Facilitatecommunicationwiththerapists.					UNDECLARED
What Health Topics Older Adults Want to Track: A Participatory Design Study - Proceedings of the 15th International ACM SIGACCESS Conference on Computers and Accessibility	2013		Davidson, Jennifer L. and Jensen, Carlos	10.1145/2513383.2513451	Older adults are increasingly savvy consumers of smartphone-based health solutions and information. We involved older adults (aged 65+) in the beginning stages of designing a mobile health and wellness application.	Qualitative	USA	Older adults	Eighteen participants were aged 65 years and older (median age 71), 12 of them were women. They were volunteer participants with a wide range of professional backgrounds	health topics older adults want to track							could focus on moving forward is expanding what is offered in terms of tracking rest. One group was interested in tracking sleep and other restful activities like meditation, yoga, or gardening. 4.5.2 Social Interaction Tracking Another aspect of a design that was not represented in the app stores, was social interaction tracking. 4.5.3 Local Stress Relief Activities Another idea for an application was to have a database of suggestions for activities to do in your local community that could relieve stress. S 4.5.4 Healthy Eating Suggestions As part of one of the designs (discussed in Section 4.1), the participants wanted a way to receive advice on how to change their diet based on their desires. These topics showed that our participants had an interest in some hardto-measure health topics, which are more subjective than blood pressure, for example.					UNDECLARED
拆aking Care of Myself as Long as I Can? How People with Dementia Configure Self-Management Systems - Proceedings of the 2021 CHI Conference on Human Factors in Computing Systems	2021		Dixon, Emma and Piper, Anne Marie and Lazar, Am and a	10.1145/3411764.3445225	Grounded in interviews with seventeen technology savvy people with mild to moderate dementia, our analysis reveals their use of technological and social resources as part of the work of self-management. We detail how participants design self-management systems to enable desired futures, function well in their social world, and maintain control.	Qualitative	USA	People with Dementia	age: 57-73; 10M, 6F; Caucasian	People with Dementia Configure Self-Management Systems						an enormously stigmatized condition [10]. Participants describe practices they employed to obscure the appearance of cognitive impairment in order to blend in socially and project normalcy. 4.2.2. Managing Emotions to Avoid Negative Social Consequences. Participants describe how dementia has changed their overall emotional well-being and how these emotional changes relate to their personal relationships. 4.2.3. Attending to the Preferences of Others. Participants actively consider the preferences of their loved-ones when designing their self-management systems.	Participants devise systems to support their self-management in the short term as well as the longer-term future, centered around an expectation that they could not rely on memory alone. 4.1.1. Building Redundancy. Not being able to depend on one's memory means that it is essential to verify information through redundancy. 4.1.2. Providing Just-in-Time Information. A second strategy used by participants to prepare for future events was building self-management systems that could provide just-in-time information. 4.1.3. Outsourcing Tasks. When designing self-management systems for future use, participants create workfows that enable them to outsource tasks that are becoming difficult given their changing abilities.			Participants describe technology as an integral part of their self-management systems	4.3.1. Avoiding Nefarious Interference. Participants avoided certain technologies that might otherwise fll a need in their self-management systems in order to prevent being exploited through scams or hacks. 4.3.2. Protecting Against Well Intentioned Others. Participants also avoided well-intended resources that threatened their control over their self-management systems.	UNDECLARED
Student Perspectives on Digital Phenotyping: The Acceptability of Using Smartphone Data to Assess Mental Health - Proceedings of the 2019 CHI Conference on Human Factors in Computing Systems	2019		Rooksby, John and Morrison, Alistair and Murray-Rust, Dave	10.1145/3290605.3300655	The approach, which is sometimes termed Digital Phenotyping, has potential to transform how mental health and wellbeing can be monitored and understood. In this paper we report findings from in-depth focus groups, prototyping and interviews with students.	Qualitative	UK	student	11participantswerefemaleand4male,8wereundergraduateand7 postgraduate.Theaverageagewas23.5(min=18,max=30, sd=3.6).	Agrowingbodyofinterdisciplinaryresearchhassuccessfullydemonstratedthatusingsensorandinteractiondata fromstudents'smartphonescangiveinsightintostress,depression,mood,suicideriskandmore.			We findtheyarepositiveaboutmentalhealthtechnology,but alsothattherearemulti-layeredissuestoaddressifdigital phenotypingistobecomeacceptable.		Therewerealsoworriesaboutneedingto chargebatteriesfrequently.						However,participantspointedoutthatthe proposedtechnologymaycausepeopleworryandanxiety bythequestionsaskedandintermsofwhatlecturersand tutorsmayfindout.Theywerealsoconcernedaboutaloss ofautonomyanddignityinnavigatingsystemsofcare.	UNDECLARED
Deep Vision Shield: Assessing the Use of HMD and Wearable Sensors in a Smart Safety Device - Proceedings of the 12th ACM International Conference on PErvasive Technologies Related to Assistive Environments	2019		Colombo, Sara and Lim, Yihyun and Casalegno, Federico	10.1145/3316782.3322754	Miniaturized and wearable technologies have the potential to augment human senses and capabilities when embedded into everyday objects. We investigate how such technologies can impact workers' health and safety in the energy industry through the design, prototyping and evaluation of a smart safety device.	Qualitative	Italy	worker in energy industry	men	the use of HMD and wearable sensors in a smart safety device helmet			In general, we collected very positive feedback from all users.		In terms of user experience, the emotional reactions to the product were very positive. Only 2 negative emotions were expressed against 20 positive ones. The ergonomics and usability of the helmet were positively assessed. Comfort was described as high by all users, but for two elements - the HMD adaptability and the weight of the system. One of the main benefits mentioned by users was the integration of different PPE in a single device. Users declared that embedding gas and noise detectors, as well as the visor and hearing protections in the helmet would make it impossible to forget or lose them, thus reducing cognitive effort and stress. Functionalities.Difficulties connected to the DVSfunctionalities mainly concern the effort required for their implementation, including the creation of Wi-Fi infrastructures or the generation of digital maps for entire plants.Two elements of discomfort were identified in the DVS. The first one concerns the adaptability of the HMD. The second, and most important concern, refers to the weight of the system.		Users stressed the need to balance technology advancements with the comfort that PPE requires. For instance, the HMD could be used to navigate users out of the plant in case of emergencies, directing them through the safest route, which can change from case to case. Additional suggested uses include training, remote assistance, or the use of the alert system to warn workers who try to access restrictedareas, such as offshore platforms or scaffoldings, without the required training certificates. These use cases are not all connected to health and safety procedures, but still confirm the usefulness of a similar system for different applications in the workplace.			Functionalities. According to users, the augmented helmet improves exposure monitoring and alert, communication, hazard report and plant navigation, compared to current solutions (Fig.9). The hands- free communication system was perceived as extremely beneficial especially when workers have their hands occupied by operating on equipment. Moreover, such a system allows users to communicate with each other also while climbing stairs, which requires to held the stair handrail, or while working at height, with no risk to drop the radio.	Another concern we collected regards the potential loss of human skills connected to the use of certain technologies. User Experience. Doubtwas mainly connected to the foreseen difficulties in the systemimplementation and to the uncertainty about the possibility to pass all the required certifications (especially the ones concerning explosion risk).	UNDECLARED

Examining and Promoting Explainable Recommendations for Personal Sensing Technology Acceptance	2022	Proc. ACM Interact. Mob. Wearable Ubiquitous Technol.	Newn, Joshua and Kelly, Ryan M. and D'Alfonso, Simon and Lederman, Reeva	10.1145/3550297	This paper explores the influence of explanations on the acceptability of recommendations based on personal sensing.	Qualitative	Unspecified	General public	Our sample consisted of 25 participants (14 women, 10 men, 1 non-binary). Participants had an average ageof 30.6 years (Range= 20–78,S.D.= 13). Eighteen were students at either undergraduate or postgraduate level,and the remaining 7 were in full-time employment. One of the student participants was retired from full-timeemployment, and two others were professionals furthering their studies (e.g.on a medical placement). Thirteenparticipants owned an Android-based smartphone and 12 owned an iPhone	Personal Sensing Technology Acceptance	We asked participants about their knowledge of the sensors in their smartphone on a 5 point scale (1 =Not atall aware, 5 =Extremely aware) during recruitment. Participants responded at the following frequencies: Notat all aware (N= 2), Slightly aware (N= 6), Somewhat aware (N= 9), Very aware (N= 5), Extremely aware (N= 3). We also asked participants if they had previous experience using health-based apps and wearables. Themost common was Headspace for meditation, wearable technologies like Fitbit, and apps like WorryTime (tomanage anxiety). The majority of participants mentioned that they were most aware of the built-in apps on theirsmartphones (i.e.Apple Health and Samsung Health), which gave them insights into their daily activity and		Our overall findings suggest that participants were optimistic about recommendations based on personal sensingtogether with the fact that they could be explained.				Personalisationcan easily be integrated through user interface design and device integration. Appropriate phrasingshould be co-designed with professionals and users using iterative methods. As a first step,personal sensing that produces recommendations could adopt a lighter, less direct phrasing, with a positive tone,as suggested by participants. Adaptive capabilityis dependent on how well the system can keep track of the users' interactions, how well itcan analyse the users' patterns, and the outcome of the adaption in relation to the users' mental health and well-being (e.g.how practical are the recommendations given the users' state, or how well is the system designed forbehavioural activation). Peer Endorsementrelies on other users using the system. Hence, as more users engage with the system as itsacceptability increases, its use will become more normalised, resulting in more users using and contributingtowards peer endorsement. However, because privacy is a major barrier to personal sensing, we must consider its privacy implications, such as anonymisingusers.				This need for understandingand control may be because privacy remains one of the primary concerns for personal sensing [44, 78, 99].	UNDECLARED
Privacy Risks Emerging from the Adoption of Innocuous Wearable Sensors in the Mobile Environment - Proceedings of the SIGCHI Conference on Human Factors in Computing Systems	2011		Raij, Andrew and Ghosh, Animikh and Kumar, Santosh and Srivastava, Mani	10.1145/1978942.1978945	We conducted a study to assess how concerned people are about disclosure of a variety of behaviors and contexts that are embedded in wearable sensor data.	Quantitative	USA	General public	MalesAge±StdAPBWONS3656%25±422%22%53%3%S3053%23±423%33%40%3%	innocuous wearable sensors in the mobile environment											First, our results indicate people cannot understandthe potential threats in the data unless they have a personalstake in it. Of the behaviors and contexts we examined, peo-ple were most concerned about revealing conversation, com-muting, and inherently private psychological states (in thisstudy, stress). Second, adding physical and temporal con-text increases concerns about the data, but those increasescan be mitigated through restriction and abstraction. Third,people are willing to share such data - even with their iden-tity - with us, other study participants, and other researchers.However, participants have significantly more concerns re-garding sharing with the general public (e.g., over the web),especially when released with identity	UNDECLARED
Users Can Deduce Sensitive Locations Protected by Privacy Zones on Fitness Tracking Apps - Proceedings of the 2022 CHI Conference on Human Factors in Computing Systems	2022		Mink, Jaron and Yuile, Amand a Rose and Pal, Uma and Aviv, Adam J and Bates, Adam	10.1145/3491102.3502136	This work presents an online user study (N=603) that investigates the privacy concerns of fitness tracking users and evaluates the efficacy of privacy zones.	Mixed	USA	General public	Participants ranged from 18-74 with the median age between 30-34; 62% of respondents identified as male and 37% as female.	Fitness Tracking Apps							Privacy Precautions. Among participants that had used a fitness app, 51% reported haven taken active steps towards protecting their privacy in some form, 28% had not taken any privacy-oriented steps, and 21% were unsure (Q6). Phone-level defenses included placing restrictions on the fitness app's access to location data or the Internet. 17% (n=37) of participants limited or were faking the information they provided to the fitness app. Other participants took additional actions (10%, n=23) such as monitoring their social media for accidental disclosure of information or sharing via alternative methods such as screenshots and text messaging. 8% (n=18) also discussed ways they ensured protection through access data control mechanisms such as authentication or secure communication.			Some participants (15%, n=46) mentioned the benefits of using fitness apps, most notably the social support that sharing an exercise provides. One participant (P112) said, "I want others to see my progress, keep me motivated in reaching my goals, and to continue to keep me accountable".	level of unease when sharing exercise maps online (Figure 5, Q4). 53% of participants reported that they were somewhat or very uncomfortable sharing, while 45% were somewhat or very comfortable. When asked to explain the reason behind these preferences (Q5), 55% (n=165) of coded participant responses (see Qualitative Response Coding) expressed concerns that included, most commonly, the risk of their location being revealed. Participants also mentioned peer judgement, being part of a vulnerable demographic group, and lack of trust in their friends on social networks as concerns.	UNDECLARED
Alexa Depression and Anxiety Self-Tests: A Preliminary Analysis of User Experience and Trust - Adjunct Proceedings of the 2020 ACM International Joint Conference on Pervasive and Ubiquitous Computing and Proceedings of the 2020 ACM International Symposium on Wearable Computers	2020		Quiroz, Juan C. and Bongolan, Tristan and Ijaz, Kiran	10.1145/3410530.3414374	We present the pilot study outcomes of an Alexa Skill that allows users to conduct depression and anxiety self-tests.	Quantitative	Australia	General public	The average age of the participants was 21.6 years old and 8/10 weremale. 9/10 of the participants used conversational agents. 7/10 of theparticipants also indicated that they had not previously used an appfor mental well-being. After the two-week period, 7/10 participantsindicated that they rarely used our Alexa Skill	Alexa depression and anxiety self-tests			The participants found the app's attractiveness, dependability, andstimulation to be above average, but attractiveness, perspicuity,efficiency, and novelty were scored below average. We conclude, participants mostlytrusted the app									UNDECLARED
User Perceptions of Smart Home IoT Privacy	2018	Proc. ACM Hum.-Comput. Interact.	Zheng, Serena and Apthorpe, Noah and Chetty, Marshini and Feamster, Nick	10.1145/3274469	In this study, we conduct eleven semi-structured interviews with smart home owners, investigating their reasons for purchasing IoT devices, perceptions of smart home privacy risks, and actions taken to protect their privacy from those external to the home who create, manage, track, or regulate IoT devices and/or their data. We note several recurring themes.	Qualitative	USA	smart home owners	There were six female and fivemale participants ranging from 23–45 years old. The majority of the households were from theSeattle metro area. Other households were from New Jersey, Colorado, and Texas. Participantscame from a variety of living arrangements, including families, couples, and roommates.Our participants were fairly affluent, technically skilled, and highly interested in new technology,fitting the profile of an "early adopter" [44]. Seven of the eight households had at least one residentwith a background in computer science or the technology industry, including one participant whowas an expert on IoT smart home devices. We had difficulty finding households who did not fitthe early adopter profile due to the relatively recent introduction of IoT smart home devices to themass market at the time of the interviews. While we would have liked to recruit a more diversesample, we were unable to do so	Smart Home IoT Privacy												First, owners of smart homesvalue convenience and connectedness, which dictate their privacy expectations and behaviors.Second, their opinions about who should have access to their smart home data depends on theperceived benefit. Third, trust in device manufacturers and brand reputation dictates purchasingbehavior and privacy assumptions. Finally, users are skeptical of privacy risks from non-audio/visualdevices	UNDECLARED
It Feels Like I'm Managing Myself: HIV+ People Tracking Their Personal Health Information - Proceedings of the 9th Nordic Conference on Human-Computer Interaction	2016		Bussone, Adrian and Stumpf, Simone and Buchanan, George	10.1145/2971485.2971542	Our paper introduces an iterative process model of Personal Health Informatics. We then describe the results of an empirical study involving HIV+ adults aimed at understanding their issues, concerns and actions in each of the stages of this process model.	Qualitative	UK	HIV+ People	[illegible] living with HIV in England through sexual health organizations, social media, and online forums:8 male and 8 female. The ages of these participants ranged from 18 to 57 years old, with an average age of 39.87. Fourteen of our participants identified as Black, Asian, orminority ethnic; nine identified as heterosexual. Participants varied in their time since diagnosis, from two participants living with HIV for less than a year to 25 years.Comparing this breakdown with that of the larger population described in Related Work, our sample is skewed against the national average by including proportionally more heterosexual Black African women, however, despite increasing diagnosis rates in this segment, this group is rarely involved in studies of this nature [19]	HIV+ People Tracking Their Personal Health Information						Seven participants tracked with the intention of acting upon their health information, using their collection to inform what actions should be taken or observe the effects of them. While behavior change is a common focus of PHI research, less than half of our participants had this intention. However, this also demonstrates that participants, in order to inform how tochange their behaviors to improve their health, needed to tracka number of different data types, and then to relate them together. Fifteenparticipants had the intention of monitoring their data. In contrast to what has been found for other conditions, our participants often needed to monitor some health data at the same time as actively managing their health. Seven of across the sixteen participantsparticipants had both monitoring and acting as their intentions.	[illegible] integrated, or transferredtheir data;suggesting that these are essential functions that need to be integrated into PHI tools designed for people living with HIV. First, we found that the perception of security influences the chosen tool for storing captured data. Second, because of the far-reaching effects of HIV, a broad range of data is captured. Security Security refers to the safety of a user's personal information, and the measures taken to protect it against unwanted access [2]. The statements by participants in our study showed that security is particularly important to HIV+ individuals because of the need to protect their status and, as such, protect themselves from discrimination and stigmatization.Our participants show that the selection of a tool to track information is largely based upon the perceived security of the tool, although additional security measures are also put in place to protect their data from unwanted access. This need for heightened security emerges when examining the choices our participants made to store tracked information in a genericstorage tool over a specificone. Specific storage was used by four, and these were storage tools that were intentionally designed for HIV+ people. The remaining twelve participants used generic storage, such as notebooks or general health applications.			people living with HIV need to track a large amount of highly varied information in order to form a better view of how their health is changingand monitor existing conditions. Nearly all of this information is self-reported into generic tools; one reason for this is the need for tools that are secure and covert.		UNDECLARED
Functional Privacy Concerns of Older Adults about Pervasive Health-Monitoring Systems - Proceedings of the 10th International Conference on PErvasive Technologies Related to Assistive Environments	2017		McNeill, Andrew and Briggs, Pam and Pywell, Jake and Coventry, Lynne	10.1145/3056540.3056559	We conducted interviews with 20 older adults (10 males, 10 females, mean age=73) to ask, under what health and wellbeing circumstances would they wish to protect their privacy.	Qualitative	UK	older adults	females who were all over sixty years of age and all lived in the north-east of England. Twelve participants had been involved in previous studies conducted within the project and so had prior knowledge of the project and the system.	privacy concerns of older adults about pervasive health-monitoring systems											we identified seven reasons why older adults want privacy in the context of a proposed system to improve the well-being of older adults.	UNDECLARED
Experiences of Accessibility and Mobility among Powered Wheelchair Users through Video and Data - Proceedings of the 2019 CHI Conference on Human Factors in Computing Systems	2019		Rodger, Sunil and Jackson, Dan and Vines, John and McLaughlin, Janice and Wright, Peter	10.1145/3290605.3300860	Recent HCI research has investigated how digital technologies might enable citizens to identify and express matters of civic concern. We extend this work by describing JourneyCam, a smartphone-based system that enables powered wheelchair users to capture video and sensor data about their experiences of mobility. Thirteen participants used JourneyCam to document journeys, after which the data they collected was used to support discussions around their experiences.	Mixed	UK	Wheelchair Users	Participants were aged 19 to, by their own expla-nation, "over 60". While some had been powered wheelchairusers their whole lives, others had previously been ambulantor used manual wheelchairs	JourneyCam: Exploring Experiences of Accessibility and Mobility among Powered Wheelchair Users through Video and Data			They viewed it as a worthwhile endeavour, unan-imous in feeling it would be useful to capture a representa-tion of their experiences of mobility, and ascribing particularvalue to the visual nature of JourneyCam data									UNDECLARED
Towards Better Transitions for Children with Diabetes: User Experiences on a Mobile Health App - Proceedings of the 18th ACM International Conference on Interaction Design and Children	2019		Shin, Ji Youn and Holtz, Bree E.	10.1145/3311927.3325319	This study, part of a larger study, comprised of three phases: mobile app development, prototype feasibility testing, and usability testing with child-parent dyads users.	Qualitative	USA	Children with Diabetes and parent	The study participants include 24 childrenbetween 10 and 15 years old with T1D and their mothers. Fifteen of the children were between 11–13 years old, and 13 children had been diagnosed for five or more years. Among parents, 18 were the biological mothers, and 11 were between the ages of 35–44 year	Children with Diabetes: User Experiences on a Mobile Health App			Overall, the study participants reported that the app was helpful, allowing them to conveniently track their BG values and generate a short and simple message (See Figure 3).				Need to provide tailored support based on illness stages Benefits of providing a calibrated mode of rewardsOne of the most frequently occurring themes during the interview was the positive impressions on the reward system in the app (See Figure 4). Adopting peer support functions Other participants interviewed expressed the necessity of having peer support components. For many families, it is difficult to find peer groups in their area (especially rural areas) to provide support and reduce feelings of isolation.	However, at the same time, regular monitoring of BG and communication with parents were challenged by many unexpected situations at school. These issues included school activities that happened out of their routine which hindered keeping BG monitoring as their natural routine, technical issues with wi-fi connections, and the school's phone regulation policy regarding students.		Participants also mentioned a sense of connection which was evoked by the app use even when they were not together. It allowed children and their parents to know their BG level without spending time and efforts, and it could tell whether the number was within the recommended range.		UNDECLARED
Ageing is Not a Disease: Pitfalls for the Acceptance of Self-Management Health Systems Supporting Healthy Ageing - Proceedings of the 21st International ACM SIGACCESS Conference on Computers and Accessibility	2019		D'Haeseleer, Ine and Gerling, Kathrin and Schreurs, Dominique and Vanrumste, Bart and V and en Abeele, Vero	10.1145/3308561.3353794	Recently, a shift from curative to preventive care is promoted, by which patients are expected to become active and use diverse forms of self-management health systems (SMHS), i.e., integrated solutions that present data from multiple sensors and/or self-reports, possibly enhanced with risk assessment and decision support, to perform health-related actions. This is promoted as contributing to living longer and healthier, assisting ageing-in-place. Hence, older adults have also become a potential target of SMHS. While studies are performed on uses and attitudes of specific patient groups towards SMHS, studies that focus on older, including the oldest, adults are scarce. Therefore, we report on a qualitative study with 20 older adults (mean age = 80).	Qualitative	Belgium	older adults	All participants were at least 65 years old, ten were older than 80 (mean age 80, range 65–92 years). All were community dwelling older adults – they still lived independently, either at home (n=11) or in a service flat (n=9). During each focus group, two or three researchers and a moderator were present	Self-Management Health Systems Supporting Healthy Ageing			[illegible] most of these systems were not currently in use, e.g., none of the participants who had an alarm bracelet was wearing this during the focus group discussion. AmbivalentattitudestowardsfutureuseofSMHS.Despite the non-use of alarms and activity trackers, all participants agreed that is was important to stay healthy and to be able to age in place. However, older adults were divided regarding their attitudes towards use of SMHS. About half of the par-ticipants showed an overall positive attitude towards SMHS. The other half of the participants showed a more negative attitude towards SMHS and asserted that they did not see the added value.				[illegible] importance of getting support when using (new) technologies. Especially children and grand-children play an important role for older adults to overcome the barriers that technologies present to them. The partic-ipants in this sample strongly depended on their (younger) relatives. These relatives played an important role in the lives of the older adults, to provide access to technology. They also helped introducing new technologies and assisted them in buying new technologies. Overall, older adults did not seem to mind this dependency on friends and relatives, as perhaps this provided a chance for social encounters.	They indicated they did not want to use such a system, as they did not want to know how they were doing. Taking such measurements could even make them unnecessarily worried. Moreover, also participants who did make an effort to stay up with ICT, voiced concerns over the incessant technological progress, and their lack of ability to keep up.			In this context, privacy was something that participants mentioned as important and it was emphasised particularly in the context of social media such as Facebook.	UNDECLARED
Danger vs Fear: An Empirical Study on Wearable Users' Privacy Coping - Proceedings of the 2020 on Computers and People Research Conference	2020		Baskaran, Krutheeka and Mathew, Saji K.	10.1145/3378539.3393856	With the arrival of Internet of Things, the magnitude of data shared through various devices has seen a drastic increase. Fitness trackers contribute to a significant part of this phenomenon. Increase in collection of individual's information has led to increase in privacy issues like identity theft and targeted marketing. This study treats information privacy as a distinct dimension and develops a model, drawing from extant privacy concern literature, Extended Parallel Process Model and coping theory to understand an individual's coping behavior when faced with a privacy breach to their user generated health data.	Quantitative	India	General public	A total of 285 subjects participated (40 percent response rate), with 225 producing complete and non-redundant data. Approximately 51 percentage of the respondents were female and 49 percent male. 49 percent were in the age bracket of 18 to 30, reflecting a sample of active users being young.	Wearable Users											H1 was supported i.e. increased privacy concern leads to increase in threat perception when confronted with a fear appeal to data privacy breach. The results also showed that women were more perceptive to threat than men and men had higher efficacy perception than women. Interestingly, people with an undergraduate education background chose problem focused coping than those with higher levels of education. Those with education level lower than under graduation chose emotion focused coping. It is also seen that since efficacy has a positive effect on problem focused coping for certain age groups enhancing the effectiveness of the proposed protection measures in the message will greatly help to improve the numbers of people who will be motivated cope rationally	UNDECLARED
Acceptance and Sustainability of Health Promotion Solutions for the Elderly in Taiwan: Evidence from SHI-LIN Elderly University in Taipei - Proceedings of the 12th ACM International Conference on PErvasive Technologies Related to Assistive Environments	2019		Wu, Irene Yipei and Yu, Ying and Cheng, Shu-Jung and Tu, Wei-Ju and Sung, Tung-Jung	10.1145/3316782.3321529	In this research, following the first phase research for identifying three senior participants' group namely as "Active Health," "Confident Health" and "Carefree Health" with different lifestyles, through workshop with semi-structured questionnaire, we analyze the acceptance and sustainability of three health promotion solutions, namely, S1 (10-minute exercise), S2 (smart bracelet) and S3 (health chatbot).	Mixed	Taiwan	older adults	Table 3. The profile of subjectCodingAgeEducation Habit Single exercise durationAA01 78 UndergraduateWalking, Runningover 60 mins A0272DiplomaReading,Cooking30-60 mins A0372DiplomaWalking, Dancing over 60 minsA0468UndergraduateNone30-60 mins A05 72 UndergraduateMusic, Photography, Ball30- 60 mins BB01 64 GraduateDancingover 60 mins B02 64 GraduateNone 30-60 mins B03 63 UndergraduateSinging, Dancing30-60 mins B04 65 UndergraduateReading, Dancing 30- 60 mins B05 60 UndergraduateBall ing over 60 mins CC0166UndergraduateTravel30-60 minsC02 72 UndergraduateMountain Climbing, Qigong30- 60 mins C03 63 UndergraduateCalligraphy, Travel30- 60 mins C0472UndergraduateNone30-60 minsC0570DiplomaQigong30-60 mins	smart bracelet						For Group A, "professionalism" is what drives their motivation. As for the needs of "professionalism," they incline towards being accompanied by professional coaches or instructors, and having professional practitioners providing them information. Also, Group A are more self-demanding, and emphasize ideals or personal beliefs. With continued "Spark" and "Signal," combined with provision of services through professional contents and means, Group A will continue to trigger themselves and enhance personal healthy behaviors.As for elderly people of Group B, they are mainly driven by affect. As for the type of exercise, they hope to be engaged in mainly group sports. They focus more on interaction and exchange within a team. According to FBM, when designing health promoting solution, the researchers perhaps can enhance emotional stimuli that trigger joy and happiness, to continue encouraging Group B to be engaged in health promoting activities. In this experiment, Group C has little consensus on the three types of solution, and their opinions are more widely distributed. However, the subjective consensus is that they do not like to feel burdened or constrained		As for Group C, they feel burdened or constrained during health promoting activities especially mentioning smart bracelet				UNDECLARED
Exploring Perceptions of Cross-Sectoral Data Sharing with People with Parkinson担 - Proceedings of the 2022 CHI Conference on Human Factors in Computing Systems	2022		McNaney, Roisin and Morgan, Catherine and Kulkarni, Pranav and Vega, Julio and Heidarivincheh, Farnoosh and McConville, Ryan and Whone, Alan and Kim, Mickey and Kirkham, Reuben and Craddock, Ian	10.1145/3491102.3501984	In this study we collected a simulated free-living dataset, in a smart home, with 12 participants (six people with Parkinson, six carers). We explored their initial perceptions of the sensors through interviews and then conducted two data exploration workshops, wherein we showed participants the collected data and discussed their views on how this data, and other data relating to their Parkinson symptoms, might be shared across different sectors.	Qualitative	Unspecified	PD patient	Within theParkinson's group, we had three males, three females with an agerange of 58 to 73. The range of years since diagnosis was betweenone and a half and nine (mean= 5.3 years) and all participantswere between Hoehn and Yahr stages one (unilateral symptomsonly) and two (bilateral symptoms with no impairment of balance)[43].	smart home	Participants were asked about their very early perceptions of thesmart home sensors (before they had even arrived at the house) andwhether they had any preconceived ideas of what the monitoringenvironment and sensors were going to be like. Several of theparticipants had no idea what to expect when coming into thesmart home environment.		Participants had very similar perspectivesand were largely positive.		As expected, participants were primarily concerned about thecamera-based sensor. All participants considered the sensors to be unobtrusive.The majority of participants' concerns with using the sensorslonger term centered around the wearable, and possible challengeswith either remembering to put it on:or whether ornot the devices would be resilient enough to fit in with some of theireveryday activities, such as working out, swimming and showering.	When discussing data sharing, par-ticipants began with a familiar narrative that they would be happyto share all of their data for 'the greater good' of the Parkinson'scommunity		.The majority of participants' concerns with using the sensorslonger term centered around the wearable, and possible challengeswith either remembering to put it on:or whether ornot the devices would be resilient enough to fit in with some of theireveryday activities, such as working out, swimming and showering.		The collection of sen-sor data was not only seen to be beneficial to researchers, but also tothe participants themselves, by providing a way to support commu-nication with a clinician during appointments.	transparencyover how their data might be used. Within the context of data use and misuse,participants extensively discussed targeted advertising from com-mercial companies. Participants were very aware of the fact thatmajor technology companies collected and used their data. Even in the case of reputable drugs companies, there was a senseof mistrust from participants due to their perception of"the way theybehave	UNDECLARED
Determinants of Patients' Intention to Adopt Diabetes Self-Management Applications - Proceedings of the 15th New Zealand Conference on Human-Computer Interaction	2015		Maniam, Ananthidewi and Dhillon, Jaspaljeet Singh and Baghaei, Nilufar	10.1145/2808047.2808059	The aim is to develop a research model to represent the adoption of Diabetes Self-Management Applications (DSMA) amongst diabetics in Malaysia.	Quantitative	Malaysia	DIABETES PATIENTS	living in Malaysia (mean age 52, SD = 8.22). With respect to demographics, there was a good balance of distribution (almost 50%) for both males and females among the respondents. Seventy five out of 105 respondents (71.43%) completed secondary level education and majority of the respondents (43.81%) had Type 1 diabetes. Table 5 shows characteristics of the study participants	Patients' Intention to Adopt Diabetes Self-Management Applications				The findings indicate that Perceived Financial Risk, Perceived Privacy and Security Risk, Technology Anxiety and Facilitating Conditions have significantly positive relationship with the intention to adopt DSMA.								UNDECLARED

Are Those Steps Worth Your Privacy? Fitness-Tracker Users' Perceptions of Privacy and Utility	2022	Proc. ACM Interact. Mob. Wearable Ubiquitous Technol.	Velykoivanenko, Lev and Niksirat, Kavous Salehzadeh and Zufferey, No\'{e} and Humbert, Mathias and Huguenin, K\'{e}vin and Cherubini, Mauro	10.1145/3494960	Fitness trackers are increasingly popular. The data they collect provides substantial benefits to their users, but it also creates privacy risks. In this work, we investigate how fitness-tracker users perceive the utility of the features they provide and the associated privacy-inference risks.	Mixed	Switherland	General public	The distribution by gender was 63.0% female, 36.6% male, 0.4% prefer not to answer. Women are over-represented in our sample, compared to the general population. Note that our sample is consistent with the market trend of women being the majority of fitness-tracker users [84]. The ages of the participants ranged from 18 to 33, with an average of 21.62 and a standard deviation of 2.57. The distribution of the major fields of studies was quite diverse	fitness-tracker	Our findings show that the participants are aware that some types of information might be inferred from the data collected by the fitness trackers. For instance, the participants correctly guessed that sexual activity could be inferred from heart-rate data. However, the participants did not realize that also the non-physiological information could be inferred from the data.Furthermore, we identify the participants' lack of understanding and common misconceptions about how the Fitbit ecosystem works.						The two most common actions were changing the privacy settings in their accounts (22.0%) and changing the security settings in their accounts (13.7%). During the interviews, we asked participants whether they were aware of the existence of such options within the Fitbit app and/or website. Most of them said that they were not aware of the existence of the options/functions				[illegible] participants (58.1%) think that they are at least slightly aware of the privacy risks associated with fitness-trackers. Age and gender were the attributes that participants were the least worried about, wherein 68.7% of the participants said they were not at all worried about their age being inferred and 71.8% said they were not worried about their gender being inferred. Hence, the participants' willingness to create one to participate in the study is another indicator that they are not worried about them. Surprisingly, many participants reported not being worried about if their sexual orientation or religion could be inferred.	UNDECLARED
Respiratory Self-Care: Identifying Current Challenges and Future Potentials for Digital Technology to Support People with Chronic Respiratory Conditions - Proceedings of the 13th EAI International Conference on Pervasive Computing Technologies for Healthcare	2019		Tendedez, Helena and Ferrario, Maria-Angela and McNaney, Roisin and Whittle, Jon	10.1145/3329189.3329190	Chronic respiratory conditions (CRCs) are life-long diseases affecting millions of people worldwide. They have a huge impact on individuals' everyday lives, resulting in a number of physical and emotional challenges. Self-management interventions for CRCs are thought to provide empowerment and improve quality of life. In this paper, we contribute to the literature by investigating the experiences and everyday challenges faced by those with CRCs, through the involvement of 156 participants via interviews and an anonymous survey.	Mixed	UK, Ireland, Europe, Asia, USA, Canada	People with Chronic Respiratory Conditions	Age, gender and CRCs of respondents.Age groupGenderCRC50 or under16Female98COPD9351 to 6020Male47Asthma4961 to 7058Other2Emphysema4371 to 8043Bronchiectasis2981 plus8Bronchitis6Other20	Digital Technology to Support People with Chronic Respiratory Conditions	[illegible] used some form of technology for self- management or self - monitoring either currently or in the past: 14% had used a dedicated mHealth app, 56% had used a pulse oximeter (a small digital device which monitors the oxygen saturation in people's blood), 12% had used wearable technology such as smart watches and 10% tried other ways such as telehealth, tablet apps and digital spirometers (a device that measures lung function.)		Taking prescribed medication, pulse oximeter readings and following exercise plans were the most popular techniques tried by respondents. Keeping a diary of symptoms and reflecting was the least popular technique.			participants adopt a reactive approach to self-managing; (4) self-monitoring technologies should link to support networks; (5) those at different stages of their condition will find value in different self-management tools.						UNDECLARED
Investigating Culturally Responsive Design for Menstrual Tracking and Sharing Practices Among Individuals with Minimal Sexual Education - Proceedings of the 2022 CHI Conference on Human Factors in Computing Systems	2022		Lin, Georgianna E and Mynatt, Elizabeth D and Kumar, Neha	10.1145/3491102.3501824	We investigate menstrual tracking and data-sharing attitudes and practices in educated, young (20-30 years old) menstruating individuals based in the United States, with self-identified minimal menstrual education backgrounds.	Mixed	USA	Individuals with Minimal Sexual Education	Wecollected62completeresponsesfromparticipantswhowereages18to50with84%oftherespondentsfallinginthe20-30agegroup.89.7%oftherespondentshadexperiencedamenstrualcycleatsomepointintheirlifeandofthose19.2%have/hadanirregularmenstrualcycleand5.4%weretransitioninginto,in,orhavegonethroughmenopause.82.1%,oftherespondentstracktheirperiod(seeTable1).Ofthe7participantswhoidentifedthemselvesasmale,oneexperiencedmenstruationandtrackedtheircycle,whiletheothersixmadeupthenon-menstruatingindividualsinoursurvey.Thesesixidentifedthemselvesaspartnersorfriendstomenstruators	Menstrual Tracking and Sharing Practices Among Individuals with Minimal Sexual Education					wefndthatexistingmechanismsfortrackingandsharingdataonmenstruationarenotadequatelyresponsivetotheneedsofthosewhoseekrelevantmenstrualeducation,arenotinthesexualmajority,and/orwishtocustomizewhatmenstrualdatatheyshareandwithwhom.Ouranalysishighlightsadesigngapforparticipantswithminimalsexualeducationbackgroundswhowishtobetterunderstandtheircycles.Wealsocontributeadeepenedunderstandingofstructuralhealthinequitiesthatimpactmenstrualtrackingandsharingpractices,makingrecommenda-tionsfortechnology-mediatedmenstrualcare	Manycitedthephone'smobileandreminderabilitiesforusingtheirphonetotrack.	Morespecifcally,participantsdesiredadesignthatisportable,easytointegrateintotheirpreexistingschedules,readilyadoptable,doesnotinvolveintrusivesensors,andoperableonmultipleplatforms(e.g.,awearableandanaccompanyingapp).					UNDECLARED
Confidence & Control: Examining Adolescent Preferences for Technologies That Promote Wellness - Proceedings of the 19th ACM Conference on Computer-Supported Cooperative Work & Social Computing	2016		Bisafar, Farnaz Irannejad and Parker, Andrea Grimes	10.1145/2818048.2820028	Our work contributes to the growing body of CSCW research examining how technology can encourage wellness. In a 10-week participatory design study, we examined how technology can help teens overcome intra-personal and social barriers to healthy eating and positive relationships (the wellness topics of greatest interest to our participants).	Qualitative	US	Adolescent	We had 15adolescents in our study (four boys and 11 girls). Seven students were African American and eight were Latino	informationaland monitoringdevices. As informational devices, participants developed technology ideas that served as companions for users telling them what to eat and what not to eat. One group conceived of a pocket-sized robot that would provide healthy recipes, and another group designed a device that entered into dialogue with the students, answering their questions about what to eat. The second group of technology ideas supported diet monitoring and provided tangible assistance to students. One of these ideas was an augmented jacket that would automatically count their calories. Another concept was a device that used charts to visually display a user's food intake on the refrigerator. We infer that these monitoring devices would help							Our findings revealed teens' desire forexpressive technology that helpsthem initiate dialogue, negotiate conflicts,and restrict communication with family, and their desired degree of engagement with tools promoting healthy eating (passively toactively involved in behavior change).					UNDECLARED
Development and Evaluation of an Exergaming Application for Improving Seniors' Well-Being - Proceedings of the 20th Pan-Hellenic Conference on Informatics	2016		Kostaki, Chaido and Goumopoulos, Christos	10.1145/3003733.3003736	We have developed an application that monitors the body posture and balance of seniors during exercise games (exergames) using a low cost gaming device. The aim of the system developed is to improve the well-being of seniors, especially in terms of body balance.	Mixed	Greece	older adults	For theevaluation of the application an occasional sample of 10 individuals(3 male,7 female) 61to 80 years old (mean=72,SD=5) was assembledfromthe local community ofthe city ofKomotini.	We have developed an application that monitors the body posture and balance of seniors during exercise games (exergames) using a low cost gaming device.			Finally, 90% of the participants indicatedthat they will definitely use the application again.		[illegible] ofapplication components (text, images, audio, and video). They also consider sufficient (100%)the amount of information on the screen and the contentof thefeedbackmessages given bythe application. The overall assessment of the application is considered to be excellent (60%) or very good (40%). Regarding accessibility 80% of the participantsconsider that itis an easy applicationto usewith 20% rating with average accessibility. Regarding the PACES subsection participants reported ahighlevel of enjoyment . Positive topics were an improvement in balance self-confidence following the program,a sense of being exercised and entertained at the same time and positivity about the technology used. A negative topic identified was the worries on getting along when more difficult exercises were introduced.							UNDECLARED
Supporting Collaborative Health Tracking in the Hospital: Patients' Perspectives - Proceedings of the 2018 CHI Conference on Human Factors in Computing Systems	2018		Mishra, Sonali R. and Miller, Andrew D. and Haldar, Shefali and Khelifi, Maher and Eschler, Jordan and Elera, Rashmi G. and Pollack, Ari H. and Pratt, W and a	10.1145/3173574.3174224	In this paper, we discuss hospitalized patients' needs for collaboratively tracking their health with their care team.	Qualitative	USA	patient	[illegible] 12 pediatric patients. Because caregivers play a critical role in pediatric settings and our goal in this study was to get a broad sample rather than to distinguish patients from caregivers, we recruited 3 adult caregiver participants (Y13, Y14, and Y15). Five of the pediatric patients also had caregivers present during the interview. Pediatric patient participants ranged in age from 7 to 17 (mean and median age 10.5). At Virginia Mason, we recruited 15 patients, who ranged in age from 18-75 (mean age 57.5, median 64). Across both groups, 15 participants were male and 15 female. Participants were recruited across services and conditions to get a broad sample of experience	Health Tracking in the Hospital			participants envisioned a world where patients and clinicians could collaboratively track the patient's health. They described this vision in terms of both the tracking activities that they currently performed and the tracking activities that they wanted to perform.									UNDECLARED
Digitization for Fun or Reward? A Study of Acceptance of Wearable Devices for Personal Healthcare - Proceedings of the 21st International Academic Mindtrek Conference	2017		Rajanen, Dorina and Weng, Min	10.1145/3131085.3131118	We examine the factors predicting behavioral intention to use wearable devices for personal healthcare with the aim to understand the role of personality characteristics in adopting digital life technology.	Quantitative	China	General public	A number of 158 respondents answered thesurvey. We retained for analysis only the subjects living in China,namely 156 (88 female, 68 male). The age of participants rangedfrom 18 to 54 (82.7% were between 18 to 29 yrs. old). They werehighly educated with 92.3% of them having graduated fromcollege or higher education	wearable devices for personal healthcare			Our data show that smart bands adoption and abandon rates of22% and 53%, respectively, are similar with prior reports on otherpopulations (e.g., [10,15,16]) indicating that the smart bands arenot yet adopted at large scale; in our sample, only 10.3% ofrespondents were current users. Moreover, 28% of the past usersreported they did not intend to use smart bands in the future	[illegible] social influence,affinity, and compatibility have a significant direct positive effecton the acceptance of smart bands. This resultmeans that higher trust and higher perceived ease of use arepredicting higher perceived usefulness, which in turn positivelyaffects the smart bands acceptance. The data show also that two personality disposition traitssignificantly moderate the influence of perceived ease of use andperceived usefulness on acceptance. Fun seeking behavioralmotivation moderates inversely the effect of ease of use onbehavioral intention to use smart bands; Rewardresponsiveness moderates positively the effect of perceivedusefulness on acceptance; people with stronger rewardresponsiveness personality are likely to				.These statistics show that understanding better the usage driversand users' perceptions on smart bands and digital life technology,in general is important in order to advance the development ofthese devices towards a better customer satisfaction. Comparingpast and current users with respect to their demographic andpersonality profile, we found that only compatibility scores weresignificantly different in the two groups; the users who abandonedthe smart bands reporting generally a lower compatibility score.This means that people who do not regularly use mobile or webtechnology for health-related searches or applications are morelikely to abandon the smart bands for personal healthcare				UNDECLARED
Can Workers Meaningfully Consent to Workplace Wellbeing Technologies? - Proceedings of the 2023 ACM Conference on Fairness, Accountability, and Transparency	2023		Chowdhary, Shreya and Kawakami, Anna and Gray, Mary L and Suh, Jina and Olteanu, Alex and ra and Saha, Koustuv	10.1145/3593013.3594023	This paper unpacks challenges workers face when consenting to workplace wellbeing technologies.	Qualitative	USA, UK, Canada,and India	worker	P#FG#CountryGenderRaceAgeRoleOrg. Years P1FG1USManAsian25-34Researcher-5-7Ys. P2FG1U.S.WomanWhite25-34ProgramManager/Legal10K+1-3Ys. P3FG1CanadaManAsian25-34SoftwareDeveloper10K+1-3Ys. P4FG2IndiaWomanAsian25-34PolicyOfficer1to493-5Ys. P5FG2Canada--25-34AIEthicsConsultant-1-3Ys. P6FG3U.S.WomanWhite35-44ContentDesigner50-1K3-5Ys. P7FG3U.S.WomanAsian25-34ProductDesigner-1-3Ys. P8FG3U.S.WomanAsian35-44DesignResearcher10K+<1Y P#FG#CountryGenderRaceAgeRoleOrg. Years P9FG4CanadaManWhite25-34PM/Editor/Researcher10K+3-5Ys. P10FG4CanadaManWhite35-44Researcher10K+3-5Ys. P11FG4U.S.WomanWhite25-34Researcher-<1Yr.	WorkplaceWellbeingTechnologies							Thetechnologyshouldhaveaclearandsensiblegoal. Provideclarityaboutdatagovernancepractices.Betransparentaboutwhohasaccesstoworkers'data. Showastrongrecordoftrustworthybehavior.Ensureoversightfromastrongpublicinterestorganization.Donotassumeconsentintheworkplace.Prioritizetheinterestsofworkersabovebusiness. Createnegotiationspacecenteringworkers'interests. Encouragedeliberationintheconsentprocesstopreserve workers'autonomy.Supportrecourseandenableworkerstointerrogatedatapractices.Enactregulationstoprotectworkers'rights&privacy. Informpeopleoftheirbasicworkplacerights.				Anothersetofchallengesthatemergedfromourfocusgroupsrelatedtotheinherent natureofpassivesensingtechnologies. Informationasymmetryaboutthetechnology.Technologymaynotsupportongoingconsent.Cannotsaynowithoutlosingaccesstothetechnology.Provideevidence&informationaboutthetechnology'sefficacy,limits,andrisks. Participantswereskepticalabouthow wellthetechnologyworked,wantingevidenceincludedintheconsentagreementsuchas"astudythatlookedthroughit,sayingthat forpeopleusingsimilartechnology,e.g.,depressionrateswent downorsomething,orcertainbehaviorsimproved"[P3].	UNDECLARED
StepStream: A School-Based Pervasive Social Fitness System for Everyday Adolescent Health - Proceedings of the SIGCHI Conference on Human Factors in Computing Systems	2014		Miller, Andrew D. and Mynatt, Elizabeth D.	10.1145/2556288.2557190	We present a new approach: school-based, pervasive social fitness systems. We describe one such system: StepStream, a pedometer-based microblog we designed and deployed for four weeks with 42 US middle school students.	Qualitative	USA	adolescent	Forty two (42) students participated in the study. Students were demographically representative of the school as a whole: 45% male (school: 53%), 55% female (school: 47%), 76% African American (school: 69%), 10% Hispanic (school: 9%), 14% Asian (school: 7%). The median age for participating students was 13, and most students were in the 6th and 7th grade. Although we didn't collect income information from the students themselves, 72% of students at the school qualify for free or reduced meals from the Title I program, a US federal meal assistance program targeted at children in low-income households. Based on self-report, students in the study are not significantly more overweight than national averages (about 1 in 3 American adolescents is overweight or obese).Thirteen students (31%) qualified as overweight (>1 standard	a school-based pervasive social fitness system for everyday adolescent health										We asked about peer social support in the focus groups and free-response section of the survey as well, and students shared a variety of ways this social support manifested. Students also reported feeling a greater sense of fitness expertise as a result of StepStream. It is also possible that some of the increase in students' interest and enthusiasm could be attributable to participating in a pedometer trial itself. However, the changes we did see were encouraging.		UNDECLARED
Understanding Sharing Preferences and Behavior for MHealth Devices - Proceedings of the 2012 ACM Workshop on Privacy in the Electronic Society	2012		Prasad, Aarathi and Sorber, Jacob and Stablein, Timothy and Anthony, Denise and Kotz, David	10.1145/2381966.2381983	We investigated users' willingness to share their personal information, collected using mHealth sensing devices, with their family, friends, third parties, and the public.	Mixed	USA	General public	Werecruited21undergraduate students,12adultworkersfromthelocalarea including Dartmouthemployees,and8femaleelderlyresidentsofa localretirementhome,asshowninTable1.It wasnot anaimofthestudytounderstandtheinfluenceofgender andoccupationonprivacyconcerns,sowedidnotfocus onthedistributionofparticipantsamongthesecategories.	mHealth devices											[illegible] lesswillingtosharethedemographicinformationwecollectedthantheactivityinformationthatwassensedbythe device.Informationsharedmorewithstrangersthan theirownfamilyandfriends. However,sharingscoresforpublicweremostlythesameor lessthanfriends.Participantswere morewillingtoshareiftheyperceivedbenefitsinsharing, especiallywhenitcametosharingwithspecificthirdparties, asopposedtothepublic. 3.Dynamicsharingbehavior.Weconfirmedthat privacyconcernsarenotstatic;mHealthdeviceusersmay changetheirsharingdecisionsovertime.	UNDECLARED
How Mass Surveillance Crowds Out Installations of COVID-19 Contact Tracing Applications	2023	Proc. ACM Hum.-Comput. Interact.	Toch, Eran and Ayalon, Oshrat	10.1145/3579491	We analyze the concurrent deployment of two contact tracing technologies in Israel: centralized mass surveillance technologies and a voluntary contact tracing mobile app.	Quantitative	Israel	General public	Themeanagewas38years(witha standarddeviationof13.80).Atotalof53%oftheparticipantswerewomen,46%weremen,andfew participantshadchosenthe"other"category.Religionorethnicbackgroundwasasfollows:Jews (82%),ArabMuslims(10.5%),ArabChristians(2%),andDruze(4%).Theseresultsareinlinewith representativesurveyscarriedoutinIsrael[19].Otherdemographicvariablesthatwererecorded includedreligiouslevel,education,andincome.Gender Age Religion Observance Education Income Male 239 18-19 10Jewish426 Secular 190Elementary3StBelowAve141 Female276 20-29 158Muslim55Traditional139Highschool 112 BelowAverage165 Other4 30-39 139 Druze 22 Religious 93 Non 138 Average138 40-49 81Christian16Orthodox57 Bachelor 184	COVID-19 ContactTracingApplications			Overall,peopleexpressednegativeattitudesregardingtheapp.Only28%willtend torecommendtheirfriendsandfamilyinstallit(versus43%thatwouldnot).Asomewhathigher proportionhaveapositiveattitudetowardsmakingitmandatoryforpeopleenteringmallsor publictransportation(35%versus43%).	Wefoundthat beliefinthesurveillanceutilitywasthestrongestandmostsignificantnegativefactorassociated withthelikelihoodofinstallingtheapplication(OR:0.547,95%CI0.372–0.803). Privacyconcernsaboutthecontacttracing applicationarealsonegativelycorrelatedwiththelikelihoodofinstallation(OR:0.758,95%CI 0.605-0.95). Wefoundthatsomeofthedemographicfactorswererelatedtoinstallation.Wefoundgender tobethemostrobustsignificantvariablewithaneffectoninstallations,withmenapproximately 100%morelikelytoinstalltheapplication(OR:2.04,95%CI1.215–3.429).Youngerpeoplearemore willingtoinstalltheapp,buttherelationshipisgenerallyweakandconcentratedonthe18-20year olddemographic.Peoplewithgraduatedegreesareapproximately100%timesmorelikelytoinstall theapplication(OR:2.034,95%CI0.984–4.20),butthelargeconfidenceintervalspointtoaweak effect.						Only27%thinkitwillreducetheirchancesofcontracting thecoronavirus,andonly32%believeitwillreducethespreadofthevirus.	Privacyconcernsare prevalent.59%feelthatitcollectssensitiveinformation(versus19%thatdisagree),and43%are worriedaboutprivacy (versus32%thatdisagree).	UNDECLARED
Persuasive Mobile Apps to Support Maternal and Child Healthcare and Help-Seeking Behaviors - 3rd African Human-Computer Interaction Conference: Inclusiveness and [illegible]			Samuel Nkwo, Makuochi and Orji, Rita and Ajah, Ifeyinwa and Igwe, Joseph and Ogbaga, Ignatius and [illegible]	10.1145/3448696.34[illegible]	We examined factors that lead to inappropriate maternal health-seeking behaviors amongst expectant and nursing mothers in developing nations. The findings from a large-scale user study of 600 participants from Africa uncover that factors such as cultural practices, religious beliefs, remoteness and inaccessibility and long waiting times at the health facility, ignorance and negative perception, continue to pose a big challenge to maternal and child health care.				weconductedaquantitativestudyofover600womenacross5Nigerianstates,including housewives,localmarketwomen,working mothers andolderwomenwhoareeitherpre[illegible]	Persuasive Mobile Apps to Support Maternal and Child Healthcare and [illegible]						InaccessibilityofHealthcare FacilitiesandLongWaitingPeriods Disapprovalbasedon culturalpracticesandreligiousbeliefs,and negativeperceptions. UnsatisfactoryServices [illegible]	RemoteandPersonalizedAccess MonitoringandSelf-guidedLearning SocialEngagement CollaborativeCare SupportforLowLiteracyWomen [illegible]					

Anyone Else Have This Experience: Sharing the Emotional Labor of Tracking Data About Me	2021	Proc. ACM Hum.-Comput. Interact.	Knittel, Megan and Kollig, Faye and Mason, Abrielle and Wash, Rick	10.1145/3449153	Our study investigates 1) how users seek out support online for emotional barriers elicited by their self-tracking data and 2) what self-described impact this sharing has on their self-tracking practices.	Qualitative	USA	Infertile patients	we analyzed discussions in two online communities on Reddit.com centered around infertility and trying to conceive that consistently describe self-tracking experiences.	Self-tracking technologies, ranging from digital thermometers to wearable fitness trackers, allow users to use personal data accumulated from their everyday activities.						It isimportant to recognize that the core, driving motivator of these community members is prob-ably not to create self-tracking data; rather, that self-tracking data becomes a stepping stonetowards becoming pregnant	[illegible] users thatdifferon variables of interest. Perhaps users could search for others whose personal data fall withincertain ranges or within certain contextual factors so they can connect with others based on thedegree of difference/similarity they are comfortable with or most interested in.Self-tracking systems not being flexible and individualized enough for all the kinds of data peoplethink is actually relevant to record is a common complaint [29,76,91]. Design suggestions tomake these technologies more personalized and customizable [76] have mostly focused on therelationship between the individual and the interface. However, our findings suggest that facilitatingties with other people in the interface could support this personalization work. If the actual tracking app let users describe a symptom(s)or feeling(s), share it with others, and then develop their own tracking metrics or variables basedalso on the details others have shared that resonate with them, users would benefit from from moreaccurate data recording and social support.				Next, the findings related to tensions surrounding hope demonstrate that the role of motivationand optimism is more complex than just serving as a positive predictor of sustained self-trackingengagement. These findings demonstrate that hope, can also increase users' distress and depression.The emotional repercussions of feeling hopeful makes hope something that is risky and always atrisk of collapse	UNDECLARED
Engaging with Health Data: The Interplay Between Self-Tracking Activities and Emotions in Fertility Struggles	2018	Proc. ACM Hum.-Comput. Interact.	Costa Figueiredo, Mayara and Caldeira, Clara and Eikey, Elizabeth Victoria and Mazmanian, Melissa and Chen, Yunan	10.1145/3274309	We explored how self-tracking activities and the emotional context characterize how people engage with personal health data through the analysis of a complex and emotionally-loaded use case: fertility self-tracking	Qualitative	USA	women	online health forums to collect data on the relationship between self-tracking and the emotional experience of infertility and fertility challenges.	fertility self-tracking.			Table 1. The different components of engagement with data Positive Burdened Obsessive Trapped Abandoning Emotion -Excited about tracking -Hope, plan, agency -Some stress/anxiety -Frustration -Increased focus -Some affect in relationship -Still mostly positive -Obsession -Full and selective trust in measures or blind hope -What is wrong with me? / Guilt -Reclusion Depression -Cannot handle it anymore Action -Learning to track -Seeing data -Becoming competent in understanding data -Increase tracking -Impact in schedule -Wants to try different measures -Wants to try different treatments -Obsessed with data -Manipulating measures -Measures take over other feelings -Dependent on tracking and data -Stop or take a break									UNDECLARED
Be Consistent, Work the Program, Be Present Every Day: Exploring Technologies for Self-Tracking in Early Recovery	2022	Proc. ACM Interact. Mob. Wearable Ubiquitous Technol.	Jones, Jasmine and Yuan, Ye and Yarosh, Svetlana	10.1145/3494955	Recovery from substance abuse disorders (SUDs) is a lifelong process of change. Self-tracking technologies have been proposed by the recovery community as a beneficial design space to support people adopting positive lifestyles and behaviors in their recovery. To explore the potential of this design space, we designed and deployed a technology probe consisting of a mobile app, wearable visualization, and ambient display to enable people to track and reflect on the activities they adopted in their recovery process. With this probe we conducted a four-week exploratory field study with 17 adults in early recovery to investigate 1) what activities people in recovery desire to track, 2) how people perceive self-tracking tools in relation to their recovery process, and 3) what digital resources self-tracking tools can provide to aid the recovery process.	Qualitative	USA	patients Recovery from substance abuse disorders (SUDs)	Participantsrangedinagefrom21-60(mean=34.6 yr,SD=9.58),andhadwidelyvaryingexperienceswithrecovery,rangingfromfirstattemptstothosemanaging years-longcyclesofabstinenceandrelapse.	Technologies for Self-Tracking in Early Recovery			Wefoundthatparticipant'sperceptionsofusefulnessforthetechnologiesin thestudyhingedontheinteractivityandtheuniquenessoffunctionordata:accessorydevicessuchaswearables orsecondarydatadisplaysmustprovideenoughuniqueutilitytoovercometheburdenofmaintenanceand justifytheirinclusionintoaperson'slimitedphysicalorpersonalspace							[illegible] urcesoneneedstobesuccessful.Peopleappreciated thevalidationandconvenienceofarecoveryfocusedsystem,envisioningwaysitcouldprovidenuancedsupport tohelpthemmaintaintheirsobrietyandimprovetheirqualityoflife.Themostsalientbenefitsidentifiedby participantsduringtheirmonth-longdeploymentstudywerethewaysthattheself-trackingpracticeandtools helpedthemtobemoremindfulanddeliberateintheiractivities,guidingtheirattentiontotheiraspirations andgoalsforthemselves.However,earlyrecoveryisatimeofsignificantchangeandvolatility.Thefeedbackofself-trackingtoolsmusthelppeoplebuildself-confidenceandself-efficacyratherthanmakingthemfeelpenalized orjudgedintheirachievements.		UNDECLARED
FutureSelf: What Happens When We Forecast Self-Trackers? Future Health Statuses? - Proceedings of the 2017 Conference on Designing Interactive Systems	2017		Rho, Saeyoung and Lee, Injung and Kim, Hankyung and Jung, Jonghyuk and Kim, Hyungi and Jun, Bong Gwan and Lim, Youn-kyung	10.1145/3064663.3064676	In this paper, we explore how future forecast information can be used in the design of self-tracking services.	Qualitative	Korea	General public	Sixmen and six women in their twenties were recruited from among 110 applicants and were divided into two groups with even distributions of age, gender, and BMI (Table 1). However, one participant dropped out due to lack of active participation; therefore, the final analysis included data for 11 participants. The average BMI was 22.3 in Group A (SD=1.62) and 22.4 in Group B (SD=1.81).	Participants used the FutureSelf app that we developed, which forecasts dieters' future weights and expected goal achievement rates based on their current behavior.										Findings from the 4-week study and follow-up interviewsrevealed that the forecasts of users' future weight based on their current behavior had unique impacts: it made participants imagine their future selves, reminded them of their ultimate goals, gave them hope that they could reach their goals, and motivated them to be more conscientious in making that future come true.		UNDECLARED
Data Engagement Reconsidered: A Study of Automatic Stress Tracking Technology in Use - Proceedings of the 2021 CHI Conference on Human Factors in Computing Systems	2021		Ding, Xianghua (Sharon) and Wei, Shuhan and Gui, Xinning and Gu, Ning and Zhang, Peng	10.1145/3411764.3445763	This paper presents an empirical study of automatic stress-tracking technologies in use in China, based on semi-structured interviews with 17 users.	Qualitative	China	General public	[illegible] malesand1female,largelyaligningwiththe maletofemaleratioofsmartwearabledevice market[85].Inaddition,mostareyoung,rangingfrom21to39yearsold.Theiroccupationsarediverse,andincludethoserelatedtoscienceandtechnology,suchasITmanufacturingtrainee,technicaldeveloper,programmer,wearablehealthworker,andex-ercisephysiologyworker,aswellasthosethatarenotITrelated,includingsalesman,governmentworker,andcustomerserviceem-ployee.Asshowninthetable,theywerefrom manydiferentcitiesinChina,rangingfrominlandcitiesinthenorth,suchasBeijing,Zhengzhou,Shenyang,andXi'an,aswellascoastal citiesinthesouth,suchasDongguan,ShenzhenandGuangzhou.Mostoftheparticipantsusedl Huawei'sWatchGTorHonorWatchMagic;P12andP17usedGarmin'sVivosmart4 andForerunner645Music,respectively.All hadusedthedevicesfrombetweenhalfamonthto12months.	AutomaticStressTrackingTechnologyinUse			Almostallofourinterviewparticipantshadalreadyadoptedandusedmostfrequently,howtheyusedtheseapplications,theirstressintegratedtheirsmartwearabledevicesintotheireverydaylives,status,theirperceivedstresssource(s),andhowtheydealwithsowedonothaveadoptionissuesasdiscussedinpreviousworksstressonadailybasis.Wethenaskedaboutdetailsoftheiruseof(e.g.[74])		OnlyafewWeconductedthematicanalysisinductively[9]withtheinter-couldmeaningfullyengagewithit,someonlyhadalimitedunder-viewdata.Wefirstfamiliarizedourselvesindividuallywiththestanding,whileotherswereconfused. Despitethatourparticipantsencounteredchallengeswhentryingtomakesenseofthestressdata,theydidnothaveeasyaccesstorelatedresourcesandcommunalsupporttohelpthemtacklethechallengestoachievemeaningfulinterpretations.	Allofourparticipantswereexcitedabouttheautomaticstress-trackingfeaturewhentheybeganusingtheirdevices.They checkedthedatafrequently.However,afteroneortwomonths,thenoveltyefectwasgone,andthemajority(exceptP12)stoppedengagingwiththedatainatimelyandfrequent manner Themainreasonforthiswasthatourparticipantsdidnotfeelthatthestress-trackingdeviceswerehelpfultoraisingtheirimme-diateawarenessduetotheirnaturalresponsestosomestressfuleventsandthelimitationsofthedevices Whensomeofthedataactuallydrewourparticipants'attentionandmotivatedthemtointerpretorrefectonit,mostofourpar-ticipants(exceptP12)founditchallengingtomakemeaningoutofit						UNDECLARED
The Role of Uncertainty as a Facilitator to Reflection in Self-Tracking - Proceedings of the 2020 ACM Designing Interactive Systems Conference	2020		Alqahtani, Deemah and Jay, Caroline and Vigo, Markel	10.1145/3357236.3395448	Self-trackers reflect on their personal data to understand their behaviour and plan accordingly. Often, this reflection involves uncertainty, which can affect decision-making. To better understand the role of uncertainty, we conducted an interview study to comprehend how uncertainty influences reflection and the resulting actions.	Qualitative	Unspecified	General public	We recruited sixteen participants (7 fe-male and 9 male,Meanage = 26.6,SD= 6.7) through snowballsampling and word-of-mouth.	Fitness trackers						Uncertaintycan be a motivator for users to log and reflect on their per-sonal data, even before engaging with tracking, Similarly, theuncertainty users have when trying to understand their activi-ties and routines is a strong motivator for self-tracking.						UNDECLARED
Self-E: Smartphone-Supported Guidance for Customizable Self-Experimentation - Proceedings of the 2021 CHI Conference on Human Factors in Computing Systems	2021		Daskalova, Nediyana and Kyi, Eindra and Ouyang, Kevin and Borem, Arthur and Chen, Sally and Park, Sung Hyun and Nugent, Nicole and Huang, Jeff	10.1145/3411764.3445100	To lower the barrier to intervention-based self-experimentation, we developed an app called Self-E, which guides users through the experiment. We conducted a 2-week diary study with 16 participants from the local population and a second study with a more advanced group of users to investigate how they perceive and carry out self-experiments with the help of Self-E, and what challenges they face.	Qualitative	USA	General public	Werecruited16participants,(P1–P16),4maleand12female,fromthelocalareabypostingflyersinpublicspacessuchascafesandsupermarketsandbypostingonlineinlocation-basedcommunitiessuchasRedditandNext Door.Participants'agesrangedfrom20to70 (M=33.7,SD=15.4),andtheirfamiliaritywithmathematicalstatisticsrangedfromnonetoexpertorprofessionallevels.	The ubiquity of self-tracking devices and smartphone apps has empowered people to collect data about themselves and try to self-improve.						Ourparticipantsreportedthattheirmostcommonuseofself-trackingdatawastomotivatethemselvestochangetheirbe-havior.Overall,participantssaidthatiftheyweretoconductaself-experiment,theirmaingoalwouldbetoidentifywhatgeneraladviceworksspecifcallyforthem	Acommontrend,whenfollowingtheinstructionswasnotpossible,wasforparticipantsto"dothebest[they]could,"mean-ingthattheysubstitutedtheexactbehaviorthattheappre-quiredwithsomethingmorefeasible Anoveltrendwenoticedwasthatparticipantswillfullydisre-gardedtheinstructionswhenaskedtofollowthebehaviortheyconsideredless healthy			Overall,twelveparticipants(75%)foundSelf-Eusefulfortheirself-experimentationneeds.	First,someparticipantsfeltthattheefectoftheintervention wastoonegligiblecomparedtotheefectsofotherthingsintheirdailylife(P15).Second,iftheresultscontradictedapreviouslyheldbelief,someparticipantsexpressedskepticismandfoundexcusesforwhytheresultswere'inaccurate.Third,assuggestedbypriorwork[18],thehighlikelihoodpercentageoritsdrasticfuctuationoverthecourse oftheex-perimentraisedsuspicion	UNDECLARED
Research on the Influencing Factors of User Trust Based on Artificial Intelligence Self Diagnosis System - Proceedings of the ACM Turing Celebration Conference - China	2020		Li, Weiheng and Fan, Xiangmin and Zhu, He and Wu, Jingzheng and Teng, Dongxing	10.1145/3393527.3393561	This paper focuses on the design of the user-friendly intelligent self diagnosis system, using interdisciplinary research methods.	Mixed	China	General public	No. Gender Age Nation Occupation Income Health 1 Male 61 Han Retired 1 3 2 Female 52 Han Painter 3 4 3 Male 49 Han Designer 3 5 4 Male 43 Han Seller 4 2 5 Male 38 Han Assistant 2 3 6 Female 33 Han Nurse 2 5 7 Male 21 Hui Athletes 2 5 8 Female 39 Han Civil servants 2 5 9 Male 37 Han Scientist 3 5 10 Male 52 Han Driver 1 2 11 Male 43 Han IT 5 5 12 Male 32 Han Scientist 3 3 13 Male 25 Zhuang Scientist 3 4	ArtificialIntelligenceSelfDiagnosis System	Volunteers generally lack theunderstandingof intelligent self-diagnosis system.		Evenso,mostofthemagreedthathealth chatbotswould be helpful topatients and expressed their willingnesstouseintelligentself-diagnosissystems. Tosumup,systemreliabilityinformation(thatis,modelaccuracy and prediction reliability) can improve users' trust and understandingof systemsuggestions,whiledetailsof system reasoningcanimprovecomprehensibility,butitisnothelpfulto improveusers'trust.	somefactorsleadingtousers distrustoftheintelligentselfdiagnosissystem.Althoughmostofthevolunteersexpressedtheir willingnesstouseintelligentself-diagnosissystem,theyremained skepticaloftheAIsystembecauseofthequality,reliabilityand accuracy. Inaddition,whetherAI-basedchatbotscancorrectly identifyrarediseasesisalsoabigchallenge.Participantssaidthe technologywasstillimmaturefromthepointofviewof providing rigorousmedicaldiagnosis.Especiallyforseriousillnesses, the volunteers' firstchoiceremainstogettothehospitalassoonas possibleandtalkbackandforthwiththedoctor.Butvolunteers agreethatAIsystemsmayprovidemorespecific,personalized informationandadvice.Somevolunteersbelievethatinthefuture AImedicalsystemswillbedesignedtobemoreaccurateand logical,but lackofhumanityandempathymaybehardforAI systems tosolve.								UNDECLARED
Wearable Identities: Understanding Wearables?Potential for Supporting the Expression of Queer Identities - Proceedings of the 2023 CHI Conference on Human Factors in Computing Systems	2023		Bolesnikov, Adrian and Cochrane, Karen Anne and Girouard, Audrey	10.1145/3544548.3581327	We conducted a two-phase qualitative study exploring queer expressive practices and wearable technologies through 16 semi-structured interviews and 15 body mapping workshops with the queer community.	Qualitative	Canada	Queer communities	ParticipantAgeGenderTrans(History)SexualityRace/EthnicitySessionLocationPI0123ManNoGayWhiteCanadaPI0234Non-BinaryYesQueerWhiteUSPI0336AgenderNoGay,QueerWhiteUKPI0437Non-Binary,GenderqueerNoPansexualWhiteUSPI0557ManNoGay,QueerWhiteUSPI0626Non-BinaryUnsureLesbianWhiteCanadaPI0720ManNoGayWhiteCandaPI0824WomanNoBisexualWhiteCanadaPI0924Trans-masc,Non-Binary,FluidYesBisexualWhiteCanadaPI1018WomanNoNolabel,Non-maleidentitiesWhite/PeruvianPeruPI1131WomanNoLesbianEast-Asian/IndianIndiaPI1218WomanNoBisexualWhiteCanadaPI1323GenderqueerYes/NoBisexualWhiteCanadaPI1431Non-BinaryUnsureQueerWhiteGermanyPI1523WomanNoGayWhiteCanadaPI1621Somethinglike"woman"NoBiromantic,AsexualWhite/PhilipinoCanadaTable1:PhaseO	wearable technologies through 16 semi-structured interviews and 15 body mapping workshops with the queer communit							P102statedthatincludingmultipleexpressiveoptionsforthingslikepronouns couldhelpinalleviatingbarrierssurroundingalackofpublicknowledge:"AllowingtheoptiontoputinwhatmostresonateswithuslikehavingexampIes[of pronouns]becausenoteveryoneknowswhattheyare."Sevenparticipantssharedthatthecustomizabilityoftheseoptionswerenecessaryforbeingabletoeasilychangedevicestoftpersonalidentities,desires,andlivedexperiences.PI13 said:"beingabletohaveopportunitiestomakethemyourownandexactlyhow youwanttobecausequeerexpressionissovaried."Fourparticipantssharedhowdevicehardware,throughcolourandshape,cansupportqueerexpression.PI02andPI08discussed thecolourofdevicesthatcommunicatediferentpenchantsforstereotypically masculineorfemininepreferences.ForPI02,theyutilizedcolourtocombatthesestereotypes:"I'vebeenexperiment-ingalittlebitmorewithcolours[ofmydevices]. . .Iwanttochallengetoxicmasculinity."Similarly,wefounddeviceshapeim-portantforqueerexpression.PI11sharedthattheircamerarefectstheirqueernessbecauseofitsutilitarianappearance:"It'sheavy,it'sbig,and,ifIhadtogenderit,itwoulddefnitelybemasculine."Weadditionallyobservedhardwaremodifcationsusingaccessorieslikestraps,stickers,andcases(n=9).PI06statedthatutilizingstick-ersisnotonlyasuccessfulwayofqueeringtheirtech,butalsofacilitatingsocial interaction:"I'mbigintostickers,Ihavealotofstickersonallmytech. . .Mytablethasawholebunchofqueerstickersthatoftengenerategreatconversationwithmycolleagueswhowantedtolearnmore."Additionally,nineparitici				Participantsemphasizedamultitudeofbarriersthatlimitaccesstowearableswithtwonoteworthy barriersincludinghighcostsandtechnologicalliteracy.Wealsofoundcriticalbarrierssurroundingbothphysicalanddatasafety.Additionally,participantsdiscussedbarriersattributedtoalackofqueeroptionsandinclusivity	UNDECLARED
Older Adults' Attitudes to Self-Management of Health and Wellness through Smart Home Data - Proceedings of the 9th International Conference on Pervasive Computing Technologies for Healthcare	2015		Doyle, Julie and Caprani, Niamh and Bond, Rodd		Our aims were to 1) examine attitudes to living with sensors and AAL technology over time; 2) gather opinions on the usefulness of this data for supporting self-management of health and wellbeing and 3) evaluate the effectiveness of various visualization techniques for presenting sensor-based health and wellness data.	Qualitative	Ireland	Older adults	Seven participants from GNH were interviewed, including 5 men and 2 women. When we perform interviews or focus groups, we ask a subset of the residents to take part, so as not to overburden the residents too much with the research. With such a long-term project, it is important ethically to ensure participants are not over-burdened. Thus, while all residents have the sensor and interactive technology in their homes, they are not required to take part in all research activities. Participant demographics are presented in Table I. Those interviewed ranged in age from 64 to 76 and had a wide range of health issues, including diabetes, heart conditions, chronic back pain and limited mobility. All take multiple medications. However, all participants are fully functioning and living independently	Older adults' attitudes to self-management of health and wellness through smart home data	all were aware that energy was being monitored, as well as temperature and doors and windows being open or closed. In terms of the PIR sensors, 6 of the 7 participants understood that the sensors monitor movement. One participant thought they were also 'counting'				As outlined in Table III, 5 of the 7 participants felt that the sensors were unobtrusive, stating that they rarely noticed them anymore. The red light from the PIR sensor was one reason provided for the sensors being somewhat obtrusive.		This suggests further information is needed to support user understanding e.g. through text, colour, icons. Therefore we believe a weekly visualisation would be more appropriate.The participants were presented with static visualisations, however one of the participants claimed that he would be interested in interacting with the graphs to explore more details about his data			Sensors and Health We began section 2 of the interview by asking participants if there was any particular information they would like to learn from the sensors regarding their health. Participants found it difficult to answer this question, until we began to use the visualisations as probes. The first topic of discussion centred around patterns. All participants felt having a regular pattern or routine was important, and reported typically going to bed, waking up at the same time each day, for example. Our data research over the years at GNH has demonstrated that this is the case with the majority of residents. The visualisations, for example the clock plot (Figure 2), were used to discuss how patterns might change, even subtly, over time and participants reported finding this information in general quite useful. P11 said, "I think seeing a pattern is very important. I'm talking about being able to observe and being able to know when you can seek professional advice, in different situations. I certainly think we need to know that. I need to know myself." Some participants said that if their patterns were changing, they would want to know why. P20 said,	Further questions asked participants about their experience of living with the technology over the years, including anybenefits or drawbacks they perceived. Safety and security were mentioned by each participant, with some stating the importance of these for emotional wellbeing. P20 said, "Before I came here I used to suffer about 4 panic attacks a year. I haven't had one panic attack since I moved here, my wellbeing here is 100%."Prior to moving to GNH, only 2 participants had previously used a computer and the Internet (Table I) and in our early interviews, many initially expressed an uneasiness regarding physically interacting with technology. However, all 7 participants reported actively using their iPads, includingapplications that had been custom-built for their homes as well as various other applications. The iPads appear to have been instrumental in participants' change of attitude towards technology. P5 said,"Ah since we got the	UNDECLARED
Design for the Elderly: The Acceptance of Smart Vests in the Senior Population - Proceedings of the 6th International Conference on Advances in Artificial Intelligence	2023		Xie, Zhuozhen	10.1145/3571560.3571580	In order to encourage the elderly to use and wear smart vests, this study investigated the factors affecting the acceptance of smart vests; moreover, it developed the technology acceptance model of smart vests for the elderly population.	Quantitative	China	older adults	The data show that most respondents are between the age of 60sto 70s, a small part of interviewees are from their 70s to 90s, andhardly anybody is over 90s. Furthermore, more than half of therespondents did not have a college education background, and therewere very few respondents with postgraduate backgrounds	Smart Vests			able 2 shows that most older adults are very interested insmart vests and want to know more about them, which positivelypromotes older adults to realize the perceived usefulness of smart vests.	Figure 3 shows that perceived usefulness greatly influencesthe attitude to use of the elderly use smart vests, which implies thatmost of the elderly are dependent on perceived usefulness. (1) The combination of aesthetic perception and TAM predictsuser acceptance of the smart vest; further, aesthetic perception hasa significant indirect impact on the actual use of the smart vest.(2) Aesthetics perception, as an external factor, has a significantdirect impact on perceived usefulness and perceived ease of use.(3) Perceived usefulness has a direct impact on attitude and anindirect effect on actual use.(4) Perceived ease of use does not directly influence the attitudeto use but indirectly affects the attitude to use through perceivedusefulness.								UNDECLARED
Mood Self-Assessment on Smartphones - Proceedings of the Conference on Wireless Health	2015		Khue, Le Minh and Ouh, Eng Lieh and Jarzabek, Stan	10.1145/2811780.2811921	We developed mobile versions of validated pen-and-paper scales for mood self-assessment to facilitate accurate in-situ mood self-assessment in real-life situations by smartphone users.	Quantitative	Singapore	General public	We asked 40 students to freely play with our app and provide their feedback The survey involves participants only in the age range above 18 with no involvement of children or young people and the ratio of males over females is estimated at 60% to 40%.	Mood Self-Assessment on Smartphones			a higher proportion (45%) of participants preferred AffectButton as the method to record the mood. However, in terms of accuracy of mood description, only 30% of participants thought that it was the best method to use. 73% of participants agreed that photos accompanying mood items in the scales helped them better relate to their mood.		Participants found photos helpful in relating them to their mood, as shown in comparison of SPANE with and without photos (Figure 9). SAM and AffectButton received highest ratings in terms of engagement and ease of use.		[illegible] the evaluation criteria. Furthermore, they favored human-like figure over abstract icon-manikin used in original SAM. Most of the participants favored male figure independently of their own gender. Viewing past mood data, social connectivity and help functions were found engaging, however there is room to improve ease of use of apps. 83% of participants preferred mobile scales to pen-and-paper scales with SAM as the overall preferred mobile mood scale as shown in Figure 11 and Figure 12. 45% of participants indicated gender of icons in mood scales to be of considerable importance in helping them relate to their mood. In most comments we read that participants preferred icons of their own gender. A potential alternative is to use phone vibration as an indicator of intensity associated with a given mood item: Weak vibration corresponding to low intensity and increasingly strong vibration corresponding higher intensity. We received an interesting feedback suggesting that the color scheme of the themes should be carefully selected since it may affect the current mood of user at the time of self-reporting and cause her to lose her objective judgment.	The post-survey discussion with participants revealed this misunderstanding and we had to discard part of the survey related to utility and engagement that was affected by the incorrect assumption. We realized that we failed provide participants with detailed enough protocol how to use our app during evaluation				UNDECLARED
Irish Attitudes Toward COVID Tracker App & Privacy: Sentiment Analysis on Twitter and Survey Data	2021	Proceedings of the 16th International Conference on Availability, Reliability and Security	Pintu Lohar and Guodong Xie and Malika Bendechache and Rob Brennan and Edoardo Celeste and Ramona Trestian and Irina Tal	10.1145/3465481.3469193	Contact tracing apps used in tracing and mitigating the spread of COVID-19 have sparked discussions and controversies worldwide. The major concerns in relation to these apps are around privacy.For this purpose we have conducted sentiment analysis on Twitter data collected from public Twitter accounts from Republic of Ireland.	Quantitative	Ireland	General public	We collected COVID-19related tweets generated in Ireland over a period of time from Janu-ary 1, 2020 up to December 31, 2020 in order to perform sentimentanalysis on this data set	COVID Tracker App &			It can be seen in the figure that only a13.88%ofthe tweets are classified as positive with more than a half (54.7%)being classified as negative.		The issueof battery consumption was a well-known issue of the HSE trackerapp that was fixed in the meantime.					he tweetsreveal privacy concerns, but other issues that relates to batteryconsumption, performance and efficacy.	[illegible] trackerapp are around privacy, for example:I understand the benefits of in-formation that privacy violating trackers can have in helping with theCovid crisis. However, it also carries a risk of enabling more unethicalprivacy violating data collection activities to be more "tolerated" and"seen as acceptable" after the Covid crisis has ceased.	UNDECLARED
